# Population-level risks of alcohol consumption by amount, geography, age, sex, and year: a systematic analysis for the Global Burden of Disease Study 2020

**DOI:** 10.1016/S0140-6736(22)00847-9

**Published:** 2022-07-16

**Authors:** GBD 2020 Alcohol Collaborators, Dana Bryazka, Marissa B Reitsma, Max G Griswold, Kalkidan Hassen Abate, Cristiana Abbafati, Mohsen Abbasi-Kangevari, Zeinab Abbasi-Kangevari, Amir Abdoli, Mohammad Abdollahi, Abu Yousuf Md Abdullah, E S Abhilash, Eman Abu-Gharbieh, Juan Manuel Acuna, Giovanni Addolorato, Oladimeji M Adebayo, Victor Adekanmbi, Kishor Adhikari, Sangeet Adhikari, Qorinah Estiningtyas Sakilah Adnani, Saira Afzal, Wubetu Yimam Agegnehu, Manik Aggarwal, Bright Opoku Ahinkorah, Araz Ramazan Ahmad, Sajjad Ahmad, Tauseef Ahmad, Ali Ahmadi, Sepideh Ahmadi, Haroon Ahmed, Tarik Ahmed Rashid, Chisom Joyqueenet Akunna, Hanadi Al Hamad, Md Zakiul Alam, Dejene Tsegaye Alem, Kefyalew Addis Alene, Yousef Alimohamadi, Atiyeh Alizadeh, Kasim Allel, Jordi Alonso, Saba Alvand, Nelson Alvis-Guzman, Firehiwot Amare, Edward Kwabena Ameyaw, Sohrab Amiri, Robert Ancuceanu, Jason A Anderson, Catalina Liliana Andrei, Tudorel Andrei, Jalal Arabloo, Muhammad Arshad, Anton A Artamonov, Zahra Aryan, Malke Asaad, Mulusew A Asemahagn, Thomas Astell-Burt, Seyyed Shamsadin Athari, Desta Debalkie Atnafu, Prince Atorkey, Alok Atreya, Floriane Ausloos, Marcel Ausloos, Getinet Ayano, Martin Amogre ayanore Ayanore, Olatunde O Ayinde, Jose L Ayuso-Mateos, Sina Azadnajafabad, Melkalem Mamuye Azanaw, Mohammadreza Azangou-Khyavy, Amirhossein Azari Jafari, Ahmed Y Azzam, Ashish D Badiye, Nasser Bagheri, Sara Bagherieh, Mohan Bairwa, Shankar M Bakkannavar, Ravleen Kaur Bakshi, Awraris Hailu Balchut/Bilchut, Till Winfried Bärnighausen, Fabio Barra, Amadou Barrow, Pritish Baskaran, Luis Belo, Derrick A Bennett, Isabela M Benseñor, Akshaya Srikanth Bhagavathula, Neeraj Bhala, Ashish Bhalla, Nikha Bhardwaj, Pankaj Bhardwaj, Sonu Bhaskar, Krittika Bhattacharyya, Vijayalakshmi S Bhojaraja, Bagas Suryo Bintoro, Elena A Elena Blokhina, Belay Boda Abule Bodicha, Archith Boloor, Cristina Bosetti, Dejana Braithwaite, Hermann Brenner, Nikolay Ivanovich Briko, Andre R Brunoni, Zahid A Butt, Chao Cao, Yin Cao, Rosario Cárdenas, Andre F Carvalho, Márcia Carvalho, Joao Mauricio Castaldelli-Maia, Giulio Castelpietra, Luis F S Castro-de-Araujo, Maria Sofia Cattaruzza, Promit Ananyo Chakraborty, Jaykaran Charan, Vijay Kumar Chattu, Akhilanand Chaurasia, Nicolas Cherbuin, Dinh-Toi Chu, Nandita Chudal, Sheng-Chia Chung, Chuchu Churko, Liliana G Ciobanu, Massimo Cirillo, Rafael M Claro, Simona Costanzo, Richard G Cowden, Michael H Criqui, Natália Cruz-Martins, Garland T Culbreth, Berihun Assefa Dachew, Omid Dadras, Xiaochen Dai, Giovanni Damiani, Lalit Dandona, Rakhi Dandona, Beniam Darge Daniel, Anna Danielewicz, Jiregna Darega Gela, Kairat Davletov, Jacyra Azevedo Paiva de Araujo, Antonio Reis de Sá-Junior, Sisay Abebe Debela, Azizallah Dehghan, Andreas K Demetriades, Meseret Derbew Molla, Rupak Desai, Abebaw Alemayehu Desta, Diana Dias da Silva, Daniel Diaz, Lankamo Ena Digesa, Mengistie Diress, Milad Dodangeh, Deepa Dongarwar, Fariba Dorostkar, Haneil Larson Dsouza, Bereket Duko, Bruce B Duncan, Kristina Edvardsson, Michael Ekholuenetale, Frank J Elgar, Muhammed Elhadi, Mohamed A Elmonem, Aman Yesuf Endries, Sharareh Eskandarieh, Azin Etemadimanesh, Adeniyi Francis Fagbamigbe, Ildar Ravisovich Fakhradiyev, Fatemeh Farahmand, Carla Sofia e Sá Farinha, Andre Faro, Farshad Farzadfar, Ali Fatehizadeh, Nelsensius Klau Fauk, Valery L Feigin, Rachel Feldman, Xiaoqi Feng, Zinabu Fentaw, Simone Ferrero, Lorenzo Ferro Desideri, Irina Filip, Florian Fischer, Joel Msafiri Francis, Richard Charles Franklin, Peter Andras Gaal, Mohamed M Gad, Silvano Gallus, Fabio Galvano, Balasankar Ganesan, Tushar Garg, Mesfin Gebrehiwot Damtew Gebrehiwot, Teferi Gebru Gebremeskel, Mathewos Alemu Gebremichael, Tadele Regasa Gemechu, Lemma Getacher, Motuma Erena Getachew, Abera Getachew Obsa, Asmare Getie, Amir Ghaderi, Mansour Ghafourifard, Alireza Ghajar, Seyyed-Hadi Ghamari, Lilian A Ghandour, Mohammad Ghasemi Nour, Ahmad Ghashghaee, Sherief Ghozy, Franklin N Glozah, Ekaterina Vladimirovna Glushkova, Justyna Godos, Amit Goel, Salime Goharinezhad, Mahaveer Golechha, Pouya Goleij, Mohamad Golitaleb, Felix Greaves, Michal Grivna, Giuseppe Grosso, Temesgen Worku Gudayu, Bhawna Gupta, Rajeev Gupta, Sapna Gupta, Veer Bala Gupta, Vivek Kumar Gupta, Nima Hafezi-Nejad, Arvin Haj-Mirzaian, Brian J Hall, Rabih Halwani, Tiilahun Beyene Handiso, Graeme J Hankey, Sanam Hariri, Josep Maria Haro, Ahmed I Hasaballah, Hossein Hassanian-Moghaddam, Simon I Hay, Khezar Hayat, Golnaz Heidari, Mohammad Heidari, Delia Hendrie, Claudiu Herteliu, Demisu Zenbaba Heyi, Kamal Hezam, Mbuzeleni Mbuzeleni Hlongwa, Ramesh Holla, Md Mahbub Hossain, Sahadat Hossain, Seyed Kianoosh Hosseini, Mehdi hosseinzadeh, Mihaela Hostiuc, Sorin Hostiuc, Guoqing Hu, Junjie Huang, Salman Hussain, Segun Emmanuel Ibitoye, Irena M Ilic, Milena D Ilic, Mustapha Immurana, Lalu Muhammad Irham, M Mofizul Islam, Rakibul M Islam, Sheikh Mohammed Shariful Islam, Hiroyasu Iso, Ramaiah Itumalla, Masao Iwagami, Roxana Jabbarinejad, Louis Jacob, Mihajlo Jakovljevic, Zahra Jamalpoor, Elham Jamshidi, Sathish Kumar Jayapal, Umesh Umesh Jayarajah, Ranil Jayawardena, Rime Jebai, Seyed Ali Jeddi, Alelign Tasew Jema, Ravi Prakash Jha, Har Ashish Jindal, Jost B Jonas, Tamas Joo, Nitin Joseph, Farahnaz Joukar, Jacek Jerzy Jozwiak, Mikk Jürisson, Ali Kabir, Robel Hussen Kabthymer, Bhushan Dattatray Kamble, Himal Kandel, Girum Gebremeskel Kanno, Neeti Kapoor, Ibraheem M Karaye, Salah Eddin Karimi, Bekalu Getnet Kassa, Rimple Jeet Kaur, Gbenga A Kayode, Mohammad Keykhaei, Himanshu Khajuria, Rovshan Khalilov, Imteyaz A Khan, Moien AB Khan, Hanna Kim, Jihee Kim, Min Seo Kim, Ruth W Kimokoti, Mika Kivimäki, Vitalii Klymchuk, Ann Kristin Skrindo Knudsen, Ali-Asghar Kolahi, Vladimir Andreevich Korshunov, Ai Koyanagi, Kewal Krishan, Yuvaraj Krishnamoorthy, G Anil Kumar, Narinder Kumar, Nithin Kumar, Ben Lacey, Tea Lallukka, Savita Lasrado, Jerrald Lau, Sang-woong Lee, Wei-Chen Lee, Yo Han Lee, Lee-Ling Lim, Stephen S Lim, Stany W Lobo, Platon D Lopukhov, Stefan Lorkowski, Rafael Lozano, Giancarlo Lucchetti, Farzan Madadizadeh, Áurea M Madureira-Carvalho, Soleiman Mahjoub, Ata Mahmoodpoor, Rashidul Alam Mahumud, Alaa Makki, Mohammad-Reza Malekpour, Narayana Manjunatha, Borhan Mansouri, Mohammad Ali Mansournia, Jose Martinez-Raga, Francisco A Martinez-Villa, Richard Matzopoulos, Pallab K Maulik, Mahsa Mayeli, John J McGrath, Jitendra Kumar Meena, Entezar Mehrabi Nasab, Ritesh G Menezes, Gert B M Mensink, Alexios-Fotios A Mentis, Atte Meretoja, Bedasa Taye Merga, Tomislav Mestrovic, Junmei Miao Jonasson, Bartosz Miazgowski, Ana Carolina Micheletti Gomide Nogueira de Sá, Ted R Miller, GK Mini, Andreea Mirica, Antonio Mirijello, Seyyedmohammadsadeq Mirmoeeni, Erkin M Mirrakhimov, Sanjeev Misra, Babak Moazen, Maryam Mobarakabadi, Marcello Moccia, Yousef Mohammad, Esmaeil Mohammadi, Abdollah Mohammadian-Hafshejani, Teroj Abdulrahman Mohammed, Nagabhishek Moka, Ali H Mokdad, Sara Momtazmanesh, Yousef Moradi, Ebrahim Mostafavi, Sumaira Mubarik, Erin C Mullany, Beemnet Tekabe Mulugeta, Efrén Murillo-Zamora, Christopher J L Murray, Julius C Mwita, Mohsen Naghavi, Mukhammad David Naimzada, Vinay Nangia, Biswa Prakash Nayak, Ionut Negoi, Ruxandra Irina Negoi, Seyed Aria Nejadghaderi, Samata Nepal, Sudan Prasad Prasad Neupane, Sandhya Neupane Kandel, Yeshambel T Nigatu, Ali Nowroozi, Khan M Nuruzzaman, Chimezie Igwegbe Nzoputam, Kehinde O Obamiro, Felix Akpojene Ogbo, Ayodipupo Sikiru Oguntade, Hassan Okati-Aliabad, Babayemi Oluwaseun Olakunde, Gláucia Maria Moraes Oliveira, Ahmed Omar Bali, Emad Omer, Doris V Ortega-Altamirano, Adrian Otoiu, Stanislav S Otstavnov, Bilcha Oumer, Mahesh P A, Alicia Padron-Monedero, Raffaele Palladino, Adrian Pana, Songhomitra Panda-Jonas, Anamika Pandey, Ashok Pandey, Shahina Pardhan, Tarang Parekh, Eun-Kee Park, Charles D H Parry, Fatemeh Pashazadeh Kan, Jay Patel, Siddhartha Pati, George C Patton, Uttam Paudel, Shrikant Pawar, Amy E Peden, Ionela-Roxana Petcu, Michael R Phillips, Marina Pinheiro, Evgenii Plotnikov, Pranil Man Singh Pradhan, Akila Prashant, Jianchao Quan, Amir Radfar, Alireza Rafiei, Pankaja Raghav Raghav, Vafa Rahimi-Movaghar, Azizur Rahman, Md Mosfequr Rahman, Mosiur Rahman, Amir Masoud Rahmani, Shayan Rahmani, Chhabi Lal Ranabhat, Priyanga Ranasinghe, Chythra R Rao, Drona Prakash Rasali, Mohammad-Mahdi Rashidi, Zubair Ahmed Ratan, David Laith Rawaf, Salman Rawaf, Lal Rawal, Andre M N Renzaho, Negar Rezaei, Saeid Rezaei, Mohsen Rezaeian, Seyed Mohammad Riahi, Esperanza Romero-Rodríguez, Gregory A Roth, Godfrey M Rwegerera, Basema Saddik, Erfan Sadeghi, Reihaneh Sadeghian, Umar Saeed, Farhad Saeedi, Rajesh Sagar, Amirhossein Sahebkar, Harihar Sahoo, Mohammad Ali Sahraian, KM Saif-Ur-Rahman, Sarvenaz Salahi, Hamideh Salimzadeh, Abdallah M Samy, Francesco Sanmarchi, Milena M Santric-Milicevic, Yaser Sarikhani, Brijesh Sathian, Ganesh Kumar Saya, Mehdi Sayyah, Maria Inês Schmidt, Aletta Elisabeth Schutte, Michaël Schwarzinger, David C Schwebel, Abdul-Aziz Seidu, Nachimuthu Senthil Kumar, SeyedAhmad SeyedAlinaghi, Allen Seylani, Feng Sha, Sarvenaz Shahin, Fariba Shahraki-Sanavi, Shayan Shahrokhi, Masood Ali Shaikh, Elaheh Shaker, Murad Ziyaudinovich Shakhmardanov, Mehran Shams-Beyranvand, Sara Sheikhbahaei, Rahim Ali Sheikhi, Adithi Shetty, Jeevan K Shetty, Damtew Solomon Shiferaw, Mika Shigematsu, Rahman Shiri, Reza Shirkoohi, K M Shivakumar, Velizar Shivarov, Parnian Shobeiri, Roman Shrestha, Negussie Boti Sidemo, Inga Dora Sigfusdottir, Diego Augusto Santos Silva, Natacha Torres da Silva, Jasvinder A Singh, Surjit Singh, Valentin Yurievich Skryabin, Anna Aleksandrovna Skryabina, David A Sleet, Marco Solmi, YONATAN SOLOMON, Suhang Song, Yimeng Song, Reed J D Sorensen, Sergey Soshnikov, Ireneous N Soyiri, Dan J Stein, Sonu Hangma Subba, Miklós Szócska, Rafael Tabarés-Seisdedos, Takahiro Tabuchi, Majid Taheri, Ker-Kan Tan, Minale Tareke, Elvis Enowbeyang Tarkang, Gebremaryam Temesgen, Worku Animaw Temesgen, Mohamad-Hani Temsah, Kavumpurathu Raman Thankappan, Rekha Thapar, Nikhil Kenny Thomas, Chalachew Tiruneh, Jovana Todorovic, Marco Torrado, Mathilde Touvier, Marcos Roberto Tovani-Palone, Mai Thi Ngoc Tran, Sergi Trias-Llimós, Jaya Prasad Tripathy, Alireza Vakilian, Rohollah Valizadeh, Mehdi Varmaghani, Shoban Babu Varthya, Tommi Juhani Vasankari, Theo Vos, Birhanu Wagaye, Yasir Waheed, Mandaras Tariku Walde, Cong Wang, Yanzhong Wang, Yuan-Pang Wang, Ronny Westerman, Nuwan Darshana Wickramasinghe, Abate Dargie Wubetu, Suowen Xu, Kazumasa Yamagishi, Lin Yang, Gesila Endashaw E Yesera, Arzu Yigit, Vahit Yiğit, Ayenew Engida Ayenew Engida Yimaw, Dong Keon Yon, Naohiro Yonemoto, Chuanhua Yu, Siddhesh Zadey, Mazyar Zahir, Iman Zare, Mikhail Sergeevich Zastrozhin, Anasthasia Zastrozhina, Zhi-Jiang Zhang, Chenwen Zhong, Mohammad Zmaili, Yves Miel H Zuniga, Emmanuela Gakidou

## Abstract

**Background:**

The health risks associated with moderate alcohol consumption continue to be debated. Small amounts of alcohol might lower the risk of some health outcomes but increase the risk of others, suggesting that the overall risk depends, in part, on background disease rates, which vary by region, age, sex, and year.

**Methods:**

For this analysis, we constructed burden-weighted dose–response relative risk curves across 22 health outcomes to estimate the theoretical minimum risk exposure level (TMREL) and non-drinker equivalence (NDE), the consumption level at which the health risk is equivalent to that of a non-drinker, using disease rates from the Global Burden of Diseases, Injuries, and Risk Factors Study (GBD) 2020 for 21 regions, including 204 countries and territories, by 5-year age group, sex, and year for individuals aged 15–95 years and older from 1990 to 2020. Based on the NDE, we quantified the population consuming harmful amounts of alcohol.

**Findings:**

The burden-weighted relative risk curves for alcohol use varied by region and age. Among individuals aged 15–39 years in 2020, the TMREL varied between 0 (95% uncertainty interval 0–0) and 0·603 (0·400–1·00) standard drinks per day, and the NDE varied between 0·002 (0–0) and 1·75 (0·698–4·30) standard drinks per day. Among individuals aged 40 years and older, the burden-weighted relative risk curve was J-shaped for all regions, with a 2020 TMREL that ranged from 0·114 (0–0·403) to 1·87 (0·500–3·30) standard drinks per day and an NDE that ranged between 0·193 (0–0·900) and 6·94 (3·40–8·30) standard drinks per day. Among individuals consuming harmful amounts of alcohol in 2020, 59·1% (54·3–65·4) were aged 15–39 years and 76·9% (73·0–81·3) were male.

**Interpretation:**

There is strong evidence to support recommendations on alcohol consumption varying by age and location. Stronger interventions, particularly those tailored towards younger individuals, are needed to reduce the substantial global health loss attributable to alcohol.

**Funding:**

Bill & Melinda Gates Foundation.

## Introduction

Alcohol use accounted for 1·78 million (95% uncertainty interval [UI] 1·39–2·27) deaths in 2020 and was the leading risk factor for mortality among males aged 15–49 years (Bryazka D, unpublished). The relationship between moderate alcohol use and health is complex, as shown in multiple previous studies.^1^, ^2^, ^3^, ^4^, ^5^, ^6^ Alcohol consumption at any level is associated with health loss from several diseases, including liver cirrhosis, breast cancer, and tuberculosis, as well as injuries.^7^, ^8^, ^9^, ^10^ At the same time, some studies have found that consumption of small amounts of alcohol lowers the risk of cardiovascular diseases and type 2 diabetes.^11^, ^12^, ^13^ As a corollary, the amount of alcohol that minimises health loss is likely to depend on the distribution of underlying causes of disease burden in a given population. Since this distribution varies widely by geography, age, sex, and time, the level of alcohol consumption associated with the lowest risk to health would depend on the age structure and disease composition of that population.^14^, ^15^, ^16^

Two quantities are crucially relevant when formulating effective, evidence-based guidelines and alcohol-control policies: the theoretical minimum risk exposure level (TMREL), which represents the level of consumption that minimises health loss from alcohol for a population, and the non-drinker equivalence (NDE) level, which measures the level of alcohol consumption at which the risk of health loss for a drinker is equivalent to that of a non-drinker. The majority of studies to date consider one or a small subset of health outcomes associated with alcohol consumption at a time, although several broader systematic meta-analyses have been done.^1^, ^4^, ^17^, ^18^, ^19^ Findings from these studies vary in their estimates of the TMREL. Several studies have found evidence of a J-shaped relationship between alcohol use and all-cause mortality.^3^, ^18^, ^20^ However, others have reported that the all-cause or attributable cause burden weighted TMREL of alcohol is zero standard drinks per day.^1^, ^21^ Uncertainty about the effect of alcohol on all-cause health loss results from differences in the relative disease composition between studies, conflicting studies on individual health outcomes, differences in study covariates and methods, estimation of drinking patterns, as well as issues relating to selection bias.^22^, ^23^

**Research in context**

**Evidence before this study**

The risks of moderate alcohol use on health outcomes have been widely studied and debated for many years. Studies have considered the health impacts associated with alcohol consumption through a variety of approaches, ranging from exploring the effects on a single disease, to considering multiple health outcomes, to using all-cause mortality as an outcome. Several systematic reviews have also been published on this topic, and in recent years several publications have used Mendelian randomisation to explore the association between alcohol use and health outcomes. Overall, the findings have varied, which partly contributes to this topic being controversial and a subject of debate. Several studies have found evidence of a J-shaped relationship between alcohol use and all-cause mortality or burden; in other words, at low levels of consumption, alcohol lowers the risk of all-cause mortality, whereas above some threshold it increases the risk. However, other studies, including a publication by the GBD 2016 Alcohol Collaborators in *The Lancet* in 2018, have reported that the level of alcohol consumption that minimises health loss is zero standard drinks per day. The apparent contradiction in findings across existing studies highlights the significance of continuing to study this topic and updating the evidence base as more information becomes available. Importantly, few previous studies analysing the effects of alcohol consumption on all-cause mortality have considered how the relationship between alcohol use and health is contingent on background rates of disease. We did a systematic review of the literature in which we searched PubMed and previous published meta-analyses using search terms such as “alcohol” and “drinking behavior”, terms concerning study outcomes such as “risk”, “odds ratio”, and “hazard ratio”, and terms concerning the specific causes included in the study, such as “ischemic heart disease” or “tuberculosis”. We searched for studies published up to Dec 31, 2019; the search was limited to English language publications.

**Added value of this study**

In this systematic analysis for the Global Burden of Diseases, Injuries, and Risk Factors Study (GBD) 2020, we estimated levels of alcohol consumption that minimise health loss using updated systematic reviews and meta-regressions, building on results from GBD 2016 and incorporating region-specific background rates of diseases and injuries within our assessment. To the best of our knowledge, this is the first study to consider the implications of background rates of disease on levels of alcohol consumption that minimise health loss. We updated the previously published systematic review and meta-regressions to consider all published studies through to December, 2019, reporting on the association between alcohol and the six alcohol-attributable health outcomes accounting for the highest number of global disability-adjusted life-years. We found insufficient evidence for an association between alcohol use and one of these outcomes and subsequently omitted it from further analysis. This analysis has yielded updated relationships on the relative risk of mortality for five causes, at various levels of alcohol consumption, which we combined with relative risk estimates from GBD 2016 for an additional 17 outcomes. We used this information, along with information on the burden of disease from these 22 diseases and injuries, to estimate the level of alcohol consumption that minimises health loss separately for each age group, sex, year, and region. In addition to estimating the level of consumption that is associated with minimising health loss, known as the theoretical minimum risk exposure level (TMREL), we also estimated the level of alcohol consumption at which the risk to health for a drinker is equivalent to that of a non-drinker—a quantity we refer to as the non-drinker equivalence.

**Implications of all the available evidence**

Our results are consistent with previous findings at the global level, and at the same time the more nuanced analysis done in this study strongly suggests that statements, guidelines, and recommendations on the optimal level of alcohol consumption need to take into consideration the background rates of diseases and injuries for each population. We provide clear evidence that the level of alcohol consumption that minimises health loss varies significantly across populations and remains zero or very close to zero for several population groups, particularly young adults. At the same time, small amounts of alcohol consumption are associated with improved health outcomes in populations that predominantly face a high burden of cardiovascular diseases, particularly older adults in many world regions. Given these findings, we recommend a modification of existing policy guidelines to focus on emphasising differential optimal consumption levels by age, rather than the current practice of recommending different consumption levels by sex. This study highlights the importance of prioritising interventions targeted at minimising alcohol consumption among young adults.

Importantly, no study to date has examined the variation in the theoretical minimum risk of alcohol consumption by geography, age, sex, and time, conditioned on background rates of disease. National dietary guidelines on low-risk drinking, such as those in the USA, UK, France, and Australia, base recommendations on studies of the risk of alcohol use on all-cause mortality and some cause-specific outcomes.^24^, ^25^, ^26^, ^27^ This complicates interpretation of the risk of alcohol use on mortality, given three aspects of all-cause mortality. First, causal pathways between alcohol use and cause-specific outcomes can differ, creating multiple confounding structures that are not readily adjustable when embedded within models analysing the effects of alcohol use on all-cause mortality.^28^ Second, all-cause mortality includes non-causally related outcomes, further increasing the threat to internal validity for evidence produced from analysing the effects of alcohol use on all-cause mortality. Third, and most importantly for the present study, the composition of causes within all-cause mortality can differ substantially between populations, changing the proportional risk of mortality due to alcohol use across these populations.^1^, ^29^ In tandem, these features limit the applicability of determining minimum risk exposures on the basis of observational data on alcohol use and all-cause mortality.

In this study, we used the distribution of causes of disability-adjusted life-years (DALYs) in each population, along with alcohol consumption patterns from the Global Burden of Diseases, Injuries, and Risk Factors Study (GBD) 2020, to estimate the TMREL and NDE for each region, age group, sex, and year from 1990 to 2020. Using these estimates, we quantified the proportion of the population consuming alcohol in amounts exceeding these thresholds by location, age, sex, and year, serving as a guide for targeting alcohol control efforts.

This manuscript was produced as part of the GBD Collaborator Network and in accordance with the GBD Protocol.^30^

## Methods

### Overview

GBD is the most comprehensive effort to date to understand the changing health challenges around the world.^31^ In the most recent revision, GBD 2020, estimates were produced for the mortality and health burden from 287 causes of death, 370 diseases and injuries, and 88 risk factors in 204 countries and territories by 5-year age groups and sex from 1990 to 2020. As part of GBD 2020, we estimated the TMREL and NDE of alcohol consumption for 21 regions by 5-year age group, sex, and year for individuals aged 15–95 years and older from 1990 to 2020 (Bryazka D, unpublished). Using the comparative risk assessment framework, we also quantified the population consuming alcohol in harmful amounts, by 5-year age group, country or territory, sex, and year. In the following sections, we provide an overview of our methods. This study adheres to the Guidelines for Accurate and Transparent Health Estimates Reporting (GATHER) statement.^32^

### Estimating dose–response relative risks

As part of GBD, a previous systematic literature review and meta-analysis was published in 2018 that included 592 cohort and case-control studies across 23 outcomes associated with alcohol use.^1^ These outcomes included ischaemic stroke, intracerebral haemorrhage, ischaemic heart disease, hypertensive heart disease, atrial fibrillation and flutter, lip and oral cavity cancer, nasopharynx cancer, other pharynx cancer, oesophageal cancer, larynx cancer, colon and rectum cancer, breast cancer, liver cancer, type 2 diabetes, cirrhosis and other chronic diseases of the liver, pancreatitis, idiopathic epilepsy, tuberculosis, lower respiratory infection, transport injuries, unintentional injuries, self-harm, and interpersonal violence. As part of this previous meta-analysis, dose–response relative risk curves for each of these outcomes were estimated through use of a Bayesian meta-regression tool, DisMod ODE.^1^, ^33^

For GBD 2020, we updated this review for the six alcohol-attributable outcomes that accounted for the greatest number of global DALYs: ischaemic heart disease, ischaemic stroke, intracerebral haemorrhage, type 2 diabetes, tuberculosis, and lower respiratory infection. Through the update, we included 71 additional studies. After evaluating all available evidence, we found insufficient evidence for a relationship between alcohol use and lower respiratory infection. Based on these results, we removed this as a risk–outcome pair for GBD 2020 and from this analysis, resulting in 22 remaining relative risk curves. Further details of the systematic review, including search strings, inclusion criteria, Preferred Reporting Items for Systematic Reviews and Meta-Analyses (PRISMA) flow diagrams, and relative risk curves are provided in appendix 1 (pp 18–47).

Using the updated data for these five outcomes, we revised the relative risk curves associated with each outcome using the meta-regression Bayesian, regularised, trimmed (MR-BRT) meta-regression tool. MR-BRT is a tool that is well suited to the complex task of estimating the dose–response risk association between alcohol and health because it is does not enforce a log-linear functional form, instead parameterising the log relative risk as a B-spline (Zheng P, Institute for Health Metrics and Evaluation, personal communication). It uses an ensemble approach for knot selection of splines based on level of exposure, and incorporates unexplained between-study heterogeneity into the uncertainty of the relative risk estimates. To adjust for aspects of study design that contribute to bias in relative risks, we included covariates for study reference group, adjustment for sick quitter bias, sex, age, population representativeness, outcome reporting method, exposure measurement timing, geographical representativeness, outcome measure (incidence versus mortality), and adjustment for confounders in risk estimation. The MR-BRT tool uses a generalised Lasso approach to select the most relevant bias covariates to adjust for in the final model. A full list of the confounders tested and included in each of these five models is summarised on in appendix 1 (p 15). Consistent with the previous systematic review,^1^ we utilised a reference group of non-drinkers. We estimated parameter uncertainty using 1000 draws from the posterior distribution, sampled at 1 g intervals of pure alcohol consumption between 0 g and 100 g per day. Further details of the meta-regression approach are available in appendix 1 (pp 14–16).

### Estimating TMREL and NDE

**Figure 1:**
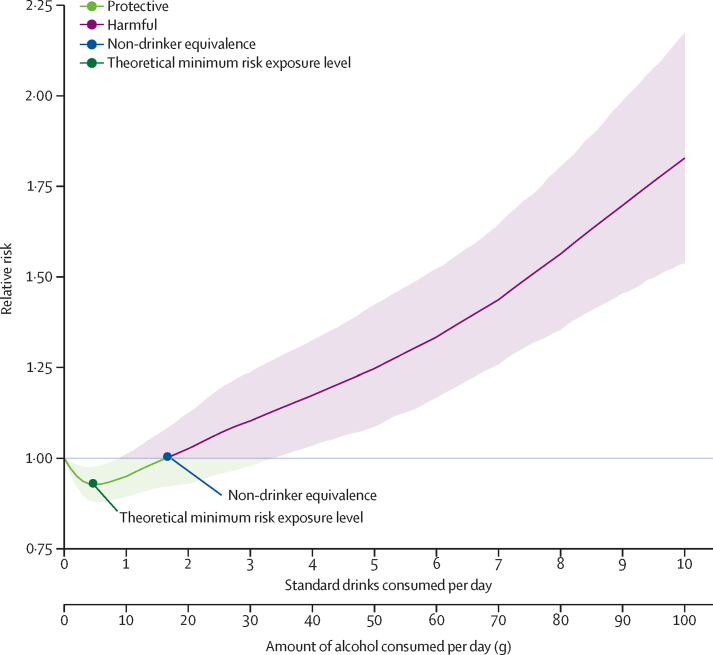
Exemplifying a weighted all-attributable cause alcohol relative risk curve Points mark the theoretical minimum risk exposure level and non-drinker equivalence level. The shaded areas denote consumption levels with a lower risk (green) and greater risk (purple), compared to no consumption. The solid line indicates the mean aggregate relative risk estimate, whereas the shaded area reflects the 95% uncertainty interval of the aggregate relative risk estimate. One standard drink is equivalent to 10 g of pure ethanol.

The TMREL and NDE are based on aggregate, burden-weighted relative risk curves across health outcomes associated with alcohol use. Burden was quantified with DALY rates for each region, age, sex, and year obtained from GBD 2020 (Bryazka D, unpublished). DALYs are the sum of years of life lost (capturing the effect of premature mortality) and years lived with disability (capturing the effect of morbidity). For each region, age, sex, and year, we produced all-attributable cause relative risk curves as a weighted average of cause-specific relative risk curves, with weights based on the share of the overall DALY rates from each cause. The step-by-step process and formula for computing the weighted all-attributable cause curves are provided in appendix 1 (p 16). Using these estimates, we computed the TMREL and NDE by region, age, sex, and year. Uncertainty in the relative risk curve, based on 1000 draws of each cause-specific relative risk curve and 1000 draws of DALY rates used for weighting, was propagated to the estimates of TMREL and NDE. All estimates are presented to three significant figures. An example of a weighted all-attributable cause alcohol relative risk curve, for all 22 alcohol associated causes combined, is shown in figure 1.

Since alcohol use contributes to the DALY rates that are used as weighting factors when constructing the TMREL and NDE, we did a sensitivity analysis that utilised risk-deleted DALY rates as alternative weights. We generated risk-deleted DALY rates by multiplying the DALY rate of each cause by the complement of the cause-specific population-attributable fraction due to alcohol (Bryazka D, unpublished). Additionally, our weighted attributable-cause relative risk curves were based on only 22 of 24 health outcomes since no relative risk curves could be computed for alcohol use disorder or alcoholic cardiomyopathy due to the paucity of studies on dose–response relative risks. To assess whether inclusion of these two outcomes could potentially affect the TMREL and NDE levels, we did a second sensitivity analysis in which we generated conservative hypothetical relative risk functions for alcohol use disorder and alcoholic cardiomyopathy and re-computed TMREL and NDE levels that reflect all 24 alcohol-associated outcomes. Additional details of the sensitivity analyses are presented in the appendix (p 17).

### Estimating prevalence of alcohol use and alcohol consumption

To estimate the proportion of the population consuming alcohol in excess of the NDE, estimates of alcohol consumption in units of grams of pure ethanol consumed per day, on average, by current drinkers for 204 countries and territories, by age, sex, and year, were obtained from GBD 2020 (Bryazka D, unpublished). Briefly, this process combines supply-side data, household survey data, and administrative data, which allows us to adjust for under-reporting due to self-report bias in surveys, account for unrecorded alcohol consumption, and adjust for consumption among tourists. Current drinkers were defined as individuals who had consumed at least one standard drink in the past 12 months. To facilitate interpretation, we report estimates in terms of standard drinks per day, where one standard drink is defined as 10 g of pure ethanol, consistent with previous GBD publications (Bryazka D, unpublished).^1^ Further details on estimation of the prevalence of alcohol use and alcohol consumption have been published previously.^1^

### Role of the funding source

The funder of the study had no role in study design, data collection, data analysis, data interpretation, or writing of the report.

## Results

**Figure 2:**
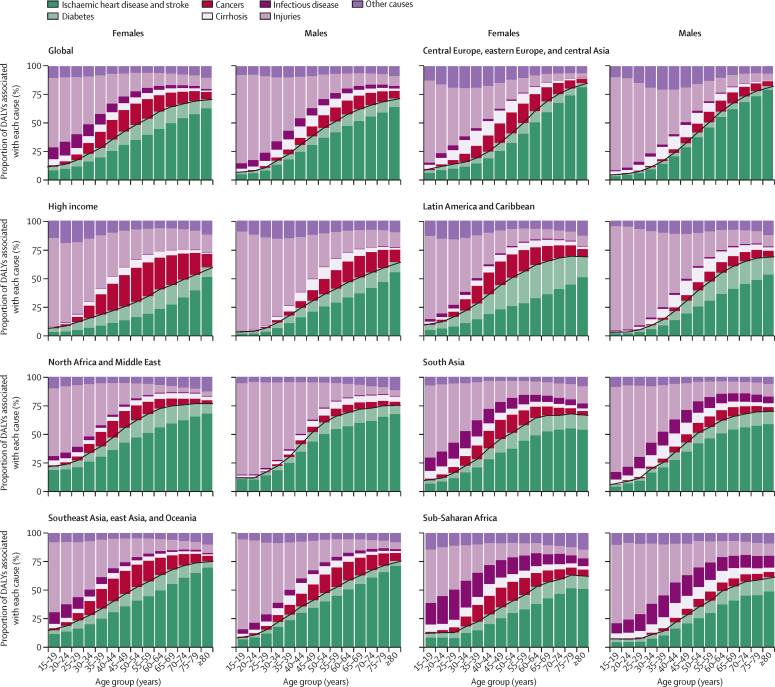
Relative proportions of DALYs for causes associated with alcohol use, by GBD super-region, age group, and sex, in 2020 The proportions represent the weights associated with each cause-specific relative risk curve when constructing each all-cause relative risk curve. The green shades signify causes with a lower risk at low levels of consumption, compared to no consumption. The red and purple shades signify causes with an entirely harmful effect at all levels of consumption. The black line separates causes for which moderate alcohol use lowers risk from causes with an entirely harmful effect. Diabetes includes only type 2 diabetes. Cancers include lip and oral cavity cancer, nasopharynx cancer, other pharynx cancer, oesophageal cancer, larynx cancer, colon and rectum cancer, breast cancer, and liver cancer. Cirrhosis includes cirrhosis and other chronic diseases of the liver. Infectious disease includes tuberculosis. Injuries includes transport injuries, unintentional injuries, self-harm, and interpersonal violence. Other causes include pancreatitis, idiopathic epilepsy, hypertensive heart disease, and atrial fibrillation and flutter. DALY=disability-adjusted life-year.

**Figure 3:**
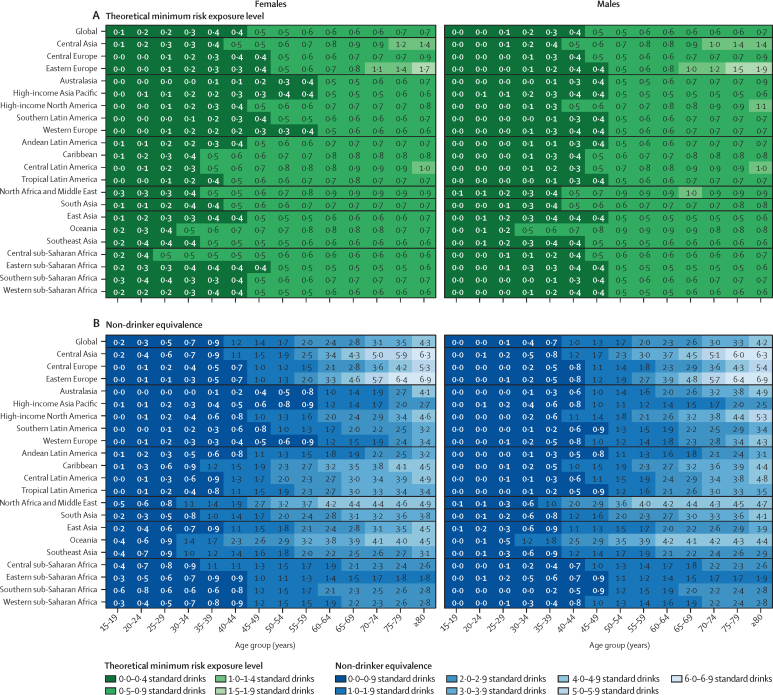
Mean theoretical minimum risk exposure levels (A) and non-drinker equivalence levels (B), in units of standard drinks per day, by region, age group, and sex, in 2020 One standard drink is equivalent to 10 g of pure ethanol.

The distribution of DALYs arising from outcomes associated with alcohol by GBD super-region, age, and sex for 2020 are shown in figure 2. The TMREL and NDE by region, age, and sex for 2020 are shown in figure 3. Overall, we found that the TMREL remained low regardless of geography, age, sex, or time, varying between 0 (95% UI 0–0) and 1·87 (0·500–3·30) standard drinks per day. As a result of the differences in the cause distributions across world regions, both the TMREL and NDE varied by region. The TMREL and NDE did not vary significantly by sex or year. There was significant variation in the TMREL and the NDE across ages, with younger age groups having much lower TMREL and NDE levels than older adults. In 2020, the TMREL varied between 0 (0–0) and 0·603 (0·400–1·00) standard drinks per day among individuals aged 15–39 years and between 0·114 (0–0·403) and 1·87 (0·500–3·30) standard drinks per day among individuals aged 40 years and older. The NDE varied between 0·002 (0–0) and 1·75 (0·698–4·30) standard drinks per day among individuals aged 15–39 years and between 0·193 (0–0·900) and 6·94 (3·40–8·30) standard drinks per day among individuals aged 40 years and older. This result was mainly driven by differences in the major causes of death and disease burden across ages, as seen in figure 2. Overall, we did not observe any significant differences in the TMREL and NDE between males and females in any age group. In all super-regions, among individuals aged 15–39 years, injuries accounted for the majority of alcohol-related DALYs in 2020. Globally, in this age range, all injuries accounted for 66·3% (95% UI 65·1–67·5) of alcohol-related DALYs for males and 47·9% (46·0–49·8) of alcohol-related DALYs for females; transport injuries comprised 25·9% (25·0–27·0) of alcohol-related DALYs among males and 12·7% (12·0–13·4) among females, self-harm comprised 11·7% (10·1–13·3) of alcohol-related DALYs among males and 12·3% (10·8–13·8) among females, and interpersonal violence comprised 12·4% (11·8–13·0) of alcohol-related DALYs among males and 6·70% (5·90–7·69) among females. The TMREL among males aged 15–39 years in 2020 was 0·136 (0–0·400) standard drinks per day. Among females aged 15–39 years in 2020, the TMREL was 0·273 (0–0·500) standard drinks per day. The NDE was 0·249 (0–1·00) standard drinks per day among males and 0·546 (0–1·30) standard drinks per day among females. The differences in the TMREL and the NDE between females and males were not statistically significant.

In individuals aged 40–64 years, the health outcomes contributing to the alcohol-related burden shifted to chronic health conditions, including cardiovascular disease and cancer. In this population, ischaemic heart disease comprised 24·1% (95% UI 23·0–25·3) of alcohol-related DALYs among males and 19·5% (18·0–21·0) among females, and intracerebral haemorrhage comprised 10·3% (9·61–10·9) of alcohol-related DALYs among males and 11·7% (10·7–12·8) among females, whereas injuries, such as transport or unintentional injuries, remained significant sources of burden, comprising 23·0% (21·7–24·4) of alcohol-related DALYs among males and 16·7% (15·3–18·3) of alcohol-related DALYs among females. Health outcomes for which moderate alcohol use is associated with a lower risk constituted an increasing portion of the cause distribution in this age group, resulting in a higher TMREL and NDE than in individuals aged 15–39 years. The global TMREL among individuals aged 40–64 years in 2020 was 0·527 (0·400–1·00) standard drinks per day among males and 0·562 (0·400–0·800) standard drinks per day among females. The global NDE in 2020 was 1·69 (0·800–3·20) standard drinks per day among males and 1·82 (1·00–3·10) standard drinks per day among females. As in the younger age group, the differences in the TMREL and the NDE between females and males aged 40–64 years were not statistically significant.

Among individuals aged 65 years and older, the major causes of disease burden were cardiovascular diseases. In 2020, ischaemic heart disease was responsible for 31·5% (95% UI 30·3–32·7) of all alcohol-related DALYs among males and 29·7% (28·2–31·2) among females, intracerebral haemorrhage was responsible for 11·6% (10·9–12·4) of all alcohol-related DALYs among males and 10·9% (10·1–11·8) among females, and ischaemic stroke was responsible for 14·2% (13·5–14·9) of all alcohol-related DALYs among males and 16·0% (15·2–16·7) among females. As a result, in this population the TMREL was higher than in the younger age groups and was estimated to be 0·636 (0·500–1·00) standard drinks per day among males and 0·656 (0·500–1·00) standard drinks per day among females, whereas the NDE was estimated to be 3·19 (1·50–5·20) standard drinks per day among males and 3·51 (1·70–5·50) standard drinks per day among females. The differences in the TMREL and the NDE between males and females aged 65 years and older were not significant.

The distribution of the causes of disease burden for a given age group varied substantially across regions, resulting in regional variations in TMRELs and NDEs, particularly in individuals aged 40 years and older. For example, among individuals aged 55–59 years in north Africa and the Middle East, 30·7% (95% UI 27·3–34·6) of alcohol-related DALYs were due to cardiovascular disease, 12·6% (11·0–14·3) were due to cancers, and 0·37% (0·27–0·55) were due to tuberculosis. By contrast, in this same age group in central sub-Saharan Africa, 20·1% (17·2–23·8) of alcohol-related DALYs were due to cardiovascular disease, 9·80% (8·31–11·7) were due to cancers, and 10·1% (6·03–14·1) were due to tuberculosis. As a result, the TMRELs for this age group were 0·876 (0·500–2·00) standard drinks per day in north Africa and the Middle East and 0·596 (0·300–2·00) standard drinks per day in central sub-Saharan Africa. The NDEs also varied, with an NDE of 3·89 (1·50–5·90) standard drinks per day in north Africa and the Middle East and 1·53 (0·600–4·70) standard drinks per day in central sub-Saharan Africa. The TMRELs and NDEs for each region by age and sex for 1990, 2000, 2010, and 2020 are shown in appendix 2 (pp 3–31).

**Figure 4:**
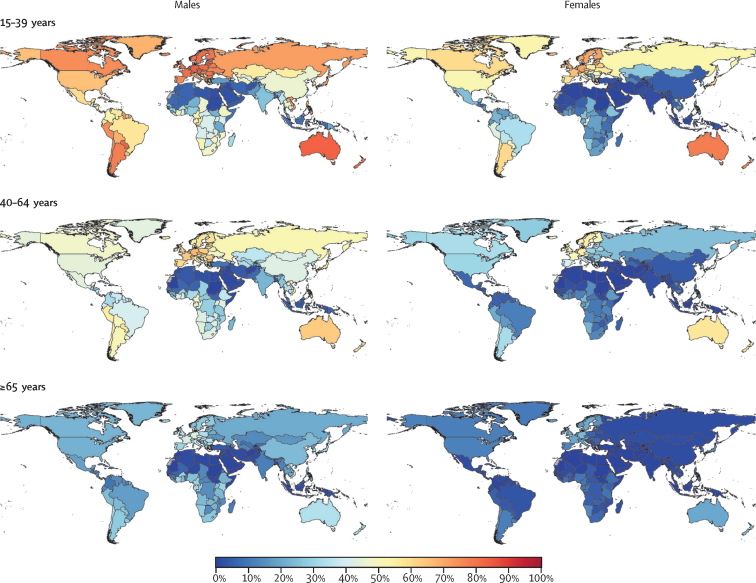
Proportion of the population consuming harmful amounts of alcohol, defined as consumption in excess of the mean non-drinker equivalence level, by sex and age group, in 2020

The distribution of the major causes of DALYs varied slightly between sexes, with injuries making up a larger share of distributions for males than for females. This resulted in mean TMRELs and NDEs that were larger among females compared to males of the same region, age, and year. When taking uncertainty into account, these differences were not significant. However, a larger proportion of males compared to females consume alcohol, and their average level of consumption is also significantly higher. As a result, young males stood out as the group with the highest level of harmful alcohol consumption (figure 4).

Globally, 1·03 billion (95% UI 0·851–1·19) males (35·1% [29·1–40·7] of the male population aged ≥15 years) and 312 million (199–432) females (10·5% [6·72–14·6] of the female population aged ≥15 years) consumed alcohol in amounts exceeding the NDE in 2020; the number and proportion of people consuming alcohol in excess of the NDE, along with the percentage change since 1990 in the proportion of people consuming alcohol in excess of the NDE, by age group, sex, and location is reported in table 1. Since 1990, the global proportion of drinkers consuming alcohol in excess of the NDE has not changed significantly. Although the proportion of the population consuming harmful amounts of alcohol stayed at the same level over the past three decades, the number of people consuming harmful amounts of alcohol increased from 983 million (718–1190) in 1990 to 1·34 billion (1·06–1·62) in 2020, driven by population growth. Overall, among individuals consuming harmful amounts of alcohol in 2020, 76·9% (73·0–81·3) were male.

**Table 1:** Number and proportion of population consuming in excess of the non-drinker equivalence, and percentage change since 1990 by country, age group, and sex, for 2020

	**Females**			**Males**		
	Number (thousands)	Proportion of population (%)	Percentage change since 1990 (%)	Number (thousands)	Proportion of population (%)	Percentage change since 1990 (%)
**Global**						
15–39 years	195 000 (124 000 to 273 000)	13·2% (8·34 to 18·4)	−3·89% (−6·02 to 2·53)	595 000 (489 000 to 658 000)	39·0% (32·1 to 43·2)	−4·62% (−6·27 to −2·64)
40–64 years	98 600 (60 200 to 137 000)	9·22% (5·63 to 12·8)	−4·22% (−6·54 to −0·279)	363 000 (274 000 to 441 000)	34·0% (25·7 to 41·4)	−2·55% (−5·65 to 3·30)
≥65 years	18 400 (8990 to 32 600)	4·49% (2·20 to 7·99)	−1·96% (−3·63 to −0·448)	69 900 (47 000 to 98 500)	20·6% (13·8 to 29·0)	−1·39% (−5·12 to 2·61)
**Central Asia**						
15–39 years	2200 (1320 to 3610)	11·9% (7·16 to 19·5)	−2·93% (−6·61 to 0·104)	8420 (6710 to 9580)	44·5% (35·4 to 50·6)	−4·11% (−9·08 to 0·506)
40–64 years	824 (494 to 1280)	6·52% (3·90 to 10·1)	−0·985% (−3·34 to 0·956)	3740 (2720 to 4910)	32·2% (23·4 to 42·3)	3·29% (−2·37 to 9·14)
≥65 years	27·3 (8·21 to 91·9)	0·841% (0·253 to 2·83)	−0·147% (−0·881 to 0·407)	263 (171 to 477)	12·0% (7·81 to 21·8)	2·42% (0·0992 to 4·88)
**Armenia**						
15–39 years	64·8 (30·9 to 126)	11·8% (5·63 to 23·1)	1·36% (−3·88 to 6·39)	325 (242 to 383)	59·3% (44·1 to 69·9)	−5·32% (−13·7 to 2·88)
40–64 years	22·5 (9·50 to 44·6)	4·50% (1·90 to 8·89)	1·68% (−0·424 to 3·81)	137 (87·4 to 199)	31·9% (20·3 to 46·3)	7·96% (−0·927 to 16·6)
≥65 years	0·657 (0·0590 to 3·52)	0·285% (0·0256 to 1·53)	0·176% (0·00923 to 0·877)	13·9 (6·82 to 32·1)	8·84% (4·33 to 20·4)	3·63% (0·491 to 6·98)
**Azerbaijan**						
15–39 years	191 (124 to 278)	9·19% (5·97 to 13·4)	−1·76% (−5·24 to 1·44)	890 (743 to 1010)	41·0% (34·2 to 46·6)	0·163% (−5·15 to 5·35)
40–64 years	77·9 (43·9 to 113)	4·75% (2·67 to 6·91)	−0·919% (−3·33 to 1·22)	502 (357 to 624)	33·3% (23·7 to 41·3)	2·46% (−3·73 to 8·66)
≥65 years	2·92 (0·698 to 6·65)	0·806% (0·193 to 1·84)	−0·0175% (−0·786 to 0·629)	45·1 (25·0 to 69·5)	16·8% (9·31 to 25·9)	1·92% (−3·77 to 6·91)
**Georgia**						
15–39 years	133 (55·0 to 270)	23·6% (9·76 to 48·0)	2·46% (−7·88 to 12·8)	427 (340 to 486)	71·9% (57·3 to 81·7)	1·18% (−6·60 to 9·21)
40–64 years	37·6 (13·0 to 82·6)	6·04% (2·09 to 13·3)	2·61% (−1·19 to 6·51)	235 (161 to 321)	42·6% (29·2 to 58·2)	13·5% (2·02 to 25·7)
≥65 years	1·14 (0·0390 to 6·83)	0·342% (0·0117 to 2·05)	0·190% (−0·0872 to 1·11)	34·6 (17·7 to 71·7)	17·2% (8·82 to 35·6)	8·13% (0·630 to 15·7)
**Kazakhstan**						
15–39 years	933 (570 to 1530)	26·9% (16·5 to 44·0)	−5·14% (−13·0 to 3·37)	1910 (1490 to 2220)	55·6% (43·3 to 64·6)	−4·33% (−12·1 to 2·87)
40–64 years	386 (227 to 590)	14·4% (8·45 to 22·0)	−3·39% (−10·1 to 2·64)	871 (599 to 1190)	36·5% (25·1 to 49·9)	−2·92% (−10·6 to 5·52)
≥65 years	16·6 (4·32 to 55·5)	1·91% (0·496 to 6·38)	−0·957% (−3·73 to 0·747)	68·2 (39·9 to 130)	13·9% (8·15 to 26·5)	−3·78% (−10·9 to 2·50)
**Kyrgyzstan**						
15–39 years	139 (75·6 to 253)	10·4% (5·63 to 18·8)	0·00207% (−3·95 to 4·51)	529 (415 to 619)	39·0% (30·6 to 45·7)	−1·29% (−6·83 to 4·25)
40–64 years	51·8 (27·7 to 85·8)	6·58% (3·52 to 10·9)	1·05% (−1·44 to 3·64)	221 (161 to 290)	31·2% (22·7 to 40·8)	1·90% (−4·13 to 8·73)
≥65 years	0·834 (0·137 to 3·28)	0·418% (0·0687 to 1·64)	0·0640% (−0·315 to 0·567)	12·7 (7·97 to 22·2)	9·82% (6·16 to 17·1)	1·19% (−2·05 to 4·50)
**Mongolia**						
15–39 years	157 (100 to 243)	25·0% (16·0 to 38·7)	5·71% (−1·72 to 12·0)	350 (282 to 398)	55·1% (44·5 to 62·7)	7·58% (1·06 to 14·4)
40–64 years	62·5 (38·4 to 95·1)	14·4% (8·86 to 22·0)	7·08% (2·93 to 11·3)	163 (122 to 210)	41·9% (31·3 to 53·9)	21·2% (12·8 to 29·5)
≥65 years	1·57 (0·462 to 4·64)	1·88% (0·555 to 5·58)	1·48% (0·476 to 3·54)	8·92 (5·45 to 16·6)	16·5% (10·1 to 30·8)	12·0% (7·81 to 16·8)
**Tajikistan**						
15–39 years	28·8 (17·2 to 45·0)	1·42% (0·847 to 2·22)	−0·182% (−0·845 to 0·466)	489 (397 to 578)	23·4% (19·0 to 27·6)	−0·934% (−5·56 to 3·47)
40–64 years	6·72 (3·98 to 10·6)	0·671% (0·397 to 1·06)	−0·0827% (−0·441 to 0·245)	172 (131 to 221)	17·6% (13·5 to 22·6)	−1·54% (−5·75 to 2·40)
≥65 years	0·137 (0·0370 to 0·364)	0·0814% (0·0219 to 0·216)	−0·0218% (−0·133 to 0·0600)	3·75 (2·25 to 6·18)	2·54% (1·52 to 4·18)	−0·259% (−1·38 to 0·969)
**Turkmenistan**						
15–39 years	123 (64·2 to 218)	12·7% (6·62 to 22·4)	2·66% (−2·83 to 7·36)	533 (417 to 619)	48·3% (37·7 to 56·0)	5·39% (−1·05 to 12·2)
40–64 years	44·4 (20·8 to 78·1)	6·92% (3·25 to 12·2)	3·70% (0·910 to 6·99)	210 (146 to 284)	34·0% (23·5 to 45·8)	15·4% (7·25 to 23·4)
≥65 years	0·974 (0·126 to 3·74)	0·620% (0·0802 to 2·38)	0·482% (0·0614 to 1·70)	14·5 (8·06 to 27·3)	13·0% (7·23 to 24·4)	8·29% (4·05 to 13·3)
**Uzbekistan**						
15–39 years	426 (234 to 728)	6·20% (3·41 to 10·6)	−0·215% (−3·48 to 2·46)	2970 (2340 to 3460)	42·4% (33·4 to 49·3)	−0·720% (−7·28 to 5·87)
40–64 years	135 (72·7 to 228)	3·12% (1·68 to 5·28)	0·981% (−0·604 to 2·44)	1220 (876 to 1650)	30·4% (21·7 to 40·9)	7·90% (−0·290 to 16·2)
≥65 years	2·43 (0·436 to 9·45)	0·289% (0·0519 to 1·12)	0·194% (0·0193 to 0·683)	61·4 (35·3 to 113)	9·73% (5·60 to 17·9)	4·90% (1·64 to 8·69)
**Central Europe**						
15–39 years	8440 (5820 to 10 900)	49·6% (34·2 to 63·7)	2·04% (−3·03 to 7·63)	14 000 (12 200 to 14 900)	78·3% (68·1 to 83·5)	3·80% (0·745 to 7·05)
40–64 years	5350 (3770 to 8180)	26·8% (18·9 to 41·0)	5·49% (1·43 to 10·2)	11 900 (9880 to 14 400)	60·6% (50·4 to 73·7)	10·3% (3·79 to 17·4)
≥65 years	769 (341 to 1620)	6·00% (2·66 to 12·6)	2·01% (0·632 to 3·48)	2980 (2160 to 4330)	33·9% (24·5 to 49·2)	7·44% (3·57 to 10·9)
**Albania**						
15–39 years	112 (58·3 to 158)	24·3% (12·7 to 34·4)	−1·18% (−9·18 to 6·39)	289 (238 to 319)	57·0% (46·9 to 62·7)	6·40% (−0·567 to 13·6)
40–64 years	33·5 (16·5 to 65·2)	7·41% (3·64 to 14·4)	3·86% (1·16 to 7·14)	169 (123 to 227)	40·6% (29·5 to 54·4)	21·2% (12·6 to 27·5)
≥65 years	2·35 (0·353 to 7·73)	1·12% (0·168 to 3·68)	0·993% (0·164 to 2·97)	36·6 (19·5 to 65·2)	18·9% (10·1 to 33·7)	14·8% (8·78 to 20·9)
**Bosnia and Herzegovina**						
15–39 years	125 (82·4 to 166)	24·2% (16·0 to 32·3)	3·65% (−2·87 to 10·1)	357 (311 to 389)	65·1% (56·8 to 71·1)	6·16% (0·147 to 11·9)
40–64 years	69·6 (43·7 to 114)	11·5 (7·21 to 18·8)	4·92 (0·916 to 8·92)	284 (233 to 353)	48·6 (39·8 to 60·4)	17·0 (7·31 to 26·1)
≥65 years	6·95 (2·39 to 15·8)	2·01% (0·690 to 4·56)	1·44% (0·417 to 3·14)	62·9 (42·8 to 93·5)	24·7% (16·8 to 36·7)	13·9% (8·01 to 19·5)
**Bulgaria**						
15–39 years	488 (351 to 624)	52·1% (37·5 to 66·6)	3·64% (−4·86 to 12·2)	798 (706 to 851)	79·9% (70·7 to 85·2)	4·62% (0·386 to 9·03)
40–64 years	382 (283 to 541)	31·1% (23·0 to 44·0)	8·55% (3·07 to 14·6)	798 (680 to 947)	64·8% (55·3 to 77·0)	13·4% (5·56 to 22·4)
≥65 years	62·3 (28·3 to 120)	6·98% (3·17 to 13·5)	3·19% (0·735 to 6·12)	230 (170 to 326)	38·7% (28·6 to 54·7)	11·4% (4·57 to 17·3)
**Croatia**						
15–39 years	315 (193 to 417)	50·8% (31·1 to 67·4)	−0·297% (−8·93 to 8·88)	513 (435 to 555)	79·2% (67·2 to 85·7)	1·08% (−3·76 to 5·60)
40–64 years	183 (111 to 290)	24·5% (14·9 to 39·0)	1·78% (−5·68 to 9·38)	401 (313 to 512)	55·0% (42·9 to 70·2)	4·54% (−3·74 to 14·7)
≥65 years	28·3 (8·87 to 68·4)	5·47% (1·72 to 13·2)	0·141% (−3·90 to 3·53)	110 (67·5 to 175)	30·4% (18·6 to 48·4)	1·72% (−6·72 to 10·1)
**Czech Republic**						
15–39 years	963 (704 to 1210)	66·2% (48·4 to 82·9)	−0·371% (−7·38 to 7·53)	1310 (1170 to 1390)	85·3% (76·2 to 90·6)	1·81% (−1·91 to 5·41)
40–64 years	708 (521 to 1040)	38·3% (28·2 to 56·1)	5·25% (−0·408 to 11·6)	1300 (1100 to 1540)	68·1% (58·0 to 81·1)	7·53% (1·20 to 15·1)
≥65 years	127 (60·0 to 250)	10·1% (4·75 to 19·8)	2·87% (−0·351 to 6·19)	391 (291 to 546)	42·5% (31·6 to 59·3)	6·30% (0·884 to 11·4)
**Hungary**						
15–39 years	695 (474 to 898)	50·7% (34·6 to 65·5)	−2·77% (−10·5 to 5·60)	1100 (972 to 1180)	77·4% (68·2 to 82·8)	−0·0753% (−4·81 to 4·02)
40–64 years	450 (309 to 716)	25·4% (17·4 to 40·3)	−0·0407% (−6·20 to 6·71)	1010 (833 to 1220)	59·5% (49·1 to 71·9)	3·11% (−2·87 to 10·1)
≥65 years	73·2 (30·1 to 155)	5·99% (2·46 to 12·6)	−0·294% (−3·88 to 2·47)	262 (179 to 379)	34·6% (23·6 to 50·1)	1·74% (−4·33 to 7·33)
**Montenegro**						
15–39 years	33·9 (22·2 to 45·6)	33·4% (21·8 to 44·8)	−2·75% (−10·6 to 5·04)	70·1 (60·3 to 76·1)	65·6% (56·4 to 71·2)	−0·548% (−5·57 to 5·10)
40–64 years	18·0 (11·3 to 28·9)	17·1% (10·7 to 27·4)	1·20% (−4·59 to 6·82)	52·5 (41·4 to 65·8)	51·5% (40·6 to 64·5)	6·14% (−2·11 to 16·3)
≥65 years	2·37 (0·774 to 5·54)	4·36% (1·42 to 10·2)	1·42% (−1·12 to 4·38)	11·9 (7·55 to 18·3)	29·5% (18·7 to 45·3)	6·39% (−2·19 to 15·5)
**North Macedonia**						
15–39 years	115 (68·7 to 162)	36·1% (21·5 to 50·8)	−4·09% (−12·4 to 5·66)	251 (212 to 272)	76·7% (64·8 to 83·3)	−0·507% (−6·11 to 4·17)
40–64 years	45·2 (24·5 to 86·3)	14·0% (7·60 to 26·7)	−1·37% (−7·27 to 4·30)	169 (131 to 218)	54·9% (42·6 to 70·7)	0·976% (−6·26 to 8·99)
≥65 years	3·54 (0·728 to 10·2)	2·37% (0·487 to 6·81)	−0·276% (−2·89 to 1·63)	34·0 (21·5 to 53·5)	29·3% (18·6 to 46·2)	−0·672% (−7·80 to 6·31)
**Poland**						
15–39 years	3330 (2270 to 4340)	54·8% (37·4 to 71·6)	4·15% (−3·89 to 12·3)	5150 (4460 to 5530)	81·8% (70·9 to 87·9)	5·91% (1·45 to 10·7)
40–64 years	1970 (1350 to 3060)	29·7% (20·3 to 46·1)	8·70% (3·00 to 14·8)	4060 (3390 to 4900)	63·0% (52·5 to 76·0)	13·8% (5·48 to 22·4)
≥65 years	254 (105 to 551)	6·04% (2·49 to 13·1)	3·32% (1·09 to 5·88)	939 (685 to 1360)	33·6% (24·5 to 48·7)	11·8% (5·68 to 16·7)
**Romania**						
15–39 years	1310 (862 to 1740)	48·5% (31·9 to 64·3)	2·36% (−5·77 to 11·3)	2290 (2010 to 2460)	80·2% (70·4 to 85·9)	3·29% (−1·10 to 7·50)
40–64 years	921 (619 to 1420)	26·9% (18·0 to 41·6)	6·36% (−0·371 to 13·0)	2110 (1750 to 2560)	61·9% (51·1 to 75·0)	11·0% (2·97 to 19·9)
≥65 years	141 (54·9 to 300)	6·32% (2·46 to 13·4)	2·37% (−0·162 to 5·16)	519 (350 to 757)	34·4% (23·2 to 50·2)	8·33% (1·91 to 14·7)
**Serbia**						
15–39 years	285 (194 to 384)	21·2% (14·5 to 28·6)	−1·90% (−8·11 to 4·37)	904 (792 to 980)	63·1% (55·3 to 68·4)	2·53% (−2·55 to 7·65)
40–64 years	186 (125 to 277)	12·6% (8·46 to 18·8)	1·47% (−4·04 to 6·49)	763 (629 to 921)	53·6% (44·2 to 64·7)	9·12% (−0·412 to 21·3)
≥65 years	30·0 (11·8 to 61·6)	3·26% (1·28 to 6·70)	1·18% (−1·52 to 3·55)	212 (151 to 297)	30·0% (21·4 to 42·1)	6·74% (−2·46 to 16·6)
**Slovakia**						
15–39 years	524 (353 to 684)	61·5% (41·4 to 80·3)	−2·67% (−10·5 to 6·55)	724 (625 to 781)	80·9% (69·8 to 87·2)	−0·197% (−5·04 to 3·74)
40–64 years	309 (209 to 483)	32·1% (21·7 to 50·1)	1·92% (−3·90 to 9·21)	600 (497 to 735)	62·8% (52·0 to 76·9)	2·45% (−3·26 to 9·04)
≥65 years	31·4 (10·3 to 77·9)	5·73% (1·88 to 14·2)	0·852% (−2·00 to 3·55)	142 (101 to 204)	39·1% (27·7 to 56·1)	0·980% (−3·64 to 6·44)
**Slovenia**						
15–39 years	151 (77·7 to 220)	54·0% (27·8 to 78·7)	−5·61% (−17·7 to 8·13)	234 (180 to 265)	77·4% (59·7 to 87·8)	−3·83% (−13·5 to 2·26)
40–64 years	68·3 (23·4 to 145)	18·6% (6·37 to 39·6)	−5·52% (−14·1 to 3·61)	165 (88·8 to 257)	43·0% (23·1 to 66·9)	−9·03% (−20·6 to 1·11)
≥65 years	5·77 (0·255 to 20·7)	2·35% (0·104 to 8·41)	−2·20% (−7·29 to 0·192)	34·3 (8·90 to 71·5)	18·8% (4·88 to 39·2)	−11·3% (−20·7 to −3·09)
**Eastern Europe**						
15–39 years	18 200 (12 300 to 22 700)	54·7% (36·8 to 68·1)	−5·33% (−16·7 to 4·98)	25 000 (21 300 to 27 000)	73·5% (62·5 to 79·4)	−2·15% (−8·36 to 2·10)
40–64 years	9930 (6560 to 15 500)	25·6% (16·9 to 39·9)	2·92% (−2·26 to 8·65)	17 000 (13 200 to 22 100)	51·3% (39·9 to 66·7)	3·75% (−2·51 to 11·2)
≥65 years	408 (127 to 1420)	1·87% (0·583 to 6·51)	0·237% (−0·514 to 1·32)	2210 (1510 to 4190)	19·7% (13·4 to 37·3)	1·09% (−2·53 to 4·42)
**Belarus**						
15–39 years	907 (592 to 1130)	61·8% (40·3 to 77·2)	−2·17% (−15·9 to 10·1)	1200 (982 to 1300)	78·8% (64·5 to 85·4)	−0·148% (−7·94 to 5·27)
40–64 years	556 (359 to 853)	30·9% (19·9 to 47·4)	3·69% (−3·19 to 11·6)	825 (610 to 1100)	53·4% (39·5 to 71·2)	3·12% (−4·07 to 12·0)
≥65 years	32·2 (10·6 to 105)	3·27% (1·07 to 10·7)	0·184% (−1·79 to 2·53)	107 (67·5 to 208)	21·8% (13·7 to 42·4)	0·262% (−5·63 to 5·59)
**Estonia**						
15–39 years	137 (97·8 to 163)	71·3% (50·9 to 84·5)	−1·86% (−14·0 to 9·83)	175 (152 to 187)	84·9% (73·5 to 90·3)	0·709% (−5·10 to 5·39)
40–64 years	79·9 (55·3 to 119)	35·9% (24·8 to 53·4)	11·9% (4·74 to 18·3)	120 (92·0 to 155)	57·6% (43·9 to 74·3)	13·8% (4·88 to 23·1)
≥65 years	4·77 (1·25 to 17·7)	2·74 (0·720 to 10·2)	1·68 (0·402 to 5·12)	19·0 (12·3 to 39·1)	20·6 (13·3 to 42·6)	9·21 (4·63 to 14·2)
**Latvia**						
15–39 years	179 (130 to 212)	66·8% (48·4 to 79·1)	−4·22% (−15·4 to 7·48)	232 (205 to 245)	82·6% (73·0 to 87·1)	0·817% (−3·58 to 5·69)
40–64 years	122 (85·0 to 175)	35·5% (24·6 to 50·8)	12·7% (6·14 to 18·5)	187 (151 to 232)	61·4% (49·7 to 76·2)	17·2% (6·96 to 27·6)
≥65 years	9·25 (3·42 to 29·6)	3·54% (1·31 to 11·3)	2·51% (0·974 to 6·09)	37·0 (27·8 to 61·4)	28·6% (21·6 to 47·6)	14·3% (9·87 to 18·6)
**Lithuania**						
15–39 years	296 (201 to 355)	73·8% (50·0 to 88·4)	−2·41% (−15·1 to 9·58)	359 (307 to 382)	85·3% (72·9 to 90·7)	1·01% (−4·81 to 5·79)
40–64 years	173 (113 to 268)	33·4% (21·8 to 51·5)	8·67% (1·56 to 15·5)	259 (196 to 343)	57·2% (43·3 to 75·8)	11·2% (1·82 to 20·8)
≥65 years	12·3 (3·87 to 44·5)	3·35% (1·05 to 12·1)	1·79% (0·415 to 4·74)	40·9 (26·9 to 82·4)	21·8% (14·3 to 43·8)	8·46% (4·09 to 13·0)
**Moldova**						
15–39 years	366 (257 to 447)	58·9% (41·3 to 71·9)	−1·16% (−13·8 to 9·36)	526 (447 to 565)	80·8% (68·7 to 86·8)	−0·983% (−8·09 to 4·18)
40–64 years	211 (147 to 303)	31·1% (21·6 to 44·6)	0·128% (−7·71 to 7·63)	334 (255 to 438)	55·7% (42·6 to 73·1)	−0·201% (−8·14 to 9·58)
≥65 years	13·0 (4·50 to 40·4)	3·91% (1·35 to 12·1)	−1·29% (−4·80 to 1·65)	45·3 (28·3 to 89·8)	22·1% (13·8 to 43·9)	−5·49% (−13·4 to 2·44)
**Russia**						
15–39 years	12 400 (8350 to 15 600)	53·1% (35·7 to 66·8)	−5·67% (−16·6 to 5·08)	17 100 (14 700 to 18 400)	71·6% (61·7 to 77·4)	−2·71% (−8·47 to 2·07)
40–64 years	6720 (4460 to 10 500)	25·0% (16·6 to 39·0)	2·57% (−3·44 to 8·74)	11 900 (9490 to 15 100)	52·0% (41·5 to 66·2)	3·08% (−3·45 to 10·7)
≥65 years	243 (57·8 to 875)	1·64% (0·390 to 5·91)	0·263% (−0·680 to 1·74)	1550 (1050 to 2820)	20·5% (13·9 to 37·3)	0·925% (−3·79 to 5·54)
**Ukraine**						
15–39 years	3900 (2420 to 4980)	56·4% (35·0 to 71·9)	−4·74% (−18·3 to 7·58)	5470 (4350 to 6030)	76·5% (60·9 to 84·4)	−0·693% (−8·78 to 4·87)
40–64 years	2070 (1280 to 3370)	24·5% (15·1 to 39·9)	2·95% (−3·55 to 9·68)	3380 (2340 to 4790)	47·3% (32·8 to 67·0)	4·14% (−4·15 to 13·0)
≥65 years	92·6 (24·6 to 320)	1·89% (0·501 to 6·53)	0·0302% (−1·30 to 1·45)	410 (223 to 921)	16·0% (8·69 to 35·9)	−0·119% (−5·36 to 5·13)
**Australasia**						
15–39 years	3910 (3290 to 4130)	77·7% (65·3 to 82·0)	−4·37% (−12·8 to 1·58)	4210 (3600 to 4400)	83·2% (71·1 to 86·9)	−2·38% (−9·26 to 0·990)
40–64 years	2700 (1870 to 3650)	57·0% (39·5 to 77·2)	4·33% (−6·08 to 14·6)	2830 (2140 to 3630)	62·3% (47·2 to 80·0)	7·69% (−1·03 to 15·4)
≥65 years	536 (283 to 865)	20·3% (10·7 to 32·7)	10·5% (2·07 to 16·7)	772 (504 to 1230)	33·6% (21·9 to 53·4)	8·12% (0·525 to 15·0)
**Australia**						
15–39 years	3280 (2740 to 3490)	78·1% (65·3 to 83·2)	−3·65% (−12·7 to 2·99)	3500 (3000 to 3670)	83·5% (71·5 to 87·4)	−2·07% (−9·33 to 1·66)
40–64 years	2230 (1530 to 3050)	56·7% (38·7 to 77·5)	4·88% (−6·07 to 15·6)	2380 (1810 to 3050)	63·0% (47·9 to 80·7)	8·04% (−0·866 to 15·9)
≥65 years	439 (226 to 726)	19·7% (10·2 to 32·6)	10·7% (2·85 to 17·2)	656 (431 to 1040)	34·0% (22·3 to 53·8)	8·35% (0·612 to 15·4)
**New Zealand**						
15–39 years	634 (546 to 665)	75·6% (65·1 to 79·3)	−7·92% (−14·7 to −2·74)	709 (605 to 742)	81·7% (69·8 to 85·5)	−3·88% (−10·5 to −0·412)
40–64 years	463 (348 to 607)	58·2% (43·6 to 76·2)	1·44% (−8·62 to 10·8)	446 (330 to 589)	58·7% (43·5 to 77·6)	6·04% (−3·22 to 13·7)
≥65 years	97·8 (57·5 to 149)	23·4% (13·7 to 35·7)	9·97% (−0·197 to 17·0)	116 (72·2 to 189)	31·5% (19·7 to 51·5)	6·85% (−1·20 to 14·4)
**High–income Asia Pacific**						
15–39 years	12 800 (7820 to 15 200)	51·3% (31·4 to 61·1)	0·211% (−12·8 to 10·9)	17 800 (12 400 to 20 000)	66·9% (46·6 to 75·1)	−3·96% (−19·1 to 4·23)
40–64 years	12 000 (9160 to 16 700)	36·5% (27·7 to 50·7)	−2·79% (−8·44 to 3·88)	17 700 (14 100 to 22 600)	52·3% (41·8 to 66·9)	−6·77% (−13·3 to −0·928)
≥65 years	3390 (2210 to 4870)	13·2% (8·61 to 19·0)	−0·103% (−3·77 to 3·58)	6110 (4340 to 8250)	30·6% (21·7 to 41·3)	−2·03% (−7·53 to 3·57)
**Brunei**						
15–39 years	2·35 (1·14 to 3·29)	2·50% (1·22 to 3·52)	−1·01% (−2·44 to 0·248)	6·36 (2·75 to 8·58)	5·78% (2·50 to 7·79)	−4·43% (−7·49 to −2·20)
40–64 years	1·02 (0·395 to 1·66)	1·68% (0·655 to 2·76)	−1·04% (−2·42 to 0·0405)	2·08 (0·331 to 3·84)	3·22% (0·511 to 5·94)	−4·92% (−7·44 to −2·81)
≥65 years	0·0632 (0·00798 to 0·119)	0·533% (0·0673 to 1·01)	−0·397% (−1·01 to 0·158)	0·135 (0·00400 to 0·263)	1·23% (0·0364 to 2·40)	−2·31% (−3·88 to −0·898)
**Japan**						
15–39 years	9850 (5760 to 11 900)	62·2% (36·4 to 75·2)	1·84% (−16·3 to 15·2)	12 100 (7770 to 13 800)	73·2% (47·0 to 83·6)	−2·86% (−21·8 to 6·97)
40–64 years	9370 (6930 to 13 400)	43·9% (32·5 to 62·9)	0·924% (−6·35 to 9·81)	12 000 (9070 to 16 000)	55·6% (42·1 to 74·3)	−5·46% (−12·7 to 1·52)
≥65 years	2910 (1820 to 4260)	14·1% (8·81 to 20·7)	0·197% (−3·97 to 4·47)	4920 (3280 to 6840)	30·7% (20·5 to 42·7)	−2·14% (−7·99 to 4·04)
**South Korea**						
15–39 years	2630 (1860 to 3230)	33·0% (23·4 to 40·5)	−0·638% (−9·15 to 8·51)	5280 (4370 to 5810)	58·7% (48·5 to 64·5)	−3·27% (−11·3 to 3·06)
40–64 years	2520 (2000 to 3230)	23·7% (18·8 to 30·4)	−1·21% (−7·71 to 5·20)	5460 (4810 to 6240)	49·4% (43·4 to 56·3)	−3·98% (−9·71 to 1·87)
≥65 years	469 (337 to 627)	10·1% (7·25 to 13·5)	−0·0438% (−3·93 to 3·88)	1160 (960 to 1380)	32·1% (26·6 to 38·2)	−0·965% (−7·45 to 5·41)
**Singapore**						
15–39 years	286 (126 to 384)	28·8% (12·8 to 38·8)	8·01% (−3·57 to 17·4)	435 (200 to 568)	42·4% (19·4 to 55·2)	−1·38% (−18·8 to 8·97)
40–64 years	157 (98·3 to 262)	15·3% (9·61 to 25·6)	4·07% (−0·107 to 10·2)	260 (154 to 431)	23·0% (13·6 to 38·1)	−1·70% (−8·43 to 4·16)
≥65 years	10·9 (5·86 to 17·9)	2·85% (1·54 to 4·71)	0·826% (−0·460 to 2·36)	27·7 (14·2 to 44·3)	8·08% (4·13 to 12·9)	0·941% (−2·00 to 4·00)
**High–income North America**						
15–39 years	32 200 (21 700 to 38 700)	53·0% (35·6 to 63·7)	−7·72% (−14·1 to −1·78)	41 000 (34 300 to 44 300)	66·2% (55·4 to 71·5)	−7·17% (−11·3 to −4·16)
40–64 years	18 500 (13 500 to 26 000)	30·9% (22·5 to 43·4)	2·00% (−5·74 to 6·46)	25 700 (19 800 to 33 100)	44·6% (34·4 to 57·5)	5·93% (−1·10 to 11·0)
≥65 years	4110 (2270 to 7140)	12·0% (6·63 to 20·9)	6·43% (3·67 to 9·71)	6120 (4050 to 9890)	22·0% (14·6 to 35·6)	7·62% (3·52 to 11·5)
**Canada**						
15–39 years	3420 (2170 to 4240)	60·1 (38·1 to 74·3)	2·39 (−7·63 to 12·5)	4340 (3540 to 4710)	75·1 (61·4 to 81·6)	1·33 (−4·50 to 5·65)
40–64 years	2060 (1460 to 3010)	32·8% (23·3 to 47·8)	2·60% (−4·85 to 8·87)	2870 (2170 to 3730)	47·5% (36·0 to 61·6)	7·20% (0·309 to 12·3)
≥65 years	425 (209 to 780)	11·5% (5·67 to 21·2)	5·77% (2·53 to 9·72)	708 (453 to 1170)	22·7% (14·5 to 37·5)	7·51% (3·07 to 12·1)
**Greenland**						
15–39 years	5·26 (3·31 to 6·50)	52·9% (33·3 to 65·4)	−5·57% (−15·5 to 4·19)	7·13 (5·76 to 7·77)	67·8% (54·9 to 74·0)	−3·25% (−9·06 to 1·69)
40–64 years	2·40 (1·42 to 3·62)	27·9% (16·5 to 42·0)	0·312% (−11·2 to 11·3)	4·41 (2·86 to 5·87)	43·3% (28·0 to 57·6)	6·42% (−5·08 to 19·4)
≥65 years	0·249 (0·0861 to 0·475)	10·5% (3·62 to 20·0)	6·04% (−0·0437 to 12·6)	0·615 (0·307 to 1·02)	22·8% (11·3 to 37·6)	10·4% (−1·09 to 21·3)
**USA**						
15–39 years	28 800 (19 500 to 34 700)	52·2% (35·4 to 63·0)	−8·78% (−14·9 to −2·88)	36 600 (30 600 to 39 600)	65·3% (54·6 to 70·5)	−8·04% (−12·3 to −4·89)
40–64 years	16 500 (12 000 to 23 000)	30·7% (22·4 to 43·0)	1·93% (−5·70 to 6·52)	22 800 (17 600 to 29 400)	44·3% (34·1 to 57·1)	5·78% (−1·65 to 11·1)
≥65 years	3690 (2040 to 6340)	12·1% (6·69 to 20·8)	6·51% (3·70 to 9·77)	5420 (3570 to 8690)	21·9% (14·5 to 35·2)	7·62% (3·29 to 11·7)
**Southern Latin America**						
15–39 years	7500 (5340 to 8610)	59·0% (42·0 to 67·8)	4·31% (−3·75 to 12·4)	9690 (8560 to 10 200)	76·7% (67·7 to 80·5)	−0·186% (−3·91 to 3·05)
40–64 years	3200 (2350 to 4670)	32·6% (24·0 to 47·6)	−1·25% (−6·45 to 5·38)	4790 (3790 to 6170)	52·3% (41·4 to 67·4)	−3·31% (−7·73 to 1·70)
≥65 years	556 (322 to 863)	12·2% (7·05 to 18·9)	−0·0526% (−3·56 to 3·17)	979 (666 to 1360)	29·1% (19·8 to 40·6)	−3·24% (−7·62 to 1·76)
**Argentina**						
15–39 years	5290 (3750 to 6190)	60·5% (42·9 to 70·7)	3·21% (−6·09 to 12·6)	6700 (5890 to 7060)	77·7% (68·4 to 81·9)	−1·40% (−5·71 to 2·81)
40–64 years	2050 (1460 to 3060)	32·3% (23·0 to 48·2)	−3·06% (−9·65 to 4·67)	3070 (2400 to 3980)	51·6% (40·3 to 67·0)	−6·09% (−11·3 to 0·455)
≥65 years	343 (187 to 565)	11·5% (6·27 to 18·9)	−1·72% (−5·99 to 2·50)	603 (397 to 856)	27·8% (18·3 to 39·5)	−6·87% (−12·1 to −1·57)
**Chile**						
15–39 years	1950 (1370 to 2280)	57·9% (40·7 to 67·8)	5·69% (−3·64 to 15·6)	2600 (2310 to 2740)	76·0% (67·5 to 80·1)	1·58% (−3·14 to 6·33)
40–64 years	1000 (752 to 1410)	34·3% (25·7 to 48·1)	2·19% (−4·80 to 8·83)	1490 (1210 to 1860)	54·8% (44·5 to 68·5)	2·21% (−2·46 to 7·34)
≥65 years	178 (106 to 268)	14·1% (8·38 to 21·2)	3·83% (−0·526 to 7·63)	318 (223 to 431)	32·5% (22·8 to 44·2)	4·90% (−0·805 to 10·6)
**Uruguay**						
15–39 years	264 (189 to 314)	43·6% (31·2 to 51·9)	4·20% (−5·20 to 13·5)	395 (345 to 423)	66·2% (57·9 to 71·0)	0·745% (−4·29 to 5·94)
40–64 years	146 (107 to 214)	27·5% (20·1 to 40·4)	1·67% (−4·43 to 8·32)	231 (183 to 294)	47·8% (37·9 to 60·9)	1·51% (−3·45 to 7·00)
≥65 years	34·6 (19·3 to 55·4)	11·1% (6·16 to 17·7)	2·03% (−2·02 to 5·89)	57·9 (39·5 to 80·4)	27·4% (18·7 to 38·1)	1·88% (−3·43 to 7·51)
**Western Europe**						
15–39 years	41 000 (28 300 to 47 000)	64·3% (44·3 to 73·6)	−4·40% (−15·8 to 3·08)	52 600 (44 500 to 56 000)	79·3% (67·1 to 84·5)	−3·15% (−9·01 to −0·0908)
40–64 years	36 000 (26 600 to 50 900)	48·0% (35·4 to 67·8)	−1·03% (−7·36 to 5·93)	45 300 (36 700 to 57 300)	61·4% (49·7 to 77·6)	−0·157% (−4·75 to 4·95)
≥65 years	9340 (6000 to 13 900)	18·9% (12·1 to 28·1)	4·92% (0·0743 to 8·75)	14 300 (10 200 to 20 700)	36·5% (25·9 to 52·7)	2·80% (−1·86 to 6·59)
**Andorra**						
15–39 years	8·28 (5·59 to 9·89)	66·9% (45·1 to 79·8)	−5·56% (−19·7 to 4·71)	10·5 (8·71 to 11·4)	80·5% (66·6 to 87·4)	−3·86% (−10·3 to 0·358)
40–64 years	8·56 (6·04 to 12·2)	50·5% (35·7 to 72·1)	−1·18% (−10·6 to 8·82)	11·4 (9·00 to 14·6)	61·5% (48·7 to 78·9)	−0·478% (−7·38 to 6·96)
≥65 years	1·26 (0·751 to 1·94)	19·3% (11·5 to 29·7)	4·90% (−2·99 to 11·6)	2·28 (1·53 to 3·42)	35·6% (23·8 to 53·3)	2·85% (−5·91 to 11·8)
**Austria**						
15–39 years	874 (612 to 1030)	63·8% (44·6 to 75·3)	−6·59% (−18·1 to 3·50)	1160 (982 to 1250)	79·9% (67·7 to 85·9)	−3·48% (−9·90 to 0·603)
40–64 years	774 (567 to 1070)	49·1% (35·9 to 68·1)	−1·47% (−9·42 to 7·35)	959 (780 to 1200)	61·4% (50·0 to 76·7)	−0·293% (−5·47 to 5·32)
≥65 years	187 (116 to 275)	19·5% (12·1 to 28·7)	3·68% (−2·40 to 8·83)	259 (179 to 378)	35·3% (24·5 to 51·5)	2·06% (−3·46 to 7·57)
**Belgium**						
15–39 years	1160 (810 to 1370)	66·8% (46·5 to 78·5)	−3·76% (−16·0 to 5·81)	1400 (1190 to 1500)	79·1% (67·7 to 85·0)	−2·61% (−8·21 to 1·53)
40–64 years	966 (700 to 1380)	50·9% (36·9 to 72·9)	0·230% (−8·05 to 10·0)	1160 (923 to 1470)	60·6% (48·3 to 76·8)	−0·459% (−5·74 to 5·14)
≥65 years	247 (146 to 381)	20·1% (11·9 to 30·9)	5·15% (−0·207 to 10·8)	336 (222 to 503)	34·5% (22·9 to 51·7)	1·25% (−4·32 to 6·50)
**Cyprus**						
15–39 years	136 (84·0 to 171)	54·8% (33·9 to 68·8)	−6·12% (−19·5 to 5·30)	212 (172 to 230)	83·1% (67·4 to 90·1)	−0·761% (−8·12 to 3·36)
40–64 years	81·6 (49·8 to 126)	36·1% (22·1 to 55·8)	0·596% (−8·51 to 10·1)	124 (96·9 to 162)	61·1% (47·8 to 79·8)	3·41% (−2·93 to 9·34)
≥65 years	11·3 (5·66 to 18·4)	11·2% (5·60 to 18·2)	4·60% (0·0601 to 8·89)	30·1 (19·6 to 44·6)	33·0% (21·5 to 48·9)	7·14% (0·266 to 13·0)
**Denmark**						
15–39 years	666 (439 to 779)	75·1% (49·5 to 87·9)	−3·91% (−18·5 to 6·36)	790 (645 to 852)	85·2% (69·6 to 91·9)	−2·19% (−9·90 to 1·69)
40–64 years	525 (358 to 775)	55·4% (37·8 to 81·9)	−2·26% (−10·8 to 8·23)	564 (417 to 774)	59·3% (43·9 to 81·3)	−1·42% (−7·31 to 5·42)
≥65 years	144 (83·8 to 230)	23·0% (13·4 to 36·7)	6·78% (−0·0593 to 13·2)	180 (110 to 292)	33·8% (20·7 to 54·9)	2·35% (−3·87 to 8·27)
**Finland**						
15–39 years	572 (379 to 676)	70·6% (46·8 to 83·4)	−4·73% (−18·5 to 5·81)	683 (562 to 741)	79·6% (65·4 to 86·2)	−3·33% (−9·16 to 0·782)
40–64 years	442 (307 to 658)	51·1% (35·6 to 76·1)	1·40% (−7·09 to 10·2)	514 (398 to 680)	58·7% (45·5 to 77·7)	3·64% (−2·83 to 9·90)
≥65 years	112 (59·6 to 184)	15·9% (8·42 to 26·0)	7·64% (2·59 to 12·6)	155 (91·0 to 250)	27·8% (16·3 to 44·8)	7·77% (1·20 to 13·5)
**France**						
15–39 years	6510 (4450 to 7700)	65·4% (44·8 to 77·4)	−5·31% (−17·1 to 4·05)	8040 (6870 to 8590)	81·0% (69·2 to 86·5)	−3·18% (−8·52 to 0·837)
40–64 years	5420 (3890 to 7720)	50·2% (36·0 to 71·5)	−1·65%% (−9·67 to 7·07)	6640 (5410 to 8370)	64·0% (52·1 to 80·7)	−0·712% (−5·72 to 4·54)
≥65 years	1500 (897 to 2270)	19·5% (11·7 to 29·6)	4·73% (−1·33 to 10·0)	2430 (1740 to 3470)	41·4% (29·6 to 59·0)	1·29% (−4·39 to 6·92)
**Germany**						
15–39 years	8470 (6000 to 9820)	70·8% (50·2 to 82·1)	−5·45% (−17·4 to 3·86)	11 100 (9550 to 11 700)	83·6% (72·2 to 88·8)	−2·97% (−8·45 to 0·596)
40–64 years	8080 (6090 to 10 900)	54·9% (41·4 to 74·1)	−1·41% (−8·98 to 6·50)	9900 (8350 to 11 900)	66·7% (56·2 to 80·1)	−0·556% (−5·44 to 4·74)
≥65 years	2440 (1620 to 3580)	23·8% (15·7 to 34·9)	5·24% (−1·12 to 11·0)	3450 (2550 to 4700)	42·7% (31·6 to 58·3)	1·53% (−3·99 to 7·04)
**Greece**						
15–39 years	999 (629 to 1210)	67·0% (42·2 to 81·1)	−4·87% (−19·6 to 6·49)	1240 (1020 to 1350)	81·1% (66·6 to 88·2)	−3·18% (−11·3 to 1·61)
40–64 years	778 (481 to 1240)	40·6% (25·1 to 64·5)	0·187% (−8·23 to 10·6)	1070 (815 to 1410)	58·3% (44·6 to 77·0)	−0·685% (−7·00 to 5·94)
≥65 years	123 (56·6 to 220)	9·64% (4·44 to 17·3)	2·13% (−2·39 to 5·91)	304 (189 to 482)	29·5% (18·3 to 46·7)	−0·997% (−6·15 to 4·53)
**Iceland**						
15–39 years	38·5 (24·4 to 46·1)	66·5% (42·1 to 79·6)	−4·38% (−18·6 to 9·44)	48·0 (38·6 to 52·4)	77·3% (62·1 to 84·3)	0·215% (−5·41 to 7·05)
40–64 years	26·0 (17·6 to 38·9)	49·1% (33·3 to 73·4)	9·38% (−1·35 to 18·2)	30·5 (22·8 to 41·0)	56·6% (42·3 to 76·1)	18·1% (7·34 to 26·0)
≥65 years	4·74 (2·60 to 7·45)	16·9% (9·27 to 26·6)	12·9% (7·91 to 18·2)	7·36 (4·45 to 11·7)	29·4% (17·8 to 46·6)	20·3% (13·7 to 26·5)
**Ireland**						
15–39 years	556 (407 to 641)	69·5% (50·9 to 80·1)	−4·42% (−15·9 to 4·48)	618 (528 to 665)	78·7% (67·2 to 84·7)	−2·38% (−7·90 to 1·91)
40–64 years	412 (302 to 561)	50·8% (37·2 to 69·3)	1·93% (−5·99 to 10·0)	459 (366 to 588)	58·0% (46·3 to 74·4)	3·38% (−2·10 to 9·41)
≥65 years	66·9 (39·9 to 101)	17·4% (10·3 to 26·3)	7·05% (1·87 to 12·0)	103 (68·0 to 154)	30·1% (19·8 to 45·0)	6·45% (1·00 to 11·3)
**Israel**						
15–39 years	827 (404 to 1060)	51·0% (24·9 to 65·6)	−1·73% (−16·6 to 11·7)	1150 (810 to 1320)	68·8% (48·6 to 79·1)	1·43% (−6·94 to 9·51)
40–64 years	378 (182 to 679)	31·0% (15·0 to 55·7)	7·49% (−1·90 to 18·2)	407 (227 to 725)	34·4% (19·2 to 61·3)	14·7% (6·89 to 23·2)
≥65 years	35·2 (12·6 to 68·8)	5·52% (1·98 to 10·8)	4·64% (1·84 to 8·15)	50·2 (16·4 to 116)	9·61% (3·15 to 22·2)	7·91% (2·95 to 14·6)
**Italy**						
15–39 years	4010 (2520 to 4820)	51·6% (32·4 to 61·9)	−2·55% (−14·0 to 6·32)	5990 (4880 to 6510)	73·5% (59·9 to 79·8)	−4·77% (−12·7 to −0·0258)
40–64 years	4550 (3350 to 6300)	39·9% (29·3 to 55·2)	−4·63% (−11·2 to 2·23)	6520 (5140 to 8430)	59·2% (46·6 to 76·5)	−3·83% (−9·27 to 2·47)
≥65 years	1420 (937 to 2010)	18·1% (11·9 to 25·6)	0·235% (−5·15 to 5·33)	2210 (1510 to 3190)	36·5% (24·9 to 52·6)	−3·30% (−9·25 to 2·95)
**Luxembourg**						
15–39 years	71·7 (47·8 to 85·6)	67·1% (44·8 to 80·2)	−5·55% (−18·7 to 4·32)	89·3 (75·2 to 96·3)	80·8% (68·1 to 87·2)	−3·23% (−9·49 to 0·854)
40–64 years	55·8 (39·9 to 79·9)	51·9% (37·1 to 74·2)	−0·380% (−9·38 to 8·85)	71·9 (58·2 to 90·2)	63·4% (51·3 to 79·5)	−0·448% (−5·54 to 5·81)
≥65 years	9·99 (5·74 to 15·9)	19·7% (11·3 to 31·4)	5·61% (−0·641 to 11·0)	17·4 (12·4 to 24·4)	41·5% (29·6 to 58·1)	1·78% (−3·66 to 7·50)
**Malta**						
15–39 years	40·4 (24·6 to 49·8)	61·3% (37·3 to 75·6)	−3·90% (−16·9 to 7·94)	55·1 (44·5 to 60·2)	78·7% (63·6 to 85·9)	−0·0659% (−6·20 to 4·71)
40–64 years	25·0 (15·7 to 39·0)	35·1% (22·1 to 54·8)	1·21% (−7·32 to 8·80)	38·4 (29·0 to 51·6)	52·6% (39·8 to 70·7)	6·32% (−1·17 to 12·9)
≥65 years	5·58 (2·92 to 9·10)	10·9% (5·67 to 17·7)	6·09% (2·27 to 10·1)	11·4 (7·08 to 17·7)	26·7% (16·6 to 41·6)	10·8% (5·11 to 16·0)
**Monaco**						
15–39 years	2·99 (1·38 to 3·70)	64·0% (29·5 to 79·2)	−4·16% (−29·5 to 18·9)	3·55 (2·38 to 3·98)	76·4% (51·1 to 85·6)	−2·14% (−19·9 to 12·9)
40–64 years	2·82 (0·286 to 4·87)	40·0% (4·07 to 69·1)	2·47% (−35·7 to 37·2)	3·18 (0·352 to 5·16)	46·1% (5·12 to 74·9)	5·93% (−41·0 to 51·4)
≥65 years	0·665 (0·00200 to 1·57)	12·6% (0·0381 to 29·8)	6·49% (−12·3 to 25·2)	0·966 (0·00598 to 2·14)	22·1% (0·137 to 48·8)	7·69% (−24·7 to 36·5)
**Netherlands**						
15–39 years	1660 (1110 to 1970)	63·8% (42·7 to 75·8)	−2·73% (−16·0 to 7·70)	2100 (1700 to 2280)	78·4% (63·6 to 85·1)	−2·34% (−9·19 to 2·30)
40–64 years	1430 (1010 to 2070)	49·1% (34·9 to 71·0)	−0·456% (−8·76 to 8·35)	1680 (1270 to 2240)	57·9% (43·9 to 77·2)	1·13% (−4·48 to 7·01)
≥65 years	397 (246 to 598)	21·8% (13·5 to 32·8)	7·76% (1·90 to 13·2)	529 (341 to 820)	33·8% (21·8 to 52·3)	5·51% (−0·705 to 11·1)
**Norway**						
15–39 years	599 (392 to 706)	69·8% (45·6 to 82·2)	−2·79% (−17·1 to 7·98)	730 (583 to 796)	80·2% (64·0 to 87·4)	−0·420% (−6·68 to 4·68)
40–64 years	431 (287 to 658)	50·8% (33·9 to 77·6)	7·08% (−1·82 to 16·2)	478 (343 to 673)	53·7% (38·5 to 75·6)	11·3% (2·41 to 18·4)
≥65 years	86·6 (45·2 to 144)	17·1% (8·91 to 28·5)	11·8% (6·77 to 16·9)	111 (59·5 to 194)	25·2% (13·6 to 44·2)	14·2% (8·74 to 19·8)
**Portugal**						
15–39 years	719 (490 to 888)	47·8% (32·6 to 59·0)	−0·371% (−11·2 to 9·71)	1110 (966 to 1200)	74·7% (64·7 to 80·2)	−0·460% (−6·71 to 4·36)
40–64 years	743 (542 to 1040)	36·6% (26·7 to 51·0)	1·14% (−6·41 to 9·92)	1180 (998 to 1410)	63·9% (54·2 to 76·7)	0·890% (−3·90 to 6·00)
≥65 years	181 (112 to 263)	13·2% (8·22 to 19·2)	2·93% (−1·95 to 7·36)	402 (298 to 544)	39·7% (29·4 to 53·7)	1·23% (−3·96 to 6·26)
**San Marino**						
15–39 years	3·01 (1·37 to 3·63)	65·7% (30·0 to 79·3)	−4·69% (−32·1 to 15·3)	3·54 (2·42 to 3·87)	79·1% (54·1 to 86·5)	−2·37% (−19·4 to 13·2)
40–64 years	3·11 (0·0910 to 4·70)	47·1% (1·38 to 71·1)	2·58% (−38·5 to 40·9)	3·32 (0·0440 to 4·65)	56·2% (0·745 to 78·9)	4·11% (−49·1 to 58·0)
≥65 years	0·658 (0 to 1·20)	18·0% (0 to 32·8)	6·68% (−13·5 to 23·5)	1·03 (0 to 1·75)	33·0% (0 to 56·2)	7·12% (−28·6 to 40·5)
**Spain**						
15–39 years	3620 (2550 to 4330)	57·6% (40·7 to 69·0)	−2·29% (−13·6 to 8·29)	4850 (4100 to 5260)	75·8% (64·1 to 82·1)	−1·66% (−8·34 to 3·39)
40–64 years	3540 (2600 to 4870)	40·6% (29·8 to 55·9)	0·135% (−7·78 to 8·23)	5170 (4190 to 6510)	59·7% (48·5 to 75·2)	0·144% (−5·16 to 5·98)
≥65 years	647 (393 to 956)	12·5% (7·62 to 18·5)	2·03% (−2·81 to 5·85)	1190 (805 to 1730)	30·3% (20·5 to 44·2)	−0·548% (−5·79 to 4·42)
**Sweden**						
15–39 years	1090 (710 to 1280)	70·1% (45·7 to 82·5)	−2·94% (−17·3 to 6·88)	1350 (1080 to 1470)	81·2% (65·3 to 88·5)	−1·16% (−8·29 to 3·22)
40–64 years	857 (599 to 1250)	54·9% (38·4 to 79·9)	2·28% (−5·77 to 11·2)	920 (692 to 1260)	57·4% (43·2 to 78·7)	4·49% (−2·22 to 10·9)
≥65 years	230 (134 to 361)	20·5% (11·9 to 32·1)	9·85% (5·09 to 15·0)	270 (158 to 457)	27·4% (16·1 to 46·5)	8·36% (2·81 to 13·9)
**Switzerland**						
15–39 years	898 (578 to 1080)	66·4% (42·7 to 80·0)	−6·21% (−20·4 to 4·45)	1140 (928 to 1240)	80·2% (65·2 to 87·2)	−4·06% (−12·0 to 0·526)
40–64 years	763 (536 to 1120)	50·1% (35·2 to 73·2)	−2·69% (−10·8 to 6·12)	938 (734 to 1230)	60·3% (47·1 to 79·1)	−2·80% (−8·28 to 3·15)
≥65 years	179 (102 to 292)	19·5% (11·1 to 31·8)	4·67% (−1·63 to 10·5)	258 (162 to 397)	34·5% (21·7 to 53·1)	−0·0187% (−5·61 to 5·10)
**UK**						
15–39 years	7460 (5310 to 8550)	69·5% (49·5 to 79·7)	−8·82% (−20·7 to 0·746)	8710 (7420 to 9320)	79·8% (68·0 to 85·4)	−4·80% (−9·99 to −1·24)
40–64 years	5700 (4150 to 8070)	52·9% (38·5 to 75·0)	3·24% (−4·76 to 11·0)	6470 (5220 to 8210)	61·5% (49·6 to 78·0)	4·11% (−2·38 to 10·5)
≥65 years	1300 (738 to 2040)	19·5% (11·1 to 30·6)	11·4% (6·19 to 16·5)	2000 (1320 to 3010)	35·6% (23·4 to 53·6)	10·9% (4·18 to 16·2)
**Andean Latin America**						
15–39 years	4470 (2630 to 6290)	34·4% (20·2 to 48·3)	6·25% (−4·55 to 35·1)	8990 (7150 to 9590)	68·9% (54·8 to 73·4)	4·58% (−1·39 to 23·9)
40–64 years	1290 (775 to 2050)	16·3% (9·83 to 26·0)	1·04% (−6·34 to 11·4)	3810 (3000 to 4880)	50·4% (39·6 to 64·5)	2·22% (−5·22 to 19·7)
≥65 years	101 (44·3 to 184)	3·94% (1·72 to 7·14)	0·521% (−2·51 to 2·93)	507 (343 to 682)	21·7% (14·7 to 29·2)	1·85% (−4·74 to 10·2)
**Bolivia**						
15–39 years	880 (477 to 1330)	36·4% (19·7 to 54·8)	7·20% (−5·61 to 38·1)	1670 (1270 to 1820)	68·3% (51·7 to 74·4)	5·02% (−3·00 to 25·7)
40–64 years	219 (124 to 378)	16·8% (9·45 to 28·9)	0·348% (−7·54 to 11·8)	641 (472 to 864)	50·2% (36·9 to 67·6)	0·532% (−7·86 to 19·0)
≥65 years	16·0 (5·78 to 32·7)	3·97% (1·44 to 8·13)	0·475% (−2·76 to 3·46)	85·3 (52·1 to 122)	24·3% (14·8 to 34·8)	1·27% (−7·05 to 11·2)
**Ecuador**						
15–39 years	598 (349 to 863)	16·7% (9·74 to 24·1)	4·40% (−2·05 to 16·7)	2040 (1630 to 2250)	57·3% (45·7 to 63·1)	5·54% (−1·59 to 21·4)
40–64 years	145 (87·4 to 236)	6·82% (4·11 to 11·1)	1·35% (−1·98 to 5·55)	820 (641 to 1060)	40·6% (31·8 to 52·6)	3·94% (−3·10 to 18·1)
≥65 years	7·20 (3·22 to 13·6)	1·02% (0·455 to 1·92)	0·435% (−0·199 to 1·15)	87·6 (58·5 to 119)	13·8% (9·23 to 18·7)	3·34% (−1·40 to 8·79)
**Peru**						
15–39 years	2990 (1770 to 4200)	42·7% (25·3 to 59·9)	7·52% (−6·70 to 42·9)	5280 (4220 to 5620)	75·0% (60·0 to 79·9)	4·55% (−2·32 to 23·7)
40–64 years	922 (549 to 1440)	20·7% (12·3 to 32·4)	1·39% (−8·86 to 14·2)	2350 (1820 to 2980)	55·0% (42·6 to 69·7)	2·20% (−6·80 to 20·3)
≥65 years	78·1 (32·4 to 145)	5·34% (2·21 to 9·92)	0·708% (−3·92 to 4·32)	334 (223 to 448)	24·8% (16·6 to 33·3)	1·40% (−7·37 to 11·7)
**Caribbean**						
15–39 years	2420 (1480 to 3350)	26·5% (16·2 to 36·7)	−3·25% (−8·37 to 1·90)	5990 (4910 to 6440)	66·1% (54·3 to 71·2)	0·677% (−3·93 to 4·05)
40–64 years	509 (292 to 777)	7·73% (4·43 to 11·8)	0·282% (−1·33 to 1·78)	2240 (1680 to 2930)	35·7% (26·8 to 46·7)	1·42% (−1·91 to 4·58)
≥65 years	31·5 (11·7 to 71·9)	1·25% (0·466 to 2·85)	0·361% (0·0367 to 0·773)	294 (182 to 470)	13·8% (8·55 to 22·1)	2·66% (0·448 to 4·54)
**Antigua and Barbuda**						
15–39 years	4·84 (2·91 to 7·26)	27·8% (16·7 to 41·6)	1·74% (−6·63 to 10·6)	11·3 (9·31 to 12·4)	66·4% (54·5 to 72·6)	6·43% (0·222 to 13·6)
40–64 years	1·31 (0·708 to 2·12)	8·63% (4·68 to 14·0)	5·01% (1·98 to 8·28)	5·16 (3·94 to 6·63)	37·4% (28·6 to 48·1)	16·3% (8·32 to 23·7)
≥65 years	0·0679 (0·0190 to 0·170)	1·45% (0·406 to 3·63)	1·23% (0·342 to 2·84)	0·644 (0·387 to 1·01)	15·8% (9·48 to 24·7)	11·8% (7·82 to 16·4)
**The Bahamas**						
15–39 years	20·8 (10·5 to 31·8)	26·5% (13·4 to 40·5)	−7·55% (−16·0 to 1·74)	48·5 (38·9 to 53·7)	64·7% (51·9 to 71·7)	−4·27% (−11·5 to 1·66)
40–64 years	4·44 (1·40 to 8·85)	7·11% (2·25 to 14·2)	−5·09% (−9·51 to −0·874)	18·9 (11·6 to 26·2)	33·3% (20·5 to 46·3)	−11·8% (−19·2 to −5·39)
≥65 years	0·192 (0·0200 to 0·602)	1·10% (0·115 to 3·47)	−0·967% (−2·44 to 0·0998)	1·71 (0·652 to 3·08)	12·7% (4·85 to 22·9)	−7·42% (−13·0 to −2·74)
**Barbados**						
15–39 years	16·1 (11·1 to 21·0)	32·2% (22·1 to 42·0)	1·27% (−6·35 to 8·66)	30·4 (27·0 to 32·8)	62·1% (55·2 to 67·0)	2·56% (−2·83 to 7·97)
40–64 years	6·81 (4·67 to 9·56)	12·5% (8·54 to 17·5)	2·50% (−0·834 to 5·94)	19·5 (16·3 to 22·8)	39·7% (33·1 to 46·5)	4·77% (−0·186 to 9·40)
≥65 years	0·770 (0·382 to 1·42)	2·94% (1·46 to 5·40)	1·44% (0·421 to 2·80)	4·24 (3·09 to 5·67)	20·2% (14·7 to 27·0)	7·09% (3·12 to 10·7)
**Belize**						
15–39 years	23·1 (14·4 to 32·5)	24·6% (15·3 to 34·6)	2·25% (−4·59 to 9·84)	53·9 (44·3 to 58·9)	59·5% (48·8 to 64·9)	2·76% (−3·71 to 8·14)
40–64 years	3·64 (2·06 to 5·62)	7·87% (4·46 to 12·2)	0·894% (−1·73 to 3·52)	16·1 (12·5 to 20·5)	35·7% (27·6 to 45·4)	0·832% (−3·91 to 5·48)
≥65 years	0·0957 (0·0310 to 0·220)	0·918% (0·297 to 2·11)	0·166% (−0·499 to 0·897)	1·43 (0·889 to 2·21)	12·9% (8·06 to 20·0)	0·726% (−2·93 to 3·90)
**Bermuda**						
15–39 years	3·69 (2·29 to 5·27)	40·8% (25·3 to 58·2)	−4·82% (−14·0 to 4·61)	7·13 (6·18 to 7·67)	81·3% (70·5 to 87·4)	−1·06% (−6·70 to 2·96)
40–64 years	1·88 (1·03 to 2·93)	15·1% (8·31 to 23·6)	−3·33% (−8·52 to 1·79)	6·12 (4·77 to 7·59)	50·1% (39·1 to 62·1)	−5·34% (−10·9 to 0·0248)
≥65 years	0·226 (0·0700 to 0·501)	3·11% (0·966 to 6·91)	−0·507% (−2·76 to 1·60)	1·30 (0·845 to 1·90)	24·0% (15·5 to 34·9)	−2·87% (−8·59 to 2·75)
**Cuba**						
15–39 years	475 (277 to 704)	27·1% (15·8 to 40·2)	−3·32% (−12·0 to 5·07)	1220 (997 to 1350)	65·9% (53·7 to 72·5)	1·12% (−4·95 to 6·88)
40–64 years	160 (83·2 to 259)	7·52% (3·92 to 12·2)	0·618% (−2·23 to 3·17)	713 (528 to 943)	34·9% (25·8 to 46·1)	2·73% (−2·38 to 7·07)
≥65 years	11·6 (3·51 to 28·4)	1·22% (0·367 to 2·98)	0·492% (−0·0380 to 1·26)	111 (67·5 to 177)	13·7% (8·35 to 21·9)	4·06% (1·02 to 7·12)
**Dominica**						
15–39 years	3·76 (2·22 to 5·51)	30·0 (17·7 to 44·0)	−5·14 (−13·8 to 3·57)	9·11 (7·49 to 9·92)	69·0 (56·7 to 75·1)	−0·620 (−7·20 to 4·66)
40–64 years	0·919 (0·462 to 1·51)	9·51% (4·78 to 15·6)	−1·26% (−5·20 to 2·59)	4·09 (3·02 to 5·32)	38·5% (28·4 to 50·1)	−3·52% (−8·66 to 2·13)
≥65 years	0·0736 (0·0200 to 0·187)	1·63% (0·444 to 4·16)	−0·00157% (−1·25 to 1·14)	0·624 (0·364 to 0·988)	16·8% (9·80 to 26·6)	0·258% (−4·24 to 5·00)
**Dominican Republic**						
15–39 years	752 (486 to 1000)	33·6% (21·7 to 44·8)	−5·14% (−13·0 to 2·45)	1580 (1280 to 1730)	68·8% (56·0 to 75·4)	−2·83% (−8·68 to 2·24)
40–64 years	131 (77·8 to 199)	9·98% (5·93 to 15·2)	0·130% (−3·04 to 3·16)	469 (336 to 639)	35·5% (25·4 to 48·3)	0·870% (−3·99 to 5·83)
≥65 years	6·30 (2·19 to 14·3)	1·49% (0·518 to 3·38)	0·438% (−0·333 to 1·30)	42·6 (21·9 to 77·1)	11·4% (5·85 to 20·6)	2·13% (−1·09 to 5·23)
**Grenada**						
15–39 years	4·75 (3·34 to 6·35)	24·4% (17·2 to 32·7)	−2·27% (−9·21 to 4·94)	11·2 (9·93 to 12·2)	53·0% (46·8 to 57·5)	−0·297% (−5·72 to 5·31)
40–64 years	1·38 (0·919 to 1·94)	9·24% (6·13 to 13·0)	1·26% (−1·78 to 4·31)	5·29 (4·48 to 6·16)	33·0% (28·0 to 38·5)	2·70% (−2·00 to 6·94)
≥65 years	0·143 (0·0681 to 0·260)	2·81% (1·34 to 5·11)	1·14% (−0·0665 to 2·58)	0·822 (0·617 to 1·07)	20·0% (15·0 to 26·0)	5·63% (1·36 to 9·57)
**Guyana**						
15–39 years	38·8 (20·7 to 58·0)	25·6% (13·6 to 38·2)	−3·72% (−11·4 to 5·77)	104 (87·4 to 112)	71·5% (60·0 to 76·9)	−1·73% (−8·59 to 3·14)
40–64 years	5·97 (2·75 to 10·3)	6·17% (2·84 to 10·6)	−3·55% (−7·02 to −0·676)	36·7 (27·7 to 47·3)	39·8% (30·1 to 51·3)	−9·77% (−14·0 to −5·51)
≥65 years	0·258 (0·0610 to 0·687)	0·963% (0·227 to 2·56)	−0·585% (−1·81 to 0·187)	3·26 (1·80 to 5·54)	14·8% (8·18 to 25·1)	−5·74% (−9·83 to −1·80)
**Haiti**						
15–39 years	567 (335 to 808)	20·1% (11·9 to 28·6)	1·78% (−4·61 to 8·33)	1710 (1410 to 1860)	65·6% (54·2 to 71·4)	4·43% (−1·64 to 9·95)
40–64 years	79·9 (43·2 to 127)	6·27% (3·39 to 9·95)	0·316% (−1·98 to 2·67)	450 (347 to 578)	39·5% (30·5 to 50·8)	2·44% (−1·80 to 6·65)
≥65 years	3·63 (1·25 to 8·52)	1·37% (0·471 to 3·20)	0·0818% (−0·829 to 0·936)	42·3 (26·7 to 65·0)	17·9% (11·3 to 27·5)	1·17% (−2·75 to 4·95)
**Jamaica**						
15–39 years	140 (76·1 to 218)	23·3% (12·7 to 36·3)	−1·52% (−9·45 to 5·74)	383 (297 to 423)	64·5% (50·1 to 71·1)	1·59% (−4·57 to 7·41)
40–64 years	17·1 (7·43 to 30·1)	4·53% (1·97 to 8·00)	0·125% (−2·04 to 2·06)	114 (79·3 to 160)	31·0% (21·5 to 43·3)	2·42% (−2·34 to 7·00)
≥65 years	0·693 (0·158 to 1·94)	0·490% (0·112 to 1·37)	0·123% (−0·213 to 0·539)	13·0 (6·81 to 24·0)	10·5% (5·47 to 19·3)	3·04% (0·232 to 5·70)
**Puerto Rico**						
15–39 years	157 (99·1 to 218)	28·7% (18·2 to 40·0)	0·768% (−5·98 to 7·79)	320 (272 to 346)	59·8% (50·9 to 64·8)	−1·40% (−7·06 to 3·62)
40–64 years	39·9 (22·1 to 61·8)	6·88% (3·82 to 10·7)	−0·0484% (−2·89 to 2·68)	167 (132 to 207)	32·9% (26·0 to 40·8)	−2·86% (−8·39 to 2·13)
≥65 years	3·27 (1·01 to 7·75)	0·829% (0·255 to 1·96)	0·106% (−0·555 to 0·676)	40·6 (26·7 to 59·9)	13·5% (8·87 to 19·9)	0·872% (−3·56 to 4·47)
**Saint Kitts and Nevis**						
15–39 years	2·54 (0·218 to 4·51)	22·4% (1·92 to 39·6)	−8·13% (−25·2 to 9·90)	5·59 (3·63 to 6·54)	49·3% (32·0 to 57·6)	−3·37% (−17·5 to 6·08)
40–64 years	0·589 (0 to 1·93)	5·88% (0 to 19·2)	−2·29% (−11·5 to 10·1)	2·02 (0·0120 to 4·00)	19·3% (0·114 to 38·1)	−6·10% (−26·7 to 20·8)
≥65 years	0·0352 (0 to 0·179)	1·25% (0 to 6·36)	−0·0265% (−2·85 to 4·42)	0·194 (0 to 0·554)	7·75% (0 to 22·1)	−1·95% (−13·3 to 11·6)
**Saint Lucia**						
15–39 years	14·5 (10·5 to 18·9)	44·2% (31·9 to 57·4)	−3·94% (−12·8 to 4·68)	23·3 (19·6 to 25·2)	68·6% (57·7 to 74·2)	−0·824% (−7·44 to 4·53)
40–64 years	5·99 (4·18 to 8·18)	19·8% (13·9 to 27·1)	−0·864% (−5·47 to 3·57)	12·7 (10·2 to 15·9)	42·3% (34·0 to 52·9)	−3·50% (−8·10 to 1·07)
≥65 years	0·427 (0·209 to 0·799)	3·96% (1·94 to 7·42)	−0·0651% (−1·83 to 1·84)	1·82 (1·20 to 2·63)	19·7% (13·0 to 28·5)	−1·70% (−5·62 to 2·46)
**Saint Vincent and the Grenadines**						
15–39 years	8·69 (5·03 to 12·5)	42·5% (24·6 to 61·4)	−0·562% (−12·0 to 10·4)	18·0 (15·1 to 19·3)	85·2% (71·5 to 91·3)	3·29% (−1·15 to 7·96)
40–64 years	2·39 (1·24 to 3·80)	14·2% (7·34 to 22·6)	5·68% (1·91 to 9·92)	8·75 (6·60 to 11·2)	48·3% (36·4 to 61·6)	11·0% (4·11 to 16·9)
≥65 years	0·146 (0·0410 to 0·373)	2·36% (0·664 to 6·05)	1·57% (0·385 to 3·62)	1·32 (0·792 to 2·08)	21·7% (13·0 to 34·2)	10·8% (6·45 to 15·0)
**Suriname**						
15–39 years	29·2 (15·5 to 44·3)	26·4% (14·0 to 40·0)	−5·53% (−13·7 to 3·16)	72·5 (58·2 to 79·7)	66·0% (53·0 to 72·6)	−1·62% (−8·43 to 3·58)
40–64 years	5·84 (2·68 to 10·1)	6·79% (3·11 to 11·7)	−1·80% (−5·23 to 1·06)	27·4 (19·5 to 37·6)	32·8% (23·4 to 45·0)	−4·15% (−9·46 to 0·947)
≥65 years	0·278 (0·0690 to 0·782)	0·977% (0·242 to 2·75)	−0·194% (−1·25 to 0·667)	2·69 (1·44 to 4·76)	12·2% (6·49 to 21·5)	−1·54% (−5·91 to 1·97)
**Trinidad and Tobago**						
15–39 years	71·0 (39·9 to 108)	28·5% (16·0 to 43·2)	−5·06% (−13·3 to 3·09)	167 (135 to 185)	65·3% (52·8 to 72·6)	−0·408% (−6·34 to 5·34)
40–64 years	20·6 (11·1 to 33·6)	9·43% (5·10 to 15·4)	−0·512% (−4·39 to 3·02)	81·8 (60·8 to 108)	36·7% (27·3 to 48·3)	0·564% (−4·50 to 5·23)
≥65 years	1·69 (0·497 to 4·29)	1·82% (0·534 to 4·62)	0·461% (−0·563 to 1·57)	13·0 (7·76 to 20·5)	16·1% (9·64 to 25·5)	3·12% (−0·733 to 6·71)
**Virgin Islands**						
15–39 years	4·88 (0·923 to 7·70)	31·2% (5·92 to 49·4)	−2·91% (−27·5 to 19·0)	10·1 (7·26 to 11·2)	69·7% (50·2 to 77·4)	2·23% (−12·1 to 18·5)
40–64 years	2·17 (0·00100 to 4·97)	11·7% (0·00541 to 26·9)	1·84% (−18·9 to 18·8)	5·69 (0·168 to 9·09)	35·2% (1·04 to 56·2)	4·99% (−37·0 to 41·9)
≥65 years	0·521 (0 to 1·58)	4·64% (0 to 14·1)	1·96% (−7·33 to 10·9)	1·46 (0·00200 to 3·06)	18·1% (0·0248 to 37·9)	6·19% (−21·8 to 28·3)
**Central Latin America**						
15–39 years	11 200 (6490 to 14 900)	21·3% (12·4 to 28·4)	−3·10% (−7·93 to 1·69)	28 300 (22 600 to 29 900)	55·6% (44·4 to 58·8)	−4·62% (−8·48 to −1·24)
40–64 years	1970 (1130 to 3070)	5·67% (3·25 to 8·82)	−1·46% (−4·00 to 0·714)	12 300 (10 300 to 15 300)	39·2% (32·8 to 48·6)	−7·33% (−11·0 to −3·71)
≥65 years	111 (40·8 to 254)	0·988% (0·362 to 2·26)	0·0384% (−0·539 to 0·493)	1410 (1000 to 2010)	15·0% (10·6 to 21·4)	−3·05% (−6·63 to 0·204)
**Colombia**						
15–39 years	2230 (1310 to 3040)	23·3% (13·6 to 31·7)	−1·43% (−7·84 to 4·88)	5060 (3990 to 5480)	52·8% (41·6 to 57·2)	−2·84% (−8·65 to 2·82)
40–64 years	385 (207 to 614)	5·48% (2·95 to 8·74)	−3·32% (−6·36 to −0·582)	1990 (1590 to 2560)	32·1% (25·6 to 41·3)	−9·89% (−14·6 to −5·14)
≥65 years	17·6 (4·68 to 47·2)	0·676% (0·180 to 1·81)	−0·513% (−1·58 to 0·133)	199 (117 to 326)	9·59% (5·67 to 15·7)	−6·61% (−11·2 to −2·81)
**Costa Rica**						
15–39 years	252 (125 to 374)	25·6% (12·8 to 38·1)	−3·41% (−11·1 to 4·30)	551 (413 to 604)	59·4% (44·5 to 65·1)	−2·83% (−10·1 to 3·50)
40–64 years	45·8 (20·9 to 83·1)	6·54% (2·99 to 11·8)	−3·70% (−8·02 to −0·400)	247 (190 to 331)	39·5% (30·4 to 53·0)	−11·0% (−16·1 to −5·99)
≥65 years	2·99 (0·672 to 8·62)	1·19% (0·267 to 3·43)	−0·659% (−2·46 to 0·300)	32·8 (19·5 to 54·3)	15·7% (9·33 to 26·0)	−8·47% (−13·8 to −3·63)
**El Salvador**						
15–39 years	107 (54·1 to 160)	7·84% (3·97 to 11·8)	−0·388% (−3·88 to 2·69)	658 (497 to 721)	53·7% (40·6 to 58·8)	−2·37% (−8·31 to 2·89)
40–64 years	18·6 (8·88 to 32·6)	2·19% (1·05 to 3·85)	0·236% (−1·06 to 1·31)	219 (172 to 290)	35·6% (27·9 to 47·0)	−3·49% (−9·84 to 2·46)
≥65 years	1·08 (0·266 to 2·87)	0·342% (0·0838 to 0·905)	0·142% (−0·0681 to 0·446)	26·2 (15·6 to 41·9)	11·2% (6·70 to 18·0)	−0·461% (−5·02 to 3·12)
**Guatemala**						
15–39 years	331 (164 to 495)	9·46% (4·69 to 14·1)	−0·715% (−4·42 to 2·82)	1420 (1090 to 1570)	43·9% (33·8 to 48·6)	−0·428% (−6·51 to 5·49)
40–64 years	43·4 (18·3 to 83·9)	2·72% (1·15 to 5·25)	−1·09% (−3·14 to 0·747)	390 (296 to 530)	28·0% (21·3 to 38·1)	−3·56% (−8·67 to 1·38)
≥65 years	2·40 (0·390 to 7·47)	0·505% (0·0821 to 1·57)	−0·136% (−0·902 to 0·410)	36·8 (19·5 to 63·1)	8·72% (4·61 to 14·9)	−3·21% (−7·14 to 0·478)
**Honduras**						
15–39 years	137 (82·1 to 197)	6·10% (3·64 to 8·75)	−0·291% (−2·77 to 2·05)	728 (591 to 815)	35·3% (28·7 to 39·6)	0·115% (−4·80 to 5·23)
40–64 years	24·6 (14·0 to 39·9)	2·49% (1·41 to 4·03)	0·251% (−0·944 to 1·41)	239 (195 to 298)	27·3% (22·3 to 34·1)	−1·24% (−5·94 to 3·27)
≥65 years	1·77 (0·619 to 3·79)	0·667% (0·233 to 1·43)	0·253% (−0·161 to 0·738)	25·7 (17·3 to 36·8)	11·1% (7·45 to 15·9)	0·119% (−3·42 to 3·33)
**Mexico**						
15–39 years	6570 (3930 to 8740)	25·2% (15·1 to 33·5)	−2·75% (−10·3 to 4·67)	15 000 (12 200 to 16 100)	59·7% (48·4 to 64·1)	−4·61% (−10·3 to 1·09)
40–64 years	1180 (659 to 1850)	6·50% (3·64 to 10·3)	−0·343% (−3·94 to 2·67)	7170 (6040 to 8670)	43·7% (36·8 to 52·8)	−5·88% (−11·7 to −0·361)
≥65 years	69·8 (25·6 to 150)	1·26% (0·463 to 2·71)	0·498% (−0·245 to 1·23)	868 (630 to 1170)	18·3% (13·3 to 24·7)	−0·0786% (−4·86 to 4·23)
**Nicaragua**						
15–39 years	254 (133 to 365)	18·0% (9·44 to 25·9)	−1·28% (−8·09 to 4·69)	781 (593 to 854)	55·3% (42·0 to 60·5)	−1·14% (−7·11 to 4·62)
40–64 years	36·0 (18·1 to 62·8)	4·79% (2·41 to 8·36)	0·851% (−1·69 to 2·94)	253 (204 to 323)	38·4% (30·9 to 48·9)	−1·55% (−8·40 to 4·42)
≥65 years	1·55 (0·396 to 4·02)	0·729% (0·186 to 1·88)	0·420% (0·0615 to 1·06)	23·6 (15·0 to 35·5)	13·6% (8·67 to 20·4)	1·01% (−3·57 to 5·08)
**Panama**						
15–39 years	148 (89·5 to 207)	19·1% (11·6 to 26·8)	−1·65% (−8·45 to 4·39)	450 (372 to 488)	57·4% (47·4 to 62·2)	−3·12% (−9·17 to 2·45)
40–64 years	34·0 (19·4 to 53·0)	6·33% (3·62 to 9·88)	−0·111% (−3·08 to 2·61)	226 (190 to 274)	42·5% (35·7 to 51·5)	−4·62% (−10·2 to 0·604)
≥65 years	2·47 (0·837 to 5·59)	1·26% (0·426 to 2·85)	0·418% (−0·382 to 1·24)	32·0 (22·9 to 44·6)	17·8% (12·8 to 24·9)	−0·900% (−5·86 to 3·86)
**Venezuela**						
15–39 years	1140 (609 to 1670)	17·7% (9·44 to 26·0)	−4·60% (−10·8 to 1·88)	3640 (2850 to 3980)	56·0% (43·8 to 61·3)	−6·80% (−13·7 to −1·22)
40–64 years	209 (97·9 to 364)	4·90% (2·30 to 8·53)	−4·34% (−7·73 to −1·20)	1580 (1250 to 2010)	38·1% (30·2 to 48·7)	−15·0% (−19·9 to −9·10)
≥65 years	11·6 (3·07 to 30·1)	0·822% (0·217 to 2·13)	−0·902% (−2·33 to 0·0288)	165 (105 to 251)	14·4% (9·13 to 21·8)	−10·9% (−15·8 to −6·24)
**Tropical Latin America**						
15–39 years	15 000 (10 400 to 18 000)	33·7% (23·4 to 40·5)	8·93% (3·06 to 14·4)	25 600 (19 000 to 27 300)	58·1% (43·2 to 61·9)	3·52% (−1·62 to 8·47)
40–64 years	4000 (2730 to 5650)	11·7% (8·00 to 16·6)	3·16% (0·355 to 6·02)	12 800 (10 600 to 15 700)	40·9% (33·8 to 50·2)	5·14% (0·613 to 9·15)
≥65 years	351 (168 to 638)	2·89% (1·39 to 5·26)	1·09% (0·0819 to 2·35)	1680 (1180 to 2310)	18·0% (12·7 to 24·9)	4·40% (1·01 to 7·31)
**Brazil**						
15–39 years	14 300 (10 000 to 17 200)	33·2% (23·3 to 39·9)	9·03% (3·24 to 14·5)	24 300 (18 100 to 26 000)	57·2% (42·5 to 61·2)	3·32% (−1·97 to 8·42)
40–64 years	3870 (2630 to 5470)	11·6% (7·90 to 16·4)	3·28% (0·385 to 6·16)	12 300 (10 200 to 15 100)	40·4% (33·4 to 49·6)	5·26% (0·707 to 9·31)
≥65 years	339 (163 to 617)	2·86% (1·37 to 5·19)	1·15% (0·125 to 2·38)	1620 (1140 to 2240)	17·9% (12·5 to 24·6)	4·62% (1·17 to 7·58)
**Paraguay**						
15–39 years	707 (402 to 882)	46·9% (26·7 to 58·5)	−0·650% (−9·47 to 7·72)	1280 (959 to 1340)	82·1% (61·4 to 86·0)	0·843% (−7·10 to 4·87)
40–64 years	127 (77·3 to 189)	16·1% (9·81 to 23·9)	−2·21% (−7·19 to 3·05)	454 (369 to 558)	57·3% (46·6 to 70·4)	−1·01% (−6·97 to 4·31)
≥65 years	11·7 (4·63 to 23·3)	4·63% (1·83 to 9·20)	−0·901% (−3·71 to 1·73)	56·5 (37·2 to 80·0)	25·9% (17·1 to 36·7)	−3·05% (−8·69 to 2·85)
**North Africa and Middle East**						
15–39 years	729 (433 to 1250)	0·585% (0·348 to 1·00)	−0·321% (−0·653 to −0·0790)	7800 (6060 to 9310)	5·81% (4·51 to 6·94)	−0·973% (−1·59 to −0·396)
40–64 years	242 (104 to 444)	0·344% (0·149 to 0·632)	−0·180% (−0·428 to −0·00198)	2820 (1810 to 4160)	3·66% (2·36 to 5·40)	−0·215% (−0·840 to 0·313)
≥65 years	18·5 (7·26 to 48·5)	0·111% (0·0433 to 0·289)	−0·0338% (−0·127 to 0·0314)	258 (166 to 446)	1·54% (0·989 to 2·66)	−0·0369% (−0·454 to 0·287)
**Afghanistan**						
15–39 years	12·6 (2·37 to 44·7)	0·144% (0·0270 to 0·509)	0·111% (0·00660 to 0·281)	278 (117 to 420)	2·99% (1·26 to 4·50)	2·21% (1·24 to 3·09)
40–64 years	0·673 (0·0160 to 2·87)	0·0226% (0·000538 to 0·0966)	0·0219% (0·000538 to 0·0952)	16·2 (1·73 to 49·3)	0·505% (0·0538 to 1·53)	0·496% (0·0538 to 1·49)
≥65 years	0·0124 (0 to 0·0821)	0·00247% (0 to 0·0164)	0·00221% (0 to 0·0164)	0·253 (0·00598 to 1·52)	0·0486% (0·00115 to 0·292)	0·0476% (0·00115 to 0·292)
**Algeria**						
15–39 years	37·1 (22·7 to 58·2)	0·439% (0·269 to 0·690)	0·0377% (−0·200 to 0·207)	542 (433 to 662)	6·28% (5·02 to 7·67)	1·12% (−0·0501 to 2·44)
40–64 years	13·0 (5·52 to 24·2)	0·246% (0·104 to 0·455)	0·0626% (−0·0746 to 0·189)	210 (147 to 285)	3·91% (2·74 to 5·31)	1·43% (0·475 to 2·32)
≥65 years	1·12 (0·384 to 2·71)	0·0834% (0·0285 to 0·201)	0·0267% (−0·0372 to 0·0829)	21·0 (13·5 to 32·0)	1·48% (0·951 to 2·25)	0·694% (0·133 to 1·18)
**Bahrain**						
15–39 years	1·06 (0·616 to 1·71)	0·433% (0·252 to 0·698)	−0·289% (−0·557 to −0·0604)	32·1 (25·7 to 39·0)	6·76% (5·43 to 8·23)	−2·73% (−4·53 to −0·983)
40–64 years	0·353 (0·148 to 0·664)	0·238% (0·0997 to 0·447)	−0·348% (−0·581 to −0·144)	13·2 (9·44 to 18·2)	4·41% (3·16 to 6·09)	−3·19% (−4·87 to −1·57)
≥65 years	0·0179 (0·00400 to 0·0470)	0·0795% (0·0178 to 0·209)	−0·203% (−0·420 to −0·0411)	0·490 (0·320 to 0·771)	1·79% (1·17 to 2·82)	−1·93% (−3·06 to −0·900)
**Egypt**						
15–39 years	34·9 (20·9 to 57·7)	0·172% (0·103 to 0·284)	−0·00651% (−0·0904 to 0·0726)	466 (352 to 570)	2·18% (1·65 to 2·68)	0·417% (−0·0498 to 0·880)
40–64 years	9·52 (3·51 to 18·2)	0·0927% (0·0342 to 0·177)	−0·00707% (−0·0769 to 0·0532)	150 (92·4 to 224)	1·33% (0·820 to 1·99)	0·372% (−0·0452 to 0·752)
≥65 years	0·518 (0·129 to 1·43)	0·0271% (0·00674 to 0·0748)	−0·00163% (−0·0361 to 0·0234)	10·7 (5·32 to 19·8)	0·462% (0·230 to 0·857)	0·194% (0·00879 to 0·420)
**Iran**						
15–39 years	76·2 (42·4 to 119)	0·440% (0·245 to 0·689)	0·402% (0·165 to 0·609)	1150 (912 to 1400)	6·40% (5·07 to 7·81)	5·28% (4·02 to 6·57)
40–64 years	29·3 (10·1 to 56·2)	0·252% (0·0866 to 0·483)	0·251% (0·0866 to 0·480)	478 (316 to 659)	4·01% (2·65 to 5·53)	4·00% (2·65 to 5·46)
≥65 years	2·53 (0·567 to 6·46)	0·0864% (0·0194 to 0·221)	0·0861% (0·0194 to 0·220)	42·3 (23·4 to 68·3)	1·48% (0·819 to 2·40)	1·48% (0·819 to 2·39)
**Iraq**						
15–39 years	40·5 (29·3 to 55·4)	0·576% (0·417 to 0·788)	−0·164% (−0·397 to 0·0560)	91·3 (75·9 to 110)	1·23% (1·02 to 1·49)	−0·330% (−0·633 to −0·00147)
40–64 years	19·0 (12·2 to 27·5)	0·483% (0·310 to 0·700)	−0·265% (−0·554 to −0·00862)	33·6 (26·9 to 41·5)	0·826% (0·662 to 1·02)	−0·337% (−0·630 to −0·0748)
≥65 years	2·12 (1·22 to 3·37)	0·241% (0·139 to 0·383)	−0·165% (−0·354 to −0·0108)	3·52 (2·61 to 4·68)	0·442% (0·328 to 0·587)	−0·182% (−0·359 to −0·0150)
**Jordan**						
15–39 years	2·83 (1·77 to 4·27)	0·116% (0·0725 to 0·175)	0·00369% (−0·0426 to 0·0520)	54·1 (43·3 to 64·9)	1·82% (1·46 to 2·18)	0·182% (−0·241 to 0·589)
40–64 years	1·19 (0·511 to 2·11)	0·0989% (0·0425 to 0·175)	−0·00937% (−0·0694 to 0·0492)	22·0 (14·7 to 30·2)	1·50% (1·00 to 2·06)	0·148% (−0·232 to 0·540)
≥65 years	0·0683 (0·0200 to 0·149)	0·0296% (0·00868 to 0·0646)	−0·00589% (−0·0394 to 0·0222)	2·32 (1·34 to 3·69)	0·958% (0·553 to 1·53)	0·0564% (−0·329 to 0·457)
**Kuwait**						
15–39 years	0·597 (0·00898 to 1·71)	0·0588% (0·000884 to 0·169)	0·0493% (0·0000985 to 0·123)	17·6 (4·32 to 27·7)	1·66% (0·407 to 2·62)	1·30% (0·331 to 1·98)
40–64 years	0·107 (0 to 0·421)	0·0174% (0 to 0·0685)	0·0171% (0 to 0·0685)	3·77 (0·0170 to 10·4)	0·474% (0·00213 to 1·30)	0·469% (0·00213 to 1·28)
≥65 years	0·00148 (0 to 0·00900)	0·00254% (0 to 0·0155)	0·00250% (0 to 0·0155)	0·0692 (0 to 0·330)	0·0725% (0 to 0·346)	0·0720% (0 to 0·346)
**Lebanon**						
15–39 years	26·7 (11·0 to 59·8)	3·01% (1·24 to 6·74)	−1·69% (−4·50 to 0·0732)	168 (124 to 208)	19·0% (14·0 to 23·4)	−5·26% (−9·02 to −1·59)
40–64 years	4·86 (0·746 to 13·8)	0·726% (0·111 to 2·06)	−0·544% (−1·61 to 0·124)	47·3 (24·7 to 79·0)	8·72% (4·57 to 14·6)	−4·61% (−7·75 to −1·52)
≥65 years	0·250 (0·0150 to 1·27)	0·0859% (0·00514 to 0·435)	−0·173% (−0·720 to 0·00429)	8·47 (3·82 to 18·3)	3·33% (1·50 to 7·20)	−3·53% (−6·96 to −1·22)
**Libya**						
15–39 years	1·82 (0·949 to 3·14)	0·125% (0·0649 to 0·215)	0·0867% (−0·0501 to 0·149)	55·5 (42·6 to 68·2)	3·55% (2·73 to 4·37)	2·32% (1·46 to 3·16)
40–64 years	0·379 (0·119 to 0·737)	0·0405% (0·0127 to 0·0788)	0·0395% (0·0127 to 0·0741)	21·2 (12·9 to 31·0)	2·10% (1·28 to 3·07)	2·05% (1·27 to 2·87)
≥65 years	0·0225 (0·00300 to 0·0650)	0·0133% (0·00178 to 0·0385)	0·0132% (0·00178 to 0·0385)	1·36 (0·642 to 2·39)	0·810% (0·382 to 1·42)	0·808% (0·382 to 1·40)
**Morocco**						
15–39 years	3·23 (0·591 to 10·7)	0·0444% (0·00811 to 0·147)	−0·0310% (−0·0709 to 0·00790)	419 (311 to 520)	5·69% (4·24 to 7·07)	−1·69% (−3·20 to −0·209)
40–64 years	0·272 (0·00900 to 1·10)	0·00556% (0·000184 to 0·0224)	−0·0160% (−0·0403 to −0·00202)	96·1 (62·3 to 136)	1·97% (1·28 to 2·79)	−0·550% (−1·25 to 0·0946)
≥65 years	0·00779 (0 to 0·0541)	0·000575% (0 to 0·00399)	−0·00418% (−0·0179 to −0·000105)	2·80 (1·54 to 5·00)	0·213% (0·117 to 0·380)	−0·0724% (−0·188 to 0·0395)
**Oman**						
15–39 years	1·96 (0·700 to 4·25)	0·261% (0·0930 to 0·565)	−0·0317% (−0·222 to 0·113)	68·5 (47·3 to 89·6)	4·94% (3·41 to 6·46)	0·246% (−0·887 to 1·22)
40–64 years	0·311 (0·0450 to 0·828)	0·0890% (0·0129 to 0·237)	−0·00643% (−0·115 to 0·0716)	15·2 (6·41 to 27·1)	2·17% (0·913 to 3·86)	0·408% (−0·405 to 1·18)
≥65 years	0·00821 (0 to 0·0370)	0·0162% (0 to 0·0731)	−0·00399% (−0·0536 to 0·0242)	0·343 (0·104 to 0·876)	0·530% (0·160 to 1·35)	0·143% (−0·229 to 0·474)
**Palestine**						
15–39 years	5·43 (3·45 to 8·15)	0·519% (0·330 to 0·779)	−0·100% (−0·389 to 0·148)	78·1 (64·8 to 92·6)	7·19% (5·96 to 8·51)	−0·319% (−1·89 to 1·27)
40–64 years	1·55 (0·765 to 2·58)	0·360% (0·177 to 0·598)	−0·118% (−0·440 to 0·173)	24·0 (17·9 to 30·7)	5·33% (3·99 to 6·83)	−0·418% (−2·06 to 1·30)
≥65 years	0·132 (0·0480 to 0·272)	0·146% (0·0533 to 0·302)	−0·0718% (−0·267 to 0·0878)	1·85 (1·22 to 2·61)	2·36% (1·56 to 3·33)	−0·253% (−1·37 to 0·859)
**Qatar**						
15–39 years	1·11 (0·495 to 2·23)	0·309% (0·137 to 0·619)	−0·0905% (−0·320 to 0·0707)	85·1 (63·7 to 108)	6·05% (4·53 to 7·68)	−0·192% (−1·60 to 1·16)
40–64 years	0·247 (0·0540 to 0·586)	0·139% (0·0304 to 0·330)	−0·0702% (−0·268 to 0·0563)	16·7 (9·41 to 26·1)	3·05% (1·71 to 4·75)	−0·475% (−1·70 to 0·612)
≥65 years	0·00343 (0 to 0·0140)	0·0304% (0 to 0·124)	−0·0220% (−0·188 to 0·0619)	0·327 (0·144 to 0·654)	1·02% (0·449 to 2·04)	−0·103% (−0·892 to 0·553)
**Saudi Arabia**						
15–39 years	8·09 (0·997 to 19·4)	0·107% (0·0131 to 0·256)	−0·0926% (−0·208 to −0·0148)	267 (136 to 372)	2·47% (1·26 to 3·43)	−0·682% (−1·31 to 0·0870)
40–64 years	0·842 (0·00700 to 2·61)	0·0220% (0·000183 to 0·0681)	−0·0225% (−0·0608 to −0·0000410)	55·8 (4·51 to 118)	0·905% (0·0732 to 1·91)	−0·593% (−1·09 to −0·132)
≥65 years	0·0183 (0 to 0·0930)	0·00493% (0 to 0·0251)	−0·00930% (−0·0297 to 0)	1·53 (0·0120 to 5·02)	0·256% (0·00201 to 0·841)	−0·306% (−0·652 to −0·0281)
**Sudan**						
15–39 years	2·13 (0 to 26·1)	0·0243% (0 to 0·297)	−0·593% (−0·815 to −0·362)	132 (0 to 324)	1·53% (0 to 3·78)	−10·7% (−13·4 to −8·07)
40–64 years	0·0183 (0 to 0·0210)	0·000561% (0 to 0·000645)	−0·533% (−0·804 to −0·303)	0·580 (0 to 1·85)	0·0175% (0 to 0·0558)	−11·8% (−14·2 to −9·49)
≥65 years	0·00114 (0 to 0)	0·000190% (0 to 0)	−0·246% (−0·413 to −0·111)	0·0214 (0 to 0)	0·00280% (0 to 0)	−6·68% (−8·73 to −5·09)
**Syria**						
15–39 years	29·3 (12·6 to 55·6)	0·557 (0·240 to 1·06)	−0·283 (−0·573 to 0·0548)	281 (209 to 349)	5·12 (3·80 to 6·36)	−1·17 (−2·47 to 0·214)
40–64 years	5·76 (1·06 to 13·2)	0·236% (0·0433 to 0·542)	−0·281% (−0·546 to −0·0633)	81·8 (38·3 to 133)	3·22% (1·51 to 5·25)	−2·35% (−3·51 to −1·23)
≥65 years	0·278 (0·0150 to 1·02)	0·0635% (0·00343 to 0·233)	−0·136% (−0·298 to −0·0262)	5·75 (1·67 to 11·9)	1·24% (0·361 to 2·58)	−1·62% (−2·59 to −0·788)
**Tunisia**						
15–39 years	3·22 (1·27 to 6·88)	0·145% (0·0571 to 0·309)	−0·00404% (−0·105 to 0·0657)	352 (294 to 414)	16·3% (13·6 to 19·1)	3·70% (1·07 to 6·38)
40–64 years	0·671 (0·104 to 1·93)	0·0374% (0·00580 to 0·108)	−0·000649% (−0·0440 to 0·0299)	154 (119 to 193)	8·82% (6·81 to 11·1)	2·16% (0·326 to 3·89)
≥65 years	0·0462 (0·00200 to 0·236)	0·00749% (0·000324 to 0·0382)	−0·00250% (−0·0266 to 0·00722)	18·4 (13·1 to 25·6)	3·18% (2·26 to 4·41)	0·757% (−0·245 to 1·67)
**Turkey**						
15–39 years	408 (225 to 685)	2·59% (1·43 to 4·35)	−0·968% (−2·47 to 0·245)	2770 (2110 to 3340)	17·0% (12·9 to 20·5)	−1·14% (−4·16 to 1·90)
40–64 years	147 (57·7 to 284)	1·20% (0·470 to 2·31)	−0·608% (−1·70 to 0·135)	1160 (699 to 1810)	9·39% (5·67 to 14·7)	0·262% (−2·32 to 2·45)
≥65 years	11·2 (3·29 to 31·0)	0·258% (0·0757 to 0·713)	−0·101% (−0·414 to 0·104)	130 (75·0 to 235)	3·65% (2·11 to 6·59)	0·412% (−1·25 to 1·58)
**United Arab Emirates**						
15–39 years	13·0 (3·24 to 36·3)	1·76% (0·437 to 4·88)	−1·78% (−4·19 to −0·231)	176 (114 to 246)	16·7% (10·9 to 23·4)	−5·29% (−8·96 to −1·62)
40–64 years	3·51 (0·209 to 13·0)	0·607% (0·0361 to 2·24)	−0·984% (−2·95 to 0·0116)	173 (72·1 to 324)	9·18% (3·83 to 17·2)	−5·52% (−10·2 to −1·23)
≥65 years	0·0376 (0 to 0·292)	0·0879% (0 to 0·683)	−0·244% (−1·14 to 0·0108)	3·76 (1·17 to 10·4)	3·26% (1·02 to 9·03)	−2·09% (−5·79 to 0·395)
**Yemen**						
15–39 years	19·0 (8·31 to 37·4)	0·284% (0·124 to 0·557)	−0·354% (−0·570 to −0·160)	307 (215 to 401)	4·54% (3·18 to 5·93)	−3·00% (−4·61 to −1·49)
40–64 years	2·64 (0·617 to 6·69)	0·109% (0·0255 to 0·276)	−0·396% (−0·621 to −0·213)	49·6 (23·3 to 86·4)	2·06% (0·967 to 3·58)	−3·88% (−5·11 to −2·73)
≥65 years	0·112 (0·0120 to 0·501)	0·0230% (0·00245 to 0·103)	−0·210% (−0·394 to −0·0950)	2·37 (0·853 to 5·64)	0·504% (0·181 to 1·20)	−2·38% (−3·27 to −1·66)
**South Asia**						
15–39 years	7260 (3860 to 10 800)	1·90% (1·01 to 2·81)	0·191% (−0·544 to 1·45)	92 400 (73 100 to 108 000)	23·2% (18·3 to 27·0)	4·63% (0·684 to 11·6)
40–64 years	3380 (1470 to 5310)	1·63% (0·708 to 2·56)	0·193% (−0·612 to 1·06)	41 500 (26 300 to 54 300)	19·6% (12·4 to 25·7)	4·76% (0·221 to 12·1)
≥65 years	370 (124 to 727)	0·612% (0·205 to 1·20)	0·0343% (−0·500 to 0·513)	5500 (2950 to 8390)	9·62% (5·16 to 14·7)	2·82% (−1·17 to 6·56)
**Bangladesh**						
15–39 years	214 (14·9 to 369)	0·602% (0·0417 to 1·04)	−0·0289% (−0·571 to 0·532)	2150 (1190 to 2750)	6·59% (3·64 to 8·42)	1·53% (−0·0384 to 3·84)
40–64 years	36·9 (0·0609 to 82·8)	0·192% (0·000317 to 0·431)	0·0981% (−0·0113 to 0·251)	508 (14·3 to 897)	2·77% (0·0782 to 4·89)	1·70% (0·0690 to 3·08)
≥65 years	2·18 (0 to 6·55)	0·0417% (0 to 0·125)	0·0273% (0 to 0·0873)	44·1 (0·0409 to 98·9)	0·740% (0·000686 to 1·66)	0·539% (0·000686 to 1·15)
**Bhutan**						
15–39 years	16·0 (2·39 to 31·7)	9·38% (1·40 to 18·6)	−2·87% (−8·84 to 6·36)	38·1 (16·9 to 55·0)	19·7% (8·73 to 28·4)	−8·24% (−16·3 to 2·13)
40–64 years	1·98 (0·0709 to 5·33)	2·48% (0·0886 to 6·66)	−6·38% (−10·8 to −0·462)	5·85 (0·568 to 13·3)	6·56% (0·637 to 14·9)	−16·0% (−22·5 to −4·46)
≥65 years	0·0816 (0 to 0·384)	0·335% (0 to 1·58)	−2·90% (−5·62 to −0·369)	0·272 (0·00400 to 1·11)	1·11% (0·0163 to 4·51)	−10·1% (−15·6 to −2·71)
**India**						
15–39 years	5390 (3070 to 7860)	1·85% (1·05 to 2·69)	0·0816% (−0·861 to 1·36)	79 900 (65 000 to 93 100)	25·7% (20·9 to 30·0)	5·24% (0·562 to 13·1)
40–64 years	2950 (1360 to 4640)	1·79% (0·828 to 2·81)	0·155% (−0·820 to 1·19)	38 800 (25 400 to 50 100)	23·0% (15·1 to 29·7)	5·63% (0·240 to 14·4)
≥65 years	349 (121 to 685)	0·696% (0·242 to 1·37)	−0·00355% (−0·670 to 0·578)	5320 (2890 to 8080)	11·6% (6·30 to 17·6)	2·88% (−2·22 to 7·56)
**Nepal**						
15–39 years	579 (229 to 875)	8·05% (3·18 to 12·2)	3·17% (0·0598 to 6·53)	1990 (1310 to 2400)	32·8% (21·7 to 39·7)	9·75% (0·599 to 20·8)
40–64 years	198 (46·9 to 352)	5·72% (1·36 to 10·2)	4·51% (1·27 to 7·46)	655 (250 to 1010)	21·9% (8·35 to 33·7)	17·9% (8·10 to 24·5)
≥65 years	12·4 (1·09 to 31·4)	1·26% (0·110 to 3·18)	1·15% (0·109 to 2·77)	68·7 (13·1 to 142)	7·72% (1·47 to 15·9)	7·17% (1·47 to 13·6)
**Pakistan**						
15–39 years	1060 (299 to 1900)	2·20% (0·621 to 3·96)	0·515% (−0·509 to 2·13)	8370 (4600 to 11 300)	17·1% (9·38 to 23·0)	1·75% (−2·43 to 8·60)
40–64 years	196 (28·4 to 425)	0·981% (0·142 to 2·12)	0·218% (−0·348 to 0·886)	1560 (353 to 2960)	7·37% (1·66 to 14·0)	1·32% (−1·47 to 4·63)
≥65 years	6·01 (0·404 to 20·1)	0·149% (0·00999 to 0·498)	0·00294% (−0·196 to 0·215)	63·2 (6·21 to 190)	1·44% (0·142 to 4·33)	0·0807% (−1·22 to 1·39)
**East Asia**						
15–39 years	12 400 (7510 to 20 800)	4·92% (2·98 to 8·27)	−0·560% (−3·15 to 1·95)	123 000 (98 400 to 145 000)	45·7% (36·5 to 53·9)	0·912% (−4·30 to 5·80)
40–64 years	10 400 (6560 to 15 500)	3·86% (2·43 to 5·73)	−0·770% (−2·74 to 1·08)	118 000 (99 300 to 140 000)	42·1% (35·4 to 49·7)	−1·61% (−7·42 to 3·76)
≥65 years	1700 (767 to 3050)	1·65% (0·745 to 2·97)	−0·741% (−1·95 to 0·434)	21 900 (16 300 to 27 800)	24·2% (18·0 to 30·6)	−1·23% (−7·00 to 4·22)
**China**						
15–39 years	12 000 (7230 to 20 100)	4·91% (2·97 to 8·25)	−0·522% (−3·12 to 2·03)	119 000 (95 600 to 141 000)	45·8% (36·7 to 53·9)	1·13% (−4·12 to 6·14)
40–64 years	10 100 (6360 to 15 100)	3·88% (2·43 to 5·79)	−0·734% (−2·75 to 1·17)	115 000 (96 900 to 136 000)	42·4% (35·6 to 50·1)	−1·34% (−7·37 to 4·16)
≥65 years	1670 (754 to 2980)	1·69% (0·761 to 3·01)	−0·721% (−1·97 to 0·488)	21 500 (16 000 to 27 200)	24·4% (18·2 to 31·0)	−0·993% (−6·87 to 4·60)
**North Korea**						
15–39 years	215 (105 to 418)	4·49% (2·19 to 8·74)	−1·25% (−4·66 to 1·51)	2180 (1550 to 2710)	41·4% (29·3 to 51·4)	−4·17% (−10·1 to 1·97)
40–64 years	128 (68·9 to 216)	2·89% (1·55 to 4·88)	−1·31% (−3·29 to 0·290)	1460 (1120 to 1850)	33·3% (25·6 to 42·2)	−8·12% (−14·6 to −2·25)
≥65 years	13·7 (4·33 to 29·7)	0·799% (0·253 to 1·73)	−1·08% (−2·23 to −0·236)	154 (102 to 214)	16·3% (10·8 to 22·6)	−6·96% (−13·0 to −1·47)
**Taiwan (province of China)**						
15–39 years	239 (129 to 423)	6·33% (3·43 to 11·2)	−1·96% (−5·39 to 1·57)	1790 (1350 to 2190)	45·0% (33·9 to 55·2)	−6·15% (−13·6 to 1·01)
40–64 years	179 (105 to 284)	3·86 (2·26 to 6·11)	−2·45 (−4·90 to −0·346)	1460 (1150 to 1810)	32·9 (26·0 to 40·7)	−11·9 (−17·6 to −6·52)
≥65 years	17·8 (6·10 to 36·4)	0·861% (0·294 to 1·76)	−1·51% (−2·71 to −0·527)	252 (178 to 343)	14·5% (10·2 to 19·7)	−10·4% (−15·5 to −4·98)
**Oceania**						
15–39 years	113 (17·3 to 192)	4·14% (0·634 to 7·05)	−0·838% (−2·90 to 1·48)	668 (456 to 822)	23·6% (16·1 to 29·0)	−3·23% (−7·19 to 0·759)
40–64 years	10·1 (1·74 to 22·2)	0·786% (0·135 to 1·72)	−0·174% (−0·718 to 0·267)	105 (35·4 to 181)	7·63% (2·58 to 13·2)	−1·60% (−4·34 to 0·891)
≥65 years	0·353 (0·0629 to 0·988)	0·151% (0·0269 to 0·423)	−0·0696% (−0·298 to 0·0577)	7·97 (2·61 to 16·6)	3·20% (1·05 to 6·65)	−0·680% (−2·42 to 0·631)
**American Samoa**						
15–39 years	0·718 (0·00200 to 1·82)	6·77% (0·0189 to 17·2)	−1·22% (−8·07 to 5·43)	2·91 (0·928 to 4·28)	27·5% (8·76 to 40·4)	−2·43% (−12·8 to 8·95)
40–64 years	0·00950 (0 to 0·0521)	0·133% (0 to 0·730)	−0·200% (−1·82 to 0·520)	0·132 (0 to 0·550)	1·88% (0 to 7·84)	−1·43% (−10·5 to 3·80)
≥65 years	0·000109 (0 to 0·00100)	0·00587% (0 to 0·0539)	−0·0238% (−0·264 to 0·0539)	0·00651 (0 to 0·0391)	0·360% (0 to 2·16)	−0·513% (−4·71 to 1·36)
**Cook Islands**						
15–39 years	1·04 (0·439 to 1·39)	33·2% (14·0 to 44·2)	15·1% (0·663 to 31·2)	1·77 (1·49 to 1·94)	64·6% (54·3 to 70·9)	25·5% (11·1 to 48·2)
40–64 years	0·383 (0·188 to 0·592)	13·2% (6·47 to 20·3)	11·6% (5·61 to 17·6)	1·20 (0·842 to 1·50)	42·8% (30·1 to 53·8)	37·1% (24·9 to 48·9)
≥65 years	0·0466 (0·0190 to 0·0842)	3·94% (1·61 to 7·12)	3·69% (1·44 to 6·70)	0·287 (0·178 to 0·395)	25·9% (16·1 to 35·6)	24·1% (15·1 to 33·2)
**Federated States of Micronesia**						
15–39 years	2·10 (0·128 to 4·00)	10·2% (0·623 to 19·4)	−3·07% (−9·27 to 4·02)	8·29 (5·12 to 10·6)	38·0% (23·5 to 48·7)	−6·71% (−15·3 to 0·785)
40–64 years	0·129 (0·00700 to 0·389)	1·06% (0·0576 to 3·20)	−1·66% (−4·24 to 0·0605)	1·35 (0·267 to 2·78)	10·9% (2·15 to 22·4)	−9·93% (−17·4 to −1·59)
≥65 years	0·00297 (0 to 0·0140)	0·116% (0 to 0·549)	−0·416% (−1·52 to 0·0392)	0·0905 (0·0140 to 0·234)	4·46% (0·690 to 11·5)	−5·82% (−12·2 to −0·392)
**Fiji**						
15–39 years	11·4 (2·32 to 19·3)	6·55% (1·33 to 11·0)	1·13% (−1·69 to 4·24)	72·7 (51·3 to 87·4)	39·8% (28·1 to 47·8)	2·74% (−2·97 to 8·46)
40–64 years	1·55 (0·266 to 3·41)	1·34% (0·229 to 2·93)	0·312% (−0·545 to 1·28)	16·5 (6·33 to 27·2)	13·8% (5·26 to 22·6)	1·82% (−2·55 to 5·89)
≥65 years	0·0584 (0·00600 to 0·182)	0·190% (0·0195 to 0·592)	0·0403% (−0·201 to 0·274)	1·47 (0·449 to 2·96)	6·00% (1·84 to 12·1)	1·23% (−1·92 to 4·07)
**Guam**						
15–39 years	5·23 (0·316 to 9·69)	18·2% (1·10 to 33·8)	3·10% (−9·72 to 16·2)	16·0 (9·72 to 20·2)	49·9% (30·4 to 63·3)	4·96% (−7·75 to 24·2)
40–64 years	0·525 (0·00200 to 1·86)	2·28% (0·00869 to 8·09)	1·32% (−2·40 to 6·71)	4·44 (0·327 to 9·38)	17·9% (1·32 to 37·8)	8·09% (−8·53 to 26·9)
≥65 years	0·0225 (0 to 0·128)	0·269% (0 to 1·53)	0·166% (−0·443 to 1·31)	0·602 (0·0170 to 1·62)	8·38% (0·237 to 22·5)	4·65% (−5·13 to 17·5)
**Kiribati**						
15–39 years	1·02 (0·0550 to 2·15)	4·04% (0·219 to 8·53)	−1·12% (−4·10 to 1·87)	5·23 (2·34 to 7·58)	21·8% (9·75 to 31·6)	−6·67% (−13·3 to −0·299)
40–64 years	0·0642 (0 to 0·255)	0·501% (0 to 1·99)	−0·527% (−1·85 to 0·172)	0·401 (0·00200 to 1·28)	3·49% (0·0174 to 11·2)	−4·63% (−10·6 to −0·0262)
≥65 years	0·00219 (0 to 0·0140)	0·0781% (0 to 0·500)	−0·161% (−0·826 to 0·114)	0·0172 (0 to 0·0772)	1·03% (0 to 4·61)	−2·14% (−6·31 to 0·0599)
**Marshall Islands**						
15–39 years	0·474 (0·0620 to 0·810)	4·14% (0·541 to 7·07)	0·416% (−1·78 to 2·65)	3·91 (2·59 to 4·80)	32·1% (21·3 to 39·4)	1·55% (−5·18 to 7·74)
40–64 years	0·0456 (0·00500 to 0·112)	0·737% (0·0808 to 1·81)	0·103% (−0·796 to 0·902)	0·717 (0·216 to 1·25)	11·4% (3·45 to 19·9)	1·27% (−4·74 to 6·83)
≥65 years	0·00151 (0 to 0·00603)	0·143% (0 to 0·571)	0·0269% (−0·408 to 0·475)	0·0585 (0·0150 to 0·126)	5·12% (1·31 to 11·1)	1·22% (−2·73 to 5·21)
**Nauru**						
15–39 years	0·365 (0·0551 to 0·595)	16·0% (2·42 to 26·1)	−0·0133% (−7·50 to 8·75)	1·16 (0·843 to 1·36)	49·5% (36·1 to 58·2)	1·61% (−5·40 to 10·6)
40–64 years	0·0364 (0·00502 to 0·0792)	3·59% (0·496 to 7·82)	0·477% (−3·51 to 3·93)	0·231 (0·0984 to 0·360)	24·1% (10·3 to 37·7)	4·13% (−7·25 to 14·4)
≥65 years	0·00156 (0 to 0·00609)	0·673% (0 to 2·62)	0·0504% (−2·33 to 1·94)	0·0207 (0·00616 to 0·0401)	12·5% (3·73 to 24·2)	2·15% (−8·08 to 11·1)
**Niue**						
15–39 years	0·0602 (0·00709 to 0·101)	21·9% (2·57 to 36·8)	0·382% (−10·8 to 12·3)	0·132 (0·0959 to 0·159)	45·9% (33·2 to 54·9)	1·91% (−6·50 to 12·9)
40–64 years	0·0130 (0 to 0·0321)	5·05% (0 to 12·5)	0·781% (−5·73 to 6·99)	0·0642 (0·0192 to 0·108)	23·7% (7·12 to 40·1)	3·54% (−10·5 to 17·6)
≥65 years	0·000706 (0 to 0·00313)	0·639% (0 to 2·83)	0·0969% (−2·31 to 2·05)	0·00871 (0·00104 to 0·0179)	11·5% (1·37 to 23·6)	2·65% (−9·47 to 14·1)
**Northern Mariana Islands**						
15–39 years	0·795 (0·0489 to 1·50)	12·2% (0·751 to 23·0)	2·04% (−5·80 to 10·7)	3·01 (1·76 to 3·71)	42·1% (24·6 to 51·9)	4·58% (−8·59 to 21·0)
40–64 years	0·131 (0 to 0·428)	1·57% (0 to 5·13)	−0·0752% (−3·98 to 3·20)	1·22 (0·0509 to 2·64)	13·8% (0·574 to 29·8)	0·988% (−16·3 to 17·1)
≥65 years	0·00586 (0 to 0·0300)	0·331% (0 to 1·70)	0·0594% (−1·19 to 1·25)	0·128 (0·00100 to 0·339)	7·09% (0·0556 to 18·8)	1·81% (−8·23 to 11·8)
**Palau**						
15–39 years	0·441 (0·0320 to 0·852)	18·0% (1·31 to 34·7)	−0·784% (−12·8 to 10·2)	1·64 (0·825 to 2·22)	46·3% (23·3 to 62·7)	−0·290% (−13·7 to 12·8)
40–64 years	0·117 (0·00100 to 0·375)	3·57% (0·0305 to 11·4)	0·0146% (−7·43 to 6·34)	0·595 (0·0420 to 1·41)	15·3% (1·08 to 36·1)	2·08% (−15·1 to 16·9)
≥65 years	0·00576 (0 to 0·0281)	0·671% (0 to 3·27)	0·0213% (−2·49 to 2·33)	0·0548 (0·00201 to 0·163)	7·29% (0·267 to 21·7)	1·53% (−9·04 to 12·7)
**Papua New Guinea**						
15–39 years	67·3 (9·27 to 116)	3·28% (0·452 to 5·67)	−0·471% (−2·27 to 1·46)	442 (304 to 554)	20·6% (14·2 to 25·9)	−2·09% (−6·49 to 2·32)
40–64 years	5·90 (0·864 to 13·3)	0·648% (0·0948 to 1·46)	−0·295% (−0·913 to 0·188)	63·0 (20·2 to 110)	6·41% (2·06 to 11·2)	−2·26% (−4·94 to 0·291)
≥65 years	0·164 (0·0130 to 0·518)	0·114% (0·00901 to 0·360)	−0·131% (−0·420 to 0·0244)	4·15 (1·15 to 8·79)	2·44% (0·680 to 5·18)	−1·23% (−3·16 to 0·242)
**Samoa**						
15–39 years	1·58 (0·194 to 2·80)	4·14% (0·508 to 7·35)	−0·588% (−2·95 to 2·00)	11·0 (7·15 to 14·1)	27·4% (17·8 to 35·0)	−3·49% (−8·90 to 2·30)
40–64 years	0·0965 (0·00898 to 0·238)	0·487% (0·0453 to 1·20)	−0·294% (−0·969 to 0·170)	2·48 (0·682 to 4·56)	11·3% (3·12 to 20·9)	−4·52% (−10·1 to 0·572)
≥65 years	0·00267 (0 to 0·0100)	0·0471% (0 to 0·176)	−0·0755% (−0·300 to 0·0542)	0·225 (0·0469 to 0·505)	4·38% (0·914 to 9·84)	−3·21% (−7·32 to 0·205)
**Solomon Islands**						
15–39 years	9·03 (0·206 to 19·7)	6·76% (0·154 to 14·7)	−0·794% (−6·63 to 4·10)	42·8 (22·0 to 58·0)	31·8% (16·4 to 43·2)	1·32% (−5·85 to 9·09)
40–64 years	0·217 (0·00300 to 0·759)	0·356% (0·00494 to 1·25)	0·155% (−0·186 to 0·751)	3·32 (0·253 to 8·44)	5·31% (0·404 to 13·5)	2·47% (0·0805 to 6·81)
≥65 years	0·00234 (0 to 0·0150)	0·0211% (0 to 0·136)	0·00919% (−0·0444 to 0·0904)	0·138 (0·00698 to 0·500)	1·23% (0·0624 to 4·48)	0·609% (−0·274 to 2·36)
**Tokelau**						
15–39 years	0·0280 (0·00304 to 0·0514)	11·5% (1·24 to 21·1)	0·0876% (−7·02 to 7·73)	0·107 (0·0702 to 0·133)	42·1% (27·6 to 52·2)	1·67% (−7·47 to 10·5)
40–64 years	0·00320 (0 to 0·0102)	1·96% (0 to 6·25)	0·541% (−2·73 to 4·36)	0·0286 (0·00607 to 0·0539)	16·8% (3·57 to 31·7)	4·90% (−5·86 to 16·7)
≥65 years	0·000243 (0 to 0·00207)	0·322% (0 to 2·74)	0·00820% (−2·02 to 2·70)	0·00505 (0 to 0·0127)	7·94% (0 to 20·0)	2·38% (−8·04 to 13·3)
**Tonga**						
15–39 years	0·762 (0·0959 to 1·40)	3·84% (0·483 to 7·04)	−0·444% (−2·89 to 1·89)	3·22 (1·89 to 4·26)	17·0% (9·98 to 22·4)	−2·95% (−7·63 to 1·86)
40–64 years	0·0869 (0·00600 to 0·237)	0·791% (0·0546 to 2·16)	−0·200% (−1·21 to 0·827)	0·451 (0·0510 to 1·02)	4·28% (0·484 to 9·68)	−1·44% (−5·16 to 1·88)
≥65 years	0·00670 (0 to 0·0251)	0·183% (0 to 0·686)	−0·150% (−0·707 to 0·240)	0·0494 (0·00300 to 0·143)	1·67% (0·102 to 4·84)	−1·05% (−3·72 to 1·08)
**Tuvalu**						
15–39 years	0·233 (0·0120 to 0·484)	10·2% (0·529 to 21·3)	0·0379% (−6·43 to 7·01)	1·06 (0·632 to 1·36)	40·6% (24·1 to 52·0)	0·275% (−7·84 to 8·87)
40–64 years	0·0128 (0 to 0·0431)	0·942% (0 to 3·17)	−0·104% (−1·79 to 1·26)	0·147 (0·0240 to 0·323)	10·3% (1·69 to 22·7)	0·843% (−5·46 to 6·69)
≥65 years	0·000548 (0 to 0·00402)	0·111% (0 to 0·815)	−0·0387% (−0·748 to 0·496)	0·0146 (0·00101 to 0·0423)	3·89% (0·269 to 11·3)	−0·250% (−5·88 to 3·88)
**Vanuatu**						
15–39 years	4·67 (0·680 to 7·95)	7·57% (1·10 to 12·9)	−0·633% (−4·93 to 3·82)	20·3 (14·4 to 24·8)	33·7% (23·9 to 41·1)	0·238% (−5·42 to 6·74)
40–64 years	0·307 (0·0450 to 0·718)	1·07% (0·157 to 2·51)	0·0336% (−0·937 to 0·878)	3·38 (1·20 to 5·69)	11·6% (4·13 to 19·6)	1·15% (−3·53 to 5·20)
≥65 years	0·0114 (0 to 0·0381)	0·190% (0 to 0·631)	−0·00162% (−0·375 to 0·324)	0·280 (0·0860 to 0·576)	4·80% (1·48 to 9·89)	0·594% (−2·36 to 3·19)
**Southeast Asia**						
15–39 years	7610 (2560 to 12 100)	5·55% (1·87 to 8·86)	1·18% (−0·0755 to 2·85)	49 500 (39 000 to 56 500)	35·0% (27·6 to 39·9)	4·52% (1·91 to 10·4)
40–64 years	2760 (1590 to 3980)	2·80% (1·61 to 4·04)	0·330% (−0·439 to 1·19)	23 600 (18 400 to 28 200)	24·7% (19·2 to 29·4)	8·49% (5·81 to 11·2)
≥65 years	327 (167 to 511)	1·19% (0·607 to 1·86)	0·139% (−0·322 to 0·560)	3120 (2040 to 3950)	14·3% (9·37 to 18·2)	6·61% (4·49 to 8·43)
**Cambodia**						
15–39 years	649 (294 to 936)	18·2% (8·26 to 26·3)	8·22% (2·69 to 13·9)	1580 (1340 to 1770)	43·7% (37·2 to 49·2)	11·7% (5·56 to 22·8)
40–64 years	241 (150 to 335)	12·2% (7·65 to 17·0)	8·68% (5·37 to 12·2)	590 (489 to 674)	34·5% (28·6 to 39·5)	20·1% (12·3 to 27·1)
≥65 years	25·4 (13·2 to 40·8)	4·56% (2·38 to 7·33)	3·61% (1·82 to 5·88)	69·4 (50·4 to 86·2)	20·4% (14·8 to 25·3)	14·7% (10·0 to 19·3)
**Indonesia**						
15–39 years	375 (72·7 to 725)	0·679% (0·132 to 1·31)	−0·0957% (−0·476 to 0·348)	4430 (2430 to 6020)	7·68% (4·21 to 10·4)	0·206% (−1·71 to 2·36)
40–64 years	140 (30·2 to 283)	0·358% (0·0772 to 0·724)	−0·122% (−0·391 to 0·130)	1230 (422 to 2010)	3·12% (1·07 to 5·11)	−0·560% (−1·64 to 0·574)
≥65 years	12·4 (1·26 to 29·8)	0·138% (0·0140 to 0·332)	−0·0898% (−0·259 to 0·0398)	83·7 (13·7 to 173)	1·05% (0·172 to 2·17)	−0·394% (−1·08 to 0·245)
**Laos**						
15–39 years	459 (214 to 649)	29·1% (13·6 to 41·2)	3·42% (−5·69 to 17·5)	1060 (829 to 1210)	66·1% (51·7 to 75·5)	2·90% (−3·39 to 15·9)
40–64 years	108 (63·6 to 154)	14·3% (8·44 to 20·4)	2·52% (−3·87 to 9·10)	348 (252 to 430)	46·6% (33·8 to 57·7)	9·47% (−1·04 to 22·2)
≥65 years	7·13 (2·92 to 12·3)	4·29% (1·76 to 7·41)	1·09% (−1·95 to 3·93)	37·4 (19·9 to 53·4)	25·3% (13·5 to 36·1)	7·30% (−1·34 to 16·3)
**Malaysia**						
15–39 years	224 (76·9 to 369)	3·42% (1·17 to 5·62)	−0·699% (−2·43 to 1·38)	1130 (760 to 1400)	15·6% (10·5 to 19·3)	−4·03% (−7·33 to −0·266)
40–64 years	74·6 (32·6 to 124)	1·97% (0·861 to 3·28)	−1·40% (−2·90 to 0·116)	344 (200 to 483)	8·63% (5·02 to 12·1)	−5·60% (−8·50 to −2·50)
≥65 years	9·24 (3·09 to 17·9)	0·810% (0·271 to 1·57)	−1·08% (−2·15 to −0·216)	39·2 (16·2 to 63·4)	3·59% (1·49 to 5·81)	−3·61% (−6·01 to −1·04)
**Maldives**						
15–39 years	1·88 (0·0716 to 5·30)	2·13% (0·0810 to 5·99)	−0·947% (−4·03 to 1·53)	42·0 (16·6 to 66·6)	25·0% (9·87 to 39·7)	0·412% (−6·97 to 9·11)
40–64 years	0·210 (0·00700 to 0·706)	0·430% (0·0143 to 1·45)	0·0295% (−1·10 to 0·840)	7·65 (1·93 to 15·5)	10·0% (2·53 to 20·3)	4·73% (−2·32 to 12·1)
≥65 years	0·00702 (0 to 0·0330)	0·0737% (0 to 0·347)	−0·0218% (−0·461 to 0·207)	0·245 (0·0170 to 0·676)	2·35% (0·163 to 6·50)	0·961% (−2·62 to 4·47)
**Mauritius**						
15–39 years	19·8 (1·65 to 49·7)	8·67% (0·722 to 21·8)	−4·64% (−12·0 to 3·01)	121 (80·9 to 151)	51·9% (34·7 to 64·7)	−6·02% (−12·8 to 4·47)
40–64 years	5·13 (0·813 to 12·4)	2·30% (0·364 to 5·55)	−2·15% (−6·40 to 0·800)	69·2 (39·3 to 97·2)	31·4% (17·9 to 44·2)	−7·35% (−15·1 to 3·35)
≥65 years	0·501 (0·0360 to 1·71)	0·564% (0·0404 to 1·92)	−0·762% (−2·77 to 0·224)	12·0 (4·47 to 19·3)	17·6% (6·55 to 28·2)	−5·92% (−13·6 to 3·42)
**Myanmar**						
15–39 years	175 (35·9 to 333)	1·53% (0·314 to 2·92)	0·720% (−0·0487 to 1·39)	4010 (3190 to 4650)	36·7% (29·2 to 42·5)	12·6% (5·95 to 21·9)
40–64 years	40·3 (10·8 to 84·7)	0·525% (0·140 to 1·10)	0·460% (0·129 to 0·912)	1770 (1370 to 2140)	26·7% (20·8 to 32·4)	18·8% (12·8 to 23·7)
≥65 years	1·82 (0·338 to 4·39)	0·0846% (0·0157 to 0·204)	0·0791% (0·0154 to 0·188)	191 (116 to 259)	12·6% (7·66 to 17·1)	10·6% (6·96 to 14·4)
**Philippines**						
15–39 years	3910 (1520 to 5960)	17·2% (6·65 to 26·1)	−1·10% (−7·59 to 8·78)	15 900 (12 700 to 18 100)	66·8% (53·1 to 75·8)	−1·85% (−7·52 to 6·18)
40–64 years	1330 (773 to 1900)	10·6% (6·17 to 15·1)	−0·952% (−5·45 to 4·44)	6240 (4760 to 7540)	48·9% (37·3 to 59·1)	0·0388% (−6·26 to 10·9)
≥65 years	165 (81·9 to 266)	4·84% (2·40 to 7·80)	−0·530% (−3·79 to 2·07)	671 (408 to 897)	26·5% (16·1 to 35·5)	1·36% (−4·89 to 9·48)
**Seychelles**						
15–39 years	0·815 (0·233 to 1·34)	4·73% (1·35 to 7·78)	−0·0942% (−2·37 to 2·61)	8·90 (7·12 to 10·3)	41·9% (33·5 to 48·7)	−0·523% (−6·45 to 6·81)
40–64 years	0·401 (0·172 to 0·677)	2·50% (1·07 to 4·23)	−0·314% (−1·83 to 1·24)	5·25 (3·94 to 6·45)	29·2% (21·9 to 35·8)	0·300% (−5·51 to 7·10)
≥65 years	0·0412 (0·0130 to 0·0821)	0·871% (0·275 to 1·74)	−0·244% (−1·17 to 0·536)	0·556 (0·332 to 0·769)	14·4% (8·63 to 20·0)	−0·0913% (−5·14 to 5·10)
**Sri Lanka**						
15–39 years	39·5 (16·4 to 59·9)	0·958% (0·399 to 1·45)	0·0624% (−0·430 to 0·602)	932 (790 to 1070)	23·7% (20·1 to 27·1)	1·61% (−2·45 to 6·44)
40–64 years	27·8 (18·3 to 38·6)	0·809% (0·533 to 1·12)	0·147% (−0·203 to 0·519)	715 (615 to 817)	22·9% (19·6 to 26·1)	5·06% (0·797 to 9·65)
≥65 years	6·48 (3·92 to 9·35)	0·480% (0·290 to 0·692)	0·0850% (−0·191 to 0·328)	141 (107 to 175)	14·0% (10·7 to 17·4)	4·05% (0·424 to 8·04)
**Thailand**						
15–39 years	679 (269 to 1010)	5·62% (2·22 to 8·32)	0·262% (−2·06 to 3·12)	5610 (4810 to 6280)	46·5% (39·9 to 52·1)	−0·106% (−6·09 to 6·57)
40–64 years	484 (284 to 692)	3·49% (2·04 to 4·99)	−0·234% (−1·88 to 1·36)	4500 (3730 to 5190)	34·7% (28·9 to 40·1)	−0·323% (−5·29 to 5·79)
≥65 years	71·5 (36·7 to 112)	1·38% (0·710 to 2·16)	−0·287% (−1·31 to 0·546)	754 (559 to 957)	18·5% (13·7 to 23·5)	−0·826% (−5·72 to 4·57)
**Timor-Leste**						
15–39 years	4·87 (1·56 to 8·32)	1·74% (0·558 to 2·97)	0·0953% (−0·880 to 1·06)	66·9 (51·8 to 77·6)	24·3% (18·8 to 28·2)	1·47% (−3·35 to 7·57)
40–64 years	1·78 (0·860 to 2·87)	1·63% (0·787 to 2·63)	0·426% (−0·403 to 1·21)	26·9 (20·1 to 33·3)	22·9% (17·1 to 28·3)	6·06% (1·16 to 11·3)
≥65 years	0·326 (0·119 to 0·591)	0·856% (0·313 to 1·55)	0·265% (−0·334 to 0·871)	4·54 (2·68 to 6·11)	13·0% (7·68 to 17·5)	4·56% (0·833 to 8·61)
**Vietnam**						
15–39 years	1070 (104 to 2410)	5·65% (0·550 to 12·8)	3·70% (0·0489 to 6·70)	14 500 (11 600 to 16 500)	73·7% (58·8 to 83·8)	23·6% (10·1 to 48·4)
40–64 years	300 (74·9 to 636)	2·03% (0·507 to 4·30)	1·98% (0·506 to 3·93)	7750 (5860 to 9310)	55·0% (41·6 to 66·1)	48·9% (36·3 to 54·9)
≥65 years	26·4 (4·86 to 62·4)	0·602% (0·111 to 1·42)	0·599% (0·111 to 1·39)	1110 (691 to 1440)	38·1% (23·7 to 49·4)	36·7% (23·6 to 45·3)
**Central sub-Saharan Africa**						
15–39 years	4950 (110 to 6840)	18·7% (0·416 to 25·8)	−3·66% (−9·61 to 7·70)	11 000 (7330 to 12 300)	41·9% (27·8 to 46·7)	−1·97% (−6·62 to 9·22)
40–64 years	1200 (100 to 2010)	11·9% (0·992 to 19·9)	−0·869% (−5·39 to 4·06)	3280 (1110 to 4730)	33·0% (11·2 to 47·6)	0·429% (−6·28 to 9·82)
≥65 years	100 (14·6 to 194)	5·00% (0·731 to 9·71)	−0·944% (−4·60 to 1·92)	262 (88·2 to 405)	18·1% (6·07 to 27·9)	−0·935% (−7·68 to 5·97)
**Angola**						
15–39 years	938 (64·3 to 1270)	15·3% (1·05 to 20·8)	1·31% (−4·13 to 8·39)	2290 (1840 to 2530)	41·2% (33·0 to 45·4)	3·47% (−2·30 to 16·3)
40–64 years	313 (64·7 to 457)	13·4% (2·78 to 19·6)	3·74% (−0·707 to 7·68)	872 (513 to 1060)	42·2% (24·8 to 51·5)	11·1% (2·97 to 21·4)
≥65 years	20·9 (6·43 to 35·5)	5·11% (1·57 to 8·66)	1·78% (−0·746 to 4·21)	75·5 (42·7 to 99·2)	24·2% (13·7 to 31·8)	8·96% (3·14 to 14·4)
**Central African Republic**						
15–39 years	240 (0·948 to 360)	18·3% (0·0720 to 27·3)	−5·73% (−14·2 to 7·10)	514 (310 to 593)	40·5% (24·4 to 46·8)	−5·50% (−11·7 to 4·77)
40–64 years	46·0 (1·06 to 88·2)	9·87% (0·228 to 18·9)	−4·53% (−10·6 to 1·65)	132 (24·1 to 210)	27·7% (5·06 to 44·1)	−7·74% (−15·5 to 2·27)
≥65 years	3·03 (0·127 to 7·00)	3·98% (0·166 to 9·19)	−3·12% (−8·32 to 0·722)	7·05 (0·948 to 13·1)	13·1% (1·77 to 24·4)	−9·37% (−17·8 to 0·313)
**Congo (Brazzaville)**						
15–39 years	461 (13·0 to 614)	41·4% (1·17 to 55·2)	1·83% (−9·08 to 19·3)	613 (436 to 679)	58·4% (41·6 to 64·7)	3·86% (−3·12 to 15·6)
40–64 years	114 (9·85 to 182)	22·7% (1·96 to 36·1)	5·26% (−1·96 to 12·5)	237 (101 to 326)	44·4% (18·9 to 61·1)	8·51% (−0·203 to 20·4)
≥65 years	9·95 (1·55 to 18·7)	10·7% (1·67 to 20·1)	3·27% (−2·18 to 8·77)	22·8 (7·87 to 33·8)	29·2% (10·1 to 43·3)	7·70% (−0·645 to 16·3)
**Democratic Republic of the Congo**						
15–39 years	3170 (8·39 to 4640)	18·3% (0·0485 to 26·9)	−4·87% (−12·8 to 7·51)	7280 (4390 to 8340)	41·0% (24·7 to 47·0)	−3·50% (−9·62 to 6·66)
40–64 years	680 (17·6 to 1250)	10·4% (0·270 to 19·1)	−2·46% (−8·33 to 3·05)	1920 (397 to 2990)	29·1% (6·00 to 45·2)	−2·98% (−11·1 to 7·17)
≥65 years	60·0 (3·43 to 137)	4·42% (0·253 to 10·0)	−1·59% (−6·33 to 2·11)	142 (22·3 to 250)	14·8% (2·32 to 26·0)	−4·00% (−12·5 to 4·31)
**Equatorial Guinea**						
15–39 years	61·2 (3·67 to 84·9)	20·8% (1·25 to 28·9)	−2·12% (−10·8 to 9·54)	173 (139 to 192)	46·1% (37·2 to 51·1)	2·59% (−4·50 to 16·4)
40–64 years	16·9 (2·92 to 25·5)	16·5% (2·85 to 24·9)	3·91% (−3·01 to 10·4)	37·5 (20·1 to 47·1)	43·7% (23·5 to 54·9)	13·0% (2·66 to 25·1)
≥65 years	1·85 (0·513 to 3·17)	9·51% (2·63 to 16·3)	3·92% (−1·16 to 8·66)	4·03 (1·92 to 5·48)	29·8% (14·2 to 40·5)	12·2% (3·12 to 20·8)
**Gabon**						
15–39 years	82·4 (5·92 to 112)	21·1% (1·52 to 28·7)	−4·78% (−12·9 to 7·76)	165 (131 to 182)	48·0% (38·1 to 52·9)	−3·77% (−9·78 to 4·73)
40–64 years	29·6 (5·46 to 44·1)	17·2% (3·17 to 25·6)	−2·79% (−9·58 to 4·64)	76·6 (42·2 to 93·8)	44·8% (24·7 to 54·9)	−3·07% (−9·76 to 6·50)
≥65 years	4·16 (1·17 to 6·81)	10·3% (2·89 to 16·9)	−2·47% (−8·50 to 2·99)	11·1 (5·88 to 14·3)	32·9% (17·4 to 42·3)	−4·05% (−11·8 to 6·07)
**Eastern sub-Saharan Africa**						
15–39 years	12 800 (2610 to 19 800)	14·4% (2·93 to 22·3)	0·00659% (−5·55 to 11·1)	31 200 (19 900 to 35 500)	36·8% (23·5 to 41·8)	1·13% (−3·78 to 18·9)
40–64 years	3280 (1440 to 5280)	10·9% (4·76 to 17·5)	0·923% (−3·16 to 5·68)	9360 (5420 to 13 000)	31·8% (18·4 to 44·2)	3·22% (−2·82 to 14·5)
≥65 years	372 (184 to 558)	5·98% (2·96 to 8·97)	0·864% (−0·731 to 2·48)	1150 (705 to 1490)	21·2% (13·0 to 27·5)	3·18% (−0·226 to 7·80)
**Burundi**						
15–39 years	244 (62·3 to 362)	10·0% (2·55 to 14·8)	−1·74% (−6·69 to 5·84)	891 (629 to 1020)	39·1% (27·6 to 44·7)	−6·31% (−12·5 to 6·43)
40–64 years	67·4 (29·6 to 104)	8·86% (3·89 to 13·7)	−3·20% (−7·77 to 2·31)	315 (204 to 391)	37·4% (24·3 to 46·5)	−10·2% (−17·0 to 0·683)
≥65 years	6·35 (2·76 to 10·3)	4·12% (1·79 to 6·71)	−2·94% (−6·11 to 0·220)	40·4 (26·5 to 52·2)	24·2% (15·8 to 31·2)	−9·85% (−16·1 to −2·42)
**Comoros**						
15–39 years	4·29 (1·43 to 6·96)	2·87% (0·958 to 4·66)	−0·300% (−2·90 to 2·57)	13·2 (8·41 to 16·3)	8·73% (5·56 to 10·8)	0·559% (−2·61 to 5·06)
40–64 years	0·609 (0·195 to 1·05)	0·794% (0·254 to 1·37)	0·0975% (−0·533 to 0·676)	3·17 (1·41 to 4·50)	4·23% (1·88 to 6·00)	1·54% (−0·0562 to 3·54)
≥65 years	0·111 (0·0300 to 0·208)	0·503% (0·135 to 0·940)	0·140% (−0·322 to 0·500)	0·448 (0·155 to 0·707)	2·65% (0·918 to 4·19)	1·26% (0·0471 to 2·57)
**Djibouti**						
15–39 years	13·0 (0·0120 to 36·6)	5·32% (0·00491 to 15·0)	−3·44% (−9·02 to 3·91)	61·9 (11·4 to 93·2)	22·7% (4·21 to 34·3)	−7·53% (−16·3 to 11·4)
40–64 years	1·61 (0·0190 to 6·57)	1·44% (0·0170 to 5·87)	−3·84% (−8·60 to 0·180)	8·57 (0·167 to 31·0)	6·42% (0·125 to 23·2)	−14·4% (−23·6 to 0·0189)
≥65 years	0·0378 (0 to 0·165)	0·209% (0 to 0·914)	−1·67% (−4·22 to −0·0104)	0·204 (0·00400 to 0·745)	0·994% (0·0195 to 3·63)	−8·53% (−15·7 to −0·373)
**Eritrea**						
15–39 years	191 (1·76 to 480)	12·2% (0·112 to 30·5)	−1·31% (−10·2 to 12·1)	567 (269 to 714)	34·8% (16·5 to 43·8)	1·01% (−6·47 to 19·1)
40–64 years	19·0 (0·148 to 90·1)	2·90% (0·0226 to 13·7)	−1·66% (−7·97 to 2·35)	125 (27·7 to 233)	20·4% (4·52 to 37·9)	−3·70% (−12·0 to 7·61)
≥65 years	0·550 (0·00198 to 2·40)	0·443% (0·00159 to 1·93)	−0·662% (−3·07 to 0·249)	7·18 (1·32 to 13·8)	9·19% (1·69 to 17·7)	−4·30% (−11·3 to 2·49)
**Ethiopia**						
15–39 years	5860 (734 to 9310)	25·9% (3·25 to 41·2)	3·08% (−8·38 to 20·8)	10 600 (6560 to 12 200)	47·9% (29·7 to 55·4)	5·41% (−3·89 to 29·0)
40–64 years	1220 (312 to 2150)	17·0% (4·35 to 30·0)	4·98% (−2·98 to 12·9)	2700 (1260 to 3910)	37·0% (17·3 to 53·7)	10·2% (−0·315 to 24·7)
≥65 years	123 (31·2 to 224)	8·16% (2·07 to 14·9)	3·17% (−0·951 to 7·31)	362 (161 to 527)	22·4% (9·92 to 32·6)	7·84% (0·327 to 14·8)
**Kenya**						
15–39 years	754 (155 to 1150)	6·96% (1·43 to 10·6)	0·0298% (−3·04 to 4·95)	4300 (2570 to 5000)	41·5% (24·9 to 48·3)	2·65% (−3·71 to 18·5)
40–64 years	279 (134 to 425)	7·20% (3·47 to 11·0)	−1·64% (−5·18 to 2·55)	1700 (987 to 2260)	43·0% (25·0 to 57·3)	−2·56% (−10·2 to 13·7)
≥65 years	50·4 (21·0 to 80·3)	5·60% (2·33 to 8·92)	−0·711% (−3·75 to 2·18)	219 (126 to 288)	30·3% (17·4 to 39·8)	−2·12% (−8·70 to 6·12)
**Madagascar**						
15–39 years	516 (32·8 to 921)	8·83% (0·563 to 15·8)	−1·40% (−6·90 to 6·63)	1760 (891 to 2170)	31·7% (16·1 to 39·2)	−2·33% (−9·59 to 16·4)
40–64 years	122 (24·2 to 255)	5·58% (1·11 to 11·7)	−1·87% (−6·46 to 2·72)	485 (166 to 792)	23·1% (7·90 to 37·8)	−4·95% (−12·3 to 9·04)
≥65 years	7·84 (1·46 to 17·2)	2·21% (0·412 to 4·85)	−1·18% (−3·75 to 1·02)	35·1 (11·8 to 57·6)	11·1% (3·73 to 18·1)	−4·58% (−11·2 to 2·69)
**Malawi**						
15–39 years	112 (31·5 to 170)	2·72% (0·764 to 4·12)	0·307% (−1·04 to 2·10)	1050 (750 to 1220)	27·7% (19·8 to 32·2)	5·12% (0·0193 to 17·4)
40–64 years	43·8 (20·3 to 68·2)	3·45% (1·60 to 5·36)	0·426% (−1·36 to 2·31)	336 (217 to 427)	27·3% (17·6 to 34·7)	5·44% (−0·756 to 15·8)
≥65 years	7·74 (3·29 to 12·8)	2·47% (1·05 to 4·09)	0·0339% (−1·71 to 1·64)	34·9 (21·1 to 46·4)	15·4% (9·33 to 20·5)	2·54% (−2·57 to 8·10)
**Mozambique**						
15–39 years	1120 (30·4 to 2100)	18·2% (0·492 to 33·9)	3·01% (−8·39 to 17·6)	2550 (1210 to 3140)	47·1% (22·4 to 58·0)	7·07% (−3·80 to 29·1)
40–64 years	180 (24·5 to 423)	8·78% (1·19 to 20·6)	4·24% (−0·980 to 9·75)	542 (134 to 968)	29·8% (7·35 to 53·3)	13·4% (0·708 to 24·5)
≥65 years	9·92 (1·24 to 24·3)	2·27% (0·285 to 5·56)	1·56% (0·107 to 3·84)	44·5 (13·1 to 79·5)	13·9% (4·10 to 24·8)	8·84% (2·60 to 15·5)
**Rwanda**						
15–39 years	688 (182 to 1010)	24·3% (6·43 to 35·6)	−5·29% (−15·3 to 15·3)	1400 (1090 to 1590)	51·2% (39·6 to 57·9)	−6·67% (−14·5 to 17·6)
40–64 years	204 (93·8 to 306)	17·7% (8·13 to 26·5)	−4·11% (−11·8 to 7·91)	433 (297 to 525)	44·9% (30·8 to 54·5)	−7·40% (−15·0 to 13·2)
≥65 years	19·5 (8·60 to 31·7)	7·39% (3·27 to 12·1)	−2·20% (−7·48 to 2·46)	49·7 (33·0 to 62·7)	28·3% (18·8 to 35·7)	−8·01% (−15·2 to 3·09)
**Somalia**						
15–39 years	127 (0 to 569)	2·50% (0 to 11·2)	0·120% (−5·31 to 6·09)	495 (0 to 919)	9·01% (0 to 16·7)	0·903% (−3·80 to 8·31)
40–64 years	1·96 (0 to 0·00100)	0·118% (0 to 0·0000599)	−0·0693% (0 to 0)	9·96 (0 to 185)	0·599% (0 to 11·1)	−0·0297% (−2·41 to 0·00395)
≥65 years	0·0127 (0 to 0)	0·00491% (0 to 0)	−0·00252% (0 to 0)	0·0141 (0 to 0)	0·00829% (0 to 0)	−0·0152% (0 to 0)
**South Sudan**						
15–39 years	132 (0·0785 to 341)	5·51% (0·00327 to 14·2)	−0·912% (−6·27 to 5·91)	541 (108 to 786)	22·1% (4·43 to 32·2)	−1·20% (−9·10 to 13·3)
40–64 years	12·0 (0·0958 to 51·0)	1·49% (0·0119 to 6·31)	−0·676% (−4·07 to 1·67)	51·7 (0·482 to 197)	6·23% (0·0581 to 23·8)	−2·25% (−10·1 to 4·94)
≥65 years	0·259 (0·00100 to 1·26)	0·228% (0·000881 to 1·11)	−0·190% (−1·22 to 0·372)	1·23 (0·00998 to 4·97)	0·917% (0·00746 to 3·72)	−1·16% (−4·95 to 0·702)
**Tanzania**						
15–39 years	1030 (508 to 1370)	8·60% (4·25 to 11·4)	−0·286% (−3·89 to 5·28)	2170 (1700 to 2510)	20·2% (15·8 to 23·4)	0·103% (−4·36 to 7·96)
40–64 years	566 (390 to 730)	12·8% (8·81 to 16·5)	0·609% (−4·05 to 6·29)	1140 (879 to 1330)	27·5% (21·3 to 32·3)	1·69% (−3·71 to 10·3)
≥65 years	87·4 (58·2 to 116)	9·40% (6·25 to 12·5)	1·05% (−2·88 to 4·54)	204 (154 to 246)	24·0% (18·2 to 29·0)	2·90% (−2·11 to 9·22)
**Uganda**						
15–39 years	1480 (588 to 2030)	17·2% (6·81 to 23·6)	−2·76% (−12·9 to 13·1)	3170 (2560 to 3560)	39·5% (31·9 to 44·3)	−3·59% (−12·0 to 20·3)
40–64 years	410 (249 to 549)	15·3% (9·26 to 20·4)	1·01% (−6·16 to 10·2)	981 (776 to 1140)	41·0% (32·4 to 47·5)	2·61% (−6·69 to 22·5)
≥65 years	43·6 (26·5 to 64·1)	7·57% (4·61 to 11·1)	1·85% (−2·04 to 5·21)	105 (78·5 to 125)	25·7% (19·3 to 30·7)	4·57% (−2·66 to 14·0)
**Zambia**						
15–39 years	547 (109 to 822)	13·6% (2·71 to 20·4)	−1·47% (−8·62 to 9·52)	1610 (1140 to 1820)	42·2% (30·0 to 47·9)	−1·41% (−8·91 to 21·4)
40–64 years	148 (59·2 to 232)	11·8% (4·74 to 18·5)	−1·34% (−7·80 to 6·43)	526 (326 to 657)	39·5% (24·5 to 49·3)	1·57% (−7·03 to 19·5)
≥65 years	14·9 (5·85 to 24·7)	6·05% (2·37 to 10·0)	−0·643% (−4·72 to 2·87)	48·4 (30·6 to 62·3)	23·7% (15·0 to 30·5)	1·29% (−5·31 to 10·4)
**Southern sub-Saharan Africa**						
15–39 years	2680 (476 to 3350)	15·8% (2·80 to 19·7)	0·718% (−3·09 to 4·30)	7940 (7070 to 8460)	47·1% (41·9 to 50·1)	1·39% (−2·54 to 5·40)
40–64 years	1100 (681 to 1430)	12·0% (7·49 to 15·7)	−2·46% (−5·68 to 0·784)	3400 (2240 to 3940)	42·1% (27·7 to 48·8)	−3·07% (−7·67 to 1·85)
≥65 years	166 (110 to 221)	6·44% (4·27 to 8·57)	−1·68% (−4·15 to 0·589)	437 (283 to 542)	25·8% (16·7 to 31·9)	−1·92% (−6·07 to 2·73)
**Botswana**						
15–39 years	77·2 (8·89 to 103)	14·5% (1·67 to 19·3)	−0·0329% (−4·97 to 4·61)	198 (173 to 218)	37·5% (32·9 to 41·4)	2·15% (−3·17 to 7·19)
40–64 years	21·8 (11·1 to 30·4)	8·40% (4·29 to 11·7)	0·0552% (−2·99 to 3·08)	73·6 (43·9 to 89·9)	30·2% (18·0 to 36·9)	0·425% (−5·25 to 6·03)
≥65 years	1·61 (0·807 to 2·59)	2·70% (1·36 to 4·35)	−0·297% (−2·06 to 1·42)	5·63 (3·24 to 7·66)	13·8% (7·92 to 18·8)	−0·856% (−5·20 to 3·62)
**Eswatini**						
15–39 years	18·0 (3·24 to 23·7)	6·90% (1·24 to 9·08)	−0·370% (−2·98 to 1·82)	67·3 (59·2 to 74·8)	27·5% (24·2 to 30·5)	1·97% (−2·30 to 6·54)
40–64 years	6·49 (3·80 to 8·67)	6·57% (3·84 to 8·76)	−1·38% (−4·28 to 1·19)	23·9 (17·2 to 28·0)	26·8% (19·3 to 31·5)	−1·44% (−6·35 to 4·27)
≥65 years	1·34 (0·847 to 1·89)	5·09% (3·21 to 7·19)	−1·54% (−4·37 to 0·997)	2·68 (1·94 to 3·33)	18·1% (13·1 to 22·4)	−0·297% (−5·11 to 4·46)
**Lesotho**						
15–39 years	83·5 (3·16 to 113)	20·0% (0·759 to 27·1)	−1·30% (−8·48 to 4·48)	202 (169 to 220)	49·2% (41·4 to 53·6)	2·87% (−2·63 to 8·45)
40–64 years	21·1 (7·27 to 32·8)	12·0% (4·13 to 18·6)	−0·947% (−5·77 to 3·69)	61·0 (25·5 to 77·3)	41·6% (17·4 to 52·7)	0·264% (−7·01 to 8·37)
≥65 years	2·08 (0·729 to 3·79)	3·64% (1·28 to 6·63)	0·0748% (−2·30 to 2·47)	6·21 (2·41 to 9·29)	20·2% (7·85 to 30·3)	1·46% (−5·62 to 8·40)
**Namibia**						
15–39 years	141 (23·2 to 180)	26·8% (4·40 to 34·1)	−1·90% (−10·3 to 5·90)	265 (236 to 287)	52·6% (46·9 to 56·9)	2·46% (−3·62 to 8·70)
40–64 years	53·8 (33·1 to 71·4)	22·6% (13·9 to 30·0)	4·00% (−4·19 to 11·8)	92·6 (54·8 to 111)	44·2% (26·1 to 53·0)	7·04% (−2·07 to 19·1)
≥65 years	9·06 (4·97 to 13·7)	13·7% (7·52 to 20·7)	5·07% (−1·62 to 11·7)	12·3 (6·74 to 16·2)	28·4% (15·6 to 37·4)	9·28% (−0·537 to 18·4)
**South Africa**						
15–39 years	2150 (424 to 2740)	18·0% (3·54 to 22·9)	2·37% (−2·50 to 7·36)	5800 (5210 to 6270)	47·4% (42·6 to 51·3)	3·68% (−1·68 to 8·72)
40–64 years	931 (599 to 1220)	13·2% (8·51 to 17·3)	−2·66% (−6·68 to 1·21)	2680 (1880 to 3090)	42·7% (30·0 to 49·2)	−3·30% (−8·99 to 2·60)
≥65 years	143 (96·1 to 193)	6·91% (4·64 to 9·31)	−2·14% (−5·03 to 0·576)	366 (247 to 446)	26·8% (18·1 to 32·7)	−2·45% (−7·29 to 2·50)
**Zimbabwe**						
15–39 years	210 (8·03 to 298)	6·38% (0·244 to 9·07)	−4·42% (−8·05 to 0·000822)	1410 (1200 to 1540)	47·6% (40·5 to 51·8)	−7·83% (−13·4 to −2·02)
40–64 years	61·1 (22·0 to 95·0)	4·76% (1·72 to 7·40)	−3·79% (−7·75 to −0·0770)	468 (200 to 590)	42·1% (17·9 to 53·1)	−4·31% (−11·5 to 5·58)
≥65 years	8·91 (3·35 to 16·3)	2·95% (1·11 to 5·41)	−1·41% (−4·24 to 0·831)	45·0 (18·2 to 65·5)	22·2% (8·99 to 32·4)	−3·21% (−11·0 to 5·42)
**Western sub-Saharan Africa**						
15–39 years	11 000 (6610 to 16 500)	11·4% (6·83 to 17·1)	−0·675% (−4·29 to 5·43)	27 700 (21 700 to 30 400)	31·5% (24·6 to 34·6)	0·576% (−2·91 to 10·2)
40–64 years	4050 (2730 to 5620)	11·5% (7·73 to 15·9)	0·584% (−2·35 to 4·22)	9890 (7470 to 12 100)	30·5% (23·0 to 37·3)	2·34% (−2·21 to 10·5)
≥65 years	470 (250 to 694)	6·74% (3·59 to 9·95)	−0·154% (−2·84 to 2·52)	1360 (918 to 1710)	21·2% (14·3 to 26·5)	1·14% (−2·88 to 6·32)
**Benin**						
15–39 years	351 (150 to 593)	13·4% (5·72 to 22·6)	−1·40% (−8·57 to 8·17)	765 (530 to 903)	31·4% (21·8 to 37·0)	−0·863% (−7·20 to 14·7)
40–64 years	94·1 (40·2 to 160)	10·5% (4·47 to 17·8)	1·60% (−3·92 to 6·55)	216 (120 to 303)	25·8% (14·4 to 36·2)	2·05% (−6·34 to 13·2)
≥65 years	4·68 (0·679 to 10·2)	2·38% (0·345 to 5·19)	0·245% (−1·97 to 2·35)	18·7 (7·31 to 29·8)	11·5% (4·49 to 18·3)	1·62% (−3·80 to 7·19)
**Burkina Faso**						
15–39 years	505 (351 to 680)	11·2% (7·80 to 15·1)	−0·510% (−5·17 to 6·11)	1330 (1130 to 1510)	34·2% (29·0 to 38·7)	−3·74% (−9·95 to 6·50)
40–64 years	175 (123 to 230)	11·5% (8·10 to 15·1)	1·37% (−3·24 to 5·70)	430 (340 to 512)	30·2% (23·9 to 35·9)	−2·96% (−10·1 to 9·86)
≥65 years	18·5 (9·89 to 29·0)	5·15% (2·75 to 8·04)	0·483% (−2·35 to 3·04)	97·4 (66·6 to 121)	30·4% (20·8 to 37·7)	0·326% (−7·58 to 11·5)
**Cameroon**						
15–39 years	1670 (872 to 2670)	26·3% (13·7 to 41·9)	0·0192% (−9·87 to 14·6)	3200 (2550 to 3560)	52·4% (41·8 to 58·2)	2·80% (−3·68 to 20·4)
40–64 years	512 (285 to 765)	23·4% (13·1 to 35·0)	4·13% (−4·14 to 12·9)	1060 (758 to 1280)	48·3% (34·7 to 58·8)	6·65% (−1·88 to 22·8)
≥65 years	43·6 (13·3 to 77·5)	9·94% (3·02 to 17·6)	1·98% (−3·63 to 6·77)	119 (73·3 to 156)	29·9% (18·4 to 39·3)	5·90% (−2·12 to 15·4)
**Cape Verde**						
15–39 years	14·7 (6·42 to 25·2)	12·2% (5·34 to 21·0)	−0·969% (−6·88 to 6·49)	85·1 (65·2 to 94·5)	65·6% (50·3 to 72·9)	3·50% (−3·61 to 19·5)
40–64 years	5·66 (2·62 to 9·30)	8·94% (4·13 to 14·7)	0·465% (−3·66 to 4·58)	32·5 (21·3 to 42·4)	50·8% (33·3 to 66·3)	4·13% (−5·29 to 21·0)
≥65 years	0·531 (0·116 to 1·12)	2·68% (0·586 to 5·64)	−0·295% (−2·64 to 1·76)	3·60 (1·72 to 5·21)	29·4% (14·0 to 42·5)	2·01% (−7·74 to 12·6)
**Chad**						
15–39 years	701 (166 to 1260)	22·1% (5·24 to 39·8)	1·82% (−10·6 to 13·9)	1310 (842 to 1520)	45·7% (29·3 to 53·0)	5·37% (−2·82 to 20·4)
40–64 years	139 (30·9 to 264)	14·5% (3·22 to 27·5)	6·55% (0·293 to 13·4)	293 (131 to 427)	29·2% (13·0 to 42·5)	12·1% (3·12 to 21·9)
≥65 years	7·89 (0·378 to 20·6)	4·39% (0·210 to 11·5)	2·54% (0·0582 to 6·53)	30·6 (6·70 to 55·6)	13·4% (2·93 to 24·3)	7·71% (1·87 to 13·8)
**Côte d'Ivoire**						
15–39 years	918 (476 to 1450)	16·9% (8·77 to 26·8)	−3·41% (−12·4 to 9·26)	2720 (2190 to 3040)	48·9% (39·4 to 54·6)	−1·19% (−7·97 to 14·4)
40–64 years	277 (145 to 431)	14·6% (7·62 to 22·7)	−0·178% (−8·00 to 7·24)	977 (719 to 1200)	42·7% (31·4 to 52·5)	4·43% (−4·20 to 19·3)
≥65 years	29·7 (8·59 to 54·8)	8·71% (2·52 to 16·0)	1·03% (−5·01 to 6·34)	115 (69·9 to 151)	31·6% (19·3 to 41·7)	5·31% (−4·44 to 15·5)
**The Gambia**						
15–39 years	53·5 (23·8 to 91·8)	10·6% (4·71 to 18·2)	−0·644% (−6·83 to 6·21)	153 (108 to 179)	32·9% (23·3 to 38·5)	1·15% (−4·97 to 15·4)
40–64 years	14·3 (6·65 to 23·1)	9·08% (4·22 to 14·6)	2·33% (−2·26 to 6·33)	45·8 (28·5 to 63·0)	28·7% (17·9 to 39·4)	7·08% (−1·60 to 17·9)
≥65 years	1·43 (0·315 to 2·88)	3·66% (0·809 to 7·39)	1·32% (−1·19 to 3·87)	5·61 (2·41 to 8·49)	16·7% (7·17 to 25·2)	5·97% (−0·811 to 12·3)
**Ghana**						
15–39 years	1310 (524 to 2210)	18·0% (7·22 to 30·4)	−2·41% (−10·9 to 8·99)	2760 (2050 to 3140)	41·3% (30·7 to 46·9)	0·651% (−5·38 to 15·5)
40–64 years	385 (168 to 635)	12·7% (5·56 to 21·0)	−1·17% (−6·44 to 4·32)	1060 (707 to 1360)	40·2% (26·8 to 51·6)	0·897% (−5·32 to 14·4)
≥65 years	33·6 (6·52 to 69·6)	4·98% (0·968 to 10·3)	−1·40% (−5·33 to 1·46)	122 (61·6 to 174)	24·3% (12·3 to 34·7)	−0·331% (−6·33 to 6·89)
**Guinea**						
15–39 years	47·8 (11·4 to 106)	1·81% (0·430 to 4·01)	−0·399% (−2·08 to 0·990)	621 (364 to 750)	28·2% (16·5 to 34·0)	−0·837% (−7·35 to 11·6)
40–64 years	12·5 (4·06 to 24·1)	1·39% (0·452 to 2·69)	0·0202% (−1·32 to 0·941)	161 (69·2 to 257)	19·5% (8·41 to 31·2)	2·33% (−4·97 to 10·3)
≥65 years	1·09 (0·105 to 2·78)	0·532% (0·0513 to 1·36)	−0·157% (−1·05 to 0·389)	20·2 (5·06 to 38·6)	9·05% (2·27 to 17·3)	1·54% (−3·89 to 6·32)
**Guinea-Bissau**						
15–39 years	55·4 (26·0 to 87·8)	12·9% (6·03 to 20·3)	−0·401% (−5·93 to 6·85)	121 (92·9 to 140)	31·0% (23·7 to 35·8)	0·974% (−4·66 to 11·4)
40–64 years	17·3 (9·49 to 25·7)	12·1% (6·66 to 18·0)	0·389% (−4·48 to 5·69)	36·9 (24·9 to 47·6)	29·3% (19·8 to 37·8)	0·217% (−6·03 to 10·7)
≥65 years	1·10 (0·328 to 2·05)	4·20% (1·25 to 7·79)	−1·00% (−4·88 to 1·80)	3·65 (2·05 to 5·15)	17·1% (9·61 to 24·2)	−1·70% (−8·12 to 5·88)
**Liberia**						
15–39 years	162 (98·0 to 236)	16·3% (9·84 to 23·7)	0·0744% (−5·47 to 7·79)	378 (285 to 429)	39·8% (30·0 to 45·2)	0·832% (−5·12 to 11·7)
40–64 years	47·7 (31·1 to 66·0)	13·9% (9·07 to 19·2)	−0·930% (−5·76 to 4·23)	120 (78·9 to 155)	33·1% (21·9 to 42·9)	−3·12% (−9·55 to 6·12)
≥65 years	3·46 (1·52 to 5·77)	5·11% (2·25 to 8·51)	−1·81% (−5·41 to 1·19)	11·4 (5·52 to 16·3)	16·7% (8·07 to 23·8)	−5·19% (−11·7 to 1·55)
**Mali**						
15–39 years	93·6 (56·2 to 140)	2·08% (1·25 to 3·10)	−0·334% (−1·43 to 1·09)	273 (211 to 330)	6·59% (5·09 to 7·95)	−0·897% (−2·82 to 1·87)
40–64 years	31·7 (19·5 to 45·7)	2·21% (1·36 to 3·19)	−0·342% (−1·65 to 0·861)	99·5 (71·7 to 127)	6·74% (4·86 to 8·57)	−1·05% (−3·78 to 2·60)
≥65 years	5·08 (2·21 to 8·30)	1·68% (0·729 to 2·74)	−0·621% (−2·18 to 0·657)	20·6 (12·0 to 29·0)	6·26% (3·66 to 8·84)	−1·56% (−4·83 to 2·14)
**Mauritania**						
15–39 years	10·9 (0 to 57·2)	1·27% (0 to 6·63)	−0·875% (−5·20 to 1·26)	55·7 (0 to 90·4)	7·02% (0 to 11·4)	−1·49% (−4·88 to 2·63)
40–64 years	0·114 (0 to 0·00615)	0·0337% (0 to 0·00183)	−0·0206% (−0·102 to 0)	0·385 (0 to 7·65)	0·123% (0 to 2·44)	−0·0788% (−1·02 to 0)
≥65 years	0 (0 to 0)	0% (0 to 0)	−0·0000223% (0 to 0)	0 (0 to 0)	0% (0 to 0)	−0·00574% (0 to 0)
**Niger**						
15–39 years	234 (18·4 to 675)	5·38% (0·423 to 15·5)	−1·23% (−7·79 to 2·88)	917 (255 to 1240)	22·0% (6·13 to 29·9)	0·529% (−6·23 to 10·5)
40–64 years	25·3 (1·99 to 89·8)	1·78% (0·140 to 6·33)	0·585% (−1·21 to 2·53)	76·8 (4·32 to 248)	5·98% (0·336 to 19·3)	1·98% (−3·24 to 6·86)
≥65 years	0·599 (0·000975 to 2·99)	0·210% (0·000341 to 1·04)	0·112% (−0·175 to 0·735)	2·79 (0·0430 to 10·6)	1·03% (0·0158 to 3·91)	0·584% (−0·304 to 2·65)
**Nigeria**						
15–39 years	4350 (2990 to 5920)	9·39% (6·47 to 12·8)	−0·224% (−3·76 to 4·76)	11 300 (9530 to 12 700)	27·9% (23·5 to 31·4)	1·22% (−3·95 to 8·81)
40–64 years	2160 (1530 to 2810)	12·4% (8·76 to 16·1)	−0·236% (−4·76 to 4·69)	4870 (3910 to 5660)	32·4% (26·1 to 37·7)	2·77% (−3·46 to 11·5)
≥65 years	307 (177 to 443)	9·71% (5·59 to 14·0)	0·351% (−4·42 to 5·20)	754 (540 to 928)	26·0% (18·6 to 32·0)	1·69% (−4·74 to 8·86)
**São Tomé and Príncipe**						
15–39 years	5·61 (3·14 to 8·62)	12·6% (7·08 to 19·4)	−1·67% (−7·84 to 6·62)	16·7 (13·6 to 19·0)	37·6% (30·5 to 42·7)	0·651% (−5·32 to 11·4)
40–64 years	2·36 (1·41 to 3·46)	12·4% (7·40 to 18·2)	0·447% (−4·97 to 5·99)	7·42 (5·70 to 8·86)	37·9% (29·1 to 45·3)	3·20% (−3·99 to 15·1)
≥65 years	0·272 (0·0920 to 0·460)	6·52% (2·21 to 11·0)	0·291% (−3·71 to 3·82)	0·925 (0·575 to 1·21)	26·7% (16·6 to 34·8)	2·79% (−4·85 to 11·5)
**Senegal**						
15–39 years	191 (22·0 to 501)	6·04% (0·695 to 15·9)	−3·01% (−8·74 to 2·90)	799 (286 to 1050)	25·5% (9·13 to 33·5)	−3·84% (−10·6 to 7·93)
40–64 years	30·5 (3·23 to 91·1)	2·44% (0·259 to 7·30)	−1·80% (−5·71 to 0·853)	99·6 (10·8 to 258)	8·60% (0·933 to 22·2)	−5·39% (−12·6 to 1·88)
≥65 years	1·09 (0·00900 to 4·77)	0·365% (0·00302 to 1·60)	−0·742% (−2·83 to 0·101)	5·37 (0·187 to 16·9)	1·96% (0·0683 to 6·17)	−3·19% (−9·13 to 0·188)
**Sierra Leone**						
15–39 years	94·4 (59·0 to 137)	5·04% (3·15 to 7·34)	−1·87% (−4·69 to 1·82)	353 (289 to 403)	20·0% (16·4 to 22·9)	−3·52% (−7·65 to 1·38)
40–64 years	42·3 (28·3 to 57·6)	7·05% (4·71 to 9·58)	−2·02% (−5·30 to 1·37)	156 (121 to 185)	24·4% (18·9 to 28·9)	−3·69% (−8·88 to 3·13)
≥65 years	4·44 (2·10 to 7·09)	3·24% (1·53 to 5·19)	−1·53% (−3·90 to 0·577)	20·2 (13·3 to 26·4)	15·2% (9·99 to 19·8)	−4·01% (−8·94 to 1·05)
**Togo**						
15–39 years	275 (123 to 454)	16·2% (7·24 to 26·7)	−1·68% (−8·02 to 7·84)	493 (332 to 574)	31·2% (21·0 to 36·3)	−0·635% (−6·23 to 8·69)
40–64 years	82·8 (39·9 to 130)	11·3% (5·46 to 17·9)	−1·09% (−6·63 to 4·41)	156 (91·1 to 218)	23·5% (13·7 to 32·8)	−2·07% (−8·12 to 7·34)
≥65 years	5·47 (1·33 to 11·0)	3·60% (0·877 to 7·23)	−1·29% (−4·53 to 1·39)	11·4 (4·84 to 18·1)	11·7% (4·95 to 18·5)	−2·78% (−8·52 to 3·24)

Data in parentheses are 95% uncertainty intervals. All data are presented to three significant figures.

Harmful consumption was predominantly concentrated among individuals aged 15–39 years, who had the lowest TMRELs and NDEs (figure 4). Of the 1·34 billion (95% UI 1·06–1·62) individuals consuming alcohol in excess of the NDE in 2020, 59·1% (54·3–65·4) were aged 15–39 years. Of these, 75·5% (70·3–80·7) were male (595 million [489–658]). Australasia (83·2% [71·1–86·9]), western Europe (79·3% [67·1–84·5]), and central Europe (78·3% [68·1–83·5]) had the highest percentages of males aged 15–39 years consuming harmful amounts of alcohol (table 1). Among females in the same age group, Australasia (77·7% [65·3–82·0]), western Europe (64·3% [44·3–73·6]), and southern Latin America (59·0% [42·0–67·8]) had the highest rates of harmful alcohol consumption. By contrast, only 6·55% (4·79–8·43) of individuals consuming alcohol in excess of the NDE globally were older than 65 years (appendix 2 pp 54–83). Between 1990 and 2019, 14 countries had significant increases in the prevalence of harmful alcohol consumption, 55 countries had significant decreases, and in 135 countries the prevalence did not change significantly (table 1).

Importantly, these results were not sensitive to our approach to constructing the weighted attributable-cause relative risk curves that are used to calculate the TMREL and NDE. The TMREL and NDE for each of the sensitivity scenarios are shown in appendix 2 (pp 39–41). By including additional risk to health from alcohol use disorders and alcoholic cardiomyopathy, which are 100% attributable to alcohol use, the TMREL decreased by an average of 0·058 (95% UI 0·00–0·30) standard drinks per day in scenario A (for alcohol use disorder and alcoholic cardiomyopathy, relative risks linearly increase with consumption to a maximum relative risk of three at four standard drinks per day, at which point they plateau through ten standard drinks), 0·092 (0·00–0·50) standard drinks per day in scenario B (relative risks linearly increase with consumption to a maximum of five, at four standard drinks per day for alcohol use disorder and alcoholic cardiomyopathy), and 0·146 (0·00–0·60) standard drinks per day in scenario C (relative risks linearly increase with consumption to a maximum of 10, at four standard drinks per day for alcohol use disorder and alcoholic cardiomyopathy), whereas the NDE decreased by an average of 0·188 (0·00–0·90) standard drinks per day in scenario A, 0·310 (0·00–1·30) standard drinks per day in scenario B, and 0·508 (0·00–2·10) standard drinks per day in scenario C. The greatest differences were observed in males in eastern Europe, where alcohol use disorder is especially prevalent, as well as in individuals aged 30–54 years, among whom DALY rates from alcohol use disorder are the greatest (appendix 2 pp 39–41; Bryazka D, unpublished).

Our results were not sensitive to the changes in relative risk estimates for the five updated outcomes, compared to the entire set of relative risk estimates published in GBD 2016 (appendix 2 pp 43–53).^1^ The global, age-standardised, both-sexes TMREL based on the full set of GBD 2016 relative risk estimates and GBD 2020 DALY rates was 0·534 (95% UI 0·00–1·00) standard drinks per day, compared to 0·511 (0·400–0·700) standard drinks per day based on the GBD 2020 relative risk estimates and GBD 2020 DALY rates, whereas the previously published global, age-standardised, both-sexes TMREL was 0·00 (0·00–0·80) standard drinks per day in 2016 based on GBD 2016 relative risk estimates and GBD 2016 DALY rates (table 2).^1^

**Table 2:** Global, age-standardised, both-sexes theoretical minimum risk exposure level and non-drinker equivalence estimates based on various iterations of GBD estimates

	**Previously published (GBD 2016 relative risk and GBD 2016 DALY rates)**	**GBD 2016 relative risk and GBD 2020 DALY rates**	**GBD 2020 relative risk and GBD 2020 DALY rates**
Theoretical minimum risk exposure level	0 (0–0·800)	0·534 (0–1·00)	0·511 (0·400–0·700)
Non-drinker equivalence	NA	0·669 (0–1·40)	1·72 (0·80–3·30)

Data in parentheses are 95% uncertainty intervals. Data are reported to three significant figures. GBD=Global Burden of Diseases, Injuries, and Risk Factors Study. DALY=disability-adjusted life-year. NA=not applicable.

## Discussion

We show that the estimation of the health effects associated with alcohol use requires consideration of both the relationship between alcohol consumption and disease outcomes, and the observed disease rates in each population. We found that the population-level health risks associated with low levels of alcohol consumption varied across regions and were greater for younger populations than for older populations. Although we did not find significant differences in the risks of ill health by sex or by year, we did find that males made up 76·9% (95% UI 73·0–81·3) of the population consuming harmful amounts of alcohol in 2020. Notably, 1·03 billion (0·851–1·19) males and 312 million (199–432) females drank harmful amounts of alcohol in excess of the NDE in 2020. Harmful use of alcohol was particularly concentrated in males aged 15–39 years, primarily in Australasia, western Europe, and central Europe. These findings highlight the need for tailored guidelines that discourage alcohol consumption among young people, as well as alcohol control policies and interventions that are targeted especially towards young males.

Understanding the variation in the level of alcohol consumption that minimises the risk of ill health for populations can aid in setting effective consumption guidelines, supporting alcohol control policies, monitoring progress in reducing harmful alcohol use, and designing public health risk messaging.^3^, ^16^, ^34^, ^35^, ^36^, ^37^, ^38^, ^39^, ^40^ Most alcohol consumption guidelines for the general population combine recommendations to avoid alcohol use with the definition of lower-risk alcohol consumption thresholds, which tend to vary between 8 g and 42 g of alcohol per day for females, and between 10 g and 52 g of alcohol per day for males.^41^ Generally, thresholds are one standard drink greater for men than for women, and some lower-risk thresholds are framed in units of weekly consumption that come with a recommendation to avoid alcohol entirely for several days of the week.

In our analysis, the population-specific TMRELs ranged between 0 (95% UI 0–0) and 0·603 (0·400–1·00) standard drinks per day among individuals aged 15–39 years across world regions, and the NDEs ranged between 0 (0–0) and 1·75 (0·698–4·30) standard drinks per day among individuals aged 15–39 years across world regions in 2020. Even if a conservative approach is taken and the lower bound of the uncertainty interval is used to set policy recommendations rather than the mean, this implies that the recommended level of alcohol consumption in existing low consumption recommendations is too high for younger populations. Our estimates, based on currently available evidence, also do not support low consumption guidelines that differ by sex. Given the known difficulties associated with translating evidence into changes in consumer behaviour, clear messaging around updates to drinking guidelines will be crucial to ensure the full improvements are realised.

One key distinction between this study and existing recommendations on alcohol consumption is that our estimates focus on minimising health loss across all alcohol-attributable outcomes in a population. Thresholds exist for different purposes; in terms of injury prevention, several countries have moved to a zero-tolerance threshold that is consistent with evidence of the entirely harmful effect of alcohol consumption on injuries. Furthermore, individual-level as opposed to population-level risk minimisation will depend on individual-level factors, including comorbid conditions and the use of pharmaceuticals, which are more prevalent among older populations. Our results for older adults should be interpreted in the context of their additional uncertainty.^42^, ^43^ Approaches to minimising individual-level risk are beyond the scope of this study and need to take into consideration not only alcohol use and specific health outcomes, but also interactions between environmental, genetic, and behavioural factors, as well as the societal and health system context of individuals.

Broadly, this analysis highlights the need to consider the existing prevalence of diseases and injuries for specific populations when determining the total harms posed by a risk factor. Although the biological effects of alcohol are unlikely to change across populations, except in the case of specific genetic interactions such as variants in alcohol dehydrogenase, disease rates vary substantially across regions, age, sex, and time.^44^ For example, alcohol use poses a greater risk to population health in areas with a high prevalence of tuberculosis than in areas with low prevalence. Although this consideration is perhaps most important for setting effective policy recommendations for risks with both harmful and protective relationships with disease, such as alcohol use and red meat consumption, it has implications for all risk factors. As countries navigate the epidemiological transition and their background rates of disease evolve from infectious diseases and injuries to non-communicable diseases, policy recommendations will need to evolve as well.

It is important to consider our findings in the context of those published by the GBD 2016 Alcohol Collaborators in 2018.^1^ Compared to that report, the analysis presented here includes three major changes: we updated five of the relative risk curves; we weighted the relative risk curves using DALY rates estimated as part of GBD 2020 rather than GBD 2016; and we estimated the TMREL separately for each region, age, sex, and year. Although the GBD 2016 Alcohol Collaborators found that the global, age-standardised, both-sexes TMREL was zero standard drinks per day, computing the global TMREL with the first two of these updates, we found that the global TMREL was still quite low, at 0·511 (95% UI 0·400–0·700) standard drinks per day. Re-estimating the TMREL with updated 2020 DALY weights but the former relative risk curves suggests a global TMREL of 0·534 (0–1·00) standard drinks per day. Region-specific, age-specific, and sex-specific differences between these approaches are summarised in appendix 2 (pp 45–55). Importantly, the differences across TMREL by region and age hold even with the relative risk curves estimated in 2016. The more nuanced analysis in the present study, where we explored the risks to ill health by age and region, represents a major step forward in our understanding of how to minimise health loss due to alcohol consumption across the world.

One challenge associated with using observational studies to measure the causal effect of alcohol consumption on health is the potential for the introduction of various forms of bias, including reverse causation, selection bias, and residual confounding. Mendelian randomisation is a method that attempts to mitigate bias by using genetic variation as a proxy for risk exposure.^5^, ^6^ Although a small number of Mendelian randomisation studies have been done on alcohol use to date, a recent meta-analysis reported that those done on cardiovascular disease and diabetes had varied in their findings, with 67% of studies on cardiovascular disease and 75% of studies on diabetes reporting a null association with alcohol.^6^ However, only five of 24 studies examined whether alcohol had a non-linear relationship with these health outcomes. As additional Mendelian randomisation studies from diverse populations are increasingly published, they have the potential to improve the evidence base, and estimates should be regularly revised to reflect new evidence.

This study had various limitations that should be taken into account when interpreting the findings. First, we did not incorporate patterns of drinking, and therefore did not distinguish between individuals who infrequently engage in heavy episodic drinking and those who consume the same amount of alcohol over several days.^45^ Manthey and colleagues^46^ estimated that in 2018, 20% of adults engaged in heavy episodic drinking—the consumption of 60 g or more of alcohol on a single occasion—over the past month. Second, due to a paucity of studies reporting a dose–response relationship between the risk of alcohol use and incidence of and mortality from alcohol use disorders, the burden of alcohol use disorders was not included in the TMREL calculation. As shown by the sensitivity analyses, which used conservative hypothetical relative risk curves for alcohol use disorder and alcoholic cardiomyopathy, inclusion of these diseases results in slightly lower estimates of TMREL and NDE, particularly among males in eastern Europe and in individuals aged 30–54 years globally. The decreases in the TMREL and NDE in the sensitivity scenarios were found to be quite small, since the risk of these two conditions is likely to be concentrated at higher levels of consumption and in younger adults, resulting in minimal impact on estimates of the TMREL and NDE. Third, although we attempted to adjust for the impacts of confounding and bias in our meta-regressions, it is possible that relative risk estimates did not account and adjust for all sources of bias, including measurement bias and selection bias, as well as the potential impacts of reverse causality. Fourth, studies reporting the relative risks of alcohol use were based on self-reported alcohol consumption, which is subject to social desirability and recall biases. Fifth, we did not consider differences in risk by type or quality of alcohol. Sixth, the weights used within the weighted alcohol-attributable relative risk curve used DALY estimates that could be due to alcohol use. However, this limitation would only have had a marginal effect on estimates of the TMREL and NDE. Seventh, our estimates of the proportion of the population consuming alcohol in excess of the NDE were derived from alcohol consumption data collected through 2019. Because of delays in routine data collection on risk factors caused by the COVID-19 pandemic, we forecasted our estimates to obtain a time series through 2020. As a result, the estimates do not reflect changes in consumption patterns associated with the pandemic.^47^ Last, our results did not include health conditions with burgeoning evidence indicating a relationship with alcohol use, such as major depressive disorder, generalised anxiety disorder, or dementia, given the current scarcity of sufficient evidence to support a meta-analysis and the potential for reverse causality. Inclusions of these outcomes would possibly reduce estimates of the TMREL and NDE.

In conclusion, the relationship between moderate alcohol use and health is complex and has raised a great deal of controversy in the scientific literature. Given that the available evidence suggests that low levels of alcohol consumption are associated with a lower risk of some disease outcomes and an increased risk of others, alcohol consumption recommendations should take into account the full epidemiological profile that includes the background rates of disease within populations. The findings of this study support the development of tailored guidelines and recommendations on alcohol consumption by age and across regions and highlight that existing low consumption thresholds are too high for younger populations in all regions. Additionally, our results suggest that guidelines should not incorporate sex-specific recommendations, given the absence of variation in TMREL and NDE by sex across geographies and locations. Finally, recognising that the majority of the world's population consuming harmful amounts of alcohol are young adults and predominantly young males, in order to minimise health loss due to alcohol consumption it is important to prioritise interventions targeted at these demographic groups.

## Data sharing

For access to the data used for this specific analysis before the full publication of GBD 2020, please contact Emmanuela Gakidou at gakidou@uw.edu.

## Declaration of interests

O M Adebayo reports grants or contracts from Merck Foundation and Servier Nigeria; payment or honoraria for lectures, presentations, speakers bureaus, manuscript writing, or educational events from Merck Foundation; support for attending meetings or travel from Servier Nigeria; and a leadership or fiduciary role in a board, society, committee or advocacy group, paid or unpaid, with the Nigerian Association of Resident Doctors; all outside the submitted work. S Afzal reports honorary participation on the Institutional Review Board of King Edward Medical University (Lahore, Pakistan), the Quality Enhancement Cell at Fatima Jinnah Medical University (Lahore, Pakistan), and the Corona Expert Advisory Group (Pakistan); an unpaid leadership or fiduciary role in board, society, committee or advocacy group, with the Pakistan Society of Community Medicine & Public Health, Pakistan Association of Medical Editors, and Pakistan Society of Medical Infectious Diseases; all outside the submitted work. R Ancuceanu reports consulting fees from AbbVie; payment or honoraria for lectures, presentations, speakers bureaus, manuscript writing or educational events from AbbVie, B. Braun Medical, Sandoz, and Laropharm; all outside the submitted work. P Atorkey reports support for the present manuscript via funding from the School of Medicine and Public Health, The University of Newcastle (Callaghan, NSW, Australia), and infrastructure support from Hunter New England-Population Health and Hunger Medical Research Institute, Australia. M Ausloos reports a research grant from the Romanian National Authority for Scientific Research and Innovation, CNDS-UEFISCDI (project number: PN-III-P4-ID-PCCF-2016-0084, title: “Understanding and modelling time-space patterns of psychology-related inequalities and polarization”) outside the submitted work. T Bärnighausen reports grants from the European Union (Horizon 2020 and EIT Health), German Research Foundation (DFG), US National Institutes of Health, German Ministry of Education and Research, Alexander von Humboldt Foundation, Else-Kröner-Fresenius-Foundation, Wellcome Trust, Bill & Melinda Gates Foundation, KfW, Joint United Nations Programme on HIV/AIDS (UNAIDS), World Health Organization; consulting fees from KfW for the OSCAR initiative in Vietnam; participation on a Data Safety Monitoring Board or Advisory Board with National Institutes of Health (US)-funded study “Healthy Options” (PIs: Smith Fawzi, Kaaya) as Chair of the Data Safety and Monitoring Board, with the German National Committee on the “Future of Public Health Research and Education”, as chair of the scientific advisory board to the EDCTP Evaluation, as a member of the UNAIDS Evaluation Expert Advisory Committee, as a National Institutes of Health (US) Study Section Member on Population and Public Health Approaches to HIV/AIDS (PPAH), with the US National Academies of Sciences, Engineering, and Medicine's Committee for the “Evaluation of Human Resources for Health in the Republic of Rwanda under the President's Emergency Plan for AIDS Relief (PEPFAR)”, with the University of Pennsylvania Population Aging Research Center (PARC) as an External Advisory Board Member; leadership or fiduciary role in a board, society, committee or advocacy group, paid or unpaid, with the Global Health Hub Germany (which was initiated by the German Ministry of Health) as a co-chair; all outside the submitted work. S M M Bhaskar reports grants or contracts from the NSW Ministry of Health, Australia; a leadership or fiduciary role in a board, society, committee or advocacy group, paid or unpaid, with the Rotary Club of Sydney as board director, with the International Rotary Fellowship of Healthcare Professionals (UK) as board director, with Global Health & Migration Hub Community, Global Health Hub Germany, Berlin as a chair or manager; all outside the submitted work. J M Castaldelli-Maia reports grants or contracts from Pfizer (Independent Grants for Learning and Change) and the French National Institute for Cancer (INCa); consulting fees from L'Oreal Mental Health Wellness International Board; all outside the submitted work. S Costanzo reports a research grant from the European Foundation for Alcohol Research (ERAB) (ID EA1767; 2018-2020); payment or honoraria for lectures, presentations, speakers bureaus, manuscript writing or educational events from The Dutch Beer Institute Foundation - The Brewers of Europe as a member of the Organizing Committee and speaker for the 9th European Beer and Health Symposium (Bruxelles 2019), and for giving a lecture at the 13th European Nutrition Conference FENS 2019 (Dublin), sponsored by the Beer and Health Initiative (The Dutch Beer Institute foundation - The Brewers of Europe); all outside the submitted work. I Filip reports financial or non-financial support from the Avicenna Medical and Clinical Research Institute (California, USA). R C Franklin reports leadership or fiduciary role in board, society, committee or advocacy group, paid or unpaid with Kidsafe, Farmsafe, Royal Life Saving Society – Australia, and PHAA – Injury Prevention SIG outside the submitted work. C Herteliu reports research grants from the Romanian Ministry of Research Innovation and Digitalization, MCID (ID-585-CTR-42-PFE-2021, Jan 2022-Jun 2023, “Enhancing institutional performance through development of infrastructure and transdisciplinary research ecosystem within socio-economic domain – PERFECTIS”), the Romanian National Authority for Scientific Research and Innovation, CNDS-UEFISCDI (PN-III-P4-ID-PCCF-2016-0084, October, 2018, to September, 2022, “Understanding and modelling time-space patterns of psychology-related inequalities and polarization”; PN-III-P2-2.1-SOL-2020-2-0351, June, 2020, to October, 2020, “Approaches within public health management in the context of COVID-19 pandemic”), and the Ministry of Labour and Social Justice, Romania (30/PSCD/2018, September, 2018, to June, 2019, “Agenda for skills Romania 2020-2025”), all outside the submitted work. J J Jozwiak reports payment or honoraria for lectures, presentations, speakers bureaus, manuscript writing or educational events from Teva Pharmaceuticals, Amgen, Synexus, Boehringer Ingelheim, ALAB Laboratories, and Zentiva, outside the submitted work. M Kivimäki reports support for the present manuscript from the Wellcome Trust (221854/Z/20/Z), the UK Medical Research Council (MR/S011676/1), the US National Institute on Aging (R01AG056477), and the Academy of Finland (350426) in the form of research grants to their institution. K Krishan reports non-financial support from the UGC Centre of Advanced Study (Phase II), Department of Anthropology, Panjab University, Chandigarh, India, outside the submitted work. S Lorkowski reports grants or contracts paid to his institution from Akcea Therapeutics Germany; consulting fees from Danone, Novartis Pharma, Swedish Orphan Biovitrum (SOBI), and Upfield; payment or honoraria for lectures, presentations, speaker's bureaus, manuscript writing, or educational events from Akcea Therapeutics Germany, Amarin Germany, Amedes Holding, Amgen, Berlin-Chemie, Boehringer Ingelheim, Daiichi Sankyo Deutschland, Danone, Hubert Burda Media Holding, Janssen-Cilag, Lilly Deutschland, Novartis, Novo Nordisk, F Hoffmann-La Roche (Roche), Sanofi-Aventis, SYNLAB Holding Deutschland, and SYNLAB Akademie; support for attending meetings or travel from Amgen; and participation on a data safety monitoring board or advisory board for Akcea Therapeutics Germany, Amgen, Daiichi Sankyo Deutschland, Novartis, and Sanofi-Aventis; all outside the submitted work. A M Madureira-Carvalho reports grants or contracts from Instituto Universitário de Ciências da Saúde (Gandra, Portugal); consulting fees from Albert Labs and Eurox Pharma paid to her and her institution; a leadership or fiduciary role in a board, society, committee or advocacy group, paid or unpaid, with the Portuguese Association of Forensic Sciences (APCF); all outside the submitted work. A-F A Mentis reports grants or contracts from “MilkSafe: A novel pipeline to enrich formula milk using omics technologies”, a research co-financed by the European Regional Development Fund of the European Union and Greek national funds through the Operational Program Competitiveness, Entrepreneurship and Innovation, under the call RESEARCH - CREATE - INNOVATE (project code: T2EDK-02222), as well as from ELIDEK (Hellenic Foundation for Research and Innovation, MIMS-860); stock or stock options in a family winery; and support from BGI Group as a scientific officer; all outside the submitted work. C D H Parry reports grants or contracts from the South African Medical Research Council paid to their institution; consulting fees paid to them from the World Health Organization (WHO) and the University of Cape Town (Cape Town, South Africa); payment or honoraria for a lecture on alcohol & NCDs in 2020 from the University of Cape Town; support for attending meetings or travel from UCT African Union 2022, UN Office on Drugs & Crime 2019, and WHO meeting on alcohol in Uganda 2021; participation on a Data Safety Monitoring Board or Advisory Board with the UK SPECTRUM Project (multi university) Global Advisory Board, and the UN Office on Drugs & Crime WDR Scientific Advisory Board, both unpaid; all outside the submitted work. G A Roth reports support for the present manuscript from the Bill & Melinda Gates Foundation via a research grant to their institution. G A Roth reports grants or contracts from the American Heart Association, the American College of Cardiology, and the National Heart, Lung, and Blood Institute paid to their institution, outside the submitted work V Shivarov reports financial support from ICON plc. J A Singh reports consulting fees from Crealta/Horizon, Medisys, Fidia, PK Med, Two Labs, Adept Field Solutions, Clinical Care Options, Clearview Healthcare Partners, Putnam Associates, Focus Forward, Navigant Consulting, Spherix, MedIQ, Jupiter Life Science, UBM, Trio Health, Medscape, WebMD, and Practice Point Communications, the National Institutes of Health, and the American College of Rheumatology; payment or honoraria for lectures, presentations, speakers' bureaus, manuscript writing, or educational events from Simply Speaking; support for attending meetings or travel from the steering committee of OMERACT; participation on a Data Safety Monitoring Board or Advisory Board with the US Food and Drug Administration Arthritis Advisory Committee; leadership or fiduciary role in board, society, committee or advocacy group, paid or unpaid, with OMERACT as a steering committee member, with the Veterans Affairs Rheumatology Field Advisory Committee as Chair (unpaid), and with the UAB Cochrane Musculoskeletal Group Satellite Center on Network Meta-analysis and editor and director (unpaid); stock or stock options in TPT Global Tech, Vaxart Pharmaceuticals, Aytu BioPharma, Adaptimmune Therapeutics, GeoVax Labs, Pieris Pharmaceuticals, Enzolytics, Seres Therapeutics, Tonix Pharmaceuticals and Charlotte's Web Holdings, and previously owned stock options in Amarin, Viking, and Moderna Pharmaceuticals; all outside the submitted work.
