# Department of Error

**DOI:** 10.1016/S0140-6736(22)01389-7

**Published:** 2022-07-30

*GBD 2020 Alcohol Collaborators. Population-level risks of alcohol consumption by amount, geography, age, sex, and year: a systematic analysis for the Global Burden of Disease Study 2020.* Lancet *2022; **400:** 185–235*—Appendices 1 and 2 of this Article have been corrected as of July 19, 2022.

