# Supplementary material for: Population-level risks of alcohol consumption by amount, geography, age, sex, and year: a systematic analysis for the Global Burden of Disease Study 2020
Source: Lancet. 2022 Jul 16;400(10347):185–235. doi: 10.1016/S0140-6736(22)00847-9 (PMC9289789; doi:10.1016/S0140-6736(22)00847-9)
Supplement: Supplementary appendix 1 [file mmc1.pdf]

THE LANCET

Supplementary appendix 1

This appendix formed part of the original submission and has been peer reviewed. We post it as supplied by the authors.

This online publication has been corrected. The corrected version first appeared at thelancet.com on July 19, 2022

Supplement to: GBD 2020 Alcohol Collaborators. Population-level risks of alcohol consumption by amount, geography, age, sex, and year: a systematic analysis for the Global Burden of Disease Study 2020. *Lancet* 2022; **400:** 185–235.

Supplementary Methods

Risks of alcohol consumption by geography, age, sex, and year: a systematic analysis from the Global Burden of Disease Study 2020

GBD 2020 Alcohol Use Collaborators

Table of Contents

Geographies Estimated ***3***
Geographic Hierarchy 3
Demographics ***13***
Relative Risks ***14***
Systematic Review **14**
Estimating Dose-Response Relative Risk Curves **14**
Theoretical Minimum Risk Exposure and Non-Drinker Equivalence Levels ***16***
Computing aggregate relative risks **16**
Theoretical Minimum Risk Exposure Level **16**
Non-drinker Equivalence Level **17**
Population Attributable Fractions ***17***
Sensitivity Analyses ***17***
Search Strings and PRISMA Diagrams ***18***
Diabetes Mellitus ***18***
Intracerebral Hemorrhage ***23***
Ischemic Stroke ***27***
Ischemic heart disease ***32***
Tuberculosis ***40***
Modeled Relative Risk Curves ***42***
GATHER Checklist ***47***
Contributions ***49***
Managing the overall research enterprise 49
Writing the first draft of the manuscript 49
Primary responsibility for applying analytical methods to produce estimates 49
Primary responsibility for seeking, cataloguing, extracting, or cleaning data; designing or coding figures and tables 49
Providing data or critical feedback on data sources 49
Developing methods or computational machinery 50
Providing critical feedback on methods or results 50
Drafting the work or revising it critically for important intellectual content 53
Managing the estimation or publications process 54

Geographies Estimated

This study utilized the standard geographical hierarchy for the Global Burden of Diseases, Injuries, and Risk Factors Study 2020 (GBD 2020). The 204 countries and territories included in this analysis are nested in 21 aggregate regions and seven aggregate super-regions (Table SM1). The regions and super-regions in the GBD are defined based on a combination of epidemiologic patterns and spatial distance.

Geographic Hierarchy

Location	Super-Region	Region
**Armenia**	Central Europe, Eastern Europe, and Central Asia	Central Asia
**Azerbaijan**	Central Europe, Eastern Europe, and Central Asia	Central Asia
**Georgia**	Central Europe, Eastern Europe, and Central Asia	Central Asia
**Kazakhstan**	Central Europe, Eastern Europe, and Central Asia	Central Asia
**Kyrgyzstan**	Central Europe, Eastern Europe, and Central Asia	Central Asia
**Mongolia**	Central Europe, Eastern Europe, and Central Asia	Central Asia
**Tajikistan**	Central Europe, Eastern Europe, and Central Asia	Central Asia
**Turkmenistan**	Central Europe, Eastern Europe, and Central Asia	Central Asia
**Uzbekistan**	Central Europe, Eastern Europe, and Central Asia	Central Asia
**Albania**	Central Europe, Eastern Europe, and Central Asia	Central Europe
**Bosnia and Herzegovina**	Central Europe, Eastern Europe, and Central Asia	Central Europe
**Bulgaria**	Central Europe, Eastern Europe, and Central Asia	Central Europe
**Croatia**	Central Europe, Eastern Europe, and Central Asia	Central Europe
**Czechia**	Central Europe, Eastern Europe, and Central Asia	Central Europe
**Hungary**	Central Europe, Eastern Europe, and Central Asia	Central Europe
**Montenegro**	Central Europe, Eastern Europe, and Central Asia	Central Europe
**North Macedonia**	Central Europe, Eastern Europe, and Central Asia	Central Europe
**Poland**	Central Europe, Eastern Europe, and Central Asia	Central Europe

**Romania**	Central Europe, Eastern Europe, and Central Asia	Central Europe
**Serbia**	Central Europe, Eastern Europe, and Central Asia	Central Europe
**Slovakia**	Central Europe, Eastern Europe, and Central Asia	Central Europe
**Slovenia**	Central Europe, Eastern Europe, and Central Asia	Central Europe
**Belarus**	Central Europe, Eastern Europe, and Central Asia	Eastern Europe
**Estonia**	Central Europe, Eastern Europe, and Central Asia	Eastern Europe
**Latvia**	Central Europe, Eastern Europe, and Central Asia	Eastern Europe
**Lithuania**	Central Europe, Eastern Europe, and Central Asia	Eastern Europe
**Republic of Moldova**	Central Europe, Eastern Europe, and Central Asia	Eastern Europe
**Russia**	Central Europe, Eastern Europe, and Central Asia	Eastern Europe
**Ukraine**	Central Europe, Eastern Europe, and Central Asia	Eastern Europe
**Australia**	High-income	Australasia
**New Zealand**	High-income	Australasia
**Brunei Darussalam**	High-income	High-income Asia Pacific
**Japan**	High-income	High-income Asia Pacific
**Republic of Korea**	High-income	High-income Asia Pacific
**Singapore**	High-income	High-income Asia Pacific
**Canada**	High-income	High-income North America
**Greenland**	High-income	High-income North America
**United States of America**	High-income	High-income North America
**Argentina**	High-income	Southern Latin America
**Chile**	High-income	Southern Latin America
**Uruguay**	High-income	Southern Latin America

**Andorra**	High-income	Western Europe
**Austria**	High-income	Western Europe
**Belgium**	High-income	Western Europe
**Cyprus**	High-income	Western Europe
**Denmark**	High-income	Western Europe
**Finland**	High-income	Western Europe
**France**	High-income	Western Europe
**Germany**	High-income	Western Europe
**Greece**	High-income	Western Europe
**Iceland**	High-income	Western Europe
**Ireland**	High-income	Western Europe
**Israel**	High-income	Western Europe
**Italy**	High-income	Western Europe
**Luxembourg**	High-income	Western Europe
**Malta**	High-income	Western Europe
**Monaco**	High-income	Western Europe
**Netherlands**	High-income	Western Europe
**Norway**	High-income	Western Europe
**Portugal**	High-income	Western Europe
**San Marino**	High-income	Western Europe
**Spain**	High-income	Western Europe
**Sweden**	High-income	Western Europe
**Switzerland**	High-income	Western Europe

**United Kingdom**	High-income	Western Europe
**Bolivia (Plurinational State of)**	Latin America and Caribbean	Andean Latin America
**Ecuador**	Latin America and Caribbean	Andean Latin America
**Peru**	Latin America and Caribbean	Andean Latin America
**Antigua and Barbuda**	Latin America and Caribbean	Caribbean
**Bahamas**	Latin America and Caribbean	Caribbean
**Barbados**	Latin America and Caribbean	Caribbean
**Belize**	Latin America and Caribbean	Caribbean
**Bermuda**	Latin America and Caribbean	Caribbean
**Cuba**	Latin America and Caribbean	Caribbean
**Dominica**	Latin America and Caribbean	Caribbean
**Dominican Republic**	Latin America and Caribbean	Caribbean
**Grenada**	Latin America and Caribbean	Caribbean
**Guyana**	Latin America and Caribbean	Caribbean
**Haiti**	Latin America and Caribbean	Caribbean
**Jamaica**	Latin America and Caribbean	Caribbean
**Puerto Rico**	Latin America and Caribbean	Caribbean
**Saint Kitts and Nevis**	Latin America and Caribbean	Caribbean
**Saint Lucia**	Latin America and Caribbean	Caribbean
**Saint Vincent and the Grenadines**	Latin America and Caribbean	Caribbean
**Suriname**	Latin America and Caribbean	Caribbean
**Trinidad and Tobago**	Latin America and Caribbean	Caribbean
**United States Virgin Islands**	Latin America and Caribbean	Caribbean

**Colombia**	Latin America and Caribbean	Central Latin America
**Costa Rica**	Latin America and Caribbean	Central Latin America
**El Salvador**	Latin America and Caribbean	Central Latin America
**Guatemala**	Latin America and Caribbean	Central Latin America
**Honduras**	Latin America and Caribbean	Central Latin America
**Mexico**	Latin America and Caribbean	Central Latin America
**Nicaragua**	Latin America and Caribbean	Central Latin America
**Panama**	Latin America and Caribbean	Central Latin America
**Venezuela (Bolivarian Republic of)**	Latin America and Caribbean	Central Latin America
**Brazil**	Latin America and Caribbean	Tropical Latin America
**Paraguay**	Latin America and Caribbean	Tropical Latin America
**Afghanistan**	North Africa and Middle East	North Africa and Middle East
**Algeria**	North Africa and Middle East	North Africa and Middle East
**Bahrain**	North Africa and Middle East	North Africa and Middle East
**Egypt**	North Africa and Middle East	North Africa and Middle East
**Iran (Islamic Republic of)**	North Africa and Middle East	North Africa and Middle East
**Iraq**	North Africa and Middle East	North Africa and Middle East
**Jordan**	North Africa and Middle East	North Africa and Middle East
**Kuwait**	North Africa and Middle East	North Africa and Middle East
**Lebanon**	North Africa and Middle East	North Africa and Middle East
**Libya**	North Africa and Middle East	North Africa and Middle East
**Morocco**	North Africa and Middle East	North Africa and Middle East
**Oman**	North Africa and Middle East	North Africa and Middle East

**Palestine**	North Africa and Middle East	North Africa and Middle East
**Qatar**	North Africa and Middle East	North Africa and Middle East
**Saudi Arabia**	North Africa and Middle East	North Africa and Middle East
**Sudan**	North Africa and Middle East	North Africa and Middle East
**Syrian Arab Republic**	North Africa and Middle East	North Africa and Middle East
**Tunisia**	North Africa and Middle East	North Africa and Middle East
**Turkey**	North Africa and Middle East	North Africa and Middle East
**United Arab Emirates**	North Africa and Middle East	North Africa and Middle East
**Yemen**	North Africa and Middle East	North Africa and Middle East
**Bangladesh**	South Asia	South Asia
**Bhutan**	South Asia	South Asia
**India**	South Asia	South Asia
**Nepal**	South Asia	South Asia
**Pakistan**	South Asia	South Asia
**China**	Southeast Asia, East Asia, and Oceania	East Asia
**Democratic People's Republic of Korea**	Southeast Asia, East Asia, and Oceania	East Asia
**Taiwan (Province of China)**	Southeast Asia, East Asia, and Oceania	East Asia
**American Samoa**	Southeast Asia, East Asia, and Oceania	Oceania
**Cook Islands**	Southeast Asia, East Asia, and Oceania	Oceania
**Fiji**	Southeast Asia, East Asia, and Oceania	Oceania
**Guam**	Southeast Asia, East Asia, and Oceania	Oceania
**Kiribati**	Southeast Asia, East Asia, and Oceania	Oceania
**Marshall Islands**	Southeast Asia, East Asia, and Oceania	Oceania

**Micronesia (Federated States of)**	Southeast Asia, East Asia, and Oceania	Oceania
**Nauru**	Southeast Asia, East Asia, and Oceania	Oceania
**Niue**	Southeast Asia, East Asia, and Oceania	Oceania
**Northern Mariana Islands**	Southeast Asia, East Asia, and Oceania	Oceania
**Palau**	Southeast Asia, East Asia, and Oceania	Oceania
**Papua New Guinea**	Southeast Asia, East Asia, and Oceania	Oceania
**Samoa**	Southeast Asia, East Asia, and Oceania	Oceania
**Solomon Islands**	Southeast Asia, East Asia, and Oceania	Oceania
**Tokelau**	Southeast Asia, East Asia, and Oceania	Oceania
**Tonga**	Southeast Asia, East Asia, and Oceania	Oceania
**Tuvalu**	Southeast Asia, East Asia, and Oceania	Oceania
**Vanuatu**	Southeast Asia, East Asia, and Oceania	Oceania
**Cambodia**	Southeast Asia, East Asia, and Oceania	Southeast Asia
**Indonesia**	Southeast Asia, East Asia, and Oceania	Southeast Asia
**Lao People's Democratic Republic**	Southeast Asia, East Asia, and Oceania	Southeast Asia
**Malaysia**	Southeast Asia, East Asia, and Oceania	Southeast Asia
**Maldives**	Southeast Asia, East Asia, and Oceania	Southeast Asia
**Mauritius**	Southeast Asia, East Asia, and Oceania	Southeast Asia
**Myanmar**	Southeast Asia, East Asia, and Oceania	Southeast Asia
**Philippines**	Southeast Asia, East Asia, and Oceania	Southeast Asia
**Seychelles**	Southeast Asia, East Asia, and Oceania	Southeast Asia
**Sri Lanka**	Southeast Asia, East Asia, and Oceania	Southeast Asia
**Thailand**	Southeast Asia, East Asia, and Oceania	Southeast Asia

**Timor-Leste**	Southeast Asia, East Asia, and Oceania	Southeast Asia
**Viet Nam**	Southeast Asia, East Asia, and Oceania	Southeast Asia
**Angola**	Sub-Saharan Africa	Central Sub-Saharan Africa
**Central African Republic**	Sub-Saharan Africa	Central Sub-Saharan Africa
**Congo**	Sub-Saharan Africa	Central Sub-Saharan Africa
**Democratic Republic of the Congo**	Sub-Saharan Africa	Central Sub-Saharan Africa
**Equatorial Guinea**	Sub-Saharan Africa	Central Sub-Saharan Africa
**Gabon**	Sub-Saharan Africa	Central Sub-Saharan Africa
**Burundi**	Sub-Saharan Africa	Eastern Sub-Saharan Africa
**Comoros**	Sub-Saharan Africa	Eastern Sub-Saharan Africa
**Djibouti**	Sub-Saharan Africa	Eastern Sub-Saharan Africa
**Eritrea**	Sub-Saharan Africa	Eastern Sub-Saharan Africa
**Ethiopia**	Sub-Saharan Africa	Eastern Sub-Saharan Africa
**Kenya**	Sub-Saharan Africa	Eastern Sub-Saharan Africa
**Madagascar**	Sub-Saharan Africa	Eastern Sub-Saharan Africa
**Malawi**	Sub-Saharan Africa	Eastern Sub-Saharan Africa
**Mozambique**	Sub-Saharan Africa	Eastern Sub-Saharan Africa
**Rwanda**	Sub-Saharan Africa	Eastern Sub-Saharan Africa
**Somalia**	Sub-Saharan Africa	Eastern Sub-Saharan Africa
**South Sudan**	Sub-Saharan Africa	Eastern Sub-Saharan Africa
**Uganda**	Sub-Saharan Africa	Eastern Sub-Saharan Africa
**United Republic of Tanzania**	Sub-Saharan Africa	Eastern Sub-Saharan Africa
**Zambia**	Sub-Saharan Africa	Eastern Sub-Saharan Africa

**Botswana**	Sub-Saharan Africa	Southern Sub-Saharan Africa
**Eswatini**	Sub-Saharan Africa	Southern Sub-Saharan Africa
**Lesotho**	Sub-Saharan Africa	Southern Sub-Saharan Africa
**Namibia**	Sub-Saharan Africa	Southern Sub-Saharan Africa
**South Africa**	Sub-Saharan Africa	Southern Sub-Saharan Africa
**Zimbabwe**	Sub-Saharan Africa	Southern Sub-Saharan Africa
**Benin**	Sub-Saharan Africa	Western Sub-Saharan Africa
**Burkina Faso**	Sub-Saharan Africa	Western Sub-Saharan Africa
**Cabo Verde**	Sub-Saharan Africa	Western Sub-Saharan Africa
**Cameroon**	Sub-Saharan Africa	Western Sub-Saharan Africa
**Chad**	Sub-Saharan Africa	Western Sub-Saharan Africa
**Côte d'Ivoire**	Sub-Saharan Africa	Western Sub-Saharan Africa
**Gambia**	Sub-Saharan Africa	Western Sub-Saharan Africa
**Ghana**	Sub-Saharan Africa	Western Sub-Saharan Africa
**Guinea**	Sub-Saharan Africa	Western Sub-Saharan Africa
**Guinea-Bissau**	Sub-Saharan Africa	Western Sub-Saharan Africa
**Liberia**	Sub-Saharan Africa	Western Sub-Saharan Africa
**Mali**	Sub-Saharan Africa	Western Sub-Saharan Africa
**Mauritania**	Sub-Saharan Africa	Western Sub-Saharan Africa
**Niger**	Sub-Saharan Africa	Western Sub-Saharan Africa
**Nigeria**	Sub-Saharan Africa	Western Sub-Saharan Africa
**São Tomé and Príncipe**	Sub-Saharan Africa	Western Sub-Saharan Africa
**Senegal**	Sub-Saharan Africa	Western Sub-Saharan Africa

**Sierra Leone**	Sub-Saharan Africa	Western Sub-Saharan Africa
**Togo**	Sub-Saharan Africa	Western Sub-Saharan Africa

Demographics

For the 204 countries and territories included in analysis, we produced estimates by sex and five-year age group from 1990 to 2020. Additionally, we produced estimates aggregated by age:

- All-age estimates reflect population-weighted average estimates for ages 15 and above.
- Estimates aggregated over specific age groups, for example estimates for ages 15-39, 40-64, and 65+, reflect population-weighted average estimates over the specified age range.
- Age-standardised estimates reflect weighting according to the GBD population standard.

Relative Risks

Systematic Review

We updated previously published systematic reviews conducted as part of the Global Burden of Disease Study for the six alcohol-attributable outcomes accounting for the largest number of global disability-adjusted life years: ischemic heart disease, ischemic stroke, intracerebral hemorrhage, diabetes mellitus type II, lower respiratory infection, and tuberculosis.

We utilized the following inclusion criteria for our review of the MEDLINE database:

- Studies published between 01/01/1970 and 12/31/2019
- Cohort and case-control studies
- Describe an association between alcohol use and a GBD outcome
- Utilize a continuous dose the exposure of alcohol consumption
- Report an effect size estimate (relative risk, hazard ratio, or odds ratio) with uncertainty

When both unadjusted and adjusted analyses were reported, we extracted the adjusted analysis. We also extracted the complete list of confounders included in each of the models, as well as metadata on study design, sample representativeness, and measurement of both exposure variables and outcomes. The set of confounders varied across the studies included in our meta-regressions, and there was substantial heterogeneity in study designs. We account for these differences in our meta-regression, with the final estimated risk curve reflecting adjusted relative risks for a general population.

Search strings and PRISMA diagrams are included in subsequent sections of the appendix.

Estimating Dose-Response Relative Risk Curves

We utilized the meta-regression – Bayesian, regularized, trimmed (MR-BRT) meta-regression tool to update relative risk curves for the six outcomes with the largest burden. We utilized previously published relative risk curves modeled using DisMod ODE for all other health outcomes.

The MR-BRT meta-regression tool produces relative risk curves using a five-stage analysis strategy. In the first stage, a mean dose- log response curve is estimated using a B-spline. Importantly, this strategy does not assume that the log relative risk has a linear relationship with exposure, which tends to overestimate the relative risk at high exposures. This stage uses data on exposure ranges and relative risk to produce a mean dose-response relative risk curve, which is called the "signal". The estimation of the signal imposes spline shape constraints, likelihood-based trimming to remove outliers, and ensemble knot placement. In the table below, we list each risk outcome pair that is updated in GBD 2020 along with several of the key modeling parameters.

MR-BRT splines and priors by type of risk

Risk-outcome	Type of risk	Spline degree, # interior knots	Priors & constraints
Ischemic heart disease	J-shaped	Quadratic, 2 I knots	No monotonicity constraint
Ischemic stroke	J-shaped	Quadratic, 3 I knots	No monotonicity constraint, right linear tail
Intracerebral hemorrhage	J-shaped	Cubic, 3 I knots	No monotonicity constraint, right linear tail
Type II diabetes mellitus	J-shaped	Cubic, 3 I knots	No monotonicity constraint, right linear tail
Tuberculosis	Harmful	Quadratic, 3 I knots	Monotonic increasing, right linear tail, Gaussian max derivative prior on the right tail (0, 0.001)
Lower Respiratory Infection	Harmful	Quadratic, 3 I knots	Monotonic increasing, right linear tail, Gaussian max derivative prior on the right tail (0, 0.001)

Stages 2 and 3 adjust for aspects of study design that contribute to potential bias in the reported relative risks. In stage 2, the signal curve is used to fit a linear mixed effects model for relative risk, to obtain a posterior variance that is used as a prior in the next step. In stage 3, a linear model is fit in which the potential bias covariates are specified as indicator variables that modify the slope of the signal curve. A generalized Lasso approach that penalizes the sum of the absolute values of the bias covariate multipliers β is used to select the bias covariates that are included in subsequent stages. Covariates are added to the model based on the order of their βs and their coefficients are tested for statistical significance. If coefficients are significant they remain in the model. As soon as any coefficient fails to be significant in the overall model, the selection process is terminated and a list of significant bias covariates is returned The bias covariates we tested are shown in the table below:

Covariates tested using MR BRT Lasso Approach

Covariates	Reference Description
cv_sick_quitters	The study adequately adjusted for a sick quitter effect
cv_non_drinkers	The study reference group was non-drinkers
percent_male	The percent of the sample that was male
cv_older	The study sample represents individuals 50 years or older
cv_subpopulation	The outcome is reported for a subpopulation within a given country or territory
cv_outcome_selfreport	The outcome is self-reported
cv_exposure_study	The outcome only measured at baseline
cv_rep_geography	The study sample is geographically representative
cv_rep_prevalent_disease	The study evaluates mortality among those who have already developed a given outcome
cv_incidence	The study outcome was incidence or incidence and mortality combined. Relative risk curves represent incidence and mortality.
cv_mortality	The study outcome was mortality or incidence and mortality combined. Relative risk curves represent incidence and mortality.
cv_adjusted_1	The study was adjusted for age or sex
cv_adjusted_2	The study was adjusted for age and sex
cv_adjusted_3	The study was adjusted for age, sex, and smoking
cv_adjusted_4	The study was adjusted for age, sex, smoking, and one additional covariate
cv_adjusted_5	The study was adjusted for age, sex, smoking, and two or more additional covariates
cv_hypertension*	The study adjusts for hypertension
cv_hypercholesterolemia*	The study adjusts for hypercholesterolemia
cv_definition*	The disease outcome is identical to the GBD definition

* covariates tested only for ischemic heart disease, ischemic stroke, and intracerebral hemorrhage curve estimation

In stage 4, the signal model is first adjusted for the bias covariates selected in stage 3. We use this adjusted model to estimate the between study heterogeneity, parameterized by γ. In some cases, the γ estimate may be 0 simply from lack of data, so we implemented the Fisher Scoring correction to γ, which corrects for sparse-data situations. The Fisher Scoring correction uses a quantile of γ, which is sensitive to the number of studies, study design, and reported uncertainty. In stage 5, we use a modified trim-and-fill method and Egger regression to test for potential publication bias. The bias covariates selected in stage 3, as well as the mean γ solution, are shown in the table below.

MR-BRT parameters by risk-outcome pair

**Risk-outcome**	**Type of risk**	**Selected covariates**	**Mean γ solution**
Ischemic heart disease	J-shaped	cv_incidence	0.158
Ischemic stroke	J-shaped	cv_incidence	0.234
Intracerebral hemorrhage	J-shaped	cv_adjusted_2, cv_adjusted_1	0.09
Type II diabetes mellitus	J-shaped	None	0.117
Tuberculosis	Harmful	cv_sick_quitters, cv_incidence	19.488
Lower Respiratory Infection	Harmful	None	0

Theoretical Minimum Risk Exposure and Non-Drinker Equivalence Levels

Computing aggregate relative risks

The theoretical minimum risk exposure and non-drinker equivalence levels are based on aggregate, burden weighted relative risk curves across health outcomes associated with alcohol use. Aggregate relative risk curves were constructed by region, age group, sex, and year to reflect differing cause compositions across these dimensions. The formula for constructing the aggregate relative risk curves is:

$$log(RR)_{w,e,l,y,a,s,} = \sum_i^{\omega} \log{(RR_{i,e})} * \frac{DALY_{i,l,y,a,s}}{\sum_i^{\omega} DALY_{i,l,y,a,s}}$$

Where:

ω *is the set of causes associated with alcohol, i is a given cause from that set, e is a given exposure level in*

units of grams per day, l is a location, y is a year, s is a sex, a is an age group, DALY is a DALY rate for a given cause, and RR is the dose response curve for a given cause

DALY rates were based on results from GBD 2020.

Theoretical Minimum Risk Exposure Level

The theoretical minimum risk exposure level of alcohol use is the minimum of this aggregate-risk curve, which will vary by region, age group, sex, and year:

$$TMREL = argmin\ log(RR)_{\omega,l,y,a,s}$$

The TMREL is the exposure level that minimizes the risk of suffering burden from any given cause related to alcohol. We weight the risk for a particular cause in our aggregation by the proportion of DALYs due to that cause (e.g., since more observed people die from ischaemic heart disease [IHD], we weight the risk for IHD more in the

above calculation of average risk compared to, say, diabetes, even if both have the same relative risk for a given level of consumption).

Non-drinker Equivalence Level

The non-drinker equivalence level is the level of consumption at which health loss is equivalent to that of non-drinkers. Consumption of alcohol in excess of this level is harmful compared to abstinence.

Population Attributable Fractions

We calculated PAFs by setting the relative risk of alcohol consumption among abstainers and drinkers consuming alcohol below the TMREL to be 1. We then calculated PAFs for drinkers consuming alcohol in excess of the TMREL. For each location, age, sex, year, and cause, we defined the PAF as:

$$PAF(x) = \frac{P_A + \int_0^{TMREL} P(x)\ dx + \int_{TMREL}^{100} P(x) * RR_C(x)\ dx - RR_C(TMREL)}{P_A + \int_0^{TMREL} P(x)\ dx + \int_{TMREL}^{100} P(x) * RR_C(x)\ dx} \qquad P(x) = P_C * \Gamma(p)$$

where:

P_C is the prevalence of current drinkers, P_A is the prevalence of abstainers, and p are parameters determined by the mean and standard deviation of exposure for that location, age, sex, and year; RR_C is the global relative risk function for current drinkers for a given cause, and TMREL is the theoretical minimum exposure level for that location's region, age, sex, and year

We performed the above equation for 1000 draws of the exposure and relative risk models. We then used the complement of the estimated PAF draws to calculate risk-deleted DALY rates as alternative weights as a sensitivity analysis. .

Sensitivity Analyses

Our weighted attributable-cause relative risk curves were based on only 22 out of 24 health outcomes since no relative risk curves could be computed for alcohol use disorder or alcoholic cardiomyopathy due to lack of studies on dose-response relative risks. To assess whether inclusion of these two outcomes could potentially impact the TMREL and NDE levels, we conducted a second sensitivity analysis in which we assumed that the risk of these outcomes was equal to a relative risk of three (scenario A), five (scenario B), and 10 (scenario C) at exposures in excess of four standard drinks per day, the lowest threshold for binge drinking found. At exposure levels lower than this, we assumed the risk increased linearly from a relative risk of 1 at zero standard drinks per day, a conservative assumption that likely overestimates risk at low-levels of alcohol consumption. We then used these hypothetical relative risk curves to re-compute a TMREL that reflects all 24 alcohol-associated outcomes.

Search Strings and PRISMA Diagrams

Diabetes Mellitus

Search String: (alcoholic beverage[MeSH Terms] OR drinking behavior[MeSH Terms] OR "alcohol"[Title/Abstract])
AND
(Diabetes Mellitus, Type 2[MeSH Terms] OR "diabetes"[Title/Abstract])
AND
(Risk[MeSH Terms] OR Odds Ratio[MeSH Terms] OR "risk"[Title/Abstract] OR "odds ratio"[Title/Abstract] OR "cross-product ratio"[Title/Abstract] OR "hazards ratio"[Title/Abstract] OR "hazard ratio"[Title/Abstract])
AND ("1970/01/01"[PDat] : "2019/12/31"[PDat])
AND (English[LA])
NOT (animals[MeSH Terms] NOT Humans[MeSH Terms])

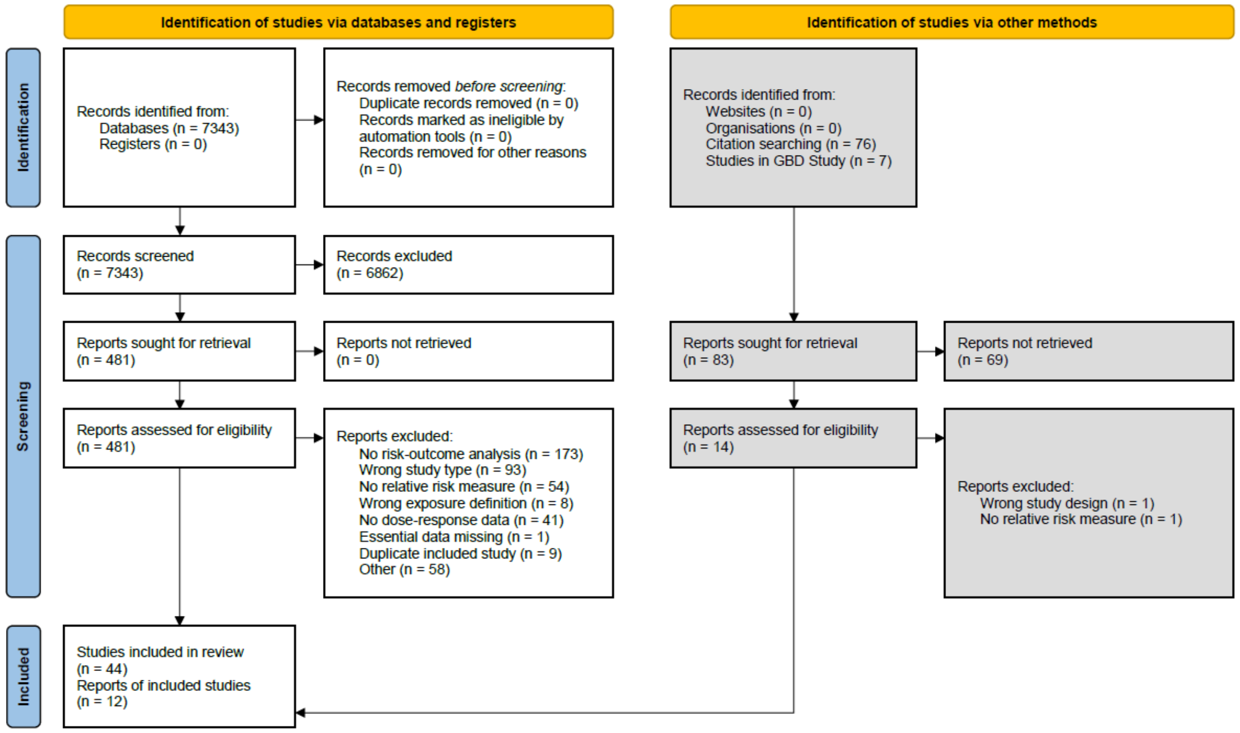

From: Page MJ, McKenzie JE, Bossuyt PM, Boutron I, Hoffmann TC, Mulrow CD, et al. The PRISMA 2020 statement: an updated guideline for reporting systematic reviews. BMJ 2021;372:n71. doi: 10.1136/bmj.n71. For more information, visit: http://www.prisma-statement.org/

References for Diabetes Mellitus

Abbasi A, Corpeleijn E, Gansevoort RT, Gans RO, Hillege HL, Stolk RP, Navis G, Bakker SJ, Dullaart RP. Role of HDL cholesterol and estimates of HDL particle composition in future development of type 2 diabetes in the general population: the PREVEND study. J Clin Endocrinol Metab. 2013; 98(8): E1352-9.

Agardh EE, Lundin A, Lager A, Allebeck P, Koupil I, Andreasson S, Östenson CG, Danielsson AK. Alcohol and type 2 diabetes: The role of socioeconomic, lifestyle and psychosocial factors. Scand J Public Health. 2019; 47(4): 408-416.

Beulens JW, Stolk RP, van der Schouw YT, Grobbee DE, Hendriks HF, Bots ML. Alcohol consumption and risk of type 2 diabetes among older women. Diabetes Care. 2005; 28(12): 2933-8.

Beulens JW, van der Schouw YT, Bergmann MM, Rohrmann S, Schulze MB, Buijsse B, Grobbee DE, Arriola L, Cauchi S, Tormo MJ, Allen NE, van der A DL, Balkau B, Boeing H, Clavel-Chapelon F, de Lauzon-Guillan B, Franks P, Froguel P, Gonzales C, Halkjaer J, Huerta JM, Kaaks R, Key TJ, Khaw KT, Krogh V, Molina-Montes E, Nilsson P, Overvad K, Palli D, Panico S, Ramon Quiros J, Rolandsson O, Ronaldsson O, Romieu I, Romaguera D, Sacerdote C, Sanchez MJ, Spijkerman AM, Teucher B, Tjonneland A, Tumino R, Sharp S, Forouhi NG, Langenberg C, Feskens EJ, Riboli E, Wareham NJ. Alcohol consumption and risk of type 2 diabetes in European men and women: influence of beverage type and body size The EPIC-InterAct study. J Intern Med. 2012; 272(4): 358-70.

Boggs DA, Rosenberg L, Ruiz-Narvaez EA, Palmer JR. Coffee, tea, and alcohol intake in relation to risk of type 2 diabetes in African American women. Am J Clin Nutr. 2010; 92(4): 960-6.

Burke V, Zhao Y, Lee AH, Hunter E, Spargo RM, Gracey M, Smith RM, Beilin LJ, Puddey IB. Predictors of type 2 diabetes and diabetes-related hospitalisation in an Australian Aboriginal cohort. Diabetes Res Clin Pract. 2007; 78(3): 360-8.

Carlsson S, Hammar N, Grill V, Kaprio J. Alcohol consumption and the incidence of type 2 diabetes: a 20-year follow-up of the Finnish twin cohort study. Diabetes Care. 2003; 26(10): 2785-90.

Conway BN, Han X, Munro HM, Gross AL, Shu XO, Hargreaves MK, Zheng W, Powers AC, Blot WJ. The obesity epidemic and rising diabetes incidence in a low-income racially diverse southern US cohort. PLoS One. 2018; 13(1): e0190993.

Cullmann M, Hilding A, Ostenson CG. Alcohol consumption and risk of pre-diabetes and type 2 diabetes development in a Swedish population. Diabet Med. 2012; 29(4): 441-52.

de Vegt F, Dekker JM, Groeneveld WJ, Nijpels G, Stehouwer CD, Bouter LM, Heine RJ. Moderate alcohol consumption is associated with lower risk for incident diabetes and mortality: the Hoorn Study. Diabetes Res Clin Pract. 2002; 57(1): 53-60.

Djousse L, Biggs ML, Mukamal KJ, Siscovick DS. Alcohol consumption and type 2 diabetes among older adults: the Cardiovascular Health Study. Obesity (Silver Spring). 2007; 15(7): 1758-65.

Dow C, Balkau B, Bonnet F, Mancini F, Rajaobelina K, Shaw J, Magliano DJ, Fagherazzi G. Strong adherence to dietary and lifestyle recommendations is associated with decreased type 2 diabetes risk in the AusDiab cohort study. Prev Med. 2019; 123: 208-216.

Feskens EJ, Kromhout D. Cardiovascular risk factors and the 25-year incidence of diabetes mellitus in middle-aged men. The Zutphen Study. Am J Epidemiol. 1989; 130(6): 1101-8.

Gurwitz JH, Field TS, Glynn RJ, Manson JE, Avorn J, Taylor JO, Hennekens CH. Risk factors for non-insulin-dependent diabetes mellitus requiring treatment in the elderly. J Am Geriatr Soc. 1994; 42(12): 1235-40.

Han T, Zhang S, Duan W, Ren X, Wei C, Sun C, Li Y. Eighteen-year alcohol consumption trajectories and their association with risk of type 2 diabetes and its related factors: the China Health and Nutrition Survey. Diabetologia. 2019; 62(6): 970-980.

He X, Rebholz CM, Daya N, Lazo M, Selvin E. Alcohol consumption and incident diabetes: The Atherosclerosis Risk in Communities (ARIC) study. Diabetologia. 2019; 62(5): 770-778.

Heianza Y, Arase Y, Saito K, Tsuji H, Fujihara K, Hsieh SD, Kodama S, Shimano H, Yamada N, Hara S, Sone H. Role of alcohol drinking pattern in type 2 diabetes in Japanese men: the Toranomon Hospital Health Management Center Study 11 (TOPICS 11). Am J Clin Nutr. 2013; 97(3): 561-8.

Hodge AM, English DR, O'Dea K, Giles GG. Alcohol intake, consumption pattern and beverage type, and the risk of Type 2 diabetes. Diabet Med. 2006; 23(6): 690-7.

Hu FB, Manson JE, Stampfer MJ, Colditz G, Liu S, Solomon CG, Willett WC. Diet, lifestyle, and the risk of type 2 diabetes mellitus in women. N Engl J Med. 2001; 345(11): 790-7.

Hu G, Jousilahti P, Peltonen M, Bidel S, Tuomilehto J. Joint association of coffee consumption and other factors to the risk of type 2 diabetes: a prospective study in Finland. Int J Obes (Lond). 2006; 30(12): 1742-9.

Imamura F, Lichtenstein AH, Dallal GE, Meigs JB, Jacques PF. Confounding by dietary patterns of the inverse association between alcohol consumption and type 2 diabetes risk. Am J Epidemiol. 2009; 170(1): 37-45.

Jee SH, Foong AW, Hur NW, Samet JM. Smoking and risk for diabetes incidence and mortality in Korean men and women. Diabetes Care. 2010; 33(12): 2567-72.

Joosten MM, Chiuve SE, Mukamal KJ, Hu FB, Hendriks HF, Rimm EB. Changes in alcohol consumption and subsequent risk of type 2 diabetes in men. Diabetes. 2011; 60(1): 74-9.

Joosten MM, Grobbee DE, van der A DL, Verschuren WM, Hendriks HF, Beulens JW. Combined effect of alcohol consumption and lifestyle behaviors on risk of type 2 diabetes. Am J Clin Nutr. 2010; 91(6): 1777-83.

Kawakami N, Takatsuka N, Shimizu H, Ishibashi H. Effects of smoking on the incidence of non-insulin-dependent diabetes mellitus. Replication and extension in a Japanese cohort of male employees. Am J Epidemiol. 1997; 145(2): 103-9.

Kerr WC, Williams E, Li L, Lui CK, Ye Y, Greenfield TK, Lown EA. Alcohol use patterns and risk of diabetes onset in the 1979 National Longitudinal Survey of Youth Cohort. Prev Med. 2018; 109: 22-27.

Koloverou E, Panagiotakos DB, Pitsavos C, Chrysohoou C, Georgousopoulou EN, Metaxa V, Stefanadis C. Effects of alcohol consumption and the metabolic syndrome on 10-year incidence of diabetes: the ATTICA study. Diabetes Metab. 2015; 41(2): 152-9.

Kotronen A, Laaksonen MA, Heliövaara M, Reunanen A, Tuomilehto J, Yki- Järvinen H, Peltonen M, Knekt P. Fatty liver score and 15-year incidence of type 2 diabetes. Hepatol Int. 2013; 7(2): 610-21.

Lee DH, Ha MH, Kim JH, Christiani DC, Gross MD, Steffes M, Blomhoff R, Jacobs DR. Gamma-glutamyltransferase and diabetes--a 4 year follow-up study. Diabetologia. 2003; 46(3): 359-64.

Lee DY, Yoo MG, Kim HJ, Jang HB, Kim JH, Lee HJ, Park SI. Association between alcohol consumption pattern and the incidence risk of type 2 diabetes in Korean men: A 12-years follow-up study. Sci Rep. 2017; 7(1): 7322.

Lv J, Yu C, Guo Y, Bian Z, Yang L, Chen Y, Hu X, Hou W, Chen J, Chen Z, Qi L, Li L, China Kadoorie Biobank Collaborative Group. Adherence to a Healthy Lifestyle and the Risk of Type 2 Diabetes in Chinese Adults. Int J Epidemiol. 2017; 46(5): 1410-20.

Marques-Vidal P, Vollenweider P, Waeber G. Alcohol consumption and incidence of type 2 diabetes. Results from the CoLaus study. Nutr Metab Cardiovasc Dis. 2015; 25(1): 75-84.

Maty SC, James SA, Kaplan GA. Life-course socioeconomic position and incidence of diabetes mellitus among blacks and whites: the Alameda County Study, 1965-1999. 2010; 100(1): 137-45.

Maty SC, Lynch JW, Raghunathan TE, Kaplan GA. Childhood socioeconomic position, gender, adult body mass index, and incidence of type 2 diabetes mellitus over 34 years in the Alameda County Study. Am J Public Health. 2008; 98(8): 1486-94.

Meisinger C, Thorand B, Schneider A, Stieber J, Doring A, Lowel H. Sex differences in risk factors for incident type 2 diabetes mellitus: the MONICA Augsburg cohort study. Arch Intern Med. 2002; 162(1): 82-9.

Nagaya T, Yoshida H, Takahashi H, Kawai M. Resting heart rate and blood pressure, independent of each other, proportionally raise the risk for type-2 diabetes mellitus. Int J Epidemiol. 2010; 39(1): 215-22.

Ng R, Sutradhar R, Yao Z, Wodchis WP, Rosella LC. Smoking, drinking, diet and physical activity-modifiable lifestyle risk factors and their associations with age to first chronic disease. Int J Epidemiol. 2019.

Onat A, Hergenc G, Kucukdurmaz Z, Ugur M, Kaya Z, Can G, Yuksel H. Moderate and heavy alcohol consumption among Turks: long-term impact on mortality and cardiometabolic risk. Turk Kardiyol Dern Ars. 2009; 37(2): 83-90.

Rasouli B, Ahlbom A, Andersson T, Grill V, Midthjell K, Olsson L, Carlsson S. Alcohol consumption is associated with reduced risk of Type 2 diabetes and autoimmune diabetes in adults: results from the Nord-Trondelag health study. Diabet Med. 2013; 30(1): 56-64.

Saremi A, Hanson RL, Tulloch-Reid M, Williams DE, Knowler WC. Alcohol consumption predicts hypertension but not diabetes. J Stud Alcohol. 2004; 65(2): 184-90.

Sato KK, Hayashi T, Harita N, Koh H, Maeda I, Endo G, Nakamura Y, Kambe H, Kiyotaki C. Relationship between drinking patterns and the risk of type 2 diabetes: the Kansai Healthcare Study. J Epidemiol Community Health. 2012; 66(6): 507-11.

Sawada SS, Lee IM, Naito H, Noguchi J, Tsukamoto K, Muto T, Higaki Y, Tanaka H, Blair SN. Long-term trends in cardiorespiratory fitness and the incidence of type 2 diabetes. Diabetes Care. 2010; 33(6): 1353-7.

Shi L, Shu XO, Li H, Cai H, Liu Q, Zheng W, Xiang YB, Villegas R. Physical activity, smoking, and alcohol consumption in association with incidence of type 2 diabetes among middle-aged and elderly Chinese men. PLoS One. 2013; 8(11): e77919.

Steele CJ, Schöttker B, Marshall AH, Kouvonen A, O'Doherty MG, Mons U, Saum KU, Boffetta P, Trichopoulou A, Brenner H, Kee F. Education achievement and type 2 diabetes-what mediates the relationship in older adults? Data from the ESTHER study: a population-based cohort study. BMJ Open. 2017; 7(4): e013569.

Steinbrecher A, Morimoto Y, Heak S, Ollberding NJ, Geller KS, Grandinetti A, Kolonel LN, Maskarinec G. The preventable proportion of type 2 diabetes by ethnicity: the multiethnic cohort. Ann Epidemiol. 2011; 21(7): 526-35.

Stringhini S, Tabak A, Akbaraly TN, Sabia S, Shipley MJ, Marmot MG, Brunner EJ, Batty GD, Bovet P, Kivimäki M. Contribution of modifiable risk factors to social inequalities in type 2 diabetes: prospective Whitehall II cohort study. BMJ. 2012; 345: e5452.

Strodl E, Kenardy J. Psychosocial and non-psychosocial risk factors for the new diagnosis of diabetes in elderly women. Diabetes Res Clin Pract. 2006; 74(1): 57-65.

Teratani T, Morimoto H, Sakata K, Oishi M, Tanaka K, Nakada S, Nogawa K, Suwazono Y. Dose-response relationship between tobacco or alcohol consumption and the development of diabetes mellitus in Japanese male workers. Drug Alcohol Depend. 2012; 125(3): 276-82.

Tsumura K, Hayashi T, Suematsu C, Endo G, Fujii S, Okada K. Daily alcohol consumption and the risk of type 2 diabetes in Japanese men: the Osaka Health Survey. Diabetes Care. 1999; 22(9): 1432-7.

Waki K, Noda M, Sasaki S, Matsumura Y, Takahashi Y, Isogawa A, Ohashi Y, Kadowaki T, Tsugane S, JPHC Study Group. Alcohol consumption and other risk factors for self-reported diabetes among middle-aged Japanese: a population-based prospective study in the JPHC study cohort I. Diabet Med. 2005; 22(3): 323-31.

Wang CS, Chang TT, Yao WJ, Wang ST, Chou P. The impact of smoking on incident type 2 diabetes in a cohort with hepatitis B but not hepatitis C infection. J Viral Hepat. 2017; 24(12): 1114-1120.

Wannamethee SG, Camargo CA, Manson JE, Willett WC, Rimm EB. Alcohol drinking patterns and risk of type 2 diabetes mellitus among younger women. Arch Intern Med. 2003; 163(11): 1329-36.

Wannamethee SG, Shaper AG, Perry IJ, Alberti KG. Alcohol consumption and the incidence of type II diabetes. J Epidemiol Community Health. 2002; 56(7): 542-8.

Wei M, Gibbons LW, Mitchell TL, Kampert JB, Blair SN. Alcohol intake and incidence of type 2 diabetes in men. Diabetes Care. 2000; 23(1): 18-22.

Yatsuya H, Li Y, Hirakawa Y, Ota A, Matsunaga M, Haregot HE, Chiang C, Zhang Y, Tamakoshi K, Toyoshima H, Aoyama A. A Point System for Predicting 10-Year Risk of Developing Type 2 Diabetes Mellitus in Japanese Men: Aichi Workers' Cohort Study. J Epidemiol. 2018; 28(8): 347-352.

Zelle DM, Agarwal PK, Ramirez JL, van der Heide JJ, Corpeleijn E, Gans RO, Navis G, Bakker SJ. Alcohol consumption, new onset of diabetes after transplantation, and all-cause mortality in renal transplant recipients. Transplantation. 2011; 92(2): 203-9.

Intracerebral Hemorrhage

Search String: (alcoholic beverage[MeSH Terms] OR drinking behavior[MeSH Terms] OR "alcohol"[Title/Abstract] NOT (alcohols[MeSH Terms] NOT drinking behavior[MeSH Terms]))
AND
(brain infarction[MeSH Terms] OR stroke[MeSH Terms] OR intracranial hemorrhages[MeSH Terms] OR "stroke"[Title/Abstract] OR "brain infarction"[Title/Abstract] OR "cerebral infarction"[Title/Abstract] OR "intracerebral hemorrhage"[Title/Abstract] OR "intracerebral haemorrhage"[Title/Abstract] OR "subarachnoid hemorrhage"[Title/Abstract] OR "subarachnoid haemorrhage"[Title/Abstract])
AND
(Case-Control Studies[MeSH Terms] OR Cross-Over Studies[MeSH Terms] OR Cohort Studies[MeSH Terms] OR Systematic Review[Publication Type] OR Meta-Analysis[Publication Type] OR "systematic review"[Title/Abstract] OR "meta-analysis"[Title/Abstract] OR "cohort"[Title/Abstract] OR "cross-over"[Title/Abstract] OR "crossover"[Title/Abstract] OR "case-control"[Title/Abstract] OR "prospective"[Title/Abstract] OR "retrospective"[Title/Abstract] OR "longitudinal"[Title/Abstract] OR "follow-up"[Title/Abstract] OR Dose-Response Relationship, Drug[MeSH Terms] OR "dose-response"[Title/Abstract])
AND
(Risk[MeSH Terms] OR Odds Ratio[MeSH Terms] OR "risk"[Title/Abstract] OR "odds ratio"[Title/Abstract] OR "cross-product ratio"[Title/Abstract] OR "hazards ratio"[Title/Abstract] OR "hazard ratio"[Title/Abstract])
AND ("1970/01/01"[PDat] : "2019/12/31"[PDat])
AND (English[LA])
NOT (animals[MeSH Terms] NOT Humans[MeSH Terms])

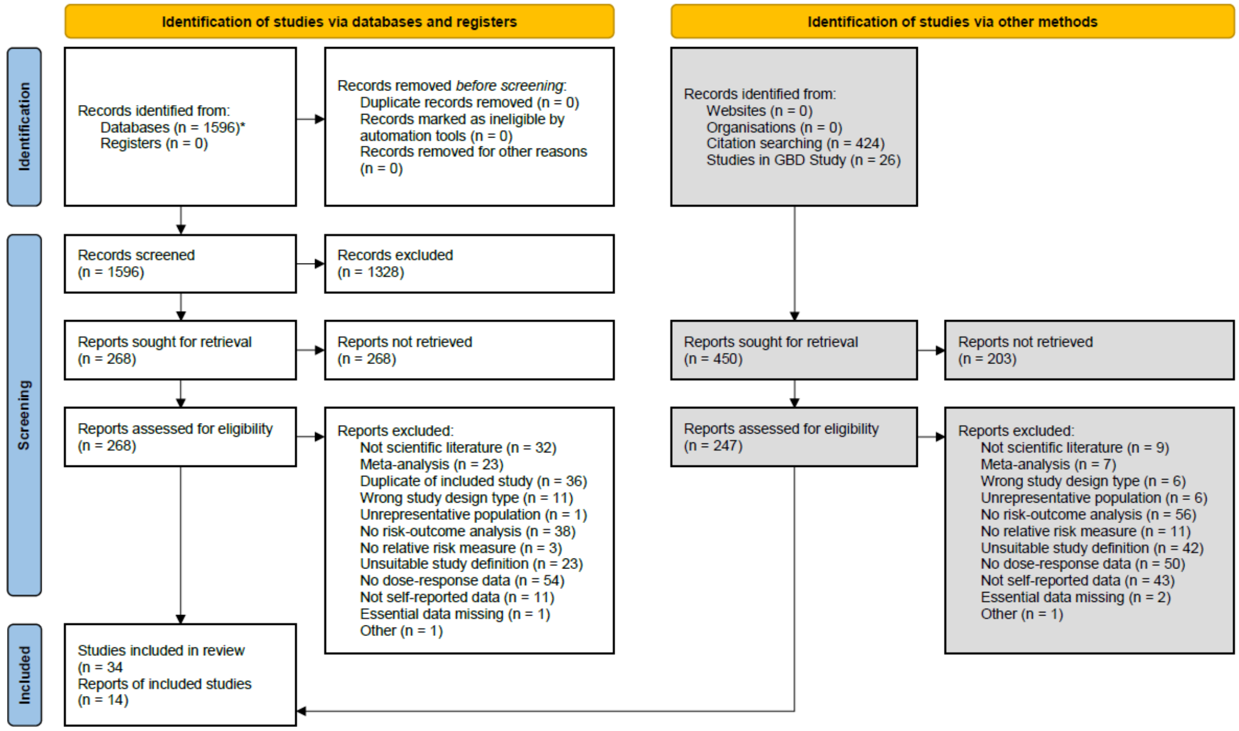

**A single search string was utilized to search for records containing an outcome of stroke, intracerebral hemorrhage, or ischemic stroke, but final included source counts are outcome-specific.*

From: Page MJ, McKenzie JE, Bossuyt PM, Boutron I, Hoffmann TC, Mulrow CD, et al. The PRISMA 2020 statement: an updated guideline for reporting systematic reviews. BMJ 2021;372:n71. doi: 10.1136/bmj.n71. For more information, visit: http://www.prisma-statement.org/

References for Intracerebral Hemorrhage

Bazzano LA, Gu D, Reynolds K, Wu X, Chen CS, Duan X, Chen J, Wildman RP, Klag MJ, He J. Alcohol consumption and risk for stroke among Chinese men. Ann Neurol. 2007; 62(6): 569-78.

Beghi E, Boglium G, Cosso P, Fiorelli G, Lorini C, Mandelli M, Bellini A. Stroke and alcohol intake in a hospital population. A case-control study. Stroke. 1995; 26(9): 1691-6.

Bell S, Daskalopoulou M, Rapsomaniki E, George J, Britton A, Bobak M, Casas JP, Dale CE, Denaxas S, Shah AD, Hemingway H. Association between clinically recorded alcohol consumption and initial presentation of 12 cardiovascular diseases: population based cohort study using linked health records. BMJ. 2017; 356: j909.

Ben-Shlomo Y, Markowe H, Shipley M, Marmot MG. Stroke risk from alcohol consumption using different control groups. Stroke. 1992; 23(8): 1093-8.

Beulens JW, Rimm EB, Ascherio A, Spiegelman D, Hendriks HF, Mukamal KJ. Alcohol consumption and risk for coronary heart disease among men with hypertension. Ann Intern Med. 2007; 146(1): 10-9.

Boffetta P, Garfinkel L. Alcohol drinking and mortality among men enrolled in an American Cancer Society prospective study. Epidemiology. 1990; 1(5): 342-8.

Chen CJ, Brown WM, Moomaw CJ, Langefeld CD, Osborne J, Worrall BB, Woo D, Koch S, ERICH Investigators. Alcohol use and risk of intracerebral hemorrhage. Neurology. 2017; 88(21): 2043-2051.

Chiuve SE, Rexrode KM, Spiegelman D, Logroscino G, Manson JE, Rimm EB. Primary prevention of stroke by healthy lifestyle. Circulation. 2008; 118(9): 947-954.

Christensen AI, Nordestgaard BG, Tolstrup JS. Alcohol Intake and Risk of Ischemic and Haemorrhagic Stroke: Results from a Mendelian Randomisation Study. J Stroke. 2018; 20(2): 218-227.

Donahue RP, Abbott RD, Reed DM, Yano K. Alcohol and hemorrhagic stroke. The Honolulu Heart Program. JAMA. 1986; 255(17): 2311-4.

Duan Y, Wang A, Wang Y, Wang X, Chen S, Zhao Q, Li X, Wu S, Yang L. Cumulative alcohol consumption and stroke risk in men. J Neurol. 2019; 266(9): 2112-2119.

Elkind MSV, Sciacca R, Boden-Albala B, Rundek T, Paik MC, Sacco RL. Moderate alcohol consumption reduces risk of ischemic stroke: the Northern Manhattan Study. Stroke. 2006; 37(1): 13-9.

Falkstedt D, Wolff V, Allebeck P, Hemmingsson T, Danielsson AK. Cannabis, Tobacco, Alcohol Use, and the Risk of Early Stroke: A Population-Based Cohort Study of 45000 Swedish Men. Stroke. 2017; 48(2): 265-270.

Gill JS, Shipley MJ, Tsementzis SA, Hornby RS, Gill SK, Hitchcock ER, Beevers DG. Alcohol consumption--a risk factor for hemorrhagic and non-hemorrhagic stroke. Am J Med. 1991; 90(4): 489-97.

Goldberg RJ, Burchfiel CM, Reed DM, Wergowske G, Chiu D. A prospective study of the health effects of alcohol consumption in middle-aged and elderly men. The Honolulu Heart Program. Circulation. 1994; 89(2): 651-9.

Gordon T, Doyle JT. Drinking and mortality. The Albany Study. Am J Epidemiol. 1987; 125(2): 263-70.

Hart CL, Smith GD. Alcohol consumption and mortality and hospital admissions in men from the Midspan collaborative cohort study. Addiction. 2008; 103(12): 1979-86.

Herman B, Schmitz PI, Leyten AC, Van Luijk JH, Frenken CW, Op De Coul AA, Schulte BP. Multivariate logistic analysis of risk factors for stroke in Tilburg, The Netherlands. Am J Epidemiol. 1983; 118(4): 514-25.

Higashiyama A, Okamura T, Watanabe M, Kokubo Y, Wakabayashi I, Okayama A, Miyamoto Y. Alcohol consumption and cardiovascular disease incidence in men with and without hypertension: the Suita study. Hypertens Res. 2013; 36(1): 58-64.

Ikehara S, Iso H, Toyoshima H, Date C, Yamamoto A, Kikuchi S, Kondo T, Watanabe Y, Koizumi A, Wada Y, Inaba Y, Tamakoshi A. Alcohol consumption and mortality from stroke and coronary heart disease among Japanese men and women: the Japan collaborative cohort study. Stroke. 2008; 39(11): 2936-42.

Ikehara S, Iso H, Yamagishi K, Yamamoto S, Inoue M, Tsugane S. Alcohol consumption, social support, and risk of

stroke and coronary heart disease among Japanese men: the JPHC Study. Alcohol Clin Exp Res. 2009; 33(6): 1025-32.

Iso H, Kitamura A, Shimamoto T, Sankai T, Naito Y, Sato S, Kiyama M, Iida M, Komachi Y. Alcohol intake and the risk of cardiovascular disease in middle-aged Japanese men. Stroke. 1995; 26(5): 767-73.

Jakovljevic B, Stojanov V, Paunovic K, Belojevic G, Milic N. Alcohol consumption and mortality in Serbia: twenty-year follow-up study. Croat Med J. 2004; 45(6): 764-8.

Jimenez M, Chiuve SE, Glynn RJ, Stampfer MJ, Camargo CA Jr, Willett WC, Manson JE, Rexrode KM. Alcohol consumption and risk of stroke in women. Stroke. 2012; 43(4): 939-45.

Jones SB, Loehr L, Avery CL, Gottesman RF, Wruck L, Shahar E, Rosamond WD. Midlife alcohol consumption and the risk of stroke in the Atherosclerosis Risk in Communities Study. Stroke. 2015; 46(11): 3124-30.

Kadlecová P, Andel R, Mikulík R, Handing EP, Pedersen NL. Alcohol consumption at midlife and risk of stroke during 43 years of follow-up: cohort and twin analyses. Stroke. 2015; 46(3): 627-33.

Kivimäki M, Gimeno D, Ferrie JE, Batty GD, Oksanen T, Jokela M, Virtanen M, Salo P, Akbaraly TN, Elovainio M, Pentti J, Vahtera J. Socioeconomic position, psychosocial work environment and cerebrovascular disease among women: the Finnish public sector study. Int J Epidemiol. 2009; 38(5): 1265-71.

Klatsky AL, Armstrong MA, Friedman GD, Sidney S. Alcohol drinking and risk of hemorrhagic stroke. Neuroepidemiology. 2002; 21(3): 115-22.

Klatsky AL, Armstrong MA, Friedman GD. Alcohol use and subsequent cerebrovascular disease hospitalizations. Stroke. 1989; 20(6): 741-6.

Knuiman MW, Vu HT. Risk factors for stroke mortality in men and women: The Busselton Study. J Cardiovasc Risk. 1996; 3(5): 447-52.

Kono S, Ikeda M, Tokudome S, Nishizumi M, Kuratsune M. Alcohol and mortality: a cohort study of male Japanese physicians. Int J Epidemiol. 1986; 15(4): 527-32.

Kurth T, Moore SC, Gaziano JM, Kase CS, Stampfer MJ, Berger K, Buring JE. Healthy lifestyle and the risk of stroke in women. Arch Intern Med. 2006; 166(13): 1403-9.

Larsson SC, Wallin A, Wolk A, Markus HS. Differing association of alcohol consumption with different stroke types: a systematic review and meta-analysis. BMC Med. 2016; 14(1): 178.

Leppala JM, Paunio M, Virtamo J, Fogelholm R, Albanes D, Taylor PR, Heinonen OP. Alcohol consumption and stroke incidence in male smokers. Circulation. 1999; 100(11): 1209-14.

Lu M, Ye W, Adami HO, Weiderpass E. Stroke incidence in women under 60 years of age related to alcohol intake and smoking habit. Cerebrovasc Dis. 2008; 25(6): 517-25.

Millwood IY, Walters RG, Mei XW, Guo Y, Yang L, Bian Z, Bennett DA, Chen Y, Dong C, Hu R, Zhou G, Yu B, Jia W, Parish S, Clarke R, Davey Smith G, Collins R, Holmes MV, Li L, Peto R, Chen Z, China Kadoorie Biobank Collaborative Group. Conventional and genetic evidence on alcohol and vascular disease aetiology: a prospective study of 500000 men and women in China. Lancet. 2019; 393(10183): 1831-1842.

Palmer AJ, Fletcher AE, Bulpitt CJ, Beevers DG, Coles EC, Ledingham JG, Petrie JC, Webster J, Dollery CT. Alcohol intake and cardiovascular mortality in hypertensive patients: report from the Department of Health Hypertension Care Computing Project. J Hypertens. 1995; 13(9): 957-64.

Ricci C, Wood A, Muller D, Gunter MJ, Agudo A, Boeing H, van der Schouw YT, Warnakula S, Saieva C, Spijkerman A, Sluijs I, Tjønneland A, Kyrø C, Weiderpass E, Kühn T, Kaaks R, Sánchez MJ, Panico S, Agnoli C, Palli D, Tumino R, Engström G, Melander O, Bonnet F, Boer JMA, Key TJ, Travis RC, Overvad K, Verschuren WMM, Quirós JR, Trichopoulou A, Papatesta EM, Peppa E, Iribas CM, Gavrila D, Forslund AS, Jansson JH, Matullo G, Arriola L, Freisling H, Lassale C, Tzoulaki I, Sharp SJ, Forouhi NG, Langenberg C, Saracci R, Sweeting M, Brennan P, Butterworth AS, Riboli E, Wareham NJ, Danesh J, Ferrari P. Alcohol intake in relation to non-fatal and fatal coronary heart disease and stroke: EPIC-CVD case-cohort study. BMJ. 2018; 361: k934.

Rist PM, Berger K, Buring JE, Kase CS, Gaziano JM, Kurth T. Alcohol consumption and functional outcome after

stroke in men. Stroke. 2010; 41(1): 141-6.

Rodgers H, Aitken PD, French JM, Curless RH, Bates D, James OF. Alcohol and stroke. A case-control study of drinking habits past and present. Stroke. 1993; 24(10): 1473-7.

Semenciw RM, Morrison HI, Mao Y, Johansen H, Davies JW, Wigle DT. Major risk factors for cardiovascular disease mortality in adults: results from the Nutrition Canada Survey cohort. Int J Epidemiol. 1988; 17(2): 317-24.

Suh I, Jee SH, Kim HC, Nam CM, Kim IS, Appel LJ. Low serum cholesterol and haemorrhagic stroke in men: Korea Medical Insurance Corporation Study. Lancet. 2001; 357(9260): 922-5.

Sull JW, Yi SW, Nam CM, Ohrr H. Binge drinking and mortality from all causes and cerebrovascular diseases in korean men and women: a Kangwha cohort study. Stroke. 2009; 40(9): 2953-8.

Thun MJ, Peto R, Lopez AD, Monaco JH, Henley SJ, Heath CW, Doll R. Alcohol consumption and mortality among middle-aged and elderly U.S. adults. N Engl J Med. 1997; 337(24): 1705-14.

Yang L, Zhou M, Sherliker P, Cai Y, Peto R, Wang L, Millwood I, Smith M, Hu Y, Yang G, Chen Z. Alcohol drinking and overall and cause-specific mortality in China: nationally representative prospective study of 220,000 men with 15 years of follow-up. Int J Epidemiol. 2012; 41(4): 1101-13.

Yusuf S, Joseph P, Rangarajan S, Islam S, Mente A, Hystad P, Brauer M, Kutty VR, Gupta R, Wielgosz A, AlHabib KF, Dans A, Lopez-Jaramillo P, Avezum A, Lanas F, Oguz A, Kruger IM, Diaz R, Yusoff K, Mony P, Chifamba J, Yeates K, Kelishadi R, Yusufali A, Khatib R, Rahman O, Zatonska K, Iqbal R, Wei L, Bo H, Rosengren A, Kaur M, Mohan V, Lear SA, Teo KK, Leong D, O'Donnell M, McKee M, Dagenais G. Modifiable risk factors, cardiovascular disease, and mortality in 155722 individuals from 21 high-income, middle-income, and low-income countries (PURE): a prospective cohort study. Lancet. 2019.

Zhang Y, Tuomilehto J, Jousilahti P, Wang Y, Antikainen R, Hu G. Lifestyle factors on the risks of ischemic and hemorrhagic stroke. Arch Intern Med. 2011; 171(20): 1811-8.

Zodpey SP, Tiwari RR, Kulkarni HR. Risk factors for haemorrhagic stroke: a case-control study. Public Health. 2000; 114(3): 177-82.

Ischemic Stroke

Search String: (alcoholic beverage[MeSH Terms] OR drinking behavior[MeSH Terms] OR "alcohol"[Title/Abstract] NOT (alcohols[MeSH Terms] NOT drinking behavior[MeSH Terms]))
AND
(brain infarction[MeSH Terms] OR stroke[MeSH Terms] OR intracranial hemorrhages[MeSH Terms] OR "stroke"[Title/Abstract] OR "brain infarction"[Title/Abstract] OR "cerebral infarction"[Title/Abstract] OR "intracerebral hemorrhage"[Title/Abstract] OR "intracerebral haemorrhage"[Title/Abstract] OR "subarachnoid hemorrhage"[Title/Abstract] OR "subarachnoid haemorrhage"[Title/Abstract])
AND
(Case-Control Studies[MeSH Terms] OR Cross-Over Studies[MeSH Terms] OR Cohort Studies[MeSH Terms] OR Systematic Review[Publication Type] OR Meta-Analysis[Publication Type] OR "systematic review"[Title/Abstract] OR "meta-analysis"[Title/Abstract] OR "cohort"[Title/Abstract] OR "cross-over"[Title/Abstract] OR "crossover"[Title/Abstract] OR "case-control"[Title/Abstract] OR "prospective"[Title/Abstract] OR "retrospective"[Title/Abstract] OR "longitudinal"[Title/Abstract] OR "follow-up"[Title/Abstract] OR Dose-Response Relationship, Drug[MeSH Terms] OR "dose-response"[Title/Abstract]) AND (Risk[MeSH Terms] OR Odds Ratio[MeSH Terms] OR "risk"[Title/Abstract] OR "odds ratio"[Title/Abstract] OR "cross-product ratio"[Title/Abstract] OR "hazards ratio"[Title/Abstract] OR "hazard ratio"[Title/Abstract])
AND ("1970/01/01"[PDat] : "2019/12/31"[PDat])
AND (English[LA])
NOT (animals[MeSH Terms] NOT Humans[MeSH Terms])

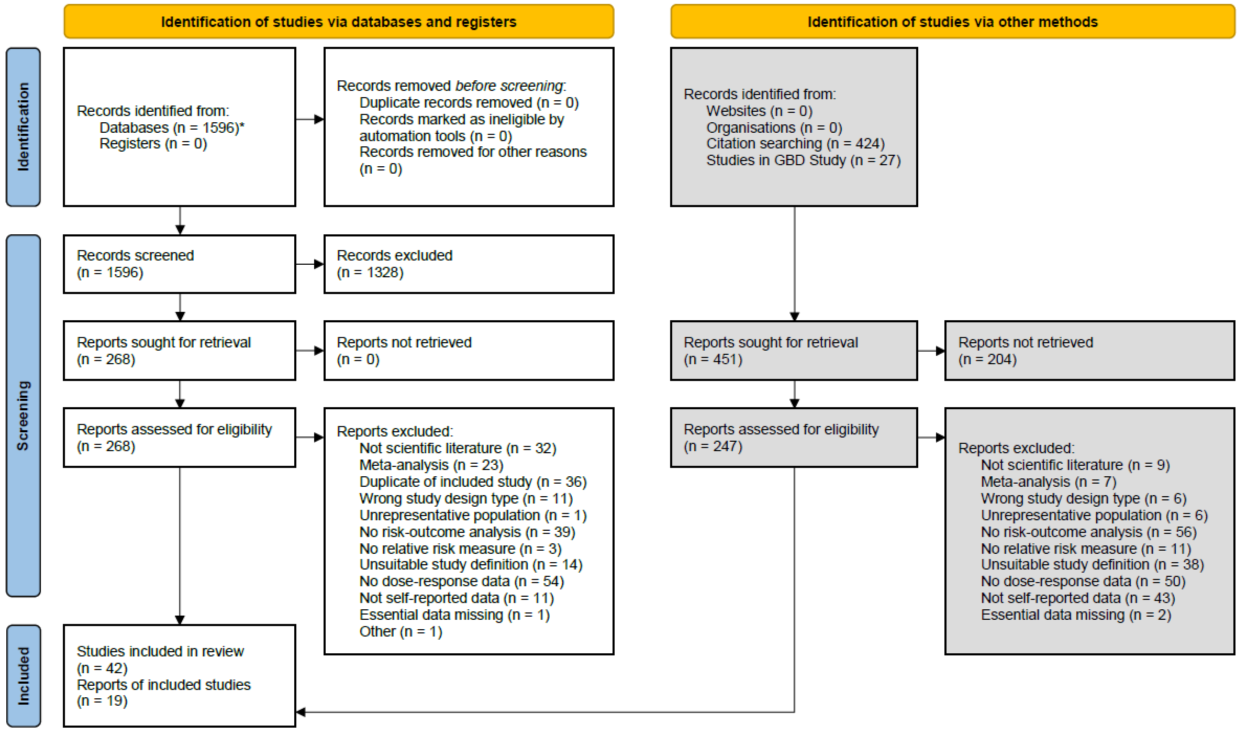

**A single search string was utilized to search for records containing an outcome of stroke, intracerebral hemorrhage, or ischemic stroke, but final included source counts are outcome-specific.*

From: Page MJ, McKenzie JE, Bossuyt PM, Boutron I, Hoffmann TC, Mulrow CD, et al. The PRISMA 2020 statement: an updated guideline for reporting systematic reviews. BMJ 2021;372:n71. doi: 10.1136/bmj.n71. For more information, visit: http://www.prisma-statement.org/

References for Ischemic Stroke

Bazzano LA, Gu D, Reynolds K, Wu X, Chen CS, Duan X, Chen J, Wildman RP, Klag MJ, He J. Alcohol consumption and risk for stroke among Chinese men. Ann Neurol. 2007; 62(6): 569-78.

Beghi E, Boglium G, Cosso P, Fiorelli G, Lorini C, Mandelli M, Bellini A. Stroke and alcohol intake in a hospital population. A case-control study. Stroke. 1995; 26(9): 1691-6.

Bell S, Daskalopoulou M, Rapsomaniki E, George J, Britton A, Bobak M, Casas JP, Dale CE, Denaxas S, Shah AD, Hemingway H. Association between clinically recorded alcohol consumption and initial presentation of 12 cardiovascular diseases: population based cohort study using linked health records. BMJ. 2017; 356: j909.

Ben-Shlomo Y, Markowe H, Shipley M, Marmot MG. Stroke risk from alcohol consumption using different control groups. Stroke. 1992; 23(8): 1093-8.

Beulens JW, Rimm EB, Ascherio A, Spiegelman D, Hendriks HF, Mukamal KJ. Alcohol consumption and risk for coronary heart disease among men with hypertension. Ann Intern Med. 2007; 146(1): 10-9.

Boffetta P, Garfinkel L. Alcohol drinking and mortality among men enrolled in an American Cancer Society prospective study. Epidemiology. 1990; 1(5): 342-8.

Chiuve SE, Rexrode KM, Spiegelman D, Logroscino G, Manson JE, Rimm EB. Primary prevention of stroke by healthy lifestyle. Circulation. 2008; 118(9): 947-954.

Christensen AI, Nordestgaard BG, Tolstrup JS. Alcohol Intake and Risk of Ischemic and Haemorrhagic Stroke: Results from a Mendelian Randomisation Study. J Stroke. 2018; 20(2): 218-227.

Cunningham SA, Mosher A, Judd SE, Matz LM, Kabagambe EK, Moy CS, Howard VJ. Alcohol Consumption and Incident Stroke Among Older Adults. J Gerontol B Psychol Sci Soc Sci. 2018; 73(4): 636-648.

Djousse L, Ellison RC, Beiser A, Scaramucci A, D'Agostino RB, Wolf PA. Alcohol consumption and risk of ischemic stroke: The Framingham Study. Stroke. 2002; 33(4): 907-12.

Donahue RP, Abbott RD, Reed DM, Yano K. Alcohol and hemorrhagic stroke. The Honolulu Heart Program. JAMA. 1986; 255(17): 2311-4.

Duan Y, Wang A, Wang Y, Wang X, Chen S, Zhao Q, Li X, Wu S, Yang L. Cumulative alcohol consumption and stroke risk in men. J Neurol. 2019; 266(9): 2112-2119.

Elkind MSV, Sciacca R, Boden-Albala B, Rundek T, Paik MC, Sacco RL. Moderate alcohol consumption reduces risk of ischemic stroke: the Northern Manhattan Study. Stroke. 2006; 37(1): 13-9.

Falkstedt D, Wolff V, Allebeck P, Hemmingsson T, Danielsson AK. Cannabis, Tobacco, Alcohol Use, and the Risk of Early Stroke: A Population-Based Cohort Study of 45000 Swedish Men. Stroke. 2017; 48(2): 265-270.

Gill JS, Shipley MJ, Tsementzis SA, Hornby RS, Gill SK, Hitchcock ER, Beevers DG. Alcohol consumption--a risk factor for hemorrhagic and non-hemorrhagic stroke. Am J Med. 1991; 90(4): 489-97.

Goldberg RJ, Burchfiel CM, Reed DM, Wergowske G, Chiu D. A prospective study of the health effects of alcohol consumption in middle-aged and elderly men. The Honolulu Heart Program. Circulation. 1994; 89(2): 651-9.

Gordon T, Doyle JT. Drinking and mortality. The Albany Study. Am J Epidemiol. 1987; 125(2): 263-70.

Gorelick PB, Rodin MB, Langenberg P, Hier DB, Costigan J, Gomez I, Spontak S. Is acute alcohol ingestion a risk factor for ischemic stroke? Results of a controlled study in middle-aged and elderly stroke patients at three urban medical centers. Stroke. 1987; 18(2): 359-64.

Gorelick PB, Rodin MB, Langenberg P, Hier DB, Costigan J. Weekly alcohol consumption, cigarette smoking, and the risk of ischemic stroke: results of a case-control study at three urban medical centers in Chicago, Illinois. Neurology. 1989; 39(3): 339-43.

Hajat C, Tilling K, Stewart JA, Lemic-Stojcevic N, Wolfe CD. Ethnic differences in risk factors for ischemic stroke: a European case-control study. Stroke. 2004; 35(7): 1562-7.

Hart CL, Smith GD. Alcohol consumption and mortality and hospital admissions in men from the Midspan

collaborative cohort study. Addiction. 2008; 103(12): 1979-86.

Henrich JB, Horwitz RI. Evidence against the association between alcohol use and ischemic stroke risk. Arch Intern Med. 1989; 149(6): 1413-6.

Herman B, Schmitz PI, Leyten AC, Van Luijk JH, Frenken CW, Op De Coul AA, Schulte BP. Multivariate logistic analysis of risk factors for stroke in Tilburg, The Netherlands. Am J Epidemiol. 1983; 118(4): 514-25.

Higashiyama A, Okamura T, Watanabe M, Kokubo Y, Wakabayashi I, Okayama A, Miyamoto Y. Alcohol consumption and cardiovascular disease incidence in men with and without hypertension: the Suita study. Hypertens Res. 2013; 36(1): 58-64.

Hillbom M, Haapaniemi H, Juvela S, Palomäki H, Numminen H, Kaste M. Recent alcohol consumption, cigarette smoking, and cerebral infarction in young adults. Stroke. 1995; 26(1): 40-5.

Ikehara S, Iso H, Toyoshima H, Date C, Yamamoto A, Kikuchi S, Kondo T, Watanabe Y, Koizumi A, Wada Y, Inaba Y, Tamakoshi A. Alcohol consumption and mortality from stroke and coronary heart disease among Japanese men and women: the Japan collaborative cohort study. Stroke. 2008; 39(11): 2936-42.

Ikehara S, Iso H, Yamagishi K, Yamamoto S, Inoue M, Tsugane S. Alcohol consumption, social support, and risk of stroke and coronary heart disease among Japanese men: the JPHC Study. Alcohol Clin Exp Res. 2009; 33(6): 1025-32.

Iso H, Kitamura A, Shimamoto T, Sankai T, Naito Y, Sato S, Kiyama M, Iida M, Komachi Y. Alcohol intake and the risk of cardiovascular disease in middle-aged Japanese men. Stroke. 1995; 26(5): 767-73.

Jakovljevic B, Stojanov V, Paunovic K, Belojevic G, Milic N. Alcohol consumption and mortality in Serbia: twenty-year follow-up study. Croat Med J. 2004; 45(6): 764-8.

Jimenez M, Chiuve SE, Glynn RJ, Stampfer MJ, Camargo CA Jr, Willett WC, Manson JE, Rexrode KM. Alcohol consumption and risk of stroke in women. Stroke. 2012; 43(4): 939-45.

Jones SB, Loehr L, Avery CL, Gottesman RF, Wruck L, Shahar E, Rosamond WD. Midlife alcohol consumption and the risk of stroke in the Atherosclerosis Risk in Communities Study. Stroke. 2015; 46(11): 3124-30.

Kadlecová P, Andel R, Mikulík R, Handing EP, Pedersen NL. Alcohol consumption at midlife and risk of stroke during 43 years of follow-up: cohort and twin analyses. Stroke. 2015; 46(3): 627-33.

Kivimäki M, Gimeno D, Ferrie JE, Batty GD, Oksanen T, Jokela M, Virtanen M, Salo P, Akbaraly TN, Elovainio M, Pentti J, Vahtera J. Socioeconomic position, psychosocial work environment and cerebrovascular disease among women: the Finnish public sector study. Int J Epidemiol. 2009; 38(5): 1265-71.

Kiyohara Y, Kato I, Iwamoto H, Nakayama K, Fujishima M. The impact of alcohol and hypertension on stroke incidence in a general Japanese population. The Hisayama Study. Stroke. 1995; 26(3): 368-72.

Klatsky AL, Armstrong MA, Friedman GD, Sidney S. Alcohol drinking and risk of hospitalization for ischemic stroke. Am J Cardiol. 2001; 88(6): 703-6.

Klatsky AL, Armstrong MA, Friedman GD. Alcohol use and subsequent cerebrovascular disease hospitalizations. Stroke. 1989; 20(6): 741-6.

Knuiman MW, Vu HT. Risk factors for stroke mortality in men and women: The Busselton Study. J Cardiovasc Risk. 1996; 3(5): 447-52.

Kono S, Ikeda M, Tokudome S, Nishizumi M, Kuratsune M. Alcohol and mortality: a cohort study of male Japanese physicians. Int J Epidemiol. 1986; 15(4): 527-32.

Kurth T, Moore SC, Gaziano JM, Kase CS, Stampfer MJ, Berger K, Buring JE. Healthy lifestyle and the risk of stroke in women. Arch Intern Med. 2006; 166(13): 1403-9.

Larsson SC, Wallin A, Wolk A, Markus HS. Differing association of alcohol consumption with different stroke types: a systematic review and meta-analysis. BMC Med. 2016; 14(1): 178.

Lee SJ, Cho YJ, Kim JG, Ko Y, Hong KS, Park JM, Kang K, Park TH, Park SS, Lee KB, Cha JK, Kim DH, Lee J, Kim JT, Lee J, Lee JS, Jang MS, Han MK, Gorelick PB, Bae HJ, CRCS-5 Investigators. Moderate alcohol intake

reduces risk of ischemic stroke in Korea. Neurology. 2015; 85(22): 1950-6.

Leppala JM, Paunio M, Virtamo J, Fogelholm R, Albanes D, Taylor PR, Heinonen OP. Alcohol consumption and stroke incidence in male smokers. Circulation. 1999; 100(11): 1209-14.

Lu M, Ye W, Adami HO, Weiderpass E. Stroke incidence in women under 60 years of age related to alcohol intake and smoking habit. Cerebrovasc Dis. 2008; 25(6): 517-25.

Malarcher AM, Giles WH, Croft JB, Wozniak MA, Wityk RJ, Stolley PD, Stern BJ, Sloan MA, Sherwin R, Price TR, Macko RF, Johnson CJ, Earley CJ, Buchholz DW, Kittner SJ. Alcohol intake, type of beverage, and the risk of cerebral infarction in young women. Stroke. 2001; 32(1): 77-83.

Millwood IY, Walters RG, Mei XW, Guo Y, Yang L, Bian Z, Bennett DA, Chen Y, Dong C, Hu R, Zhou G, Yu B, Jia W, Parish S, Clarke R, Davey Smith G, Collins R, Holmes MV, Li L, Peto R, Chen Z, China Kadoorie Biobank Collaborative Group. Conventional and genetic evidence on alcohol and vascular disease aetiology: a prospective study of 500000 men and women in China. Lancet. 2019; 393(10183): 1831-1842.

Mukamal KJ, Chung H, Jenny NS, Kuller LH, Longstreth WT, Mittleman MA, Burke GL, Cushman M, Beauchamp NJ, Siscovick DS. Alcohol use and risk of ischemic stroke among older adults: the cardiovascular health study. Stroke. 2005; 36(9): 1830-4.

Nightingale AL, Farmer RD. Ischemic stroke in young women: a nested case-control study using the UK General Practice Research Database. Stroke. 2004; 35(7): 1574-8.

Numminen H, Hillbom M, Juvela S. Platelets, alcohol consumption, and onset of brain infarction. J Neurol Neurosurg Psychiatry. 1996; 61(4): 376-80.

Palmer AJ, Fletcher AE, Bulpitt CJ, Beevers DG, Coles EC, Ledingham JG, Petrie JC, Webster J, Dollery CT. Alcohol intake and cardiovascular mortality in hypertensive patients: report from the Department of Health Hypertension Care Computing Project. J Hypertens. 1995; 13(9): 957-64.

Palomaki H, Kaste M. Regular light-to-moderate intake of alcohol and the risk of ischemic stroke Is there a beneficial effect?. Stroke. 1993; 24(12): 1828-32.

Pikija S, Trkulja V, Malojcic B, Mutzenbach JS, Sellner J. A High Burden of Ischemic Stroke in Regions of Eastern/Central Europe is Largely Due to Modifiable Risk Factors. Curr Neurovasc Res. 2015; 12(4): 341-52.

Ricci C, Wood A, Muller D, Gunter MJ, Agudo A, Boeing H, van der Schouw YT, Warnakula S, Saieva C, Spijkerman A, Sluijs I, Tjønneland A, Kyrø C, Weiderpass E, Kühn T, Kaaks R, Sánchez MJ, Panico S, Agnoli C, Palli D, Tumino R, Engström G, Melander O, Bonnet F, Boer JMA, Key TJ, Travis RC, Overvad K, Verschuren WMM, Quirós JR, Trichopoulou A, Papatesta EM, Peppa E, Iribas CM, Gavrila D, Forslund AS, Jansson JH, Matullo G, Arriola L, Freisling H, Lassale C, Tzoulaki I, Sharp SJ, Forouhi NG, Langenberg C, Saracci R, Sweeting M, Brennan P, Butterworth AS, Riboli E, Wareham NJ, Danesh J, Ferrari P. Alcohol intake in relation to non-fatal and fatal coronary heart disease and stroke: EPIC-CVD case-cohort study. BMJ. 2018; 361: k934.

Rist PM, Berger K, Buring JE, Kase CS, Gaziano JM, Kurth T. Alcohol consumption and functional outcome after stroke in men. Stroke. 2010; 41(1): 141-6.

Rodgers H, Aitken PD, French JM, Curless RH, Bates D, James OF. Alcohol and stroke. A case-control study of drinking habits past and present. Stroke. 1993; 24(10): 1473-7.

Semenciw RM, Morrison HI, Mao Y, Johansen H, Davies JW, Wigle DT. Major risk factors for cardiovascular disease mortality in adults: results from the Nutrition Canada Survey cohort. Int J Epidemiol. 1988; 17(2): 317-24.

Sull JW, Yi SW, Nam CM, Ohrr H. Binge drinking and mortality from all causes and cerebrovascular diseases in korean men and women: a Kangwha cohort study. Stroke. 2009; 40(9): 2953-8.

Syrjänen J, Valtonen VV, Iivanainen M, Kaste M, Huttunen JK. Preceding infection as an important risk factor for ischaemic brain infarction in young and middle aged patients. Br Med J (Clin Res Ed). 1988; 296(6630): 1156-60.

Thun MJ, Peto R, Lopez AD, Monaco JH, Henley SJ, Heath CW, Doll R. Alcohol consumption and mortality among middle-aged and elderly U.S. adults. N Engl J Med. 1997; 337(24): 1705-14.

Yang L, Zhou M, Sherliker P, Cai Y, Peto R, Wang L, Millwood I, Smith M, Hu Y, Yang G, Chen Z. Alcohol

drinking and overall and cause-specific mortality in China: nationally representative prospective study of 220,000 men with 15 years of follow-up. Int J Epidemiol. 2012; 41(4): 1101-13.

Yusuf S, Joseph P, Rangarajan S, Islam S, Mente A, Hystad P, Brauer M, Kutty VR, Gupta R, Wielgosz A, AlHabib KF, Dans A, Lopez-Jaramillo P, Avezum A, Lanas F, Oguz A, Kruger IM, Diaz R, Yusoff K, Mony P, Chifamba J, Yeates K, Kelishadi R, Yusufali A, Khatib R, Rahman O, Zatonska K, Iqbal R, Wei L, Bo H, Rosengren A, Kaur M, Mohan V, Lear SA, Teo KK, Leong D, O'Donnell M, McKee M, Dagenais G. Modifiable risk factors, cardiovascular disease, and mortality in 155722 individuals from 21 high-income, middle-income, and low-income countries (PURE): a prospective cohort study. Lancet. 2019.

Zhang Y, Tuomilehto J, Jousilahti P, Wang Y, Antikainen R, Hu G. Lifestyle factors on the risks of ischemic and hemorrhagic stroke. Arch Intern Med. 2011; 171(20): 1811-8.

Ischemic heart disease

Search String: (alcoholic beverage[MeSH Terms] OR drinking behavior[MeSH Terms] OR "alcohol"[Title/Abstract])
AND
(Coronary Artery Disease[Mesh] OR Myocardial Ischemia[Mesh] OR atherosclerosis[Mesh] OR Coronary Artery Disease[TiAb] OR Myocardial Ischemia[TiAb] OR cardiac ischemia[TiAb] OR silent ischemia[TiAb] OR atherosclerosis[TiAb] OR Ischaemic heart disease[TiAb] OR Ischemic heart disease[TiAb] OR coronary heart disease[TiAb] OR myocardial infarction[TiAb] OR heart attack[TiAb] OR heart infarction[TiAb])
AND
(Risk[MeSH Terms] OR Odds Ratio[MeSH Terms] OR "risk"[Title/Abstract] OR "odds ratio"[Title/Abstract] OR "cross-product ratio"[Title/Abstract] OR "hazards ratio"[Title/Abstract] OR "hazard ratio"[Title/Abstract])
AND ("1970/01/01"[PDat] : "2019/12/31"[PDat])
AND (English[LA])
NOT
(animals[MeSH Terms] NOT Humans[MeSH Terms])

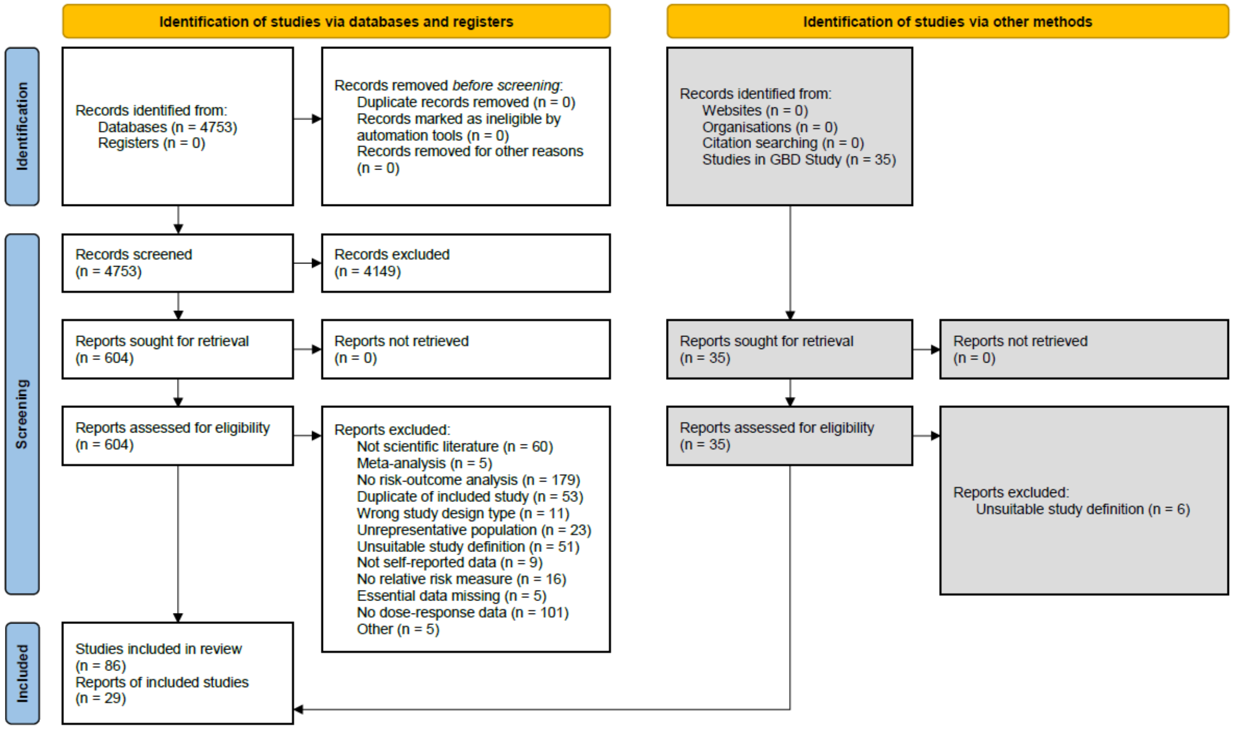

From: Page MJ, McKenzie JE, Bossuyt PM, Boutron I, Hoffmann TC, Mulrow CD, et al. The PRISMA 2020 statement: an updated guideline for reporting systematic reviews. BMJ 2021;372:n71. doi: 10.1136/bmj.n71. For more information, visit: http://www.prisma-statement.org/

References for Ischemic heart disease

Albert CM, Manson JE, Cook NR, Ajani UA, Gaziano JM, Hennekens CH. Moderate alcohol consumption and the risk of sudden cardiac death among US male physicians. Circulation. 1999; 100(9): 944-50.

Arriola L, Martinez-Camblor P, Larranaga N, Basterretxea M, Amiano P, Moreno-Iribas C, Carracedo R, Agudo A, Ardanaz E, Barricarte A, Buckland G, Cirera L, Chirlaque MD, Martinez C, Molina E, Navarro C, Quiros JR, Rodriguez L, Sanchez MJ, Tormo MJ, Gonzalez CA, Dorronsoro M. Alcohol intake and the risk of coronary heart disease in the Spanish EPIC cohort study. Heart. 2010; 96(2): 124-30.

Augustin LS, Gallus S, Tavani A, Bosetti C, Negri E, La Vecchia C. Alcohol consumption and acute myocardial infarction: a benefit of alcohol consumed with meals?. Epidemiology. 2004; 15(6): 767-9.

Bazzano LA, Gu D, Reynolds K, Chen J, Wu X, Chen CS, Duan X, Chen J, He J. Alcohol consumption and risk of coronary heart disease among Chinese men. Int J Cardiol. 2009; 135(1): 78-85.

Bell S, Daskalopoulou M, Rapsomaniki E, George J, Britton A, Bobak M, Casas JP, Dale CE, Denaxas S, Shah AD, Hemingway H. Association between clinically recorded alcohol consumption and initial presentation of 12 cardiovascular diseases: population based cohort study using linked health records. BMJ. 2017; 356: j909.

Bergmann MM, Rehm J, Klipstein-Grobusch K, Boeing H, Schutze M, Drogan D, Overvad K, Tjonneland A, Halkjaer J, Fagherazzi G, Boutron-Ruault MC, Clavel-Chapelon F, Teucher B, Kaaks R, Trichopoulou A, Benetou V, Trichopoulos D, Palli D, Pala V, Tumino R, Vineis P, Beulens JW, Redondo ML, Duell EJ, Molina-Montes E, Navarro C, Barricarte A, Arriola L, Allen NE, Crowe FL, Khaw KT, Wareham N, Romaguera D, Wark PA, Romieu I, Nunes L, Riboli E, Ferrari P. The association of pattern of lifetime alcohol use and cause of death in the European prospective investigation into cancer and nutrition (EPIC) study. Int J Epidemiol. 2013; 42(6): 1772-90.

Beulens JW, Rimm EB, Ascherio A, Spiegelman D, Hendriks HF, Mukamal KJ. Alcohol consumption and risk for coronary heart disease among men with hypertension. Ann Intern Med. 2007; 146(1): 10-9.

Bianchi C, Negri E, La Vecchia C, Franceschi S. Alcohol consumption and the risk of acute myocardial infarction in women. J Epidemiol Community Health. 1993; 47(4): 308-11.

Bobak M, Malyutina S, Horvat P, Pajak A, Tamosiunas A, Kubinova R, Simonova G, Topor-Madry R, Peasey A, Pikhart H, Marmot MG. Alcohol, drinking pattern and all-cause, cardiovascular and alcohol-related mortality in Eastern Europe. Eur J Epidemiol. 2016; 31(1): 21-30.

Boffetta P, Garfinkel L. Alcohol drinking and mortality among men enrolled in an American Cancer Society prospective study. Epidemiology. 1990; 1(5): 342-8.

Brenner H, Rothenbacher D, Bode G, März W, Hoffmeister A, Koenig W. Coronary heart disease risk reduction in a predominantly beer-drinking population. Epidemiology. 2001; 12(4): 390-5.

Britton A, Marmot M. Different measures of alcohol consumption and risk of coronary heart disease and all-cause mortality: 11-year follow-up of the Whitehall II Cohort Study. Addiction. 2004; 99(1): 109-16.

Camargo CA, Stampfer MJ, Glynn RJ, Grodstein F, Gaziano JM, Manson JE, Buring JE, Hennekens CH. Moderate alcohol consumption and risk for angina pectoris or myocardial infarction in U.S. male physicians. Ann Intern Med. 1997; 126(5): 372-5.

Chiuve SE, Rimm EB, Mukamal KJ, Rexrode KM, Stampfer MJ, Manson JE, Albert CM. Light-to-moderate alcohol consumption and risk of sudden cardiac death in women. Heart Rhythm. 2010; 7(10): 1374-80.

Colditz GA, Branch LG, Lipnick RJ, Willett WC, Rosner B, Posner B, Hennekens CH. Moderate alcohol and decreased cardiovascular mortality in an elderly cohort. Am Heart J. 1985; 109(4): 886-9.

Dai J, Mukamal KJ, Krasnow RE, Swan GE, Reed T. Higher usual alcohol consumption was associated with a lower 41-y mortality risk from coronary artery disease in men independent of genetic and common environmental factors: the prospective NHLBI Twin Study. Am J Clin Nutr. 2015; 102(1): 31-9.

Dam MK, Hvidtfeldt UA, Tjønneland A, Overvad K, Grønbæk M, Tolstrup JS. Five year change in alcohol intake and risk of breast cancer and coronary heart disease among postmenopausal women: prospective cohort study. BMJ. 2016; 353: i2314.

de Labry LO, Glynn RJ, Levenson MR, Hermos JA, LoCastro JS, Vokonas PS. Alcohol consumption and mortality in an American male population: recovering the U-shaped curve--findings from the normative Aging Study. J Stud Alcohol. 1992; 53(1): 25-32.

Doll R, Peto R, Boreham J, Sutherland I. Mortality in relation to alcohol consumption: a prospective study among male British doctors. Int J Epidemiol. 2005; 34(1): 199-204.

Dorn JM, Hovey K, Williams BA, Freudenheim JL, Russell M, Nochajski TH, Trevisan M. Alcohol drinking pattern and non-fatal myocardial infarction in women. Addiction. 2007; 102(5): 730-9.

Dyer AR, Stamler J, Paul O, Lepper M, Shekelle RB, McKean H, Garside D. Alcohol consumption and 17-year mortality in the Chicago Western Electric Company study. Prev Med. 1980; 9(1): 78-90.

Ebbert JO, Janney CA, Sellers TA, Folsom AR, Cerhan JR. The association of alcohol consumption with coronary heart disease mortality and cancer incidence varies by smoking history. J Gen Intern Med. 2005; 20(1): 14-20.

Ebrahim S, Lawlor DA, Shlomo YB, Timpson N, Harbord R, Christensen M, Baban J, Kiessling M, Day I, Gaunt T, Davey Smith G. Alcohol dehydrogenase type 1C (ADH1C) variants, alcohol consumption traits, HDL-cholesterol and risk of coronary heart disease in women and men: British Women's Heart and Health Study and Caerphilly cohorts. Atherosclerosis. 2008; 196(2): 871-8.

Fan AZ, Ruan WJ, Chou SP. Re-examining the relationship between alcohol consumption and coronary heart disease with a new lens. Prev Med. 2019; 118: 336-343.

Friedman LA, Kimball AW. Coronary heart disease mortality and alcohol consumption in Framingham. Am J Epidemiol. 1986; 124(3): 481-9.

Fuchs FD, Chambless LE, Folsom AR, Eigenbrodt ML, Duncan BB, Gilbert A, Szklo M. Association between alcoholic beverage consumption and incidence of coronary heart disease in whites and blacks: the Atherosclerosis Risk in Communities Study. Am J Epidemiol. 2004; 160(5): 466-74.

Fumeron F, Betoulle D, Luc G, Behague I, Ricard S, Poirier O, Jemaa R, Evans A, Arveiler D, Marques-Vidal P. Alcohol intake modulates the effect of a polymorphism of the cholesteryl ester transfer protein gene on plasma high density lipoprotein and the risk of myocardial infarction. J Clin Invest. 1995; 96(3): 1664-71.

Gémes K, Janszky I, Laugsand LE, László KD, Ahnve S, Vatten LJ, Mukamal KJ. Alcohol consumption is associated with a lower incidence of acute myocardial infarction: results from a large prospective population-based study in Norway. J Intern Med. 2016; 279(4): 365-75.

Garfinkel L, Boffetta P, Stellman SD. Alcohol and breast cancer: a cohort study. Prev Med. 1988; 17(6): 686-93.

Gaziano JM, Buring JE, Breslow JL, Goldhaber SZ, Rosner B, VanDenburgh M, Willett W, Hennekens CH. Moderate alcohol intake, increased levels of high-density lipoprotein and its subfractions, and decreased risk of myocardial infarction. N Engl J Med. 1993; 329(25): 1829-34.

Genchev GD, Georgieva LM, Weijenberg MP, Powles JW. Does alcohol protect against ischaemic heart disease in Bulgaria? A case-control study of non-fatal myocardial infarction in Sofia. Cent Eur J Public Health. 2001; 9(2): 83-6.

Gigleux I, Gagnon J, St-Pierre A, Cantin B, Dagenais GR, Meyer F, Després JP, Lamarche B. Moderate alcohol consumption is more cardioprotective in men with the metabolic syndrome. J Nutr. 2006; 136(12): 3027-32.

Goldberg RJ, Burchfiel CM, Benfante R, Chiu D, Reed DM, Yano K. Lifestyle and biologic factors associated with atherosclerotic disease in middle-aged men. 20-year findings from the Honolulu Heart Program. Arch Intern Med. 1995; 155(7): 686-94.

Goldberg RJ, Burchfiel CM, Reed DM, Wergowske G, Chiu D. A prospective study of the health effects of alcohol consumption in middle-aged and elderly men. The Honolulu Heart Program. Circulation. 1994; 89(2): 651-9.

Gordon T, Doyle JT. Drinking and coronary heart disease: the Albany Study. Am Heart J. 1985; 110(2): 331-4.

Gun RT, Pratt N, Ryan P, Gordon I, Roder D. Tobacco and alcohol-related mortality in men: estimates from the Australian cohort of petroleum industry workers. Aust N Z J Public Health. 2006; 30(4): 318-24.

Hammar N, Romelsjo A, Alfredsson L. Alcohol consumption, drinking pattern and acute myocardial infarction. A

case referent study based on the Swedish Twin Register. J Intern Med. 1997; 241(2): 125-31.

Harriss LR, English DR, Hopper JL, Powles J, Simpson JA, O'Dea K, Giles GG, Tonkin AM. Alcohol consumption and cardiovascular mortality accounting for possible misclassification of intake: 11-year follow-up of the Melbourne Collaborative Cohort Study. Addiction. 2007; 102(10): 1574-85.

Hart CL, Smith GD. Alcohol consumption and mortality and hospital admissions in men from the Midspan collaborative cohort study. Addiction. 2008; 103(12): 1979-86.

Henderson SO, Haiman CA, Wilkens LR, Kolonel LN, Wan P, Pike MC. Established risk factors account for most of the racial differences in cardiovascular disease mortality. PLoS One. 2007; 2(4): e377.

Hines LM, Stampfer MJ, Ma J, Gaziano JM, Ridker PM, Hankinson SE, Sacks F, Rimm EB, Hunter DJ. Genetic variation in alcohol dehydrogenase and the beneficial effect of moderate alcohol consumption on myocardial infarction. N Engl J Med. 2001; 344(8): 549-55.

Hippe M, Vestbo J, Hein HO, Borch-Johnsen K, Jensen G, Sorensen TI. Familial predisposition and susceptibility to the effect of other risk factors for myocardial infarction. J Epidemiol Community Health. 1999; 53(5): 269-76.

Ikehara S, Iso H, Toyoshima H, Date C, Yamamoto A, Kikuchi S, Kondo T, Watanabe Y, Koizumi A, Wada Y, Inaba Y, Tamakoshi A. Alcohol consumption and mortality from stroke and coronary heart disease among Japanese men and women: the Japan collaborative cohort study. Stroke. 2008; 39(11): 2936-42.

Ikehara S, Iso H, Yamagishi K, Yamamoto S, Inoue M, Tsugane S. Alcohol consumption, social support, and risk of stroke and coronary heart disease among Japanese men: the JPHC Study. Alcohol Clin Exp Res. 2009; 33(6): 1025-32.

Ilic M, Grujicic Sipetic S, Ristic B, Ilic I. Myocardial infarction and alcohol consumption: A case-control study. PLoS One. 2018; 13(6): e0198129.

Ilomäki J, Hajat A, Kauhanen J, Kurl S, Kaufman JS, Tuomainen TP, Korhonen MJ. Relationship between alcohol consumption and myocardial infarction among ageing men using a marginal structural model. Eur J Public Health. 2012; 22(6): 825-30.

Iso H, Kitamura A, Shimamoto T, Sankai T, Naito Y, Sato S, Kiyama M, Iida M, Komachi Y. Alcohol intake and the risk of cardiovascular disease in middle-aged Japanese men. Stroke. 1995; 26(5): 767-73.

Jackson R, Scragg R, Beaglehole R. Alcohol consumption and risk of coronary heart disease. BMJ. 1991; 303(6796): 211-6.

Jakovljevic B, Stojanov V, Paunovic K, Belojevic G, Milic N. Alcohol consumption and mortality in Serbia: twenty-year follow-up study. Croat Med J. 2004; 45(6): 764-8.

Kabagambe EK, Baylin A, Ruiz-Narvaez E, Rimm EB, Campos H. Alcohol intake, drinking patterns, and risk of nonfatal acute myocardial infarction in Costa Rica. Am J Clin Nutr. 2005; 82(6): 1336-45.

Kalandidi A, Tzonou A, Toupadaki N, Lan SJ, Koutis C, Drogari P, Notara V, Hsieh CC, Toutouzas P, Trichopoulos D. A case-control study of coronary heart disease in Athens, Greece. Int J Epidemiol. 1992; 21(6): 1074-80.

Kaufman DW, Rosenberg L, Helmrich SP, Shapiro S. Alcoholic beverages and myocardial infarction in young men. Am J Epidemiol. 1985; 121(4): 548-54.

Kawanishi M, Nakamoto A, Konemori G, Horiuchi I, Kajiyama G. Coronary sclerosis risk factors in males with special reference to lipoproteins and apoproteins: establishing an index. Hiroshima J Med Sci. 1990; 39(3): 61-4.

Keil U, Chambless LE, Döring A, Filipiak B, Stieber J. The relation of alcohol intake to coronary heart disease and all-cause mortality in a beer-drinking population. Epidemiology. 1997; 8(2): 150-6.

Key TJ, Appleby PN, Spencer EA, Travis RC, Roddam AW, Allen NE. Mortality in British vegetarians: results from the European Prospective Investigation into Cancer and Nutrition (EPIC-Oxford). Am J Clin Nutr. 2009; 89(5): 1613-1619.

Kitamura A, Iso H, Sankai T, Naito Y, Sato S, Kiyama M, Okamura T, Nakagawa Y, Iida M, Shimamoto T, Komachi Y. Alcohol intake and premature coronary heart disease in urban Japanese men. Am J Epidemiol. 1998;

147(1): 59-65.

Kivela SL, Nissinen A, Ketola A, Punsar S, Puska P, Karvonen M. Alcohol consumption and mortality in aging or aged Finnish men. J Clin Epidemiol. 1989; 42(1): 61-8.

Klatsky AL, Chartier D, Udaltsova N, Gronningen S, Brar S, Friedman GD, Lundstrom RJ. Alcohol drinking and risk of hospitalization for heart failure with and without associated coronary artery disease. Am J Cardiol. 2005; 96(3): 346-51.

Kono S, Handa K, Kawano T, Hiroki T, Ishihara Y, Arakawa K. Alcohol intake and nonfatal acute myocardial infarction in Japan. Am J Cardiol. 1991; 68(10): 1011-4.

Kono S, Ikeda M, Tokudome S, Nishizumi M, Kuratsune M. Alcohol and mortality: a cohort study of male Japanese physicians. Int J Epidemiol. 1986; 15(4): 527-32.

Larsson SC, Wallin A, Wolk A. Contrasting association between alcohol consumption and risk of myocardial infarction and heart failure: Two prospective cohorts. Int J Cardiol. 2017; 231: 207-210.

Lazarus NB, Kaplan GA, Cohen RD, Leu DJ. Change in alcohol consumption and risk of death from all causes and from ischaemic heart disease. BMJ. 1991; 303(6802): 553-6.

Lee DH, Folsom AR, Jacobs DR. Dietary iron intake and Type 2 diabetes incidence in postmenopausal women: the Iowa Women's Health Study. Diabetologia. 2004; 47(2): 185-94.

Liao Y, McGee DL, Cao G, Cooper RS. Alcohol intake and mortality: findings from the National Health Interview Surveys (1988 and 1990). Am J Epidemiol. 2000; 151(7): 651-9.

Licaj I, Sandin S, Skeie G, Adami HO, Roswall N, Weiderpass E. Alcohol consumption over time and mortality in the Swedish Women's Lifestyle and Health cohort. BMJ Open. 2016; 6(11): e012862.

Lindschou Hansen J, Tolstrup JS, Jensen MK, Gronbaek M, Tjonneland A, Schmidt EB, Overvad K. Alcohol intake and risk of acute coronary syndrome and mortality in men and women with and without hypertension. Eur J Epidemiol. 2011; 26(6): 439-47.

Makela P, Paljarvi T, Poikolainen K. Heavy and nonheavy drinking occasions, all-cause and cardiovascular mortality and hospitalizations: a follow-up study in a population with a low consumption level. J Stud Alcohol. 2005; 66(6): 722-8.

Malyutina S, Bobak M, Kurilovitch S, Gafarov V, Simonova G, Nikitin Y, Marmot M. Relation between heavy and binge drinking and all-cause and cardiovascular mortality in Novosibirsk, Russia: a prospective cohort study. Lancet. 2002; 360(9344): 1448-54.

Maraldi C, Volpato S, Kritchevsky SB, Cesari M, Andresen E, Leeuwenburgh C, Harris TB, Newman AB, Kanaya A, Johnson KC, Rodondi N, Pahor M. Impact of inflammation on the relationship among alcohol consumption, mortality, and cardiac events: the health, aging, and body composition study. Arch Intern Med. 2006; 166(14): 1490-7.

Marques-Vidal P, Montaye M, Arveiler D, Evans A, Bingham A, Ruidavets JB, Amouyel P, Haas B, Yarnell J, Ducimetiere P, Ferrieres J. Alcohol consumption and cardiovascular disease: differential effects in France and Northern Ireland. The PRIME study. Eur J Cardiovasc Prev Rehabil. 2004; 11(4): 336-43.

Mehlig K, Strandhagen E, Svensson PA, Rosengren A, Torén K, Thelle DS, Lissner L. CETP TaqIB genotype modifies the association between alcohol and coronary heart disease: the INTERGENE case-control study. Alcohol. 2014; 48(7): 695-700.

Meisinger C, Döring A, Schneider A, Löwel H, KORA Study Group. Serum gamma-glutamyltransferase is a predictor of incident coronary events in apparently healthy men from the general population. Atherosclerosis. 2006; 189(2): 297-302.

Merry AH, Boer JM, Schouten LJ, Feskens EJ, Verschuren WM, Gorgels AP, van den Brandt PA. Smoking, alcohol consumption, physical activity, and family history and the risks of acute myocardial infarction and unstable angina pectoris: a prospective cohort study. BMC Cardiovasc Disord. 2011; 11: 13.

Miller GJ, Beckles GL, Maude GH, Carson DC. Alcohol consumption: protection against coronary heart disease

and risks to health. Int J Epidemiol. 1990; 19(4): 923-30.

Millwood IY, Walters RG, Mei XW, Guo Y, Yang L, Bian Z, Bennett DA, Chen Y, Dong C, Hu R, Zhou G, Yu B, Jia W, Parish S, Clarke R, Davey Smith G, Collins R, Holmes MV, Li L, Peto R, Chen Z, China Kadoorie Biobank Collaborative Group. Conventional and genetic evidence on alcohol and vascular disease aetiology: a prospective study of 500000 men and women in China. Lancet. 2019; 393(10183): 1831-1842.

Miyake Y. Risk factors for non-fatal acute myocardial infarction in middle-aged and older Japanese. Fukuoka Heart Study Group. Jpn Circ J. 2000; 64(2): 103-9.

Mukamal KJ, Chiuve SE, Rimm EB. Alcohol consumption and risk for coronary heart disease in men with healthy lifestyles. Arch Intern Med. 2006; 166(19): 2145-50.

Ng R, Sutradhar R, Yao Z, Wodchis WP, Rosella LC. Smoking, drinking, diet and physical activity-modifiable lifestyle risk factors and their associations with age to first chronic disease. Int J Epidemiol. 2019.

Oliveira A, Barros H, Azevedo A, Bastos J, Lopes C. Impact of risk factors for non-fatal acute myocardial infarction. Eur J Epidemiol. 2009; 24(8): 425-32.

Oliveira A, Barros H, Lopes C. Gender heterogeneity in the association between lifestyles and non-fatal acute myocardial infarction. Public Health Nutr . 2009; 12(10): 1799-806.

Onat A, Hergenc G, Kucukdurmaz Z, Ugur M, Kaya Z, Can G, Yuksel H. Moderate and heavy alcohol consumption among Turks: long-term impact on mortality and cardiometabolic risk. Turk Kardiyol Dern Ars. 2009; 37(2): 83-90.

Pedersen JØ, Heitmann BL, Schnohr P, Grønbaek M. The combined influence of leisure-time physical activity and weekly alcohol intake on fatal ischaemic heart disease and all-cause mortality. Eur Heart J. 2008; 29(2): 204-12.

Rehm JT, Bondy SJ, Sempos CT, Vuong CV. Alcohol consumption and coronary heart disease morbidity and mortality. Am J Epidemiol. 1997; 146(6): 495-501.

Renaud SC, Gueguen R, Schenker J, d'Houtaud A. Alcohol and mortality in middle-aged men from eastern France. Epidemiology. 1998; 9(2): 184-8.

Ricci C, Wood A, Muller D, Gunter MJ, Agudo A, Boeing H, van der Schouw YT, Warnakula S, Saieva C, Spijkerman A, Sluijs I, Tjønneland A, Kyrø C, Weiderpass E, Kühn T, Kaaks R, Sánchez MJ, Panico S, Agnoli C, Palli D, Tumino R, Engström G, Melander O, Bonnet F, Boer JMA, Key TJ, Travis RC, Overvad K, Verschuren WMM, Quirós JR, Trichopoulou A, Papatesta EM, Peppa E, Iribas CM, Gavrila D, Forslund AS, Jansson JH, Matullo G, Arriola L, Freisling H, Lassale C, Tzoulaki I, Sharp SJ, Forouhi NG, Langenberg C, Saracci R, Sweeting M, Brennan P, Butterworth AS, Riboli E, Wareham NJ, Danesh J, Ferrari P. Alcohol intake in relation to non-fatal and fatal coronary heart disease and stroke: EPIC-CVD case-cohort study. BMJ. 2018; 361: k934.

Rimm EB, Giovannucci EL, Willett WC, Colditz GA, Ascherio A, Rosner B, Stampfer MJ. Prospective study of alcohol consumption and risk of coronary disease in men. Lancet. 1991; 338(8765): 464-8.

Roerecke M, Greenfield TK, Kerr WC, Bondy S, Cohen J, Rehm J. Heavy drinking occasions in relation to ischaemic heart disease mortality-- an 11-22 year follow-up of the 1984 and 1995 US National Alcohol Surveys. Int J Epidemiol. 2011; 40(5): 1401-10.

Romelsjo A, Allebeck P, Andreasson S, Leifman A. Alcohol, mortality and cardiovascular events in a 35 year follow-up of a nationwide representative cohort of 50,000 Swedish conscripts up to age 55. Alcohol Alcohol. 2012; 47(3): 322-7.

Romelsjo A, Branting M, Hallqvist J, Alfredsson L, Hammar N, Leifman A, Ahlbom A. Abstention, alcohol use and risk of myocardial infarction in men and women taking account of social support and working conditions: the SHEEP case-control study. Addiction. 2003; 98(10): 1453-62.

Rostron B. Alcohol consumption and mortality risks in the USA. Alcohol Alcohol. 2012; 47(3): 334-9.

Ruidavets JB, Ducimetière P, Evans A, Montaye M, Haas B, Bingham A, Yarnell J, Amouyel P, Arveiler D, Kee F, Bongard V, Ferrières J. Patterns of alcohol consumption and ischaemic heart disease in culturally divergent countries: the Prospective Epidemiological Study of Myocardial Infarction (PRIME). BMJ. 2010; 341: c6077.

Schooling CM, Sun W, Ho SY, Chan WM, Tham MK, Ho KS, Leung GM, Lam TH. Moderate alcohol use and

mortality from ischaemic heart disease: a prospective study in older Chinese people. PLoS One. 2008; 3(6): e2370.

Schroder H, Masabeu A, Marti MJ, Cols M, Lisbona JM, Romagosa C, Carion T, Vilert E, Marrugat J. Myocardial infarction and alcohol consumption: a population-based case-control study. Nutr Metab Cardiovasc Dis. 2007; 17(8): 609-15.

Scragg R, Stewart A, Jackson R, Beaglehole R. Alcohol and exercise in myocardial infarction and sudden coronary death in men and women. Am J Epidemiol. 1987; 126(1): 77-85.

Sempos CT, Rehm J, Crespo C, Trevisan M. No Protective Effect of Alcohol Consumption on Coronary Heart Disease (CHD) in African Americans: Average Volume of Drinking over the Life Course and CHD Morbidity and Mortality in a U.S. National Cohort. Contemp Drug Probs. 2002; 29(4): 805-20.

Shaper AG, Wannamethee G, Walker M. Alcohol and coronary heart disease: a perspective from the British Regional Heart Study. Int J Epidemiol. 1994; 23(3): 482-94.

Simons LA, McCallum J, Friedlander Y, Simons J. Alcohol intake and survival in the elderly: a 77 month follow-up in the Dubbo study. Aust N Z J Med. 1996; 26(5): 662-70.

Skov-Ettrup LS, Eliasen M, Ekholm O, Grønbæk M, Tolstrup JS. Binge drinking, drinking frequency, and risk of ischaemic heart disease: a population-based cohort study. Scand J Public Health. 2011; 39(8): 880-7.

Snow WM, Murray R, Ekuma O, Tyas SL, Barnes GE. Alcohol use and cardiovascular health outcomes: a comparison across age and gender in the Winnipeg Health and Drinking Survey Cohort. Age Ageing. 2009; 38(2): 206-12.

Song RJ, Nguyen XT, Quaden R, Ho YL, Justice AC, Gagnon DR, Cho K, O'Donnell CJ, Concato J, Gaziano JM, Djoussé L, VA Million Veteran Program. Alcohol Consumption and Risk of Coronary Artery Disease (from the Million Veteran Program). Am J Cardiol. 2018; 121(10): 1162-1168.

Streppel MT, Ocke MC, Boshuizen HC, Kok FJ, Kromhout D. Long-term wine consumption is related to cardiovascular mortality and life expectancy independently of moderate alcohol intake: the Zutphen Study. J Epidemiol Community Health. 2009; 63(7): 534-40.

Suhonen O, Aromaa A, Reunanen A, Knekt P. Alcohol consumption and sudden coronary death in middle-aged Finnish men. Acta Med Scand. 1987; 221(4): 335-41.

Tavani A, Bertuzzi M, Gallus S, Negri E, La Vecchia C. Risk factors for non-fatal acute myocardial infarction in Italian women. Prev Med. 2004; 39(1): 128-34.

Tavani A, Spertini L, Bosetti C, Parpinel M, Gnagnarella P, Bravi F, Peterson J, Dwyer J, Lagiou P, Negri E, La Vecchia C. Intake of specific flavonoids and risk of acute myocardial infarction in Italy. Public Health Nutr. 2006; 9(3): 369-74.

Thun MJ, Peto R, Lopez AD, Monaco JH, Henley SJ, Heath CW, Doll R. Alcohol consumption and mortality among middle-aged and elderly U.S. adults. N Engl J Med. 1997; 337(24): 1705-14.

Tolstrup J, Jensen MK, Tjonneland A, Overvad K, Mukamal KJ, Gronbaek M. Prospective study of alcohol drinking patterns and coronary heart disease in women and men. BMJ. 2006; 332(7552): 1244-8.

Wannamethee G, Shaper AG. Alcohol and sudden cardiac death. Br Heart J. 1992; 68(5): 443-8.

Wannamethee SG, Shaper AG. Type of alcoholic drink and risk of major coronary heart disease events and all-cause mortality. 1999; 89(5): 685-90.

Wilkins K. Moderate alcohol consumption and heart disease. Health Rep. 2002; 14(1): 9-24.

Yang L, Zhou M, Sherliker P, Cai Y, Peto R, Wang L, Millwood I, Smith M, Hu Y, Yang G, Chen Z. Alcohol drinking and overall and cause-specific mortality in China: nationally representative prospective study of 220,000 men with 15 years of follow-up. Int J Epidemiol. 2012; 41(4): 1101-13.

Yi SW, Yoo SH, Sull JW, Ohrr H. Association between Alcohol Drinking and Cardiovascular Disease Mortality and All-cause Mortality: Kangwha Cohort Study. J Prev Med Public Health. 2004; 37(2): 120-6.

Younis J, Cooper JA, Miller GJ, Humphries SE, Talmud PJ. Genetic variation in alcohol dehydrogenase 1C and the

beneficial effect of alcohol intake on coronary heart disease risk in the Second Northwick Park Heart Study. Atherosclerosis. 2005; 180(2): 225-32.

Yusuf S, Joseph P, Rangarajan S, Islam S, Mente A, Hystad P, Brauer M, Kutty VR, Gupta R, Wielgosz A, AlHabib KF, Dans A, Lopez-Jaramillo P, Avezum A, Lanas F, Oguz A, Kruger IM, Diaz R, Yusoff K, Mony P, Chifamba J, Yeates K, Kelishadi R, Yusufali A, Khatib R, Rahman O, Zatonska K, Iqbal R, Wei L, Bo H, Rosengren A, Kaur M, Mohan V, Lear SA, Teo KK, Leong D, O'Donnell M, McKee M, Dagenais G. Modifiable risk factors, cardiovascular disease, and mortality in 155722 individuals from 21 high-income, middle-income, and low-income countries (PURE): a prospective cohort study. Lancet. 2019.

Zhang Y, Yu Y, Yuan Y, Yu K, Yang H, Li X, Min X, Zhang C, He M, Zhang X, Wu T. Association of drinking pattern with risk of coronary heart disease incidence in the middle-aged and older Chinese men: Results from the Dongfeng-Tongji cohort. PLoS One. 2017; 12(5): e0178070.

Zhou X, Li C, Xu W, Hong X, Chen J. Relation of alcohol consumption to angiographically proved coronary artery disease in chinese men. Am J Cardiol. 2010; 106(8): 1101-3.

Tuberculosis

Search String: (alcoholic beverage[MeSH Terms] OR drinking behavior[MeSH Terms] OR "alcohol"[Title/Abstract])
AND
(tuberculosis[MeSH Terms] OR tuberculosis[Title/Abstract] OR TB[Title/Abstract] OR "Mycobacterium tuberculosis"[Title/Abstract])
AND
(Risk[MeSH Terms] OR Odds Ratio[MeSH Terms] OR "risk"[Title/Abstract] OR "odds ratio"[Title/Abstract] OR "cross-product ratio"[Title/Abstract] OR "hazards ratio"[Title/Abstract] OR "hazard ratio"[Title/Abstract])
AND ("1970/01/01"[PDat] : "2019/12/31"[PDat])
AND (English[LA])
NOT (animals[MeSH Terms] NOT Humans[MeSH Terms])

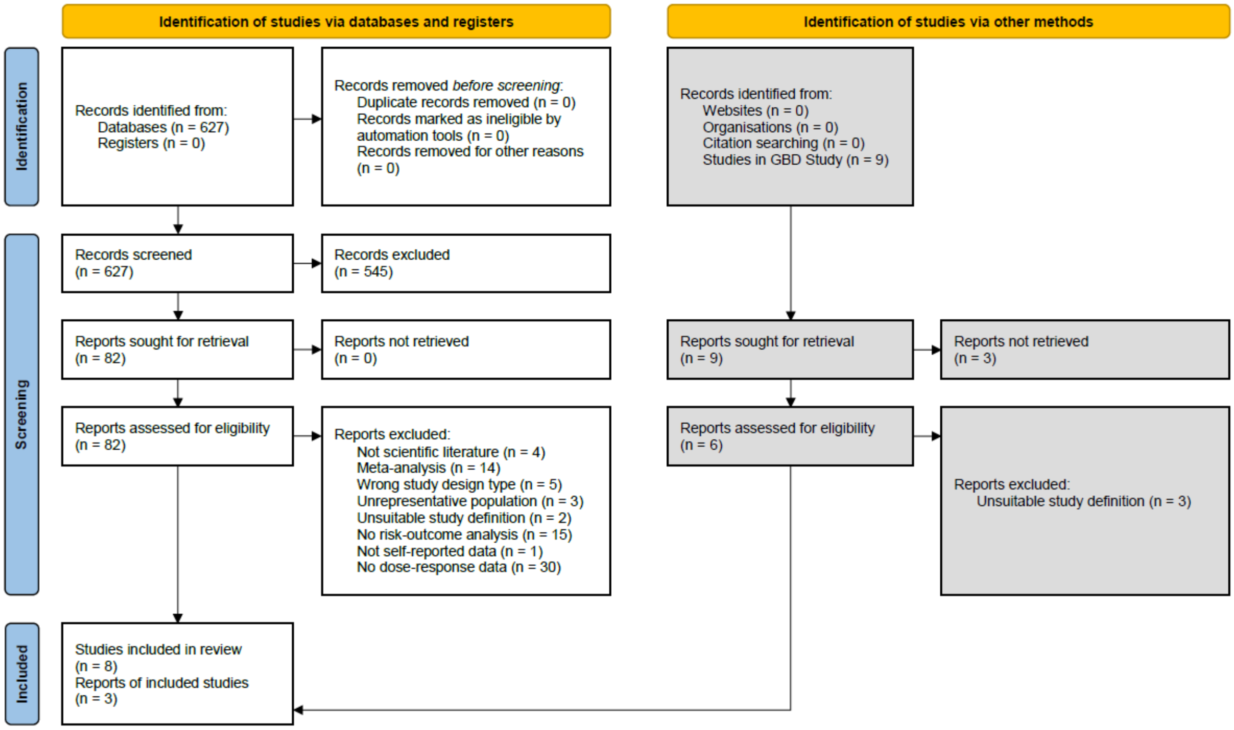

From: Page MJ, McKenzie JE, Bossuyt PM, Boutron I, Hoffmann TC, Mulrow CD, et al. The PRISMA 2020 statement: an updated guideline for reporting systematic reviews. BMJ 2021;372:n71. doi: 10.1136/bmj.n71. For more information, visit: http://www.prisma-statement.org/

References for Tuberculosis

Balakrishnan S, Rakesh PS, Viswanathan K, Nelson V, Simon S, Nair SA, Mathew ME, Sunilkumar M. Higher risk of developing active TB among adult diabetics exposed to TB during childhood: A study from Kerala, India. J Fam Med Prim Care. 2019; 8(2): 695-700.

Brown KE, Campbell AH. Tobacco, Alcohol and Tuberculosis. Br J Dis Chest. 1961; 55(3): 150-8.

Buskin SE, Gale JL, Weiss NS, Nolan CM. Tuberculosis risk factors in adults in King County, Washington, 1988 through 1990. Am J Public Health. 1994; 84(11): 1750-6.

Crampin AC, Glynn JR, Floyd S, Malema SS, Mwinuka VK, Ngwira BM, Mwaungulu FD, Warndorff DK, Fine PE. Tuberculosis and gender: exploring the patterns in a case control study in Malawi. Int J Tuberc Lung Dis. 2004; 8(2): 194-203.

Jee SH, Golub JE, Jo J, Park IS, Ohrr H, Samet JM. Smoking and risk of tuberculosis incidence, mortality, and recurrence in South Korean men and women. Am J Epidemiol. 2009; 170(12): 1478-85.

Leal ML, Maciel ELN, Cade NV. Factors associated with tuberculosis in a population of diabetics: A case-control study. CiÍnc Sa˙de Colet. 2018; 24(9): 3247-3256.

Lewis JG, Chamberlain DA. Alcohol consumption and smoking habits in male patients with Pulmonary Tuberculosis. Br J Prev Soc Med. 1963; 17: 149-52.

Rosenman KD, Hall N. Occupational risk factors for developing tuberculosis. Am J Ind Med. 1996; 30(2): 148-54.

Soh AZ, Chee CBE, Wang YT, Yuan JM, Koh WP. Alcohol drinking and cigarette smoking in relation to risk of active tuberculosis: prospective cohort study. BMJ Open Respir Res. 2017; 4(1): e000247.

Souza de Lima D, Morishi Ogusku M, Porto Dos Santos M, de Melo Silva CM, Alves de Almeida V, Assumpção Antunes I, Boechat AL, Ramasawmy R, Sadahiro A. Alleles of HLA-DRB1-04 Associated with Pulmonary Tuberculosis in Amazon Brazilian Population. PLoS One. 2016; 11(2): e0147543.

Tekkel M, Rahu M, Loit HM, Baburin A. Risk factors for pulmonary tuberculosis in Estonia. Int J Tuberc Lung Dis. 2002; 6(10): 887-94.

Modeled Relative Risk Curves

The green shaded area depicts the 95% uncertainty interval of the estimates. The solid green line depicts the mean estimates. Horizontal grey lines depict study data points, the darker the points the smaller the uncertainty of the data. Horizontal lines span the exposure range reported for the data point. The horizontal dashed line highlights a relative risk of one, compared to a non-drinker reference.

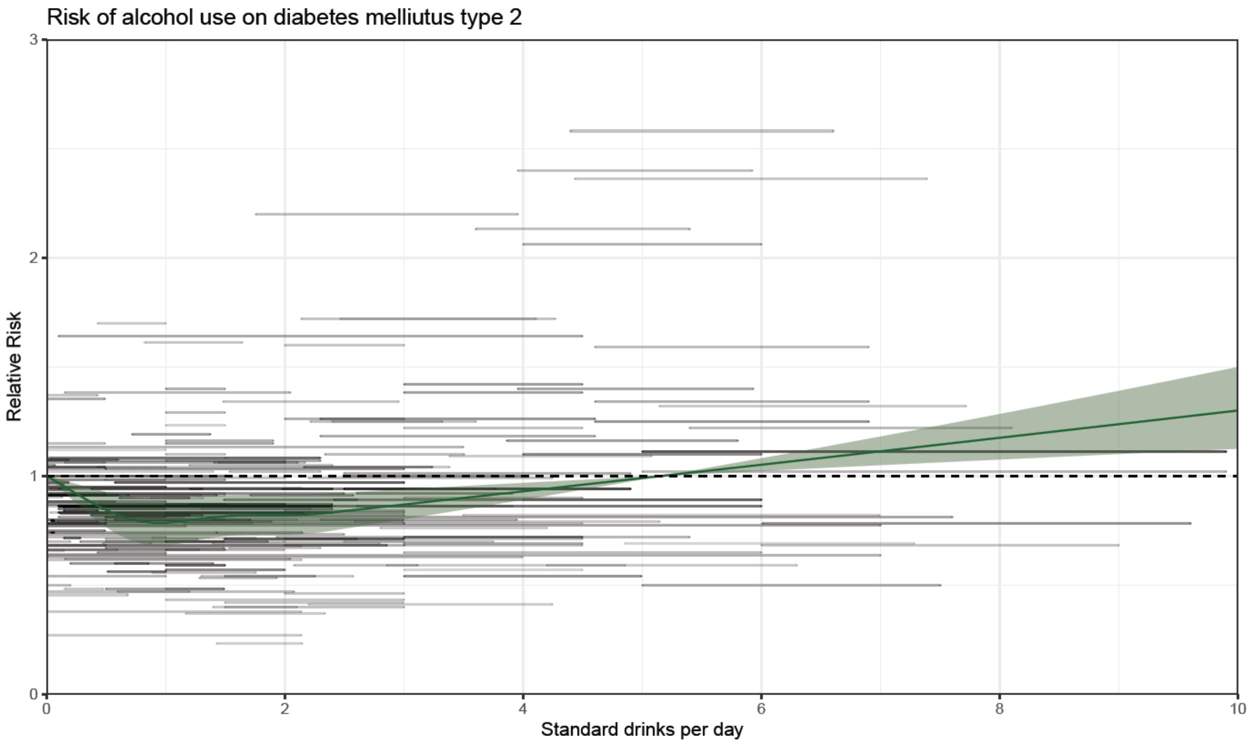

Risk of alcohol use on intracerebral hemorrhage

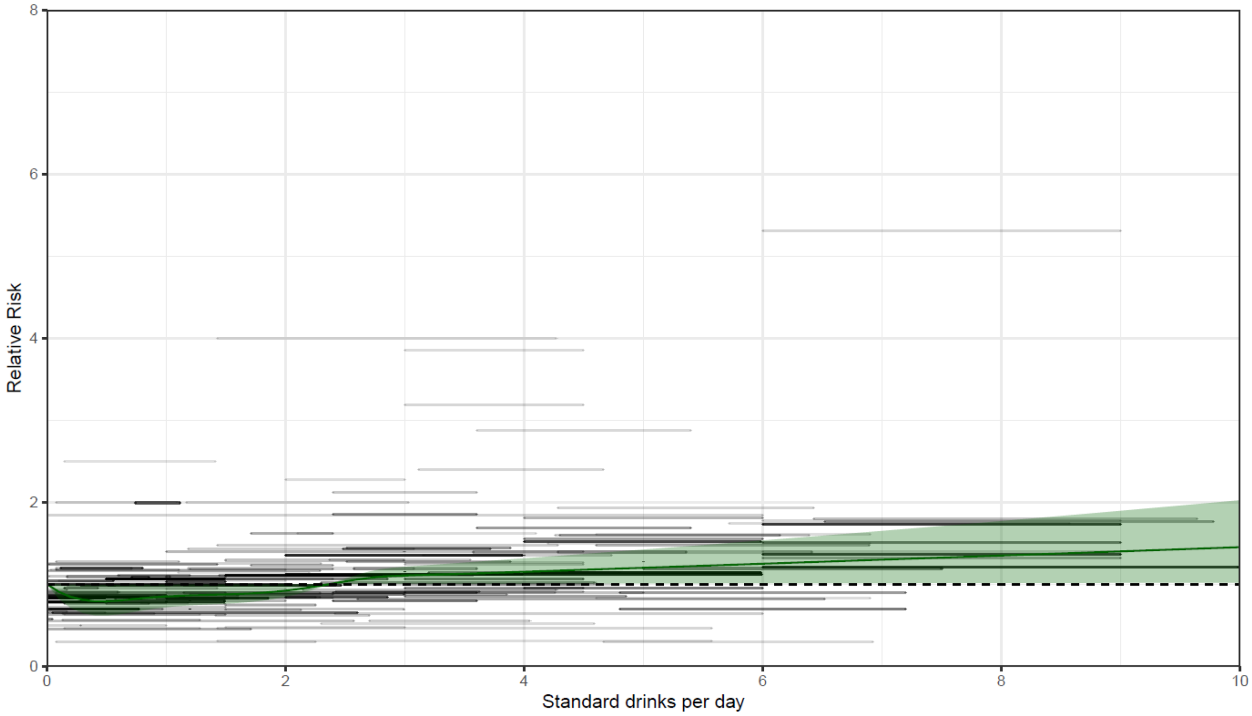

Risk of alcohol use on ischemic stroke

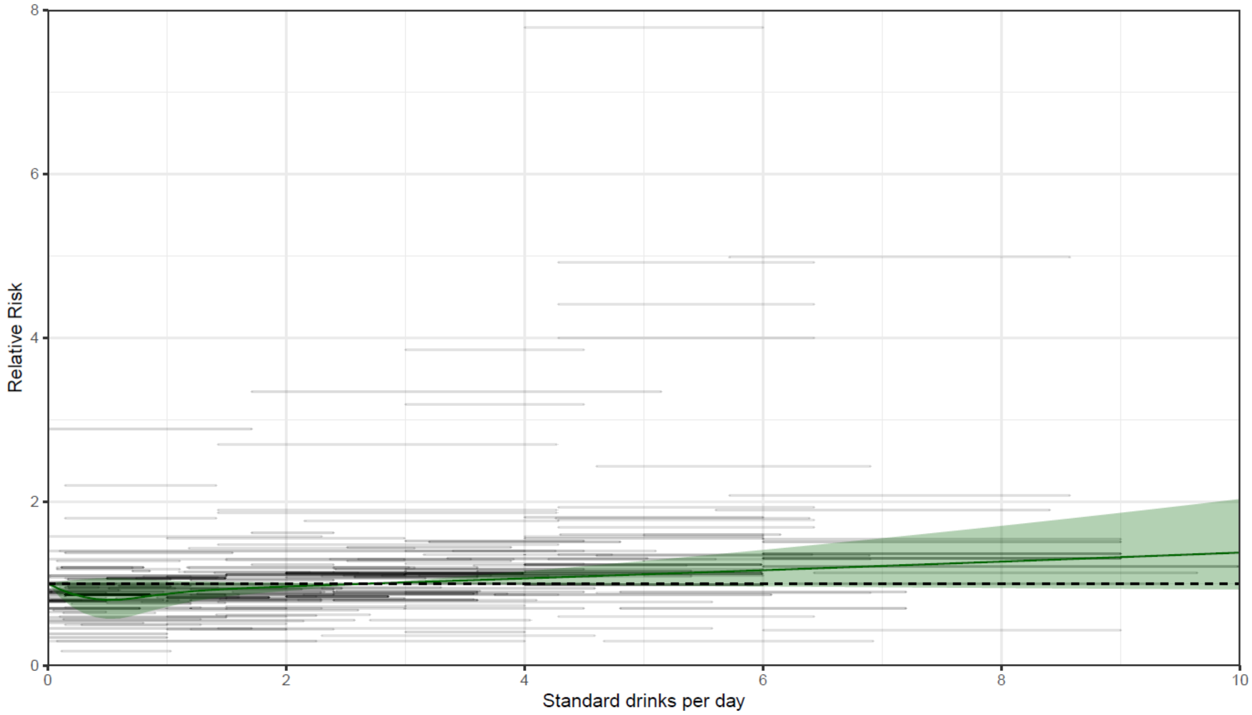

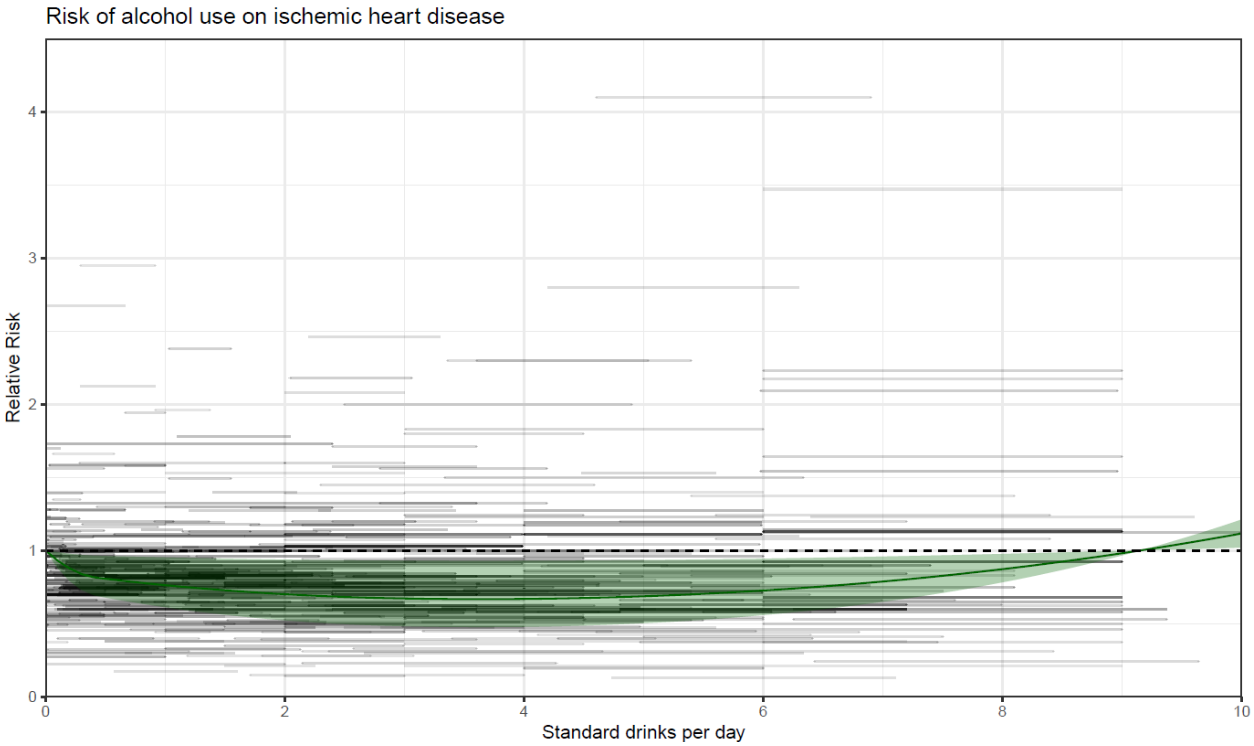
Risk of alcohol use on ischemic heart disease
Relative Risk
Standard drinks per day
0
1
2
3
4
0
2
4
6
8
10

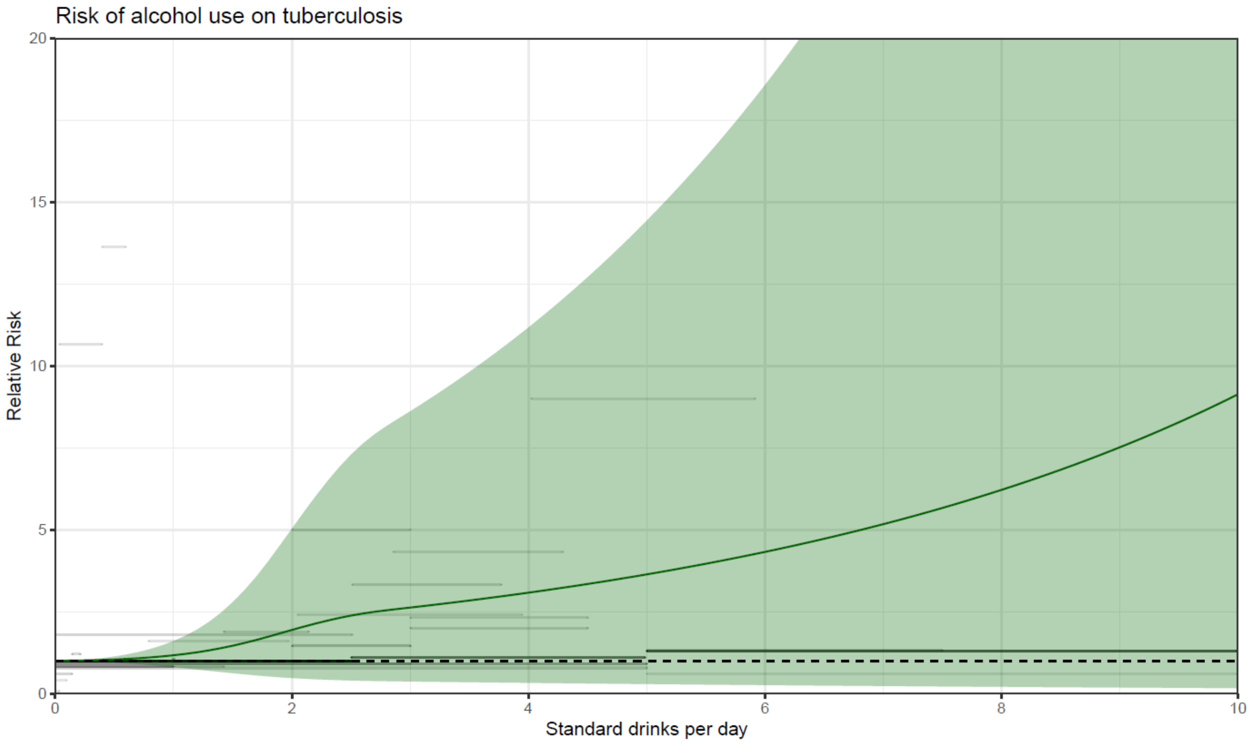
Risk of alcohol use on tuberculosis
Relative Risk
Standard drinks per day
0
5
10
15
20
0
2
4
6
8
10

GATHER Checklist

This study complies with the Guidelines for Accurate and Transparent Health Estimates Reporting (GATHER) recommendations.

Item #	Checklist item	Reported on page #
**Objectives and funding**		
1	Define the indicator(s), populations (including age, sex, and geographic entities), and time period(s) for which estimates were made.	Main 8-12; Methods Appendix 3-9
2	List the funding sources for the work.	3
**Data Inputs**		
For all data inputs from multiple sources that are synthesized as part of the study:		
3	Describe how the data were identified and how the data were accessed.	Methods Appendix 10, 13-17
4	Specify the inclusion and exclusion criteria. Identify all ad-hoc exclusions.	Methods Appendix 10
5	Provide information on all included data sources and their main characteristics. For each data source used, report reference information or contact name/institution, population represented, data collection method, year(s) of data collection, sex and age range, diagnostic criteria or measurement method, and sample size, as relevant.	http://ghdx.healthdata.org/gbd-2019/data-input-sources
6	Identify and describe any categories of input data that have potentially important biases (e.g., based on characteristics listed in item 5).	20
For data inputs that contribute to the analysis but were not synthesized as part of the study:		
7	Describe and give sources for any other data inputs.	8-12
For all data inputs:		
8	Provide all data inputs in a file format from which data can be efficiently extracted (e.g., a spreadsheet rather than a PDF), including all relevant meta-data listed in item 5. For any data inputs that cannot be shared because of ethical or legal reasons, such as third-party ownership, provide a contact name or the name of the institution that retains the right to the data.	http://ghdx.healthdata.org/gbd-2019/data-input-sources
**Data analysis**		
9	Provide a conceptual overview of the data analysis method. A diagram may be helpful.	8; http://ghdx.healthdata.org/gbd-2019/code/risk-1

**10**	Provide a detailed description of all steps of the analysis, including mathematical formulae. This description should cover, as relevant, data cleaning, data pre-processing, data adjustments and weighting of data sources, and mathematical or statistical model(s).	Main 8-12; Methods Appendix 12
**11**	Describe how candidate models were evaluated and how the final model(s) were selected.	Main 10, methods appendix 10, referenced manuscript with additional details published separately
**12**	Provide the results of an evaluation of model performance, if done, as well as the results of any relevant sensitivity analysis.	17
**13**	Describe methods for calculating uncertainty of the estimates. State which sources of uncertainty were, and were not, accounted for in the uncertainty analysis.	11
**14**	State how analytic or statistical source code used to generate estimates can be accessed.	http://ghdx.healthdata.org/gbd-2019/code/risk-1
**Results and Discussion**		
**15**	Provide published estimates in a file format from which data can be efficiently extracted.	Will be made public through http://ghdx.healthdata.org upon publication
**16**	Report a quantitative measure of the uncertainty of the estimates (e.g. uncertainty intervals).	Main 12-17; Supplemental Results;
**17**	Interpret results in light of existing evidence. If updating a previous set of estimates, describe the reasons for changes in estimates.	4-6; 19-20
**18**	Discuss limitations of the estimates. Include a discussion of any modelling assumptions or data limitations that affect interpretation of the estimates.	20

Contributions

Managing the overall research enterprise

Emmanuela Gakidou, Erin C Mullany, Christopher J L Murray.

Writing the first draft of the manuscript

Dana Bryazka, Max G Griswold, Marissa Reitsma, Emmanuela Gakidou.

Primary responsibility for applying analytical methods to produce estimates

Dana Bryazka, Max G Griswold, Marissa Reitsma.

Primary responsibility for seeking, cataloguing, extracting, or cleaning data; designing or coding figures and tables

Jason Anderson, Dana Bryazka, Rachel Feldman.

Providing data or critical feedback on data sources

Kalkidan Hassen Abate, Cristiana Abbafati, Amir Abdoli, E S Abhilash, Oladimeji M Adebayo, Victor Adekanmbi, Sangeet Adhikari, Qorinah Estiningtyas Sakilah Adnani, Saira Afzal, Manik Aggarwal, Bright Opoku Ahinkorah, Sajjad Ahmad, Tauseef Ahmad, Sepideh Ahmadi, Ali Ahmadi, Haroon Ahmed, Tarik Ahmed Rashid, Chisom Joyqueenet Akunna, Hanadi Al Hamad, Kefyalew Addis Alene, atiyeh alizadeh, Nelson Alvis-Guzman, Jason A Anderson, Catalina Liliana Andrei, Tudorel Andrei, Jalal Arabloo, Mulusew A Asemahagn, Seyyed Shamsadin Athari, Alok Atreya, Floriane Ausloos, Marcel Ausloos, Melkalem Mamuye Azanaw, Ahmed Y Azzam, Ashish D Badiye, Nasser Bagheri, Sara Bagherieh, Awraris Hailu Balchut/Bilchut, Till Winfried Bärnighausen, Fabio Barra, Amadou Barrow, Pritish Baskaran, Derrick A Bennett, Akshaya Srikanth Bhagavathula, Neeraj Bhala, Sonu Bhaskar, Bagas Suryo Bintoro, Belay Boda Abule Bodicha, Archith Boloor, Dejana Braithwaite, Dana Bryazka, Joao Mauricio Castaldelli-Maia, Promit Ananyo Chakraborty, Vijay Kumar Chattu, Dinh-Toi Chu, Chuchu Churko, Massimo Cirillo, Rafael M Claro, Richard G Cowden, Michael H Criqui, Natália Cruz-Martins, Xiaochen Dai, Giovanni Damiani, Rakhi Dandona, Lalit Dandona, Andreas K Demetriades, Meseret Derbew Molla, Rupak Desai, Diana Dias da Silva, Milad Dodangeh, Fariba Dorostkar, Haneil Larson Dsouza, Bruce B Duncan, Michael Ekholuenetale, Frank J Elgar, Aman Yesuf Endries, Sharareh Eskandarieh, Adeniyi Francis Fagbamigbe, Ildar Ravisovich Fakhradiyev, Fatemeh Farahmand, Carla Sofia e Sá Farinha, Andre Faro, Farshad Farzadfar, Ali Fatehizadeh, Valery L Feigin, Rachel Feldman, Simone Ferrero, Lorenzo Ferro Desideri, Joel Msafiri Francis, Richard Charles Franklin, Peter Andras Gaal, Mohamed M Gad, Emmanuela Gakidou, Fabio Galvano, Tushar Garg, Teferi Gebru Gebremeskel, Abera Getachew Obsa, Amir Ghaderi, Mansour Ghafourifard, Lilian A Ghandour, Ahmad Ghashghaee, Sherief Ghozy, Justyna Godos, Amit Goel, Salime Goharinezhad, Mahaveer Golechha, Pouya Goleij, Felix Greaves, Max G Griswold, Giuseppe Grosso, Sapna Gupta, Vivek Kumar Gupta, Veer Bala Gupta, Nima Hafezi-Nejad, Arvin Haj-Mirzaian, Brian J Hall, Rabih Halwani, Josep Maria Haro, Ahmed I Hasaballah, Claudiu Herteliu, Demisu Zenbaba Heyi, Kamal Hezam, Mbuzeleni Mbuzeleni Hlongwa, Mehdi hosseinzadeh, Segun Emmanuel Ibitoye, Sheikh Mohammed Shariful Islam, Hiroyasu Iso, Mihajlo Jakovljevic, Sathish Kumar Jayapal, Seyed Ali Jeddi, Jost B Jonas, Tamas Joo, Farahnaz Joukar, Jacek Jerzy Jozwiak, Mikk Jürisson, Himal Kandel, Girum Gebremeskel Kanno, Neeti Kapoor, Bekalu Getnet Kassa, Rimple Jeet Kaur, Gbenga A Kayode, Mohammad Keykhaei, Himanshu Khajuria, Rovshan Khalilov, Imteyaz A Khan, Moien AB Khan, Jihee Kim, Ann Kristin Skrindo Knudsen, Kewal Krishan, G Anil Kumar, Savita Lasrado, Yo Han Lee, Sang-woong Lee,

Stephen S Lim, Lee-Ling Lim, Stefan Lorkowski, Farzan Madadizadeh, Áurea M Madureira-Carvalho, Rashidul Alam Mahumud, Alaa Makki, Narayana Manjunatha, Borhan Mansouri, Mohammad Ali Mansournia, Jose Martinez-Raga, Jitendra Kumar Meena, Entezar Mehrabi Nasab, Ritesh G Menezes, Gert B M Mensink, Atte Meretoja, Tomislav Mestrovic, Bartosz Miazgowski, Ted R Miller, Erkin M Mirrakhimov, Babak Moazen, Abdollah Mohammadian-Hafshejani, Nagabhishek Moka, Ali H Mokdad, Sara Momtazmanesh, Yousef Moradi, Ebrahim Mostafavi, Sumaira Mubarik, Erin C Mullany, Efrén Murillo-Zamora, Mohsen Naghavi, Mukhammad David Naimzada, Biswa Prakash Nayak, Ruxandra Irina Negoi, Ionut Negoi, Samata Nepal, Sudan Prasad Prasad Neupane, Sandhya Neupane Kandel, Khan M Nuruzzaman, Chimezie Igwegbe Nzoputam, Kehinde O Obamiro, Felix Akpojene Ogbo, Ayodipupo Sikiru Oguntade, Emad Omer, Adrian Otoiu, Bilcha Oumer, Mahesh P A, Raffaele Palladino, Adrian Pana, Songhomitra Panda-Jonas, Anamika Pandey, Ashok Pandey, Shahina Pardhan, Fatemeh Pashazadeh Kan, Jay Patel, Siddhartha Pati, Shrikant Pawar, Marina Pinheiro, Vafa Rahimi-Movaghar, Azizur Rahman, Amir Masoud Rahmani, Chhabi Lal Ranabhat, Chythra R Rao, Salman Rawaf, Lal Rawal, Marissa B Reitsma, Andre M N Renzaho, Seyed Mohammad Riahi, Esperanza Romero-Rodríguez, Godfrey M Rwegerera, Basema Saddik, Reihaneh Sadeghian, Umar Saeed, Rajesh Sagar, Harihar Sahoo, Mohammad Ali Sahraian, KM Saif-Ur-Rahman, Abdallah M Samy, Milena M Santric-Milicevic, Brijesh Sathian, Ganesh Kumar Saya, Mehdi Sayyah, Maria Inês Schmidt, David C Schwebel, Abdul-Aziz Seidu, Allen Seylani, Feng Sha, Fariba Shahraki-Sanavi, Masood Ali Shaikh, Murad Ziyaudinovich Shakhmardanov, Sara Sheikhbahaei, Adithi Shetty, K M Shivakumar, Parnian Shobeiri, Roman Shrestha, Negussie Boti Sidemo, Jasvinder A Singh, Valentin Yurievich Skryabin, Anna Aleksandrovna Skryabina, Marco Solmi, Sergey Soshnikov, Miklós Szócska, Rafael Tabarés-Seisdedos, Takahiro Tabuchi, Ker-Kan Tan, Elvis Enowbeyang Tarkang, Nikhil Kenny Thomas, Marco Torrado, Mathilde Touvier, Marcos Roberto Tovani-Palone, Mai Thi Ngoc Tran, Alireza Vakilian, Mehdi Varmaghani, Shoban Babu Varthya, Tommi Juhani Vasankari, Theo Vos, Yasir Waheed, Mandaras Tariku Walde, Ronny Westerman, Abate Dargie Wubetu, Suowen Xu, Kazumasa Yamagishi, Gesila Endashaw E Yesera, Vahit Yiğit, Dong Keon Yon, Naohiro Yonemoto, Chuanhua Yu, Siddhesh Zadey, Iman Zare, Mikhail Sergeevich Zastrozhin, Yves Miel H Zuniga.

Developing methods or computational machinery

Qorinah Estiningtyas Sakilah Adnani, Saira Afzal, Araz Ramazan Ahmad, Ali Ahmadi, Tarik Ahmed Rashid, Zahra Aryan, Ahmed Y Azzam, Akshaya Srikanth Bhagavathula, Dana Bryazka, Rafael M Claro, Garland T Culbreth, Xiaochen Dai, Ali Fatehizadeh, Emmanuela Gakidou, Amir Ghaderi, Max G Griswold, Mehdi hosseinzadeh, Rovshan Khalilov, Sang-woong Lee, Ata Mahmoodpoor, Rashidul Alam Mahumud, Alaa Makki, Borhan Mansouri, Jose Martinez-Raga, Jitendra Kumar Meena, Teroj Abdulrahman Mohammed, Ali H Mokdad, Yousef Moradi, Mohsen Naghavi, Biswa Prakash Nayak, Emad Omer, Bilcha Oumer, Ashok Pandey, Charles D H Parry, Siddhartha Pati, Alireza Rafiei, Shayan Rahmani, Amir Masoud Rahmani, Chhabi Lal Ranabhat, Marissa B Reitsma, Seyed Mohammad Riahi, Gregory A Roth, Reihaneh Sadeghian, Umar Saeed, Abdallah M Samy, Ganesh Kumar Saya, Mehdi Sayyah, Rahim Ali Sheikhi, Negussie Boti Sidemo, Valentin Yurievich Skryabin, Reed J D Sorensen, Mehdi Varmaghani, Theo Vos, Ronny Westerman, Gesila Endashaw E Yesera, Mikhail Sergeevich Zastrozhin.

Providing critical feedback on methods or results

Cristiana Abbafati, Mohammad Abdollahi, Abu Yousuf Md Abdullah, E S Abhilash, Juan Manuel Acuna, Oladimeji M Adebayo, Victor Adekanmbi, Sangeet Adhikari, Kishor Adhikari, Qorinah Estiningtyas Sakilah Adnani, Saira Afzal, Wubetu Yimam Agegnehu, Manik Aggarwal, Bright Opoku Ahinkorah, Sajjad Ahmad, Tauseef Ahmad, Araz Ramazan Ahmad, Ali Ahmadi, Haroon Ahmed, Tarik Ahmed Rashid, Chisom

Joyqueenet Akunna, Hanadi Al Hamad, Md Zakiul Alam, Dejene Tsegaye Alem, Kefyalew Addis Alene, Yousef Alimohamadi, atiyeh alizadeh, Kasim Allel, Saba Alvand, Nelson Alvis-Guzman, Edward Kwabena Ameyaw, Sohrab Amiri, Robert Ancuceanu, Jason A Anderson, Catalina Liliana Andrei, Tudorel Andrei, Jalal Arabloo, Muhammad Arshad, Anton A Artamonov, Zahra Aryan, Mulusew A Asemahagn, Thomas Astell-Burt, Seyyed Shamsadin Athari, Desta Debalkie Atnafu, Prince Atorkey, Alok Atreya, Floriane Ausloos, Marcel Ausloos, Getinet Ayano, Martin Amogre ayanore Ayanore, Olatunde O Ayinde, Jose L Ayuso-Mateos, Sina Azadnajafabad, Melkalem Mamuye Azanaw, Mohammadreza Azangou-Khyavy, Amirhossein Azari Jafari, Ahmed Y Azzam, Ashish D Badiye, Nasser Bagheri, Sara Bagherieh, Mohan Bairwa, Ravleen Kaur Bakshi, Awraris Hailu Balchut/Bilchut, Till Winfried Bärnighausen, Fabio Barra, Amadou Barrow, Pritish Baskaran, Derrick A Bennett, Akshaya Srikanth Bhagavathula, Pankaj Bhardwaj, Nikha Bhardwaj, Sonu Bhaskar, Krittika Bhattacharyya, Vijayalakshmi S Bhojaraja, Bagas Suryo Bintoro, Elena A Elena Blokhina, Belay Boda Abule Bodicha, Archith Boloor, Dejana Braithwaite, Andre R Brunoni, Dana Bryazka, Zahid A Butt, Chao Cao, Rosario Cárdenas, Andre F Carvalho, Joao Mauricio Castaldelli-Maia, Giulio Castelpietra, Luis F S Castro-de-Araujo, Promit Ananyo Chakraborty, Jaykaran Charan, Vijay Kumar Chattu, Akhilanand Chaurasia, Nicolas Cherbuin, Dinh-Toi Chu, Sheng-Chia Chung, Chuchu Churko, Liliana G Ciobanu, Massimo Cirillo, Rafael M Claro, Simona Costanzo, Michael H Criqui, Natália Cruz-Martins, Berihun Assefa Dachew, Omid Dadras, Xiaochen Dai, Giovanni Damiani, Rakhi Dandona, Lalit Dandona, Beniam Darge Daniel, Anna Danielewicz, Jiregna Darega Gela, Kairat Davletov, Jacyra Azevedo Paiva de Araujo, Antonio Reis de Sá-Junior, Sisay Abebe Debela, Azizallah Dehghan, Andreas K Demetriades, Meseret Derbew Molla, Rupak Desai, Abebaw Alemayehu Desta, Diana Dias da Silva, Daniel Diaz, Lankamo Ena Digesa, Mengistie Diress, Milad Dodangeh, Deepa Dongarwar, Fariba Dorostkar, Haneil Larson Dsouza, Bereket Duko, Kristina Edvardsson, Michael Ekholuenetale, Frank J Elgar, Muhammed Elhadi, Mohamed A Elmonem, Aman Yesuf Endries, Sharareh Eskandarieh, Adeniyi Francis Fagbamigbe, Ildar Ravisovich Fakhradiyev, Fatemeh Farahmand, Carla Sofia e Sá Farinha, Andre Faro, Farshad Farzadfar, Ali Fatehizadeh, Rachel Feldman, Xiaoqi Feng, Zinabu Fentaw, Lorenzo Ferro Desideri, Irina Filip, Florian Fischer, Joel Msafiri Francis, Richard Charles Franklin, Peter Andras Gaal, Mohamed M Gad, Emmanuela Gakidou, Fabio Galvano, Balasankar Ganesan, Mesfin Gebrehiwot Damtew Gebrehiwot, Teferi Gebru Gebremeskel, Mathewos Alemu Gebremichael, Tadele Regasa Gemechu, Lemma Getacher, Motuma Erena Getachew, Abera Getachew Obsa, Amir Ghaderi, Mansour Ghafourifard, Alireza Ghajar, Lilian A Ghandour, Mohammad Ghasemi Nour, Ahmad Ghashghaee, Sherief Ghozy, Franklin N Glozah, Justyna Godos, Mahaveer Golechha, Mohamad Golitaleb, Felix Greaves, Max G Griswold, Michal Grivna, Giuseppe Grosso, Temesgen Worku Gudayu, Bhawna Gupta, Rajeev Gupta, Sapna Gupta, Vivek Kumar Gupta, Veer Bala Gupta, Nima Hafezi-Nejad, Arvin Haj-Mirzaian, Brian J Hall, Rabih Halwani, Tiilahun Beyene Handiso, Sanam Hariri, Josep Maria Haro, Ahmed I Hasaballah, Hossein Hassanian-Moghaddam, Simon I Hay, Khezar Hayat, Golnaz Heidari, Mohammad Heidari, Delia Hendrie, Claudiu Herteliu, Demisu Zenbaba Heyi, Kamal Hezam, Mbuzeleni Mbuzeleni Hlongwa, Ramesh Holla, Md Mahbub Hossain, Sahadat Hossain, Seyed Kianoosh Hosseini, Mehdi hosseinzadeh, Mihaela Hostiuc, Guoqing Hu, Salman Hussain, Segun Emmanuel Ibitoye, Irena M Ilic, Milena D Ilic, Mustapha Immurana, Lalu Muhammad Irham, Sheikh Mohammed Shariful Islam, M Mofizul Islam, Rakibul M Islam, Ramaiah Itumalla, Masao Iwagami, Roxana Jabbarinejad, Louis Jacob, Mihajlo Jakovljevic, Zahra Jamalpoor, Elham Jamshidi, Sathish Kumar Jayapal, Umesh Umesh Jayarajah, Ranil Jayawardena, Rime Jebai, Seyed Ali Jeddi, Alelign Tasew Jema, Ravi Prakash Jha, Har Ashish Jindal, Jost B Jonas, Tamas Joo, Nitin Joseph, Farahnaz Joukar, Jacek Jerzy Jozwiak, Mikk Jürisson, Ali Kabir, Robel Hussen Kabthymer, Bhushan Dattatray Kamble, Himal Kandel, Neeti Kapoor, Ibraheem M Karaye,

Salah Eddin Karimi, Bekalu Getnet Kassa, Rimple Jeet Kaur, Gbenga A Kayode, Himanshu Khajuria, Rovshan Khalilov, Imteyaz A Khan, Moien AB Khan, Jihee Kim, Min Seo Kim, Ruth W Kimokoti, Mika Kivimäki, Vitalii Klymchuk, Ann Kristin Skrindo Knudsen, Ali-Asghar Kolahi, Ai Koyanagi, Kewal Krishan, Yuvaraj Krishnamoorthy, G Anil Kumar, Narinder Kumar, Nithin Kumar, Savita Lasrado, Jerrald Lau, Wei-Chen Lee, Sang-woong Lee, Stephen S Lim, Lee-Ling Lim, Stany W Lobo, Stefan Lorkowski, Giancarlo Lucchetti, Farzan Madadizadeh, Áurea M Madureira-Carvalho, Ata Mahmoodpoor, Rashidul Alam Mahumud, Alaa Makki, Mohammad-Reza Malekpour, Narayana Manjunatha, Borhan Mansouri, Mohammad Ali Mansournia, Jose Martinez-Raga, Francisco A Martinez-Villa, Richard Matzopoulos, Pallab K Maulik, Mahsa Mayeli, John J McGrath, Jitendra Kumar Meena, Entezar Mehrabi Nasab, Ritesh G Menezes, Gert B M Mensink, Alexios-Fotios A Mentis, Atte Meretoja, Bedasa Taye Merga, Tomislav Mestrovic, Junmei Miao Jonasson, Ana Carolina Micheletti Gomide Nogueira de Sá, Ted R Miller, GK Mini, Andreea Mirica, Seyyedmohammadsadeq Mirmoeeni, Erkin M Mirrakhimov, Sanjeev Misra, Babak Moazen, Marcello Moccia, Yousef Mohammad, Esmaeil Mohammadi, Abdollah Mohammadian-Hafshejani, Teroj Abdulrahman Mohammed, Nagabhishek Moka, Ali H Mokdad, Sara Momtazmanesh, Yousef Moradi, Ebrahim Mostafavi, Sumaira Mubarik, Erin C Mullany, Beemnet Tekabe Mulugeta, Efrén Murillo-Zamora, Christopher J L Murray, Julius C Mwita, Mohsen Naghavi, Mukhammad David Naimzada, Vinay Nangia, Biswa Prakash Nayak, Ruxandra Irina Negoi, Ionut Negoi, Seyed Aria Nejadghaderi, Samata Nepal, Sudan Prasad Prasad Neupane, Sandhya Neupane Kandel, Ali Nowroozi, Khan M Nuruzzaman, Chimezie Igwegbe Nzoputam, Kehinde O Obamiro, Felix Akpojene Ogbo, Ayodipupo Sikiru Oguntade, Hassan Okati-Aliabad, Gláucia Maria Moraes Oliveira, Ahmed Omar Bali, Emad Omer, Doris V Ortega-Altamirano, Adrian Otoiu, Stanislav S Otstavnov, Bilcha Oumer, Mahesh P A, Raffaele Palladino, Adrian Pana, Songhomitra Panda-Jonas, Anamika Pandey, Ashok Pandey, Shahina Pardhan, Tarang Parekh, Eun-Kee Park, Jay Patel, Siddhartha Pati, George C Patton, Uttam Paudel, Shrikant Pawar, Amy E Peden, Ionela-Roxana Petcu, Michael R Phillips, Pranil Man Singh Pradhan, Akila Prashant, Jianchao Quan, Amir Radfar, Pankaja Raghav Raghav, Vafa Rahimi-Movaghar, Azizur Rahman, Md Mosfequr Rahman, Mosiur Rahman, Shayan Rahmani, Amir Masoud Rahmani, Chhabi Lal Ranabhat, Priyanga Ranasinghe, Chythra R Rao, Drona Prakash Rasali, Mohammad-Mahdi Rashidi, David Laith Rawaf, Salman Rawaf, Lal Rawal, Marissa B Reitsma, Negar Rezaei, Mohsen Rezaeian, Seyed Mohammad Riahi, Esperanza Romero-Rodríguez, Godfrey M Rwegerera, Basema Saddik, Erfan Sadeghi, Reihaneh Sadeghian, Umar Saeed, Farhad Saeedi, Rajesh Sagar, Harihar Sahoo, KM Saif-Ur-Rahman, Sarvenaz Salahi, Hamideh Salimzadeh, Abdallah M Samy, Francesco Sanmarchi, Milena M Santric-Milicevic, Yaser Sarikhani, Brijesh Sathian, Ganesh Kumar Saya, Mehdi Sayyah, Aletta Elisabeth Schutte, Michaël Schwarzinger, David C Schwebel, Abdul-Aziz Seidu, Nachimuthu Senthil Kumar, SeyedAhmad SeyedAlinaghi, Allen Seylani, Feng Sha, Sarvenaz Shahin, Masood Ali Shaikh, Elaheh Shaker, Murad Ziyaudinovich Shakhmardanov, Mehran Shams-Beyranvand, Sara Sheikhbahaei, Rahim Ali Sheikhi, Jeevan K Shetty, Damtew Solomon Solomon Shiferaw, Mika Shigematsu, Rahman Shiri, K M Shivakumar, Velizar Shivarov, Parnian Shobeiri, Roman Shrestha, Negussie Boti Sidemo, Inga Dora Sigfusdottir, Natacha Torres da Silva, Diego Augusto Santos Silva, Jasvinder A Singh, Surjit Singh, Anna Aleksandrovna Skryabina, Marco Solmi, YONATAN SOLOMON, Suhang Song, Yimeng Song, Reed J D Sorensen, Sergey Soshnikov, Ireneous N Soyiri, Sonu Hangma Subba, Miklós Szócska, Rafael Tabarés-Seisdedos, Takahiro Tabuchi, Ker-Kan Tan, Minale Tareke, Elvis Enowbeyang Tarkang, Worku Animaw Temesgen, Gebremaryam Temesgen, Mohamad-Hani Temsah, Kavumpurathu Raman Thankappan, Rekha Thapar, Nikhil Kenny Thomas, Chalachew Chalachew Tiruneh, Jovana Todorovic, Mathilde Touvier, Marcos Roberto Tovani-Palone, Mai Thi Ngoc Tran, Sergi Trias-Llimós, Rohollah Valizadeh, Mehdi Varmaghani,

Shoban Babu Varthya, Theo Vos, Birhanu Wagaye, Yasir Waheed, Mandaras Tariku Walde, Cong Wang, Yanzhong Wang, Yuan-Pang Wang, Ronny Westerman, Nuwan Darshana Wickramasinghe, Abate Dargie Wubetu, Suowen Xu, Gesila Endashaw E Yesera, Arzu Yigit, Vahit Yiğit, Ayenew Engida Ayenew Engida Yimaw, Dong Keon Yon, Naohiro Yonemoto, Chuanhua Yu, Siddhesh Zadey, Mazyar Zahir, Mikhail Sergeevich Zastrozhin, Zhi-Jiang Zhang, Chenwen Zhong, Mohammad Zmaili, Yves Miel H Zuniga.

Drafting the work or revising it critically for important intellectual content

Kalkidan Hassen Abate, Cristiana Abbafati, Zeinab Abbasi-Kangevari, Mohsen Abbasi-Kangevari, Eman Abu-Gharbieh, Juan Manuel Acuna, Giovanni Addolorato, Oladimeji M Adebayo, Victor Adekanmbi, Qorinah Estiningtyas Sakilah Adnani, Saira Afzal, Manik Aggarwal, Bright Opoku Ahinkorah, Araz Ramazan Ahmad, Sepideh Ahmadi, Ali Ahmadi, Md Zakiul Alam, Dejene Tsegaye Alem, Kefyalew Addis Alene, Kasim Allel, Jordi Alonso, Saba Alvand, Nelson Alvis-Guzman, Firehiwot Amare, Sohrab Amiri, Robert Ancuceanu, Jason A Anderson, Catalina Liliana Andrei, Tudorel Andrei, Jalal Arabloo, Muhammad Arshad, Zahra Aryan, Malke Asaad, Mulusew A Asemahagn, Prince Atorkey, Alok Atreya, Floriane Ausloos, Marcel Ausloos, Getinet Ayano, Martin Amogre ayanore Ayanore, Olatunde O Ayinde, Sina Azadnajafabad, Melkalem Mamuye Azanaw, Mohammadreza Azangou-Khyavy, Amirhossein Azari Jafari, Ahmed Y Azzam, Ashish D Badiye, Sara Bagherieh, Mohan Bairwa, Shankar M Bakkannavar, Awraris Hailu Balchut/Bilchut, Till Winfried Bärnighausen, Amadou Barrow, Pritish Baskaran, Luis Belo, Isabela M Bensenor, Akshaya Srikanth Bhagavathula, Neeraj Bhala, Ashish Bhalla, Sonu Bhaskar, Krittika Bhattacharyya, Vijayalakshmi S Bhojaraja, Elena A Elena Blokhina, Belay Boda Abule Bodicha, Cristina Bosetti, Dejana Braithwaite, Hermann Brenner, Nikolay Ivanovich Briko, Dana Bryazka, Chao Cao, Yin Cao, Márcia Carvalho, Andre F Carvalho, Joao Mauricio Castaldelli-Maia, Giulio Castelpietra, Maria Sofia Cattaruzza, Vijay Kumar Chattu, Akhilanand Chaurasia, Nicolas Cherbuin, Dinh-Toi Chu, Nandita Chudal, Chuchu Churko, Liliana G Ciobanu, Richard G Cowden, Michael H Criqui, Natália Cruz-Martins, Giovanni Damiani, Beniam Darge Daniel, Anna Danielewicz, Jacyra Azevedo Paiva de Araujo, Andreas K Demetriades, Meseret Derbew Molla, Rupak Desai, Diana Dias da Silva, Daniel Diaz, Milad Dodangeh, Deepa Dongarwar, Haneil Larson Dsouza, Bruce B Duncan, Frank J Elgar, Muhammed Elhadi, Mohamed A Elmonem, Aman Yesuf Endries, Sharareh Eskandarieh, Azin Etemadimanesh, Adeniyi Francis Fagbamigbe, Andre Faro, Ali Fatehizadeh, Nelsensius Klau Fauk, Valery L Feigin, Rachel Feldman, Zinabu Fentaw, Simone Ferrero, Irina Filip, Florian Fischer, Joel Msafiri Francis, Peter Andras Gaal, Mohamed M Gad, Emmanuela Gakidou, Silvano Gallus, Balasankar Ganesan, Tushar Garg, Lemma Getacher, Motuma Erena Getachew, Abera Getachew Obsa, Asmare Getie, Amir Ghaderi, Mansour Ghafourifard, Alireza Ghajar, Seyyed-Hadi Ghamari, Lilian A Ghandour, Mohammad Ghasemi Nour, Ahmad Ghashghaee, Sherief Ghozy, Franklin N Glozah, Ekaterina Vladimirovna Glushkova, Justyna Godos, Mahaveer Golechha, Mohamad Golitaleb, Felix Greaves, Max G Griswold, Michal Grivna, Giuseppe Grosso, Temesgen Worku Gudayu, Rajeev Gupta, Sapna Gupta, Vivek Kumar Gupta, Veer Bala Gupta, Nima Hafezi-Nejad, Arvin Haj-Mirzaian, Brian J Hall, Rabih Halwani, Tiilahun Beyene Handiso, Graeme J Hankey, Josep Maria Haro, Ahmed I Hasaballah, Simon I Hay, Khezar Hayat, Golnaz Heidari, Mohammad Heidari, Claudiu Herteliu, Demisu Zenbaba Heyi, Kamal Hezam, Ramesh Holla, Md Mahbub Hossain, Sahadat Hossain, Seyed Kianoosh Hosseini, Sorin Hostiuc, Junjie Huang, Salman Hussain, Segun Emmanuel Ibitoye, Irena M Ilic, Milena D Ilic, Mustapha Immurana, Lalu Muhammad Irham, Sheikh Mohammed Shariful Islam, Rakibul M Islam, Ramaiah Itumalla, Louis Jacob, Mihajlo Jakovljevic, Zahra Jamalpoor, Sathish Kumar Jayapal, Umesh Umesh Jayarajah, Rime Jebai, Alelign Tasew Jema, Ravi Prakash Jha, Har Ashish Jindal, Jost B Jonas, Tamas Joo, Nitin Joseph, Jacek Jerzy Jozwiak, Mikk Jürisson, Ali Kabir, Robel Hussen Kabthymer, Himal Kandel, Neeti Kapoor, Bekalu Getnet Kassa, Rimple Jeet Kaur,

Gbenga A Kayode, Himanshu Khajuria, Rovshan Khalilov, Imteyaz A Khan, Moien AB Khan, Jihee Kim, Min Seo Kim, Hanna Kim, Mika Kivimäki, Ann Kristin Skrindo Knudsen, Ali-Asghar Kolahi, Vladimir Andreevich Korshunov, Ai Koyanagi, Kewal Krishan, Yuvaraj Krishnamoorthy, Narinder Kumar, Ben Lacey, Tea Lallukka, Savita Lasrado, Jerrald Lau, Lee-Ling Lim, Platon D Lopukhov, Stefan Lorkowski, Giancarlo Lucchetti, Áurea M Madureira-Carvalho, Soleiman Mahjoub, Ata Mahmoodpoor, Rashidul Alam Mahumud, Mohammad-Reza Malekpour, Narayana Manjunatha, Borhan Mansouri, Jose Martinez-Raga, Francisco A Martinez-Villa, Richard Matzopoulos, Pallab K Maulik, Mahsa Mayeli, John J McGrath, Jitendra Kumar Meena, Entezar Mehrabi Nasab, Ritesh G Menezes, Alexios-Fotios A Mentis, Atte Meretoja, Tomislav Mestrovic, Ana Carolina Micheletti Gomide Nogueira de Sá, Ted R Miller, Antonio Mirijello, Seyyedmohammadsadeq Mirmoeeni, Babak Moazen, Maryam Mobarakabadi, Marcello Moccia, Yousef Mohammad, Esmaeil Mohammadi, Abdollah Mohammadian-Hafshejani, Teroj Abdulrahman Mohammed, Nagabhishek Moka, Ali H Mokdad, Sara Momtazmanesh, Yousef Moradi, Ebrahim Mostafavi, Erin C Mullany, Efrén Murillo-Zamora, Christopher J L Murray, Julius C Mwita, Mohsen Naghavi, Mukhammad David Naimzada, Biswa Prakash Nayak, Ruxandra Irina Negoi, Ionut Negoi, Seyed Aria Nejadghaderi, Sudan Prasad Prasad Neupane, Sandhya Neupane Kandel, Yeshambel T Nigatu, Ali Nowroozi, Khan M Nuruzzaman, Chimezie Igwegbe Nzoputam, Kehinde O Obamiro, Felix Akpojene Ogbo, Babayemi Oluwaseun Olakunde, Gláucia Maria Moraes Oliveira, Doris V Ortega-Altamirano, Adrian Otoiu, Stanislav S Otstavnov, Bilcha Oumer, Mahesh P A, Alicia Padron-Monedero, Raffaele Palladino, Adrian Pana, Songhomitra Panda-Jonas, Ashok Pandey, Shahina Pardhan, Jay Patel, George C Patton, Uttam Paudel, Shrikant Pawar, Amy E Peden, Ionela-Roxana Petcu, Michael R Phillips, Evgenii Plotnikov, Pranil Man Singh Pradhan, Akila Prashant, Amir Radfar, Alireza Rafiei, Pankaja Raghav Raghav, Vafa Rahimi-Movaghar, Azizur Rahman, Md Mosfequr Rahman, Shayan Rahmani, Chhabi Lal Ranabhat, Priyanga Ranasinghe, Chythra R Rao, Zubair Ahmed Ratan, David Laith Rawaf, Salman Rawaf, Lal Rawal, Marissa B Reitsma, Andre M N Renzaho, Saeid Rezaei, Seyed Mohammad Riahi, Esperanza Romero-Rodríguez, Gregory A Roth, Godfrey M Rwegerera, Basema Saddik, Reihaneh Sadeghian, Umar Saeed, Farhad Saeedi, Rajesh Sagar, Amirhossein Sahebkar, Harihar Sahoo, Mohammad Ali Sahraian, Hamideh Salimzadeh, Abdallah M Samy, Francesco Sanmarchi, Milena M Santric-Milicevic, Yaser Sarikhani, Ganesh Kumar Saya, Mehdi Sayyah, Maria Inês Schmidt, Aletta Elisabeth Schutte, David C Schwebel, Nachimuthu Senthil Kumar, Allen Seylani, Shayan Shahrokhi, Elaheh Shaker, Murad Ziyaudinovich Shakhmardanov, Mehran Shams-Beyranvand, Sara Sheikhbahaei, Jeevan K Shetty, Mika Shigematsu, Reza Shirkoohi, K M Shivakumar, Velizar Shivarov, Parnian Shobeiri, Roman Shrestha, Negussie Boti Sidemo, Inga Dora Sigfusdottir, Natacha Torres da Silva, Diego Augusto Santos Silva, Jasvinder A Singh, Valentin Yurievich Skryabin, Anna Aleksandrovna Skryabina, David A Sleet, Marco Solmi, Yonatan Solomon, Suhang Song, Sergey Soshnikov, Ireneous N Soyiri, Dan J Stein, Miklós Szócska, Takahiro Tabuchi, Majid Taheri, Ker-Kan Tan, Minale Tareke, Elvis Enowbeyang Tarkang, Gebremaryam Temesgen, Mohamad-Hani Temsah, Marco Torrado, Mathilde Touvier, Marcos Roberto Tovani-Palone, Mai Thi Ngoc Tran, Sergi Trias-Llimós, Jaya Prasad Tripathy, Alireza Vakilian, Mehdi Varmaghani, Shoban Babu Varthya, Tommi Juhani Vasankari, Birhanu Wagaye, Mandaras Tariku Walde, Yanzhong Wang, Yuan-Pang Wang, Ronny Westerman, Nuwan Darshana Wickramasinghe, Abate Dargie Wubetu, Kazumasa Yamagishi, Lin Yang, Gesila Endashaw E Yesera, Arzu Yigit, Vahit Yiğit, Ayenew Engida Ayenew Engida Yimaw, Dong Keon Yon, Naohiro Yonemoto, Mazyar Zahir, Iman Zare, Mikhail Sergeevich Zastrozhin, Anasthasia Zastrozhina, Zhi-Jiang Zhang, Chenwen Zhong, Mohammad Zmaili.

Managing the estimation or publications process

Emmanuela Gakidou, Simon I Hay, Ali Mokdad, Erin Mullany, Christopher J L Murray, Mohsen Naghavi.
