# Supplementary material for: Population-level risks of alcohol consumption by amount, geography, age, sex, and year: a systematic analysis for the Global Burden of Disease Study 2020
Source: Lancet. 2022 Jul 16;400(10347):185–235. doi: 10.1016/S0140-6736(22)00847-9 (PMC9289789; doi:10.1016/S0140-6736(22)00847-9)
Supplement: Supplementary appendix 2 [file mmc2.pdf]

THE LANCET

Supplementary appendix 2

This appendix formed part of the original submission and has been peer reviewed. We post it as supplied by the authors.

This online publication has been corrected. The corrected version first appeared at thelancet.com on July 19, 2022

Supplement to: GBD 2020 Alcohol Collaborators. Population-level risks of alcohol consumption by amount, geography, age, sex, and year: a systematic analysis for the Global Burden of Disease Study 2020. *Lancet* 2022; **400:** 185–235.

Supplementary Results

Risks of alcohol consumption by geography, age, sex, and year: a systematic analysis from the Global Burden of Disease Study 2020

GBD 2020 Alcohol Use Collaborators

Tables and Figures

Table S1: Theoretical minimum risk exposure level (TMREL) and non-drinker equivalence (NDE) in standard drinks per day, by region, age, and sex, for 1990, 2000, 2010, and 2020

Figure S1: Percent of the population consuming harmful amounts of alcohol in excess of the non-drinker equivalence (NDE), 2020

Figure S2: Theoretical minimum risk exposure level (TMREL) and non-drinker equivalence (NDE) in units of standard drinks, by region, age, and sex, 2020, for sensitivity scenario A

Figure S3: Theoretical minimum risk exposure level (TMREL) and non-drinker equivalence (NDE) in units of standard drinks, by region, age, and sex, 2020, for sensitivity scenario B

Figure S4: Theoretical minimum risk exposure level (TMREL) and non-drinker equivalence (NDE) in units of standard drinks, by region, age, and sex, 2020, for sensitivity scenario C

Figure S5: Theoretical minimum risk exposure level (TMREL) and non-drinker equivalence (NDE) in units of standard drinks, by region, age, and sex, 2020, for risk-deletion sensitivity scenario

Table S2: Theoretical minimum risk exposure level (TMREL) computed using GBD 2017 and GBD 2020 risk curves in standard drinks per day, by region, age, and sex, for 2020

Table S3: Demographic makeup of the population consuming harmful amounts of alcohol in excess of the non-drinker equivalence (NDE) by location, 2020

Supplementary Table 1

Region	Age Group	Year	Female TMREL	Female NDE	Male TMREL	Male NDE
Central Asia	15 to 19	1990	0·0735 (0–0·300)	0·126 (0–0·600)	0·00280 (0–0)	0·00480 (0–0)
Central Asia	20 to 24	1990	0·202 (0–0·400)	0·373 (0–1·00)	0·00720 (0–0·100)	0·0123 (0–0·200)
Central Asia	25 to 29	1990	0·264 (0–0·400)	0·527 (0–1·10)	0·0337 (0–0·300)	0·0570 (0–0·500)
Central Asia	30 to 34	1990	0·314 (0–0·500)	0·688 (0–1·30)	0·181 (0–0·400)	0·351 (0–1·00)
Central Asia	35 to 39	1990	0·358 (0·100–0·500)	0·862 (0·200–1·60)	0·330 (0–0·500)	0·844 (0–2·20)
Central Asia	40 to 44	1990	0·407 (0·298–0·600)	1·13 (0·498–2·00)	0·448 (0·200–1·00)	1·54 (0·400–3·60)
Central Asia	45 to 49	1990	0·461 (0·300–0·600)	1·48 (0·700–2·30)	0·527 (0·300–1·00)	1·98 (0·700–4·10)
Central Asia	50 to 54	1990	0·507 (0·400–0·700)	1·86 (1·00–3·00)	0·605 (0·400–1·70)	2·51 (0·900–4·80)
Central Asia	55 to 59	1990	0·569 (0·400–1·00)	2·37 (1·20–4·00)	0·656 (0·400–1·80)	2·91 (1·10–5·10)
Central Asia	60 to 64	1990	0·656 (0·500–1·50)	3·31 (1·50–5·30)	0·716 (0·500–1·90)	3·56 (1·40–5·70)
Central Asia	65 to 69	1990	0·737 (0·500–1·90)	4·04 (1·70–6·10)	0·810 (0·500–2·00)	4·20 (1·60–6·20)
Central Asia	70 to 74	1990	0·888 (0·500–2·00)	5·00 (2·00–6·80)	0·967 (0·500–2·20)	5·00 (1·80–6·80)
Central Asia	75 to 79	1990	1·11 (0·500–2·60)	5·73 (2·20–7·30)	1·20 (0·500–2·70)	5·73 (2·10–7·30)
Central Asia	80 plus	1990	1·54 (0·500–3·00)	6·59 (2·70–7·90)	1·68 (0·500–3·00)	6·64 (2·50–7·90)
Central Asia	15 to 19	2000	0·0728 (0–0·300)	0·123 (0–0·700)	0·00840 (0–0·100)	0·0128 (0–0·200)
Central Asia	20 to 24	2000	0·172 (0–0·400)	0·316 (0–1·10)	0·0301 (0–0·200)	0·0495 (0–0·400)
Central Asia	25 to 29	2000	0·284 (0–0·600)	0·571 (0–1·40)	0·113 (0–0·400)	0·199 (0–1·00)
Central Asia	30 to 34	2000	0·341 (0–0·600)	0·761 (0–2·00)	0·283 (0–1·00)	0·598 (0–2·40)
Central Asia	35 to 39	2000	0·380 (0·100–0·600)	0·917 (0·200–2·00)	0·392 (0–1·00)	0·987 (0–3·00)
Central Asia	40 to 44	2000	0·429 (0·300–0·700)	1·16 (0·500–2·10)	0·501 (0·200–1·90)	1·48 (0·400–3·60)
Central Asia	45 to 49	2000	0·490 (0·400–0·700)	1·57 (0·800–2·50)	0·587 (0·300–2·00)	2·00 (0·700–4·50)
Central Asia	50 to 54	2000	0·542 (0·400–1·00)	2·00 (1·00–3·20)	0·651 (0·400–1·90)	2·56 (0·998–5·10)
Central Asia	55 to 59	2000	0·611 (0·400–1·00)	2·49 (1·30–4·20)	0·706 (0·400–1·90)	3·01 (1·10–5·40)
Central Asia	60 to 64	2000	0·705 (0·500–1·60)	3·52 (1·70–5·50)	0·795 (0·500–2·00)	3·85 (1·50–6·00)
Central Asia	65 to 69	2000	0·830 (0·500–2·00)	4·37 (1·90–6·30)	0·926 (0·500–2·00)	4·59 (1·70–6·60)
Central Asia	70 to 74	2000	0·998 (0·500–2·20)	5·32 (2·20–7·00)	1·13 (0·500–2·50)	5·45 (2·00–7·10)
Central Asia	75 to 79	2000	1·20 (0·500–2·70)	5·94 (2·30–7·40)	1·35 (0·500–2·90)	6·01 (2·30–7·50)
Central Asia	80 plus	2000	1·61 (0·500–3·00)	6·72 (2·80–8·00)	1·81 (0·500–3·20)	6·83 (2·70–8·00)
Central Asia	15 to 19	2010	0·0777 (0–0·400)	0·138 (0–0·802)	0·0104 (0–0·200)	0·0168 (0–0·300)
Central Asia	20 to 24	2010	0·170 (0–0·400)	0·320 (0–1·00)	0·0250 (0–0·300)	0·0420 (0–0·500)
Central Asia	25 to 29	2010	0·270 (0–0·600)	0·546 (0–1·20)	0·0849 (0–0·400)	0·151 (0–0·900)
Central Asia	30 to 34	2010	0·321 (0–0·700)	0·683 (0–1·30)	0·229 (0–0·500)	0·464 (0–1·20)
Central Asia	35 to 39	2010	0·378 (0·100–0·700)	0·852 (0·100–1·60)	0·346 (0–1·00)	0·798 (0–1·90)
Central Asia	40 to 44	2010	0·442 (0·200–0·800)	1·11 (0·400–2·00)	0·450 (0·200–1·00)	1·21 (0·300–2·40)
Central Asia	45 to 49	2010	0·508 (0·398–1·00)	1·50 (0·700–2·30)	0·554 (0·300–1·00)	1·76 (0·700–3·30)
Central Asia	50 to 54	2010	0·568 (0·400–1·00)	1·90 (1·00–2·90)	0·650 (0·400–1·00)	2·40 (1·00–4·40)
Central Asia	55 to 59	2010	0·641 (0·497–1·00)	2·40 (1·30–3·90)	0·724 (0·400–1·80)	3·01 (1·20–5·20)
Central Asia	60 to 64	2010	0·712 (0·500–1·00)	3·29 (1·60–5·20)	0·789 (0·500–2·00)	3·75 (1·50–5·80)

Central Asia	65 to 69	2010	0·807 (0·500–2·00)	4·11 (1·90–6·10)	0·879 (0·500–2·00)	4·34 (1·80–6·30)
Central Asia	70 to 74	2010	0·968 (0·500–2·10)	5·13 (2·10–6·90)	1·07 (0·500–2·30)	5·28 (2·00–7·00)
Central Asia	75 to 79	2010	1·19 (0·500–2·70)	5·82 (2·30–7·40)	1·34 (0·500–2·90)	5·96 (2·20–7·50)
Central Asia	80 plus	2010	1·43 (0·500–3·00)	6·42 (2·50–7·80)	1·47 (0·500–3·00)	6·34 (2·40–7·70)
Central Asia	15 to 19	2020	0·112 (0–0·500)	0·201 (0–1·00)	0·0212 (0–0·200)	0·0333 (0–0·400)
Central Asia	20 to 24	2020	0·221 (0–0·500)	0·429 (0–1·10)	0·0416 (0–0·300)	0·0731 (0–0·600)
Central Asia	25 to 29	2020	0·293 (0–0·600)	0·590 (0–1·20)	0·0921 (0–0·400)	0·165 (0–1·00)
Central Asia	30 to 34	2020	0·344 (0–0·700)	0·721 (0–1·30)	0·224 (0–0·502)	0·451 (0–1·20)
Central Asia	35 to 39	2020	0·402 (0–0·900)	0·884 (0–1·50)	0·351 (0–1·00)	0·773 (0–1·60)
Central Asia	40 to 44	2020	0·471 (0·298–1·00)	1·15 (0·400–2·00)	0·466 (0·200–1·00)	1·18 (0·300–2·20)
Central Asia	45 to 49	2020	0·535 (0·400–1·00)	1·54 (0·800–2·40)	0·568 (0·300–1·00)	1·71 (0·698–3·10)
Central Asia	50 to 54	2020	0·595 (0·400–1·00)	1·94 (1·10–3·00)	0·672 (0·400–1·00)	2·32 (1·00–4·10)
Central Asia	55 to 59	2020	0·668 (0·500–1·00)	2·47 (1·30–3·90)	0·753 (0·400–1·70)	2·98 (1·30–5·00)
Central Asia	60 to 64	2020	0·751 (0·500–1·00)	3·40 (1·70–5·30)	0·823 (0·500–2·00)	3·72 (1·50–5·70)
Central Asia	65 to 69	2020	0·852 (0·500–2·00)	4·26 (2·00–6·20)	0·937 (0·500–2·00)	4·51 (1·90–6·40)
Central Asia	70 to 74	2020	0·921 (0·500–2·00)	5·01 (2·10–6·80)	1·02 (0·500–2·20)	5·10 (2·00–6·90)
Central Asia	75 to 79	2020	1·23 (0·500–2·60)	5·90 (2·30–7·40)	1·38 (0·500–2·90)	6·02 (2·30–7·50)
Central Asia	80 plus	2020	1·37 (0·500–3·00)	6·32 (2·50–7·70)	1·43 (0·500–3·00)	6·28 (2·40–7·70)
Central Europe	15 to 19	1990	0·00830 (0–0·100)	0·0132 (0–0·200)	0·00120 (0–0)	0·00210 (0–0)
Central Europe	20 to 24	1990	0·0407 (0–0·300)	0·0671 (0–0·500)	0·00460 (0–0·00250)	0·00810 (0–0·00250)
Central Europe	25 to 29	1990	0·134 (0–0·400)	0·239 (0–0·800)	0·0330 (0–0·300)	0·0590 (0–0·600)
Central Europe	30 to 34	1990	0·224 (0–0·400)	0·434 (0–1·00)	0·178 (0–0·400)	0·352 (0–1·10)
Central Europe	35 to 39	1990	0·294 (0–0·400)	0·620 (0–1·20)	0·324 (0–0·600)	0·798 (0–1·70)
Central Europe	40 to 44	1990	0·364 (0·100–0·600)	0·878 (0·200–1·40)	0·422 (0·200–1·00)	1·27 (0·300–2·60)
Central Europe	45 to 49	1990	0·423 (0·300–0·600)	1·16 (0·500–1·90)	0·488 (0·300–1·00)	1·66 (0·500–3·20)
Central Europe	50 to 54	1990	0·482 (0·300–0·800)	1·54 (0·800–2·40)	0·559 (0·300–1·00)	2·15 (0·798–4·00)
Central Europe	55 to 59	1990	0·558 (0·400–1·00)	2·11 (1·10–3·50)	0·636 (0·400–1·00)	2·71 (1·00–4·80)
Central Europe	60 to 64	1990	0·619 (0·500–1·00)	3·00 (1·40–4·80)	0·685 (0·500–1·70)	3·39 (1·30–5·40)
Central Europe	65 to 69	1990	0·666 (0·500–1·00)	3·82 (1·80–5·70)	0·743 (0·500–2·00)	4·05 (1·60–6·00)
Central Europe	70 to 74	1990	0·701 (0·500–1·90)	4·53 (2·00–6·30)	0·770 (0·500–2·00)	4·69 (1·90–6·50)
Central Europe	75 to 79	1990	0·757 (0·500–2·00)	5·04 (2·20–6·80)	0·852 (0·500–2·30)	5·25 (2·10–6·90)
Central Europe	80 plus	1990	0·911 (0·500–3·00)	5·71 (2·30–7·40)	1·06 (0·500–3·00)	5·96 (2·40–7·50)
Central Europe	15 to 19	2000	0·00730 (0–0·100)	0·0112 (0–0·200)	0·00170 (0–0)	0·00270 (0–0)
Central Europe	20 to 24	2000	0·0312 (0–0·300)	0·0511 (0–0·500)	0·00590 (0–0)	0·00980 (0–0)
Central Europe	25 to 29	2000	0·114 (0–0·300)	0·197 (0–0·700)	0·0225 (0–0·300)	0·0379 (0–0·500)
Central Europe	30 to 34	2000	0·211 (0–0·400)	0·399 (0–1·00)	0·126 (0–0·400)	0·231 (0–1·00)
Central Europe	35 to 39	2000	0·278 (0–0·402)	0·569 (0–1·10)	0·273 (0–0·500)	0·584 (0–1·30)
Central Europe	40 to 44	2000	0·350 (0·100–0·600)	0·799 (0·100–1·30)	0·376 (0–1·00)	0·935 (0–1·90)
Central Europe	45 to 49	2000	0·410 (0·200–0·700)	1·04 (0·400–1·70)	0·452 (0·200–1·00)	1·27 (0·400–2·30)
Central Europe	50 to 54	2000	0·472 (0·300–0·800)	1·35 (0·698–2·10)	0·515 (0·300–1·00)	1·69 (0·600–3·10)
Central Europe	55 to 59	2000	0·543 (0·400–1·00)	1·82 (0·998–2·90)	0·581 (0·400–1·00)	2·16 (0·900–3·80)

Central Europe	60 to 64	2000	0·604 (0·500–1·00)	2·62 (1·30–4·20)	0·632 (0·400–1·00)	2·85 (1·20–4·80)
Central Europe	65 to 69	2000	0·641 (0·500–1·00)	3·43 (1·70–5·30)	0·674 (0·500–1·50)	3·57 (1·50–5·50)
Central Europe	70 to 74	2000	0·682 (0·500–1·50)	4·26 (1·90–6·10)	0·718 (0·500–2·00)	4·34 (1·80–6·20)
Central Europe	75 to 79	2000	0·725 (0·500–2·00)	4·83 (2·10–6·60)	0·778 (0·500–2·00)	4·94 (2·00–6·60)
Central Europe	80 plus	2000	0·920 (0·500–3·00)	5·69 (2·30–7·40)	1·01 (0·500–3·00)	5·81 (2·30–7·40)
Central Europe	15 to 19	2010	0·00620 (0–0·100)	0·00980 (0–0·200)	0·00160 (0–0)	0·00300 (0–0)
Central Europe	20 to 24	2010	0·0294 (0–0·300)	0·0480 (0–0·500)	0·00740 (0–0)	0·0106 (0–0)
Central Europe	25 to 29	2010	0·0972 (0–0·300)	0·167 (0–0·700)	0·0198 (0–0·300)	0·0340 (0–0·402)
Central Europe	30 to 34	2010	0·180 (0–0·400)	0·327 (0–0·900)	0·104 (0–0·400)	0·190 (0–1·00)
Central Europe	35 to 39	2010	0·256 (0–0·500)	0·500 (0–1·00)	0·254 (0–0·600)	0·524 (0–1·20)
Central Europe	40 to 44	2010	0·342 (0–0·600)	0·732 (0–1·20)	0·370 (0–0·802)	0·858 (0–1·60)
Central Europe	45 to 49	2010	0·412 (0·200–0·700)	0·946 (0·300–1·50)	0·450 (0·200–1·00)	1·17 (0·300–2·10)
Central Europe	50 to 54	2010	0·470 (0·300–0·802)	1·17 (0·500–1·90)	0·501 (0·300–1·00)	1·46 (0·500–2·60)
Central Europe	55 to 59	2010	0·543 (0·400–0·900)	1·53 (0·800–2·30)	0·563 (0·400–1·00)	1·85 (0·800–3·20)
Central Europe	60 to 64	2010	0·612 (0·497–1·00)	2·16 (1·10–3·40)	0·619 (0·400–1·00)	2·41 (1·10–4·10)
Central Europe	65 to 69	2010	0·646 (0·500–1·00)	2·90 (1·40–4·60)	0·660 (0·500–1·00)	3·03 (1·30–4·90)
Central Europe	70 to 74	2010	0·668 (0·500–1·00)	3·74 (1·80–5·60)	0·687 (0·500–1·50)	3·81 (1·60–5·70)
Central Europe	75 to 79	2010	0·704 (0·500–1·70)	4·40 (1·90–6·20)	0·734 (0·500–2·00)	4·51 (1·90–6·30)
Central Europe	80 plus	2010	0·874 (0·500–2·60)	5·44 (2·20–7·20)	0·925 (0·500–2·70)	5·49 (2·10–7·10)
Central Europe	15 to 19	2020	0·00720 (0–0·100)	0·0116 (0–0·200)	0·00150 (0–0)	0·00270 (0–0)
Central Europe	20 to 24	2020	0·0411 (0–0·300)	0·0668 (0–0·500)	0·00830 (0–0)	0·0117 (0–0)
Central Europe	25 to 29	2020	0·118 (0–0·400)	0·208 (0–0·700)	0·0237 (0–0·300)	0·0396 (0–0·500)
Central Europe	30 to 34	2020	0·194 (0–0·400)	0·359 (0–0·902)	0·109 (0–0·400)	0·200 (0–1·00)
Central Europe	35 to 39	2020	0·267 (0–0·500)	0·517 (0–1·10)	0·260 (0–0·700)	0·526 (0–1·20)
Central Europe	40 to 44	2020	0·355 (0–0·700)	0·733 (0–1·20)	0·379 (0–0·900)	0·845 (0–1·60)
Central Europe	45 to 49	2020	0·433 (0·200–0·800)	0·952 (0·300–1·50)	0·462 (0·200–1·00)	1·13 (0·300–2·00)
Central Europe	50 to 54	2020	0·496 (0·300–0·900)	1·18 (0·600–1·90)	0·510 (0·300–1·00)	1·39 (0·500–2·30)
Central Europe	55 to 59	2020	0·567 (0·400–0·900)	1·53 (0·800–2·30)	0·576 (0·400–1·00)	1·77 (0·800–3·00)
Central Europe	60 to 64	2020	0·624 (0·500–1·00)	2·06 (1·10–3·10)	0·626 (0·400–1·00)	2·26 (1·00–3·80)
Central Europe	65 to 69	2020	0·661 (0·500–1·00)	2·77 (1·40–4·30)	0·665 (0·500–1·00)	2·88 (1·30–4·70)
Central Europe	70 to 74	2020	0·677 (0·500–1·00)	3·57 (1·70–5·30)	0·694 (0·500–1·21)	3·65 (1·60–5·50)
Central Europe	75 to 79	2020	0·704 (0·500–1·50)	4·24 (1·90–6·10)	0·728 (0·500–1·90)	4·34 (1·80–6·10)
Central Europe	80 plus	2020	0·868 (0·500–2·40)	5·35 (2·10–7·10)	0·918 (0·500–2·50)	5·41 (2·10–7·10)
Eastern Europe	15 to 19	1990	0·00530 (0–0·100)	0·00900 (0–0·100)	0·000600 (0–0)	0·00110 (0–0)
Eastern Europe	20 to 24	1990	0·0201 (0–0·202)	0·0332 (0–0·400)	0·00140 (0–0)	0·00290 (0–0)
Eastern Europe	25 to 29	1990	0·0427 (0–0·300)	0·0731 (0–0·600)	0·00790 (0–0·100)	0·0137 (0–0·200)
Eastern Europe	30 to 34	1990	0·102 (0–0·400)	0·177 (0–0·700)	0·0706 (0–0·400)	0·128 (0–0·900)
Eastern Europe	35 to 39	1990	0·216 (0–0·400)	0·413 (0–1·00)	0·235 (0–0·400)	0·504 (0–1·40)
Eastern Europe	40 to 44	1990	0·327 (0·0975–0·500)	0·737 (0·0975–1·30)	0·369 (0–0·600)	1·14 (0–2·80)
Eastern Europe	45 to 49	1990	0·403 (0·300–0·600)	1·12 (0·498–1·90)	0·457 (0·200–1·00)	1·72 (0·400–4·10)
Eastern Europe	50 to 54	1990	0·468 (0·400–0·600)	1·66 (0·800–2·70)	0·538 (0·300–1·00)	2·42 (0·700–4·90)

Eastern Europe	55 to 59	1990	0·538 (0·400–0·802)	2·56 (1·10–4·40)	0·619 (0·400–1·90)	3·16 (0·900–5·50)
Eastern Europe	60 to 64	1990	0·634 (0·500–1·70)	3·89 (1·50–5·90)	0·733 (0·500–2·00)	4·17 (1·40–6·20)
Eastern Europe	65 to 69	1990	0·801 (0·500–2·20)	5·01 (2·00–6·80)	0·886 (0·500–2·30)	4·95 (1·70–6·80)
Eastern Europe	70 to 74	1990	1·00 (0·500–2·80)	5·81 (2·40–7·40)	1·08 (0·500–2·80)	5·66 (2·10–7·20)
Eastern Europe	75 to 79	1990	1·18 (0·500–3·10)	6·35 (2·90–8·00)	1·27 (0·500–3·00)	6·26 (2·50–7·80)
Eastern Europe	80 plus	1990	1·52 (0·500–3·40)	6·93 (3·50–8·50)	1·66 (0·500–3·30)	6·89 (3·30–8·30)
Eastern Europe	15 to 19	2000	0·00430 (0–0)	0·00740 (0–0)	0·00630 (0–0)	0·00840 (0–0)
Eastern Europe	20 to 24	2000	0·0124 (0–0·200)	0·0216 (0–0·400)	0·0100 (0–0·100)	0·0146 (0–0·100)
Eastern Europe	25 to 29	2000	0·0387 (0–0·300)	0·0646 (0–0·600)	0·0252 (0–0·300)	0·0402 (0–0·500)
Eastern Europe	30 to 34	2000	0·118 (0–0·400)	0·210 (0–0·800)	0·104 (0–0·400)	0·188 (0–1·00)
Eastern Europe	35 to 39	2000	0·233 (0–0·400)	0·459 (0–1·00)	0·258 (0–0·500)	0·562 (0–1·40)
Eastern Europe	40 to 44	2000	0·332 (0–0·500)	0·754 (0–1·30)	0·372 (0·0975–0·602)	1·07 (0·0975–2·50)
Eastern Europe	45 to 49	2000	0·408 (0·200–0·600)	1·14 (0·400–2·00)	0·475 (0·200–1·00)	1·67 (0·400–4·20)
Eastern Europe	50 to 54	2000	0·471 (0·300–0·600)	1·65 (0·800–2·70)	0·554 (0·300–1·70)	2·36 (0·700–5·00)
Eastern Europe	55 to 59	2000	0·550 (0·400–1·00)	2·51 (1·10–4·40)	0·643 (0·400–2·00)	3·15 (0·900–5·60)
Eastern Europe	60 to 64	2000	0·656 (0·500–1·80)	3·95 (1·50–6·00)	0·799 (0·500–2·10)	4·40 (1·40–6·40)
Eastern Europe	65 to 69	2000	0·835 (0·500–2·20)	5·11 (2·00–6·80)	0·971 (0·500–2·40)	5·20 (1·80–7·00)
Eastern Europe	70 to 74	2000	1·06 (0·500–2·90)	5·94 (2·40–7·50)	1·19 (0·500–3·00)	5·93 (2·20–7·50)
Eastern Europe	75 to 79	2000	1·27 (0·500–3·10)	6·46 (2·90–8·00)	1·45 (0·500–3·10)	6·48 (2·70–7·90)
Eastern Europe	80 plus	2000	1·58 (0·500–3·50)	7·00 (3·60–8·50)	1·73 (0·500–3·40)	6·97 (3·40–8·30)
Eastern Europe	15 to 19	2010	0·00820 (0–0·102)	0·0141 (0–0·200)	0·00970 (0–0)	0·0113 (0–0)
Eastern Europe	20 to 24	2010	0·0207 (0–0·300)	0·0358 (0–0·400)	0·0116 (0–0·100)	0·0162 (0–0·102)
Eastern Europe	25 to 29	2010	0·0614 (0–0·300)	0·105 (0–0·700)	0·0305 (0–0·300)	0·0488 (0–0·600)
Eastern Europe	30 to 34	2010	0·137 (0–0·400)	0·249 (0–1·00)	0·123 (0–0·400)	0·225 (0–1·00)
Eastern Europe	35 to 39	2010	0·233 (0–0·600)	0·455 (0–1·10)	0·253 (0–0·600)	0·523 (0–1·30)
Eastern Europe	40 to 44	2010	0·332 (0–0·602)	0·714 (0–1·30)	0·366 (0–1·00)	0·910 (0–1·80)
Eastern Europe	45 to 49	2010	0·405 (0·200–0·700)	0·983 (0·300–1·70)	0·455 (0·200–1·00)	1·42 (0·400–3·10)
Eastern Europe	50 to 54	2010	0·469 (0·300–1·00)	1·37 (0·600–2·20)	0·551 (0·300–1·00)	2·10 (0·700–4·20)
Eastern Europe	55 to 59	2010	0·559 (0·400–1·00)	2·05 (0·900–3·50)	0·658 (0·400–1·80)	2·98 (0·900–5·30)
Eastern Europe	60 to 64	2010	0·670 (0·500–1·40)	3·44 (1·30–5·40)	0·820 (0·400–2·00)	4·15 (1·30–6·20)
Eastern Europe	65 to 69	2010	0·899 (0·500–2·20)	4·96 (1·80–6·70)	1·07 (0·500–2·50)	5·14 (1·60–6·90)
Eastern Europe	70 to 74	2010	1·18 (0·500–2·90)	5·94 (2·30–7·50)	1·29 (0·500–2·90)	5·90 (2·10–7·40)
Eastern Europe	75 to 79	2010	1·44 (0·500–3·10)	6·50 (2·80–7·90)	1·57 (0·500–3·00)	6·49 (2·60–7·90)
Eastern Europe	80 plus	2010	1·77 (0·500–3·40)	7·04 (3·60–8·40)	1·92 (0·500–3·30)	7·00 (3·30–8·20)
Eastern Europe	15 to 19	2020	0·0134 (0–0·200)	0·0228 (0–0·400)	0·0100 (0–0)	0·0119 (0–0)
Eastern Europe	20 to 24	2020	0·0315 (0–0·300)	0·0539 (0–0·500)	0·0136 (0–0·100)	0·0201 (0–0·200)
Eastern Europe	25 to 29	2020	0·0800 (0–0·400)	0·139 (0–0·800)	0·0315 (0–0·300)	0·0506 (0–0·700)
Eastern Europe	30 to 34	2020	0·156 (0–0·500)	0·286 (0–1·00)	0·111 (0–0·400)	0·204 (0–1·00)
Eastern Europe	35 to 39	2020	0·255 (0–0·600)	0·494 (0–1·10)	0·243 (0–0·700)	0·494 (0–1·20)
Eastern Europe	40 to 44	2020	0·342 (0–0·700)	0·720 (0–1·30)	0·362 (0–1·00)	0·845 (0–1·60)
Eastern Europe	45 to 49	2020	0·418 (0·200–0·800)	0·982 (0·300–1·70)	0·448 (0·200–1·00)	1·25 (0·300–2·30)

Eastern Europe	50 to 54	2020	0·483 (0·300–1·00)	1·35 (0·600–2·20)	0·534 (0·300–1·00)	1·89 (0·600–3·60)
Eastern Europe	55 to 59	2020	0·572 (0·400–1·00)	1·97 (0·900–3·30)	0·624 (0·400–1·00)	2·72 (0·900–4·80)
Eastern Europe	60 to 64	2020	0·670 (0·500–1·00)	3·25 (1·30–5·20)	0·779 (0·400–2·00)	3·90 (1·20–5·90)
Eastern Europe	65 to 69	2020	0·817 (0·500–2·00)	4·62 (1·90–6·40)	0·961 (0·500–2·30)	4·83 (1·50–6·60)
Eastern Europe	70 to 74	2020	1·08 (0·500–2·50)	5·69 (2·30–7·30)	1·21 (0·500–2·70)	5·67 (2·00–7·20)
Eastern Europe	75 to 79	2020	1·37 (0·500–3·00)	6·39 (2·90–7·80)	1·52 (0·500–3·00)	6·37 (2·50–7·70)
Eastern Europe	80 plus	2020	1·69 (0·600–3·30)	6·94 (3·40–8·30)	1·87 (0·500–3·30)	6·92 (3·30–8·20)
Australasia	15 to 19	1990	0·000300 (0–0)	0·000400 (0–0)	0·00260 (0–0)	0·00320 (0–0)
Australasia	20 to 24	1990	0·00120 (0–0)	0·00190 (0–0)	0·00770 (0–0)	0·00970 (0–0)
Australasia	25 to 29	1990	0·00330 (0–0)	0·00590 (0–0)	0·00830 (0–0)	0·0123 (0–0)
Australasia	30 to 34	1990	0·0154 (0–0·200)	0·0248 (0–0·400)	0·0328 (0–0·300)	0·0587 (0–0·602)
Australasia	35 to 39	1990	0·0368 (0–0·300)	0·0615 (0–0·500)	0·182 (0–0·400)	0·372 (0–1·30)
Australasia	40 to 44	1990	0·135 (0–0·400)	0·237 (0–0·800)	0·355 (0–0·802)	1·00 (0–2·50)
Australasia	45 to 49	1990	0·244 (0–0·402)	0·482 (0–1·10)	0·511 (0·200–1·00)	1·91 (0·200–4·10)
Australasia	50 to 54	1990	0·362 (0–0·900)	0·873 (0–1·80)	0·701 (0·300–2·00)	2·83 (0·600–5·20)
Australasia	55 to 59	1990	0·483 (0·200–1·00)	1·57 (0·400–3·20)	0·897 (0·400–2·00)	3·62 (0·900–5·80)
Australasia	60 to 64	1990	0·610 (0·300–1·00)	2·70 (0·800–5·00)	1·11 (0·400–2·30)	4·36 (1·10–6·30)
Australasia	65 to 69	1990	0·800 (0·400–2·00)	3·93 (1·10–6·00)	1·36 (0·500–2·60)	5·11 (1·40–6·80)
Australasia	70 to 74	1990	1·06 (0·500–2·40)	4·98 (1·50–6·80)	1·52 (0·500–2·80)	5·71 (1·90–7·20)
Australasia	75 to 79	1990	1·16 (0·500–2·60)	5·51 (2·00–7·10)	1·59 (0·500–2·90)	6·11 (2·30–7·50)
Australasia	80 plus	1990	1·19 (0·500–2·90)	5·85 (2·10–7·40)	1·57 (0·500–3·00)	6·29 (2·60–7·70)
Australasia	15 to 19	2000	0·000300 (0–0)	0·000400 (0–0)	0·00350 (0–0)	0·00380 (0–0)
Australasia	20 to 24	2000	0·00110 (0–0)	0·00190 (0–0)	0·00990 (0–0)	0·0126 (0–0)
Australasia	25 to 29	2000	0·00500 (0–0)	0·00910 (0–0)	0·0171 (0–0·200)	0·0237 (0–0·302)
Australasia	30 to 34	2000	0·0210 (0–0·300)	0·0358 (0–0·402)	0·0513 (0–0·400)	0·0865 (0–1·00)
Australasia	35 to 39	2000	0·0453 (0–0·300)	0·0753 (0–0·600)	0·161 (0–0·500)	0·316 (0–1·20)
Australasia	40 to 44	2000	0·109 (0–0·400)	0·188 (0–0·702)	0·314 (0–0·800)	0·766 (0–2·00)
Australasia	45 to 49	2000	0·198 (0–0·400)	0·363 (0–1·00)	0·432 (0–1·00)	1·29 (0–3·00)
Australasia	50 to 54	2000	0·286 (0–0·500)	0·569 (0–1·20)	0·552 (0·200–1·00)	1·95 (0·400–4·00)
Australasia	55 to 59	2000	0·405 (0·100–0·900)	0·969 (0·100–1·90)	0·667 (0·300–1·00)	2·62 (0·700–4·80)
Australasia	60 to 64	2000	0·532 (0·300–1·00)	1·60 (0·600–3·10)	0·763 (0·400–1·90)	3·23 (0·998–5·30)
Australasia	65 to 69	2000	0·635 (0·400–1·00)	2·59 (0·900–4·50)	0·900 (0·500–2·00)	3·96 (1·20–6·00)
Australasia	70 to 74	2000	0·708 (0·500–1·00)	3·69 (1·20–5·70)	1·04 (0·500–2·30)	4·68 (1·50–6·40)
Australasia	75 to 79	2000	0·799 (0·500–2·10)	4·50 (1·70–6·40)	1·21 (0·500–2·50)	5·31 (2·00–7·00)
Australasia	80 plus	2000	1·03 (0·500–2·70)	5·44 (1·90–7·10)	1·34 (0·600–2·90)	5·89 (2·30–7·40)
Australasia	15 to 19	2010	0·000300 (0–0)	0·000500 (0–0)	0·00350 (0–0)	0·00440 (0–0)
Australasia	20 to 24	2010	0·00200 (0–0)	0·00350 (0–0)	0·00690 (0–0)	0·00790 (0–0)
Australasia	25 to 29	2010	0·00390 (0–0)	0·00650 (0–0)	0·0105 (0–0)	0·0148 (0–0)
Australasia	30 to 34	2010	0·0145 (0–0·200)	0·0236 (0–0·400)	0·0430 (0–0·400)	0·0727 (0–0·900)
Australasia	35 to 39	2010	0·0408 (0–0·300)	0·0686 (0–0·500)	0·143 (0–0·500)	0·274 (0–1·20)
Australasia	40 to 44	2010	0·0937 (0–0·400)	0·159 (0–0·700)	0·283 (0–0·700)	0·631 (0–1·70)

Australasia	45 to 49	2010	0·188 (0–0·500)	0·340 (0–1·00)	0·393 (0–0·802)	0·999 (0–2·20)
Australasia	50 to 54	2010	0·257 (0–0·600)	0·478 (0–1·10)	0·477 (0·198–1·00)	1·34 (0·298–2·70)
Australasia	55 to 59	2010	0·354 (0–0·700)	0·730 (0–1·30)	0·540 (0·300–1·00)	1·69 (0·500–3·30)
Australasia	60 to 64	2010	0·461 (0·200–0·800)	1·06 (0·300–1·90)	0·615 (0·400–1·00)	2·22 (0·798–4·00)
Australasia	65 to 69	2010	0·545 (0·300–0·900)	1·54 (0·700–2·80)	0·688 (0·400–1·00)	2·81 (1·00–4·80)
Australasia	70 to 74	2010	0·601 (0·400–0·900)	2·28 (1·00–3·90)	0·733 (0·500–1·10)	3·44 (1·20–5·40)
Australasia	75 to 79	2010	0·637 (0·500–1·00)	3·14 (1·20–5·10)	0·794 (0·500–2·00)	4·14 (1·50–6·00)
Australasia	80 plus	2010	0·743 (0·500–2·20)	4·52 (1·60–6·40)	1·01 (0·597–2·40)	5·16 (1·90–6·80)
Australasia	15 to 19	2020	0·00290 (0–0)	0·00370 (0–0)	0·00600 (0–0)	0·00710 (0–0)
Australasia	20 to 24	2020	0·00490 (0–0)	0·00740 (0–0)	0·0143 (0–0)	0·0185 (0–0)
Australasia	25 to 29	2020	0·00660 (0–0)	0·0108 (0–0)	0·0203 (0–0·202)	0·0259 (0–0·400)
Australasia	30 to 34	2020	0·0188 (0–0·300)	0·0315 (0–0·400)	0·0492 (0–0·400)	0·0848 (0–1·00)
Australasia	35 to 39	2020	0·0509 (0–0·400)	0·0835 (0–0·602)	0·148 (0–0·600)	0·284 (0–1·20)
Australasia	40 to 44	2020	0·114 (0–0·402)	0·193 (0–0·900)	0·294 (0–0·800)	0·637 (0–1·60)
Australasia	45 to 49	2020	0·196 (0–0·500)	0·352 (0–1·00)	0·414 (0–0·900)	1·02 (0–2·20)
Australasia	50 to 54	2020	0·279 (0–0·700)	0·520 (0–1·10)	0·499 (0·200–0·900)	1·37 (0·300–2·80)
Australasia	55 to 59	2020	0·381 (0–0·800)	0·763 (0–1·30)	0·550 (0·300–1·00)	1·59 (0·500–3·00)
Australasia	60 to 64	2020	0·470 (0·200–0·800)	1·04 (0·300–1·80)	0·610 (0·400–1·00)	2·02 (0·798–3·60)
Australasia	65 to 69	2020	0·534 (0·300–0·800)	1·39 (0·700–2·40)	0·671 (0·400–1·00)	2·56 (1·00–4·40)
Australasia	70 to 74	2020	0·579 (0·400–0·800)	1·92 (0·900–3·40)	0·702 (0·500–1·00)	3·18 (1·20–5·10)
Australasia	75 to 79	2020	0·609 (0·500–0·900)	2·65 (1·10–4·40)	0·743 (0·500–1·80)	3·83 (1·50–5·70)
Australasia	80 plus	2020	0·674 (0·500–1·50)	4·08 (1·40–6·10)	0·902 (0·500–2·30)	4·87 (1·70–6·60)
High-income Asia Pacific	15 to 19	1990	0·0393 (0–0·300)	0·0671 (0–0·500)	0·0113 (0–0·200)	0·0197 (0–0·300)
High-income Asia Pacific	20 to 24	1990	0·0551 (0–0·300)	0·0947 (0–0·700)	0·0109 (0–0·200)	0·0200 (0–0·300)
High-income Asia Pacific	25 to 29	1990	0·118 (0–0·400)	0·212 (0–0·900)	0·0422 (0–0·300)	0·0759 (0–0·700)
High-income Asia Pacific	30 to 34	1990	0·166 (0–0·400)	0·308 (0–0·900)	0·131 (0–0·400)	0·245 (0–1·00)
High-income Asia Pacific	35 to 39	1990	0·217 (0–0·400)	0·413 (0–1·00)	0·236 (0–0·500)	0·474 (0–1·20)
High-income Asia Pacific	40 to 44	1990	0·286 (0–0·400)	0·580 (0–1·10)	0·321 (0–0·600)	0·688 (0–1·30)
High-income Asia Pacific	45 to 49	1990	0·356 (0·100–0·500)	0·802 (0·100–1·30)	0·378 (0–0·700)	0·861 (0–1·50)
High-income Asia Pacific	50 to 54	1990	0·405 (0·200–0·600)	0·997 (0·398–1·50)	0·414 (0·200–0·700)	0·987 (0·300–1·70)
High-income Asia Pacific	55 to 59	1990	0·447 (0·300–0·600)	1·19 (0·600–1·80)	0·442 (0·300–0·600)	1·13 (0·500–1·90)
High-income Asia Pacific	60 to 64	1990	0·496 (0·400–0·600)	1·53 (0·900–2·20)	0·477 (0·300–0·600)	1·42 (0·798–2·20)
High-income Asia Pacific	65 to 69	1990	0·519 (0·400–0·600)	1·87 (1·10–2·70)	0·500 (0·400–0·600)	1·69 (0·998–2·60)
High-income Asia Pacific	70 to 74	1990	0·544 (0·500–0·700)	2·30 (1·40–3·50)	0·521 (0·400–0·600)	2·07 (1·10–3·40)
High-income Asia Pacific	75 to 79	1990	0·560 (0·500–0·802)	2·79 (1·60–4·40)	0·550 (0·500–0·902)	2·58 (1·30–4·30)
High-income Asia Pacific	80 plus	1990	0·607 (0·500–1·20)	3·57 (1·80–5·40)	0·591 (0·500–1·00)	3·29 (1·70–5·30)
High-income Asia Pacific	15 to 19	2000	0·0124 (0–0·200)	0·0213 (0–0·300)	0·00460 (0–0)	0·00840 (0–0)
High-income Asia Pacific	20 to 24	2000	0·0287 (0–0·300)	0·0485 (0–0·600)	0·0158 (0–0·200)	0·0249 (0–0·400)
High-income Asia Pacific	25 to 29	2000	0·0644 (0–0·400)	0·113 (0–1·00)	0·0386 (0–0·400)	0·0665 (0–0·900)
High-income Asia Pacific	30 to 34	2000	0·104 (0–0·400)	0·186 (0–1·00)	0·114 (0–0·400)	0·212 (0–1·10)
High-income Asia Pacific	35 to 39	2000	0·152 (0–0·400)	0·277 (0–0·900)	0·207 (0–0·500)	0·406 (0–1·10)

High-income Asia Pacific	40 to 44	2000	0·231 (0–0·400)	0·442 (0–1·00)	0·294 (0–0·600)	0·609 (0–1·20)
High-income Asia Pacific	45 to 49	2000	0·306 (0–0·500)	0·624 (0–1·10)	0·364 (0–0·600)	0·803 (0–1·40)
High-income Asia Pacific	50 to 54	2000	0·361 (0–0·500)	0·785 (0–1·20)	0·405 (0·100–0·700)	0·948 (0·200–1·60)
High-income Asia Pacific	55 to 59	2000	0·426 (0·200–0·600)	1·01 (0·400–1·50)	0·430 (0·200–0·600)	1·05 (0·400–1·80)
High-income Asia Pacific	60 to 64	2000	0·489 (0·300–0·600)	1·32 (0·700–2·00)	0·468 (0·300–0·600)	1·25 (0·600–2·00)
High-income Asia Pacific	65 to 69	2000	0·519 (0·400–0·600)	1·61 (1·00–2·40)	0·488 (0·400–0·600)	1·46 (0·800–2·30)
High-income Asia Pacific	70 to 74	2000	0·536 (0·400–0·700)	1·93 (1·20–2·80)	0·509 (0·400–0·600)	1·76 (1·00–2·70)
High-income Asia Pacific	75 to 79	2000	0·546 (0·500–0·700)	2·27 (1·30–3·50)	0·523 (0·400–0·600)	2·12 (1·20–3·30)
High-income Asia Pacific	80 plus	2000	0·573 (0·500–1·00)	2·98 (1·60–4·80)	0·558 (0·500–1·00)	2·74 (1·40–4·50)
High-income Asia Pacific	15 to 19	2010	0·0326 (0–0·300)	0·0510 (0–0·600)	0·0119 (0–0·100)	0·0170 (0–0·100)
High-income Asia Pacific	20 to 24	2010	0·0789 (0–1·00)	0·126 (0–1·10)	0·0675 (0–1·00)	0·0956 (0–1·10)
High-income Asia Pacific	25 to 29	2010	0·112 (0–1·00)	0·184 (0–1·20)	0·100 (0–1·00)	0·153 (0–1·20)
High-income Asia Pacific	30 to 34	2010	0·124 (0–0·900)	0·214 (0–1·20)	0·165 (0–1·00)	0·299 (0–1·40)
High-income Asia Pacific	35 to 39	2010	0·148 (0–0·600)	0·266 (0–1·10)	0·253 (0–1·00)	0·494 (0–1·40)
High-income Asia Pacific	40 to 44	2010	0·200 (0–0·500)	0·373 (0–1·10)	0·335 (0–1·00)	0·702 (0–1·50)
High-income Asia Pacific	45 to 49	2010	0·269 (0–0·500)	0·523 (0–1·10)	0·387 (0–0·900)	0·848 (0–1·50)
High-income Asia Pacific	50 to 54	2010	0·335 (0–0·600)	0·680 (0–1·20)	0·424 (0·200–0·800)	0·989 (0·298–1·70)
High-income Asia Pacific	55 to 59	2010	0·393 (0·100–0·600)	0·840 (0·200–1·30)	0·449 (0·300–0·700)	1·13 (0·400–1·90)
High-income Asia Pacific	60 to 64	2010	0·455 (0·300–0·602)	1·08 (0·500–1·70)	0·474 (0·300–0·700)	1·29 (0·600–2·10)
High-income Asia Pacific	65 to 69	2010	0·505 (0·400–0·700)	1·34 (0·800–2·10)	0·489 (0·400–0·600)	1·43 (0·800–2·20)
High-income Asia Pacific	70 to 74	2010	0·534 (0·400–0·700)	1·63 (1·00–2·40)	0·510 (0·400–0·600)	1·64 (0·900–2·50)
High-income Asia Pacific	75 to 79	2010	0·543 (0·400–0·700)	1·93 (1·10–3·00)	0·518 (0·400–0·600)	1·94 (1·10–3·10)
High-income Asia Pacific	80 plus	2010	0·564 (0·500–1·00)	2·68 (1·30–4·40)	0·548 (0·500–0·900)	2·54 (1·30–4·20)
High-income Asia Pacific	15 to 19	2020	0·0433 (0–0·400)	0·0719 (0–0·800)	0·0179 (0–0·200)	0·0256 (0–0·400)
High-income Asia Pacific	20 to 24	2020	0·0888 (0–1·00)	0·147 (0–1·10)	0·0743 (0–1·00)	0·105 (0–1·20)
High-income Asia Pacific	25 to 29	2020	0·123 (0–0·805)	0·212 (0–1·20)	0·115 (0–1·00)	0·180 (0–1·30)
High-income Asia Pacific	30 to 34	2020	0·153 (0–0·700)	0·271 (0–1·20)	0·196 (0–1·00)	0·359 (0–1·40)
High-income Asia Pacific	35 to 39	2020	0·194 (0–0·600)	0·352 (0–1·10)	0·291 (0–1·00)	0·574 (0–1·40)
High-income Asia Pacific	40 to 44	2020	0·256 (0–0·600)	0·478 (0–1·10)	0·380 (0–0·900)	0·818 (0–1·60)
High-income Asia Pacific	45 to 49	2020	0·322 (0–0·600)	0·627 (0–1·20)	0·436 (0·100–0·900)	0·986 (0·198–1·70)
High-income Asia Pacific	50 to 54	2020	0·375 (0–0·700)	0·762 (0–1·20)	0·462 (0·200–0·800)	1·10 (0·398–1·90)
High-income Asia Pacific	55 to 59	2020	0·438 (0·200–0·700)	0·942 (0·398–1·40)	0·480 (0·300–0·700)	1·19 (0·500–2·00)
High-income Asia Pacific	60 to 64	2020	0·505 (0·300–0·700)	1·20 (0·700–1·80)	0·500 (0·300–0·700)	1·37 (0·700–2·10)
High-income Asia Pacific	65 to 69	2020	0·531 (0·400–0·700)	1·43 (0·898–2·10)	0·511 (0·400–0·602)	1·54 (0·800–2·40)
High-income Asia Pacific	70 to 74	2020	0·548 (0·400–0·700)	1·68 (1·00–2·50)	0·522 (0·400–0·600)	1·73 (1·00–2·70)
High-income Asia Pacific	75 to 79	2020	0·557 (0·400–0·700)	2·00 (1·20–3·00)	0·530 (0·400–0·700)	1·98 (1·10–3·10)
High-income Asia Pacific	80 plus	2020	0·572 (0·500–1·00)	2·69 (1·40–4·30)	0·554 (0·500–0·900)	2·54 (1·30–4·10)
High-income North America	15 to 19	1990	0·00610 (0–0)	0·0106 (0–0)	0·00250 (0–0)	0·00400 (0–0)
High-income North America	20 to 24	1990	0·0116 (0–0·200)	0·0205 (0–0·302)	0·00430 (0–0)	0·00620 (0–0)
High-income North America	25 to 29	1990	0·0330 (0–0·300)	0·0584 (0–0·600)	0·00950 (0–0·102)	0·0172 (0–0·202)
High-income North America	30 to 34	1990	0·0845 (0–0·400)	0·148 (0–0·800)	0·0503 (0–0·400)	0·0928 (0–1·00)

High-income North America	35 to 39	1990	0·167 (0–0·400)	0·306 (0–1·00)	0·238 (0–0·600)	0·489 (0–1·30)
High-income North America	40 to 44	1990	0·277 (0–0·600)	0·549 (0–1·10)	0·428 (0–1·00)	1·15 (0–2·30)
High-income North America	45 to 49	1990	0·400 (0–0·900)	0·911 (0–1·60)	0·623 (0·300–1·00)	2·05 (0·500–4·00)
High-income North America	50 to 54	1990	0·521 (0·200–1·00)	1·41 (0·400–2·50)	0·823 (0·400–2·00)	2·98 (0·800–5·20)
High-income North America	55 to 59	1990	0·634 (0·300–1·00)	2·08 (0·800–3·70)	0·989 (0·400–2·00)	3·70 (1·10–5·80)
High-income North America	60 to 64	1990	0·742 (0·400–1·00)	3·02 (1·10–5·00)	1·17 (0·500–2·30)	4·43 (1·30–6·30)
High-income North America	65 to 69	1990	0·830 (0·500–2·00)	3·79 (1·20–5·80)	1·35 (0·500–2·40)	5·01 (1·60–6·70)
High-income North America	70 to 74	1990	0·968 (0·500–2·20)	4·64 (1·60–6·40)	1·53 (0·500–2·70)	5·58 (1·90–7·10)
High-income North America	75 to 79	1990	1·15 (0·500–2·40)	5·29 (1·90–6·90)	1·66 (0·600–2·80)	5·99 (2·20–7·40)
High-income North America	80 plus	1990	1·50 (0·500–3·00)	6·18 (2·40–7·60)	1·85 (0·600–3·00)	6·46 (2·50–7·70)
High-income North America	15 to 19	2000	0·0115 (0–0·200)	0·0203 (0–0·300)	0·00440 (0–0)	0·00660 (0–0)
High-income North America	20 to 24	2000	0·0227 (0–0·300)	0·0400 (0–0·600)	0·00670 (0–0)	0·0104 (0–0)
High-income North America	25 to 29	2000	0·0665 (0–0·400)	0·116 (0–0·802)	0·0166 (0–0·300)	0·0282 (0–0·500)
High-income North America	30 to 34	2000	0·149 (0–0·500)	0·268 (0–1·00)	0·0770 (0–0·500)	0·138 (0–1·00)
High-income North America	35 to 39	2000	0·239 (0–0·600)	0·456 (0–1·10)	0·263 (0–0·800)	0·539 (0–1·30)
High-income North America	40 to 44	2000	0·340 (0–0·700)	0·706 (0–1·30)	0·430 (0–1·00)	1·06 (0–2·20)
High-income North America	45 to 49	2000	0·429 (0·100–0·900)	0·962 (0·198–1·70)	0·575 (0·200–1·00)	1·59 (0·400–3·00)
High-income North America	50 to 54	2000	0·538 (0·300–1·00)	1·36 (0·500–2·30)	0·730 (0·398–1·00)	2·27 (0·700–4·10)
High-income North America	55 to 59	2000	0·640 (0·400–1·00)	1·92 (0·800–3·30)	0·854 (0·400–2·00)	3·03 (1·00–5·00)
High-income North America	60 to 64	2000	0·720 (0·500–1·00)	2·71 (1·10–4·50)	0·957 (0·500–2·00)	3·77 (1·20–5·70)
High-income North America	65 to 69	2000	0·767 (0·500–1·00)	3·32 (1·30–5·20)	1·07 (0·500–2·10)	4·32 (1·50–6·10)
High-income North America	70 to 74	2000	0·794 (0·500–1·90)	3·97 (1·50–5·80)	1·20 (0·600–2·30)	4·92 (1·80–6·60)
High-income North America	75 to 79	2000	0·876 (0·500–2·10)	4·63 (1·70–6·40)	1·34 (0·600–2·40)	5·45 (2·10–7·00)
High-income North America	80 plus	2000	1·20 (0·500–2·80)	5·70 (2·10–7·30)	1·58 (0·600–2·90)	6·10 (2·40–7·50)
High-income North America	15 to 19	2010	0·0211 (0–0·300)	0·0370 (0–0·600)	0·00730 (0–0)	0·0112 (0–0)
High-income North America	20 to 24	2010	0·0360 (0–0·400)	0·0625 (0–0·700)	0·0109 (0–0)	0·0170 (0–0)
High-income North America	25 to 29	2010	0·0920 (0–0·500)	0·163 (0–1·00)	0·0268 (0–0·300)	0·0436 (0–0·700)
High-income North America	30 to 34	2010	0·197 (0–0·600)	0·360 (0–1·10)	0·106 (0–0·500)	0·187 (0–1·10)
High-income North America	35 to 39	2010	0·291 (0–0·700)	0·556 (0–1·20)	0·287 (0–0·800)	0·582 (0–1·40)
High-income North America	40 to 44	2010	0·384 (0–0·800)	0·784 (0–1·40)	0·452 (0–1·00)	1·05 (0–2·10)
High-income North America	45 to 49	2010	0·472 (0·200–0·900)	1·02 (0·300–1·70)	0·571 (0·200–1·00)	1·43 (0·400–2·60)
High-income North America	50 to 54	2010	0·549 (0·300–1·00)	1·27 (0·500–2·10)	0·660 (0·300–1·00)	1·76 (0·600–3·10)
High-income North America	55 to 59	2010	0·626 (0·400–1·00)	1·57 (0·800–2·50)	0·743 (0·400–1·00)	2·13 (0·898–3·60)
High-income North America	60 to 64	2010	0·686 (0·400–1·00)	2·08 (1·10–3·30)	0·814 (0·500–1·00)	2·77 (1·10–4·50)
High-income North America	65 to 69	2010	0·716 (0·500–1·00)	2·53 (1·20–4·00)	0·863 (0·500–1·50)	3·40 (1·30–5·20)
High-income North America	70 to 74	2010	0·715 (0·500–1·00)	3·07 (1·40–4·80)	0·888 (0·600–2·00)	3·99 (1·70–5·80)
High-income North America	75 to 79	2010	0·722 (0·500–1·00)	3·65 (1·50–5·50)	0·970 (0·600–2·00)	4·58 (1·90–6·30)
High-income North America	80 plus	2010	0·854 (0·500–2·30)	4·89 (1·80–6·70)	1·24 (0·600–2·50)	5·49 (2·10–7·10)
High-income North America	15 to 19	2020	0·0207 (0–0·300)	0·0346 (0–0·600)	0·0132 (0–0)	0·0169 (0–0)
High-income North America	20 to 24	2020	0·0359 (0–0·400)	0·0611 (0–0·702)	0·0168 (0–0·102)	0·0227 (0–0·202)
High-income North America	25 to 29	2020	0·0951 (0–0·500)	0·166 (0–1·00)	0·0319 (0–0·400)	0·0496 (0–0·900)

High-income North America	30 to 34	2020	0·211 (0–0·600)	0·384 (0–1·10)	0·119 (0–0·700)	0·212 (0–1·10)
High-income North America	35 to 39	2020	0·318 (0–0·700)	0·611 (0–1·20)	0·305 (0–0·800)	0·627 (0–1·40)
High-income North America	40 to 44	2020	0·419 (0–0·800)	0·846 (0–1·40)	0·475 (0·100–1·00)	1·10 (0·100–2·10)
High-income North America	45 to 49	2020	0·498 (0·200–0·900)	1·04 (0·300–1·70)	0·589 (0·300–1·00)	1·45 (0·500–2·60)
High-income North America	50 to 54	2020	0·567 (0·300–1·00)	1·28 (0·600–2·10)	0·678 (0·398–1·00)	1·78 (0·700–3·10)
High-income North America	55 to 59	2020	0·636 (0·400–1·00)	1·57 (0·800–2·50)	0·745 (0·400–1·00)	2·10 (0·900–3·50)
High-income North America	60 to 64	2020	0·689 (0·500–1·00)	2·00 (1·10–3·20)	0·801 (0·500–1·00)	2·62 (1·10–4·30)
High-income North America	65 to 69	2020	0·709 (0·500–1·00)	2·36 (1·20–3·70)	0·846 (0·500–1·00)	3·18 (1·30–5·00)
High-income North America	70 to 74	2020	0·705 (0·500–1·00)	2·87 (1·30–4·50)	0·859 (0·600–1·90)	3·79 (1·60–5·60)
High-income North America	75 to 79	2020	0·698 (0·500–1·00)	3·36 (1·40–5·20)	0·901 (0·600–2·00)	4·37 (1·90–6·10)
High-income North America	80 plus	2020	0·782 (0·500–2·10)	4·64 (1·70–6·50)	1·13 (0·600–2·40)	5·30 (2·10–6·90)
Southern Latin America	15 to 19	1990	0·0438 (0–0·300)	0·0717 (0–0·500)	0·00330 (0–0)	0·00560 (0–0)
Southern Latin America	20 to 24	1990	0·0850 (0–0·300)	0·145 (0–0·600)	0·00510 (0–0·100)	0·00920 (0–0·100)
Southern Latin America	25 to 29	1990	0·143 (0–0·300)	0·258 (0–0·700)	0·0157 (0–0·200)	0·0274 (0–0·400)
Southern Latin America	30 to 34	1990	0·206 (0–0·400)	0·386 (0–0·900)	0·0769 (0–0·300)	0·136 (0–0·700)
Southern Latin America	35 to 39	1990	0·270 (0–0·400)	0·555 (0–1·10)	0·227 (0–0·400)	0·461 (0–1·10)
Southern Latin America	40 to 44	1990	0·328 (0–0·500)	0·744 (0–1·20)	0·345 (0–0·500)	0·841 (0–1·60)
Southern Latin America	45 to 49	1990	0·378 (0·100–0·500)	0·950 (0·200–1·50)	0·418 (0·200–1·00)	1·16 (0·300–2·10)
Southern Latin America	50 to 54	1990	0·439 (0·300–0·700)	1·22 (0·500–2·00)	0·480 (0·300–1·00)	1·51 (0·600–2·60)
Southern Latin America	55 to 59	1990	0·501 (0·400–0·800)	1·52 (0·800–2·30)	0·540 (0·400–1·00)	1·86 (0·800–3·20)
Southern Latin America	60 to 64	1990	0·572 (0·400–0·802)	2·03 (1·10–3·10)	0·602 (0·400–1·00)	2·34 (1·10–4·00)
Southern Latin America	65 to 69	1990	0·612 (0·500–1·00)	2·56 (1·40–4·10)	0·655 (0·500–1·00)	2·87 (1·30–4·90)
Southern Latin America	70 to 74	1990	0·641 (0·500–1·00)	3·20 (1·60–5·00)	0·679 (0·500–1·50)	3·40 (1·50–5·40)
Southern Latin America	75 to 79	1990	0·666 (0·500–1·00)	3·82 (1·80–5·70)	0·712 (0·500–1·90)	3·98 (1·60–5·90)
Southern Latin America	80 plus	1990	0·789 (0·500–2·10)	4·87 (1·90–6·70)	0·792 (0·500–2·00)	4·78 (1·90–6·60)
Southern Latin America	15 to 19	2000	0·00900 (0–0·102)	0·0149 (0–0·202)	0·00250 (0–0)	0·00370 (0–0)
Southern Latin America	20 to 24	2000	0·0223 (0–0·200)	0·0363 (0–0·400)	0·00400 (0–0)	0·00650 (0–0)
Southern Latin America	25 to 29	2000	0·0571 (0–0·300)	0·0940 (0–0·500)	0·00700 (0–0·100)	0·0125 (0–0·200)
Southern Latin America	30 to 34	2000	0·118 (0–0·300)	0·205 (0–0·700)	0·0369 (0–0·300)	0·0653 (0–0·600)
Southern Latin America	35 to 39	2000	0·195 (0–0·400)	0·366 (0–0·900)	0·152 (0–0·400)	0·289 (0–1·00)
Southern Latin America	40 to 44	2000	0·288 (0–0·400)	0·600 (0–1·10)	0·301 (0–0·500)	0·672 (0–1·30)
Southern Latin America	45 to 49	2000	0·366 (0·100–0·500)	0·857 (0·100–1·30)	0·401 (0·100–0·700)	1·05 (0·200–2·00)
Southern Latin America	50 to 54	2000	0·436 (0·200–0·700)	1·11 (0·400–1·70)	0·474 (0·300–1·00)	1·40 (0·500–2·30)
Southern Latin America	55 to 59	2000	0·509 (0·300–0·800)	1·38 (0·700–2·10)	0·532 (0·300–1·00)	1·68 (0·700–2·80)
Southern Latin America	60 to 64	2000	0·577 (0·400–0·900)	1·79 (1·10–2·70)	0·586 (0·400–1·00)	2·05 (1·00–3·40)
Southern Latin America	65 to 69	2000	0·615 (0·500–0·900)	2·18 (1·20–3·30)	0·629 (0·497–1·00)	2·43 (1·20–4·00)
Southern Latin America	70 to 74	2000	0·631 (0·500–1·00)	2·62 (1·40–4·10)	0·652 (0·500–1·00)	2·92 (1·40–4·70)
Southern Latin America	75 to 79	2000	0·638 (0·500–1·00)	3·09 (1·60–4·80)	0·669 (0·500–1·00)	3·38 (1·60–5·30)
Southern Latin America	80 plus	2000	0·685 (0·500–1·50)	3·94 (1·60–5·90)	0·701 (0·500–1·50)	3·98 (1·60–5·90)
Southern Latin America	15 to 19	2010	0·00670 (0–0·100)	0·0114 (0–0·200)	0·00370 (0–0)	0·00670 (0–0)
Southern Latin America	20 to 24	2010	0·0132 (0–0·200)	0·0226 (0–0·300)	0·00420 (0–0)	0·00780 (0–0)

Southern Latin America	25 to 29	2010	0·0366 (0–0·300)	0·0607 (0–0·500)	0·00660 (0–0·100)	0·0116 (0–0·100)
Southern Latin America	30 to 34	2010	0·0809 (0–0·300)	0·136 (0–0·600)	0·0257 (0–0·300)	0·0467 (0–0·600)
Southern Latin America	35 to 39	2010	0·152 (0–0·400)	0·271 (0–0·800)	0·114 (0–0·400)	0·212 (0–1·00)
Southern Latin America	40 to 44	2010	0·259 (0–0·500)	0·511 (0–1·00)	0·264 (0–0·500)	0·557 (0–1·20)
Southern Latin America	45 to 49	2010	0·354 (0–0·600)	0·777 (0–1·20)	0·387 (0–0·800)	0·917 (0–1·70)
Southern Latin America	50 to 54	2010	0·441 (0·200–0·800)	1·03 (0·400–1·60)	0·479 (0·200–1·00)	1·27 (0·400–2·20)
Southern Latin America	55 to 59	2010	0·522 (0·300–0·800)	1·29 (0·700–2·00)	0·550 (0·300–1·00)	1·57 (0·698–2·60)
Southern Latin America	60 to 64	2010	0·592 (0·400–0·900)	1·66 (1·00–2·50)	0·599 (0·400–1·00)	1·91 (0·900–3·10)
Southern Latin America	65 to 69	2010	0·625 (0·500–0·900)	1·99 (1·20–3·00)	0·638 (0·400–1·00)	2·21 (1·10–3·60)
Southern Latin America	70 to 74	2010	0·636 (0·500–1·00)	2·33 (1·30–3·50)	0·646 (0·500–1·00)	2·60 (1·30–4·20)
Southern Latin America	75 to 79	2010	0·632 (0·500–1·00)	2·70 (1·40–4·20)	0·659 (0·500–1·00)	3·02 (1·50–4·80)
Southern Latin America	80 plus	2010	0·666 (0·500–1·00)	3·41 (1·50–5·30)	0·681 (0·500–1·10)	3·58 (1·50–5·40)
Southern Latin America	15 to 19	2020	0·0116 (0–0·200)	0·0189 (0–0·300)	0·00630 (0–0)	0·00900 (0–0)
Southern Latin America	20 to 24	2020	0·0202 (0–0·200)	0·0347 (0–0·400)	0·00890 (0–0)	0·0117 (0–0)
Southern Latin America	25 to 29	2020	0·0446 (0–0·300)	0·0753 (0–0·600)	0·0115 (0–0·102)	0·0184 (0–0·202)
Southern Latin America	30 to 34	2020	0·0972 (0–0·400)	0·168 (0–0·700)	0·0331 (0–0·300)	0·0583 (0–0·700)
Southern Latin America	35 to 39	2020	0·175 (0–0·400)	0·315 (0–0·900)	0·130 (0–0·400)	0·243 (0–1·00)
Southern Latin America	40 to 44	2020	0·283 (0–0·500)	0·551 (0–1·10)	0·284 (0–0·600)	0·598 (0–1·20)
Southern Latin America	45 to 49	2020	0·376 (0–0·700)	0·798 (0–1·30)	0·410 (0–0·900)	0·948 (0–1·70)
Southern Latin America	50 to 54	2020	0·467 (0·200–0·800)	1·04 (0·400–1·60)	0·507 (0·200–1·00)	1·27 (0·400–2·10)
Southern Latin America	55 to 59	2020	0·550 (0·300–0·900)	1·30 (0·700–2·00)	0·572 (0·300–1·00)	1·52 (0·700–2·40)
Southern Latin America	60 to 64	2020	0·618 (0·400–0·900)	1·66 (1·00–2·40)	0·621 (0·400–1·00)	1·86 (1·00–3·00)
Southern Latin America	65 to 69	2020	0·641 (0·500–0·900)	1·95 (1·10–2·90)	0·656 (0·500–1·00)	2·17 (1·10–3·40)
Southern Latin America	70 to 74	2020	0·646 (0·500–0·902)	2·24 (1·30–3·30)	0·663 (0·500–1·00)	2·51 (1·30–4·00)
Southern Latin America	75 to 79	2020	0·641 (0·500–1·00)	2·55 (1·40–3·90)	0·668 (0·500–1·00)	2·88 (1·50–4·50)
Southern Latin America	80 plus	2020	0·661 (0·500–1·00)	3·19 (1·40–5·10)	0·681 (0·500–1·00)	3·43 (1·60–5·30)
Western Europe	15 to 19	1990	0·00750 (0–0·100)	0·0123 (0–0·200)	0·00270 (0–0)	0·00480 (0–0)
Western Europe	20 to 24	1990	0·0191 (0–0·200)	0·0335 (0–0·400)	0·00670 (0–0·00250)	0·0118 (0–0·00250)
Western Europe	25 to 29	1990	0·0516 (0–0·300)	0·0883 (0–0·700)	0·0224 (0–0·300)	0·0375 (0–0·500)
Western Europe	30 to 34	1990	0·0940 (0–0·400)	0·165 (0–0·800)	0·0873 (0–0·400)	0·161 (0–1·00)
Western Europe	35 to 39	1990	0·130 (0–0·400)	0·233 (0–0·800)	0·237 (0–0·600)	0·493 (0–1·30)
Western Europe	40 to 44	1990	0·177 (0–0·400)	0·328 (0–1·00)	0·351 (0–0·702)	0·863 (0–1·90)
Western Europe	45 to 49	1990	0·255 (0–0·500)	0·494 (0–1·10)	0·442 (0·100–1·00)	1·27 (0·200–2·50)
Western Europe	50 to 54	1990	0·342 (0–0·700)	0·741 (0–1·30)	0·524 (0·300–1·00)	1·68 (0·400–3·20)
Western Europe	55 to 59	1990	0·443 (0·200–1·00)	1·12 (0·300–2·00)	0·628 (0·300–1·00)	2·22 (0·700–4·10)
Western Europe	60 to 64	1990	0·568 (0·400–1·00)	1·92 (0·800–3·30)	0·746 (0·400–1·90)	3·01 (1·00–5·10)
Western Europe	65 to 69	1990	0·643 (0·400–1·00)	2·85 (1·10–4·70)	0·846 (0·500–2·00)	3·81 (1·20–5·80)
Western Europe	70 to 74	1990	0·685 (0·500–1·20)	3·82 (1·50–5·70)	0·900 (0·500–2·10)	4·57 (1·60–6·40)
Western Europe	75 to 79	1990	0·712 (0·500–2·00)	4·37 (1·70–6·20)	0·910 (0·500–2·20)	4·93 (1·90–6·70)
Western Europe	80 plus	1990	0·765 (0·500–2·30)	4·91 (1·90–6·80)	0·929 (0·500–2·50)	5·30 (2·00–7·00)
Western Europe	15 to 19	2000	0·00330 (0–0)	0·00520 (0–0)	0·00180 (0–0)	0·00300 (0–0)

Western Europe	20 to 24	2000	0·0119 (0–0·200)	0·0203 (0–0·300)	0·00660 (0–0)	0·00980 (0–0)
Western Europe	25 to 29	2000	0·0366 (0–0·300)	0·0619 (0–0·600)	0·0155 (0–0·200)	0·0259 (0–0·400)
Western Europe	30 to 34	2000	0·0756 (0–0·400)	0·127 (0–0·700)	0·0608 (0–0·400)	0·109 (0–1·00)
Western Europe	35 to 39	2000	0·112 (0–0·400)	0·195 (0–0·800)	0·192 (0–0·600)	0·380 (0–1·10)
Western Europe	40 to 44	2000	0·166 (0–0·400)	0·303 (0–1·00)	0·326 (0–0·800)	0·726 (0–1·50)
Western Europe	45 to 49	2000	0·229 (0–0·500)	0·429 (0–1·00)	0·410 (0–1·00)	1·04 (0–2·00)
Western Europe	50 to 54	2000	0·299 (0–0·700)	0·590 (0–1·10)	0·484 (0·200–1·00)	1·37 (0·300–2·50)
Western Europe	55 to 59	2000	0·397 (0·100–0·900)	0·871 (0·100–1·50)	0·568 (0·300–1·00)	1·82 (0·600–3·30)
Western Europe	60 to 64	2000	0·522 (0·300–1·00)	1·41 (0·698–2·30)	0·656 (0·400–1·00)	2·37 (0·900–4·10)
Western Europe	65 to 69	2000	0·604 (0·400–1·00)	2·10 (1·00–3·50)	0·733 (0·400–1·60)	3·07 (1·10–5·00)
Western Europe	70 to 74	2000	0·639 (0·500–1·00)	2·95 (1·20–4·80)	0·764 (0·500–2·00)	3·83 (1·40–5·80)
Western Europe	75 to 79	2000	0·664 (0·500–1·00)	3·67 (1·50–5·60)	0·813 (0·500–2·00)	4·44 (1·70–6·20)
Western Europe	80 plus	2000	0·734 (0·500–2·00)	4·58 (1·70–6·40)	0·888 (0·500–2·30)	5·09 (2·00–6·80)
Western Europe	15 to 19	2010	0·00130 (0–0)	0·00220 (0–0)	0·000800 (0–0)	0·00150 (0–0)
Western Europe	20 to 24	2010	0·0148 (0–0·200)	0·0251 (0–0·400)	0·00630 (0–0)	0·00910 (0–0)
Western Europe	25 to 29	2010	0·0537 (0–0·400)	0·0906 (0–0·700)	0·0222 (0–0·300)	0·0367 (0–0·502)
Western Europe	30 to 34	2010	0·0900 (0–0·400)	0·153 (0–0·800)	0·0813 (0–0·400)	0·148 (0–1·00)
Western Europe	35 to 39	2010	0·113 (0–0·400)	0·193 (0–0·800)	0·205 (0–0·600)	0·408 (0–1·20)
Western Europe	40 to 44	2010	0·162 (0–0·500)	0·287 (0–1·00)	0·329 (0–0·800)	0·709 (0–1·50)
Western Europe	45 to 49	2010	0·232 (0–0·600)	0·425 (0–1·00)	0·413 (0–1·00)	0·966 (0–1·90)
Western Europe	50 to 54	2010	0·302 (0–0·700)	0·576 (0–1·10)	0·476 (0·200–1·00)	1·21 (0·300–2·20)
Western Europe	55 to 59	2010	0·384 (0–0·800)	0·785 (0–1·30)	0·533 (0·300–1·00)	1·47 (0·500–2·60)
Western Europe	60 to 64	2010	0·492 (0·300–0·900)	1·12 (0·400–1·80)	0·605 (0·400–1·00)	1·89 (0·800–3·30)
Western Europe	65 to 69	2010	0·568 (0·400–0·900)	1·54 (0·800–2·50)	0·663 (0·400–1·00)	2·43 (1·00–4·10)
Western Europe	70 to 74	2010	0·608 (0·400–0·900)	2·11 (1·10–3·40)	0·693 (0·500–1·00)	3·03 (1·20–4·90)
Western Europe	75 to 79	2010	0·622 (0·500–1·00)	2·69 (1·30–4·30)	0·712 (0·500–1·10)	3·64 (1·50–5·50)
Western Europe	80 plus	2010	0·663 (0·500–1·01)	3·71 (1·50–5·70)	0·765 (0·500–2·00)	4·46 (1·70–6·20)
Western Europe	15 to 19	2020	0·00240 (0–0)	0·00410 (0–0)	0·00130 (0–0)	0·00240 (0–0)
Western Europe	20 to 24	2020	0·0305 (0–0·302)	0·0510 (0–0·600)	0·0106 (0–0)	0·0155 (0–0)
Western Europe	25 to 29	2020	0·114 (0–0·502)	0·191 (0–1·00)	0·0381 (0–0·402)	0·0625 (0–0·800)
Western Europe	30 to 34	2020	0·157 (0–0·600)	0·265 (0–1·00)	0·124 (0–0·600)	0·222 (0–1·10)
Western Europe	35 to 39	2020	0·175 (0–0·600)	0·297 (0–1·00)	0·254 (0–0·700)	0·493 (0–1·30)
Western Europe	40 to 44	2020	0·207 (0–0·600)	0·358 (0–1·00)	0·358 (0–0·800)	0·764 (0–1·60)
Western Europe	45 to 49	2020	0·264 (0–0·700)	0·476 (0–1·10)	0·436 (0–0·900)	0·988 (0–1·90)
Western Europe	50 to 54	2020	0·339 (0–0·800)	0·642 (0–1·20)	0·502 (0·200–1·00)	1·23 (0·300–2·20)
Western Europe	55 to 59	2020	0·429 (0–0·800)	0·855 (0–1·40)	0·553 (0·300–1·00)	1·45 (0·500–2·50)
Western Europe	60 to 64	2020	0·524 (0·300–0·802)	1·16 (0·500–1·80)	0·611 (0·400–1·00)	1·83 (0·798–3·10)
Western Europe	65 to 69	2020	0·576 (0·400–0·900)	1·49 (0·800–2·30)	0·658 (0·400–1·00)	2·27 (1·00–3·70)
Western Europe	70 to 74	2020	0·602 (0·400–0·900)	1·94 (1·00–3·10)	0·680 (0·500–1·00)	2·85 (1·20–4·50)
Western Europe	75 to 79	2020	0·616 (0·500–0·900)	2·45 (1·20–3·90)	0·702 (0·500–1·00)	3·45 (1·40–5·30)
Western Europe	80 plus	2020	0·647 (0·500–1·00)	3·39 (1·40–5·30)	0·740 (0·500–1·90)	4·25 (1·60–6·10)

Andean Latin America	15 to 19	1990	0·318 (0–2·50)	0·562 (0–6·50)	0·0684 (0–0·202)	0·0869 (0–0·400)
Andean Latin America	20 to 24	1990	0·431 (0–3·00)	0·865 (0–10·0)	0·0888 (0–1·00)	0·118 (0–0·602)
Andean Latin America	25 to 29	1990	0·509 (0–3·00)	1·03 (0–10·0)	0·108 (0–1·00)	0·147 (0–0·902)
Andean Latin America	30 to 34	1990	0·476 (0–2·60)	1·02 (0–8·30)	0·168 (0–2·00)	0·282 (0–2·60)
Andean Latin America	35 to 39	1990	0·449 (0–2·30)	0·976 (0–5·60)	0·219 (0–2·00)	0·397 (0–2·50)
Andean Latin America	40 to 44	1990	0·465 (0·100–2·10)	1·04 (0·198–4·70)	0·292 (0–1·00)	0·584 (0–2·40)
Andean Latin America	45 to 49	1990	0·469 (0·200–2·00)	1·14 (0·300–3·20)	0·366 (0–1·00)	0·795 (0–2·40)
Andean Latin America	50 to 54	1990	0·499 (0·300–1·01)	1·30 (0·500–3·10)	0·440 (0·100–1·00)	1·02 (0·200–2·50)
Andean Latin America	55 to 59	1990	0·543 (0·300–1·00)	1·43 (0·700–3·00)	0·497 (0·200–1·00)	1·21 (0·400–3·00)
Andean Latin America	60 to 64	1990	0·594 (0·400–1·00)	1·70 (0·898–3·50)	0·543 (0·300–1·01)	1·48 (0·600–3·40)
Andean Latin America	65 to 69	1990	0·624 (0·400–1·00)	1·99 (1·00–4·20)	0·588 (0·400–2·00)	1·77 (0·800–4·00)
Andean Latin America	70 to 74	1990	0·645 (0·500–1·00)	2·31 (1·10–4·70)	0·623 (0·400–2·00)	2·08 (0·900–5·00)
Andean Latin America	75 to 79	1990	0·652 (0·500–1·01)	2·60 (1·20–5·10)	0·649 (0·400–2·00)	2·45 (1·00–5·50)
Andean Latin America	80 plus	1990	0·709 (0·500–1·60)	3·53 (1·40–6·00)	0·704 (0·500–2·00)	3·17 (1·20–6·10)
Andean Latin America	15 to 19	2000	0·0884 (0–0·300)	0·154 (0–0·602)	0·0102 (0–0·100)	0·0177 (0–0·200)
Andean Latin America	20 to 24	2000	0·193 (0–2·20)	0·333 (0–3·00)	0·0146 (0–0·102)	0·0217 (0–0·202)
Andean Latin America	25 to 29	2000	0·254 (0–2·10)	0·491 (0–3·10)	0·0266 (0–0·200)	0·0441 (0–0·400)
Andean Latin America	30 to 34	2000	0·273 (0–2·00)	0·541 (0–2·60)	0·0671 (0–0·300)	0·116 (0–0·600)
Andean Latin America	35 to 39	2000	0·302 (0–0·600)	0·622 (0–2·10)	0·130 (0–0·400)	0·231 (0–1·00)
Andean Latin America	40 to 44	2000	0·366 (0–0·700)	0·798 (0–2·00)	0·224 (0–0·500)	0·437 (0–1·10)
Andean Latin America	45 to 49	2000	0·427 (0·200–0·700)	1·01 (0·398–2·00)	0·326 (0–0·802)	0·691 (0–1·50)
Andean Latin America	50 to 54	2000	0·494 (0·300–0·800)	1·21 (0·600–2·20)	0·421 (0·100–1·00)	0·943 (0·200–2·00)
Andean Latin America	55 to 59	2000	0·556 (0·400–0·900)	1·38 (0·800–2·30)	0·486 (0·200–1·00)	1·14 (0·400–2·20)
Andean Latin America	60 to 64	2000	0·616 (0·400–0·902)	1·65 (1·00–2·60)	0·544 (0·300–1·00)	1·38 (0·600–2·50)
Andean Latin America	65 to 69	2000	0·641 (0·400–1·00)	1·87 (1·10–3·00)	0·580 (0·400–1·00)	1·60 (0·800–2·80)
Andean Latin America	70 to 74	2000	0·652 (0·500–1·00)	2·15 (1·20–3·50)	0·604 (0·400–1·00)	1·88 (1·00–3·30)
Andean Latin America	75 to 79	2000	0·655 (0·500–1·00)	2·41 (1·30–4·00)	0·627 (0·500–1·00)	2·25 (1·10–4·20)
Andean Latin America	80 plus	2000	0·695 (0·500–1·00)	3·29 (1·50–5·20)	0·678 (0·500–1·50)	3·00 (1·30–5·20)
Andean Latin America	15 to 19	2010	0·0549 (0–0·300)	0·0932 (0–0·600)	0·0114 (0–0·200)	0·0197 (0–0·300)
Andean Latin America	20 to 24	2010	0·0870 (0–0·300)	0·151 (0–0·700)	0·0102 (0–0·200)	0·0184 (0–0·300)
Andean Latin America	25 to 29	2010	0·155 (0–0·400)	0·278 (0–0·900)	0·0197 (0–0·200)	0·0333 (0–0·400)
Andean Latin America	30 to 34	2010	0·216 (0–0·400)	0·408 (0–1·00)	0·0533 (0–0·300)	0·0912 (0–0·600)
Andean Latin America	35 to 39	2010	0·267 (0–0·500)	0·535 (0–1·10)	0·111 (0–0·400)	0·201 (0–1·00)
Andean Latin America	40 to 44	2010	0·339 (0–0·600)	0·719 (0–1·30)	0·210 (0–0·500)	0·413 (0–1·10)
Andean Latin America	45 to 49	2010	0·438 (0·200–0·700)	0·998 (0·400–1·60)	0·327 (0–0·700)	0·682 (0–1·30)
Andean Latin America	50 to 54	2010	0·528 (0·300–0·800)	1·23 (0·700–2·00)	0·435 (0·100–1·00)	0·953 (0·200–1·70)
Andean Latin America	55 to 59	2010	0·597 (0·400–0·900)	1·40 (0·800–2·20)	0·518 (0·300–1·00)	1·18 (0·500–2·10)
Andean Latin America	60 to 64	2010	0·646 (0·400–1·00)	1·63 (1·00–2·40)	0·572 (0·400–1·00)	1·41 (0·700–2·30)
Andean Latin America	65 to 69	2010	0·673 (0·500–1·00)	1·81 (1·10–2·60)	0·610 (0·400–1·00)	1·61 (0·900–2·60)
Andean Latin America	70 to 74	2010	0·680 (0·500–1·00)	2·04 (1·20–3·00)	0·637 (0·400–1·00)	1·86 (1·00–3·00)
Andean Latin America	75 to 79	2010	0·680 (0·500–1·00)	2·26 (1·30–3·40)	0·651 (0·500–1·00)	2·17 (1·10–3·60)

Andean Latin America	80 plus	2010	0·708 (0·500–1·00)	3·03 (1·50–4·80)	0·689 (0·500–1·00)	2·83 (1·30–4·70)
Andean Latin America	15 to 19	2020	0·0616 (0–0·300)	0·107 (0–0·700)	0·0139 (0–0·200)	0·0232 (0–0·300)
Andean Latin America	20 to 24	2020	0·0897 (0–0·300)	0·155 (0–0·602)	0·0133 (0–0·200)	0·0232 (0–0·400)
Andean Latin America	25 to 29	2020	0·170 (0–0·400)	0·309 (0–1·00)	0·0229 (0–0·200)	0·0408 (0–0·400)
Andean Latin America	30 to 34	2020	0·235 (0–0·400)	0·451 (0–1·00)	0·0721 (0–0·300)	0·127 (0–0·700)
Andean Latin America	35 to 39	2020	0·301 (0–0·500)	0·613 (0–1·10)	0·146 (0–0·400)	0·273 (0–1·00)
Andean Latin America	40 to 44	2020	0·380 (0·100–0·700)	0·824 (0·100–1·40)	0·259 (0–0·600)	0·523 (0–1·20)
Andean Latin America	45 to 49	2020	0·487 (0·300–0·800)	1·10 (0·498–1·80)	0·385 (0–0·800)	0·810 (0–1·50)
Andean Latin America	50 to 54	2020	0·576 (0·300–0·900)	1·33 (0·700–2·10)	0·490 (0·200–1·00)	1·08 (0·300–2·00)
Andean Latin America	55 to 59	2020	0·644 (0·400–0·902)	1·52 (0·900–2·30)	0·582 (0·300–1·00)	1·34 (0·600–2·20)
Andean Latin America	60 to 64	2020	0·691 (0·500–1·00)	1·77 (1·10–2·50)	0·633 (0·400–1·00)	1·58 (0·800–2·50)
Andean Latin America	65 to 69	2020	0·712 (0·500–1·00)	1·95 (1·20–2·80)	0·674 (0·400–1·00)	1·81 (1·00–2·90)
Andean Latin America	70 to 74	2020	0·716 (0·500–1·00)	2·19 (1·30–3·20)	0·692 (0·500–1·00)	2·07 (1·10–3·30)
Andean Latin America	75 to 79	2020	0·715 (0·500–1·00)	2·45 (1·40–3·70)	0·696 (0·500–1·00)	2·39 (1·30–4·00)
Andean Latin America	80 plus	2020	0·736 (0·500–1·00)	3·25 (1·60–5·00)	0·724 (0·500–1·00)	3·10 (1·40–5·00)
Caribbean	15 to 19	1990	0·0427 (0–0·300)	0·0729 (0–0·600)	0·00290 (0–0)	0·00520 (0–0)
Caribbean	20 to 24	1990	0·0990 (0–0·400)	0·171 (0–1·00)	0·00580 (0–0·00250)	0·0106 (0–0·00250)
Caribbean	25 to 29	1990	0·206 (0–0·500)	0·388 (0–1·10)	0·0237 (0–0·300)	0·0411 (0–0·500)
Caribbean	30 to 34	1990	0·333 (0–0·600)	0·715 (0–1·50)	0·109 (0–0·400)	0·195 (0–0·900)
Caribbean	35 to 39	1990	0·414 (0·200–0·600)	1·02 (0·300–2·10)	0·254 (0–0·500)	0·532 (0–1·20)
Caribbean	40 to 44	1990	0·498 (0·300–0·800)	1·43 (0·700–2·50)	0·394 (0·100–0·700)	0·973 (0·200–1·90)
Caribbean	45 to 49	1990	0·584 (0·400–0·900)	1·90 (1·00–3·00)	0·499 (0·300–1·00)	1·46 (0·500–2·60)
Caribbean	50 to 54	1990	0·658 (0·500–1·00)	2·30 (1·30–3·60)	0·601 (0·400–1·00)	1·97 (0·900–3·40)
Caribbean	55 to 59	1990	0·716 (0·500–1·00)	2·73 (1·50–4·20)	0·666 (0·400–1·00)	2·37 (1·10–4·10)
Caribbean	60 to 64	1990	0·759 (0·500–1·00)	3·27 (1·80–5·00)	0·718 (0·500–1·00)	2·87 (1·30–4·80)
Caribbean	65 to 69	1990	0·793 (0·600–1·00)	3·74 (2·00–5·50)	0·771 (0·500–1·50)	3·40 (1·50–5·40)
Caribbean	70 to 74	1990	0·780 (0·600–1·50)	4·16 (2·10–5·90)	0·785 (0·500–1·90)	3·95 (1·70–6·00)
Caribbean	75 to 79	1990	0·813 (0·600–2·00)	4·63 (2·20–6·30)	0·847 (0·500–2·00)	4·48 (1·90–6·30)
Caribbean	80 plus	1990	0·885 (0·600–2·20)	5·20 (2·20–6·80)	0·993 (0·600–2·30)	5·20 (2·00–6·90)
Caribbean	15 to 19	2000	0·0468 (0–0·300)	0·0815 (0–0·600)	0·00480 (0–0)	0·00760 (0–0)
Caribbean	20 to 24	2000	0·114 (0–0·400)	0·200 (0–0·900)	0·00800 (0–0)	0·0138 (0–0)
Caribbean	25 to 29	2000	0·223 (0–0·500)	0·424 (0–1·10)	0·0214 (0–0·300)	0·0363 (0–0·500)
Caribbean	30 to 34	2000	0·333 (0–0·600)	0·707 (0–1·30)	0·102 (0–0·400)	0·181 (0–0·900)
Caribbean	35 to 39	2000	0·418 (0·200–0·700)	0·988 (0·400–1·70)	0·256 (0–0·500)	0·526 (0–1·20)
Caribbean	40 to 44	2000	0·516 (0·300–0·800)	1·38 (0·700–2·20)	0·401 (0·100–0·700)	0·968 (0·198–1·80)
Caribbean	45 to 49	2000	0·606 (0·400–0·900)	1·86 (1·10–2·80)	0·512 (0·300–1·00)	1·41 (0·600–2·40)
Caribbean	50 to 54	2000	0·678 (0·500–1·00)	2·25 (1·30–3·40)	0·620 (0·400–1·00)	1·93 (0·900–3·20)
Caribbean	55 to 59	2000	0·737 (0·500–1·00)	2·70 (1·60–4·10)	0·696 (0·497–1·00)	2·38 (1·10–3·90)
Caribbean	60 to 64	2000	0·770 (0·600–1·00)	3·22 (1·80–4·80)	0·734 (0·500–1·00)	2·82 (1·30–4·60)
Caribbean	65 to 69	2000	0·790 (0·600–1·00)	3·62 (2·10–5·30)	0·763 (0·500–1·00)	3·29 (1·60–5·10)
Caribbean	70 to 74	2000	0·767 (0·600–1·00)	3·98 (2·10–5·60)	0·768 (0·500–1·50)	3·77 (1·80–5·60)

Caribbean	75 to 79	2000	0·768 (0·600–1·10)	4·33 (2·20–6·00)	0·791 (0·500–1·90)	4·20 (2·00–6·00)
Caribbean	80 plus	2000	0·804 (0·600–2·00)	4·85 (2·10–6·50)	0·862 (0·600–2·00)	4·83 (2·00–6·50)
Caribbean	15 to 19	2010	0·0512 (0–0·300)	0·0895 (0–0·700)	0·00850 (0–0)	0·0122 (0–0)
Caribbean	20 to 24	2010	0·124 (0–0·400)	0·220 (0–1·00)	0·0135 (0–0·102)	0·0190 (0–0·202)
Caribbean	25 to 29	2010	0·233 (0–0·500)	0·450 (0–1·10)	0·0249 (0–0·300)	0·0389 (0–0·500)
Caribbean	30 to 34	2010	0·350 (0–0·600)	0·748 (0–1·40)	0·0807 (0–0·400)	0·146 (0–1·00)
Caribbean	35 to 39	2010	0·436 (0·200–0·700)	1·02 (0·400–1·70)	0·235 (0–0·500)	0·471 (0–1·20)
Caribbean	40 to 44	2010	0·526 (0·300–0·800)	1·36 (0·700–2·20)	0·389 (0·100–0·700)	0·915 (0·100–1·70)
Caribbean	45 to 49	2010	0·615 (0·400–0·900)	1·82 (1·10–2·70)	0·510 (0·300–0·900)	1·36 (0·598–2·30)
Caribbean	50 to 54	2010	0·684 (0·500–1·00)	2·21 (1·30–3·30)	0·613 (0·400–1·00)	1·84 (0·900–3·00)
Caribbean	55 to 59	2010	0·732 (0·500–1·00)	2·58 (1·50–3·90)	0·678 (0·400–1·00)	2·23 (1·10–3·60)
Caribbean	60 to 64	2010	0·768 (0·600–1·00)	3·11 (1·80–4·60)	0·721 (0·500–1·00)	2·71 (1·30–4·30)
Caribbean	65 to 69	2010	0·783 (0·600–1·00)	3·45 (2·00–5·10)	0·753 (0·500–1·00)	3·17 (1·50–4·90)
Caribbean	70 to 74	2010	0·756 (0·600–1·00)	3·82 (2·10–5·40)	0·754 (0·500–1·00)	3·61 (1·80–5·40)
Caribbean	75 to 79	2010	0·750 (0·600–1·00)	4·08 (2·20–5·80)	0·761 (0·597–1·50)	3·95 (1·90–5·70)
Caribbean	80 plus	2010	0·777 (0·600–1·90)	4·61 (2·00–6·40)	0·802 (0·600–2·00)	4·50 (2·00–6·20)
Caribbean	15 to 19	2020	0·0764 (0–0·400)	0·137 (0–0·800)	0·0100 (0–0)	0·0130 (0–0)
Caribbean	20 to 24	2020	0·175 (0–0·500)	0·318 (0–1·00)	0·0164 (0–0·200)	0·0238 (0–0·400)
Caribbean	25 to 29	2020	0·280 (0–0·600)	0·555 (0–1·20)	0·0321 (0–0·300)	0·0521 (0–0·700)
Caribbean	30 to 34	2020	0·389 (0·100–0·700)	0·853 (0·100–1·60)	0·105 (0–0·400)	0·189 (0–1·00)
Caribbean	35 to 39	2020	0·481 (0·300–0·800)	1·16 (0·500–2·00)	0·263 (0–0·600)	0·541 (0–1·20)
Caribbean	40 to 44	2020	0·570 (0·400–0·800)	1·53 (0·800–2·40)	0·422 (0·100–0·800)	1·01 (0·200–1·90)
Caribbean	45 to 49	2020	0·643 (0·400–0·902)	1·92 (1·10–3·00)	0·541 (0·300–1·00)	1·45 (0·600–2·40)
Caribbean	50 to 54	2020	0·704 (0·500–1·00)	2·29 (1·40–3·40)	0·637 (0·400–1·00)	1·89 (0·900–3·00)
Caribbean	55 to 59	2020	0·750 (0·500–1·00)	2·65 (1·60–3·90)	0·697 (0·500–1·00)	2·26 (1·10–3·70)
Caribbean	60 to 64	2020	0·786 (0·600–1·00)	3·19 (1·90–4·70)	0·733 (0·500–1·00)	2·74 (1·30–4·30)
Caribbean	65 to 69	2020	0·798 (0·600–1·00)	3·52 (2·10–5·10)	0·770 (0·500–1·00)	3·21 (1·60–5·00)
Caribbean	70 to 74	2020	0·774 (0·600–1·00)	3·83 (2·10–5·40)	0·774 (0·600–1·00)	3·62 (1·80–5·40)
Caribbean	75 to 79	2020	0·764 (0·600–1·00)	4·10 (2·20–5·70)	0·778 (0·600–1·50)	3·94 (1·90–5·70)
Caribbean	80 plus	2020	0·775 (0·600–1·51)	4·52 (2·00–6·20)	0·806 (0·600–2·00)	4·44 (1·90–6·20)
Central Latin America	15 to 19	1990	0·0274 (0–0·202)	0·0465 (0–0·400)	0·0105 (0–0)	0·0143 (0–0)
Central Latin America	20 to 24	1990	0·0707 (0–0·300)	0·120 (0–0·602)	0·0185 (0–0·100)	0·0241 (0–0·200)
Central Latin America	25 to 29	1990	0·157 (0–0·400)	0·289 (0–1·00)	0·0224 (0–0·300)	0·0311 (0–0·502)
Central Latin America	30 to 34	1990	0·269 (0–0·500)	0·535 (0–1·10)	0·0347 (0–0·400)	0·0562 (0–0·902)
Central Latin America	35 to 39	1990	0·364 (0·100–0·600)	0·786 (0·100–1·40)	0·0907 (0–0·600)	0·156 (0–1·00)
Central Latin America	40 to 44	1990	0·477 (0·300–0·800)	1·11 (0·500–2·00)	0·229 (0–0·800)	0·438 (0–1·20)
Central Latin America	45 to 49	1990	0·587 (0·400–0·900)	1·46 (0·800–2·30)	0·385 (0–1·00)	0·769 (0–1·50)
Central Latin America	50 to 54	1990	0·682 (0·400–1·00)	1·85 (1·10–2·80)	0·523 (0·200–1·00)	1·14 (0·300–2·10)
Central Latin America	55 to 59	1990	0·749 (0·500–1·00)	2·21 (1·30–3·30)	0·616 (0·300–1·00)	1·49 (0·600–2·50)
Central Latin America	60 to 64	1990	0·781 (0·600–1·00)	2·66 (1·50–4·00)	0·685 (0·400–1·00)	1·94 (0·998–3·30)
Central Latin America	65 to 69	1990	0·800 (0·600–1·00)	3·03 (1·70–4·60)	0·731 (0·500–1·00)	2·35 (1·10–3·90)

Central Latin America	70 to 74	1990	0·789 (0·600–1·00)	3·50 (1·90–5·20)	0·741 (0·500–1·00)	2·85 (1·30–4·70)
Central Latin America	75 to 79	1990	0·788 (0·600–1·00)	3·85 (2·00–5·60)	0·754 (0·500–1·50)	3·32 (1·50–5·40)
Central Latin America	80 plus	1990	0·807 (0·600–2·00)	4·53 (2·00–6·30)	0·771 (0·500–1·90)	3·95 (1·60–6·00)
Central Latin America	15 to 19	2000	0·0161 (0–0·200)	0·0278 (0–0·400)	0·0210 (0–0·200)	0·0265 (0–0·300)
Central Latin America	20 to 24	2000	0·0493 (0–0·300)	0·0850 (0–0·700)	0·0249 (0–0·400)	0·0319 (0–1·00)
Central Latin America	25 to 29	2000	0·127 (0–0·402)	0·224 (0–1·00)	0·0277 (0–0·400)	0·0390 (0–1·00)
Central Latin America	30 to 34	2000	0·242 (0–0·600)	0·464 (0–1·10)	0·0422 (0–0·400)	0·0676 (0–1·00)
Central Latin America	35 to 39	2000	0·361 (0–0·700)	0·739 (0–1·30)	0·101 (0–0·800)	0·173 (0–1·10)
Central Latin America	40 to 44	2000	0·499 (0·300–0·800)	1·10 (0·400–1·70)	0·254 (0–1·00)	0·474 (0–1·20)
Central Latin America	45 to 49	2000	0·621 (0·400–0·900)	1·48 (0·900–2·20)	0·429 (0–1·00)	0·840 (0–1·60)
Central Latin America	50 to 54	2000	0·715 (0·500–1·00)	1·87 (1·20–2·70)	0·568 (0·200–1·00)	1·21 (0·398–2·10)
Central Latin America	55 to 59	2000	0·781 (0·500–1·00)	2·23 (1·30–3·20)	0·666 (0·300–1·00)	1·57 (0·700–2·50)
Central Latin America	60 to 64	2000	0·818 (0·600–1·00)	2·68 (1·60–3·80)	0·739 (0·400–1·00)	2·02 (1·00–3·20)
Central Latin America	65 to 69	2000	0·835 (0·600–1·00)	3·04 (1·80–4·30)	0·784 (0·500–1·00)	2·49 (1·20–4·00)
Central Latin America	70 to 74	2000	0·824 (0·600–1·00)	3·48 (2·00–5·00)	0·790 (0·500–1·00)	2·99 (1·40–4·70)
Central Latin America	75 to 79	2000	0·820 (0·600–1·00)	3·85 (2·10–5·40)	0·794 (0·600–1·00)	3·43 (1·70–5·20)
Central Latin America	80 plus	2000	0·852 (0·600–2·00)	4·66 (2·20–6·30)	0·832 (0·600–2·00)	4·24 (1·80–6·10)
Central Latin America	15 to 19	2010	0·0138 (0–0·200)	0·0238 (0–0·400)	0·0202 (0–0·200)	0·0254 (0–0·300)
Central Latin America	20 to 24	2010	0·0447 (0–0·400)	0·0768 (0–0·700)	0·0246 (0–0·400)	0·0323 (0–0·805)
Central Latin America	25 to 29	2010	0·128 (0–0·500)	0·225 (0–1·00)	0·0321 (0–0·600)	0·0445 (0–1·00)
Central Latin America	30 to 34	2010	0·265 (0–0·600)	0·502 (0–1·10)	0·0495 (0–0·600)	0·0800 (0–1·00)
Central Latin America	35 to 39	2010	0·409 (0–0·700)	0·814 (0–1·30)	0·118 (0–0·800)	0·203 (0–1·10)
Central Latin America	40 to 44	2010	0·555 (0·300–0·800)	1·18 (0·500–1·90)	0·278 (0–0·900)	0·521 (0–1·30)
Central Latin America	45 to 49	2010	0·669 (0·400–0·900)	1·58 (0·900–2·40)	0·473 (0–1·00)	0·931 (0–1·70)
Central Latin America	50 to 54	2010	0·748 (0·500–1·00)	1·96 (1·20–2·80)	0·623 (0·300–1·00)	1·34 (0·500–2·20)
Central Latin America	55 to 59	2010	0·797 (0·600–1·00)	2·27 (1·40–3·20)	0·719 (0·400–1·00)	1·73 (0·900–2·80)
Central Latin America	60 to 64	2010	0·831 (0·600–1·00)	2·65 (1·60–3·70)	0·781 (0·500–1·00)	2·18 (1·10–3·40)
Central Latin America	65 to 69	2010	0·847 (0·600–1·00)	2·95 (1·80–4·20)	0·824 (0·500–1·00)	2·63 (1·30–4·10)
Central Latin America	70 to 74	2010	0·840 (0·600–1·00)	3·39 (2·00–4·90)	0·832 (0·600–1·00)	3·11 (1·50–4·80)
Central Latin America	75 to 79	2010	0·841 (0·600–1·00)	3·80 (2·10–5·40)	0·842 (0·600–1·21)	3·58 (1·70–5·30)
Central Latin America	80 plus	2010	0·904 (0·600–2·00)	4·79 (2·20–6·40)	0·909 (0·600–2·00)	4·49 (1·90–6·20)
Central Latin America	15 to 19	2020	0·0208 (0–0·300)	0·0352 (0–0·500)	0·0195 (0–0·200)	0·0249 (0–0·300)
Central Latin America	20 to 24	2020	0·0664 (0–0·400)	0·117 (0–0·900)	0·0267 (0–0·400)	0·0339 (0–1·00)
Central Latin America	25 to 29	2020	0·180 (0–0·600)	0·325 (0–1·10)	0·0352 (0–0·502)	0·0501 (0–1·00)
Central Latin America	30 to 34	2020	0·330 (0–0·700)	0·635 (0–1·20)	0·0638 (0–0·700)	0·105 (0–1·00)
Central Latin America	35 to 39	2020	0·470 (0·100–0·800)	0·935 (0·200–1·50)	0·160 (0–0·802)	0·283 (0–1·10)
Central Latin America	40 to 44	2020	0·610 (0·300–0·900)	1·31 (0·700–2·10)	0·341 (0–1·00)	0·648 (0–1·30)
Central Latin America	45 to 49	2020	0·705 (0·400–0·900)	1·69 (1·00–2·50)	0·531 (0–1·00)	1·05 (0–2·00)
Central Latin America	50 to 54	2020	0·770 (0·500–1·00)	2·04 (1·20–2·90)	0·674 (0·300–1·00)	1·49 (0·600–2·40)
Central Latin America	55 to 59	2020	0·814 (0·600–1·00)	2·35 (1·40–3·30)	0·768 (0·400–1·00)	1·92 (1·00–3·00)
Central Latin America	60 to 64	2020	0·841 (0·600–1·00)	2·70 (1·60–3·70)	0·825 (0·500–1·00)	2·42 (1·20–3·70)

Central Latin America	65 to 69	2020	0·863 (0·700–1·00)	3·01 (1·80–4·20)	0·864 (0·600–1·00)	2·93 (1·50–4·50)
Central Latin America	70 to 74	2020	0·857 (0·600–1·00)	3·44 (2·10–4·90)	0·870 (0·600–1·00)	3·42 (1·70–5·10)
Central Latin America	75 to 79	2020	0·862 (0·600–1·00)	3·89 (2·30–5·40)	0·888 (0·600–1·80)	3·85 (1·90–5·50)
Central Latin America	80 plus	2020	0·953 (0·600–2·00)	4·92 (2·30–6·60)	0·983 (0·600–2·00)	4·75 (2·10–6·40)
Tropical Latin America	15 to 19	1990	0·0210 (0–0·200)	0·0358 (0–0·400)	0·0131 (0–0)	0·0163 (0–0)
Tropical Latin America	20 to 24	1990	0·0651 (0–0·300)	0·115 (0–0·700)	0·0153 (0–0·100)	0·0209 (0–0·102)
Tropical Latin America	25 to 29	1990	0·200 (0–0·400)	0·383 (0–1·00)	0·0211 (0–0·202)	0·0331 (0–0·402)
Tropical Latin America	30 to 34	1990	0·312 (0–0·500)	0·681 (0–1·20)	0·0752 (0–0·400)	0·134 (0–1·00)
Tropical Latin America	35 to 39	1990	0·387 (0·200–0·500)	0·991 (0·300–1·70)	0·209 (0–0·500)	0·417 (0–1·10)
Tropical Latin America	40 to 44	1990	0·452 (0·300–0·600)	1·36 (0·600–2·10)	0·331 (0–0·502)	0·762 (0–1·40)
Tropical Latin America	45 to 49	1990	0·524 (0·400–0·800)	1·84 (1·00–2·80)	0·422 (0·200–0·700)	1·15 (0·400–2·00)
Tropical Latin America	50 to 54	1990	0·586 (0·500–0·900)	2·20 (1·30–3·30)	0·487 (0·300–0·900)	1·58 (0·700–2·60)
Tropical Latin America	55 to 59	1990	0·641 (0·500–1·00)	2·61 (1·50–4·10)	0·541 (0·400–1·00)	2·02 (1·00–3·40)
Tropical Latin America	60 to 64	1990	0·679 (0·500–1·00)	3·20 (1·80–5·00)	0·597 (0·500–1·00)	2·59 (1·30–4·30)
Tropical Latin America	65 to 69	1990	0·700 (0·500–1·00)	3·55 (2·00–5·30)	0·627 (0·500–1·00)	3·06 (1·50–5·00)
Tropical Latin America	70 to 74	1990	0·693 (0·500–1·00)	3·91 (2·10–5·60)	0·649 (0·500–1·00)	3·50 (1·70–5·40)
Tropical Latin America	75 to 79	1990	0·692 (0·500–1·00)	4·11 (2·20–5·80)	0·659 (0·500–1·50)	3·80 (1·90–5·70)
Tropical Latin America	80 plus	1990	0·705 (0·500–1·50)	4·45 (2·10–6·20)	0·685 (0·500–1·70)	4·20 (1·90–6·10)
Tropical Latin America	15 to 19	2000	0·0149 (0–0·200)	0·0255 (0–0·300)	0·0228 (0–0·200)	0·0278 (0–0·302)
Tropical Latin America	20 to 24	2000	0·0379 (0–0·300)	0·0665 (0–0·600)	0·0245 (0–0·302)	0·0301 (0–0·707)
Tropical Latin America	25 to 29	2000	0·118 (0–0·400)	0·212 (0–0·800)	0·0235 (0–0·300)	0·0304 (0–0·600)
Tropical Latin America	30 to 34	2000	0·258 (0–0·400)	0·518 (0–1·10)	0·0395 (0–0·300)	0·0647 (0–0·702)
Tropical Latin America	35 to 39	2000	0·355 (0·0975–0·500)	0·824 (0·0975–1·40)	0·134 (0–0·402)	0·245 (0–1·00)
Tropical Latin America	40 to 44	2000	0·444 (0·300–0·700)	1·24 (0·500–2·00)	0·286 (0–0·502)	0·607 (0–1·30)
Tropical Latin America	45 to 49	2000	0·530 (0·400–0·800)	1·68 (0·900–2·50)	0·397 (0·198–0·700)	0·984 (0·200–1·80)
Tropical Latin America	50 to 54	2000	0·603 (0·400–0·900)	2·05 (1·10–3·10)	0·477 (0·300–1·00)	1·38 (0·600–2·30)
Tropical Latin America	55 to 59	2000	0·664 (0·500–1·00)	2·44 (1·40–3·80)	0·537 (0·400–1·00)	1·79 (0·900–3·00)
Tropical Latin America	60 to 64	2000	0·697 (0·500–1·00)	2·96 (1·70–4·50)	0·594 (0·400–1·00)	2·30 (1·20–3·80)
Tropical Latin America	65 to 69	2000	0·716 (0·500–1·00)	3·26 (1·90–4·90)	0·625 (0·500–1·00)	2·75 (1·40–4·40)
Tropical Latin America	70 to 74	2000	0·699 (0·500–1·00)	3·56 (2·00–5·20)	0·646 (0·500–1·00)	3·16 (1·60–4·90)
Tropical Latin America	75 to 79	2000	0·694 (0·500–1·00)	3·71 (2·00–5·30)	0·656 (0·500–1·00)	3·47 (1·80–5·30)
Tropical Latin America	80 plus	2000	0·687 (0·500–1·00)	3·89 (1·90–5·60)	0·665 (0·500–1·00)	3·69 (1·80–5·50)
Tropical Latin America	15 to 19	2010	0·0126 (0–0·200)	0·0220 (0–0·300)	0·0266 (0–0·415)	0·0319 (0–1·00)
Tropical Latin America	20 to 24	2010	0·0274 (0–0·300)	0·0480 (0–0·500)	0·0244 (0–0·300)	0·0287 (0–0·505)
Tropical Latin America	25 to 29	2010	0·0830 (0–0·400)	0·147 (0–0·800)	0·0226 (0–0·300)	0·0302 (0–0·700)
Tropical Latin America	30 to 34	2010	0·216 (0–0·500)	0·416 (0–1·00)	0·0334 (0–0·300)	0·0526 (0–0·602)
Tropical Latin America	35 to 39	2010	0·338 (0–0·600)	0·738 (0–1·30)	0·104 (0–0·402)	0·188 (0–1·00)
Tropical Latin America	40 to 44	2010	0·449 (0·200–0·800)	1·13 (0·400–1·80)	0·252 (0–0·600)	0·517 (0–1·20)
Tropical Latin America	45 to 49	2010	0·546 (0·400–0·900)	1·57 (0·800–2·40)	0·381 (0·0975–0·700)	0·887 (0·0975–1·60)
Tropical Latin America	50 to 54	2010	0·629 (0·400–0·900)	1·96 (1·10–3·00)	0·479 (0·300–1·00)	1·27 (0·500–2·10)
Tropical Latin America	55 to 59	2010	0·690 (0·500–1·00)	2·31 (1·30–3·50)	0·553 (0·400–1·00)	1·66 (0·800–2·70)

Tropical Latin America	60 to 64	2010	0·719 (0·500–1·00)	2·75 (1·60–4·10)	0·614 (0·400–1·00)	2·12 (1·10–3·40)
Tropical Latin America	65 to 69	2010	0·737 (0·500–1·00)	3·07 (1·80–4·50)	0·652 (0·500–1·00)	2·58 (1·30–4·10)
Tropical Latin America	70 to 74	2010	0·712 (0·500–1·00)	3·34 (1·90–4·90)	0·662 (0·500–1·00)	2·99 (1·50–4·60)
Tropical Latin America	75 to 79	2010	0·703 (0·500–1·00)	3·48 (2·00–5·00)	0·667 (0·500–1·00)	3·32 (1·70–5·00)
Tropical Latin America	80 plus	2010	0·682 (0·500–1·00)	3·57 (1·90–5·20)	0·675 (0·500–1·00)	3·51 (1·80–5·20)
Tropical Latin America	15 to 19	2020	0·0168 (0–0·200)	0·0284 (0–0·400)	0·0352 (0–1·00)	0·0424 (0–1·00)
Tropical Latin America	20 to 24	2020	0·0371 (0–0·300)	0·0641 (0–0·602)	0·0306 (0–1·00)	0·0387 (0–1·00)
Tropical Latin America	25 to 29	2020	0·0960 (0–0·400)	0·170 (0–0·900)	0·0266 (0–0·400)	0·0352 (0–0·802)
Tropical Latin America	30 to 34	2020	0·227 (0–0·500)	0·431 (0–1·10)	0·0380 (0–0·400)	0·0617 (0–1·00)
Tropical Latin America	35 to 39	2020	0·352 (0–0·600)	0·761 (0–1·30)	0·103 (0–0·402)	0·188 (0–1·00)
Tropical Latin America	40 to 44	2020	0·469 (0·300–0·800)	1·12 (0·400–1·70)	0·250 (0–0·600)	0·512 (0–1·20)
Tropical Latin America	45 to 49	2020	0·568 (0·400–0·900)	1·55 (0·898–2·30)	0·382 (0–0·700)	0·882 (0–1·60)
Tropical Latin America	50 to 54	2020	0·642 (0·400–0·900)	1·89 (1·10–2·80)	0·483 (0·300–0·900)	1·25 (0·500–2·10)
Tropical Latin America	55 to 59	2020	0·709 (0·500–1·00)	2·28 (1·30–3·40)	0·563 (0·400–1·00)	1·63 (0·800–2·60)
Tropical Latin America	60 to 64	2020	0·737 (0·500–1·00)	2·75 (1·60–4·10)	0·628 (0·400–1·00)	2·12 (1·10–3·40)
Tropical Latin America	65 to 69	2020	0·755 (0·500–1·00)	3·05 (1·80–4·40)	0·669 (0·500–1·00)	2·57 (1·30–4·10)
Tropical Latin America	70 to 74	2020	0·729 (0·600–1·00)	3·30 (1·90–4·70)	0·681 (0·500–1·00)	2·98 (1·50–4·50)
Tropical Latin America	75 to 79	2020	0·714 (0·600–1·00)	3·44 (2·00–4·90)	0·683 (0·500–1·00)	3·30 (1·80–4·90)
Tropical Latin America	80 plus	2020	0·686 (0·500–1·00)	3·44 (1·80–5·10)	0·682 (0·500–1·00)	3·49 (1·80–5·10)
North Africa and Middle East	15 to 19	1990	0·250 (0–0·400)	0·505 (0–1·10)	0·0583 (0–0·300)	0·108 (0–0·700)
North Africa and Middle East	20 to 24	1990	0·265 (0–0·400)	0·543 (0–1·20)	0·0534 (0–0·300)	0·0972 (0–0·700)
North Africa and Middle East	25 to 29	1990	0·312 (0–0·500)	0·699 (0–1·60)	0·150 (0–0·400)	0·292 (0–1·10)
North Africa and Middle East	30 to 34	1990	0·381 (0·200–0·500)	1·04 (0·300–2·40)	0·254 (0–0·500)	0·562 (0–1·50)
North Africa and Middle East	35 to 39	1990	0·425 (0·300–0·600)	1·32 (0·500–2·80)	0·352 (0–0·600)	0·994 (0–2·40)
North Africa and Middle East	40 to 44	1990	0·482 (0·300–0·802)	1·74 (0·700–3·30)	0·473 (0·200–1·00)	1·80 (0·400–4·00)
North Africa and Middle East	45 to 49	1990	0·573 (0·400–1·00)	2·39 (1·00–4·50)	0·627 (0·300–1·90)	2·65 (0·700–5·10)
North Africa and Middle East	50 to 54	1990	0·647 (0·400–1·00)	2·69 (1·20–4·80)	0·773 (0·400–2·00)	3·24 (1·00–5·50)
North Africa and Middle East	55 to 59	1990	0·707 (0·500–1·50)	3·06 (1·30–5·10)	0·863 (0·400–2·00)	3·76 (1·20–6·00)
North Africa and Middle East	60 to 64	1990	0·755 (0·500–1·50)	3·50 (1·50–5·60)	0·841 (0·500–2·00)	3·83 (1·30–6·00)
North Africa and Middle East	65 to 69	1990	0·784 (0·500–1·90)	3·73 (1·60–5·90)	0·857 (0·500–2·00)	3·99 (1·40–6·10)
North Africa and Middle East	70 to 74	1990	0·762 (0·500–1·90)	3·65 (1·50–5·80)	0·796 (0·500–2·00)	3·88 (1·50–6·00)
North Africa and Middle East	75 to 79	1990	0·766 (0·500–2·00)	3·75 (1·60–5·90)	0·780 (0·500–2·00)	3·95 (1·60–6·10)
North Africa and Middle East	80 plus	1990	0·768 (0·500–2·00)	4·00 (1·60–6·20)	0·783 (0·500–2·00)	4·04 (1·60–6·10)
North Africa and Middle East	15 to 19	2000	0·241 (0–0·400)	0·481 (0–1·10)	0·0570 (0–0·300)	0·104 (0–0·700)
North Africa and Middle East	20 to 24	2000	0·259 (0–0·400)	0·527 (0–1·10)	0·0487 (0–0·300)	0·0875 (0–0·602)
North Africa and Middle East	25 to 29	2000	0·303 (0–0·500)	0·667 (0–1·50)	0·129 (0–0·400)	0·246 (0–1·00)
North Africa and Middle East	30 to 34	2000	0·383 (0·200–0·500)	1·04 (0·300–2·50)	0·245 (0–0·500)	0·533 (0–1·40)
North Africa and Middle East	35 to 39	2000	0·428 (0·300–0·600)	1·32 (0·500–2·60)	0·352 (0–0·600)	0·999 (0–2·40)
North Africa and Middle East	40 to 44	2000	0·491 (0·400–0·702)	1·82 (0·700–3·30)	0·479 (0·200–1·00)	1·88 (0·400–4·10)
North Africa and Middle East	45 to 49	2000	0·583 (0·400–1·00)	2·49 (1·00–4·60)	0·648 (0·300–2·00)	2·77 (0·700–5·10)
North Africa and Middle East	50 to 54	2000	0·668 (0·500–1·00)	2·86 (1·20–4·90)	0·795 (0·400–2·00)	3·32 (1·00–5·50)

North Africa and Middle East	55 to 59	2000	0·729 (0·500–1·50)	3·23 (1·40–5·30)	0·877 (0·400–2·00)	3·80 (1·20–6·00)
North Africa and Middle East	60 to 64	2000	0·789 (0·500–1·80)	3·75 (1·50–5·80)	0·867 (0·500–2·00)	3·97 (1·30–6·10)
North Africa and Middle East	65 to 69	2000	0·826 (0·500–2·00)	4·06 (1·70–6·10)	0·901 (0·500–2·00)	4·22 (1·50–6·20)
North Africa and Middle East	70 to 74	2000	0·799 (0·500–2·00)	4·06 (1·70–6·10)	0·841 (0·500–2·00)	4·14 (1·50–6·10)
North Africa and Middle East	75 to 79	2000	0·807 (0·500–2·00)	4·17 (1·70–6·20)	0·836 (0·500–2·00)	4·29 (1·60–6·30)
North Africa and Middle East	80 plus	2000	0·828 (0·500–2·00)	4·40 (1·70–6·40)	0·840 (0·500–2·00)	4·38 (1·60–6·30)
North Africa and Middle East	15 to 19	2010	0·238 (0–0·400)	0·470 (0–1·10)	0·0697 (0–0·300)	0·127 (0–0·702)
North Africa and Middle East	20 to 24	2010	0·268 (0–0·400)	0·546 (0–1·20)	0·0634 (0–0·300)	0·114 (0–0·800)
North Africa and Middle East	25 to 29	2010	0·318 (0–0·500)	0·702 (0–1·40)	0·145 (0–0·400)	0·280 (0–1·00)
North Africa and Middle East	30 to 34	2010	0·394 (0·200–0·600)	1·06 (0·300–2·20)	0·248 (0–0·500)	0·537 (0–1·40)
North Africa and Middle East	35 to 39	2010	0·443 (0·300–0·600)	1·33 (0·500–2·50)	0·349 (0–0·600)	0·945 (0–2·30)
North Africa and Middle East	40 to 44	2010	0·514 (0·400–0·800)	1·84 (0·800–3·40)	0·481 (0·200–1·00)	1·87 (0·400–4·00)
North Africa and Middle East	45 to 49	2010	0·614 (0·400–1·00)	2·55 (1·10–4·50)	0·670 (0·400–1·90)	2·81 (0·800–5·10)
North Africa and Middle East	50 to 54	2010	0·700 (0·500–1·00)	3·02 (1·30–5·00)	0·820 (0·400–2·00)	3·36 (1·10–5·60)
North Africa and Middle East	55 to 59	2010	0·771 (0·500–1·50)	3·47 (1·50–5·40)	0·893 (0·500–2·00)	3·75 (1·30–5·90)
North Africa and Middle East	60 to 64	2010	0·827 (0·500–2·00)	3·92 (1·70–5·90)	0·894 (0·500–2·00)	3·94 (1·40–6·00)
North Africa and Middle East	65 to 69	2010	0·854 (0·500–2·00)	4·17 (1·80–6·10)	0·912 (0·500–2·00)	4·18 (1·60–6·20)
North Africa and Middle East	70 to 74	2010	0·833 (0·500–2·00)	4·20 (1·70–6·20)	0·856 (0·500–2·00)	4·14 (1·60–6·10)
North Africa and Middle East	75 to 79	2010	0·846 (0·500–2·00)	4·37 (1·80–6·30)	0·855 (0·500–2·00)	4·35 (1·70–6·30)
North Africa and Middle East	80 plus	2010	0·855 (0·500–2·00)	4·65 (1·80–6·60)	0·869 (0·500–2·00)	4·56 (1·70–6·40)
North Africa and Middle East	15 to 19	2020	0·266 (0–0·400)	0·539 (0–1·10)	0·0749 (0–0·400)	0·138 (0–0·800)
North Africa and Middle East	20 to 24	2020	0·302 (0–0·500)	0·639 (0–1·20)	0·0741 (0–0·400)	0·136 (0–0·800)
North Africa and Middle East	25 to 29	2020	0·349 (0·100–0·500)	0·792 (0·100–1·50)	0·168 (0–0·400)	0·327 (0–1·10)
North Africa and Middle East	30 to 34	2020	0·416 (0·200–0·600)	1·12 (0·400–2·20)	0·266 (0–0·500)	0·589 (0–1·50)
North Africa and Middle East	35 to 39	2020	0·465 (0·300–0·700)	1·40 (0·600–2·60)	0·372 (0–0·700)	1·04 (0–2·60)
North Africa and Middle East	40 to 44	2020	0·540 (0·400–0·800)	1·94 (0·800–3·50)	0·508 (0·298–1·00)	1·99 (0·498–4·20)
North Africa and Middle East	45 to 49	2020	0·639 (0·400–1·00)	2·65 (1·20–4·60)	0·695 (0·400–1·80)	2·95 (0·800–5·20)
North Africa and Middle East	50 to 54	2020	0·723 (0·500–1·00)	3·15 (1·40–5·10)	0·852 (0·500–2·00)	3·58 (1·20–5·70)
North Africa and Middle East	55 to 59	2020	0·798 (0·500–1·50)	3·68 (1·60–5·70)	0·940 (0·500–2·00)	4·01 (1·50–6·00)
North Africa and Middle East	60 to 64	2020	0·860 (0·600–2·00)	4·20 (1·80–6·10)	0·949 (0·500–2·00)	4·20 (1·60–6·10)
North Africa and Middle East	65 to 69	2020	0·892 (0·600–2·00)	4·43 (2·00–6·30)	0·957 (0·500–2·00)	4·40 (1·70–6·30)
North Africa and Middle East	70 to 74	2020	0·873 (0·600–2·00)	4·42 (1·90–6·30)	0·890 (0·500–2·00)	4·31 (1·70–6·20)
North Africa and Middle East	75 to 79	2020	0·884 (0·500–2·00)	4·61 (1·90–6·60)	0·882 (0·500–2·00)	4·46 (1·70–6·40)
North Africa and Middle East	80 plus	2020	0·897 (0·500–2·00)	4·93 (1·90–6·80)	0·907 (0·500–2·00)	4·71 (1·70–6·60)
South Asia	15 to 19	1990	0·186 (0–2·40)	0·322 (0–3·50)	0·0426 (0–0·100)	0·0617 (0–0·100)
South Asia	20 to 24	1990	0·277 (0–2·50)	0·483 (0–4·20)	0·0871 (0–1·00)	0·126 (0–1·00)
South Asia	25 to 29	1990	0·384 (0–2·60)	0·724 (0–5·80)	0·168 (0–2·10)	0·292 (0–3·00)
South Asia	30 to 34	1990	0·531 (0–2·80)	1·14 (0–9·10)	0·348 (0–2·30)	0·670 (0–4·10)
South Asia	35 to 39	1990	0·544 (0–2·60)	1·18 (0–7·50)	0·440 (0–2·30)	0·916 (0–5·10)
South Asia	40 to 44	1990	0·613 (0·100–2·50)	1·50 (0·200–7·91)	0·544 (0·100–2·30)	1·26 (0·100–6·10)
South Asia	45 to 49	1990	0·610 (0·300–2·30)	1·60 (0·400–6·50)	0·608 (0·200–2·40)	1·56 (0·300–6·50)

South Asia	50 to 54	1990	0·642 (0·300–2·30)	1·78 (0·500–7·00)	0·640 (0·300–2·30)	1·82 (0·500–6·80)
South Asia	55 to 59	1990	0·665 (0·300–2·20)	2·09 (0·700–7·30)	0·670 (0·300–2·30)	2·02 (0·600–7·20)
South Asia	60 to 64	1990	0·647 (0·400–2·10)	2·29 (0·900–6·60)	0·698 (0·400–2·30)	2·28 (0·700–7·60)
South Asia	65 to 69	1990	0·653 (0·400–2·00)	2·44 (1·00–6·50)	0·691 (0·400–2·20)	2·45 (0·800–7·40)
South Asia	70 to 74	1990	0·654 (0·400–2·10)	2·56 (1·00–7·00)	0·716 (0·400–2·30)	2·70 (0·900–8·00)
South Asia	75 to 79	1990	0·675 (0·400–2·20)	2·84 (1·00–7·30)	0·726 (0·400–2·30)	2·88 (1·00–8·00)
South Asia	80 plus	1990	0·679 (0·400–2·30)	2·86 (1·00–7·00)	0·745 (0·400–2·40)	3·01 (1·00–8·00)
South Asia	15 to 19	2000	0·144 (0–2·00)	0·237 (0–2·70)	0·0182 (0–0·100)	0·0262 (0–0·100)
South Asia	20 to 24	2000	0·217 (0–2·20)	0·375 (0–3·20)	0·0518 (0–0·300)	0·0859 (0–0·600)
South Asia	25 to 29	2000	0·315 (0–2·40)	0·589 (0–4·10)	0·132 (0–1·00)	0·237 (0–2·20)
South Asia	30 to 34	2000	0·453 (0–2·50)	0·947 (0–6·10)	0·336 (0–2·00)	0·647 (0–3·10)
South Asia	35 to 39	2000	0·475 (0–2·30)	1·01 (0–5·40)	0·420 (0–2·10)	0·864 (0–4·00)
South Asia	40 to 44	2000	0·560 (0·200–2·30)	1·33 (0·300–5·90)	0·530 (0·100–2·20)	1·19 (0·100–4·80)
South Asia	45 to 49	2000	0·579 (0·300–2·10)	1·54 (0·500–5·30)	0·594 (0·200–2·10)	1·49 (0·400–5·30)
South Asia	50 to 54	2000	0·605 (0·300–2·10)	1·69 (0·600–5·40)	0·632 (0·300–2·10)	1·79 (0·598–5·80)
South Asia	55 to 59	2000	0·649 (0·400–2·10)	2·05 (0·700–6·00)	0·664 (0·300–2·10)	2·03 (0·600–6·20)
South Asia	60 to 64	2000	0·632 (0·400–2·00)	2·27 (1·00–5·60)	0·678 (0·400–2·10)	2·27 (0·800–6·40)
South Asia	65 to 69	2000	0·635 (0·400–2·00)	2·48 (1·10–6·00)	0·684 (0·400–2·10)	2·49 (0·900–6·50)
South Asia	70 to 74	2000	0·645 (0·400–2·00)	2·63 (1·10–6·20)	0·712 (0·400–2·20)	2·74 (0·900–7·10)
South Asia	75 to 79	2000	0·667 (0·500–2·00)	2·93 (1·10–6·60)	0·727 (0·400–2·20)	3·00 (1·00–7·30)
South Asia	80 plus	2000	0·666 (0·500–2·00)	2·97 (1·20–6·50)	0·750 (0·400–2·30)	3·20 (1·10–7·30)
South Asia	15 to 19	2010	0·107 (0–1·00)	0·175 (0–1·20)	0·00830 (0–0·100)	0·0112 (0–0·100)
South Asia	20 to 24	2010	0·155 (0–1·00)	0·267 (0–1·50)	0·0387 (0–0·300)	0·0614 (0–0·500)
South Asia	25 to 29	2010	0·250 (0–2·00)	0·453 (0–2·70)	0·0946 (0–0·400)	0·164 (0–1·00)
South Asia	30 to 34	2010	0·384 (0–2·10)	0·787 (0–3·70)	0·265 (0–1·00)	0·528 (0–2·10)
South Asia	35 to 39	2010	0·428 (0–2·00)	0·936 (0–3·40)	0·371 (0–2·00)	0·770 (0–2·70)
South Asia	40 to 44	2010	0·523 (0·200–2·00)	1·31 (0·400–4·10)	0·484 (0·100–2·00)	1·14 (0·100–3·30)
South Asia	45 to 49	2010	0·568 (0·300–2·00)	1·60 (0·600–4·10)	0·565 (0·200–2·00)	1·49 (0·400–4·20)
South Asia	50 to 54	2010	0·605 (0·400–2·00)	1·76 (0·700–4·30)	0·622 (0·300–2·00)	1·86 (0·600–4·80)
South Asia	55 to 59	2010	0·642 (0·400–2·00)	2·08 (0·900–4·80)	0·661 (0·400–2·00)	2·11 (0·700–5·20)
South Asia	60 to 64	2010	0·648 (0·400–1·90)	2·44 (1·10–5·10)	0·682 (0·400–2·00)	2·41 (0·900–5·70)
South Asia	65 to 69	2010	0·655 (0·500–1·90)	2·70 (1·20–5·60)	0·690 (0·400–2·00)	2·68 (1·00–6·00)
South Asia	70 to 74	2010	0·661 (0·500–1·90)	2·87 (1·20–5·80)	0·717 (0·400–2·00)	2·98 (1·10–6·40)
South Asia	75 to 79	2010	0·687 (0·500–2·00)	3·22 (1·30–6·20)	0·728 (0·500–2·00)	3·25 (1·20–6·50)
South Asia	80 plus	2010	0·692 (0·500–1·90)	3·31 (1·30–6·10)	0·763 (0·500–2·20)	3·57 (1·20–6·80)
South Asia	15 to 19	2020	0·103 (0–1·00)	0·171 (0–1·10)	0·00640 (0–0·100)	0·00970 (0–0·100)
South Asia	20 to 24	2020	0·148 (0–1·00)	0·263 (0–1·20)	0·0427 (0–0·300)	0·0719 (0–0·600)
South Asia	25 to 29	2020	0·241 (0–1·00)	0·451 (0–1·50)	0·0943 (0–0·400)	0·171 (0–1·00)
South Asia	30 to 34	2020	0·362 (0–1·00)	0·772 (0–2·90)	0·277 (0–0·702)	0·584 (0–1·70)
South Asia	35 to 39	2020	0·416 (0·100–1·00)	0·973 (0·100–2·80)	0·367 (0–1·00)	0·826 (0–2·30)
South Asia	40 to 44	2020	0·525 (0·300–2·00)	1·41 (0·500–3·70)	0·498 (0·200–1·90)	1·22 (0·300–3·10)

South Asia	45 to 49	2020	0·569 (0·400–1·00)	1·70 (0·700–3·70)	0·584 (0·300–2·00)	1·61 (0·500–3·80)
South Asia	50 to 54	2020	0·623 (0·400–1·00)	2·00 (0·900–4·30)	0·647 (0·400–2·00)	2·01 (0·700–4·50)
South Asia	55 to 59	2020	0·679 (0·400–1·90)	2·41 (1·10–5·00)	0·695 (0·400–2·00)	2·33 (0·900–5·10)
South Asia	60 to 64	2020	0·678 (0·500–1·00)	2·81 (1·30–5·10)	0·711 (0·400–2·00)	2·69 (1·10–5·40)
South Asia	65 to 69	2020	0·685 (0·500–1·01)	3·07 (1·40–5·40)	0·718 (0·500–2·00)	3·00 (1·20–5·70)
South Asia	70 to 74	2020	0·681 (0·500–1·50)	3·24 (1·40–5·70)	0·741 (0·500–2·00)	3·34 (1·30–6·10)
South Asia	75 to 79	2020	0·718 (0·500–1·80)	3·64 (1·50–6·10)	0·765 (0·500–2·00)	3·65 (1·40–6·30)
South Asia	80 plus	2020	0·726 (0·500–1·50)	3·81 (1·60–6·20)	0·811 (0·500–2·10)	4·05 (1·40–6·70)
East Asia	15 to 19	1990	0·0773 (0–0·400)	0·134 (0–1·00)	0·0313 (0–0·200)	0·0526 (0–0·400)
East Asia	20 to 24	1990	0·134 (0–0·500)	0·244 (0–1·10)	0·0502 (0–0·300)	0·0860 (0–0·600)
East Asia	25 to 29	1990	0·215 (0–0·600)	0·416 (0–1·30)	0·108 (0–0·400)	0·194 (0–0·800)
East Asia	30 to 34	1990	0·254 (0–0·500)	0·515 (0–1·30)	0·196 (0–0·400)	0·375 (0–1·00)
East Asia	35 to 39	1990	0·311 (0–0·500)	0·685 (0–1·40)	0·262 (0–0·400)	0·545 (0–1·10)
East Asia	40 to 44	1990	0·371 (0·100–0·500)	0·932 (0·200–1·70)	0·326 (0–0·500)	0·740 (0–1·30)
East Asia	45 to 49	1990	0·414 (0·300–0·500)	1·18 (0·500–2·00)	0·370 (0·100–0·500)	0·902 (0·200–1·50)
East Asia	50 to 54	1990	0·455 (0·400–0·502)	1·43 (0·700–2·10)	0·409 (0·300–0·500)	1·12 (0·500–1·80)
East Asia	55 to 59	1990	0·481 (0·400–0·600)	1·62 (0·900–2·30)	0·432 (0·300–0·500)	1·29 (0·600–2·00)
East Asia	60 to 64	1990	0·502 (0·400–0·600)	1·83 (1·10–2·50)	0·463 (0·400–0·500)	1·51 (0·800–2·20)
East Asia	65 to 69	1990	0·511 (0·400–0·600)	1·95 (1·20–2·60)	0·486 (0·400–0·600)	1·72 (1·00–2·50)
East Asia	70 to 74	1990	0·515 (0·400–0·600)	2·06 (1·30–2·80)	0·499 (0·400–0·600)	1·86 (1·10–2·70)
East Asia	75 to 79	1990	0·520 (0·500–0·600)	2·20 (1·40–3·10)	0·506 (0·400–0·600)	2·04 (1·30–3·00)
East Asia	80 plus	1990	0·534 (0·500–0·707)	2·58 (1·70–4·10)	0·522 (0·500–0·600)	2·38 (1·50–3·80)
East Asia	15 to 19	2000	0·0680 (0–0·300)	0·117 (0–0·700)	0·0259 (0–0·200)	0·0431 (0–0·400)
East Asia	20 to 24	2000	0·141 (0–0·400)	0·263 (0–1·10)	0·0439 (0–0·300)	0·0775 (0–0·600)
East Asia	25 to 29	2000	0·221 (0–0·500)	0·445 (0–1·30)	0·100 (0–0·400)	0·182 (0–0·900)
East Asia	30 to 34	2000	0·263 (0–0·500)	0·545 (0–1·30)	0·189 (0–0·400)	0·368 (0–1·00)
East Asia	35 to 39	2000	0·321 (0–0·500)	0·710 (0–1·30)	0·271 (0–0·400)	0·577 (0–1·20)
East Asia	40 to 44	2000	0·380 (0·200–0·500)	0·981 (0·300–1·60)	0·347 (0–0·500)	0·826 (0–1·40)
East Asia	45 to 49	2000	0·425 (0·300–0·500)	1·29 (0·600–2·00)	0·384 (0·200–0·500)	0·989 (0·300–1·60)
East Asia	50 to 54	2000	0·463 (0·400–0·600)	1·53 (0·800–2·20)	0·417 (0·300–0·500)	1·20 (0·500–1·90)
East Asia	55 to 59	2000	0·493 (0·400–0·600)	1·70 (1·00–2·30)	0·440 (0·300–0·500)	1·38 (0·700–2·10)
East Asia	60 to 64	2000	0·514 (0·400–0·600)	1·99 (1·20–2·60)	0·477 (0·400–0·600)	1·65 (0·900–2·30)
East Asia	65 to 69	2000	0·521 (0·500–0·600)	2·16 (1·40–3·00)	0·496 (0·400–0·600)	1·89 (1·10–2·60)
East Asia	70 to 74	2000	0·524 (0·500–0·600)	2·33 (1·60–3·30)	0·503 (0·400–0·600)	2·06 (1·30–2·90)
East Asia	75 to 79	2000	0·527 (0·500–0·600)	2·49 (1·70–3·70)	0·510 (0·400–0·600)	2·26 (1·50–3·30)
East Asia	80 plus	2000	0·541 (0·500–0·700)	3·02 (1·90–4·90)	0·524 (0·500–0·600)	2·74 (1·80–4·40)
East Asia	15 to 19	2010	0·0814 (0–0·300)	0·143 (0–0·700)	0·0287 (0–0·300)	0·0501 (0–0·500)
East Asia	20 to 24	2010	0·164 (0–0·400)	0·303 (0–1·00)	0·0578 (0–0·300)	0·105 (0–0·700)
East Asia	25 to 29	2010	0·247 (0–0·500)	0·490 (0–1·10)	0·125 (0–0·400)	0·241 (0–1·00)
East Asia	30 to 34	2010	0·295 (0–0·500)	0·622 (0–1·20)	0·230 (0–0·400)	0·468 (0–1·20)
East Asia	35 to 39	2010	0·346 (0·100–0·500)	0·784 (0·100–1·40)	0·302 (0–0·500)	0·677 (0–1·30)

East Asia	40 to 44	2010	0·398 (0·200–0·500)	1·06 (0·400–1·70)	0·367 (0·100–0·500)	0·937 (0·200–1·70)
East Asia	45 to 49	2010	0·445 (0·300–0·600)	1·42 (0·700–2·10)	0·408 (0·200–0·500)	1·16 (0·400–2·00)
East Asia	50 to 54	2010	0·487 (0·400–0·600)	1·71 (1·00–2·30)	0·444 (0·300–0·500)	1·43 (0·700–2·20)
East Asia	55 to 59	2010	0·512 (0·400–0·600)	1·94 (1·20–2·70)	0·466 (0·400–0·600)	1·61 (0·800–2·40)
East Asia	60 to 64	2010	0·534 (0·500–0·700)	2·30 (1·40–3·30)	0·491 (0·400–0·600)	1·89 (1·10–2·70)
East Asia	65 to 69	2010	0·548 (0·500–0·700)	2·58 (1·70–3·90)	0·506 (0·400–0·600)	2·15 (1·30–3·10)
East Asia	70 to 74	2010	0·559 (0·500–0·700)	2·93 (1·90–4·50)	0·518 (0·500–0·600)	2·45 (1·60–3·70)
East Asia	75 to 79	2010	0·566 (0·500–0·800)	3·33 (1·90–5·10)	0·528 (0·500–0·600)	2·82 (1·80–4·40)
East Asia	80 plus	2010	0·626 (0·500–1·50)	4·22 (2·00–6·10)	0·576 (0·500–1·00)	3·69 (1·90–5·60)
East Asia	15 to 19	2020	0·112 (0–0·400)	0·195 (0–0·702)	0·0362 (0–0·300)	0·0633 (0–0·500)
East Asia	20 to 24	2020	0·212 (0–0·500)	0·403 (0–1·00)	0·0868 (0–0·400)	0·160 (0–0·900)
East Asia	25 to 29	2020	0·301 (0–0·500)	0·610 (0–1·20)	0·172 (0–0·400)	0·334 (0–1·10)
East Asia	30 to 34	2020	0·344 (0–0·500)	0·741 (0–1·30)	0·282 (0–0·500)	0·614 (0–1·30)
East Asia	35 to 39	2020	0·379 (0·198–0·600)	0·889 (0·298–1·50)	0·352 (0·0975–0·500)	0·870 (0·0975–1·60)
East Asia	40 to 44	2020	0·420 (0·300–0·600)	1·14 (0·500–1·80)	0·402 (0·200–0·500)	1·12 (0·400–2·00)
East Asia	45 to 49	2020	0·468 (0·400–0·600)	1·46 (0·800–2·20)	0·430 (0·300–0·600)	1·27 (0·600–2·10)
East Asia	50 to 54	2020	0·505 (0·400–0·600)	1·79 (1·00–2·50)	0·459 (0·400–0·600)	1·51 (0·800–2·30)
East Asia	55 to 59	2020	0·530 (0·400–0·700)	2·05 (1·20–2·90)	0·481 (0·400–0·600)	1·72 (0·900–2·50)
East Asia	60 to 64	2020	0·555 (0·500–0·700)	2·45 (1·50–3·60)	0·502 (0·400–0·600)	2·00 (1·10–3·00)
East Asia	65 to 69	2020	0·569 (0·500–0·800)	2·78 (1·80–4·30)	0·513 (0·400–0·600)	2·25 (1·30–3·30)
East Asia	70 to 74	2020	0·579 (0·500–0·802)	3·14 (1·90–4·80)	0·525 (0·500–0·600)	2·56 (1·60–3·90)
East Asia	75 to 79	2020	0·593 (0·500–1·00)	3·53 (2·00–5·40)	0·540 (0·500–0·602)	2·93 (1·80–4·50)
East Asia	80 plus	2020	0·662 (0·500–1·50)	4·46 (2·00–6·40)	0·610 (0·500–1·50)	3·92 (1·90–5·80)
Oceania	15 to 19	1990	0·269 (0–2·60)	0·499 (0–6·81)	0·0297 (0–0·200)	0·0429 (0–0·400)
Oceania	20 to 24	1990	0·330 (0–2·50)	0·646 (0–6·91)	0·0620 (0–0·300)	0·0987 (0–0·600)
Oceania	25 to 29	1990	0·419 (0–2·30)	0·952 (0–4·80)	0·230 (0–1·90)	0·442 (0–2·50)
Oceania	30 to 34	1990	0·520 (0·200–2·30)	1·34 (0·300–6·60)	0·483 (0·100–2·30)	1·16 (0·100–5·10)
Oceania	35 to 39	1990	0·592 (0·300–2·20)	1·64 (0·500–6·10)	0·544 (0·300–2·00)	1·54 (0·500–4·50)
Oceania	40 to 44	1990	0·659 (0·400–2·10)	2·14 (0·800–6·70)	0·705 (0·400–2·10)	2·33 (0·800–6·20)
Oceania	45 to 49	1990	0·645 (0·400–1·00)	2·45 (1·10–5·00)	0·784 (0·400–2·10)	2·76 (1·00–6·50)
Oceania	50 to 54	1990	0·690 (0·500–1·80)	2·66 (1·30–5·40)	0·821 (0·500–2·10)	3·23 (1·20–7·00)
Oceania	55 to 59	1990	0·706 (0·500–1·70)	2·88 (1·40–5·60)	0·835 (0·500–2·00)	3·55 (1·40–6·90)
Oceania	60 to 64	1990	0·704 (0·500–1·90)	3·29 (1·50–6·30)	0·818 (0·500–2·10)	3·69 (1·40–7·40)
Oceania	65 to 69	1990	0·692 (0·500–1·50)	3·38 (1·70–6·20)	0·810 (0·500–2·10)	3·62 (1·40–7·40)
Oceania	70 to 74	1990	0·699 (0·500–1·90)	3·63 (1·60–6·80)	0·760 (0·500–2·10)	3·70 (1·50–7·50)
Oceania	75 to 79	1990	0·662 (0·500–1·80)	3·51 (1·70–6·40)	0·758 (0·500–2·00)	3·82 (1·60–7·40)
Oceania	80 plus	1990	0·664 (0·500–1·50)	4·07 (2·00–6·40)	0·747 (0·500–2·00)	4·00 (1·60–7·40)
Oceania	15 to 19	2000	0·258 (0–2·40)	0·484 (0–4·50)	0·0235 (0–0·200)	0·0360 (0–0·302)
Oceania	20 to 24	2000	0·354 (0–2·50)	0·692 (0–5·11)	0·0661 (0–0·300)	0·110 (0–0·700)
Oceania	25 to 29	2000	0·438 (0–2·30)	1·00 (0–4·30)	0·245 (0–0·502)	0·466 (0–2·10)
Oceania	30 to 34	2000	0·530 (0·200–2·20)	1·41 (0·400–5·80)	0·484 (0·100–2·20)	1·17 (0·100–4·60)

Oceania	35 to 39	2000	0·596 (0·300–2·10)	1·72 (0·600–5·20)	0·567 (0·300–2·00)	1·64 (0·500–4·40)
Oceania	40 to 44	2000	0·680 (0·400–2·10)	2·28 (0·900–6·00)	0·742 (0·400–2·10)	2·49 (0·900–6·00)
Oceania	45 to 49	2000	0·679 (0·500–1·00)	2·64 (1·30–5·20)	0·819 (0·400–2·10)	2·89 (1·10–6·30)
Oceania	50 to 54	2000	0·720 (0·500–1·00)	2·89 (1·40–5·40)	0·864 (0·500–2·10)	3·47 (1·30–6·80)
Oceania	55 to 59	2000	0·739 (0·500–1·00)	3·15 (1·60–5·60)	0·875 (0·500–2·00)	3·78 (1·60–6·80)
Oceania	60 to 64	2000	0·759 (0·500–1·80)	3·70 (1·80–6·30)	0·863 (0·500–2·10)	3·98 (1·60–7·20)
Oceania	65 to 69	2000	0·749 (0·500–1·50)	3·78 (2·00–6·20)	0·838 (0·500–2·10)	3·94 (1·60–7·10)
Oceania	70 to 74	2000	0·722 (0·500–1·90)	3·91 (1·80–6·70)	0·793 (0·500–2·10)	3·95 (1·60–7·30)
Oceania	75 to 79	2000	0·671 (0·500–1·60)	3·71 (1·80–6·50)	0·780 (0·500–2·00)	3·99 (1·60–7·20)
Oceania	80 plus	2000	0·673 (0·500–1·50)	4·14 (2·00–6·50)	0·770 (0·500–2·10)	4·10 (1·60–7·40)
Oceania	15 to 19	2010	0·274 (0–2·40)	0·514 (0–4·40)	0·0300 (0–0·200)	0·0412 (0–0·300)
Oceania	20 to 24	2010	0·347 (0–2·50)	0·668 (0–4·90)	0·0861 (0–0·400)	0·134 (0–0·702)
Oceania	25 to 29	2010	0·430 (0·100–2·20)	0·994 (0·100–3·80)	0·252 (0–0·602)	0·502 (0–2·30)
Oceania	30 to 34	2010	0·542 (0·300–2·20)	1·44 (0·400–5·20)	0·497 (0·100–2·10)	1·22 (0·200–4·50)
Oceania	35 to 39	2010	0·606 (0·300–2·10)	1·73 (0·700–5·10)	0·579 (0·300–2·00)	1·70 (0·600–4·40)
Oceania	40 to 44	2010	0·691 (0·400–2·00)	2·33 (1·00–5·70)	0·758 (0·400–2·10)	2·52 (0·900–6·10)
Oceania	45 to 49	2010	0·694 (0·500–1·00)	2·70 (1·40–5·00)	0·828 (0·500–2·10)	2·92 (1·10–6·30)
Oceania	50 to 54	2010	0·738 (0·500–1·00)	2·98 (1·50–5·40)	0·875 (0·500–2·10)	3·51 (1·30–6·70)
Oceania	55 to 59	2010	0·765 (0·500–1·00)	3·29 (1·80–5·60)	0·899 (0·500–2·10)	3·89 (1·60–6·80)
Oceania	60 to 64	2010	0·776 (0·500–1·50)	3·81 (1·90–6·20)	0·888 (0·500–2·10)	4·12 (1·70–7·10)
Oceania	65 to 69	2010	0·772 (0·600–1·50)	3·93 (2·10–6·20)	0·866 (0·500–2·10)	4·06 (1·70–7·20)
Oceania	70 to 74	2010	0·746 (0·600–1·70)	4·11 (2·10–6·60)	0·824 (0·500–2·10)	4·13 (1·70–7·20)
Oceania	75 to 79	2010	0·704 (0·500–1·50)	3·97 (2·00–6·40)	0·810 (0·500–2·10)	4·23 (1·70–7·10)
Oceania	80 plus	2010	0·679 (0·500–1·50)	4·28 (2·10–6·50)	0·788 (0·500–2·10)	4·20 (1·60–7·60)
Oceania	15 to 19	2020	0·224 (0–2·20)	0·416 (0–3·20)	0·0216 (0–0·200)	0·0348 (0–0·400)
Oceania	20 to 24	2020	0·298 (0–2·20)	0·569 (0–3·30)	0·0674 (0–0·400)	0·113 (0–0·700)
Oceania	25 to 29	2020	0·396 (0·100–0·702)	0·922 (0·100–3·00)	0·249 (0–0·602)	0·488 (0–1·40)
Oceania	30 to 34	2020	0·524 (0·298–2·00)	1·35 (0·400–4·00)	0·482 (0·200–2·10)	1·23 (0·298–4·10)
Oceania	35 to 39	2020	0·602 (0·400–1·00)	1·68 (0·700–4·10)	0·583 (0·300–1·90)	1·75 (0·698–4·30)
Oceania	40 to 44	2020	0·685 (0·400–1·00)	2·26 (1·00–5·00)	0·750 (0·400–2·00)	2·54 (1·00–5·90)
Oceania	45 to 49	2020	0·696 (0·500–1·00)	2·62 (1·40–4·70)	0·820 (0·500–2·00)	2·93 (1·20–6·10)
Oceania	50 to 54	2020	0·737 (0·500–1·00)	2·93 (1·60–5·10)	0·869 (0·500–2·00)	3·52 (1·40–6·50)
Oceania	55 to 59	2020	0·761 (0·500–1·00)	3·25 (1·80–5·40)	0·898 (0·600–2·00)	3·92 (1·70–6·60)
Oceania	60 to 64	2020	0·774 (0·600–1·00)	3·77 (2·10–5·90)	0·882 (0·600–2·10)	4·18 (1·80–6·80)
Oceania	65 to 69	2020	0·770 (0·600–1·00)	3·94 (2·20–6·00)	0·868 (0·600–2·10)	4·12 (1·80–6·90)
Oceania	70 to 74	2020	0·753 (0·600–1·50)	4·14 (2·10–6·40)	0·817 (0·500–2·00)	4·17 (1·80–7·10)
Oceania	75 to 79	2020	0·714 (0·500–1·50)	4·05 (2·10–6·50)	0·814 (0·500–2·00)	4·29 (1·90–7·10)
Oceania	80 plus	2020	0·701 (0·500–1·50)	4·46 (2·30–6·50)	0·798 (0·500–2·10)	4·37 (1·80–7·30)
Southeast Asia	15 to 19	1990	0·294 (0–2·40)	0·543 (0–5·11)	0·0140 (0–0·100)	0·0191 (0–0·200)
Southeast Asia	20 to 24	1990	0·442 (0–3·00)	0·890 (0–10·0)	0·0578 (0–0·300)	0·0810 (0–0·500)
Southeast Asia	25 to 29	1990	0·552 (0–3·00)	1·20 (0–10·0)	0·142 (0–2·00)	0·251 (0–2·40)

Southeast Asia	30 to 34	1990	0·483 (0·100–2·30)	1·08 (0·100–5·50)	0·255 (0–2·00)	0·490 (0–2·20)
Southeast Asia	35 to 39	1990	0·537 (0·200–2·20)	1·32 (0·300–6·41)	0·345 (0–1·02)	0·733 (0–2·40)
Southeast Asia	40 to 44	1990	0·487 (0·300–2·00)	1·29 (0·400–3·80)	0·415 (0·100–1·00)	0·963 (0·100–2·40)
Southeast Asia	45 to 49	1990	0·505 (0·300–2·00)	1·48 (0·600–4·20)	0·455 (0·200–1·00)	1·16 (0·400–2·70)
Southeast Asia	50 to 54	1990	0·521 (0·400–1·90)	1·64 (0·798–3·90)	0·494 (0·300–1·51)	1·38 (0·500–3·20)
Southeast Asia	55 to 59	1990	0·537 (0·400–1·00)	1·78 (0·900–4·00)	0·510 (0·300–1·00)	1·55 (0·700–3·60)
Southeast Asia	60 to 64	1990	0·556 (0·400–1·00)	1·90 (1·00–4·00)	0·533 (0·400–1·70)	1·74 (0·800–4·20)
Southeast Asia	65 to 69	1990	0·562 (0·400–1·00)	2·08 (1·10–4·30)	0·547 (0·400–1·90)	1·87 (0·800–4·60)
Southeast Asia	70 to 74	1990	0·570 (0·400–1·00)	2·20 (1·10–4·70)	0·566 (0·400–1·90)	2·07 (0·900–5·40)
Southeast Asia	75 to 79	1990	0·565 (0·497–1·00)	2·30 (1·20–4·80)	0·574 (0·400–1·90)	2·22 (1·00–5·80)
Southeast Asia	80 plus	1990	0·580 (0·500–1·00)	2·54 (1·20–5·20)	0·608 (0·400–2·00)	2·46 (1·00–6·50)
Southeast Asia	15 to 19	2000	0·261 (0–2·30)	0·467 (0–3·90)	0·0102 (0–0·200)	0·0160 (0–0·300)
Southeast Asia	20 to 24	2000	0·380 (0–2·50)	0·771 (0–7·50)	0·0378 (0–0·202)	0·0556 (0–0·402)
Southeast Asia	25 to 29	2000	0·467 (0–2·50)	0·978 (0–7·41)	0·113 (0–0·400)	0·194 (0–1·10)
Southeast Asia	30 to 34	2000	0·443 (0·100–2·10)	0·987 (0·100–4·20)	0·220 (0–0·500)	0·435 (0–1·30)
Southeast Asia	35 to 39	2000	0·496 (0·200–2·00)	1·24 (0·300–4·80)	0·327 (0–1·00)	0·699 (0–2·10)
Southeast Asia	40 to 44	2000	0·475 (0·300–1·02)	1·28 (0·500–3·20)	0·402 (0·100–1·00)	0·962 (0·200–2·30)
Southeast Asia	45 to 49	2000	0·496 (0·300–1·00)	1·50 (0·700–3·50)	0·450 (0·200–1·00)	1·19 (0·400–2·50)
Southeast Asia	50 to 54	2000	0·522 (0·400–1·00)	1·68 (0·900–3·50)	0·495 (0·300–1·00)	1·45 (0·600–3·00)
Southeast Asia	55 to 59	2000	0·539 (0·400–1·00)	1·82 (1·00–3·60)	0·523 (0·400–1·00)	1·65 (0·798–3·50)
Southeast Asia	60 to 64	2000	0·560 (0·400–1·00)	1·96 (1·10–3·80)	0·542 (0·400–1·00)	1·84 (0·900–4·10)
Southeast Asia	65 to 69	2000	0·568 (0·500–1·00)	2·17 (1·20–4·10)	0·546 (0·400–1·00)	1·96 (1·00–4·30)
Southeast Asia	70 to 74	2000	0·572 (0·500–1·00)	2·27 (1·20–4·30)	0·566 (0·400–1·01)	2·13 (1·00–5·00)
Southeast Asia	75 to 79	2000	0·566 (0·500–1·00)	2·39 (1·30–4·70)	0·572 (0·400–1·50)	2·28 (1·10–5·20)
Southeast Asia	80 plus	2000	0·590 (0·500–1·00)	2·71 (1·40–5·20)	0·612 (0·400–2·00)	2·57 (1·10–6·10)
Southeast Asia	15 to 19	2010	0·230 (0–2·00)	0·423 (0–3·00)	0·0170 (0–0·200)	0·0279 (0–0·400)
Southeast Asia	20 to 24	2010	0·386 (0–2·40)	0·764 (0–5·90)	0·0522 (0–0·300)	0·0862 (0–0·600)
Southeast Asia	25 to 29	2010	0·463 (0–2·30)	1·00 (0–6·10)	0·173 (0–0·512)	0·307 (0–1·30)
Southeast Asia	30 to 34	2010	0·428 (0·100–2·00)	1·01 (0·200–3·20)	0·283 (0–0·602)	0·585 (0–1·70)
Southeast Asia	35 to 39	2010	0·464 (0·200–2·00)	1·21 (0·400–3·50)	0·359 (0–1·00)	0·826 (0–2·10)
Southeast Asia	40 to 44	2010	0·460 (0·300–0·700)	1·31 (0·600–2·70)	0·419 (0·200–1·00)	1·06 (0·300–2·20)
Southeast Asia	45 to 49	2010	0·488 (0·400–0·700)	1·53 (0·800–2·90)	0·457 (0·300–1·00)	1·26 (0·500–2·40)
Southeast Asia	50 to 54	2010	0·518 (0·400–0·702)	1·73 (0·998–3·10)	0·496 (0·300–1·00)	1·52 (0·700–2·90)
Southeast Asia	55 to 59	2010	0·548 (0·400–0·800)	1·91 (1·10–3·40)	0·525 (0·400–1·00)	1·74 (0·900–3·30)
Southeast Asia	60 to 64	2010	0·569 (0·500–0·800)	2·07 (1·20–3·60)	0·552 (0·400–1·00)	1·95 (1·00–3·90)
Southeast Asia	65 to 69	2010	0·581 (0·500–0·900)	2·30 (1·30–3·90)	0·556 (0·400–1·00)	2·08 (1·10–4·20)
Southeast Asia	70 to 74	2010	0·584 (0·500–1·00)	2·38 (1·40–4·10)	0·573 (0·400–1·00)	2·25 (1·10–4·80)
Southeast Asia	75 to 79	2010	0·580 (0·500–1·00)	2·50 (1·50–4·30)	0·575 (0·400–1·00)	2·38 (1·20–5·00)
Southeast Asia	80 plus	2010	0·602 (0·500–1·00)	2·88 (1·50–5·10)	0·607 (0·500–1·50)	2·67 (1·20–5·80)
Southeast Asia	15 to 19	2020	0·220 (0–0·500)	0·422 (0–2·10)	0·0244 (0–0·200)	0·0405 (0–0·400)
Southeast Asia	20 to 24	2020	0·368 (0–2·20)	0·724 (0–3·60)	0·0575 (0–0·300)	0·100 (0–0·700)

Southeast Asia	25 to 29	2020	0·422 (0·100–2·00)	0·935 (0·100–3·90)	0·166 (0–0·400)	0·307 (0–1·10)
Southeast Asia	30 to 34	2020	0·414 (0·200–0·800)	1·02 (0·300–2·50)	0·299 (0–0·502)	0·632 (0–1·50)
Southeast Asia	35 to 39	2020	0·454 (0·300–0·800)	1·22 (0·500–2·70)	0·378 (0·100–0·700)	0·895 (0·100–2·00)
Southeast Asia	40 to 44	2020	0·485 (0·300–0·700)	1·39 (0·700–2·50)	0·444 (0·200–1·00)	1·17 (0·400–2·20)
Southeast Asia	45 to 49	2020	0·511 (0·400–0·800)	1·61 (0·900–2·60)	0·481 (0·300–1·00)	1·39 (0·600–2·50)
Southeast Asia	50 to 54	2020	0·543 (0·400–0·800)	1·83 (1·10–2·90)	0·518 (0·400–1·00)	1·65 (0·900–2·90)
Southeast Asia	55 to 59	2020	0·573 (0·400–0·800)	2·02 (1·20–3·20)	0·539 (0·400–1·00)	1·87 (1·00–3·30)
Southeast Asia	60 to 64	2020	0·594 (0·500–0·800)	2·21 (1·40–3·40)	0·560 (0·400–1·00)	2·07 (1·10–3·70)
Southeast Asia	65 to 69	2020	0·608 (0·500–0·900)	2·48 (1·60–3·90)	0·572 (0·500–1·00)	2·21 (1·20–4·00)
Southeast Asia	70 to 74	2020	0·608 (0·500–1·00)	2·57 (1·60–4·10)	0·586 (0·500–1·00)	2·42 (1·30–4·50)
Southeast Asia	75 to 79	2020	0·600 (0·500–1·00)	2·69 (1·60–4·40)	0·588 (0·500–1·00)	2·56 (1·40–4·80)
Southeast Asia	80 plus	2020	0·619 (0·500–1·00)	3·07 (1·70–5·00)	0·619 (0·500–1·50)	2·85 (1·40–5·40)
Central Sub-Saharan Africa	15 to 19	1990	0·428 (0–5·00)	0·730 (0–10·0)	0·0200 (0–0·100)	0·0263 (0–0·200)
Central Sub-Saharan Africa	20 to 24	1990	0·631 (0–8·02)	1·04 (0–10·0)	0·0516 (0–0·200)	0·0715 (0–0·400)
Central Sub-Saharan Africa	25 to 29	1990	0·761 (0–10·0)	1·23 (0–10·0)	0·112 (0–2·10)	0·192 (0–2·90)
Central Sub-Saharan Africa	30 to 34	1990	0·734 (0–9·00)	1·28 (0–10·0)	0·191 (0–2·30)	0·340 (0–3·80)
Central Sub-Saharan Africa	35 to 39	1990	0·708 (0–7·00)	1·38 (0–10·0)	0·272 (0–2·20)	0·488 (0–4·40)
Central Sub-Saharan Africa	40 to 44	1990	0·616 (0–2·70)	1·38 (0–10·0)	0·359 (0–2·00)	0·683 (0–3·70)
Central Sub-Saharan Africa	45 to 49	1990	0·612 (0·100–2·50)	1·52 (0·200–10·0)	0·472 (0–2·20)	0·981 (0–4·61)
Central Sub-Saharan Africa	50 to 54	1990	0·663 (0·200–2·50)	1·70 (0·400–10·0)	0·554 (0·100–2·20)	1·26 (0·200–6·10)
Central Sub-Saharan Africa	55 to 59	1990	0·650 (0·300–2·40)	1·87 (0·500–10·0)	0·585 (0·200–2·10)	1·41 (0·400–6·10)
Central Sub-Saharan Africa	60 to 64	1990	0·655 (0·300–2·30)	2·02 (0·600–9·20)	0·623 (0·300–2·10)	1·64 (0·500–6·20)
Central Sub-Saharan Africa	65 to 69	1990	0·634 (0·400–2·10)	2·18 (0·700–7·80)	0·629 (0·300–2·00)	1·74 (0·600–6·10)
Central Sub-Saharan Africa	70 to 74	1990	0·636 (0·400–2·00)	2·34 (0·900–7·20)	0·658 (0·400–2·10)	2·07 (0·700–7·30)
Central Sub-Saharan Africa	75 to 79	1990	0·621 (0·400–2·00)	2·39 (0·900–6·80)	0·662 (0·400–2·10)	2·15 (0·800–7·30)
Central Sub-Saharan Africa	80 plus	1990	0·624 (0·400–1·50)	2·48 (1·00–6·10)	0·676 (0·400–2·20)	2·39 (0·800–7·40)
Central Sub-Saharan Africa	15 to 19	2000	0·443 (0–5·00)	0·776 (0–10·0)	0·0287 (0–0·100)	0·0332 (0–0·100)
Central Sub-Saharan Africa	20 to 24	2000	0·624 (0–8·02)	1·06 (0–10·0)	0·0506 (0–0·200)	0·0718 (0–0·300)
Central Sub-Saharan Africa	25 to 29	2000	0·762 (0–10·0)	1·26 (0–10·0)	0·114 (0–2·10)	0·199 (0–3·10)
Central Sub-Saharan Africa	30 to 34	2000	0·734 (0–8·02)	1·31 (0–10·0)	0·199 (0–2·40)	0·368 (0–4·41)
Central Sub-Saharan Africa	35 to 39	2000	0·720 (0–7·00)	1·44 (0–10·0)	0·262 (0–2·30)	0·493 (0–4·11)
Central Sub-Saharan Africa	40 to 44	2000	0·624 (0–2·71)	1·42 (0–10·0)	0·363 (0–2·10)	0·725 (0–4·50)
Central Sub-Saharan Africa	45 to 49	2000	0·623 (0·100–2·50)	1·54 (0·200–10·0)	0·485 (0–2·20)	1·02 (0–5·90)
Central Sub-Saharan Africa	50 to 54	2000	0·674 (0·200–2·50)	1·73 (0·400–10·0)	0·556 (0·200–2·20)	1·28 (0·300–6·31)
Central Sub-Saharan Africa	55 to 59	2000	0·677 (0·300–2·40)	1·89 (0·500–10·0)	0·595 (0·200–2·10)	1·45 (0·400–6·30)
Central Sub-Saharan Africa	60 to 64	2000	0·672 (0·300–2·30)	2·03 (0·600–8·70)	0·626 (0·300–2·10)	1·67 (0·500–6·10)
Central Sub-Saharan Africa	65 to 69	2000	0·652 (0·400–2·20)	2·19 (0·700–7·70)	0·632 (0·300–2·00)	1·78 (0·600–6·20)
Central Sub-Saharan Africa	70 to 74	2000	0·640 (0·400–2·10)	2·35 (0·800–7·60)	0·666 (0·400–2·20)	2·09 (0·700–7·40)
Central Sub-Saharan Africa	75 to 79	2000	0·627 (0·400–2·00)	2·39 (0·900–6·90)	0·663 (0·400–2·10)	2·18 (0·800–7·20)
Central Sub-Saharan Africa	80 plus	2000	0·636 (0·400–1·80)	2·47 (0·900–6·20)	0·686 (0·400–2·20)	2·43 (0·800–7·60)
Central Sub-Saharan Africa	15 to 19	2010	0·359 (0–3·02)	0·642 (0–10·0)	0·0177 (0–0·100)	0·0256 (0–0·100)

Central Sub-Saharan Africa	20 to 24	2010	0·545 (0–7·00)	0·967 (0–10·0)	0·0418 (0–0·200)	0·0520 (0–0·300)
Central Sub-Saharan Africa	25 to 29	2010	0·651 (0–7·00)	1·16 (0–10·0)	0·102 (0–2·00)	0·169 (0–2·70)
Central Sub-Saharan Africa	30 to 34	2010	0·647 (0–7·00)	1·20 (0–10·0)	0·185 (0–2·30)	0·332 (0–3·90)
Central Sub-Saharan Africa	35 to 39	2010	0·655 (0–5·02)	1·36 (0–10·0)	0·258 (0–2·20)	0·487 (0–4·10)
Central Sub-Saharan Africa	40 to 44	2010	0·583 (0–2·60)	1·32 (0–10·0)	0·373 (0–2·00)	0·710 (0–3·90)
Central Sub-Saharan Africa	45 to 49	2010	0·611 (0·200–2·40)	1·47 (0·300–10·0)	0·470 (0–2·10)	0·994 (0–4·61)
Central Sub-Saharan Africa	50 to 54	2010	0·647 (0·200–2·50)	1·68 (0·400–10·0)	0·563 (0·200–2·20)	1·27 (0·300–6·10)
Central Sub-Saharan Africa	55 to 59	2010	0·663 (0·300–2·30)	1·84 (0·500–10·0)	0·592 (0·200–2·00)	1·43 (0·400–5·40)
Central Sub-Saharan Africa	60 to 64	2010	0·659 (0·300–2·30)	2·03 (0·700–8·80)	0·625 (0·300–2·00)	1·67 (0·600–5·41)
Central Sub-Saharan Africa	65 to 69	2010	0·645 (0·400–2·20)	2·14 (0·798–7·70)	0·630 (0·300–2·00)	1·79 (0·700–5·30)
Central Sub-Saharan Africa	70 to 74	2010	0·631 (0·400–2·00)	2·31 (0·900–6·90)	0·659 (0·400–2·10)	2·13 (0·800–7·00)
Central Sub-Saharan Africa	75 to 79	2010	0·623 (0·400–2·00)	2·37 (0·900–6·60)	0·652 (0·400–2·10)	2·21 (0·800–6·80)
Central Sub-Saharan Africa	80 plus	2010	0·628 (0·400–1·50)	2·47 (1·00–6·00)	0·680 (0·400–2·10)	2·49 (0·900–7·10)
Central Sub-Saharan Africa	15 to 19	2020	0·212 (0–2·60)	0·362 (0–5·01)	0·0137 (0–0·100)	0·0172 (0–0·100)
Central Sub-Saharan Africa	20 to 24	2020	0·355 (0–3·00)	0·658 (0–10·0)	0·0195 (0–0·100)	0·0292 (0–0·200)
Central Sub-Saharan Africa	25 to 29	2020	0·459 (0–4·02)	0·826 (0–10·0)	0·0630 (0–0·302)	0·0996 (0–0·802)
Central Sub-Saharan Africa	30 to 34	2020	0·476 (0–3·00)	0·932 (0–10·0)	0·127 (0–2·00)	0·227 (0–2·60)
Central Sub-Saharan Africa	35 to 39	2020	0·518 (0–2·60)	1·11 (0–10·0)	0·211 (0–2·00)	0·378 (0–3·10)
Central Sub-Saharan Africa	40 to 44	2020	0·503 (0·100–2·30)	1·15 (0·100–6·61)	0·353 (0–2·00)	0·655 (0–3·00)
Central Sub-Saharan Africa	45 to 49	2020	0·534 (0·200–2·20)	1·33 (0·300–6·20)	0·463 (0–2·00)	0·958 (0–3·60)
Central Sub-Saharan Africa	50 to 54	2020	0·571 (0·298–2·20)	1·51 (0·400–6·50)	0·556 (0·200–2·00)	1·27 (0·398–4·00)
Central Sub-Saharan Africa	55 to 59	2020	0·611 (0·300–2·10)	1·72 (0·600–6·70)	0·599 (0·300–2·00)	1·45 (0·500–4·20)
Central Sub-Saharan Africa	60 to 64	2020	0·624 (0·400–2·00)	1·94 (0·798–6·20)	0·625 (0·400–2·00)	1·73 (0·700–4·90)
Central Sub-Saharan Africa	65 to 69	2020	0·625 (0·400–2·00)	2·09 (0·900–5·50)	0·621 (0·400–2·00)	1·85 (0·800–4·40)
Central Sub-Saharan Africa	70 to 74	2020	0·626 (0·400–1·50)	2·31 (1·00–5·70)	0·649 (0·400–2·00)	2·20 (0·900–5·50)
Central Sub-Saharan Africa	75 to 79	2020	0·625 (0·400–1·50)	2·42 (1·10–5·50)	0·650 (0·400–2·00)	2·31 (1·00–5·60)
Central Sub-Saharan Africa	80 plus	2020	0·638 (0·497–1·50)	2·58 (1·10–5·60)	0·673 (0·500–2·00)	2·64 (1·10–6·20)
Eastern Sub-Saharan Africa	15 to 19	1990	0·355 (0–2·60)	0·668 (0–8·30)	0·106 (0–1·02)	0·180 (0–2·00)
Eastern Sub-Saharan Africa	20 to 24	1990	0·607 (0–5·00)	1·14 (0–10·0)	0·205 (0–2·40)	0·368 (0–4·10)
Eastern Sub-Saharan Africa	25 to 29	1990	0·731 (0–8·00)	1·29 (0–10·0)	0·283 (0–2·50)	0·546 (0–7·20)
Eastern Sub-Saharan Africa	30 to 34	1990	0·673 (0–6·00)	1·34 (0–10·0)	0·473 (0–3·00)	0·966 (0–10·0)
Eastern Sub-Saharan Africa	35 to 39	1990	0·695 (0–5·00)	1·48 (0–10·0)	0·488 (0–2·80)	0·994 (0–8·51)
Eastern Sub-Saharan Africa	40 to 44	1990	0·522 (0·100–2·20)	1·17 (0·100–6·41)	0·452 (0–2·20)	0·927 (0–5·01)
Eastern Sub-Saharan Africa	45 to 49	1990	0·540 (0·100–2·20)	1·27 (0·200–6·70)	0·522 (0·0975–2·30)	1·19 (0·0975–6·80)
Eastern Sub-Saharan Africa	50 to 54	1990	0·612 (0·200–2·40)	1·50 (0·300–10·0)	0·555 (0·100–2·30)	1·30 (0·100–7·11)
Eastern Sub-Saharan Africa	55 to 59	1990	0·591 (0·200–2·20)	1·55 (0·400–7·91)	0·580 (0·200–2·30)	1·44 (0·300–7·40)
Eastern Sub-Saharan Africa	60 to 64	1990	0·579 (0·300–2·10)	1·55 (0·500–6·40)	0·540 (0·300–2·00)	1·42 (0·500–4·40)
Eastern Sub-Saharan Africa	65 to 69	1990	0·526 (0·300–2·00)	1·51 (0·600–4·10)	0·521 (0·300–2·00)	1·44 (0·500–4·10)
Eastern Sub-Saharan Africa	70 to 74	1990	0·553 (0·300–2·00)	1·68 (0·700–5·30)	0·580 (0·300–2·10)	1·69 (0·600–6·10)
Eastern Sub-Saharan Africa	75 to 79	1990	0·549 (0·400–1·50)	1·74 (0·800–4·60)	0·561 (0·300–2·00)	1·68 (0·600–6·10)
Eastern Sub-Saharan Africa	80 plus	1990	0·562 (0·300–1·50)	1·74 (0·800–4·80)	0·588 (0·300–2·10)	1·84 (0·700–6·80)

Eastern Sub-Saharan Africa	15 to 19	2000	0·299 (0–2·50)	0·553 (0–6·21)	0·0732 (0–0·300)	0·118 (0–0·600)
Eastern Sub-Saharan Africa	20 to 24	2000	0·472 (0–3·00)	0·950 (0–10·0)	0·130 (0–2·00)	0·230 (0–3·10)
Eastern Sub-Saharan Africa	25 to 29	2000	0·558 (0–4·00)	1·10 (0–10·0)	0·219 (0–2·40)	0·394 (0–4·41)
Eastern Sub-Saharan Africa	30 to 34	2000	0·554 (0–3·00)	1·17 (0–10·0)	0·389 (0–2·60)	0·778 (0–7·90)
Eastern Sub-Saharan Africa	35 to 39	2000	0·573 (0–2·60)	1·31 (0–10·0)	0·420 (0–2·40)	0·818 (0–6·40)
Eastern Sub-Saharan Africa	40 to 44	2000	0·491 (0·100–2·10)	1·09 (0·100–4·61)	0·406 (0–2·00)	0·821 (0–3·70)
Eastern Sub-Saharan Africa	45 to 49	2000	0·505 (0·200–2·10)	1·18 (0·298–5·00)	0·488 (0–2·10)	1·06 (0–5·00)
Eastern Sub-Saharan Africa	50 to 54	2000	0·558 (0·200–2·20)	1·37 (0·300–7·40)	0·518 (0·100–2·10)	1·18 (0·200–5·61)
Eastern Sub-Saharan Africa	55 to 59	2000	0·563 (0·300–2·10)	1·47 (0·498–6·40)	0·546 (0·200–2·10)	1·34 (0·400–5·61)
Eastern Sub-Saharan Africa	60 to 64	2000	0·555 (0·300–2·00)	1·50 (0·500–5·10)	0·525 (0·300–2·00)	1·41 (0·500–3·90)
Eastern Sub-Saharan Africa	65 to 69	2000	0·527 (0·300–1·51)	1·51 (0·700–3·60)	0·515 (0·300–2·00)	1·43 (0·600–3·60)
Eastern Sub-Saharan Africa	70 to 74	2000	0·551 (0·400–1·90)	1·68 (0·700–4·50)	0·570 (0·300–2·00)	1·68 (0·700–5·60)
Eastern Sub-Saharan Africa	75 to 79	2000	0·547 (0·400–1·00)	1·76 (0·800–4·20)	0·567 (0·300–2·00)	1·70 (0·700–5·20)
Eastern Sub-Saharan Africa	80 plus	2000	0·563 (0·400–1·50)	1·78 (0·800–4·50)	0·591 (0·400–2·00)	1·87 (0·700–6·70)
Eastern Sub-Saharan Africa	15 to 19	2010	0·212 (0–2·20)	0·382 (0–3·30)	0·0374 (0–0·200)	0·0563 (0–0·400)
Eastern Sub-Saharan Africa	20 to 24	2010	0·352 (0–2·50)	0·669 (0–6·51)	0·0698 (0–0·415)	0·118 (0–1·00)
Eastern Sub-Saharan Africa	25 to 29	2010	0·413 (0–2·60)	0·798 (0–8·00)	0·132 (0–2·00)	0·242 (0–3·00)
Eastern Sub-Saharan Africa	30 to 34	2010	0·441 (0–2·40)	0·911 (0–7·20)	0·313 (0–2·40)	0·600 (0–5·00)
Eastern Sub-Saharan Africa	35 to 39	2010	0·479 (0–2·30)	1·03 (0–6·70)	0·344 (0–2·20)	0·674 (0–4·30)
Eastern Sub-Saharan Africa	40 to 44	2010	0·422 (0·100–2·00)	0·936 (0·100–3·00)	0·371 (0–2·00)	0·739 (0–2·70)
Eastern Sub-Saharan Africa	45 to 49	2010	0·452 (0·200–2·00)	1·05 (0·300–3·10)	0·442 (0–2·00)	0·961 (0–3·40)
Eastern Sub-Saharan Africa	50 to 54	2010	0·503 (0·200–2·00)	1·22 (0·300–4·30)	0·470 (0·100–2·00)	1·09 (0·200–3·90)
Eastern Sub-Saharan Africa	55 to 59	2010	0·523 (0·300–2·00)	1·38 (0·500–4·10)	0·511 (0·200–2·00)	1·26 (0·400–4·00)
Eastern Sub-Saharan Africa	60 to 64	2010	0·527 (0·300–1·90)	1·45 (0·600–3·80)	0·507 (0·300–1·00)	1·39 (0·600–3·00)
Eastern Sub-Saharan Africa	65 to 69	2010	0·523 (0·400–1·00)	1·50 (0·700–3·20)	0·501 (0·300–1·00)	1·43 (0·698–2·90)
Eastern Sub-Saharan Africa	70 to 74	2010	0·544 (0·400–1·00)	1·69 (0·800–4·00)	0·546 (0·400–2·00)	1·68 (0·700–4·40)
Eastern Sub-Saharan Africa	75 to 79	2010	0·548 (0·400–1·00)	1·79 (0·900–3·80)	0·552 (0·400–1·90)	1·69 (0·800–4·40)
Eastern Sub-Saharan Africa	80 plus	2010	0·565 (0·400–1·00)	1·80 (0·898–4·10)	0·584 (0·400–2·00)	1·89 (0·800–5·50)
Eastern Sub-Saharan Africa	15 to 19	2020	0·154 (0–2·00)	0·270 (0–2·10)	0·0239 (0–0·200)	0·0407 (0–0·400)
Eastern Sub-Saharan Africa	20 to 24	2020	0·269 (0–2·30)	0·499 (0–3·50)	0·0452 (0–0·300)	0·0732 (0–0·500)
Eastern Sub-Saharan Africa	25 to 29	2020	0·304 (0–2·30)	0·583 (0–4·10)	0·0930 (0–1·00)	0·164 (0–1·30)
Eastern Sub-Saharan Africa	30 to 34	2020	0·369 (0–2·10)	0·726 (0–3·50)	0·274 (0–2·10)	0·501 (0–3·20)
Eastern Sub-Saharan Africa	35 to 39	2020	0·408 (0–2·00)	0·851 (0–3·70)	0·313 (0–2·00)	0·595 (0–3·20)
Eastern Sub-Saharan Africa	40 to 44	2020	0·403 (0·100–1·00)	0·890 (0·100–2·30)	0·358 (0–1·00)	0·731 (0–2·30)
Eastern Sub-Saharan Africa	45 to 49	2020	0·437 (0·200–1·00)	1·02 (0·300–2·40)	0·427 (0–2·00)	0·935 (0–2·80)
Eastern Sub-Saharan Africa	50 to 54	2020	0·461 (0·200–1·51)	1·15 (0·400–2·90)	0·457 (0·200–2·00)	1·06 (0·300–2·80)
Eastern Sub-Saharan Africa	55 to 59	2020	0·500 (0·300–1·00)	1·35 (0·600–3·00)	0·492 (0·300–1·51)	1·25 (0·500–3·00)
Eastern Sub-Saharan Africa	60 to 64	2020	0·519 (0·300–1·00)	1·45 (0·700–3·10)	0·506 (0·400–1·00)	1·43 (0·700–2·70)
Eastern Sub-Saharan Africa	65 to 69	2020	0·524 (0·400–1·00)	1·54 (0·800–2·70)	0·504 (0·400–0·900)	1·47 (0·700–2·70)
Eastern Sub-Saharan Africa	70 to 74	2020	0·545 (0·400–1·00)	1·70 (0·900–3·40)	0·543 (0·400–1·00)	1·70 (0·900–3·60)
Eastern Sub-Saharan Africa	75 to 79	2020	0·550 (0·400–1·00)	1·83 (1·00–3·30)	0·547 (0·400–1·00)	1·70 (0·900–3·50)

Eastern Sub-Saharan Africa	80 plus	2020	0·567<br>(0·400–1·00)	1·83<br>(0·900–3·60)	0·582<br>(0·400–1·50)	1·89<br>(0·900–4·40)
Southern Sub-Saharan Africa	15 to 19	1990	0·282<br>(0–3·00)	0·485<br>(0–7·63)	0·0121<br>(0–0)	0·0169<br>(0–0)
Southern Sub-Saharan Africa	20 to 24	1990	0·346<br>(0–5·00)	0·595<br>(0–10·0)	0·0176<br>(0–0)	0·0306<br>(0–0)
Southern Sub-Saharan Africa	25 to 29	1990	0·276<br>(0–3·00)	0·506<br>(0–7·30)	0·0239<br>(0–0·100)	0·0309<br>(0–0·102)
Southern Sub-Saharan Africa	30 to 34	1990	0·270<br>(0–2·60)	0·487<br>(0–5·41)	0·0559<br>(0–0·300)	0·0581<br>(0–0·500)
Southern Sub-Saharan Africa	35 to 39	1990	0·306<br>(0–2·30)	0·582<br>(0–4·01)	0·122<br>(0–1·00)	0·202<br>(0–2·11)
Southern Sub-Saharan Africa	40 to 44	1990	0·365<br>(0–2·00)	0·776<br>(0–3·40)	0·255<br>(0–2·20)	0·480<br>(0–3·70)
Southern Sub-Saharan Africa	45 to 49	1990	0·447<br>(0·200–2·00)	1·10<br>(0·300–3·30)	0·381<br>(0–2·20)	0·807<br>(0–4·71)
Southern Sub-Saharan Africa	50 to 54	1990	0·509<br>(0·300–1·00)	1·32<br>(0·600–3·00)	0·478<br>(0·100–2·10)	1·10<br>(0·200–4·30)
Southern Sub-Saharan Africa	55 to 59	1990	0·555<br>(0·400–1·00)	1·51<br>(0·800–3·10)	0·537<br>(0·200–2·10)	1·36<br>(0·400–4·70)
Southern Sub-Saharan Africa	60 to 64	1990	0·614<br>(0·400–1·00)	1·84<br>(1·00–3·80)	0·622<br>(0·300–2·20)	1·74<br>(0·600–6·80)
Southern Sub-Saharan Africa	65 to 69	1990	0·617<br>(0·500–1·00)	1·97<br>(1·10–3·60)	0·617<br>(0·400–2·00)	1·82<br>(0·700–6·10)
Southern Sub-Saharan Africa	70 to 74	1990	0·617<br>(0·500–1·00)	2·13<br>(1·20–3·80)	0·623<br>(0·400–2·00)	1·99<br>(0·800–6·00)
Southern Sub-Saharan Africa	75 to 79	1990	0·615<br>(0·500–1·00)	2·29<br>(1·40–4·00)	0·628<br>(0·400–2·00)	2·17<br>(0·900–5·70)
Southern Sub-Saharan Africa	80 plus	1990	0·627<br>(0·500–1·00)	2·59<br>(1·40–4·50)	0·642<br>(0·500–1·90)	2·53<br>(1·10–6·00)
Southern Sub-Saharan Africa	15 to 19	2000	0·319<br>(0–3·00)	0·552<br>(0–10·0)	0·0138<br>(0–0·100)	0·0201<br>(0–0·102)
Southern Sub-Saharan Africa	20 to 24	2000	0·426<br>(0–5·00)	0·728<br>(0–10·0)	0·0257<br>(0–0·100)	0·0430<br>(0–0·200)
Southern Sub-Saharan Africa	25 to 29	2000	0·282<br>(0–2·80)	0·516<br>(0–7·80)	0·0354<br>(0–0·200)	0·0533<br>(0–0·400)
Southern Sub-Saharan Africa	30 to 34	2000	0·255<br>(0–2·50)	0·494<br>(0–6·71)	0·0666<br>(0–0·400)	0·0933<br>(0–1·00)
Southern Sub-Saharan Africa	35 to 39	2000	0·301<br>(0–2·30)	0·558<br>(0–4·20)	0·128<br>(0–2·00)	0·210<br>(0–2·50)
Southern Sub-Saharan Africa	40 to 44	2000	0·366<br>(0–2·00)	0·759<br>(0–3·30)	0·274<br>(0–2·20)	0·506<br>(0–3·40)
Southern Sub-Saharan Africa	45 to 49	2000	0·460<br>(0·200–2·00)	1·10<br>(0·300–3·50)	0·403<br>(0–2·10)	0·841<br>(0–4·10)
Southern Sub-Saharan Africa	50 to 54	2000	0·530<br>(0·300–1·00)	1·38<br>(0·600–3·20)	0·480<br>(0·200–2·00)	1·14<br>(0·300–4·20)
Southern Sub-Saharan Africa	55 to 59	2000	0·581<br>(0·400–1·00)	1·60<br>(0·800–3·40)	0·552<br>(0·300–2·00)	1·40<br>(0·500–4·60)
Southern Sub-Saharan Africa	60 to 64	2000	0·638<br>(0·500–1·00)	1·97<br>(1·10–4·20)	0·635<br>(0·300–2·10)	1·78<br>(0·600–6·50)
Southern Sub-Saharan Africa	65 to 69	2000	0·644<br>(0·500–1·00)	2·10<br>(1·20–3·90)	0·619<br>(0·400–2·00)	1·87<br>(0·800–5·70)
Southern Sub-Saharan Africa	70 to 74	2000	0·638<br>(0·500–1·00)	2·27<br>(1·30–4·00)	0·622<br>(0·400–2·00)	2·03<br>(0·900–5·30)
Southern Sub-Saharan Africa	75 to 79	2000	0·631<br>(0·500–1·00)	2·37<br>(1·40–4·00)	0·622<br>(0·400–2·00)	2·17<br>(1·00–5·40)
Southern Sub-Saharan Africa	80 plus	2000	0·637<br>(0·500–1·00)	2·62<br>(1·50–4·50)	0·634<br>(0·500–1·50)	2·46<br>(1·10–5·60)
Southern Sub-Saharan Africa	15 to 19	2010	0·452<br>(0–5·00)	0·793<br>(0–10·0)	0·0248<br>(0–0·100)	0·0374<br>(0–0·200)
Southern Sub-Saharan Africa	20 to 24	2010	0·553<br>(0–7·00)	0·963<br>(0–10·0)	0·0439<br>(0–0·200)	0·0706<br>(0–0·400)
Southern Sub-Saharan Africa	25 to 29	2010	0·420<br>(0–5·00)	0·797<br>(0–10·0)	0·0655<br>(0–0·500)	0·0778<br>(0–0·702)
Southern Sub-Saharan Africa	30 to 34	2010	0·378<br>(0–2·80)	0·732<br>(0–10·0)	0·0867<br>(0–1·00)	0·122<br>(0–1·00)
Southern Sub-Saharan Africa	35 to 39	2010	0·383<br>(0–2·50)	0·769<br>(0–7·60)	0·176<br>(0–2·30)	0·298<br>(0–3·30)
Southern Sub-Saharan Africa	40 to 44	2010	0·442<br>(0–2·30)	0·919<br>(0–5·10)	0·311<br>(0–2·40)	0·585<br>(0–4·90)
Southern Sub-Saharan Africa	45 to 49	2010	0·511<br>(0·200–2·10)	1·22<br>(0·400–4·60)	0·456<br>(0–2·30)	0·967<br>(0–5·71)
Southern Sub-Saharan Africa	50 to 54	2010	0·556<br>(0·300–1·80)	1·46<br>(0·700–3·80)	0·535<br>(0·200–2·20)	1·26<br>(0·300–5·40)
Southern Sub-Saharan Africa	55 to 59	2010	0·612<br>(0·400–1·01)	1·68<br>(0·898–3·80)	0·601<br>(0·300–2·10)	1·52<br>(0·500–5·80)
Southern Sub-Saharan Africa	60 to 64	2010	0·665<br>(0·500–1·00)	2·06<br>(1·00–4·70)	0·667<br>(0·300–2·20)	1·89<br>(0·700–7·00)
Southern Sub-Saharan Africa	65 to 69	2010	0·671<br>(0·500–1·00)	2·19<br>(1·20–4·30)	0·647<br>(0·400–2·00)	1·97<br>(0·800–5·90)
Southern Sub-Saharan Africa	70 to 74	2010	0·657<br>(0·500–1·00)	2·33<br>(1·30–4·10)	0·645<br>(0·400–2·00)	2·12<br>(0·900–5·60)

Southern Sub-Saharan Africa	75 to 79	2010	0·647 (0·500–1·00)	2·45 (1·50–4·10)	0·643 (0·497–1·90)	2·27 (1·00–5·40)
Southern Sub-Saharan Africa	80 plus	2010	0·645 (0·500–1·00)	2·63 (1·50–4·30)	0·652 (0·500–1·50)	2·57 (1·20–5·50)
Southern Sub-Saharan Africa	15 to 19	2020	0·332 (0–3·00)	0·607 (0–10·0)	0·0182 (0–0·100)	0·0269 (0–0·200)
Southern Sub-Saharan Africa	20 to 24	2020	0·431 (0–5·00)	0·798 (0–10·0)	0·0194 (0–0·100)	0·0303 (0–0·100)
Southern Sub-Saharan Africa	25 to 29	2020	0·354 (0–3·00)	0·645 (0–10·0)	0·0215 (0–0·100)	0·0419 (0–0·102)
Southern Sub-Saharan Africa	30 to 34	2020	0·306 (0–2·60)	0·593 (0–7·40)	0·0351 (0–0·200)	0·0434 (0–0·300)
Southern Sub-Saharan Africa	35 to 39	2020	0·321 (0–2·30)	0·599 (0–3·81)	0·112 (0–1·00)	0·178 (0–2·00)
Southern Sub-Saharan Africa	40 to 44	2020	0·382 (0–2·00)	0·800 (0–3·20)	0·270 (0–2·10)	0·492 (0–3·20)
Southern Sub-Saharan Africa	45 to 49	2020	0·493 (0·300–1·00)	1·16 (0·400–3·00)	0·422 (0–2·10)	0·871 (0–3·80)
Southern Sub-Saharan Africa	50 to 54	2020	0·568 (0·400–1·00)	1·46 (0·800–2·80)	0·489 (0·200–2·00)	1·21 (0·400–3·70)
Southern Sub-Saharan Africa	55 to 59	2020	0·630 (0·400–1·00)	1·71 (1·00–3·10)	0·565 (0·300–2·00)	1·49 (0·600–4·20)
Southern Sub-Saharan Africa	60 to 64	2020	0·683 (0·500–1·00)	2·11 (1·20–3·90)	0·640 (0·400–2·00)	1·86 (0·800–5·50)
Southern Sub-Saharan Africa	65 to 69	2020	0·685 (0·500–1·00)	2·27 (1·30–3·70)	0·643 (0·400–1·90)	2·01 (1·00–4·70)
Southern Sub-Saharan Africa	70 to 74	2020	0·676 (0·500–1·00)	2·45 (1·50–3·90)	0·656 (0·500–1·01)	2·21 (1·10–4·70)
Southern Sub-Saharan Africa	75 to 79	2020	0·666 (0·500–1·00)	2·58 (1·60–4·00)	0·658 (0·500–1·00)	2·42 (1·20–4·90)
Southern Sub-Saharan Africa	80 plus	2020	0·661 (0·500–1·00)	2·78 (1·70–4·40)	0·670 (0·500–1·50)	2·79 (1·40–5·30)
Western Sub-Saharan Africa	15 to 19	1990	0·188 (0–2·00)	0·337 (0–2·10)	0·0296 (0–0·200)	0·0450 (0–0·400)
Western Sub-Saharan Africa	20 to 24	1990	0·294 (0–2·30)	0·558 (0–4·90)	0·0735 (0–0·300)	0·109 (0–0·602)
Western Sub-Saharan Africa	25 to 29	1990	0·351 (0–2·40)	0·670 (0–6·30)	0·130 (0–2·00)	0·219 (0–2·30)
Western Sub-Saharan Africa	30 to 34	1990	0·402 (0–2·10)	0·829 (0–3·90)	0·263 (0–2·20)	0·468 (0–3·51)
Western Sub-Saharan Africa	35 to 39	1990	0·437 (0–2·10)	0·945 (0–4·10)	0·304 (0–2·00)	0·569 (0–3·31)
Western Sub-Saharan Africa	40 to 44	1990	0·408 (0·100–1·00)	0·940 (0·200–2·60)	0·398 (0–2·00)	0·816 (0–3·10)
Western Sub-Saharan Africa	45 to 49	1990	0·478 (0·200–2·00)	1·23 (0·400–3·40)	0·470 (0·100–2·00)	1·07 (0·200–3·80)
Western Sub-Saharan Africa	50 to 54	1990	0·501 (0·300–1·90)	1·47 (0·600–3·70)	0·528 (0·200–2·00)	1·32 (0·400–4·71)
Western Sub-Saharan Africa	55 to 59	1990	0·511 (0·300–1·90)	1·49 (0·600–3·70)	0·531 (0·300–2·00)	1·37 (0·500–4·10)
Western Sub-Saharan Africa	60 to 64	1990	0·558 (0·400–1·50)	1·78 (0·800–4·30)	0·503 (0·300–1·00)	1·42 (0·600–3·10)
Western Sub-Saharan Africa	65 to 69	1990	0·562 (0·400–1·01)	1·97 (1·00–4·40)	0·544 (0·400–1·80)	1·66 (0·800–3·70)
Western Sub-Saharan Africa	70 to 74	1990	0·583 (0·400–2·00)	2·03 (0·900–5·10)	0·587 (0·400–2·00)	2·00 (0·800–5·50)
Western Sub-Saharan Africa	75 to 79	1990	0·572 (0·400–1·50)	2·22 (1·10–5·20)	0·615 (0·400–2·00)	2·15 (0·900–6·20)
Western Sub-Saharan Africa	80 plus	1990	0·594 (0·400–1·50)	2·37 (1·10–5·60)	0·635 (0·400–2·00)	2·37 (0·900–6·90)
Western Sub-Saharan Africa	15 to 19	2000	0·170 (0–0·502)	0·312 (0–1·30)	0·0219 (0–0·200)	0·0348 (0–0·400)
Western Sub-Saharan Africa	20 to 24	2000	0·280 (0–2·20)	0·526 (0–3·40)	0·0502 (0–0·300)	0·0800 (0–0·500)
Western Sub-Saharan Africa	25 to 29	2000	0·326 (0–2·30)	0·621 (0–5·00)	0·0867 (0–0·400)	0·145 (0–1·00)
Western Sub-Saharan Africa	30 to 34	2000	0·397 (0–2·00)	0·804 (0–3·40)	0·216 (0–2·00)	0·383 (0–2·90)
Western Sub-Saharan Africa	35 to 39	2000	0·429 (0–2·00)	0·914 (0–3·70)	0·275 (0–2·00)	0·522 (0–2·70)
Western Sub-Saharan Africa	40 to 44	2000	0·411 (0·100–1·00)	0·949 (0·200–2·40)	0·392 (0–2·00)	0·821 (0–2·70)
Western Sub-Saharan Africa	45 to 49	2000	0·479 (0·300–1·90)	1·25 (0·500–3·20)	0·460 (0·100–2·00)	1·09 (0·200–3·50)
Western Sub-Saharan Africa	50 to 54	2000	0·509 (0·300–1·80)	1·49 (0·698–3·60)	0·529 (0·200–2·00)	1·35 (0·400–4·40)
Western Sub-Saharan Africa	55 to 59	2000	0·519 (0·300–1·80)	1·53 (0·700–3·90)	0·537 (0·300–2·00)	1·41 (0·500–4·00)
Western Sub-Saharan Africa	60 to 64	2000	0·569 (0·400–1·50)	1·85 (0·900–4·20)	0·512 (0·300–1·00)	1·49 (0·700–3·10)
Western Sub-Saharan Africa	65 to 69	2000	0·577 (0·400–1·00)	2·05 (1·00–4·50)	0·559 (0·400–1·70)	1·75 (0·800–3·80)

Western Sub-Saharan Africa	70 to 74	2000	0·588 (0·400–1·60)	2·09 (1·00–5·10)	0·591 (0·400–2·00)	2·08 (0·900–5·60)
Western Sub-Saharan Africa	75 to 79	2000	0·584 (0·400–1·50)	2·28 (1·10–5·30)	0·617 (0·400–2·00)	2·23 (0·900–6·10)
Western Sub-Saharan Africa	80 plus	2000	0·608 (0·500–1·50)	2·42 (1·10–5·50)	0·641 (0·400–2·00)	2·47 (1·00–6·60)
Western Sub-Saharan Africa	15 to 19	2010	0·167 (0–0·402)	0·305 (0–1·00)	0·0161 (0–0·200)	0·0260 (0–0·300)
Western Sub-Saharan Africa	20 to 24	2010	0·240 (0–2·00)	0·464 (0–2·40)	0·0322 (0–0·200)	0·0525 (0–0·400)
Western Sub-Saharan Africa	25 to 29	2010	0·277 (0–2·00)	0·529 (0–2·90)	0·0617 (0–0·300)	0·103 (0–0·700)
Western Sub-Saharan Africa	30 to 34	2010	0·354 (0–1·00)	0·740 (0–2·30)	0·155 (0–1·00)	0·281 (0–1·40)
Western Sub-Saharan Africa	35 to 39	2010	0·376 (0–1·00)	0·829 (0–2·40)	0·224 (0–1·00)	0·425 (0–1·90)
Western Sub-Saharan Africa	40 to 44	2010	0·398 (0·100–0·805)	0·920 (0·200–2·10)	0·351 (0–1·00)	0·754 (0–2·20)
Western Sub-Saharan Africa	45 to 49	2010	0·469 (0·300–1·00)	1·20 (0·500–2·50)	0·427 (0·198–1·00)	1·00 (0·298–2·50)
Western Sub-Saharan Africa	50 to 54	2010	0·503 (0·300–1·00)	1·48 (0·700–3·00)	0·489 (0·300–1·90)	1·26 (0·498–3·10)
Western Sub-Saharan Africa	55 to 59	2010	0·516 (0·400–1·00)	1·54 (0·800–3·10)	0·500 (0·300–1·00)	1·35 (0·600–3·00)
Western Sub-Saharan Africa	60 to 64	2010	0·578 (0·400–1·00)	1·90 (1·00–3·80)	0·511 (0·400–1·00)	1·48 (0·700–2·80)
Western Sub-Saharan Africa	65 to 69	2010	0·588 (0·400–1·00)	2·14 (1·10–4·20)	0·552 (0·400–1·00)	1·74 (0·900–3·40)
Western Sub-Saharan Africa	70 to 74	2010	0·588 (0·400–1·00)	2·17 (1·10–4·60)	0·578 (0·400–1·80)	2·06 (1·00–4·80)
Western Sub-Saharan Africa	75 to 79	2010	0·588 (0·500–1·00)	2·43 (1·30–5·00)	0·604 (0·400–1·90)	2·24 (1·00–5·20)
Western Sub-Saharan Africa	80 plus	2010	0·620 (0·500–1·00)	2·60 (1·20–5·30)	0·625 (0·400–2·00)	2·51 (1·10–5·80)
Western Sub-Saharan Africa	15 to 19	2020	0·174 (0–0·400)	0·317 (0–1·00)	0·0169 (0–0·200)	0·0261 (0–0·400)
Western Sub-Saharan Africa	20 to 24	2020	0·221 (0–0·500)	0·423 (0–1·30)	0·0267 (0–0·200)	0·0445 (0–0·400)
Western Sub-Saharan Africa	25 to 29	2020	0·241 (0–1·00)	0·454 (0–2·00)	0·0484 (0–0·300)	0·0837 (0–0·600)
Western Sub-Saharan Africa	30 to 34	2020	0·339 (0–0·600)	0·710 (0–1·70)	0·149 (0–0·402)	0·262 (0–1·00)
Western Sub-Saharan Africa	35 to 39	2020	0·355 (0–0·600)	0·774 (0–2·00)	0·216 (0–0·500)	0·405 (0–1·10)
Western Sub-Saharan Africa	40 to 44	2020	0·397 (0·100–0·700)	0·905 (0·200–1·80)	0·360 (0–1·00)	0·775 (0–2·00)
Western Sub-Saharan Africa	45 to 49	2020	0·467 (0·300–1·00)	1·17 (0·500–2·20)	0·426 (0·200–1·00)	1·03 (0·300–2·30)
Western Sub-Saharan Africa	50 to 54	2020	0·498 (0·400–0·802)	1·46 (0·800–2·60)	0·485 (0·300–1·00)	1·29 (0·500–2·70)
Western Sub-Saharan Africa	55 to 59	2020	0·519 (0·400–1·00)	1·54 (0·800–2·70)	0·512 (0·300–1·00)	1·42 (0·700–2·80)
Western Sub-Saharan Africa	60 to 64	2020	0·591 (0·400–1·00)	1·95 (1·10–3·60)	0·529 (0·400–1·00)	1·59 (0·800–2·70)
Western Sub-Saharan Africa	65 to 69	2020	0·603 (0·500–1·00)	2·23 (1·20–4·00)	0·574 (0·400–1·00)	1·89 (1·00–3·40)
Western Sub-Saharan Africa	70 to 74	2020	0·608 (0·500–1·00)	2·29 (1·20–4·40)	0·583 (0·400–1·00)	2·23 (1·10–4·50)
Western Sub-Saharan Africa	75 to 79	2020	0·604 (0·500–1·00)	2·61 (1·40–5·00)	0·614 (0·500–1·01)	2·44 (1·20–5·20)
Western Sub-Saharan Africa	80 plus	2020	0·633 (0·500–1·00)	2·79 (1·40–5·10)	0·645 (0·500–1·80)	2·78 (1·20–5·80)

Figure S1

Central Europe, Eastern Europe, and Central Asia

Female

	15 to 19	20 to 24	25 to 29	30 to 34	35 to 39	40 to 44	45 to 49	50 to 54	55 to 59	60 to 64	65 to 69	70 to 74	75 to 79	80 plus
Global	11	16	14	13	12	11	10	9	8	7	5	5	4	3
Central Asia	8	14	13	13	12	11	8	6	4	2	1	1	0	0
Armenia	11	15	13	12	10	8	6	4	3	1	0	0	0	0
Azerbaijan	9	10	10	9	8	7	7	5	3	2	1	1	1	0
Georgia	34	30	23	19	16	13	9	5	3	1	1	0	0	0
Kazakhstan	15	33	31	29	26	23	18	13	9	5	3	2	1	1
Kyrgyzstan	3	12	13	12	12	11	8	6	4	2	1	0	0	0
Mongolia	13	31	29	26	24	21	17	13	9	6	3	2	1	1
Tajikistan	1	2	1	1	1	1	1	1	0	0	0	0	0	0
Turkmenistan	8	16	14	14	11	11	9	6	4	2	1	1	0	0
Uzbekistan	4	7	7	7	6	5	4	2	2	1	0	0	0	0
Central Europe	49	60	53	47	42	37	32	27	21	16	10	6	4	2
Albania	25	32	25	21	16	12	10	7	5	4	2	1	1	0
Bosnia and Herzegovina	28	29	24	22	19	17	15	12	9	6	3	2	1	1
Bulgaria	53	61	55	51	45	41	38	32	25	19	11	8	5	2
Croatia	64	62	52	43	39	33	29	25	20	16	10	6	4	2
Czechia	69	80	70	62	57	50	43	37	31	24	16	11	8	4
Hungary	64	63	51	44	38	35	30	25	19	15	10	6	5	2
Montenegro	30	42	37	32	27	23	19	18	14	11	7	5	3	1
North Macedonia	45	46	37	31	25	20	17	14	10	7	4	2	1	1
Poland	50	68	59	53	48	42	36	30	23	16	10	6	4	2
Romania	45	59	53	47	41	36	32	26	21	16	11	7	5	2
Serbia	18	27	24	20	17	16	14	14	11	9	5	3	2	1
Slovakia	60	76	66	58	52	45	39	32	24	17	9	6	4	2
Slovenia	70	71	57	46	38	30	23	18	13	9	5	3	2	1
Eastern Europe	44	65	62	56	48	41	35	26	18	9	4	2	1	0
Belarus	51	73	69	63	55	49	41	33	23	13	6	3	2	1
Estonia	64	84	78	72	62	55	47	37	27	15	6	3	2	1
Latvia	58	75	72	69	61	54	46	37	27	17	7	4	3	1
Lithuania	63	87	81	74	62	53	46	35	25	14	7	4	2	1
Republic of Moldova	51	66	65	60	53	47	41	32	23	14	6	4	2	1
Russian Federation	40	63	61	55	48	41	35	26	17	8	3	1	1	0
Ukraine	55	67	63	56	47	40	33	25	17	9	4	2	1	0

Male

	15 to 19	20 to 24	25 to 29	30 to 34	35 to 39	40 to 44	45 to 49	50 to 54	55 to 59	60 to 64	65 to 69	70 to 74	75 to 79	80 plus
Global	24	41	46	43	41	38	37	34	31	28	24	22	18	13
Central Asia	21	49	54	50	46	41	37	31	26	20	15	12	8	6
Armenia	41	68	70	61	53	46	40	31	25	18	13	9	6	3
Azerbaijan	20	43	49	45	42	39	37	32	30	26	20	17	13	10
Georgia	70	78	78	70	64	57	50	42	35	28	22	18	13	9
Kazakhstan	28	63	68	61	55	47	42	35	29	22	17	14	9	6
Kyrgyzstan	11	44	51	46	43	39	36	29	24	20	13	10	6	3
Mongolia	25	61	67	62	56	51	45	39	33	27	21	17	12	8
Tajikistan	5	27	31	29	25	24	21	17	12	8	4	2	1	0
Turkmenistan	25	55	58	54	50	43	38	31	27	22	17	13	9	6
Uzbekistan	19	47	52	47	45	40	35	29	23	17	12	9	6	4
Central Europe	61	84	86	82	75	70	66	60	56	49	43	35	28	19
Albania	35	58	69	63	57	50	46	41	37	32	26	20	15	8
Bosnia and Herzegovina	60	67	70	66	61	57	54	49	44	38	32	26	20	13
Bulgaria	65	84	88	84	77	72	69	65	61	55	48	41	34	23
Croatia	76	85	85	80	71	64	60	55	50	45	38	33	27	18
Czechia	72	92	92	88	81	75	72	67	63	58	51	44	38	27
Hungary	74	81	83	78	72	67	64	59	54	49	43	36	29	21
Montenegro	45	72	75	71	64	59	55	52	48	43	37	30	24	16
North Macedonia	62	84	85	80	71	64	60	55	50	44	37	30	23	15
Poland	56	91	91	87	79	73	68	62	58	51	43	34	27	18
Romania	69	87	87	83	76	70	67	61	57	50	45	36	29	19
Serbia	42	66	72	69	65	61	58	54	50	44	38	31	25	17
Slovakia	64	87	89	84	77	71	67	62	57	52	47	39	34	23
Slovenia	77	87	87	78	64	54	48	42	37	33	26	20	15	9
Eastern Europe	50	80	83	79	72	66	60	51	43	34	27	20	14	8
Belarus	58	85	88	83	75	68	63	54	45	36	28	22	17	11
Estonia	73	90	92	87	81	74	67	57	48	37	28	22	17	11
Latvia	65	86	91	87	81	75	70	62	54	45	36	30	25	18
Lithuania	63	94	94	89	81	74	68	58	49	38	29	24	18	12
Republic of Moldova	68	85	88	83	77	70	64	55	47	36	29	23	17	11
Russian Federation	46	78	81	78	71	66	60	51	44	36	28	20	14	8
Ukraine	62	83	86	79	71	63	57	47	38	28	22	16	12	7

High−income

Female

	15 to 19	20 to 24	25 to 29	30 to 34	35 to 39	40 to 44	45 to 49	50 to 54	55 to 59	60 to 64	65 to 69	70 to 74	75 to 79	80 plus
Australasia	63	84	82	81	77	73	65	56	48	41	31	23	16	9
Australia	62	84	83	82	78	73	65	56	47	40	30	23	16	9
New Zealand	67	82	78	76	74	71	66	59	51	44	36	27	18	10
High−income Asia Pacific	25	62	58	56	52	48	43	36	30	23	18	16	12	8
Brunei Darussalam	2	3	3	3	2	2	2	2	1	1	1	1	0	0
Japan	24	77	73	70	64	58	51	43	36	27	21	18	13	8
Republic of Korea	26	36	35	34	32	30	28	24	20	17	13	11	9	6
Singapore	25	35	31	29	25	22	18	14	10	7	5	3	2	1
High−income North America	38	65	64	53	44	40	36	31	27	21	17	13	10	6
Canada	54	74	68	57	49	43	39	33	28	23	17	13	10	5
Greenland	44	65	60	51	43	38	34	28	23	19	14	11	8	4
United States of America	36	64	64	53	44	40	36	31	27	21	17	13	10	6
Southern Latin America	69	62	60	55	49	42	37	31	26	22	17	14	11	7
Argentina	75	62	60	55	49	42	36	30	25	21	16	13	10	6
Chile	59	63	61	57	50	45	39	33	28	24	19	16	12	8
Uruguay	38	48	47	44	39	35	31	27	23	20	15	13	10	7
Western Europe	61	73	66	62	60	58	55	48	42	36	28	22	17	11
Andorra	65	76	68	65	63	61	57	50	43	36	28	22	16	10
Austria	55	72	66	61	63	61	56	50	42	36	30	23	17	11
Belgium	71	73	65	64	62	62	58	52	45	37	30	24	17	12
Cyprus	65	65	56	49	48	46	42	35	28	23	16	13	8	5
Denmark	77	82	76	70	69	69	63	56	48	41	33	27	20	12
Finland	64	80	73	69	67	65	61	53	44	36	25	19	13	7
France	64	74	66	62	63	62	58	51	44	37	29	23	17	10
Germany	68	79	73	68	67	66	63	56	50	44	35	28	21	15
Greece	72	77	69	63	59	57	49	40	31	24	17	12	8	4
Iceland	46	80	72	67	65	63	57	49	41	34	25	19	14	8
Ireland	59	82	74	68	67	63	58	49	41	36	26	19	14	8
Israel	41	64	55	50	47	45	39	28	20	15	9	6	4	2
Italy	42	63	54	51	48	46	45	40	36	32	23	24	18	10
Luxembourg	62	76	69	64	65	62	57	53	45	38	30	23	16	10
Malta	74	71	63	54	52	47	42	35	28	23	17	12	8	5
Monaco	68	74	64	59	57	54	49	41	33	26	20	15	11	6
Netherlands	65	71	64	60	59	58	55	50	45	38	32	24	18	12
Norway	69	81	70	65	65	63	59	51	44	35	27	20	14	7
Portugal	42	51	50	48	48	46	41	36	31	27	20	16	11	7
San Marino	64	75	67	62	61	59	54	47	40	34	27	21	16	10
Spain	57	66	58	55	54	53	48	39	32	26	20	15	11	7
Sweden	65	81	71	67	68	67	62	55	48	41	32	24	18	10
Switzerland	60	76	69	64	63	62	57	51	43	37	29	23	18	11
United Kingdom	70	77	70	66	66	64	60	54	46	39	30	23	17	9

Male

	15 to 19	20 to 24	25 to 29	30 to 34	35 to 39	40 to 44	45 to 49	50 to 54	55 to 59	60 to 64	65 to 69	70 to 74	75 to 79	80 plus
Australasia	63	89	91	88	82	74	67	61	57	51	44	37	29	19
Australia	62	90	91	89	83	74	67	62	58	52	45	37	30	19
New Zealand	67	86	89	86	79	70	63	57	55	48	42	34	26	16
High−income Asia Pacific	40	77	77	71	65	60	56	52	49	43	38	35	29	19
Brunei Darussalam	5	6	7	6	5	4	3	3	3	2	2	1	1	0
Japan	39	88	86	79	72	64	60	55	52	45	39	35	29	19
Republic of Korea	42	61	65	61	59	55	53	49	47	43	38	33	29	21
Singapore	35	50	50	44	36	29	25	21	19	15	11	8	5	2
High−income North America	39	74	80	74	63	55	49	44	41	34	29	24	18	12
Canada	59	83	85	79	68	59	54	47	43	37	30	25	19	11
Greenland	41	76	79	74	63	55	50	44	39	33	28	22	17	11
United States of America	37	73	79	74	63	55	49	44	40	34	28	23	18	12
Southern Latin America	75	77	80	78	72	63	56	50	46	41	36	31	26	17
Argentina	80	77	81	78	72	62	55	48	45	40	35	30	24	16
Chile	66	78	81	79	73	65	59	53	49	45	40	35	29	20
Uruguay	54	69	73	70	66	58	51	46	43	38	34	30	25	17
Western Europe	68	85	87	81	74	69	65	61	58	53	47	40	33	24
Andorra	71	87	88	83	75	69	66	60	57	52	45	38	31	21
Austria	67	87	87	82	75	70	66	61	58	52	46	38	32	23
Belgium	72	85	85	79	74	68	65	60	57	52	45	37	30	21
Cyprus	70	89	90	85	77	72	66	59	55	48	42	35	28	18
Denmark	82	90	91	85	75	68	64	59	56	50	43	37	30	20
Finland	62	86	89	84	75	68	65	59	55	47	39	30	22	13
France	73	87	88	82	76	71	68	64	61	56	51	45	38	28
Germany	74	88	89	85	79	74	70	67	64	59	53	47	40	30
Greece	76	88	88	82	74	67	63	57	54	48	41	34	26	17
Iceland	51	88	88	82	73	66	62	56	52	46	39	32	25	16
Ireland	66	87	88	81	74	67	63	57	53	45	39	32	25	17
Israel	60	80	80	69	55	45	39	31	27	21	15	10	6	3
Italy	53	81	84	78	70	65	63	58	56	52	44	43	36	23
Luxembourg	67	87	88	83	77	70	67	63	60	55	51	44	37	28
Malta	72	85	86	80	71	63	58	52	49	41	35	29	22	14
Monaco	68	86	86	78	66	57	52	46	43	36	31	25	19	12
Netherlands	67	84	86	80	72	66	62	58	55	49	43	36	29	20
Norway	73	88	88	80	71	63	59	52	49	43	35	28	20	12
Portugal	57	81	83	79	74	71	68	63	61	55	50	43	37	27
San Marino	71	87	87	81	71	65	61	56	53	48	42	36	30	22
Spain	64	81	84	79	72	68	64	59	55	49	41	34	26	17
Sweden	68	89	90	83	75	67	63	57	53	46	39	31	23	14
Switzerland	66	87	88	83	75	69	65	60	57	51	45	38	31	21
United Kingdom	70	86	86	82	74	69	65	61	58	53	47	39	31	22

Latin America and Caribbean

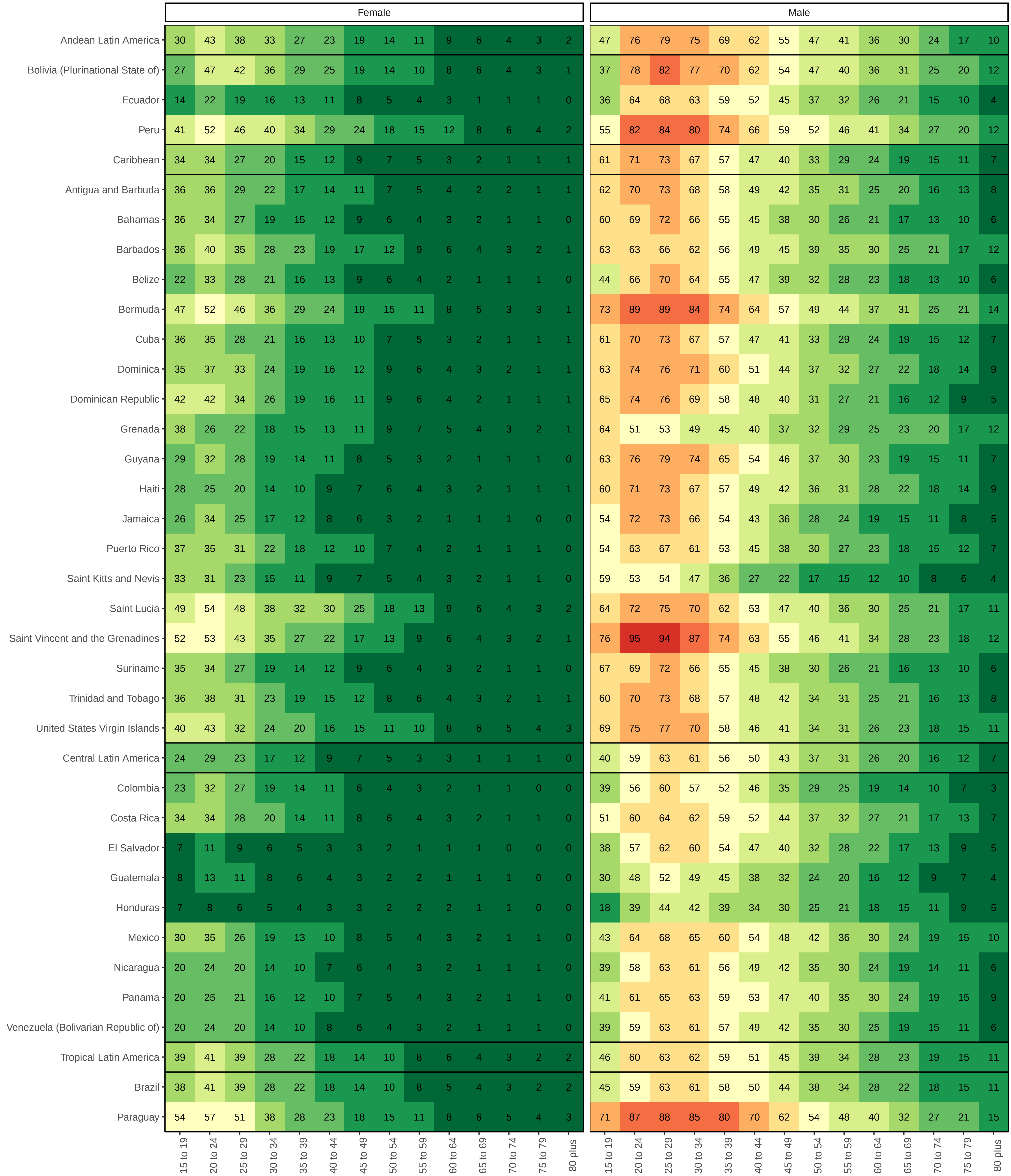

North Africa and Middle East

Female

	15 to 19	20 to 24	25 to 29	30 to 34	35 to 39	40 to 44	45 to 49	50 to 54	55 to 59	60 to 64	65 to 69	70 to 74	75 to 79	80 plus
North Africa and Middle East	0	1	1	1	1	0	0	0	0	0	0	0	0	0
Afghanistan	0	0	0	0	0	0	0	0	0	0	0	0	0	0
Algeria	0	1	1	0	0	0	0	0	0	0	0	0	0	0
Bahrain	0	1	1	0	0	0	0	0	0	0	0	0	0	0
Egypt	0	0	0	0	0	0	0	0	0	0	0	0	0	0
Iran (Islamic Republic of)	0	1	1	0	0	0	0	0	0	0	0	0	0	0
Iraq	0	1	1	1	1	1	1	0	0	0	0	0	0	0
Jordan	0	0	0	0	0	0	0	0	0	0	0	0	0	0
Kuwait	0	0	0	0	0	0	0	0	0	0	0	0	0	0
Lebanon	3	4	4	3	2	1	1	1	0	0	0	0	0	0
Libya	0	0	0	0	0	0	0	0	0	0	0	0	0	0
Morocco	0	0	0	0	0	0	0	0	0	0	0	0	0	0
Oman	0	0	0	0	0	0	0	0	0	0	0	0	0	0
Palestine	0	1	1	1	1	0	0	0	0	0	0	0	0	0
Qatar	0	0	0	0	0	0	0	0	0	0	0	0	0	0
Saudi Arabia	0	0	0	0	0	0	0	0	0	0	0	0	0	0
Sudan	0	0	0	0	0	0	0	0	0	0	0	0	0	0
Syrian Arab Republic	0	1	1	1	0	0	0	0	0	0	0	0	0	0
Tunisia	0	0	0	0	0	0	0	0	0	0	0	0	0	0
Turkey	2	3	3	3	2	2	1	1	1	1	0	0	0	0
United Arab Emirates	0	3	3	2	2	1	1	0	0	0	0	0	0	0
Yemen	0	0	0	0	0	0	0	0	0	0	0	0	0	0

Male

	15 to 19	20 to 24	25 to 29	30 to 34	35 to 39	40 to 44	45 to 49	50 to 54	55 to 59	60 to 64	65 to 69	70 to 74	75 to 79	80 plus
North Africa and Middle East	4	6	7	6	6	5	4	3	3	2	2	2	1	1
Afghanistan	3	4	4	3	2	1	1	0	0	0	0	0	0	0
Algeria	5	6	7	7	6	5	4	3	3	2	2	2	1	1
Bahrain	5	7	8	7	7	5	5	4	3	3	2	2	1	1
Egypt	1	2	3	2	2	2	2	1	1	1	1	0	0	0
Iran (Islamic Republic of)	5	6	8	7	6	5	5	4	3	2	2	2	1	1
Iraq	1	1	1	1	1	1	1	1	1	1	1	0	0	0
Jordan	2	2	2	2	2	2	2	1	1	1	1	1	1	0
Kuwait	1	2	2	2	1	1	0	0	0	0	0	0	0	0
Lebanon	21	19	20	19	16	12	10	7	7	6	4	4	3	2
Libya	4	3	4	3	3	3	2	2	2	1	1	1	1	0
Morocco	6	6	6	6	5	4	3	1	1	1	0	0	0	0
Oman	4	5	6	5	4	3	2	1	1	1	1	1	0	0
Palestine	5	7	9	8	7	7	6	5	4	4	3	2	2	1
Qatar	4	6	7	6	5	4	3	2	2	1	1	1	1	0
Saudi Arabia	2	3	3	2	2	1	1	1	0	0	0	0	0	0
Sudan	2	3	2	1	0	0	0	0	0	0	0	0	0	0
Syrian Arab Republic	3	5	6	6	6	4	4	3	2	2	2	1	1	1
Tunisia	7	18	21	19	16	13	10	8	7	5	4	3	3	2
Turkey	13	18	20	17	16	12	11	8	7	6	5	4	3	2
United Arab Emirates	5	18	21	19	18	13	10	7	5	4	4	3	2	1
Yemen	4	5	6	5	4	3	2	1	1	1	1	1	0	0

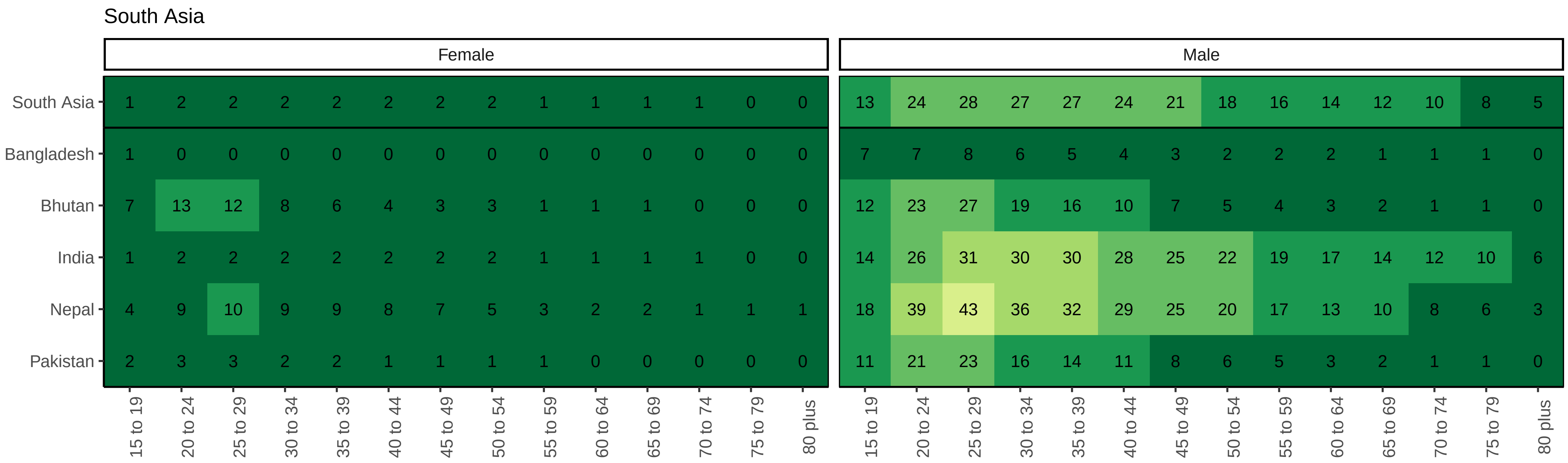
South Asia
Female
Male
South Asia 1 2 2 2 2 2 2 2 1 1 1 1 0 0 | 13 24 28 27 27 24 21 18 16 14 12 10 8 5
Bangladesh 1 0 0 0 0 0 0 0 0 0 0 0 0 0 | 7 7 8 6 5 4 3 2 2 2 1 1 1 0
Bhutan 7 13 12 8 6 4 3 3 1 1 1 0 0 0 | 12 23 27 19 16 10 7 5 4 3 2 1 1 0
India 1 2 2 2 2 2 2 2 1 1 1 1 0 0 | 14 26 31 30 30 28 25 22 19 17 14 12 10 6
Nepal 4 9 10 9 9 8 7 5 3 2 2 1 1 1 | 18 39 43 36 32 29 25 20 17 13 10 8 6 3
Pakistan 2 3 3 2 2 1 1 1 1 0 0 0 0 0 | 11 21 23 16 14 11 8 6 5 3 2 1 1 0
15 to 19
20 to 24
25 to 29
30 to 34
35 to 39
40 to 44
45 to 49
50 to 54
55 to 59
60 to 64
65 to 69
70 to 74
75 to 79
80 plus

Southeast Asia, East Asia, and Oceania

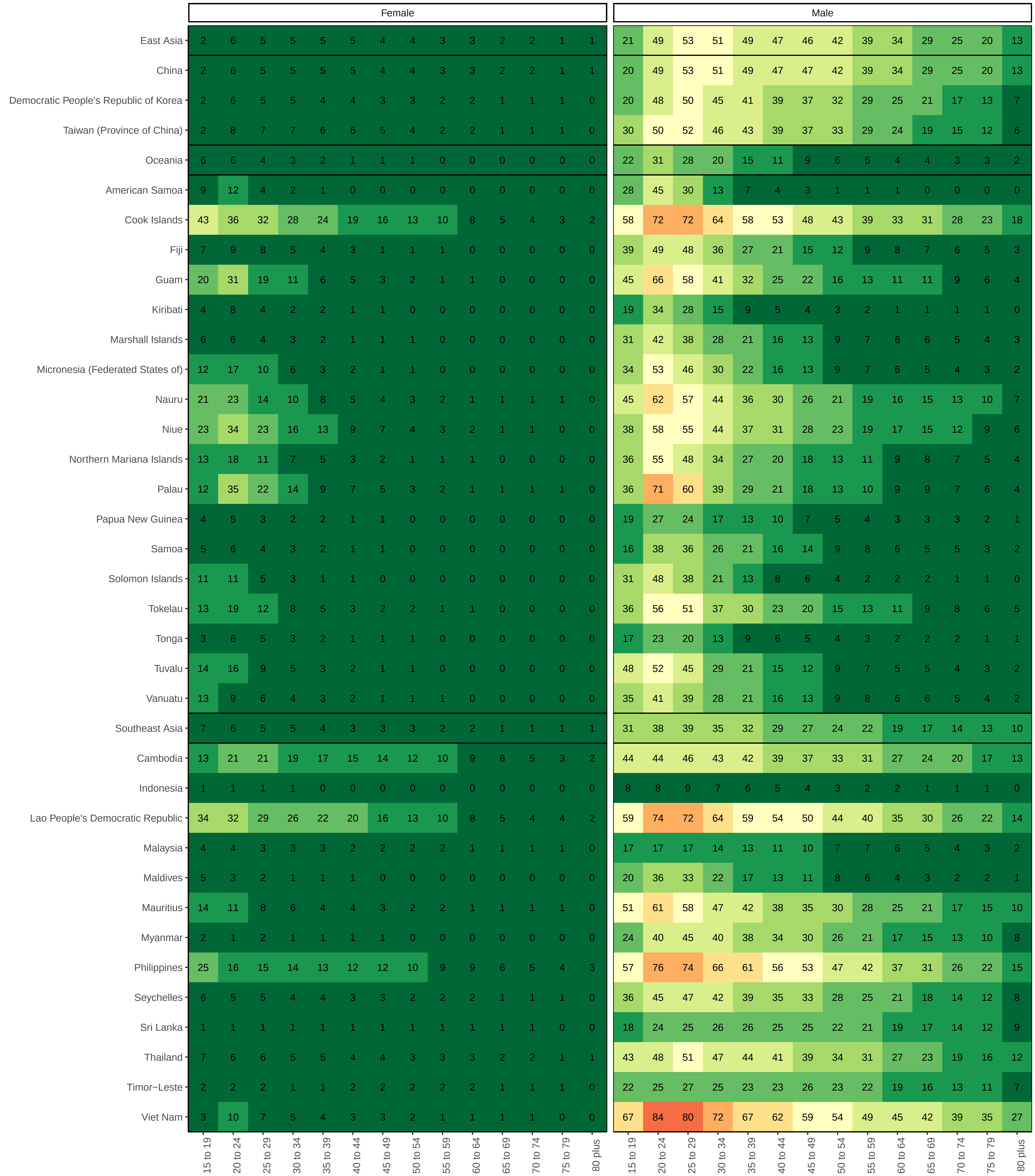

Sub−Saharan Africa

Female

	15 to 19	20 to 24	25 to 29	30 to 34	35 to 39	40 to 44	45 to 49	50 to 54	55 to 59	60 to 64	65 to 69	70 to 74	75 to 79	80 plus
Central Sub−Saharan Africa	13	22	23	20	17	15	13	11	9	7	6	5	4	3
Angola	6	17	20	20	18	16	15	12	10	8	6	5	4	3
Central African Republic	13	22	22	19	15	13	11	9	7	6	5	4	3	2
Congo	43	46	44	39	33	28	25	20	17	13	12	11	10	7
Democratic Republic of the Congo	13	22	22	19	16	13	12	9	8	6	5	5	4	3
Equatorial Guinea	14	24	25	23	20	19	17	16	13	12	10	10	9	7
Gabon	14	24	24	23	21	20	19	16	14	13	11	11	10	7
Eastern Sub−Saharan Africa	13	16	16	14	13	12	12	10	9	9	8	6	4	4
Burundi	6	12	12	11	11	10	10	8	7	6	5	4	3	3
Comoros	8	2	2	1	1	1	1	1	1	1	1	1	0	0
Djibouti	5	7	7	4	3	2	2	1	1	0	0	0	0	0
Eritrea	13	15	15	8	7	4	3	3	1	1	1	1	0	0
Ethiopia	26	28	27	24	21	19	19	16	13	13	11	8	6	4
Kenya	4	8	8	7	7	7	7	8	6	9	8	5	3	3
Madagascar	7	11	11	9	8	7	6	5	4	4	3	2	2	1
Malawi	2	3	3	3	3	3	4	3	3	4	3	2	2	2
Mozambique	15	23	22	16	14	11	10	8	6	5	3	2	2	1
Rwanda	22	27	26	24	22	21	20	17	14	12	9	7	6	4
Somalia	3	3	3	1	1	0	0	0	0	0	0	0	0	0
South Sudan	5	7	7	4	3	2	2	1	1	1	0	0	0	0
Uganda	15	18	18	17	17	16	17	14	13	13	10	7	5	4
United Republic of Tanzania	4	10	10	11	11	13	13	12	12	13	11	10	8	6
Zambia	8	16	17	15	14	14	13	11	8	8	7	6	5	5
Southern Sub−Saharan Africa	10	18	17	18	16	15	13	11	10	9	7	7	6	5
Botswana	15	17	17	13	10	11	9	7	6	5	3	3	2	2
Eswatini	5	7	8	8	7	7	7	6	5	6	5	5	5	5
Lesotho	15	23	22	22	19	17	14	11	9	7	5	4	3	2
Namibia	19	29	31	30	27	26	23	22	20	19	16	14	12	11
South Africa	11	21	19	20	18	17	14	12	11	10	8	7	6	5
Zimbabwe	5	7	7	7	7	6	5	4	4	4	3	3	2	2
Western Sub−Saharan Africa	7	13	14	13	12	13	12	11	11	9	8	7	6	4
Benin	9	15	17	14	14	13	11	9	8	5	3	3	2	1
Burkina Faso	10	12	12	11	12	12	12	12	11	8	6	5	4	3
Cabo Verde	7	15	15	12	12	12	10	8	8	5	3	3	2	2
Cameroon	13	31	34	29	30	28	26	21	19	15	11	11	8	7
Chad	19	26	27	20	20	18	16	12	10	8	5	5	3	2
Côte d'Ivoire	12	20	20	17	17	16	15	14	14	12	10	9	7	6
Gambia	7	13	13	11	11	11	10	8	8	6	4	4	3	2
Ghana	13	21	21	18	17	16	14	11	10	8	6	6	4	3
Guinea	1	2	2	2	2	2	2	1	1	1	1	1	0	0
Guinea−Bissau	7	14	16	15	15	15	14	10	9	7	5	5	3	2
Liberia	10	20	20	17	17	16	16	12	11	8	6	5	4	3
Mali	1	2	2	2	2	2	2	2	2	2	2	2	2	1
Mauritania	2	1	1	0	0	0	0	0	0	0	0	0	0	0
Niger	4	7	8	4	4	3	2	1	1	1	0	0	0	0
Nigeria	5	11	11	12	11	12	12	13	13	12	11	10	8	6
Sao Tome and Principe	8	14	15	14	14	14	13	12	11	10	7	7	6	4
Senegal	4	8	8	5	5	4	3	2	1	1	0	0	0	0
Sierra Leone	1	6	7	7	7	7	7	7	7	6	4	4	3	2
Togo	15	17	19	15	15	14	12	10	10	6	4	4	3	2

Male

	15 to 19	20 to 24	25 to 29	30 to 34	35 to 39	40 to 44	45 to 49	50 to 54	55 to 59	60 to 64	65 to 69	70 to 74	75 to 79	80 plus
Central Sub−Saharan Africa	22	46	53	51	48	41	35	29	26	23	21	18	16	10
Angola	16	45	53	56	54	49	44	40	36	32	28	24	20	15
Central African Republic	22	45	51	48	44	35	30	23	21	17	15	13	11	7
Congo	50	60	66	62	57	51	48	41	38	33	32	29	28	20
Democratic Republic of the Congo	23	45	52	49	45	37	31	25	23	19	17	14	13	8
Equatorial Guinea	26	50	57	56	53	49	45	41	38	35	33	30	28	21
Gabon	27	51	58	57	55	51	47	43	41	37	35	33	31	23
Eastern Sub−Saharan Africa	26	41	45	39	38	35	33	30	29	26	24	21	19	13
Burundi	24	42	48	44	44	42	39	36	34	31	28	24	21	15
Comoros	17	6	7	6	6	5	5	4	4	3	3	3	2	2
Djibouti	15	29	32	20	16	10	7	5	3	2	1	1	1	0
Eritrea	26	39	44	34	31	25	22	18	15	13	11	9	7	4
Ethiopia	44	52	54	46	44	41	38	35	34	30	27	23	20	14
Kenya	18	48	55	51	50	46	44	41	41	38	36	29	24	18
Madagascar	20	36	41	34	32	28	25	21	19	15	13	11	9	6
Malawi	12	32	38	36	33	31	28	26	24	20	17	15	14	10
Mozambique	32	57	59	48	43	36	32	27	24	19	17	13	11	7
Rwanda	45	52	57	53	51	49	47	44	41	36	32	28	24	18
Somalia	6	13	14	6	4	1	1	0	0	0	0	0	0	0
South Sudan	15	28	32	20	16	10	7	5	3	2	1	1	1	0
Uganda	30	41	47	45	45	44	43	40	38	35	30	25	21	16
United Republic of Tanzania	7	22	26	27	28	29	28	28	26	25	26	26	23	17
Zambia	24	46	53	49	47	44	42	37	35	30	27	24	21	15
Southern Sub−Saharan Africa	27	48	53	55	53	48	43	40	38	34	30	26	22	16
Botswana	22	39	44	43	41	36	32	28	24	20	16	14	11	8
Eswatini	15	28	34	33	32	29	28	26	24	22	20	18	15	12
Lesotho	29	51	58	60	57	50	44	38	34	28	23	19	15	10
Namibia	37	55	60	59	57	52	46	40	38	35	32	29	26	19
South Africa	27	48	53	55	53	48	44	41	39	35	31	27	23	16
Zimbabwe	26	50	58	60	58	51	43	37	33	29	26	22	19	13
Western Sub−Saharan Africa	18	34	40	38	37	33	31	29	28	26	24	22	19	15
Benin	17	34	41	38	36	30	28	24	22	19	14	12	9	6
Burkina Faso	38	31	34	32	33	31	31	29	30	29	31	31	31	25
Cabo Verde	44	73	75	70	65	57	53	48	47	42	36	31	26	18
Cameroon	31	58	64	60	59	54	51	46	43	39	34	29	26	20
Chad	37	50	55	48	44	36	32	27	23	19	16	14	10	7
Côte d'Ivoire	36	51	57	53	52	46	44	40	40	38	35	32	28	22
Gambia	18	36	42	39	37	32	30	27	26	23	20	17	15	10
Ghana	21	43	52	50	49	44	42	38	37	33	28	24	21	15
Guinea	16	32	37	33	30	24	21	18	17	14	11	9	7	4
Guinea−Bissau	15	33	40	39	38	33	31	27	26	23	20	17	14	10
Liberia	22	46	49	47	43	38	36	30	28	25	20	17	14	9
Mali	5	6	8	7	7	7	7	7	7	7	7	6	6	5
Mauritania	6	10	10	5	2	0	0	0	0	0	0	0	0	0
Niger	13	27	31	24	18	10	7	4	4	2	1	1	1	0
Nigeria	13	30	36	36	36	34	33	32	31	30	29	28	24	18
Sao Tome and Principe	21	40	47	44	44	40	39	37	36	34	30	27	24	18
Senegal	19	29	33	26	21	13	10	7	6	4	3	2	1	1
Sierra Leone	6	22	27	27	27	25	25	25	24	21	18	16	13	10
Togo	23	33	37	34	32	27	24	22	21	17	14	11	9	6

Figure S2

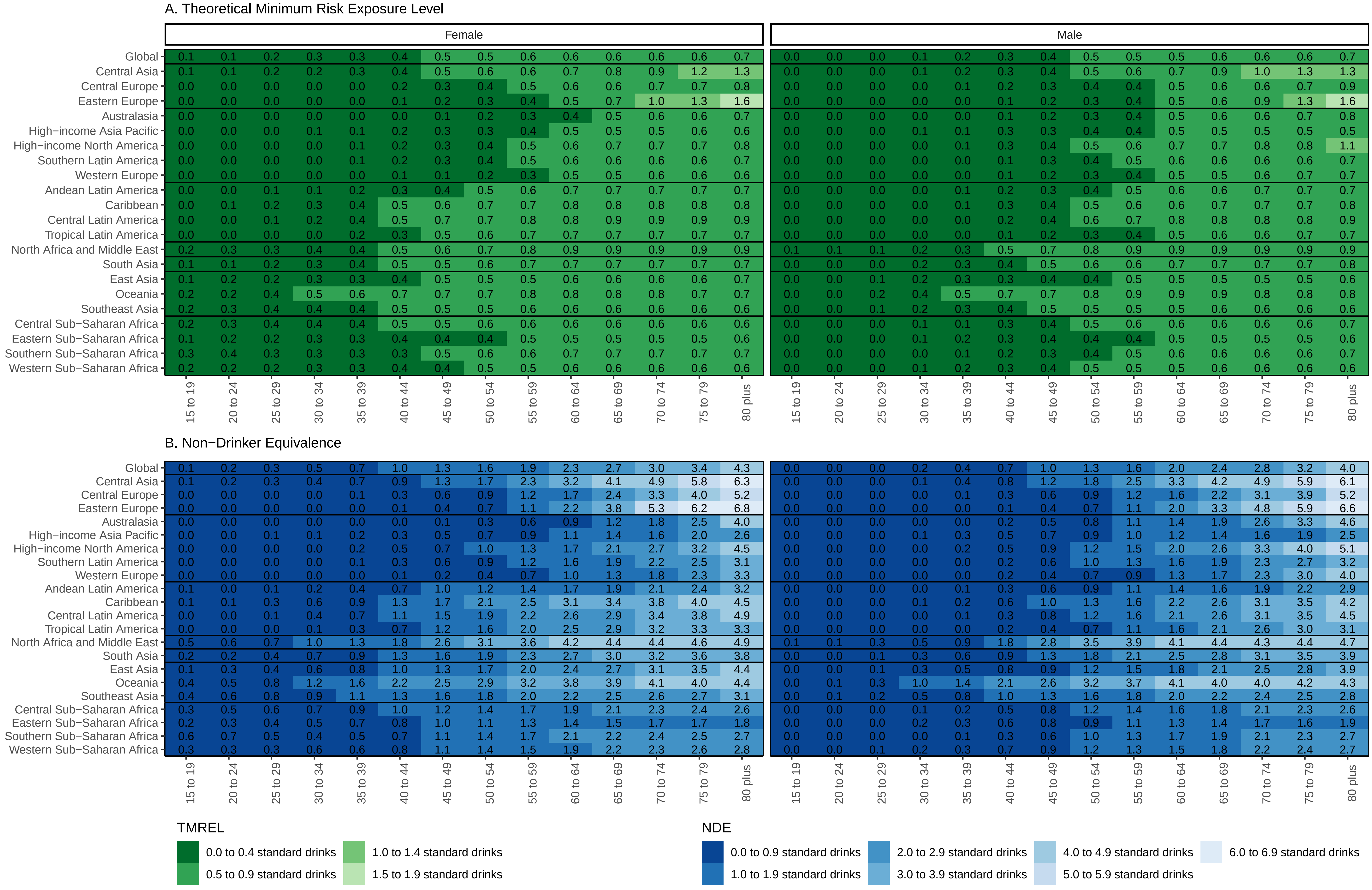
A. Theoretical Minimum Risk Exposure Level
Female
Male
Global
Central Asia
Central Europe
Eastern Europe
Australasia
High−income Asia Pacific
High−income North America
Southern Latin America
Western Europe
Andean Latin America
Caribbean
Central Latin America
Tropical Latin America
North Africa and Middle East
South Asia
East Asia
Oceania
Southeast Asia
Central Sub−Saharan Africa
Eastern Sub−Saharan Africa
Southern Sub−Saharan Africa
Western Sub−Saharan Africa
15 to 19
20 to 24
25 to 29
30 to 34
35 to 39
40 to 44
45 to 49
50 to 54
55 to 59
60 to 64
65 to 69
70 to 74
75 to 79
80 plus
B. Non−Drinker Equivalence
TMREL
0.0 to 0.4 standard drinks
0.5 to 0.9 standard drinks
1.0 to 1.4 standard drinks
1.5 to 1.9 standard drinks
NDE
0.0 to 0.9 standard drinks
1.0 to 1.9 standard drinks
2.0 to 2.9 standard drinks
3.0 to 3.9 standard drinks
4.0 to 4.9 standard drinks
5.0 to 5.9 standard drinks
6.0 to 6.9 standard drinks

Figure S3

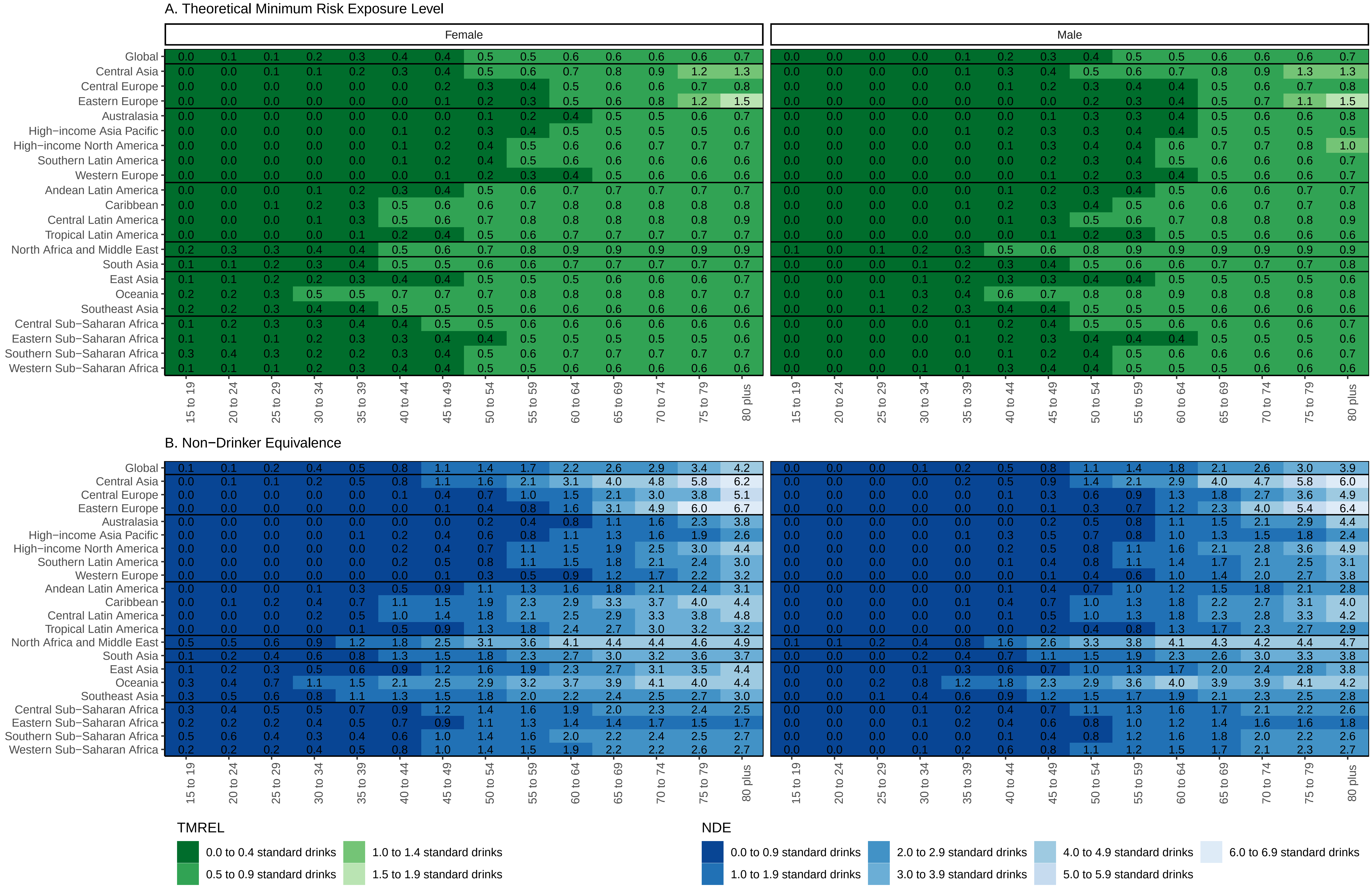
A. Theoretical Minimum Risk Exposure Level
Female
Male
Global
Central Asia
Central Europe
Eastern Europe
Australasia
High−income Asia Pacific
High−income North America
Southern Latin America
Western Europe
Andean Latin America
Caribbean
Central Latin America
Tropical Latin America
North Africa and Middle East
South Asia
East Asia
Oceania
Southeast Asia
Central Sub−Saharan Africa
Eastern Sub−Saharan Africa
Southern Sub−Saharan Africa
Western Sub−Saharan Africa
15 to 19
20 to 24
25 to 29
30 to 34
35 to 39
40 to 44
45 to 49
50 to 54
55 to 59
60 to 64
65 to 69
70 to 74
75 to 79
80 plus
B. Non−Drinker Equivalence
TMREL
0.0 to 0.4 standard drinks
0.5 to 0.9 standard drinks
1.0 to 1.4 standard drinks
1.5 to 1.9 standard drinks
NDE
0.0 to 0.9 standard drinks
1.0 to 1.9 standard drinks
2.0 to 2.9 standard drinks
3.0 to 3.9 standard drinks
4.0 to 4.9 standard drinks
5.0 to 5.9 standard drinks
6.0 to 6.9 standard drinks

Figure S4

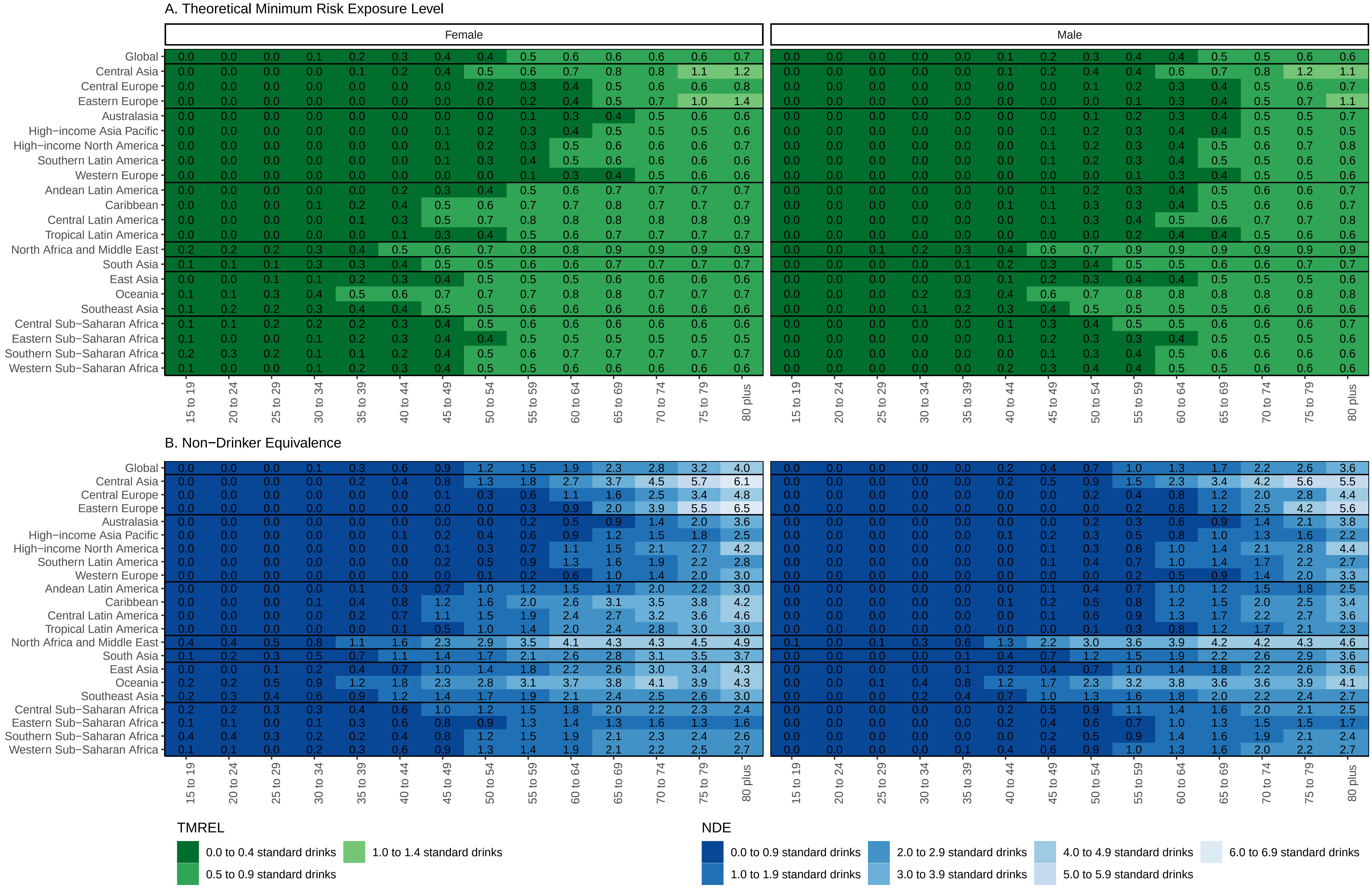
A. Theoretical Minimum Risk Exposure Level
Female
Global 0.0 0.0 0.0 0.1 0.2 0.3 0.4 0.4 0.5 0.6 0.6 0.6 0.6 0.7
Central Asia 0.0 0.0 0.0 0.0 0.1 0.2 0.4 0.5 0.6 0.7 0.8 0.8 1.1 1.2
Central Europe 0.0 0.0 0.0 0.0 0.0 0.0 0.0 0.2 0.3 0.4 0.5 0.6 0.6 0.8
Eastern Europe 0.0 0.0 0.0 0.0 0.0 0.0 0.0 0.0 0.2 0.4 0.5 0.7 1.0 1.4
Australasia 0.0 0.0 0.0 0.0 0.0 0.0 0.0 0.0 0.1 0.3 0.4 0.5 0.6 0.6
High−income Asia Pacific 0.0 0.0 0.0 0.0 0.0 0.0 0.1 0.2 0.3 0.4 0.5 0.5 0.5 0.6
High−income North America 0.0 0.0 0.0 0.0 0.0 0.0 0.1 0.2 0.3 0.5 0.6 0.6 0.6 0.7
Southern Latin America 0.0 0.0 0.0 0.0 0.0 0.0 0.1 0.3 0.4 0.5 0.6 0.6 0.6 0.6
Western Europe 0.0 0.0 0.0 0.0 0.0 0.0 0.0 0.0 0.1 0.3 0.4 0.5 0.6 0.6
Andean Latin America 0.0 0.0 0.0 0.0 0.0 0.2 0.3 0.4 0.5 0.6 0.7 0.7 0.7 0.7
Caribbean 0.0 0.0 0.0 0.1 0.2 0.4 0.5 0.6 0.7 0.7 0.8 0.7 0.7 0.7
Central Latin America 0.0 0.0 0.0 0.0 0.1 0.3 0.5 0.7 0.8 0.8 0.8 0.8 0.8 0.9
Tropical Latin America 0.0 0.0 0.0 0.0 0.0 0.1 0.3 0.4 0.5 0.6 0.7 0.7 0.7 0.7
North Africa and Middle East 0.2 0.2 0.2 0.3 0.4 0.5 0.6 0.7 0.8 0.8 0.9 0.9 0.9 0.9
South Asia 0.1 0.1 0.1 0.3 0.3 0.4 0.5 0.5 0.6 0.6 0.7 0.7 0.7 0.7
East Asia 0.0 0.0 0.1 0.1 0.2 0.3 0.4 0.5 0.5 0.5 0.6 0.6 0.6 0.6
Oceania 0.1 0.1 0.3 0.4 0.5 0.6 0.7 0.7 0.7 0.8 0.8 0.7 0.7 0.7
Southeast Asia 0.1 0.2 0.2 0.3 0.4 0.4 0.5 0.5 0.6 0.6 0.6 0.6 0.6 0.6
Central Sub−Saharan Africa 0.1 0.1 0.2 0.2 0.2 0.3 0.4 0.5 0.6 0.6 0.6 0.6 0.6 0.6
Eastern Sub−Saharan Africa 0.1 0.0 0.0 0.1 0.2 0.3 0.4 0.4 0.5 0.5 0.5 0.5 0.5 0.5
Southern Sub−Saharan Africa 0.2 0.3 0.2 0.1 0.1 0.2 0.4 0.5 0.6 0.7 0.7 0.7 0.7 0.7
Western Sub−Saharan Africa 0.1 0.0 0.0 0.1 0.2 0.3 0.4 0.5 0.5 0.6 0.6 0.6 0.6 0.6
15 to 19 20 to 24 25 to 29 30 to 34 35 to 39 40 to 44 45 to 49 50 to 54 55 to 59 60 to 64 65 to 69 70 to 74 75 to 79 80 plus
Male
Global 0.0 0.0 0.0 0.0 0.0 0.1 0.2 0.3 0.4 0.4 0.5 0.5 0.6 0.6
Central Asia 0.0 0.0 0.0 0.0 0.0 0.1 0.2 0.4 0.4 0.6 0.7 0.8 1.2 1.1
Central Europe 0.0 0.0 0.0 0.0 0.0 0.0 0.0 0.1 0.2 0.3 0.4 0.5 0.6 0.7
Eastern Europe 0.0 0.0 0.0 0.0 0.0 0.0 0.0 0.0 0.1 0.3 0.4 0.5 0.7 1.1
Australasia 0.0 0.0 0.0 0.0 0.0 0.0 0.0 0.1 0.2 0.3 0.4 0.5 0.5 0.7
High−income Asia Pacific 0.0 0.0 0.0 0.0 0.0 0.0 0.1 0.2 0.3 0.4 0.4 0.5 0.5 0.5
High−income North America 0.0 0.0 0.0 0.0 0.0 0.0 0.1 0.2 0.3 0.4 0.5 0.6 0.7 0.8
Southern Latin America 0.0 0.0 0.0 0.0 0.0 0.0 0.1 0.2 0.3 0.4 0.5 0.5 0.6 0.6
Western Europe 0.0 0.0 0.0 0.0 0.0 0.0 0.0 0.0 0.1 0.3 0.4 0.5 0.5 0.6
Andean Latin America 0.0 0.0 0.0 0.0 0.0 0.0 0.1 0.2 0.3 0.4 0.5 0.6 0.6 0.7
Caribbean 0.0 0.0 0.0 0.0 0.0 0.1 0.1 0.3 0.3 0.4 0.5 0.6 0.6 0.7
Central Latin America 0.0 0.0 0.0 0.0 0.0 0.0 0.1 0.3 0.4 0.5 0.6 0.7 0.7 0.8
Tropical Latin America 0.0 0.0 0.0 0.0 0.0 0.0 0.0 0.0 0.2 0.4 0.4 0.5 0.6 0.6
North Africa and Middle East 0.0 0.0 0.1 0.2 0.3 0.4 0.6 0.7 0.9 0.9 0.9 0.9 0.9 0.9
South Asia 0.0 0.0 0.0 0.0 0.1 0.2 0.3 0.4 0.5 0.5 0.6 0.6 0.7 0.7
East Asia 0.0 0.0 0.0 0.0 0.0 0.1 0.2 0.3 0.4 0.4 0.5 0.5 0.5 0.6
Oceania 0.0 0.0 0.0 0.2 0.3 0.4 0.6 0.7 0.8 0.8 0.8 0.8 0.8 0.8
Southeast Asia 0.0 0.0 0.0 0.1 0.2 0.3 0.4 0.5 0.5 0.5 0.5 0.6 0.6 0.6
Central Sub−Saharan Africa 0.0 0.0 0.0 0.0 0.0 0.1 0.3 0.4 0.5 0.5 0.6 0.6 0.6 0.7
Eastern Sub−Saharan Africa 0.0 0.0 0.0 0.0 0.0 0.1 0.2 0.3 0.3 0.4 0.5 0.5 0.5 0.6
Southern Sub−Saharan Africa 0.0 0.0 0.0 0.0 0.0 0.0 0.1 0.3 0.4 0.5 0.6 0.6 0.6 0.6
Western Sub−Saharan Africa 0.0 0.0 0.0 0.0 0.0 0.2 0.3 0.4 0.4 0.5 0.5 0.6 0.6 0.6
15 to 19 20 to 24 25 to 29 30 to 34 35 to 39 40 to 44 45 to 49 50 to 54 55 to 59 60 to 64 65 to 69 70 to 74 75 to 79 80 plus
B. Non−Drinker Equivalence
Female
Global 0.0 0.0 0.0 0.1 0.3 0.6 0.9 1.2 1.5 1.9 2.3 2.8 3.2 4.0
Central Asia 0.0 0.0 0.0 0.0 0.2 0.4 0.8 1.3 1.8 2.7 3.7 4.5 5.7 6.1
Central Europe 0.0 0.0 0.0 0.0 0.0 0.0 0.1 0.3 0.6 1.1 1.6 2.5 3.4 4.8
Eastern Europe 0.0 0.0 0.0 0.0 0.0 0.0 0.0 0.0 0.3 0.9 2.0 3.9 5.5 6.5
Australasia 0.0 0.0 0.0 0.0 0.0 0.0 0.0 0.0 0.2 0.5 0.9 1.4 2.0 3.6
High−income Asia Pacific 0.0 0.0 0.0 0.0 0.0 0.1 0.2 0.4 0.6 0.9 1.2 1.5 1.8 2.5
High−income North America 0.0 0.0 0.0 0.0 0.0 0.0 0.1 0.3 0.7 1.1 1.5 2.1 2.7 4.2
Southern Latin America 0.0 0.0 0.0 0.0 0.0 0.0 0.2 0.5 0.9 1.3 1.6 1.9 2.2 2.8
Western Europe 0.0 0.0 0.0 0.0 0.0 0.0 0.0 0.1 0.2 0.6 1.0 1.4 2.0 3.0
Andean Latin America 0.0 0.0 0.0 0.0 0.1 0.3 0.7 1.0 1.2 1.5 1.7 2.0 2.2 3.0
Caribbean 0.0 0.0 0.0 0.1 0.4 0.8 1.2 1.6 2.0 2.6 3.1 3.5 3.8 4.2
Central Latin America 0.0 0.0 0.0 0.0 0.2 0.7 1.1 1.5 1.9 2.4 2.7 3.2 3.6 4.6
Tropical Latin America 0.0 0.0 0.0 0.0 0.0 0.1 0.5 1.0 1.4 2.0 2.4 2.8 3.0 3.0
North Africa and Middle East 0.4 0.4 0.5 0.8 1.1 1.6 2.3 2.9 3.5 4.1 4.3 4.3 4.5 4.9
South Asia 0.1 0.2 0.3 0.5 0.7 1.1 1.4 1.7 2.1 2.6 2.8 3.1 3.5 3.7
East Asia 0.0 0.0 0.1 0.2 0.4 0.7 1.0 1.4 1.8 2.2 2.6 3.0 3.4 4.3
Oceania 0.2 0.2 0.5 0.9 1.2 1.8 2.3 2.8 3.1 3.7 3.8 4.1 3.9 4.3
Southeast Asia 0.2 0.3 0.4 0.6 0.9 1.2 1.4 1.7 1.9 2.1 2.4 2.5 2.6 3.0
Central Sub−Saharan Africa 0.2 0.2 0.3 0.3 0.4 0.6 1.0 1.2 1.5 1.8 2.0 2.2 2.3 2.4
Eastern Sub−Saharan Africa 0.1 0.1 0.0 0.1 0.3 0.6 0.8 0.9 1.3 1.4 1.3 1.6 1.3 1.6
Southern Sub−Saharan Africa 0.4 0.4 0.3 0.2 0.2 0.4 0.8 1.2 1.5 1.9 2.1 2.3 2.4 2.6
Western Sub−Saharan Africa 0.1 0.1 0.0 0.2 0.3 0.6 0.9 1.3 1.4 1.9 2.1 2.2 2.5 2.7
15 to 19 20 to 24 25 to 29 30 to 34 35 to 39 40 to 44 45 to 49 50 to 54 55 to 59 60 to 64 65 to 69 70 to 74 75 to 79 80 plus
Male
Global 0.0 0.0 0.0 0.0 0.0 0.2 0.4 0.7 1.0 1.3 1.7 2.2 2.6 3.6
Central Asia 0.0 0.0 0.0 0.0 0.0 0.2 0.5 0.9 1.5 2.3 3.4 4.2 5.6 5.5
Central Europe 0.0 0.0 0.0 0.0 0.0 0.0 0.0 0.2 0.4 0.8 1.2 2.0 2.8 4.4
Eastern Europe 0.0 0.0 0.0 0.0 0.0 0.0 0.0 0.0 0.2 0.6 1.2 2.5 4.2 5.6
Australasia 0.0 0.0 0.0 0.0 0.0 0.0 0.0 0.2 0.3 0.6 0.9 1.4 2.1 3.8
High−income Asia Pacific 0.0 0.0 0.0 0.0 0.0 0.1 0.2 0.3 0.5 0.8 1.0 1.3 1.6 2.2
High−income North America 0.0 0.0 0.0 0.0 0.0 0.0 0.1 0.3 0.6 1.0 1.4 2.1 2.8 4.4
Southern Latin America 0.0 0.0 0.0 0.0 0.0 0.0 0.1 0.4 0.7 1.0 1.4 1.7 2.2 2.7
Western Europe 0.0 0.0 0.0 0.0 0.0 0.0 0.0 0.0 0.2 0.5 0.9 1.4 2.0 3.3
Andean Latin America 0.0 0.0 0.0 0.0 0.0 0.0 0.1 0.4 0.7 1.0 1.2 1.5 1.8 2.5
Caribbean 0.0 0.0 0.0 0.0 0.0 0.1 0.2 0.5 0.8 1.2 1.5 2.0 2.5 3.4
Central Latin America 0.0 0.0 0.0 0.0 0.0 0.0 0.1 0.6 0.9 1.3 1.7 2.2 2.7 3.6
Tropical Latin America 0.0 0.0 0.0 0.0 0.0 0.0 0.0 0.1 0.3 0.8 1.2 1.7 2.1 2.3
North Africa and Middle East 0.1 0.0 0.1 0.3 0.6 1.3 2.2 3.0 3.6 3.9 4.2 4.2 4.3 4.6
South Asia 0.0 0.0 0.0 0.0 0.1 0.4 0.7 1.2 1.5 1.9 2.2 2.6 2.9 3.6
East Asia 0.0 0.0 0.0 0.0 0.1 0.2 0.4 0.7 1.0 1.4 1.8 2.2 2.6 3.6
Oceania 0.0 0.0 0.1 0.4 0.8 1.2 1.7 2.3 3.2 3.8 3.6 3.6 3.9 4.1
Southeast Asia 0.0 0.0 0.0 0.2 0.4 0.7 1.0 1.3 1.6 1.8 2.0 2.2 2.4 2.7
Central Sub−Saharan Africa 0.0 0.0 0.0 0.0 0.0 0.2 0.5 0.9 1.1 1.4 1.6 2.0 2.1 2.5
Eastern Sub−Saharan Africa 0.0 0.0 0.0 0.0 0.0 0.2 0.4 0.6 0.7 1.0 1.3 1.5 1.5 1.7
Southern Sub−Saharan Africa 0.0 0.0 0.0 0.0 0.0 0.0 0.2 0.5 0.9 1.4 1.6 1.9 2.1 2.4
Western Sub−Saharan Africa 0.0 0.0 0.0 0.0 0.1 0.4 0.6 0.9 1.0 1.3 1.6 2.0 2.2 2.7
15 to 19 20 to 24 25 to 29 30 to 34 35 to 39 40 to 44 45 to 49 50 to 54 55 to 59 60 to 64 65 to 69 70 to 74 75 to 79 80 plus
TMREL
0.0 to 0.4 standard drinks
0.5 to 0.9 standard drinks
1.0 to 1.4 standard drinks
NDE
0.0 to 0.9 standard drinks
1.0 to 1.9 standard drinks
2.0 to 2.9 standard drinks
3.0 to 3.9 standard drinks
4.0 to 4.9 standard drinks
5.0 to 5.9 standard drinks
6.0 to 6.9 standard drinks

Figure S5

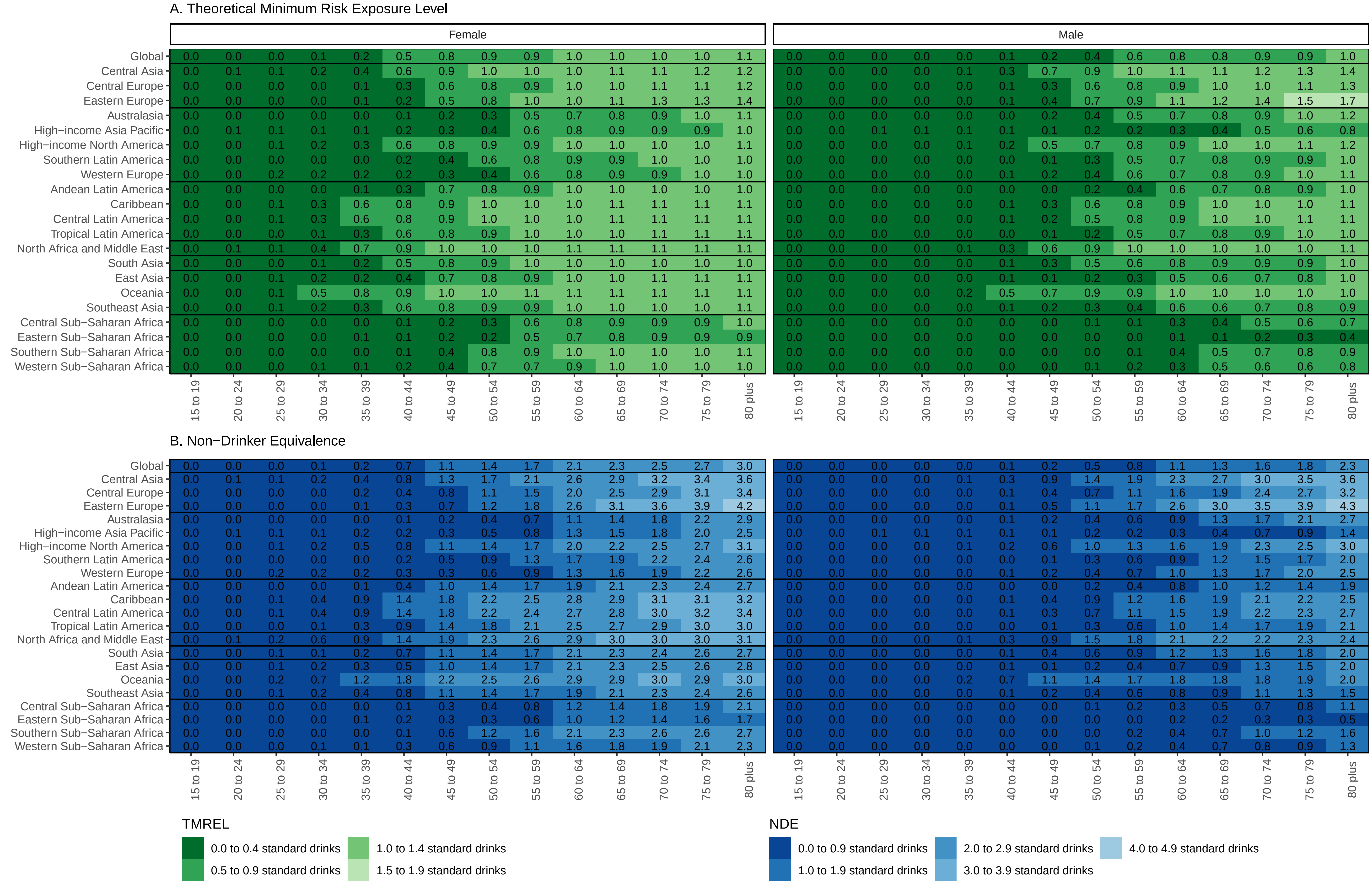
A. Theoretical Minimum Risk Exposure Level
Female
Male
Global
Central Asia
Central Europe
Eastern Europe
Australasia
High−income Asia Pacific
High−income North America
Southern Latin America
Western Europe
Andean Latin America
Caribbean
Central Latin America
Tropical Latin America
North Africa and Middle East
South Asia
East Asia
Oceania
Southeast Asia
Central Sub−Saharan Africa
Eastern Sub−Saharan Africa
Southern Sub−Saharan Africa
Western Sub−Saharan Africa
15 to 19
20 to 24
25 to 29
30 to 34
35 to 39
40 to 44
45 to 49
50 to 54
55 to 59
60 to 64
65 to 69
70 to 74
75 to 79
80 plus
B. Non−Drinker Equivalence
TMREL
0.0 to 0.4 standard drinks
0.5 to 0.9 standard drinks
1.0 to 1.4 standard drinks
1.5 to 1.9 standard drinks
NDE
0.0 to 0.9 standard drinks
1.0 to 1.9 standard drinks
2.0 to 2.9 standard drinks
3.0 to 3.9 standard drinks
4.0 to 4.9 standard drinks

Supplementary Table 2

Location	Age Group	Female GBD 2016	Female GBD 2020	Male GBD 2016	Male GBD 2020
Global	15 to 19	0 (0–0)	0·09 (0–0·4)	0 (0–0)	0·01 (0–0·2)
Global	20 to 24	0·01 (0–0)	0·15 (0–0·4)	0 (0–0)	0·02 (0–0·3)
Global	25 to 29	0·02 (0–0·5)	0·24 (0–0·5)	0 (0–0)	0·06 (0–0·3)
Global	30 to 34	0·06 (0–1)	0·32 (0–0·6)	0·01 (0–0)	0·21 (0–0·4)
Global	35 to 39	0·15 (0–1)	0·38 (0·1–0·6)	0·01 (0–0)	0·31 (0–0·6)
Global	40 to 44	0·41 (0–1)	0·45 (0·3–0·7)	0·05 (0–1)	0·41 (0·2–0·8)
Global	45 to 49	0·71 (0–1)	0·5 (0·4–0·7)	0·11 (0–1)	0·47 (0·3–1)
Global	50 to 54	0·85 (0–1)	0·55 (0·4–0·8)	0·22 (0–1)	0·52 (0·4–1)
Global	55 to 59	0·93 (0·5–1)	0·59 (0·4–0·9)	0·38 (0–1)	0·57 (0·4–1)
Global	60 to 64	0·97 (0·5–1)	0·62 (0·5–0·9)	0·6 (0–1)	0·6 (0·4–1)
Global	65 to 69	1 (0·5–1)	0·63 (0·5–1)	0·71 (0–1)	0·61 (0·5–1)
Global	70 to 74	1·02 (1–1·5)	0·64 (0·5–1)	0·81 (0–1)	0·62 (0·5–1)
Global	75 to 79	1·04 (1–2)	0·65 (0·5–1)	0·87 (0–1)	0·64 (0·5–1)
Global	80 plus	1·08 (1–2)	0·7 (0·5–1·5)	0·98 (0·5–2)	0·69 (0·5–1·7)
Central Asia	15 to 19	0·04 (0–1)	0·11 (0–0·5)	0 (0–0)	0·02 (0–0·2)
Central Asia	20 to 24	0·06 (0–1)	0·22 (0–0·5)	0 (0–0)	0·04 (0–0·3)
Central Asia	25 to 29	0·11 (0–1)	0·29 (0–0·6)	0 (0–0)	0·09 (0–0·4)
Central Asia	30 to 34	0·19 (0–1)	0·34 (0–0·7)	0·02 (0–0)	0·22 (0–0·5)
Central Asia	35 to 39	0·32 (0–1)	0·4 (0–0·9)	0·07 (0–1)	0·35 (0–1)
Central Asia	40 to 44	0·53 (0–1)	0·47 (0·3–1)	0·19 (0–1)	0·47 (0·2–1)
Central Asia	45 to 49	0·8 (0–1)	0·54 (0·4–1)	0·41 (0–1)	0·57 (0·3–1)
Central Asia	50 to 54	0·94 (0·5–1)	0·59 (0·4–1)	0·66 (0–1)	0·67 (0·4–1)
Central Asia	55 to 59	0·98 (0·5–1)	0·67 (0·5–1)	0·84 (0–1)	0·75 (0·4–1·7)
Central Asia	60 to 64	1·02 (1–1·01)	0·75 (0·5–1)	0·95 (0–1)	0·82 (0·5–2)
Central Asia	65 to 69	1·05 (1–2)	0·85 (0·5–2)	1·01 (0·5–2)	0·94 (0·5–2)
Central Asia	70 to 74	1·09 (1–2)	0·92 (0·5–2)	1·07 (0·5–2)	1·02 (0·5–2·2)
Central Asia	75 to 79	1·16 (1–2)	1·23 (0·5–2·6)	1·21 (1–3)	1·38 (0·5–2·9)
Central Asia	80 plus	1·21 (1–3)	1·37 (0·5–3)	1·27 (1–3)	1·43 (0·5–3)

Central Europe	15 to 19	0 (0–0)	0·01 (0–0·1)	0 (0–0)	0 (0–0)
Central Europe	20 to 24	0 (0–0)	0·04 (0–0·3)	0 (0–0)	0·01 (0–0)
Central Europe	25 to 29	0 (0–0)	0·12 (0–0·4)	0 (0–0)	0·02 (0–0·3)
Central Europe	30 to 34	0·02 (0–0·5)	0·19 (0–0·4)	0·01 (0–0)	0·11 (0–0·4)
Central Europe	35 to 39	0·1 (0–1)	0·27 (0–0·5)	0·02 (0–0)	0·26 (0–0·7)
Central Europe	40 to 44	0·23 (0–1)	0·36 (0–0·7)	0·07 (0–1)	0·38 (0–0·9)
Central Europe	45 to 49	0·45 (0–1)	0·43 (0·2–0·8)	0·15 (0–1)	0·46 (0·2–1)
Central Europe	50 to 54	0·64 (0–1)	0·5 (0·3–0·9)	0·25 (0–1)	0·51 (0·3–1)
Central Europe	55 to 59	0·85 (0–1)	0·57 (0·4–0·9)	0·42 (0–1)	0·58 (0·4–1)
Central Europe	60 to 64	0·95 (0·5–1)	0·62 (0·5–1)	0·68 (0–1)	0·63 (0·4–1)
Central Europe	65 to 69	0·99 (0·5–1)	0·66 (0·5–1)	0·84 (0–1)	0·66 (0·5–1)
Central Europe	70 to 74	1·05 (1–2)	0·68 (0·5–1)	0·93 (0·5–1)	0·69 (0·5–1·21)
Central Europe	75 to 79	1·1 (1–2)	0·7 (0·5–1·5)	1 (0·5–2)	0·73 (0·5–1·9)
Central Europe	80 plus	1·19 (1–2)	0·87 (0·5–2·4)	1·17 (0·5–3)	0·92 (0·5–2·5)
Eastern Europe	15 to 19	0 (0–0)	0·01 (0–0·2)	0·01 (0–0)	0·01 (0–0)
Eastern Europe	20 to 24	0 (0–0)	0·03 (0–0·3)	0·01 (0–0)	0·01 (0–0·1)
Eastern Europe	25 to 29	0·01 (0–0)	0·08 (0–0·4)	0·01 (0–0)	0·03 (0–0·3)
Eastern Europe	30 to 34	0·04 (0–1)	0·16 (0–0·5)	0·01 (0–0)	0·11 (0–0·4)
Eastern Europe	35 to 39	0·09 (0–1)	0·25 (0–0·6)	0·04 (0–1)	0·24 (0–0·7)
Eastern Europe	40 to 44	0·18 (0–1)	0·34 (0–0·7)	0·09 (0–1)	0·36 (0–1)
Eastern Europe	45 to 49	0·39 (0–1)	0·42 (0·2–0·8)	0·19 (0–1)	0·45 (0·2–1)
Eastern Europe	50 to 54	0·68 (0–1)	0·48 (0·3–1)	0·44 (0–1)	0·53 (0·3–1)
Eastern Europe	55 to 59	0·93 (0·5–1)	0·57 (0·4–1)	0·73 (0–1)	0·62 (0·4–1)
Eastern Europe	60 to 64	1 (0·5–1)	0·67 (0·5–1)	0·93 (0–1)	0·78 (0·4–2)
Eastern Europe	65 to 69	1·08 (1–2)	0·82 (0·5–2)	1·05 (0·5–2)	0·96 (0·5–2·3)
Eastern Europe	70 to 74	1·22 (1–3)	1·08 (0·5–2·5)	1·23 (0·5–3)	1·21 (0·5–2·7)
Eastern Europe	75 to 79	1·33 (1–3)	1·37 (0·5–3)	1·4 (1–3)	1·52 (0·5–3)
Eastern Europe	80 plus	1·42 (1–3)	1·69 (0·6–3·3)	1·63 (1–3)	1·87 (0·5–3·3)
Australasia	15 to 19	0 (0–0)	0 (0–0)	0 (0–0)	0·01 (0–0)
Australasia	20 to 24	0 (0–0)	0 (0–0)	0·01 (0–0)	0·01 (0–0)
Australasia	25 to 29	0 (0–0)	0·01 (0–0)	0·01 (0–0)	0·02 (0–0·2)

Australasia	30 to 34	0·01<br>(0–0)	0·02<br>(0–0·3)	0·01<br>(0–0)	0·05<br>(0–0·4)
Australasia	35 to 39	0·01<br>(0–0)	0·05<br>(0–0·4)	0·02<br>(0–0)	0·15<br>(0–0·6)
Australasia	40 to 44	0·04<br>(0–0·5)	0·11<br>(0–0·4)	0·03<br>(0–0·51)	0·29<br>(0–0·8)
Australasia	45 to 49	0·09<br>(0–1)	0·2<br>(0–0·5)	0·07<br>(0–1)	0·41<br>(0–0·9)
Australasia	50 to 54	0·18<br>(0–1)	0·28<br>(0–0·7)	0·17<br>(0–1)	0·5<br>(0·2–0·9)
Australasia	55 to 59	0·37<br>(0–1)	0·38<br>(0–0·8)	0·25<br>(0–1)	0·55<br>(0·3–1)
Australasia	60 to 64	0·6<br>(0–1)	0·47<br>(0·2–0·8)	0·39<br>(0–1)	0·61<br>(0·4–1)
Australasia	65 to 69	0·77<br>(0–1)	0·53<br>(0·3–0·8)	0·56<br>(0–1)	0·67<br>(0·4–1)
Australasia	70 to 74	0·9<br>(0·5–1)	0·58<br>(0·4–0·8)	0·76<br>(0–1)	0·7<br>(0·5–1)
Australasia	75 to 79	0·96<br>(0·5–1)	0·61<br>(0·5–0·9)	0·9<br>(0–1)	0·74<br>(0·5–1·8)
Australasia	80 plus	1·05<br>(0·5–2)	0·67<br>(0·5–1·5)	1·06<br>(0·5–2)	0·9<br>(0·5–2·3)
High-income Asia Pacific	15 to 19	0·02<br>(0–0)	0·04<br>(0–0·4)	0·01<br>(0–0)	0·02<br>(0–0·2)
High-income Asia Pacific	20 to 24	0·06<br>(0–1)	0·09<br>(0–1)	0·05<br>(0–1)	0·07<br>(0–1)
High-income Asia Pacific	25 to 29	0·09<br>(0–1)	0·12<br>(0–0·8)	0·06<br>(0–1)	0·11<br>(0–1)
High-income Asia Pacific	30 to 34	0·09<br>(0–1)	0·15<br>(0–0·7)	0·06<br>(0–1)	0·2<br>(0–1)
High-income Asia Pacific	35 to 39	0·1<br>(0–1)	0·19<br>(0–0·6)	0·06<br>(0–1)	0·29<br>(0–1)
High-income Asia Pacific	40 to 44	0·12<br>(0–1)	0·26<br>(0–0·6)	0·07<br>(0–1)	0·38<br>(0–0·9)
High-income Asia Pacific	45 to 49	0·18<br>(0–1)	0·32<br>(0–0·6)	0·1<br>(0–1)	0·44<br>(0·1–0·9)
High-income Asia Pacific	50 to 54	0·29<br>(0–1)	0·38<br>(0–0·7)	0·11<br>(0–1)	0·46<br>(0·2–0·8)
High-income Asia Pacific	55 to 59	0·48<br>(0–1)	0·44<br>(0·2–0·7)	0·12<br>(0–1)	0·48<br>(0·3–0·7)
High-income Asia Pacific	60 to 64	0·72<br>(0–1)	0·5<br>(0·3–0·7)	0·15<br>(0–1)	0·5<br>(0·3–0·7)
High-income Asia Pacific	65 to 69	0·82<br>(0·5–1)	0·53<br>(0·4–0·7)	0·2<br>(0–1)	0·51<br>(0·4–0·6)
High-income Asia Pacific	70 to 74	0·89<br>(0·5–1)	0·55<br>(0·4–0·7)	0·34<br>(0–1)	0·52<br>(0·4–0·6)
High-income Asia Pacific	75 to 79	0·93<br>(0·5–1)	0·56<br>(0·4–0·7)	0·49<br>(0–1)	0·53<br>(0·4–0·7)
High-income Asia Pacific	80 plus	1·01<br>(0·5–2)	0·57<br>(0·5–1)	0·73<br>(0–1)	0·55<br>(0·5–0·9)
High-income North America	15 to 19	0·01<br>(0–0)	0·02<br>(0–0·3)	0·01<br>(0–0)	0·01<br>(0–0)
High-income North America	20 to 24	0·02<br>(0–0)	0·04<br>(0–0·4)	0·01<br>(0–0)	0·02<br>(0–0·1)
High-income North America	25 to 29	0·05<br>(0–0·5)	0·1<br>(0–0·5)	0·01<br>(0–0)	0·03<br>(0–0·4)
High-income North America	30 to 34	0·11<br>(0–1)	0·21<br>(0–0·6)	0·02<br>(0–0)	0·12<br>(0–0·7)
High-income North America	35 to 39	0·24<br>(0–1)	0·32<br>(0–0·7)	0·04<br>(0–1)	0·3<br>(0–0·8)
High-income North America	40 to 44	0·44<br>(0–1)	0·42<br>(0–0·8)	0·14<br>(0–1)	0·47<br>(0·1–1)

High-income North America	45 to 49	0·62 (0–1)	0·5 (0·2–0·9)	0·28 (0–1)	0·59 (0·3–1)
High-income North America	50 to 54	0·77 (0–1)	0·57 (0·3–1)	0·46 (0–1)	0·68 (0·4–1)
High-income North America	55 to 59	0·88 (0·5–1)	0·64 (0·4–1)	0·6 (0–1)	0·74 (0·4–1)
High-income North America	60 to 64	0·94 (0·5–1)	0·69 (0·5–1)	0·76 (0–1)	0·8 (0·5–1)
High-income North America	65 to 69	0·96 (0·5–1)	0·71 (0·5–1)	0·86 (0–1)	0·85 (0·5–1)
High-income North America	70 to 74	0·99 (0·5–1)	0·71 (0·5–1)	0·94 (0–1)	0·86 (0·6–1·9)
High-income North America	75 to 79	1·01 (1–1)	0·7 (0·5–1)	1 (0·5–2)	0·9 (0·6–2)
High-income North America	80 plus	1·08 (1–2)	0·78 (0·5–2·1)	1·15 (0·5–3)	1·13 (0·6–2·4)
Southern Latin America	15 to 19	0 (0–0)	0·01 (0–0·2)	0 (0–0)	0·01 (0–0)
Southern Latin America	20 to 24	0 (0–0)	0·02 (0–0·2)	0·01 (0–0)	0·01 (0–0)
Southern Latin America	25 to 29	0 (0–0)	0·04 (0–0·3)	0 (0–0)	0·01 (0–0·1)
Southern Latin America	30 to 34	0 (0–0)	0·1 (0–0·4)	0 (0–0)	0·03 (0–0·3)
Southern Latin America	35 to 39	0·02 (0–0·5)	0·17 (0–0·4)	0 (0–0)	0·13 (0–0·4)
Southern Latin America	40 to 44	0·1 (0–1)	0·28 (0–0·5)	0·02 (0–0)	0·28 (0–0·6)
Southern Latin America	45 to 49	0·26 (0–1)	0·38 (0–0·7)	0·08 (0–1)	0·41 (0–0·9)
Southern Latin America	50 to 54	0·53 (0–1)	0·47 (0·2–0·8)	0·18 (0–1)	0·51 (0·2–1)
Southern Latin America	55 to 59	0·74 (0–1)	0·55 (0·3–0·9)	0·3 (0–1)	0·57 (0·3–1)
Southern Latin America	60 to 64	0·88 (0·5–1)	0·62 (0·4–0·9)	0·46 (0–1)	0·62 (0·4–1)
Southern Latin America	65 to 69	0·93 (0·5–1)	0·64 (0·5–0·9)	0·6 (0–1)	0·66 (0·5–1)
Southern Latin America	70 to 74	0·96 (0·5–1)	0·65 (0·5–0·9)	0·76 (0–1)	0·66 (0·5–1)
Southern Latin America	75 to 79	0·98 (0·5–1)	0·64 (0·5–1)	0·83 (0–1)	0·67 (0·5–1)
Southern Latin America	80 plus	1·02 (0·5–1·51)	0·66 (0·5–1)	0·92 (0·5–1·5)	0·68 (0·5–1)
Western Europe	15 to 19	0 (0–0)	0 (0–0)	0 (0–0)	0 (0–0)
Western Europe	20 to 24	0·02 (0–0·5)	0·03 (0–0·3)	0 (0–0)	0·01 (0–0)
Western Europe	25 to 29	0·09 (0–0·5)	0·11 (0–0·5)	0·01 (0–0)	0·04 (0–0·4)
Western Europe	30 to 34	0·12 (0–1)	0·16 (0–0·6)	0·01 (0–0)	0·12 (0–0·6)
Western Europe	35 to 39	0·11 (0–1)	0·17 (0–0·6)	0·02 (0–0)	0·25 (0–0·7)
Western Europe	40 to 44	0·12 (0–1)	0·21 (0–0·6)	0·04 (0–1)	0·36 (0–0·8)
Western Europe	45 to 49	0·16 (0–1)	0·26 (0–0·7)	0·09 (0–1)	0·44 (0–0·9)
Western Europe	50 to 54	0·27 (0–1)	0·34 (0–0·8)	0·16 (0–1)	0·5 (0·2–1)
Western Europe	55 to 59	0·47 (0–1)	0·43 (0–0·8)	0·24 (0–1)	0·55 (0·3–1)

Western Europe	60 to 64	0·71 (0–1)	0·52 (0·3–0·8)	0·4 (0–1)	0·61 (0·4–1)
Western Europe	65 to 69	0·84 (0·5–1)	0·58 (0·4–0·9)	0·58 (0–1)	0·66 (0·4–1)
Western Europe	70 to 74	0·93 (0·5–1)	0·6 (0·4–0·9)	0·77 (0–1)	0·68 (0·5–1)
Western Europe	75 to 79	0·96 (0·5–1)	0·62 (0·5–0·9)	0·89 (0–1)	0·7 (0·5–1)
Western Europe	80 plus	1·02 (0·5–2)	0·65 (0·5–1)	0·99 (0·5–2)	0·74 (0·5–1·9)
Andean Latin America	15 to 19	0 (0–0)	0·06 (0–0·3)	0 (0–0)	0·01 (0–0·2)
Andean Latin America	20 to 24	0 (0–0)	0·09 (0–0·3)	0 (0–0)	0·01 (0–0·2)
Andean Latin America	25 to 29	0 (0–0)	0·17 (0–0·4)	0 (0–0)	0·02 (0–0·2)
Andean Latin America	30 to 34	0·02 (0–0·01)	0·23 (0–0·4)	0 (0–0)	0·07 (0–0·3)
Andean Latin America	35 to 39	0·07 (0–1)	0·3 (0–0·5)	0 (0–0)	0·15 (0–0·4)
Andean Latin America	40 to 44	0·26 (0–1)	0·38 (0·1–0·7)	0·01 (0–0)	0·26 (0–0·6)
Andean Latin America	45 to 49	0·58 (0–1)	0·49 (0·3–0·8)	0·05 (0–1)	0·38 (0–0·8)
Andean Latin America	50 to 54	0·79 (0–1)	0·58 (0·3–0·9)	0·13 (0–1)	0·49 (0·2–1)
Andean Latin America	55 to 59	0·87 (0·5–1)	0·64 (0·4–0·9)	0·24 (0–1)	0·58 (0·3–1)
Andean Latin America	60 to 64	0·93 (0·5–1)	0·69 (0·5–1)	0·38 (0–1)	0·63 (0·4–1)
Andean Latin America	65 to 69	0·96 (0·5–1)	0·71 (0·5–1)	0·51 (0–1)	0·67 (0·4–1)
Andean Latin America	70 to 74	0·98 (0·5–1)	0·72 (0·5–1)	0·64 (0–1)	0·69 (0·5–1)
Andean Latin America	75 to 79	0·99 (0·5–1)	0·72 (0·5–1)	0·75 (0–1)	0·7 (0·5–1)
Andean Latin America	80 plus	1·02 (1–1·5)	0·74 (0·5–1)	0·9 (0–1)	0·72 (0·5–1)
Caribbean	15 to 19	0·01 (0–0)	0·08 (0–0·4)	0 (0–0)	0·01 (0–0)
Caribbean	20 to 24	0·03 (0–0·5)	0·18 (0–0·5)	0·01 (0–0)	0·02 (0–0·2)
Caribbean	25 to 29	0·08 (0–1)	0·28 (0–0·6)	0·01 (0–0)	0·03 (0–0·3)
Caribbean	30 to 34	0·25 (0–1)	0·39 (0·1–0·7)	0·01 (0–0)	0·11 (0–0·4)
Caribbean	35 to 39	0·56 (0–1)	0·48 (0·3–0·8)	0·01 (0–0)	0·26 (0–0·6)
Caribbean	40 to 44	0·8 (0–1)	0·57 (0·4–0·8)	0·06 (0–1)	0·42 (0·1–0·8)
Caribbean	45 to 49	0·92 (0·5–1)	0·64 (0·4–0·9)	0·21 (0–1)	0·54 (0·3–1)
Caribbean	50 to 54	0·97 (0·5–1)	0·7 (0·5–1)	0·43 (0–1)	0·64 (0·4–1)
Caribbean	55 to 59	0·99 (0·5–1)	0·75 (0·5–1)	0·61 (0–1)	0·7 (0·5–1)
Caribbean	60 to 64	1·02 (0·5–2)	0·79 (0·6–1)	0·79 (0–1)	0·73 (0·5–1)
Caribbean	65 to 69	1·04 (1–2)	0·8 (0·6–1)	0·88 (0–1)	0·77 (0·5–1)
Caribbean	70 to 74	1·06 (1–2)	0·77 (0·6–1)	0·95 (0–1)	0·77 (0·6–1)

Caribbean	75 to 79	1·07 (1–2)	0·76 (0·6–1)	0·97 (0·5–1·5)	0·78 (0·6–1·5)
Caribbean	80 plus	1·08 (1–2)	0·78 (0·6–1·51)	1·03 (0·5–2)	0·81 (0·6–2)
Central Latin America	15 to 19	0 (0–0)	0·02 (0–0·3)	0·02 (0–0)	0·02 (0–0·2)
Central Latin America	20 to 24	0·02 (0–0·5)	0·07 (0–0·4)	0·02 (0–0)	0·03 (0–0·4)
Central Latin America	25 to 29	0·09 (0–1)	0·18 (0–0·6)	0·02 (0–0)	0·04 (0–0·5)
Central Latin America	30 to 34	0·27 (0–1)	0·33 (0–0·7)	0·02 (0–0)	0·06 (0–0·7)
Central Latin America	35 to 39	0·55 (0–1)	0·47 (0·1–0·8)	0·03 (0–1)	0·16 (0–0·8)
Central Latin America	40 to 44	0·8 (0–1)	0·61 (0·3–0·9)	0·07 (0–1)	0·34 (0–1)
Central Latin America	45 to 49	0·9 (0·5–1)	0·7 (0·4–0·9)	0·18 (0–1)	0·53 (0–1)
Central Latin America	50 to 54	0·96 (0·5–1)	0·77 (0·5–1)	0·36 (0–1)	0·67 (0·3–1)
Central Latin America	55 to 59	0·99 (0·5–2)	0·81 (0·6–1)	0·56 (0–1)	0·77 (0·4–1)
Central Latin America	60 to 64	1·02 (0·5–2)	0·84 (0·6–1)	0·73 (0–1)	0·82 (0·5–1)
Central Latin America	65 to 69	1·04 (0·5–2)	0·86 (0·7–1)	0·87 (0–1)	0·86 (0·6–1)
Central Latin America	70 to 74	1·06 (1–2)	0·86 (0·6–1)	0·95 (0–2)	0·87 (0·6–1)
Central Latin America	75 to 79	1·07 (1–2)	0·86 (0·6–1)	0·99 (0·5–2)	0·89 (0·6–1·8)
Central Latin America	80 plus	1·09 (1–2)	0·95 (0·6–2)	1·07 (0·5–2)	0·98 (0·6–2)
Tropical Latin America	15 to 19	0·01 (0–0)	0·02 (0–0·2)	0·03 (0–1)	0·04 (0–1)
Tropical Latin America	20 to 24	0·01 (0–0)	0·04 (0–0·3)	0·03 (0–1)	0·03 (0–1)
Tropical Latin America	25 to 29	0·02 (0–0·5)	0·1 (0–0·4)	0·02 (0–0)	0·03 (0–0·4)
Tropical Latin America	30 to 34	0·05 (0–0·5)	0·23 (0–0·5)	0·02 (0–0)	0·04 (0–0·4)
Tropical Latin America	35 to 39	0·22 (0–1)	0·35 (0–0·6)	0·02 (0–0)	0·1 (0–0·4)
Tropical Latin America	40 to 44	0·55 (0–1)	0·47 (0·3–0·8)	0·02 (0–0)	0·25 (0–0·6)
Tropical Latin America	45 to 49	0·81 (0–1)	0·57 (0·4–0·9)	0·05 (0–1)	0·38 (0–0·7)
Tropical Latin America	50 to 54	0·91 (0·5–1)	0·64 (0·4–0·9)	0·13 (0–1)	0·48 (0·3–0·9)
Tropical Latin America	55 to 59	0·96 (0·5–1)	0·71 (0·5–1)	0·28 (0–1)	0·56 (0·4–1)
Tropical Latin America	60 to 64	1 (0·5–1)	0·74 (0·5–1)	0·54 (0–1)	0·63 (0·4–1)
Tropical Latin America	65 to 69	1·01 (0·5–1·51)	0·76 (0·5–1)	0·73 (0–1)	0·67 (0·5–1)
Tropical Latin America	70 to 74	1·05 (1–2)	0·73 (0·6–1)	0·86 (0–1)	0·68 (0·5–1)
Tropical Latin America	75 to 79	1·06 (1–2)	0·71 (0·6–1)	0·9 (0–1)	0·68 (0·5–1)
Tropical Latin America	80 plus	1·07 (1–2)	0·69 (0·5–1)	0·94 (0·5–1·51)	0·68 (0·5–1)
North Africa and Middle East	15 to 19	0·03 (0–0·5)	0·27 (0–0·4)	0 (0–0)	0·07 (0–0·4)

		0·06	0·3	0	0·07
North Africa and Middle East	20 to 24	(0–1)	(0–0·5)	(0–0)	(0–0·4)
		0·15	0·35	0	0·17
North Africa and Middle East	25 to 29	(0–1)	(0·1–0·5)	(0–0)	(0–0·4)
		0·44	0·42	0·02	0·27
North Africa and Middle East	30 to 34	(0–1)	(0·2–0·6)	(0–0)	(0–0·5)
		0·66	0·47	0·06	0·37
North Africa and Middle East	35 to 39	(0–1)	(0·3–0·7)	(0–1)	(0–0·7)
		0·87	0·54	0·27	0·51
North Africa and Middle East	40 to 44	(0–1)	(0·4–0·8)	(0–1)	(0·3–1)
		0·96	0·64	0·62	0·7
North Africa and Middle East	45 to 49	(0·5–1)	(0·4–1)	(0–1)	(0·4–1·8)
		1·01	0·72	0·86	0·85
North Africa and Middle East	50 to 54	(1–1)	(0·5–1)	(0–1)	(0·5–2)
		1·02	0·8	0·95	0·94
North Africa and Middle East	55 to 59	(1–1·5)	(0·5–1·5)	(0–1·5)	(0·5–2)
		1·05	0·86	0·99	0·95
North Africa and Middle East	60 to 64	(1–2)	(0·6–2)	(0·5–1·5)	(0·5–2)
		1·05	0·89	1·01	0·96
North Africa and Middle East	65 to 69	(1–2)	(0·6–2)	(0·5–2)	(0·5–2)
		1·06	0·87	1·01	0·89
North Africa and Middle East	70 to 74	(1–2)	(0·6–2)	(0·5–1·5)	(0·5–2)
		1·07	0·88	1·03	0·88
North Africa and Middle East	75 to 79	(1–2)	(0·5–2)	(0·5–2)	(0·5–2)
		1·09	0·9	1·05	0·91
North Africa and Middle East	80 plus	(1–2)	(0·5–2)	(0·5–2)	(0·5–2)
		0·03	0·1	0	0·01
South Asia	15 to 19	(0–0·5)	(0–1)	(0–0)	(0–0·1)
		0·03	0·15	0	0·04
South Asia	20 to 24	(0–1)	(0–1)	(0–0)	(0–0·3)
		0·04	0·24	0	0·09
South Asia	25 to 29	(0–1)	(0–1)	(0–0)	(0–0·4)
		0·08	0·36	0·01	0·28
South Asia	30 to 34	(0–1)	(0–1)	(0–0)	(0–0·7)
		0·15	0·42	0·03	0·37
South Asia	35 to 39	(0–1)	(0·1–1)	(0–1)	(0–1)
		0·48	0·53	0·09	0·5
South Asia	40 to 44	(0–1)	(0·3–2)	(0–1)	(0·2–1·9)
		0·79	0·57	0·19	0·58
South Asia	45 to 49	(0–1)	(0·4–1)	(0–1)	(0·3–2)
		0·89	0·62	0·35	0·65
South Asia	50 to 54	(0–1)	(0·4–1)	(0–1)	(0·4–2)
		0·96	0·68	0·48	0·7
South Asia	55 to 59	(0·5–1)	(0·4–1·9)	(0–1)	(0·4–2)
		0·99	0·68	0·66	0·71
South Asia	60 to 64	(0·5–1)	(0·5–1)	(0–1)	(0·4–2)
		1	0·68	0·76	0·72
South Asia	65 to 69	(0·5–1)	(0·5–1·01)	(0–1)	(0·5–2)
		1·01	0·68	0·85	0·74
South Asia	70 to 74	(1–1)	(0·5–1·5)	(0–1)	(0·5–2)
		1·02	0·72	0·9	0·76
South Asia	75 to 79	(1–1·5)	(0·5–1·8)	(0–1)	(0·5–2)
		1·03	0·73	0·96	0·81
South Asia	80 plus	(1–2)	(0·5–1·5)	(0·5–1)	(0·5–2·1)
		0	0·11	0	0·04
East Asia	15 to 19	(0–0)	(0–0·4)	(0–0)	(0–0·3)
		0·03	0·21	0	0·09
East Asia	20 to 24	(0–0·5)	(0–0·5)	(0–0)	(0–0·4)
		0·1	0·3	0	0·17
East Asia	25 to 29	(0–1)	(0–0·5)	(0–0)	(0–0·4)
		0·14	0·34	0	0·28
East Asia	30 to 34	(0–1)	(0–0·5)	(0–0)	(0–0·5)

East Asia	35 to 39	0·22 (0–1)	0·38 (0·2–0·6)	0·01 (0–0)	0·35 (0·1–0·5)
East Asia	40 to 44	0·37 (0–1)	0·42 (0·3–0·6)	0·03 (0–0·51)	0·4 (0·2–0·5)
East Asia	45 to 49	0·64 (0–1)	0·47 (0·4–0·6)	0·04 (0–1)	0·43 (0·3–0·6)
East Asia	50 to 54	0·81 (0–1)	0·5 (0·4–0·6)	0·08 (0–1)	0·46 (0·4–0·6)
East Asia	55 to 59	0·9 (0·5–1)	0·53 (0·4–0·7)	0·16 (0–1)	0·48 (0·4–0·6)
East Asia	60 to 64	0·97 (0·5–1·5)	0·56 (0·5–0·7)	0·33 (0–1)	0·5 (0·4–0·6)
East Asia	65 to 69	1·02 (0·5–2)	0·57 (0·5–0·8)	0·48 (0–1)	0·51 (0·4–0·6)
East Asia	70 to 74	1·04 (0·5–2)	0·58 (0·5–0·8)	0·64 (0–1)	0·52 (0·5–0·6)
East Asia	75 to 79	1·07 (0·5–2)	0·59 (0·5–1)	0·75 (0–1)	0·54 (0·5–0·6)
East Asia	80 plus	1·12 (1–2)	0·66 (0·5–1·5)	0·92 (0–2)	0·61 (0·5–1·5)
Oceania	15 to 19	0·01 (0–0)	0·22 (0–2·2)	0 (0–0)	0·02 (0–0·2)
Oceania	20 to 24	0·02 (0–0·5)	0·3 (0–2·2)	0 (0–0)	0·07 (0–0·4)
Oceania	25 to 29	0·12 (0–1)	0·4 (0·1–0·7)	0 (0–0)	0·25 (0–0·6)
Oceania	30 to 34	0·46 (0–1)	0·52 (0·3–2)	0·02 (0–0)	0·48 (0·2–2·1)
Oceania	35 to 39	0·75 (0–1)	0·6 (0·4–1)	0·15 (0–1)	0·58 (0·3–1·9)
Oceania	40 to 44	0·92 (0·5–1)	0·68 (0·4–1)	0·45 (0–1)	0·75 (0·4–2)
Oceania	45 to 49	0·97 (0·5–1)	0·7 (0·5–1)	0·65 (0–1)	0·82 (0·5–2)
Oceania	50 to 54	1·03 (0·5–2)	0·74 (0·5–1)	0·81 (0–1)	0·87 (0·5–2)
Oceania	55 to 59	1·05 (0·5–2)	0·76 (0·5–1)	0·91 (0–2)	0·9 (0·6–2)
Oceania	60 to 64	1·09 (0·5–2)	0·77 (0·6–1)	0·93 (0–2)	0·88 (0·6–2·1)
Oceania	65 to 69	1·13 (1–2)	0·77 (0·6–1)	0·93 (0–2)	0·87 (0·6–2·1)
Oceania	70 to 74	1·12 (1–2)	0·75 (0·6–1·5)	0·94 (0–2)	0·82 (0·5–2)
Oceania	75 to 79	1·11 (1–2)	0·71 (0·5–1·5)	0·96 (0–2)	0·81 (0·5–2)
Oceania	80 plus	1·13 (1–2)	0·7 (0·5–1·5)	0·98 (0–2)	0·8 (0·5–2·1)
Southeast Asia	15 to 19	0·01 (0–0)	0·22 (0–0·5)	0 (0–0)	0·02 (0–0·2)
Southeast Asia	20 to 24	0·02 (0–0·01)	0·37 (0–2·2)	0 (0–0)	0·06 (0–0·3)
Southeast Asia	25 to 29	0·06 (0–1)	0·42 (0·1–2)	0 (0–0)	0·17 (0–0·4)
Southeast Asia	30 to 34	0·17 (0–1)	0·41 (0·2–0·8)	0·01 (0–0)	0·3 (0–0·5)
Southeast Asia	35 to 39	0·31 (0–1)	0·45 (0·3–0·8)	0·03 (0–1)	0·38 (0·1–0·7)
Southeast Asia	40 to 44	0·57 (0–1)	0·48 (0·3–0·7)	0·09 (0–1)	0·44 (0·2–1)
Southeast Asia	45 to 49	0·73 (0–1)	0·51 (0·4–0·8)	0·14 (0–1)	0·48 (0·3–1)

Southeast Asia	50 to 54	0·85 (0–1)	0·54 (0·4–0·8)	0·22 (0–1)	0·52 (0·4–1)
Southeast Asia	55 to 59	0·92 (0·5–1)	0·57 (0·4–0·8)	0·3 (0–1)	0·54 (0·4–1)
Southeast Asia	60 to 64	0·96 (0·5–1)	0·59 (0·5–0·8)	0·46 (0–1)	0·56 (0·4–1)
Southeast Asia	65 to 69	0·99 (0·5–1)	0·61 (0·5–0·9)	0·53 (0–1)	0·57 (0·5–1)
Southeast Asia	70 to 74	1·01 (0·5–2)	0·61 (0·5–1)	0·67 (0–1)	0·59 (0·5–1)
Southeast Asia	75 to 79	1·01 (0·5–2)	0·6 (0·5–1)	0·71 (0–1)	0·59 (0·5–1)
Southeast Asia	80 plus	1·05 (0·5–2)	0·62 (0·5–1)	0·81 (0–1)	0·62 (0·5–1·5)
Central Sub-Saharan Africa	15 to 19	0 (0–0)	0·21 (0–2·6)	0 (0–0)	0·01 (0–0·1)
Central Sub-Saharan Africa	20 to 24	0·01 (0–0)	0·35 (0–3)	0 (0–0)	0·02 (0–0·1)
Central Sub-Saharan Africa	25 to 29	0·02 (0–0)	0·46 (0–4·02)	0 (0–0)	0·06 (0–0·3)
Central Sub-Saharan Africa	30 to 34	0·02 (0–0·5)	0·48 (0–3)	0·01 (0–0)	0·13 (0–2)
Central Sub-Saharan Africa	35 to 39	0·03 (0–0·51)	0·52 (0–2·6)	0·01 (0–0)	0·21 (0–2)
Central Sub-Saharan Africa	40 to 44	0·08 (0–1)	0·5 (0·1–2·3)	0·02 (0–0)	0·35 (0–2)
Central Sub-Saharan Africa	45 to 49	0·16 (0–1)	0·53 (0·2–2·2)	0·03 (0–1)	0·46 (0–2)
Central Sub-Saharan Africa	50 to 54	0·27 (0–1)	0·57 (0·3–2·2)	0·06 (0–1)	0·56 (0·2–2)
Central Sub-Saharan Africa	55 to 59	0·51 (0–1)	0·61 (0·3–2·1)	0·1 (0–1)	0·6 (0·3–2)
Central Sub-Saharan Africa	60 to 64	0·74 (0–1)	0·62 (0·4–2)	0·2 (0–1)	0·62 (0·4–2)
Central Sub-Saharan Africa	65 to 69	0·84 (0–1)	0·62 (0·4–2)	0·26 (0–1)	0·62 (0·4–2)
Central Sub-Saharan Africa	70 to 74	0·91 (0·5–1)	0·63 (0·4–1·5)	0·4 (0–1)	0·65 (0·4–2)
Central Sub-Saharan Africa	75 to 79	0·93 (0·5–1)	0·63 (0·4–1·5)	0·47 (0–1)	0·65 (0·4–2)
Central Sub-Saharan Africa	80 plus	0·97 (0·5–1·5)	0·64 (0·5–1·5)	0·66 (0–1)	0·67 (0·5–2)
Eastern Sub-Saharan Africa	15 to 19	0 (0–0)	0·15 (0–2)	0 (0–0)	0·02 (0–0·2)
Eastern Sub-Saharan Africa	20 to 24	0·01 (0–0)	0·27 (0–2·3)	0 (0–0)	0·05 (0–0·3)
Eastern Sub-Saharan Africa	25 to 29	0·01 (0–0)	0·3 (0–2·3)	0 (0–0)	0·09 (0–1)
Eastern Sub-Saharan Africa	30 to 34	0·02 (0–0·5)	0·37 (0–2·1)	0 (0–0)	0·27 (0–2·1)
Eastern Sub-Saharan Africa	35 to 39	0·05 (0–1)	0·41 (0–2)	0·01 (0–0)	0·31 (0–2)
Eastern Sub-Saharan Africa	40 to 44	0·11 (0–1)	0·4 (0·1–1)	0·02 (0–0)	0·36 (0–1)
Eastern Sub-Saharan Africa	45 to 49	0·18 (0–1)	0·44 (0·2–1)	0·02 (0–0)	0·43 (0–2)
Eastern Sub-Saharan Africa	50 to 54	0·18 (0–1)	0·46 (0·2–1·51)	0·02 (0–0)	0·46 (0·2–2)
Eastern Sub-Saharan Africa	55 to 59	0·38 (0–1)	0·5 (0·3–1)	0·03 (0–1)	0·49 (0·3–1·51)
Eastern Sub-Saharan Africa	60 to 64	0·61 (0–1)	0·52 (0·3–1)	0·1 (0–1)	0·51 (0·4–1)

Eastern Sub-Saharan Africa	65 to 69	0·74 (0–1)	0·52 (0·4–1)	0·1 (0–1)	0·5 (0·4–0·9)
Eastern Sub-Saharan Africa	70 to 74	0·83 (0–1)	0·54 (0·4–1)	0·15 (0–1)	0·54 (0·4–1)
Eastern Sub-Saharan Africa	75 to 79	0·88 (0·5–1)	0·55 (0·4–1)	0·17 (0–1)	0·55 (0·4–1)
Eastern Sub-Saharan Africa	80 plus	0·88 (0·5–1)	0·57 (0·4–1)	0·31 (0–1)	0·58 (0·4–1·51)
Southern Sub-Saharan Africa	15 to 19	0·01 (0–0)	0·33 (0–3)	0 (0–0)	0·02 (0–0·1)
Southern Sub-Saharan Africa	20 to 24	0·01 (0–0)	0·43 (0–5)	0 (0–0)	0·02 (0–0·1)
Southern Sub-Saharan Africa	25 to 29	0 (0–0)	0·35 (0–3)	0 (0–0)	0·02 (0–0·1)
Southern Sub-Saharan Africa	30 to 34	0·01 (0–0)	0·31 (0–2·6)	0 (0–0)	0·04 (0–0·2)
Southern Sub-Saharan Africa	35 to 39	0·02 (0–0·01)	0·32 (0–2·3)	0 (0–0)	0·11 (0–1)
Southern Sub-Saharan Africa	40 to 44	0·07 (0–1)	0·38 (0–2)	0 (0–0)	0·27 (0–2·1)
Southern Sub-Saharan Africa	45 to 49	0·35 (0–1)	0·49 (0·3–1)	0·01 (0–0)	0·42 (0–2·1)
Southern Sub-Saharan Africa	50 to 54	0·74 (0–1)	0·57 (0·4–1)	0·02 (0–0)	0·49 (0·2–2)
Southern Sub-Saharan Africa	55 to 59	0·87 (0·5–1)	0·63 (0·4–1)	0·06 (0–1)	0·56 (0·3–2)
Southern Sub-Saharan Africa	60 to 64	0·95 (0·5–1)	0·68 (0·5–1)	0·18 (0–1)	0·64 (0·4–2)
Southern Sub-Saharan Africa	65 to 69	0·98 (0·5–1·5)	0·69 (0·5–1)	0·34 (0–1)	0·64 (0·4–1·9)
Southern Sub-Saharan Africa	70 to 74	1·01 (0·5–2)	0·68 (0·5–1)	0·55 (0–1)	0·66 (0·5–1·01)
Southern Sub-Saharan Africa	75 to 79	1·02 (0·5–2)	0·67 (0·5–1)	0·68 (0–1)	0·66 (0·5–1)
Southern Sub-Saharan Africa	80 plus	1·04 (0·5–2)	0·66 (0·5–1)	0·84 (0–1)	0·67 (0·5–1·5)
Western Sub-Saharan Africa	15 to 19	0·01 (0–0)	0·17 (0–0·4)	0 (0–0)	0·02 (0–0·2)
Western Sub-Saharan Africa	20 to 24	0·01 (0–0)	0·22 (0–0·5)	0 (0–0)	0·03 (0–0·2)
Western Sub-Saharan Africa	25 to 29	0·02 (0–0)	0·24 (0–1)	0 (0–0)	0·05 (0–0·3)
Western Sub-Saharan Africa	30 to 34	0·06 (0–1)	0·34 (0–0·6)	0 (0–0)	0·15 (0–0·4)
Western Sub-Saharan Africa	35 to 39	0·07 (0–1)	0·35 (0–0·6)	0·01 (0–0)	0·22 (0–0·5)
Western Sub-Saharan Africa	40 to 44	0·17 (0–1)	0·4 (0·1–0·7)	0·01 (0–0)	0·36 (0–1)
Western Sub-Saharan Africa	45 to 49	0·36 (0–1)	0·47 (0·3–1)	0·03 (0–0·5)	0·43 (0·2–1)
Western Sub-Saharan Africa	50 to 54	0·61 (0–1)	0·5 (0·4–0·8)	0·06 (0–1)	0·48 (0·3–1)
Western Sub-Saharan Africa	55 to 59	0·68 (0–1)	0·52 (0·4–1)	0·1 (0–1)	0·51 (0·3–1)
Western Sub-Saharan Africa	60 to 64	0·91 (0·5–1)	0·59 (0·4–1)	0·24 (0–1)	0·53 (0·4–1)
Western Sub-Saharan Africa	65 to 69	0·94 (0·5–1)	0·6 (0·5–1)	0·37 (0–1)	0·57 (0·4–1)
Western Sub-Saharan Africa	70 to 74	0·95 (0·5–1)	0·61 (0·5–1)	0·46 (0–1)	0·58 (0·4–1)
Western Sub-Saharan Africa	75 to 79	0·98 (0·5–1)	0·6 (0·5–1)	0·54 (0–1)	0·61 (0·5–1·01)

Western Sub-Saharan Africa	80 plus	0·99 (0·5–1·5)	0·63 (0·5–1)	0·73 (0–1)	0·64 (0·5–1·8)

Supplementary Table 3				
Location	Age Group	Male	Female	Both
Global	15 to 39	44·6 (40·0–50·6)	14·5 (11·2–17·5)	59·1 (54·3–65·4)
Global	40 to 64	27·1 (24·2–29·8)	7·30 (5·40–8·73)	34·4 (29·8–37·8)
Global	65 plus	5·20 (3·97–6·40)	1·35 (0·759–2·12)	6·55 (4·79–8·43)
Global	15 to 95 plus	76·9 (73·0–81·3)	23·1 (18·7–27·0)	100 (100–100)
Central Asia	15 to 39	54·7 (48·4–60·4)	14·0 (10·5–18·8)	68·7 (63·4–74·1)
Central Asia	40 to 64	24·2 (20·4–27·8)	5·27 (3·86–6·69)	29·4 (24·5–33·8)
Central Asia	65 plus	1·69 (1·17–2·64)	0·169 (0·0599–0·490)	1·86 (1·24–3·14)
Central Asia	15 to 95 plus	80·6 (74·7–84·4)	19·4 (15·6–25·3)	100 (100–100)
Armenia	15 to 39	58·2 (48·6–66·3)	11·2 (7·14–17·2)	69·4 (61·6–77·0)
Armenia	40 to 64	24·2 (18·7–29·0)	3·89 (2·11–6·19)	28·1 (21·4–33·9)
Armenia	65 plus	2·42 (1·35–4·49)	0·104 (0·0118–0·498)	2·52 (1·37–4·92)
Armenia	15 to 95 plus	84·8 (77·4–89·6)	15·2 (10·4–22·6)	100 (100–100)
Azerbaijan	15 to 39	52·3 (47·0–58·4)	11·1 (8·36–14·6)	63·4 (58·4–69·5)
Azerbaijan	40 to 64	29·3 (24·8–33·0)	4·52 (3·08–6·06)	33·8 (28·2–38·1)
Azerbaijan	65 plus	2·62 (1·71–3·56)	0·166 (0·0496–0·353)	2·79 (1·78–3·92)
Azerbaijan	15 to 95 plus	84·2 (80·6–87·5)	15·8 (12·5–19·4)	100 (100–100)
Georgia	15 to 39	49·9 (40·4–58·4)	14·8 (8·19–23·1)	64·7 (57·0–72·3)
Georgia	40 to 64	27·1 (22·2–32·0)	4·16 (1·95–6·89)	31·3 (25·0–37·2)
Georgia	65 plus	3·92 (2·38–6·56)	0·113 (0·00541–0·624)	4·04 (2·39–7·24)
Georgia	15 to 95 plus	80·9 (70·0–88·6)	19·1 (11·4–30·0)	100 (100–100)
Kazakhstan	15 to 39	46·0 (38·9–52·4)	22·0 (17·1–28·6)	68·1 (61·9–74·6)
Kazakhstan	40 to 64	20·8 (16·9–24·6)	9·14 (6·68–11·5)	30·0 (24·0–35·2)
Kazakhstan	65 plus	1·62 (1·01–2·63)	0·380 (0·115–1·10)	2·00 (1·17–3·69)
Kazakhstan	15 to 95 plus	68·5 (61·5–73·8)	31·5 (26·2–38·5)	100 (100–100)
Kyrgyzstan	15 to 39	55·7 (48·2–61·8)	14·3 (9·58–21·1)	70·0 (64·1–75·7)
Kyrgyzstan	40 to 64	23·3 (19·1–27·5)	5·35 (3·46–7·37)	28·6 (23·3–33·9)
Kyrgyzstan	65 plus	1·33 (0·868–2·04)	0·0824 (0·0156–0·288)	1·41 (0·895–2·31)
Kyrgyzstan	15 to 95 plus	80·3 (72·3–85·9)	19·7 (14·1–27·7)	100 (100–100)
Mongolia	15 to 39	47·4 (41·1–52·7)	20·9 (16·1–26·6)	68·3 (62·9–73·4)

Mongolia	40 to 64	22·0 (18·5–25·4)	8·34 (6·14–10·6)	30·4 (25·5–35·1)
Mongolia	65 plus	1·19 (0·787–1·90)	0·204 (0·0683–0·542)	1·40 (0·866–2·39)
Mongolia	15 to 95 plus	70·6 (63·8–75·6)	29·4 (24·4–36·2)	100 (100–100)
Tajikistan	15 to 39	69·9 (64·8–74·5)	4·08 (2·64–5·89)	74·0 (69·2–78·2)
Tajikistan	40 to 64	24·5 (20·4–28·9)	0·956 (0·610–1·40)	25·5 (21·3–30·1)
Tajikistan	65 plus	0·534 (0·332–0·827)	0·0192 (0·00558–0·0480)	0·553 (0·341–0·865)
Tajikistan	15 to 95 plus	94·9 (93·1–96·4)	5·06 (3·56–6·94)	100 (100–100)
Turkmenistan	15 to 39	57·9 (50·3–65·0)	13·0 (8·50–18·9)	70·9 (64·8–76·7)
Turkmenistan	40 to 64	22·7 (18·9–26·6)	4·71 (2·83–6·86)	27·4 (22·3–32·6)
Turkmenistan	65 plus	1·55 (0·944–2·56)	0·0988 (0·0157–0·345)	1·65 (0·979–2·89)
Turkmenistan	15 to 95 plus	82·2 (75·1–87·4)	17·8 (12·6–24·9)	100 (100–100)
Uzbekistan	15 to 39	61·8 (55·5–67·8)	8·71 (5·74–12·9)	70·5 (64·9–75·9)
Uzbekistan	40 to 64	25·4 (20·9–29·9)	2·77 (1·74–4·01)	28·2 (23·2–33·2)
Uzbekistan	65 plus	1·26 (0·782–2·08)	0·0481 (0·0101–0·166)	1·31 (0·793–2·23)
Uzbekistan	15 to 95 plus	88·5 (84·0–91·8)	11·5 (8·20–16·0)	100 (100–100)
Central Europe	15 to 39	32·4 (28·0–36·2)	19·4 (15·3–22·9)	51·8 (46·3–56·8)
Central Europe	40 to 64	27·4 (25·5–29·7)	12·2 (10·1–15·5)	39·6 (36·5–43·2)
Central Europe	65 plus	6·86 (5·27–8·65)	1·74 (0·888–3·21)	8·59 (6·24–11·8)
Central Europe	15 to 95 plus	66·7 (61·6–71·0)	33·3 (29·0–38·4)	100 (100–100)
Albania	15 to 39	45·4 (38·1–52·7)	17·2 (11·1–22·2)	62·6 (54·3–70·1)
Albania	40 to 64	26·3 (22·4–30·2)	5·08 (3·02–8·37)	31·4 (26·1–36·8)
Albania	65 plus	5·64 (3·39–8·59)	0·347 (0·0611–1·01)	5·98 (3·47–9·47)
Albania	15 to 95 plus	77·4 (71·2–83·9)	22·6 (16·1–28·8)	100 (100–100)
Bosnia and Herzegovina	15 to 39	39·6 (34·3–44·4)	13·7 (10·3–17·2)	53·3 (47·4–58·5)
Bosnia and Herzegovina	40 to 64	31·4 (28·8–34·1)	7·61 (5·45–10·6)	39·0 (35·4–43·1)
Bosnia and Herzegovina	65 plus	6·92 (5·11–9·15)	0·750 (0·296–1·54)	7·67 (5·50–10·5)
Bosnia and Herzegovina	15 to 95 plus	77·9 (73·2–82·1)	22·1 (17·9–26·8)	100 (100–100)
Bulgaria	15 to 39	29·1 (25·4–32·2)	17·7 (14·5–21·0)	46·7 (41·7–51·5)
Bulgaria	40 to 64	29·0 (27·2–31·0)	13·8 (11·6–16·7)	42·7 (40·1–45·8)
Bulgaria	65 plus	8·31 (6·56–10·2)	2·22 (1·12–3·85)	10·5 (7·85–13·9)
Bulgaria	15 to 95 plus	66·3 (62·1–70·0)	33·7 (30·0–37·9)	100 (100–100)

Croatia	15 to 39	33·4 (27·7–38·8)	20·2 (14·8–24·8)	53·6 (46·7–60·3)
Croatia	40 to 64	25·9 (23·5–28·7)	11·6 (8·79–15·1)	37·6 (33·5–42·1)
Croatia	65 plus	7·05 (4·87–9·51)	1·77 (0·696–3·79)	8·82 (5·70–13·1)
Croatia	15 to 95 plus	66·4 (60·8–72·2)	33·6 (27·8–39·2)	100 (100–100)
Czechia	15 to 39	27·5 (23·7–30·8)	20·0 (16·5–23·4)	47·5 (42·4–52·2)
Czechia	40 to 64	27·1 (25·3–29·0)	14·7 (12·6–17·9)	41·7 (39·0–44·7)
Czechia	65 plus	8·13 (6·50–10·0)	2·60 (1·40–4·52)	10·7 (8·02–14·4)
Czechia	15 to 95 plus	62·7 (58·1–66·6)	37·3 (33·4–41·9)	100 (100–100)
Hungary	15 to 39	30·9 (26·2–35·0)	19·3 (15·0–23·0)	50·2 (44·4–55·5)
Hungary	40 to 64	28·1 (26·1–30·5)	12·4 (10·0–16·3)	40·6 (37·3–44·3)
Hungary	65 plus	7·27 (5·50–9·34)	2·00 (0·930–3·72)	9·26 (6·48–12·8)
Hungary	15 to 95 plus	66·3 (61·3–70·9)	33·7 (29·1–38·7)	100 (100–100)
Montenegro	15 to 39	37·4 (31·8–42·6)	17·9 (13·7–21·9)	55·3 (48·9–61·3)
Montenegro	40 to 64	27·8 (25·3–30·3)	9·43 (6·99–12·7)	37·3 (33·2–41·5)
Montenegro	65 plus	6·27 (4·42–8·42)	1·22 (0·456–2·49)	7·49 (4·97–10·8)
Montenegro	15 to 95 plus	71·5 (66·2–76·3)	28·5 (23·7–33·8)	100 (100–100)
North Macedonia	15 to 39	40·9 (34·4–46·7)	18·5 (13·5–23·2)	59·4 (52·6–65·6)
North Macedonia	40 to 64	27·4 (24·5–30·6)	7·17 (4·66–11·2)	34·6 (30·0–39·6)
North Macedonia	65 plus	5·48 (3·80–7·54)	0·549 (0·136–1·40)	6·03 (3·95–8·88)
North Macedonia	15 to 95 plus	73·8 (67·4–79·8)	26·2 (20·2–32·6)	100 (100–100)
Poland	15 to 39	33·0 (28·6–36·9)	21·1 (16·6–25·2)	54·1 (48·6–59·0)
Poland	40 to 64	25·9 (23·8–28·4)	12·5 (10·0–16·0)	38·4 (35·1–41·9)
Poland	65 plus	5·97 (4·58–7·60)	1·59 (0·721–3·05)	7·56 (5·43–10·6)
Poland	15 to 95 plus	64·9 (59·5–69·7)	35·1 (30·3–40·5)	100 (100–100)
Romania	15 to 39	31·6 (26·8–35·7)	17·9 (13·8–21·6)	49·5 (44·0–55·2)
Romania	40 to 64	29·0 (26·8–31·5)	12·5 (9·89–16·0)	41·5 (38·3–45·2)
Romania	65 plus	7·08 (5·35–9·09)	1·90 (0·856–3·53)	8·98 (6·43–12·4)
Romania	15 to 95 plus	67·7 (62·2–72·5)	32·3 (27·5–37·8)	100 (100–100)
Serbia	15 to 39	38·1 (33·8–42·4)	11·9 (9·15–15·0)	50·1 (45·0–54·8)
Serbia	40 to 64	32·1 (29·9–34·3)	7·75 (5·90–10·0)	39·8 (36·8–43·0)
Serbia	65 plus	8·88 (6·96–11·2)	1·23 (0·539–2·29)	10·1 (7·61–13·4)

Serbia	15 to 95 plus	79·1 (75·2–82·7)	20·9 (17·3–24·8)	100 (100–100)
Slovakia	15 to 39	31·3 (26·6–35·2)	22·4 (17·7–26·6)	53·7 (48·0–58·8)
Slovakia	40 to 64	25·8 (23·6–28·5)	13·1 (10·7–17·0)	38·9 (35·4–43·0)
Slovakia	65 plus	6·08 (4·67–7·79)	1·31 (0·500–2·82)	7·39 (5·26–10·4)
Slovakia	15 to 95 plus	63·2 (57·5–68·3)	36·8 (31·7–42·5)	100 (100–100)
Slovenia	15 to 39	36·4 (27·5–46·9)	22·9 (16·2–30·4)	59·3 (47·7–73·2)
Slovenia	40 to 64	25·0 (18·8–28·9)	9·93 (4·94–15·4)	34·9 (24·8–42·6)
Slovenia	65 plus	5·03 (1·80–8·37)	0·784 (0·0518–2·40)	5·82 (1·85–10·7)
Slovenia	15 to 95 plus	66·4 (58·6–74·6)	33·6 (25·4–41·4)	100 (100–100)
Eastern Europe	15 to 39	34·6 (29·4–39·3)	24·9 (19·8–28·9)	59·6 (52·2–65·2)
Eastern Europe	40 to 64	23·4 (20·4–26·3)	13·5 (10·8–17·4)	36·9 (32·0–42·0)
Eastern Europe	65 plus	3·02 (2·17–4·78)	0·535 (0·195–1·64)	3·55 (2·41–6·33)
Eastern Europe	15 to 95 plus	61·0 (56·4–66·2)	39·0 (33·8–43·6)	100 (100–100)
Belarus	15 to 39	33·4 (27·8–38·6)	24·9 (19·6–29·4)	58·3 (50·0–64·8)
Belarus	40 to 64	22·7 (19·5–25·9)	15·2 (12·2–19·3)	37·9 (32·4–43·8)
Belarus	65 plus	2·93 (2·00–4·64)	0·852 (0·326–2·27)	3·78 (2·36–6·97)
Belarus	15 to 95 plus	59·0 (54·2–64·2)	41·0 (35·8–45·8)	100 (100–100)
Estonia	15 to 39	32·9 (27·7–37·2)	25·6 (21·1–29·3)	58·5 (50·5–64·3)
Estonia	40 to 64	22·4 (19·7–24·9)	14·8 (12·1–18·4)	37·2 (32·4–42·0)
Estonia	65 plus	3·49 (2·42–6·00)	0·840 (0·258–2·72)	4·33 (2·74–9·01)
Estonia	15 to 95 plus	58·8 (55·1–63·1)	41·2 (36·9–44·9)	100 (100–100)
Latvia	15 to 39	30·5 (25·8–34·2)	23·3 (19·2–26·7)	53·8 (46·7–59·1)
Latvia	40 to 64	24·4 (22·1–26·7)	15·8 (13·0–19·5)	40·2 (36·0–44·5)
Latvia	65 plus	4·80 (3·81–6·67)	1·16 (0·473–3·35)	5·96 (4·36–9·90)
Latvia	15 to 95 plus	59·7 (55·6–64·0)	40·3 (36·0–44·4)	100 (100–100)
Lithuania	15 to 39	31·7 (26·1–36·3)	26·0 (20·4–30·1)	57·7 (49·1–64·3)
Lithuania	40 to 64	22·7 (19·6–25·5)	15·0 (12·0–19·2)	37·7 (32·2–43·4)
Lithuania	65 plus	3·55 (2·49–5·77)	1·03 (0·370–3·20)	4·58 (2·94–9·00)
Lithuania	15 to 95 plus	58·0 (53·7–62·8)	42·0 (37·2–46·3)	100 (100–100)
Republic of Moldova	15 to 39	35·4 (30·3–39·9)	24·5 (20·3–28·1)	59·9 (52·8–65·7)
Republic of Moldova	40 to 64	22·3 (19·4–24·9)	14·0 (11·6–17·1)	36·3 (31·6–41·2)

Republic of Moldova	65 plus	3·00 (2·06–4·94)	0·839 (0·327–2·21)	3·84 (2·44–7·20)
Republic of Moldova	15 to 95 plus	60·7 (56·9–65·0)	39·3 (35·0–43·1)	100 (100–100)
Russian Federation	15 to 39	34·4 (29·2–39·0)	24·8 (19·7–28·9)	59·2 (52·4–64·5)
Russian Federation	40 to 64	23·9 (21·0–26·7)	13·3 (10·6–17·4)	37·2 (32·7–41·9)
Russian Federation	65 plus	3·09 (2·23–4·77)	0·465 (0·130–1·51)	3·55 (2·38–6·26)
Russian Federation	15 to 95 plus	61·4 (56·6–66·7)	38·6 (33·3–43·4)	100 (100–100)
Ukraine	15 to 39	36·1 (29·7–41·7)	25·4 (19·9–30·1)	61·4 (52·4–69·0)
Ukraine	40 to 64	22·0 (18·1–25·6)	13·3 (10·4–17·3)	35·3 (29·1–41·7)
Ukraine	65 plus	2·64 (1·61–4·83)	0·575 (0·181–1·69)	3·22 (1·84–6·45)
Ukraine	15 to 95 plus	60·7 (56·1–66·2)	39·3 (33·8–43·9)	100 (100–100)
Australasia	15 to 39	28·3 (24·6–31·9)	26·3 (22·5–29·7)	54·6 (47·2–61·1)
Australasia	40 to 64	18·8 (16·7–21·2)	17·9 (14·6–21·4)	36·8 (32·5–41·4)
Australasia	65 plus	5·12 (3·68–7·10)	3·54 (2·20–5·11)	8·67 (6·13–12·0)
Australasia	15 to 95 plus	52·3 (49·4–55·6)	47·7 (44·4–50·6)	100 (100–100)
Australia	15 to 39	28·2 (24·4–31·8)	26·4 (22·5–29·8)	54·5 (47·1–61·0)
Australia	40 to 64	19·0 (16·9–21·4)	17·8 (14·3–21·4)	36·8 (32·4–41·4)
Australia	65 plus	5·22 (3·76–7·20)	3·47 (2·06–5·05)	8·68 (6·12–12·2)
Australia	15 to 95 plus	52·4 (49·5–56·0)	47·6 (44·0–50·5)	100 (100–100)
New Zealand	15 to 39	28·9 (25·1–32·3)	25·8 (22·3–29·1)	54·7 (47·6–60·9)
New Zealand	40 to 64	18·0 (15·5–20·8)	18·7 (15·9–21·9)	36·7 (32·7–41·3)
New Zealand	65 plus	4·64 (3·25–6·64)	3·92 (2·67–5·30)	8·57 (6·03–11·9)
New Zealand	15 to 95 plus	51·5 (49·0–54·0)	48·5 (46·0–51·0)	100 (100–100)
High-income Asia Pacific	15 to 39	25·6 (22·0–28·5)	18·2 (14·0–21·1)	43·8 (36·7–48·7)
High-income Asia Pacific	40 to 64	25·4 (23·0–28·8)	17·2 (15·1–20·3)	42·6 (39·5–47·3)
High-income Asia Pacific	65 plus	8·76 (6·75–11·1)	4·84 (3·53–6·46)	13·6 (10·5–17·6)
High-income Asia Pacific	15 to 95 plus	59·7 (56·3–63·0)	40·3 (37·0–43·7)	100 (100–100)
Brunei Darussalam	15 to 39	53·1 (41·9–66·5)	19·7 (14·1–26·5)	72·8 (61·2–90·7)
Brunei Darussalam	40 to 64	17·0 (4·05–25·4)	8·54 (4·72–13·3)	25·6 (9·09–36·5)
Brunei Darussalam	65 plus	1·11 (0·0469–2·17)	0·529 (0·0770–0·975)	1·64 (0·134–3·02)
Brunei Darussalam	15 to 95 plus	71·2 (64·2–77·2)	28·8 (22·8–35·8)	100 (100–100)
Japan	15 to 39	23·7 (19·6–27·0)	19·2 (14·5–22·6)	43·0 (34·7–48·8)

Japan	40 to 64	23·4 (20·8–26·8)	18·3 (15·8–21·8)	41·7 (38·2–46·8)
Japan	65 plus	9·65 (7·16–12·6)	5·68 (4·03–7·71)	15·3 (11·4–20·3)
Japan	15 to 95 plus	56·8 (53·1–60·3)	43·2 (39·7–46·9)	100 (100–100)
Republic of Korea	15 to 39	30·2 (27·4–32·6)	14·9 (11·8–17·7)	45·1 (40·6–48·6)
Republic of Korea	40 to 64	31·2 (28·8–34·2)	14·4 (12·1–17·4)	45·6 (42·7–49·3)
Republic of Korea	65 plus	6·63 (5·62–7·89)	2·68 (1·95–3·54)	9·31 (7·77–10·9)
Republic of Korea	15 to 95 plus	68·0 (64·7–71·2)	32·0 (28·8–35·3)	100 (100–100)
Singapore	15 to 39	37·0 (29·5–42·3)	24·1 (17·5–29·9)	61·1 (48·7–68·6)
Singapore	40 to 64	22·2 (17·0–29·3)	13·4 (10·2–17·9)	35·6 (29·1–46·3)
Singapore	65 plus	2·39 (1·33–3·95)	0·940 (0·507–1·61)	3·33 (1·92–5·39)
Singapore	15 to 95 plus	61·6 (55·8–66·8)	38·4 (33·2–44·2)	100 (100–100)
High-income North America	15 to 39	32·3 (27·9–36·2)	25·2 (20·3–28·5)	57·5 (50·5–63·4)
High-income North America	40 to 64	20·1 (17·8–22·6)	14·5 (12·4–17·3)	34·6 (30·8–39·2)
High-income North America	65 plus	4·77 (3·52–6·74)	3·18 (2·02–4·81)	7·95 (5·58–11·4)
High-income North America	15 to 95 plus	57·2 (53·8–60·9)	42·8 (39·1–46·2)	100 (100–100)
Canada	15 to 39	31·6 (26·9–35·9)	24·7 (19·4–28·6)	56·3 (48·7–62·6)
Canada	40 to 64	20·7 (18·1–23·7)	14·8 (12·4–17·9)	35·6 (31·3–40·8)
Canada	65 plus	5·08 (3·67–7·30)	3·02 (1·72–4·85)	8·11 (5·49–11·9)
Canada	15 to 95 plus	57·4 (53·5–61·7)	42·6 (38·3–46·5)	100 (100–100)
Greenland	15 to 39	35·8 (30·7–42·3)	26·2 (20·3–30·7)	62·0 (54·0–70·6)
Greenland	40 to 64	21·9 (17·6–25·7)	11·9 (8·70–15·2)	33·8 (27·0–39·8)
Greenland	65 plus	3·03 (1·74–4·50)	1·21 (0·505–2·07)	4·25 (2·30–6·41)
Greenland	15 to 95 plus	60·8 (56·9–65·5)	39·2 (34·5–43·1)	100 (100–100)
United States of America	15 to 39	32·4 (28·0–36·4)	25·3 (20·4–28·5)	57·6 (50·7–63·6)
United States of America	40 to 64	20·0 (17·7–22·5)	14·4 (12·4–17·2)	34·4 (30·6–39·0)
United States of America	65 plus	4·73 (3·48–6·70)	3·20 (2·03–4·80)	7·93 (5·56–11·4)
United States of America	15 to 95 plus	57·1 (53·8–60·8)	42·9 (39·2–46·2)	100 (100–100)
Southern Latin America	15 to 39	36·4 (32·1–40·6)	28·0 (24·0–30·9)	64·5 (58·4–69·0)
Southern Latin America	40 to 64	17·9 (15·8–20·4)	11·9 (9·84–15·3)	29·8 (26·3–35·1)
Southern Latin America	65 plus	3·65 (2·66–4·73)	2·07 (1·34–3·00)	5·72 (4·11–7·66)
Southern Latin America	15 to 95 plus	58·0 (54·8–62·2)	42·0 (37·8–45·2)	100 (100–100)

Argentina	15 to 39	37·3 (32·7–41·5)	29·3 (25·2–32·5)	66·5 (60·1–71·3)
Argentina	40 to 64	17·0 (14·7–19·6)	11·3 (9·01–14·8)	28·2 (24·4–33·8)
Argentina	65 plus	3·33 (2·33–4·38)	1·89 (1·14–2·87)	5·22 (3·64–7·18)
Argentina	15 to 95 plus	57·6 (54·0–62·0)	42·4 (38·0–46·0)	100 (100–100)
Chile	15 to 39	34·6 (30·5–38·8)	25·8 (21·5–28·8)	60·4 (54·6–65·0)
Chile	40 to 64	19·8 (17·7–22·1)	13·3 (11·1–16·5)	33·1 (29·4–37·8)
Chile	65 plus	4·21 (3·20–5·30)	2·35 (1·54–3·28)	6·56 (4·85–8·40)
Chile	15 to 95 plus	58·6 (55·4–62·8)	41·4 (37·2–44·6)	100 (100–100)
Uruguay	15 to 39	35·1 (30·5–39·4)	23·4 (19·6–26·8)	58·5 (52·3–63·8)
Uruguay	40 to 64	20·5 (18·1–23·0)	12·9 (10·6–16·3)	33·3 (29·7–38·4)
Uruguay	65 plus	5·12 (3·83–6·54)	3·04 (1·92–4·42)	8·16 (5·89–10·8)
Uruguay	15 to 95 plus	60·7 (57·1–64·5)	39·3 (35·5–42·9)	100 (100–100)
Western Europe	15 to 39	26·6 (23·2–30·0)	20·6 (17·0–23·6)	47·3 (41·4–51·7)
Western Europe	40 to 64	22·8 (20·7–25·1)	18·0 (15·6–21·6)	40·9 (38·2–44·8)
Western Europe	65 plus	7·19 (5·53–9·27)	4·67 (3·36–6·04)	11·9 (8·91–15·1)
Western Europe	15 to 95 plus	56·7 (52·5–61·1)	43·3 (38·9–47·5)	100 (100–100)
Andorra	15 to 39	25·1 (21·6–28·6)	19·6 (15·9–22·7)	44·6 (39·1–49·0)
Andorra	40 to 64	26·9 (24·1–30·0)	20·1 (16·8–24·5)	47·0 (43·7–51·3)
Andorra	65 plus	5·39 (3·96–7·12)	2·96 (1·98–3·97)	8·35 (6·12–11·0)
Andorra	15 to 95 plus	57·4 (52·6–62·4)	42·6 (37·6–47·4)	100 (100–100)
Austria	15 to 39	27·7 (23·9–31·2)	20·7 (17·1–23·9)	48·4 (42·7–53·0)
Austria	40 to 64	22·8 (20·5–25·2)	18·3 (15·5–21·9)	41·0 (37·9–45·2)
Austria	65 plus	6·12 (4·55–8·03)	4·42 (3·17–5·77)	10·5 (7·89–13·6)
Austria	15 to 95 plus	56·6 (52·5–61·0)	43·4 (39·0–47·5)	100 (100–100)
Belgium	15 to 39	26·7 (22·9–30·3)	22·0 (18·2–25·3)	48·8 (42·6–53·8)
Belgium	40 to 64	22·0 (19·7–24·3)	18·2 (15·6–22·2)	40·2 (37·2–44·4)
Belgium	65 plus	6·35 (4·58–8·43)	4·66 (3·21–6·31)	11·0 (7·95–14·6)
Belgium	15 to 95 plus	55·1 (50·7–59·7)	44·9 (40·3–49·3)	100 (100–100)
Cyprus	15 to 39	36·0 (31·0–41·1)	22·7 (18·0–26·9)	58·7 (52·3–64·0)
Cyprus	40 to 64	20·8 (18·2–23·7)	13·5 (10·3–17·9)	34·4 (30·8–39·4)
Cyprus	65 plus	5·05 (3·53–6·83)	1·88 (1·10–2·79)	6·93 (4·71–9·48)

Cyprus	15 to 95 plus	61·9 (56·4–68·4)	38·1 (31·6–43·6)	100 (100–100)
Denmark	15 to 39	27·8 (23·2–32·1)	23·2 (18·9–26·8)	51·0 (43·8–56·9)
Denmark	40 to 64	19·7 (17·0–22·4)	18·2 (15·0–22·4)	37·8 (34·0–42·7)
Denmark	65 plus	6·24 (4·26–8·65)	4·97 (3·34–6·81)	11·2 (7·79–15·4)
Denmark	15 to 95 plus	53·7 (48·8–58·5)	46·3 (41·5–51·2)	100 (100–100)
Finland	15 to 39	27·8 (23·3–32·2)	23·1 (18·6–27·0)	50·9 (44·1–56·8)
Finland	40 to 64	20·8 (18·2–23·5)	17·7 (14·7–22·0)	38·4 (34·9–42·8)
Finland	65 plus	6·21 (4·12–8·69)	4·48 (2·82–6·29)	10·7 (7·06–14·9)
Finland	15 to 95 plus	54·8 (50·1–60·2)	45·2 (39·8–49·9)	100 (100–100)
France	15 to 39	26·5 (22·7–30·0)	21·3 (17·3–24·5)	47·8 (42·1–52·6)
France	40 to 64	21·8 (19·7–23·9)	17·6 (14·8–21·4)	39·4 (36·4–43·2)
France	65 plus	7·96 (6·15–10·1)	4·86 (3·34–6·52)	12·8 (9·68–16·6)
France	15 to 95 plus	56·2 (51·8–61·1)	43·8 (38·9–48·2)	100 (100–100)
Germany	15 to 39	25·6 (22·4–28·5)	19·5 (16·0–22·4)	45·1 (40·2–49·3)
Germany	40 to 64	22·8 (21·1–25·0)	18·5 (16·1–22·0)	41·4 (39·0–44·6)
Germany	65 plus	7·93 (6·33–9·77)	5·60 (4·15–7·25)	13·5 (10·5–16·9)
Germany	15 to 95 plus	56·4 (52·6–60·3)	43·6 (39·7–47·4)	100 (100–100)
Greece	15 to 39	27·8 (23·1–32·6)	22·1 (17·5–26·2)	49·9 (42·7–56·0)
Greece	40 to 64	23·7 (20·7–27·0)	17·0 (13·1–22·0)	40·7 (36·8–45·7)
Greece	65 plus	6·73 (4·68–9·39)	2·68 (1·49–4·14)	9·41 (6·37–13·3)
Greece	15 to 95 plus	58·3 (52·5–64·8)	41·7 (35·2–47·5)	100 (100–100)
Iceland	15 to 39	31·2 (26·7–35·9)	24·7 (19·9–28·7)	55·9 (49·2–61·3)
Iceland	40 to 64	19·7 (17·0–22·9)	16·6 (13·7–20·8)	36·3 (32·4–41·4)
Iceland	65 plus	4·73 (3·19–6·62)	3·03 (1·93–4·24)	7·76 (5·22–10·6)
Iceland	15 to 95 plus	55·6 (50·7–61·4)	44·4 (38·6–49·3)	100 (100–100)
Ireland	15 to 39	28·1 (24·6–31·4)	25·1 (21·7–28·0)	53·1 (47·6–57·5)
Ireland	40 to 64	20·7 (18·5–23·1)	18·5 (15·8–22·0)	39·2 (36·2–43·1)
Ireland	65 plus	4·64 (3·33–6·24)	3·00 (2·02–4·01)	7·64 (5·45–10·0)
Ireland	15 to 95 plus	53·4 (49·3–57·6)	46·6 (42·4–50·7)	100 (100–100)
Israel	15 to 39	41·0 (33·0–49·5)	28·9 (22·2–34·7)	69·9 (59·0–77·7)
Israel	40 to 64	14·2 (10·2–19·9)	13·0 (8·63–18·9)	27·2 (20·4–36·9)

Israel	65 plus	1·74 (0·685–3·42)	1·22 (0·537–2·09)	2·96 (1·32–5·41)
Israel	15 to 95 plus	56·9 (49·3–65·2)	43·1 (34·8–50·7)	100 (100–100)
Italy	15 to 39	24·4 (20·8–27·6)	16·2 (12·5–19·3)	40·6 (35·1–45·7)
Italy	40 to 64	26·4 (24·0–28·9)	18·3 (15·7–21·8)	44·8 (42·0–48·4)
Italy	65 plus	8·93 (6·90–11·5)	5·71 (4·37–7·25)	14·6 (11·4–18·4)
Italy	15 to 95 plus	59·7 (55·4–64·2)	40·3 (35·8–44·6)	100 (100–100)
Luxembourg	15 to 39	28·4 (24·6–32·1)	22·6 (18·4–25·9)	51·0 (45·1–55·5)
Luxembourg	40 to 64	22·8 (20·4–25·5)	17·5 (14·7–21·4)	40·3 (37·2–44·5)
Luxembourg	65 plus	5·52 (4·15–7·11)	3·14 (2·09–4·37)	8·65 (6·34–11·2)
Luxembourg	15 to 95 plus	56·7 (52·3–61·8)	43·3 (38·2–47·7)	100 (100–100)
Malta	15 to 39	31·6 (26·6–36·6)	22·9 (17·7–27·1)	54·5 (47·3–60·7)
Malta	40 to 64	21·9 (19·1–25·2)	14·0 (10·9–18·5)	35·9 (32·0–40·8)
Malta	65 plus	6·45 (4·40–8·81)	3·14 (1·90–4·61)	9·59 (6·55–13·2)
Malta	15 to 95 plus	60·0 (54·4–66·6)	40·0 (33·4–45·6)	100 (100–100)
Monaco	15 to 39	27·2 (18·8–53·6)	22·0 (15·4–36·5)	49·2 (35·2–84·4)
Monaco	40 to 64	21·5 (6·87–26·6)	19·0 (5·75–26·4)	40·5 (15·1–48·7)
Monaco	65 plus	6·14 (0·102–11·2)	4·16 (0·0378–8·07)	10·3 (0·136–19·0)
Monaco	15 to 95 plus	54·9 (45·5–65·2)	45·1 (34·8–54·5)	100 (100–100)
Netherlands	15 to 39	27·1 (23·1–31·0)	21·3 (17·2–24·8)	48·4 (41·7–54·0)
Netherlands	40 to 64	21·5 (18·9–24·2)	18·2 (15·3–22·3)	39·8 (36·4–44·3)
Netherlands	65 plus	6·76 (4·90–9·18)	5·06 (3·61–6·78)	11·8 (8·69–15·9)
Netherlands	15 to 95 plus	55·4 (50·9–59·9)	44·6 (40·1–49·1)	100 (100–100)
Norway	15 to 39	30·3 (25·4–35·1)	24·6 (20·2–28·5)	54·8 (47·2–61·3)
Norway	40 to 64	19·6 (16·7–22·9)	17·5 (14·5–22·0)	37·1 (33·0–42·7)
Norway	65 plus	4·51 (2·76–6·79)	3·52 (2·13–4·98)	8·03 (4·92–11·7)
Norway	15 to 95 plus	54·4 (49·4–59·8)	45·6 (40·2–50·6)	100 (100–100)
Portugal	15 to 39	25·8 (22·6–28·9)	16·5 (13·0–19·6)	42·4 (37·6–46·4)
Portugal	40 to 64	27·2 (25·0–29·8)	17·0 (14·1–20·8)	44·2 (41·5–47·4)
Portugal	65 plus	9·26 (7·39–11·5)	4·15 (2·92–5·64)	13·4 (10·6–16·6)
Portugal	15 to 95 plus	62·3 (57·8–66·9)	37·7 (33·1–42·2)	100 (100–100)
San Marino	15 to 39	26·2 (19·8–56·1)	21·5 (16·4–38·8)	47·7 (37·4–95·2)

San Marino	40 to 64	21·6 (0·726–25·9)	20·2 (1·79–26·5)	41·8 (4·79–49·1)
San Marino	65 plus	6·45 (0–9·67)	4·10 (0–6·54)	10·6 (0–15·9)
San Marino	15 to 95 plus	54·3 (45·3–64·9)	45·7 (35·1–54·7)	100 (100–100)
Spain	15 to 39	25·6 (22·4–28·7)	19·0 (15·8–21·9)	44·7 (39·6–48·7)
Spain	40 to 64	27·2 (24·7–30·0)	18·5 (15·5–22·3)	45·7 (42·9–49·5)
Spain	65 plus	6·22 (4·68–8·23)	3·38 (2·33–4·55)	9·60 (7·19–12·6)
Spain	15 to 95 plus	59·1 (54·7–63·4)	40·9 (36·6–45·3)	100 (100–100)
Sweden	15 to 39	28·8 (24·3–32·9)	23·1 (18·9–26·7)	51·9 (44·8–57·7)
Sweden	40 to 64	19·5 (16·9–22·1)	18·1 (15·3–22·0)	37·6 (34·1–42·1)
Sweden	65 plus	5·68 (3·74–8·20)	4·85 (3·32–6·65)	10·5 (7·18–14·7)
Sweden	15 to 95 plus	54·0 (49·4–58·7)	46·0 (41·3–50·6)	100 (100–100)
Switzerland	15 to 39	27·5 (23·4–31·7)	21·4 (17·1–25·3)	49·0 (42·3–54·6)
Switzerland	40 to 64	22·5 (19·8–25·4)	18·1 (15·2–22·3)	40·6 (37·3–45·1)
Switzerland	65 plus	6·15 (4·42–8·35)	4·25 (2·81–5·89)	10·4 (7·36–14·1)
Switzerland	15 to 95 plus	56·2 (51·2–61·3)	43·8 (38·7–48·8)	100 (100–100)
United Kingdom	15 to 39	27·7 (24·1–31·2)	23·6 (19·9–26·7)	51·3 (45·3–56·4)
United Kingdom	40 to 64	20·5 (18·3–22·7)	17·9 (15·4–21·7)	38·4 (35·6–42·2)
United Kingdom	65 plus	6·30 (4·55–8·41)	4·07 (2·70–5·67)	10·4 (7·42–14·0)
United Kingdom	15 to 95 plus	54·5 (50·6–58·8)	45·5 (41·2–49·4)	100 (100–100)
Andean Latin America	15 to 39	47·2 (40·4–53·6)	23·1 (17·5–28·0)	70·3 (65·5–74·4)
Andean Latin America	40 to 64	19·9 (17·7–22·7)	6·65 (4·76–8·97)	26·5 (23·1–30·8)
Andean Latin America	65 plus	2·65 (1·90–3·34)	0·522 (0·267–0·859)	3·17 (2·20–4·13)
Andean Latin America	15 to 95 plus	69·8 (63·6–75·8)	30·2 (24·2–36·4)	100 (100–100)
Bolivia (Plurinational State of)	15 to 39	48·0 (40·0–55·4)	24·7 (17·9–31·0)	72·7 (67·1–77·4)
Bolivia (Plurinational State of)	40 to 64	18·3 (15·7–21·4)	6·16 (4·16–8·77)	24·4 (20·3–29·2)
Bolivia (Plurinational State of)	65 plus	2·43 (1·61–3·27)	0·449 (0·183–0·846)	2·88 (1·85–4·01)
Bolivia (Plurinational State of)	15 to 95 plus	68·7 (61·2–76·4)	31·3 (23·6–38·8)	100 (100–100)
Ecuador	15 to 39	55·4 (48·7–61·3)	16·0 (11·3–20·9)	71·4 (66·8–75·6)
Ecuador	40 to 64	22·1 (19·1–25·5)	3·89 (2·63–5·67)	26·0 (22·3–30·4)
Ecuador	65 plus	2·37 (1·68–3·05)	0·193 (0·0948–0·333)	2·56 (1·79–3·35)
Ecuador	15 to 95 plus	79·9 (74·6–85·0)	20·1 (15·0–25·4)	100 (100–100)

Peru	15 to 39	44·5<br>(37·8–51·2)	24·8<br>(18·9–29·6)	69·2<br>(64·4–73·7)
Peru	40 to 64	19·7<br>(17·5–22·4)	7·64<br>(5·40–10·4)	27·3<br>(23·6–31·4)
Peru	65 plus	2·79<br>(2·00–3·52)	0·645<br>(0·316–1·06)	3·44<br>(2·37–4·44)
Peru	15 to 95 plus	67·0<br>(60·7–73·7)	33·0<br>(26·3–39·3)	100<br>(100–100)
Caribbean	15 to 39	52·4<br>(45·9–58·7)	20·9<br>(16·0–25·4)	73·3<br>(67·6–78·3)
Caribbean	40 to 64	19·5<br>(16·6–22·6)	4·39<br>(2·82–6·03)	23·9<br>(19·7–28·5)
Caribbean	65 plus	2·55<br>(1·72–3·67)	0·270<br>(0·112–0·576)	2·82<br>(1·85–4·26)
Caribbean	15 to 95 plus	74·5<br>(69·5–79·8)	25·5<br>(20·2–30·5)	100<br>(100–100)
Antigua and Barbuda	15 to 39	48·9<br>(42·0–55·4)	20·5<br>(15·0–26·2)	69·4<br>(63·4–75·1)
Antigua and Barbuda	40 to 64	22·1<br>(18·8–25·7)	5·53<br>(3·30–7·89)	27·6<br>(22·9–32·5)
Antigua and Barbuda	65 plus	2·75<br>(1·81–3·91)	0·284<br>(0·0879–0·640)	3·03<br>(1·94–4·50)
Antigua and Barbuda	15 to 95 plus	73·7<br>(67·6–80·1)	26·3<br>(19·9–32·4)	100<br>(100–100)
Bahamas	15 to 39	51·9<br>(42·8–62·5)	21·7<br>(14·5–28·2)	73·5<br>(65·9–81·5)
Bahamas	40 to 64	19·9<br>(15·1–24·0)	4·57<br>(1·85–7·87)	24·5<br>(17·6–30·9)
Bahamas	65 plus	1·78<br>(0·829–2·93)	0·193<br>(0·0257–0·546)	1·98<br>(0·858–3·41)
Bahamas	15 to 95 plus	73·5<br>(66·4–82·1)	26·5<br>(17·9–33·6)	100<br>(100–100)
Barbados	15 to 39	39·2<br>(34·9–43·4)	20·6<br>(16·1–24·9)	59·8<br>(55·0–64·4)
Barbados	40 to 64	25·1<br>(22·7–27·6)	8·72<br>(6·51–11·1)	33·8<br>(30·2–37·4)
Barbados	65 plus	5·44<br>(4·22–6·83)	0·981<br>(0·520–1·68)	6·42<br>(4·85–8·30)
Barbados	15 to 95 plus	69·7<br>(65·3–74·2)	30·3<br>(25·8–34·7)	100<br>(100–100)
Belize	15 to 39	55·1<br>(48·6–61·7)	23·3<br>(17·4–29·0)	78·4<br>(74·1–82·6)
Belize	40 to 64	16·4<br>(13·8–19·4)	3·67<br>(2·27–5·20)	20·1<br>(16·2–23·9)
Belize	65 plus	1·45<br>(0·938–2·14)	0·0963<br>(0·0341–0·204)	1·55<br>(0·994–2·30)
Belize	15 to 95 plus	72·9<br>(67·1–79·2)	27·1<br>(20·8–32·9)	100<br>(100–100)
Bermuda	15 to 39	35·3<br>(29·9–41·3)	18·0<br>(13·4–22·4)	53·3<br>(47·3–59·9)
Bermuda	40 to 64	30·1<br>(27·4–33·2)	9·10<br>(5·97–12·0)	39·2<br>(34·4–43·3)
Bermuda	65 plus	6·38<br>(4·81–8·16)	1·08<br>(0·405–2·12)	7·46<br>(5·29–10·2)
Bermuda	15 to 95 plus	71·8<br>(66·7–77·5)	28·2<br>(22·5–33·3)	100<br>(100–100)
Cuba	15 to 39	45·7<br>(38·9–52·6)	17·4<br>(12·2–22·6)	63·2<br>(56·1–69·7)
Cuba	40 to 64	26·5<br>(22·5–30·6)	5·85<br>(3·62–8·33)	32·3<br>(27·0–37·6)
Cuba	65 plus	4·10<br>(2·74–5·87)	0·421<br>(0·151–0·950)	4·52<br>(2·93–6·77)

Cuba	15 to 95 plus	76·3 (70·7–82·1)	23·7 (17·9–29·3)	100 (100–100)
Dominica	15 to 39	49·4 (42·3–56·3)	20·0 (14·1–25·6)	69·4 (63·1–75·6)
Dominica	40 to 64	22·0 (18·5–25·4)	4·88 (2·82–7·07)	26·9 (21·9–31·9)
Dominica	65 plus	3·34 (2·18–4·80)	0·386 (0·120–0·903)	3·73 (2·34–5·59)
Dominica	15 to 95 plus	74·7 (68·6–81·1)	25·3 (18·9–31·4)	100 (100–100)
Dominican Republic	15 to 39	53·2 (46·8–59·4)	25·1 (20·0–29·5)	78·3 (72·9–83·1)
Dominican Republic	40 to 64	15·7 (12·8–19·0)	4·37 (2·85–6·03)	20·1 (15·9–24·6)
Dominican Republic	65 plus	1·42 (0·826–2·34)	0·209 (0·0805–0·439)	1·63 (0·915–2·77)
Dominican Republic	15 to 95 plus	70·3 (65·8–75·6)	29·7 (24·4–34·2)	100 (100–100)
Grenada	15 to 39	47·7 (42·8–52·5)	20·0 (15·4–24·7)	67·7 (63·5–71·8)
Grenada	40 to 64	22·4 (19·8–25·0)	5·84 (4·23–7·74)	28·2 (24·7–31·7)
Grenada	65 plus	3·48 (2·70–4·38)	0·602 (0·303–1·06)	4·08 (3·08–5·30)
Grenada	15 to 95 plus	73·6 (68·8–78·5)	26·4 (21·5–31·2)	100 (100–100)
Guyana	15 to 39	55·4 (48·0–62·6)	20·2 (13·6–26·5)	75·6 (70·3–80·4)
Guyana	40 to 64	19·4 (16·3–23·0)	3·11 (1·63–4·83)	22·5 (18·3–27·1)
Guyana	65 plus	1·72 (1·00–2·64)	0·133 (0·0361–0·338)	1·85 (1·04–2·93)
Guyana	15 to 95 plus	76·6 (69·8–83·5)	23·4 (16·5–30·2)	100 (100–100)
Haiti	15 to 39	60·2 (53·9–66·5)	19·6 (14·1–24·9)	79·8 (75·6–83·7)
Haiti	40 to 64	15·8 (13·2–18·7)	2·78 (1·63–4·04)	18·6 (15·3–22·1)
Haiti	65 plus	1·48 (0·964–2·11)	0·126 (0·0472–0·277)	1·61 (1·03–2·35)
Haiti	15 to 95 plus	77·4 (72·0–83·3)	22·6 (16·7–28·0)	100 (100–100)
Jamaica	15 to 39	57·8 (49·5–66·0)	20·6 (14·1–27·2)	78·3 (72·4–83·6)
Jamaica	40 to 64	17·1 (13·5–21·1)	2·52 (1·24–3·95)	19·6 (15·1–24·5)
Jamaica	65 plus	1·94 (1·08–3·16)	0·101 (0·0256–0·273)	2·04 (1·10–3·40)
Jamaica	15 to 95 plus	76·8 (69·9–83·6)	23·2 (16·4–30·1)	100 (100–100)
Puerto Rico	15 to 39	44·2 (38·2–50·4)	21·4 (16·1–26·1)	65·6 (59·8–71·1)
Puerto Rico	40 to 64	23·0 (20·3–25·9)	5·43 (3·48–7·46)	28·4 (24·3–32·5)
Puerto Rico	65 plus	5·57 (3·97–7·57)	0·439 (0·156–0·962)	6·01 (4·16–8·40)
Puerto Rico	15 to 95 plus	72·8 (67·4–78·5)	27·2 (21·5–32·6)	100 (100–100)
Saint Kitts and Nevis	15 to 39	54·7 (36·1–92·1)	22·1 (4·72–31·7)	76·7 (59·6–99·7)
Saint Kitts and Nevis	40 to 64	17·0 (0·283–25·5)	4·49 (0–11·9)	21·5 (0·283–36·3)

Saint Kitts and Nevis	65 plus	1·53 (0–3·57)	0·250 (0–1·11)	1·78 (0–4·58)
Saint Kitts and Nevis	15 to 95 plus	73·2 (61·9–95·1)	26·8 (4·88–38·1)	100 (100–100)
Saint Lucia	15 to 39	39·8 (34·9–44·5)	24·6 (20·5–28·7)	64·4 (59·0–69·2)
Saint Lucia	40 to 64	21·6 (19·3–24·4)	10·2 (7·89–12·7)	31·8 (27·8–35·9)
Saint Lucia	65 plus	3·08 (2·26–4·09)	0·719 (0·383–1·27)	3·80 (2·68–5·26)
Saint Lucia	15 to 95 plus	64·5 (60·3–68·7)	35·5 (31·3–39·7)	100 (100–100)
Saint Vincent and the Grenadines	15 to 39	46·2 (39·2–53·3)	21·9 (15·8–27·4)	68·0 (61·5–74·1)
Saint Vincent and the Grenadines	40 to 64	22·3 (19·1–25·4)	6·00 (3·60–8·58)	28·3 (23·4–33·1)
Saint Vincent and the Grenadines	65 plus	3·34 (2·24–4·69)	0·361 (0·111–0·836)	3·70 (2·38–5·49)
Saint Vincent and the Grenadines	15 to 95 plus	71·8 (65·6–78·7)	28·2 (21·3–34·4)	100 (100–100)
Suriname	15 to 39	53·0 (45·2–60·6)	20·9 (14·3–27·2)	73·8 (67·3–79·6)
Suriname	40 to 64	19·8 (16·3–23·7)	4·18 (2·24–6·27)	24·0 (19·0–29·5)
Suriname	65 plus	1·94 (1·13–3·08)	0·196 (0·0532–0·498)	2·14 (1·19–3·50)
Suriname	15 to 95 plus	74·7 (67·8–81·9)	25·3 (18·1–32·2)	100 (100–100)
Trinidad and Tobago	15 to 39	47·3 (40·1–54·4)	19·8 (13·8–25·4)	67·1 (60·7–73·3)
Trinidad and Tobago	40 to 64	23·1 (19·8–26·8)	5·73 (3·46–8·15)	28·8 (23·9–33·9)
Trinidad and Tobago	65 plus	3·64 (2·44–5·09)	0·463 (0·153–1·10)	4·10 (2·63–6·22)
Trinidad and Tobago	15 to 95 plus	74·0 (67·6–80·6)	26·0 (19·4–32·4)	100 (100–100)
United States Virgin Islands	15 to 39	44·2 (29·7–82·0)	19·2 (8·72–26·9)	63·3 (48·2–98·3)
United States Virgin Islands	40 to 64	21·8 (1·65–26·8)	7·75 (0·0118–13·8)	29·6 (1·69–39·2)
United States Virgin Islands	65 plus	5·33 (0·0207–8·76)	1·79 (0–4·43)	7·13 (0·0207–13·3)
United States Virgin Islands	15 to 95 plus	71·3 (62·4–90·8)	28·7 (9·17–37·6)	100 (100–100)
Central Latin America	15 to 39	51·4 (45·6–56·6)	20·0 (14·6–24·0)	71·4 (66·6–75·1)
Central Latin America	40 to 64	22·3 (20·2–25·3)	3·54 (2·24–4·97)	25·8 (22·8–29·9)
Central Latin America	65 plus	2·55 (1·85–3·44)	0·199 (0·0789–0·412)	2·75 (1·95–3·77)
Central Latin America	15 to 95 plus	76·2 (71·6–81·9)	23·8 (18·1–28·4)	100 (100–100)
Colombia	15 to 39	51·4 (44·5–57·8)	22·4 (16·6–27·0)	73·8 (68·3–78·0)
Colombia	40 to 64	20·1 (17·6–23·4)	3·86 (2·29–5·71)	24·0 (20·3–28·5)
Colombia	65 plus	2·01 (1·26–3·08)	0·175 (0·0477–0·435)	2·18 (1·34–3·52)
Colombia	15 to 95 plus	73·6 (68·2–79·8)	26·4 (20·2–31·8)	100 (100–100)
Costa Rica	15 to 39	49·1 (40·8–56·6)	21·9 (14·7–28·1)	71·0 (63·9–76·3)

Costa Rica	40 to 64	21·8 (18·8–26·0)	3·99 (2·12–6·38)	25·8 (21·4–31·2)
Costa Rica	65 plus	2·90 (1·83–4·48)	0·257 (0·0650–0·667)	3·16 (1·92–5·07)
Costa Rica	15 to 95 plus	73·9 (66·7–81·6)	26·1 (18·4–33·3)	100 (100–100)
El Salvador	15 to 39	64·1 (57·1–69·7)	10·3 (6·34–14·2)	74·3 (68·3–78·7)
El Salvador	40 to 64	21·2 (18·1–25·6)	1·79 (0·949–2·91)	23·0 (19·3–28·0)
El Salvador	65 plus	2·54 (1·60–3·79)	0·103 (0·0274–0·265)	2·65 (1·64–4·06)
El Salvador	15 to 95 plus	87·8 (83·6–92·0)	12·2 (8·02–16·4)	100 (100–100)
Guatemala	15 to 39	64·1 (56·3–71·0)	14·7 (9·37–19·7)	78·8 (72·8–83·1)
Guatemala	40 to 64	17·5 (14·4–21·6)	1·93 (0·879–3·40)	19·5 (15·7–24·6)
Guatemala	65 plus	1·65 (0·923–2·73)	0·106 (0·0187–0·306)	1·76 (0·947–3·00)
Guatemala	15 to 95 plus	83·3 (77·4–88·9)	16·7 (11·1–22·6)	100 (100–100)
Honduras	15 to 39	63·0 (57·1–68·0)	11·8 (8·04–15·8)	74·8 (70·2–78·8)
Honduras	40 to 64	20·7 (17·7–24·2)	2·12 (1·25–3·21)	22·8 (19·4–26·9)
Honduras	65 plus	2·23 (1·52–3·11)	0·152 (0·0533–0·311)	2·38 (1·59–3·40)
Honduras	15 to 95 plus	85·9 (81·4–89·7)	14·1 (10·3–18·6)	100 (100–100)
Mexico	15 to 39	48·8 (43·3–54·1)	21·1 (15·6–25·5)	69·9 (65·3–73·5)
Mexico	40 to 64	23·3 (21·0–26·4)	3·78 (2·34–5·51)	27·1 (23·9–31·0)
Mexico	65 plus	2·82 (2·11–3·74)	0·224 (0·0867–0·443)	3·04 (2·22–4·13)
Mexico	15 to 95 plus	74·9 (69·9–80·9)	25·1 (19·1–30·1)	100 (100–100)
Nicaragua	15 to 39	58·1 (51·0–64·3)	18·6 (12·4–23·8)	76·7 (71·3–80·4)
Nicaragua	40 to 64	18·8 (16·3–22·3)	2·64 (1·45–4·15)	21·4 (18·2–26·2)
Nicaragua	65 plus	1·75 (1·13–2·52)	0·114 (0·0325–0·271)	1·86 (1·18–2·79)
Nicaragua	15 to 95 plus	78·7 (72·6–85·0)	21·3 (15·0–27·4)	100 (100–100)
Panama	15 to 39	50·6 (44·9–55·6)	16·4 (11·8–20·9)	67·0 (62·6–71·0)
Panama	40 to 64	25·3 (22·6–28·4)	3·78 (2·34–5·43)	29·1 (25·8–32·9)
Panama	65 plus	3·58 (2·70–4·84)	0·273 (0·102–0·570)	3·85 (2·82–5·35)
Panama	15 to 95 plus	79·5 (74·6–84·7)	20·5 (15·3–25·4)	100 (100–100)
Venezuela (Bolivarian Republic of)	15 to 39	54·2 (47·0–60·4)	16·8 (11·3–21·8)	70·9 (65·5–75·6)
Venezuela (Bolivarian Republic of)	40 to 64	23·4 (20·4–27·3)	3·06 (1·61–4·81)	26·5 (22·3–31·0)
Venezuela (Bolivarian Republic of)	65 plus	2·45 (1·61–3·41)	0·169 (0·0476–0·397)	2·62 (1·69–3·78)
Venezuela (Bolivarian Republic of)	15 to 95 plus	80·0 (74·2–85·9)	20·0 (14·1–25·8)	100 (100–100)

Tropical Latin America	15 to 39	43·2 (38·3–47·6)	25·2 (20·9–27·9)	68·4 (63·0–72·4)
Tropical Latin America	40 to 64	21·5 (19·5–24·4)	6·70 (4·94–8·69)	28·2 (24·9–32·8)
Tropical Latin America	65 plus	2·82 (2·11–3·65)	0·587 (0·306–0·979)	3·41 (2·43–4·56)
Tropical Latin America	15 to 95 plus	67·5 (64·3–71·6)	32·5 (28·4–35·7)	100 (100–100)
Brazil	15 to 39	42·9 (38·0–47·4)	25·1 (20·9–27·9)	68·1 (62·6–72·1)
Brazil	40 to 64	21·7 (19·7–24·6)	6·79 (5·03–8·81)	28·5 (25·2–33·1)
Brazil	65 plus	2·86 (2·12–3·70)	0·593 (0·310–0·991)	3·45 (2·45–4·61)
Brazil	15 to 95 plus	67·5 (64·2–71·4)	32·5 (28·6–35·8)	100 (100–100)
Paraguay	15 to 39	48·8 (43·3–54·6)	26·6 (19·8–30·2)	75·4 (69·9–79·4)
Paraguay	40 to 64	17·2 (15·4–20·4)	4·79 (3·24–6·48)	22·0 (18·8–26·2)
Paraguay	65 plus	2·14 (1·48–2·92)	0·440 (0·186–0·814)	2·58 (1·69–3·75)
Paraguay	15 to 95 plus	68·2 (64·0–75·0)	31·8 (25·0–36·0)	100 (100–100)
North Africa and Middle East	15 to 39	66·0 (58·3–73·1)	6·07 (4·53–8·56)	72·1 (65·0–78·7)
North Africa and Middle East	40 to 64	23·6 (18·1–29·0)	1·99 (1·06–3·14)	25·6 (19·5–31·7)
North Africa and Middle East	65 plus	2·16 (1·57–3·09)	0·151 (0·0701–0·336)	2·31 (1·65–3·38)
North Africa and Middle East	15 to 95 plus	91·8 (88·7–94·0)	8·21 (6·02–11·3)	100 (100–100)
Afghanistan	15 to 39	91·0 (80·2–97·6)	3·79 (1·18–9·41)	94·8 (87·0–99·3)
Afghanistan	40 to 64	4·97 (0·685–12·2)	0·198 (0·00731–0·718)	5·16 (0·696–12·6)
Afghanistan	65 plus	0·0732 (0·00208–0·365)	0·00318 (0–0·0227)	0·0764 (0·00215–0·388)
Afghanistan	15 to 95 plus	96·0 (90·2–98·8)	3·99 (1·21–9·84)	100 (100–100)
Algeria	15 to 39	65·9 (57·7–73·0)	4·47 (3·06–6·48)	70·3 (63·0–77·1)
Algeria	40 to 64	25·4 (19·6–31·6)	1·56 (0·754–2·65)	27·0 (20·7–33·7)
Algeria	65 plus	2·55 (1·73–3·62)	0·134 (0·0490–0·298)	2·68 (1·80–3·85)
Algeria	15 to 95 plus	93·8 (91·3–95·8)	6·17 (4·18–8·70)	100 (100–100)
Bahrain	15 to 39	68·1 (60·6–74·9)	2·23 (1·44–3·32)	70·3 (63·3–76·9)
Bahrain	40 to 64	27·9 (21·6–34·5)	0·741 (0·345–1·28)	28·6 (22·0–35·5)
Bahrain	65 plus	1·04 (0·693–1·55)	0·0372 (0·00815–0·0901)	1·07 (0·712–1·64)
Bahrain	15 to 95 plus	97·0 (95·6–98·0)	3·01 (1·97–4·40)	100 (100–100)
Egypt	15 to 39	69·6 (60·5–78·1)	5·16 (3·51–7·49)	74·7 (66·2–82·7)
Egypt	40 to 64	22·2 (15·4–29·4)	1·39 (0·627–2·45)	23·6 (16·2–31·6)
Egypt	65 plus	1·58 (0·872–2·58)	0·0750 (0·0213–0·187)	1·65 (0·900–2·75)

Egypt	15 to 95 plus	93·4 (90·6–95·4)	6·63 (4·55–9·39)	100 (100–100)
Iran (Islamic Republic of)	15 to 39	64·9 (57·1–73·2)	4·25 (2·80–6·14)	69·2 (61·9–76·8)
Iran (Islamic Republic of)	40 to 64	26·7 (20·2–32·8)	1·62 (0·656–2·86)	28·3 (21·2–34·9)
Iran (Islamic Republic of)	65 plus	2·36 (1·50–3·42)	0·138 (0·0382–0·326)	2·50 (1·55–3·71)
Iran (Islamic Republic of)	15 to 95 plus	94·0 (91·5–96·2)	6·01 (3·81–8·47)	100 (100–100)
Iraq	15 to 39	48·1 (40·9–55·0)	21·3 (16·3–27·2)	69·3 (64·0–74·8)
Iraq	40 to 64	17·7 (14·4–21·3)	9·97 (6·77–13·6)	27·7 (22·6–32·7)
Iraq	65 plus	1·86 (1·39–2·44)	1·11 (0·664–1·75)	2·97 (2·21–3·92)
Iraq	15 to 95 plus	67·6 (61·3–73·6)	32·4 (26·4–38·7)	100 (100–100)
Jordan	15 to 39	65·7 (58·0–74·4)	3·42 (2·31–4·80)	69·1 (61·6–77·4)
Jordan	40 to 64	26·6 (19·7–32·9)	1·43 (0·702–2·35)	28·0 (20·4–34·6)
Jordan	65 plus	2·80 (1·76–4·15)	0·0816 (0·0259–0·168)	2·88 (1·80–4·30)
Jordan	15 to 95 plus	95·1 (93·2–96·7)	4·93 (3·35–6·76)	100 (100–100)
Kuwait	15 to 39	82·3 (64·6–99·3)	2·37 (0·195–4·78)	84·7 (68·0–99·7)
Kuwait	40 to 64	14·7 (0·293–30·2)	0·389 (0–1·22)	15·0 (0·293–31·2)
Kuwait	65 plus	0·245 (0–1·02)	0·00492 (0–0·0301)	0·250 (0–1·04)
Kuwait	15 to 95 plus	97·2 (94·4–99·8)	2·76 (0·195–5·57)	100 (100–100)
Lebanon	15 to 39	66·6 (54·1–77·3)	10·1 (5·60–18·1)	76·6 (66·4–85·4)
Lebanon	40 to 64	18·2 (11·9–24·9)	1·78 (0·378–4·04)	20·0 (12·6–28·0)
Lebanon	65 plus	3·24 (1·75–5·60)	0·0858 (0·00724–0·384)	3·33 (1·76–5·90)
Lebanon	15 to 95 plus	88·1 (79·6–93·6)	11·9 (6·41–20·4)	100 (100–100)
Libya	15 to 39	69·4 (60·5–78·4)	2·24 (1·38–3·51)	71·6 (63·0–80·2)
Libya	40 to 64	26·2 (18·3–34·2)	0·463 (0·181–0·816)	26·7 (18·6–34·9)
Libya	65 plus	1·68 (0·901–2·66)	0·0271 (0·00421–0·0699)	1·71 (0·907–2·73)
Libya	15 to 95 plus	97·3 (95·9–98·3)	2·73 (1·68–4·11)	100 (100–100)
Morocco	15 to 39	80·3 (73·7–86·3)	0·595 (0·139–1·83)	80·9 (74·5–86·6)
Morocco	40 to 64	18·5 (13·0–24·7)	0·0495 (0·00206–0·187)	18·5 (13·0–24·8)
Morocco	65 plus	0·537 (0·302–0·889)	0·00137 (0–0·00905)	0·539 (0·302–0·897)
Morocco	15 to 95 plus	99·4 (98·1–99·8)	0·646 (0·152–1·89)	100 (100–100)
Oman	15 to 39	79·7 (69·9–89·1)	2·20 (1·13–3·90)	81·9 (72·7–90·6)
Oman	40 to 64	17·3 (9·19–25·7)	0·341 (0·0623–0·787)	17·7 (9·25–26·5)

Oman	65 plus	0·386 (0·142–0·861)	0·00871 (0–0·0381)	0·395 (0·143–0·899)
Oman	15 to 95 plus	97·4 (95·5–98·8)	2·55 (1·25–4·47)	100 (100–100)
Palestine	15 to 39	70·4 (64·2–76·8)	4·88 (3·16–6·93)	75·2 (69·6–81·0)
Palestine	40 to 64	21·6 (16·7–26·4)	1·39 (0·740–2·26)	23·0 (17·6–28·3)
Palestine	65 plus	1·67 (1·14–2·31)	0·118 (0·0445–0·232)	1·79 (1·21–2·51)
Palestine	15 to 95 plus	93·6 (91·2–95·7)	6·39 (4·29–8·76)	100 (100–100)
Qatar	15 to 39	82·3 (75·4–88·7)	1·06 (0·581–1·84)	83·3 (76·9–89·6)
Qatar	40 to 64	16·1 (10·1–22·3)	0·233 (0·0636–0·508)	16·3 (10·2–22·7)
Qatar	65 plus	0·314 (0·144–0·570)	0·00319 (0–0·0122)	0·317 (0·144–0·582)
Qatar	15 to 95 plus	98·7 (97·8–99·3)	1·30 (0·709–2·23)	100 (100–100)
Saudi Arabia	15 to 39	81·4 (68·2–96·8)	2·27 (0·571–4·47)	83·7 (71·4–97·6)
Saudi Arabia	40 to 64	15·7 (2·36–27·0)	0·225 (0·00353–0·636)	15·9 (2·36–27·5)
Saudi Arabia	65 plus	0·411 (0·00656–1·15)	0·00465 (0–0·0207)	0·416 (0·00656–1·17)
Saudi Arabia	15 to 95 plus	97·5 (95·2–99·4)	2·50 (0·603–4·80)	100 (100–100)
Sudan	15 to 39	94·4 (0–100)	0·826 (0–7·84)	95·2 (0–100)
Sudan	40 to 64	0·149 (0–0·828)	0·00442 (0–0·0173)	0·153 (0–0·855)
Sudan	65 plus	0·00411 (0–0)	0·000220 (0–0)	0·00433 (0–0)
Sudan	15 to 95 plus	94·6 (0–100)	0·831 (0–7·90)	95·4 (0–100)
Syrian Arab Republic	15 to 39	70·1 (59·1–81·1)	7·11 (4·18–11·2)	77·2 (67·5–86·7)
Syrian Arab Republic	40 to 64	20·0 (12·0–28·1)	1·37 (0·312–2·90)	21·4 (12·6–30·1)
Syrian Arab Republic	65 plus	1·39 (0·528–2·56)	0·0642 (0·00447–0·220)	1·45 (0·536–2·77)
Syrian Arab Republic	15 to 95 plus	91·5 (86·9–95·1)	8·54 (4·86–13·1)	100 (100–100)
Tunisia	15 to 39	66·7 (60·8–72·6)	0·600 (0·267–1·22)	67·3 (61·4–73·2)
Tunisia	40 to 64	29·1 (23·7–34·2)	0·123 (0·0217–0·336)	29·2 (23·8–34·4)
Tunisia	65 plus	3·48 (2·54–4·62)	0·00831 (0·000428–0·0403)	3·49 (2·55–4·64)
Tunisia	15 to 95 plus	99·3 (98·5–99·7)	0·732 (0·298–1·50)	100 (100–100)
Turkey	15 to 39	60·3 (50·9–69·4)	8·73 (6·16–12·3)	69·1 (60·2–77·7)
Turkey	40 to 64	24·8 (18·4–31·2)	3·10 (1·52–5·11)	27·9 (19·9–35·4)
Turkey	65 plus	2·78 (1·82–4·22)	0·232 (0·0851–0·542)	3·01 (1·94–4·72)
Turkey	15 to 95 plus	87·9 (83·5–91·7)	12·1 (8·29–16·5)	100 (100–100)
United Arab Emirates	15 to 39	49·2 (35·2–65·3)	3·33 (1·38–6·82)	52·5 (39·2–68·1)

United Arab Emirates	40 to 64	45·7 (31·0–57·9)	0·815 (0·0826–2·36)	46·5 (31·2–59·4)
United Arab Emirates	65 plus	0·962 (0·442–1·90)	0·00750 (0–0·0536)	0·969 (0·443–1·93)
United Arab Emirates	15 to 95 plus	95·9 (91·3–98·4)	4·15 (1·62–8·68)	100 (100–100)
Yemen	15 to 39	80·9 (71·9–88·5)	4·89 (2·78–8·10)	85·8 (78·4–92·4)
Yemen	40 to 64	12·9 (7·04–19·1)	0·671 (0·189–1·50)	13·6 (7·32–20·4)
Yemen	65 plus	0·612 (0·253–1·31)	0·0277 (0·00353–0·109)	0·640 (0·260–1·42)
Yemen	15 to 95 plus	94·4 (90·8–96·9)	5·59 (3·12–9·21)	100 (100–100)
South Asia	15 to 39	61·7 (55·6–69·9)	4·79 (3·06–6·51)	66·4 (60·5–74·6)
South Asia	40 to 64	27·5 (21·5–32·0)	2·22 (1·18–3·21)	29·7 (22·9–34·7)
South Asia	65 plus	3·62 (2·29–4·98)	0·240 (0·101–0·432)	3·86 (2·40–5·33)
South Asia	15 to 95 plus	92·8 (90·6–95·2)	7·25 (4·83–9·35)	100 (100–100)
Bangladesh	15 to 39	74·0 (63·0–92·8)	7·09 (0·953–12·1)	81·1 (70·4–99·0)
Bangladesh	40 to 64	16·3 (1·00–24·9)	1·15 (0·00318–2·30)	17·4 (1·00–27·0)
Bangladesh	65 plus	1·38 (0·00236–2·80)	0·0667 (0–0·179)	1·44 (0·00236–2·93)
Bangladesh	15 to 95 plus	91·7 (87·0–98·7)	8·30 (1·26–13·0)	100 (100–100)
Bhutan	15 to 39	63·3 (50·3–83·3)	24·3 (8·44–34·6)	87·6 (77·1–98·2)
Bhutan	40 to 64	8·95 (1·42–15·6)	2·96 (0·229–6·17)	11·9 (1·78–21·2)
Bhutan	65 plus	0·384 (0·0111–1·23)	0·111 (0–0·465)	0·496 (0·0153–1·71)
Bhutan	15 to 95 plus	72·6 (62·8–89·7)	27·4 (10·3–37·2)	100 (100–100)
India	15 to 39	60·4 (54·3–68·5)	4·04 (2·65–5·65)	64·4 (58·5–72·5)
India	40 to 64	29·1 (23·2–33·9)	2·20 (1·20–3·20)	31·3 (24·5–36·3)
India	65 plus	3·98 (2·55–5·44)	0·258 (0·108–0·470)	4·23 (2·67–5·85)
India	15 to 95 plus	93·5 (91·5–95·5)	6·50 (4·47–8·49)	100 (100–100)
Nepal	15 to 39	57·7 (48·3–73·8)	16·3 (9·57–21·3)	74·0 (65·4–86·8)
Nepal	40 to 64	18·4 (10·3–23·7)	5·45 (1·90–8·31)	23·8 (12·4–31·2)
Nepal	65 plus	1·88 (0·506–3·28)	0·333 (0·0399–0·736)	2·21 (0·541–3·88)
Nepal	15 to 95 plus	78·0 (72·6–86·7)	22·0 (13·3–27·4)	100 (100–100)
Pakistan	15 to 39	75·1 (64·7–88·1)	9·16 (4·14–13·9)	84·2 (74·6–95·3)
Pakistan	40 to 64	13·5 (4·14–21·2)	1·68 (0·336–3·21)	15·2 (4·63–24·3)
Pakistan	65 plus	0·525 (0·0794–1·31)	0·0492 (0·00465–0·147)	0·574 (0·0849–1·43)
Pakistan	15 to 95 plus	89·1 (84·2–94·6)	10·9 (5·42–15·8)	100 (100–100)

East Asia	15 to 39	42·8 (38·8–46·8)	4·26 (2·94–6·35)	47·1 (42·7–51·3)
East Asia	40 to 64	41·1 (37·9–44·7)	3·60 (2·63–4·88)	44·7 (41·4–48·6)
East Asia	65 plus	7·60 (6·13–9·08)	0·584 (0·292–0·997)	8·19 (6·47–9·85)
East Asia	15 to 95 plus	91·6 (88·9–93·5)	8·45 (6·51–11·1)	100 (100–100)
China	15 to 39	42·7 (38·6–46·6)	4·23 (2·90–6·32)	46·9 (42·4–51·1)
China	40 to 64	41·3 (38·0–44·8)	3·60 (2·62–4·89)	44·8 (41·5–48·7)
China	65 plus	7·67 (6·18–9·16)	0·590 (0·294–1·01)	8·27 (6·54–9·95)
China	15 to 95 plus	91·6 (89·0–93·6)	8·41 (6·44–11·0)	100 (100–100)
Democratic People's Republic of Korea	15 to 39	52·5 (46·9–58·0)	5·07 (3·11–8·00)	57·6 (51·2–63·3)
Democratic People's Republic of Korea	40 to 64	35·3 (31·0–40·2)	3·05 (1·95–4·37)	38·3 (33·3–43·7)
Democratic People's Republic of Korea	65 plus	3·73 (2·64–4·94)	0·324 (0·116–0·653)	4·05 (2·78–5·41)
Democratic People's Republic of Korea	15 to 95 plus	91·6 (87·8–94·1)	8·44 (5·90–12·2)	100 (100–100)
Taiwan (Province of China)	15 to 39	45·5 (40·5–50·3)	5·98 (3·92–9·11)	51·4 (45·5–56·5)
Taiwan (Province of China)	40 to 64	37·2 (33·6–41·6)	4·51 (3·06–6·30)	41·7 (37·8–46·4)
Taiwan (Province of China)	65 plus	6·43 (4·74–8·19)	0·446 (0·172–0·831)	6·87 (4·93–8·90)
Taiwan (Province of China)	15 to 95 plus	89·1 (85·3–91·9)	10·9 (8·11–14·7)	100 (100–100)
Oceania	15 to 39	74·7 (66·2–87·3)	12·0 (2·68–17·6)	86·7 (80·0–94·0)
Oceania	40 to 64	11·3 (5·25–16·6)	1·07 (0·278–2·00)	12·4 (5·60–18·4)
Oceania	65 plus	0·856 (0·362–1·53)	0·0372 (0·00904–0·0937)	0·893 (0·370–1·60)
Oceania	15 to 95 plus	86·9 (81·2–96·7)	13·1 (3·33–18·8)	100 (100–100)
American Samoa	15 to 39	79·8 (62·6–99·5)	16·8 (0·144–32·2)	96·6 (87·2–100)
American Samoa	40 to 64	3·09 (0–11·5)	0·212 (0–1·05)	3·30 (0–12·2)
American Samoa	65 plus	0·147 (0–0·870)	0·00246 (0–0·0324)	0·150 (0–0·894)
American Samoa	15 to 95 plus	83·0 (67·5–99·9)	17·0 (0·145–32·5)	100 (100–100)
Cook Islands	15 to 39	37·9 (32·9–46·0)	21·8 (13·2–26·5)	59·7 (53·4–67·2)
Cook Islands	40 to 64	25·3 (21·7–28·7)	8·00 (5·17–10·7)	33·3 (27·4–38·0)
Cook Islands	65 plus	6·04 (4·60–7·46)	0·969 (0·466–1·62)	7·01 (5·26–8·83)
Cook Islands	15 to 95 plus	69·2 (64·4–78·8)	30·8 (21·2–35·6)	100 (100–100)
Fiji	15 to 39	70·9 (61·8–83·4)	10·7 (3·42–15·4)	81·6 (73·8–90·5)
Fiji	40 to 64	15·6 (8·39–21·9)	1·42 (0·398–2·67)	17·0 (8·74–24·1)
Fiji	65 plus	1·36 (0·589–2·40)	0·0526 (0·00703–0·142)	1·42 (0·601–2·54)

Fiji	15 to 95 plus	87·8 (82·8–95·9)	12·2 (4·11–17·2)	100 (100–100)
Guam	15 to 39	62·4 (47·0–87·4)	18·4 (2·55–28·2)	80·8 (66·8–97·1)
Guam	40 to 64	15·5 (2·65–24·6)	1·65 (0·0125–4·75)	17·1 (2·72–28·6)
Guam	65 plus	2·03 (0·157–4·12)	0·0671 (0–0·315)	2·09 (0·157–4·43)
Guam	15 to 95 plus	79·9 (70·3–96·8)	20·1 (3·23–29·7)	100 (100–100)
Kiribati	15 to 39	79·6 (66·6–96·7)	14·1 (1·68–23·0)	93·7 (83·5–99·9)
Kiribati	40 to 64	5·21 (0·0591–13·4)	0·819 (0–2·56)	6·03 (0·0721–15·5)
Kiribati	65 plus	0·216 (0–0·788)	0·0268 (0–0·153)	0·243 (0–0·881)
Kiribati	15 to 95 plus	85·1 (76·1–98·1)	14·9 (1·93–23·9)	100 (100–100)
Marshall Islands	15 to 39	75·8 (67·2–87·9)	8·83 (1·83–13·4)	84·7 (77·2–93·2)
Marshall Islands	40 to 64	13·4 (6·15–19·4)	0·828 (0·144–1·76)	14·2 (6·32–21·0)
Marshall Islands	65 plus	1·08 (0·357–2·00)	0·0270 (0–0·102)	1·11 (0·363–2·05)
Marshall Islands	15 to 95 plus	90·3 (85·8–97·7)	9·69 (2·32–14·2)	100 (100–100)
Micronesia (Federated States of)	15 to 39	70·8 (59·0–89·9)	16·7 (2·00–26·1)	87·5 (77·7–96·4)
Micronesia (Federated States of)	40 to 64	10·8 (3·27–18·7)	0·984 (0·0954–2·32)	11·8 (3·42–20·7)
Micronesia (Federated States of)	65 plus	0·715 (0·165–1·52)	0·0222 (0–0·101)	0·737 (0·165–1·60)
Micronesia (Federated States of)	15 to 95 plus	82·3 (72·7–97·7)	17·7 (2·28–27·3)	100 (100–100)
Nauru	15 to 39	64·8 (55·9–81·8)	19·5 (4·78–27·5)	84·3 (77·9–91·3)
Nauru	40 to 64	12·6 (7·34–17·1)	1·92 (0·402–3·72)	14·5 (8·03–20·2)
Nauru	65 plus	1·12 (0·424–1·91)	0·0809 (0–0·304)	1·20 (0·452–2·11)
Nauru	15 to 95 plus	78·5 (70·5–94·5)	21·5 (5·51–29·5)	100 (100–100)
Niue	15 to 39	49·1 (37·9–69·2)	20·9 (5·88–29·7)	70·1 (59·1–87·1)
Niue	40 to 64	22·4 (10·4–30·3)	4·29 (0–8·50)	26·7 (11·4–36·4)
Niue	65 plus	3·00 (0·543–5·39)	0·229 (0–1·05)	3·23 (0·543–5·84)
Niue	15 to 95 plus	74·5 (66·1–91·1)	25·5 (8·90–33·9)	100 (100–100)
Northern Mariana Islands	15 to 39	60·0 (43·1–90·0)	14·4 (1·83–24·7)	74·4 (56·8–98·4)
Northern Mariana Islands	40 to 64	21·3 (1·55–34·2)	2·11 (0–5·44)	23·4 (1·55–39·0)
Northern Mariana Islands	65 plus	2·16 (0·0459–4·34)	0·0896 (0–0·377)	2·25 (0·0459–4·65)
Northern Mariana Islands	15 to 95 plus	83·4 (74·1–97·9)	16·6 (2·08–25·9)	100 (100–100)
Palau	15 to 39	60·8 (44·4–85·8)	14·9 (2·97–23·4)	75·6 (58·6–95·7)
Palau	40 to 64	19·0 (3·69–30·1)	3·49 (0·140–8·19)	22·5 (4·10–37·4)

Palau	65 plus	1·68 (0·130–3·63)	0·163 (0–0·614)	1·85 (0·130–4·16)
Palau	15 to 95 plus	81·5 (73·5–95·3)	18·5 (4·68–26·5)	100 (100–100)
Papua New Guinea	15 to 39	76·6 (68·1–88·9)	11·2 (2·53–16·9)	87·8 (81·2–94·8)
Papua New Guinea	40 to 64	10·6 (4·61–16·1)	0·970 (0·210–1·93)	11·5 (4·91–17·5)
Papua New Guinea	65 plus	0·690 (0·247–1·29)	0·0267 (0·00298–0·0799)	0·717 (0·249–1·34)
Papua New Guinea	15 to 95 plus	87·8 (81·9–96·8)	12·2 (3·21–18·1)	100 (100–100)
Samoa	15 to 39	72·6 (62·2–87·2)	9·91 (2·18–15·3)	82·5 (73·0–92·8)
Samoa	40 to 64	15·5 (6·49–23·6)	0·586 (0·0832–1·24)	16·1 (6·65–24·7)
Samoa	65 plus	1·39 (0·426–2·64)	0·0158 (0–0·0560)	1·41 (0·430–2·68)
Samoa	15 to 95 plus	89·5 (84·2–97·4)	10·5 (2·59–15·8)	100 (100–100)
Solomon Islands	15 to 39	78·7 (66·2–97·2)	15·1 (0·710–25·8)	93·8 (86·4–99·3)
Solomon Islands	40 to 64	5·63 (0·670–12·0)	0·350 (0·00998–1·03)	5·98 (0·695–13·0)
Solomon Islands	65 plus	0·227 (0·0143–0·718)	0·00364 (0–0·0198)	0·231 (0·0143–0·742)
Solomon Islands	15 to 95 plus	84·5 (73·4–99·3)	15·5 (0·736–26·6)	100 (100–100)
Tokelau	15 to 39	63·8 (51·3–81·6)	15·6 (3·24–23·9)	79·4 (67·6–92·8)
Tokelau	40 to 64	16·0 (5·62–24·3)	1·70 (0–4·66)	17·7 (6·01–27·7)
Tokelau	65 plus	2·78 (0–6·10)	0·126 (0–0·954)	2·91 (0–6·36)
Tokelau	15 to 95 plus	82·5 (73·9–95·3)	17·5 (4·70–26·1)	100 (100–100)
Tonga	15 to 39	71·7 (59·3–90·0)	16·1 (3·85–23·7)	87·8 (76·9–97·8)
Tonga	40 to 64	9·27 (1·71–17·0)	1·76 (0·207–3·92)	11·0 (2·00–20·4)
Tonga	65 plus	0·999 (0·101–2·39)	0·133 (0–0·433)	1·13 (0·101–2·80)
Tonga	15 to 95 plus	82·0 (74·1–94·8)	18·0 (5·18–25·9)	100 (100–100)
Tuvalu	15 to 39	73·9 (61·1–92·3)	14·8 (1·42–24·5)	88·7 (78·8–97·2)
Tuvalu	40 to 64	9·51 (2·47–17·2)	0·790 (0–2·12)	10·3 (2·59–18·8)
Tuvalu	65 plus	0·928 (0·0801–2·25)	0·0322 (0–0·212)	0·961 (0·0846–2·40)
Tuvalu	15 to 95 plus	84·4 (74·6–98·2)	15·6 (1·85–25·4)	100 (100–100)
Vanuatu	15 to 39	71·0 (61·6–85·1)	15·6 (3·61–23·5)	86·6 (79·9–93·8)
Vanuatu	40 to 64	11·4 (5·48–17·0)	1·01 (0·194–2·03)	12·5 (5·72–18·4)
Vanuatu	65 plus	0·939 (0·370–1·71)	0·0370 (0–0·114)	0·976 (0·379–1·80)
Vanuatu	15 to 95 plus	83·4 (75·4–96·0)	16·6 (4·05–24·6)	100 (100–100)
Southeast Asia	15 to 39	57·1 (52·9–63·3)	8·59 (3·89–11·7)	65·7 (61·8–69·8)

Southeast Asia	40 to 64	27·2 (24·7–30·1)	3·15 (2·19–4·03)	30·3 (27·1–33·6)
Southeast Asia	65 plus	3·58 (2·70–4·25)	0·373 (0·216–0·520)	3·96 (2·96–4·73)
Southeast Asia	15 to 95 plus	87·9 (84·2–93·2)	12·1 (6·82–15·8)	100 (100–100)
Cambodia	15 to 39	50·3 (45·0–58·9)	20·3 (11·8–25·9)	70·6 (67·3–73·9)
Cambodia	40 to 64	18·8 (16·7–21·4)	7·59 (5·48–9·50)	26·4 (23·4–29·3)
Cambodia	65 plus	2·21 (1·72–2·71)	0·800 (0·475–1·20)	3·01 (2·31–3·68)
Cambodia	15 to 95 plus	71·3 (65·2–81·6)	28·7 (18·4–34·8)	100 (100–100)
Indonesia	15 to 39	71·3 (61·7–84·1)	5·77 (2·06–8·98)	77·1 (68·7–88·4)
Indonesia	40 to 64	19·3 (10·3–26·2)	2·16 (0·735–3·72)	21·4 (11·3–29·1)
Indonesia	65 plus	1·29 (0·331–2·35)	0·189 (0·0309–0·395)	1·48 (0·362–2·69)
Indonesia	15 to 95 plus	91·9 (87·8–96·6)	8·12 (3·38–12·2)	100 (100–100)
Lao People's Democratic Republic	15 to 39	52·8 (47·9–61·8)	22·4 (13·3–26·7)	75·3 (71·2–79·3)
Lao People's Democratic Republic	40 to 64	17·2 (14·9–19·8)	5·30 (3·85–6·80)	22·5 (19·1–26·0)
Lao People's Democratic Republic	65 plus	1·84 (1·17–2·39)	0·349 (0·168–0·574)	2·19 (1·36–2·89)
Lao People's Democratic Republic	15 to 95 plus	71·9 (67·4–81·9)	28·1 (18·1–32·6)	100 (100–100)
Malaysia	15 to 39	62·4 (55·3–72·7)	12·1 (5·63–16·4)	74·5 (68·6–81·7)
Malaysia	40 to 64	18·8 (14·2–22·9)	4·04 (2·25–5·84)	22·9 (16·7–28·1)
Malaysia	65 plus	2·13 (1·10–3·07)	0·499 (0·212–0·900)	2·63 (1·35–3·82)
Malaysia	15 to 95 plus	83·4 (78·4–90·7)	16·6 (9·33–21·6)	100 (100–100)
Maldives	15 to 39	81·6 (71·9–92·2)	3·22 (0·307–6·52)	84·8 (76·3–93·9)
Maldives	40 to 64	14·4 (5·97–22·3)	0·361 (0·0235–0·979)	14·8 (5·99–22·9)
Maldives	65 plus	0·444 (0·0530–1·01)	0·0119 (0–0·0486)	0·456 (0·0530–1·04)
Maldives	15 to 95 plus	96·4 (93·1–99·6)	3·59 (0·398–6·95)	100 (100–100)
Mauritius	15 to 39	53·9 (44·7–66·4)	8·12 (1·04–15·7)	62·0 (53·9–71·3)
Mauritius	40 to 64	30·5 (24·8–36·4)	2·11 (0·500–4·07)	32·6 (25·5–38·8)
Mauritius	65 plus	5·22 (2·78–7·18)	0·205 (0·0222–0·576)	5·43 (2·83–7·63)
Mauritius	15 to 95 plus	89·6 (80·9–97·4)	10·4 (2·56–19·1)	100 (100–100)
Myanmar	15 to 39	64·9 (60·0–70·6)	2·76 (0·737–4·71)	67·7 (63·2–72·2)
Myanmar	40 to 64	28·6 (24·9–32·6)	0·636 (0·206–1·21)	29·2 (25·3–33·2)
Myanmar	65 plus	3·07 (2·06–4·01)	0·0288 (0·00613–0·0648)	3·10 (2·07–4·06)
Myanmar	15 to 95 plus	96·6 (94·2–99·0)	3·42 (1·05–5·83)	100 (100–100)

Philippines	15 to 39	56·6 (51·8–64·3)	13·6 (7·16–17·7)	70·2 (66·0–74·4)
Philippines	40 to 64	22·1 (19·9–25·0)	4·69 (3·31–6·08)	26·8 (23·6–30·4)
Philippines	65 plus	2·37 (1·65–2·93)	0·581 (0·315–0·856)	2·95 (2·05–3·69)
Philippines	15 to 95 plus	81·1 (76·5–88·2)	18·9 (11·8–23·5)	100 (100–100)
Seychelles	15 to 39	55·9 (51·0–62·6)	5·01 (1·89–7·41)	60·9 (56·1–66·7)
Seychelles	40 to 64	32·9 (28·9–36·9)	2·47 (1·27–3·75)	35·4 (30·5–39·6)
Seychelles	65 plus	3·47 (2·43–4·47)	0·254 (0·0888–0·480)	3·73 (2·56–4·83)
Seychelles	15 to 95 plus	92·3 (89·2–96·5)	7·73 (3·55–10·8)	100 (100–100)
Sri Lanka	15 to 39	50·1 (45·8–54·2)	2·10 (1·02–3·04)	52·2 (48·0–56·3)
Sri Lanka	40 to 64	38·4 (34·9–42·1)	1·49 (1·04–1·99)	39·9 (36·2–43·7)
Sri Lanka	65 plus	7·58 (6·07–9·22)	0·348 (0·224–0·484)	7·92 (6·34–9·61)
Sri Lanka	15 to 95 plus	96·1 (94·9–97·4)	3·94 (2·64–5·10)	100 (100–100)
Thailand	15 to 39	46·4 (42·5–51·3)	5·56 (2·61–7·65)	52·0 (48·4–56·2)
Thailand	40 to 64	37·2 (34·2–40·5)	3·98 (2·71–5·37)	41·2 (37·8–44·6)
Thailand	65 plus	6·23 (5·05–7·47)	0·588 (0·331–0·886)	6·82 (5·46–8·15)
Thailand	15 to 95 plus	89·9 (86·9–93·6)	10·1 (6·42–13·1)	100 (100–100)
Timor-Leste	15 to 39	63·6 (57·9–70·7)	4·55 (1·75–7·11)	68·2 (62·8–73·7)
Timor-Leste	40 to 64	25·5 (21·4–29·6)	1·67 (0·940–2·51)	27·2 (22·7–31·6)
Timor-Leste	65 plus	4·30 (2·92–5·60)	0·306 (0·126–0·522)	4·61 (3·08–6·02)
Timor-Leste	15 to 95 plus	93·5 (90·4–96·8)	6·53 (3·24–9·57)	100 (100–100)
Viet Nam	15 to 39	58·8 (54·2–65·5)	4·13 (0·519–8·06)	63·0 (59·1–67·3)
Viet Nam	40 to 64	31·3 (28·5–34·2)	1·17 (0·374–2·15)	32·5 (29·0–35·6)
Viet Nam	65 plus	4·47 (3·24–5·34)	0·103 (0·0228–0·223)	4·57 (3·28–5·49)
Viet Nam	15 to 95 plus	94·6 (89·8–99·0)	5·40 (1·03–10·2)	100 (100–100)
Central Sub-Saharan Africa	15 to 39	54·4 (45·8–82·4)	22·8 (1·29–28·8)	77·2 (71·1–86·0)
Central Sub-Saharan Africa	40 to 64	15·6 (10·5–19·4)	5·54 (1·09–8·13)	21·1 (13·0–26·7)
Central Sub-Saharan Africa	65 plus	1·24 (0·614–1·77)	0·465 (0·112–0·831)	1·71 (0·738–2·53)
Central Sub-Saharan Africa	15 to 95 plus	71·2 (64·4–97·2)	28·8 (2·80–35·6)	100 (100–100)
Angola	15 to 39	51·6 (45·3–71·1)	20·2 (3·26–25·3)	71·7 (67·6–77·6)
Angola	40 to 64	19·3 (16·6–22·6)	6·79 (2·62–9·11)	26·1 (20·9–29·9)
Angola	65 plus	1·67 (1·21–2·12)	0·458 (0·184–0·736)	2·13 (1·50–2·72)

Angola	15 to 95 plus	72·6 (66·8–93·8)	27·4 (6·22–33·2)	100 (100–100)
Central African Republic	15 to 39	56·5 (45·3–91·4)	24·2 (0·207–31·8)	80·7 (73·3–93·1)
Central African Republic	40 to 64	13·7 (5·57–18·6)	4·62 (0·278–7·79)	18·3 (6·47–25·3)
Central African Republic	65 plus	0·724 (0·191–1·20)	0·310 (0·0208–0·655)	1·03 (0·250–1·81)
Central African Republic	15 to 95 plus	70·8 (62·3–99·2)	29·2 (0·781–37·7)	100 (100–100)
Congo	15 to 39	43·7 (35·9–75·9)	30·4 (2·73–36·8)	74·0 (68·3–83·4)
Congo	40 to 64	16·2 (12·3–19·9)	7·53 (1·71–10·6)	23·8 (15·5–29·1)
Congo	65 plus	1·55 (0·848–2·09)	0·663 (0·169–1·14)	2·21 (1·07–3·11)
Congo	15 to 95 plus	61·4 (54·4–94·6)	38·6 (5·42–45·6)	100 (100–100)
Democratic Republic of the Congo	15 to 39	56·7 (46·3–89·4)	22·8 (0·200–30·1)	79·5 (71·9–91·2)
Democratic Republic of the Congo	40 to 64	14·2 (7·37–19·1)	4·87 (0·309–7·91)	19·1 (8·03–26·0)
Democratic Republic of the Congo	65 plus	1·04 (0·273–1·67)	0·436 (0·0445–0·896)	1·48 (0·366–2·49)
Democratic Republic of the Congo	15 to 95 plus	71·9 (63·9–99·3)	28·1 (0·715–36·1)	100 (100–100)
Equatorial Guinea	15 to 39	59·5 (52·6–81·2)	20·1 (2·27–25·8)	79·7 (75·7–85·5)
Equatorial Guinea	40 to 64	12·7 (10·3–15·1)	5·60 (1·65–7·91)	18·3 (13·2–21·9)
Equatorial Guinea	65 plus	1·36 (0·854–1·75)	0·620 (0·220–0·997)	1·98 (1·15–2·67)
Equatorial Guinea	15 to 95 plus	73·6 (67·1–95·4)	26·4 (4·58–32·9)	100 (100–100)
Gabon	15 to 39	45·6 (39·4–66·7)	21·7 (3·17–27·4)	67·3 (62·8–74·0)
Gabon	40 to 64	20·8 (17·9–24·5)	7·83 (2·98–10·7)	28·6 (23·2–32·5)
Gabon	65 plus	3·00 (2·19–3·64)	1·11 (0·470–1·70)	4·12 (2·75–5·16)
Gabon	15 to 95 plus	69·4 (63·3–92·8)	30·6 (7·21–36·7)	100 (100–100)
Eastern Sub-Saharan Africa	15 to 39	54·3 (46·6–67·9)	21·3 (7·16–27·2)	75·6 (70·9–80·0)
Eastern Sub-Saharan Africa	40 to 64	16·2 (13·7–19·2)	5·57 (3·82–7·17)	21·7 (17·8–25·8)
Eastern Sub-Saharan Africa	65 plus	2·01 (1·49–2·55)	0·640 (0·411–0·875)	2·65 (1·94–3·34)
Eastern Sub-Saharan Africa	15 to 95 plus	72·4 (65·5–86·5)	27·6 (13·5–34·5)	100 (100–100)
Burundi	15 to 39	57·3 (51·6–66·7)	15·3 (5·43–20·3)	72·5 (68·4–76·9)
Burundi	40 to 64	20·2 (17·7–23·3)	4·26 (2·77–5·89)	24·5 (20·6–28·2)
Burundi	65 plus	2·60 (1·98–3·25)	0·403 (0·204–0·604)	3·00 (2·24–3·73)
Burundi	15 to 95 plus	80·1 (74·5–90·7)	19·9 (9·29–25·5)	100 (100–100)
Comoros	15 to 39	60·9 (52·0–74·7)	19·2 (9·03–27·3)	80·1 (73·9–88·1)
Comoros	40 to 64	14·5 (9·07–19·2)	2·77 (1·31–4·39)	17·3 (10·5–22·7)

Comoros	65 plus	2·05 (0·949–3·07)	0·535 (0·181–0·891)	2·59 (1·12–3·80)
Comoros	15 to 95 plus	77·5 (69·7–88·4)	22·5 (11·6–30·3)	100 (100–100)
Djibouti	15 to 39	75·8 (52·6–98·3)	13·3 (0·0900–25·0)	89·0 (74·3–99·4)
Djibouti	40 to 64	8·96 (0·488–20·1)	1·75 (0·0635–4·65)	10·7 (0·554–24·6)
Djibouti	65 plus	0·221 (0·00897–0·642)	0·0399 (0–0·139)	0·261 (0·0109–0·747)
Djibouti	15 to 95 plus	84·9 (72·2–99·5)	15·1 (0·473–27·8)	100 (100–100)
Eritrea	15 to 39	65·1 (45·3–87·1)	18·6 (0·491–33·3)	83·8 (75·6–93·1)
Eritrea	40 to 64	13·6 (6·41–18·9)	1·76 (0·0295–6·10)	15·4 (6·50–23·4)
Eritrea	65 plus	0·791 (0·250–1·31)	0·0515 (0·000351–0·196)	0·843 (0·252–1·49)
Eritrea	15 to 95 plus	79·6 (61·7–99·3)	20·4 (0·731–38·3)	100 (100–100)
Ethiopia	15 to 39	52·0 (42·8–72·4)	27·0 (7·03–34·8)	79·0 (73·8–85·3)
Ethiopia	40 to 64	13·0 (9·88–15·9)	5·70 (2·87–8·15)	18·7 (13·1–23·3)
Ethiopia	65 plus	1·75 (1·11–2·36)	0·582 (0·229–0·955)	2·33 (1·34–3·18)
Ethiopia	15 to 95 plus	66·7 (57·7–88·2)	33·3 (11·8–42·3)	100 (100–100)
Kenya	15 to 39	59·2 (52·8–68·3)	10·0 (3·34–13·7)	69·2 (64·1–75·2)
Kenya	40 to 64	23·2 (19·0–27·0)	3·79 (2·50–5·08)	27·0 (21·9–31·8)
Kenya	65 plus	3·02 (2·20–3·77)	0·693 (0·393–0·980)	3·72 (2·68–4·68)
Kenya	15 to 95 plus	85·5 (81·3–92·2)	14·5 (7·78–18·7)	100 (100–100)
Madagascar	15 to 39	61·3 (50·4–79·4)	16·8 (2·59–24·3)	78·0 (70·8–87·0)
Madagascar	40 to 64	16·5 (10·5–21·0)	4·02 (1·47–6·61)	20·5 (12·3–27·4)
Madagascar	65 plus	1·20 (0·605–1·76)	0·263 (0·0708–0·485)	1·46 (0·700–2·18)
Madagascar	15 to 95 plus	79·0 (70·7–94·1)	21·0 (5·89–29·3)	100 (100–100)
Malawi	15 to 39	66·5 (61·3–72·9)	6·94 (2·75–9·60)	73·4 (69·0–77·7)
Malawi	40 to 64	21·2 (18·0–24·7)	2·74 (1·71–3·82)	23·9 (19·9–27·9)
Malawi	65 plus	2·21 (1·57–2·83)	0·486 (0·254–0·756)	2·69 (1·88–3·46)
Malawi	15 to 95 plus	89·8 (86·7–94·8)	10·2 (5·24–13·3)	100 (100–100)
Mozambique	15 to 39	59·2 (45·3–85·7)	23·6 (1·84–33·9)	82·8 (75·8–91·1)
Mozambique	40 to 64	12·1 (6·85–16·6)	3·89 (1·09–6·70)	16·0 (8·31–22·8)
Mozambique	65 plus	1·01 (0·433–1·60)	0·219 (0·0440–0·452)	1·23 (0·495–1·98)
Mozambique	15 to 95 plus	72·3 (60·6–96·0)	27·7 (3·97–39·4)	100 (100–100)
Rwanda	15 to 39	50·7 (44·1–63·4)	24·0 (10·1–30·3)	74·7 (70·7–78·9)

Rwanda	40 to 64	15·6 (13·5–18·5)	7·22 (4·83–9·70)	22·8 (19·1–26·5)
Rwanda	65 plus	1·80 (1·40–2·24)	0·691 (0·389–1·03)	2·49 (1·84–3·10)
Rwanda	15 to 95 plus	68·1 (61·1–83·4)	31·9 (16·6–38·9)	100 (100–100)
Somalia	15 to 39	75·1 (0–100)	11·7 (0–39·9)	86·8 (0–100)
Somalia	40 to 64	0·640 (0–11·2)	0·113 (0–0·0000663)	0·753 (0–13·2)
Somalia	65 plus	0·00105 (0–0)	0·000693 (0–0)	0·00174 (0–0)
Somalia	15 to 95 plus	75·7 (0–100)	11·9 (0–40·7)	87·6 (0–100)
South Sudan	15 to 39	76·3 (54·7–99·0)	15·7 (0·0394–29·3)	92·0 (80·3–99·6)
South Sudan	40 to 64	6·24 (0·314–15·7)	1·51 (0·0358–4·35)	7·75 (0·397–19·3)
South Sudan	65 plus	0·153 (0·00295–0·514)	0·0541 (0·000186–0·142)	0·207 (0·00361–0·638)
South Sudan	15 to 95 plus	82·7 (68·2–99·8)	17·3 (0·203–31·8)	100 (100–100)
Uganda	15 to 39	51·5 (46·1–60·1)	23·6 (13·0–29·3)	75·1 (71·5–78·2)
Uganda	40 to 64	15·9 (14·0–18·6)	6·60 (4·92–8·31)	22·5 (19·7–25·8)
Uganda	65 plus	1·70 (1·37–2·06)	0·704 (0·463–0·990)	2·41 (1·93–2·90)
Uganda	15 to 95 plus	69·1 (63·2–79·8)	30·9 (20·2–36·8)	100 (100–100)
United Republic of Tanzania	15 to 39	41·8 (37·3–47·6)	19·6 (12·9–24·1)	61·5 (57·0–65·2)
United Republic of Tanzania	40 to 64	22·0 (19·3–25·0)	10·9 (8·96–13·1)	32·9 (29·6–36·6)
United Republic of Tanzania	65 plus	3·94 (3·27–4·72)	1·69 (1·26–2·16)	5·63 (4·63–6·65)
United Republic of Tanzania	15 to 95 plus	67·8 (63·1–74·5)	32·2 (25·5–36·9)	100 (100–100)
Zambia	15 to 39	56·1 (49·6–67·9)	18·4 (6·56–24·2)	74·5 (70·7–78·4)
Zambia	40 to 64	18·3 (15·9–21·2)	5·04 (3·08–6·87)	23·3 (19·9–26·8)
Zambia	65 plus	1·69 (1·27–2·13)	0·511 (0·268–0·770)	2·20 (1·59–2·79)
Zambia	15 to 95 plus	76·0 (69·4–89·3)	24·0 (10·7–30·6)	100 (100–100)
Southern Sub-Saharan Africa	15 to 39	50·9 (46·3–66·2)	16·8 (4·28–20·1)	67·7 (64·2–71·9)
Southern Sub-Saharan Africa	40 to 64	21·6 (18·9–23·6)	6·93 (5·51–8·49)	28·5 (24·9–31·6)
Southern Sub-Saharan Africa	65 plus	2·78 (2·17–3·28)	1·06 (0·778–1·39)	3·84 (3·00–4·58)
Southern Sub-Saharan Africa	15 to 95 plus	75·2 (71·7–88·5)	24·8 (11·5–28·3)	100 (100–100)
Botswana	15 to 39	52·9 (46·9–72·7)	20·0 (3·49–25·0)	72·9 (69·0–77·9)
Botswana	40 to 64	19·4 (16·2–22·4)	5·73 (3·82–7·53)	25·2 (20·4–28·9)
Botswana	65 plus	1·49 (0·985–1·96)	0·425 (0·251–0·682)	1·91 (1·27–2·51)
Botswana	15 to 95 plus	73·8 (68·5–91·4)	26·2 (8·59–31·5)	100 (100–100)

Eswatini	15 to 39	56·5 (51·3–70·5)	14·8 (3·29–18·8)	71·3 (68·0–75·2)
Eswatini	40 to 64	19·9 (17·2–22·5)	5·40 (3·87–6·91)	25·3 (21·8–28·4)
Eswatini	65 plus	2·25 (1·76–2·71)	1·12 (0·723–1·54)	3·37 (2·61–4·17)
Eswatini	15 to 95 plus	78·7 (74·3–91·2)	21·3 (8·80–25·7)	100 (100–100)
Lesotho	15 to 39	54·6 (47·2–81·6)	21·6 (1·40–27·1)	76·2 (71·2–84·1)
Lesotho	40 to 64	16·1 (11·2–18·9)	5·53 (3·02–7·77)	21·6 (14·5–26·1)
Lesotho	65 plus	1·64 (0·858–2·29)	0·549 (0·221–0·952)	2·18 (1·09–3·19)
Lesotho	15 to 95 plus	72·4 (66·3–94·5)	27·6 (5·52–33·7)	100 (100–100)
Namibia	15 to 39	46·8 (41·3–66·1)	24·1 (6·72–29·1)	70·9 (66·9–75·8)
Namibia	40 to 64	16·1 (13·6–18·1)	9·34 (7·18–11·6)	25·4 (21·3–28·9)
Namibia	65 plus	2·13 (1·50–2·72)	1·58 (0·971–2·33)	3·71 (2·55–4·84)
Namibia	15 to 95 plus	65·0 (60·1–82·6)	35·0 (17·4–39·9)	100 (100–100)
South Africa	15 to 39	48·4 (43·7–62·9)	17·6 (4·75–21·2)	65·9 (62·4–69·7)
South Africa	40 to 64	22·2 (19·9–24·5)	7·68 (6·07–9·51)	29·9 (26·8–33·0)
South Africa	65 plus	3·03 (2·40–3·59)	1·19 (0·867–1·58)	4·21 (3·34–5·01)
South Africa	15 to 95 plus	73·6 (69·5–87·0)	26·4 (13·0–30·5)	100 (100–100)
Zimbabwe	15 to 39	64·5 (58·2–83·2)	9·29 (0·483–12·6)	73·8 (68·7–84·0)
Zimbabwe	40 to 64	21·0 (13·1–24·6)	2·73 (1·36–4·04)	23·8 (14·4–28·1)
Zimbabwe	65 plus	2·02 (1·00–2·79)	0·401 (0·168–0·680)	2·42 (1·22–3·39)
Zimbabwe	15 to 95 plus	87·6 (83·8–97·7)	12·4 (2·29–16·2)	100 (100–100)
Western Sub-Saharan Africa	15 to 39	51·1 (44·9–57·6)	20·0 (14·8–25·5)	71·0 (67·1–75·5)
Western Sub-Saharan Africa	40 to 64	18·2 (15·9–20·5)	7·41 (5·73–8·93)	25·6 (22·0–28·9)
Western Sub-Saharan Africa	65 plus	2·50 (1·84–3·07)	0·861 (0·493–1·19)	3·37 (2·35–4·21)
Western Sub-Saharan Africa	15 to 95 plus	71·8 (65·8–77·7)	28·2 (22·3–34·2)	100 (100–100)
Benin	15 to 39	53·5 (43·1–65·9)	23·5 (14·6–32·8)	77·0 (71·4–83·9)
Benin	40 to 64	15·0 (11·4–18·5)	6·42 (3·68–9·13)	21·4 (15·2–26·3)
Benin	65 plus	1·29 (0·599–1·86)	0·316 (0·0648–0·634)	1·60 (0·644–2·42)
Benin	15 to 95 plus	69·8 (60·1–80·4)	30·2 (19·6–39·9)	100 (100–100)
Burkina Faso	15 to 39	52·2 (46·8–58·1)	19·6 (15·1–24·3)	71·8 (68·0–76·0)
Burkina Faso	40 to 64	16·8 (14·5–19·2)	6·84 (5·12–8·65)	23·6 (20·4–27·0)
Burkina Faso	65 plus	3·80 (2·75–4·68)	0·723 (0·400–1·09)	4·53 (3·17–5·62)

Burkina Faso	15 to 95 plus	72·8 (67·8–78·1)	27·2 (21·9–32·2)	100 (100–100)
Cabo Verde	15 to 39	60·2 (53·4–69·1)	10·1 (5·69–15·0)	70·3 (65·5–76·7)
Cabo Verde	40 to 64	22·9 (18·9–26·2)	3·93 (2·37–5·68)	26·8 (21·6–30·9)
Cabo Verde	65 plus	2·52 (1·45–3·33)	0·366 (0·0948–0·694)	2·89 (1·59–3·94)
Cabo Verde	15 to 95 plus	85·6 (79·9–91·1)	14·4 (8·94–20·1)	100 (100–100)
Cameroon	15 to 39	48·9 (41·2–58·5)	24·9 (17·0–33·1)	73·8 (69·6–78·7)
Cameroon	40 to 64	16·1 (13·8–18·5)	7·70 (5·49–9·89)	23·8 (19·6–27·3)
Cameroon	65 plus	1·81 (1·21–2·27)	0·652 (0·233–1·06)	2·46 (1·50–3·22)
Cameroon	15 to 95 plus	66·8 (58·2–76·0)	33·2 (24·0–41·8)	100 (100–100)
Chad	15 to 39	54·5 (41·7–75·5)	26·9 (12·3–39·0)	81·3 (75·1–90·1)
Chad	40 to 64	11·7 (7·44–15·1)	5·41 (1·86–8·59)	17·2 (9·43–22·5)
Chad	65 plus	1·21 (0·335–1·95)	0·299 (0·0208–0·683)	1·51 (0·366–2·58)
Chad	15 to 95 plus	67·4 (55·2–85·4)	32·6 (14·6–44·8)	100 (100–100)
Côte d'Ivoire	15 to 39	54·3 (47·1–62·6)	17·9 (11·8–24·2)	72·2 (68·1–77·3)
Côte d'Ivoire	40 to 64	19·4 (16·9–22·0)	5·45 (3·36–7·47)	24·9 (20·8–28·6)
Côte d'Ivoire	65 plus	2·28 (1·49–2·89)	0·582 (0·186–0·977)	2·86 (1·75–3·70)
Côte d'Ivoire	15 to 95 plus	76·0 (68·6–83·4)	24·0 (16·6–31·4)	100 (100–100)
Gambia	15 to 39	56·4 (47·8–67·4)	19·1 (11·9–27·3)	75·5 (70·2–82·1)
Gambia	40 to 64	16·8 (13·1–20·1)	5·19 (3·04–7·19)	22·0 (16·6–26·4)
Gambia	65 plus	2·05 (1·04–2·86)	0·511 (0·132–0·945)	2·56 (1·16–3·71)
Gambia	15 to 95 plus	75·2 (66·6–84·2)	24·8 (15·8–33·4)	100 (100–100)
Ghana	15 to 39	49·4 (40·5–61·2)	22·4 (13·2–31·5)	71·8 (66·6–78·2)
Ghana	40 to 64	18·8 (15·5–22·0)	6·70 (4·11–9·33)	25·5 (20·1–29·9)
Ghana	65 plus	2·15 (1·24–2·87)	0·579 (0·138–1·06)	2·73 (1·39–3·85)
Ghana	15 to 95 plus	70·3 (60·6–81·9)	29·7 (18·1–39·4)	100 (100–100)
Guinea	15 to 39	72·4 (61·8–84·6)	5·27 (1·89–9·98)	77·7 (69·4–87·8)
Guinea	40 to 64	18·5 (10·5–25·0)	1·42 (0·604–2·34)	19·9 (11·3–27·2)
Guinea	65 plus	2·31 (0·688–3·95)	0·122 (0·0145–0·292)	2·43 (0·702–4·17)
Guinea	15 to 95 plus	93·2 (88·0–97·1)	6·82 (2·92–12·0)	100 (100–100)
Guinea-Bissau	15 to 39	52·0 (43·9–62·1)	23·1 (15·1–30·8)	75·0 (70·3–80·5)
Guinea-Bissau	40 to 64	15·7 (12·7–18·4)	7·29 (4·86–9·60)	23·0 (18·3–27·3)

Guinea-Bissau	65 plus	1·55 (0·946–2·08)	0·462 (0·158–0·807)	2·01 (1·13–2·77)
Guinea-Bissau	15 to 95 plus	69·2 (60·5–78·6)	30·8 (21·4–39·5)	100 (100–100)
Liberia	15 to 39	52·6 (46·4–60·4)	22·2 (17·1–28·1)	74·8 (70·2–80·3)
Liberia	40 to 64	16·5 (13·0–19·3)	6·61 (5·01–8·33)	23·2 (18·5–27·2)
Liberia	65 plus	1·58 (0·876–2·14)	0·479 (0·234–0·755)	2·05 (1·15–2·81)
Liberia	15 to 95 plus	70·7 (65·1–76·3)	29·3 (23·7–34·9)	100 (100–100)
Mali	15 to 39	52·3 (44·9–61·0)	17·7 (12·3–24·2)	70·0 (64·5–76·4)
Mali	40 to 64	19·0 (15·3–22·7)	6·04 (4·19–8·35)	25·1 (20·1–29·7)
Mali	65 plus	3·93 (2·45–5·35)	0·965 (0·445–1·52)	4·89 (2·95–6·57)
Mali	15 to 95 plus	75·3 (68·7–81·6)	24·7 (18·4–31·3)	100 (100–100)
Mauritania	15 to 39	83·9 (0–100)	8·97 (0–41·1)	92·9 (0–100)
Mauritania	40 to 64	0·255 (0–5·47)	0·0706 (0–0·00494)	0·326 (0–5·84)
Mauritania	65 plus	0 (0–0)	0 (0–0)	0 (0–0)
Mauritania	15 to 95 plus	84·2 (0–100)	9·04 (0–41·8)	93·2 (0–100)
Niger	15 to 39	75·7 (52·9–94·6)	16·2 (2·84–33·2)	92·0 (81·8–98·9)
Niger	40 to 64	5·81 (0·697–13·2)	1·97 (0·287–4·98)	7·78 (1·06–17·6)
Niger	65 plus	0·214 (0·00432–0·724)	0·0453 (0·0000884–0·196)	0·260 (0·00492–0·898)
Niger	15 to 95 plus	81·8 (63·7–96·6)	18·2 (3·41–36·3)	100 (100–100)
Nigeria	15 to 39	47·8 (42·9–53·4)	18·2 (14·0–22·6)	66·0 (62·1–70·0)
Nigeria	40 to 64	20·5 (18·0–23·0)	9·06 (7·02–11·3)	29·5 (26·1–32·9)
Nigeria	65 plus	3·17 (2·44–3·85)	1·29 (0·809–1·84)	4·46 (3·35–5·48)
Nigeria	15 to 95 plus	71·5 (66·6–76·4)	28·5 (23·6–33·4)	100 (100–100)
Sao Tome and Principe	15 to 39	50·4 (44·0–58·5)	16·6 (11·3–22·5)	67·0 (62·3–72·4)
Sao Tome and Principe	40 to 64	22·3 (19·5–25·5)	7·05 (4·82–9·30)	29·4 (25·1–33·3)
Sao Tome and Principe	65 plus	2·78 (1·89–3·49)	0·808 (0·313–1·34)	3·58 (2·23–4·60)
Sao Tome and Principe	15 to 95 plus	75·5 (69·1–81·8)	24·5 (18·2–30·9)	100 (100–100)
Senegal	15 to 39	73·1 (52·8–92·2)	15·2 (3·77–28·6)	88·3 (77·5–97·2)
Senegal	40 to 64	8·55 (1·90–15·9)	2·62 (0·486–5·68)	11·2 (2·80–21·4)
Senegal	65 plus	0·462 (0·0227–1·23)	0·0909 (0·000792–0·351)	0·553 (0·0238–1·58)
Senegal	15 to 95 plus	82·1 (66·8–95·3)	17·9 (4·68–33·2)	100 (100–100)
Sierra Leone	15 to 39	52·8 (47·1–59·3)	14·0 (10·1–18·7)	66·7 (62·6–71·5)

Sierra Leone	40 to 64	23·3 (20·2–26·4)	6·30 (4·64–8·22)	29·6 (25·6–33·3)
Sierra Leone	65 plus	3·02 (2·19–3·84)	0·659 (0·342–1·03)	3·68 (2·61–4·70)
Sierra Leone	15 to 95 plus	79·1 (73·9–83·6)	20·9 (16·4–26·1)	100 (100–100)
Togo	15 to 39	48·9 (38·8–61·2)	26·2 (17·3–34·9)	75·0 (69·2–82·1)
Togo	40 to 64	15·3 (11·4–18·5)	8·05 (5·22–10·9)	23·3 (16·9–28·7)
Togo	65 plus	1·12 (0·555–1·67)	0·527 (0·164–0·946)	1·65 (0·740–2·52)
Togo	15 to 95 plus	65·2 (55·9–75·1)	34·8 (24·9–44·1)	100 (100–100)
